# Coach behaviors, coach–athlete relationships, and competitive psychological processes in elite sport: a temporal narrative synthesis

**DOI:** 10.3389/fpsyg.2026.1969318

**Published:** 2026-09-29

**Authors:** Oğuzhan Gül, Turhan Toros, Mehmet Gül, Mehmet Emre Eryücel, Cihan Bülbül, Mehmet Bayansalduz

**Affiliations:** 1 Department of Health, Culture and Sports, Sivas Cumhuriyet University, Sivas, Türkiye; 2 Department of Coaching Education, Faculty of Sports Sciences, Mersin University, Mersin, Türkiye; 3 Department of Sports Management, Faculty of Sports Sciences, Sivas Cumhuriyet University, Sivas, Türkiye; 4 Department of Sports Management, Faculty of Sports Sciences, Akdeniz University, Antalya, Türkiye; 5 Department of Sport Management, Faculty of Sport Sciences, İstanbul Topkapı University, İstanbul, Türkiye

**Keywords:** coach-athlete relationship, coaching behavior, competitive psychological processes, elite sport, seasonal dynamics

## Abstract

**Background:**

Research on coach behavior, coach–athlete relationships, and competitive psychological processes has developed in partly separate literatures, limiting understanding of how these domains interact across an elite competitive cycle.

**Aims:**

This review examined how these domains are represented across foundation building, competitive maintenance, peak competition, and post-competition transition, while distinguishing direct temporal evidence from interpretive phase placement.

**Methods:**

Web of Science, SPORTDiscus, and Scopus were searched for peer-reviewed English-language studies published from January 2016 to 22 June 2026. Of 645 records identified, 481 unique records were screened, 205 underwent eligibility assessment, and 30 empirical studies were included.

**Results:**

Evidence was strongest for competitive maintenance and peak competition. Repeated-measures, diary, prospective, and event-based studies linked coaching behavior and relationship-related judgments with motivation, well-being, perceived justice, burnout, appraisal, coping, affect, confidence, and perceived performance. Evidence for foundation building was limited, while direct evidence on post-season evaluation and cross-cycle carryover was largely absent.

**Conclusion:**

The proposed temporal framework should be viewed as an evidence-informed research architecture rather than an established causal sequence. It highlights phase-specific hypotheses for future research, coach education, and psychological support.

## Highlights

- Align communication, autonomy support, and feedback with the competitive phase, emphasizing expectation setting early, ongoing explanation during the season, and concise, task-focused support around major events.
- Use brief, repeated monitoring of relationship quality, perceived justice, athlete well-being, and coach recovery to detect short-term changes that one-off assessments may miss.
- Build structured post-competition debriefs to review roles, repair relationships, and support continuity into the next cycle, while recognizing that direct evidence for this phase remains limited.

## 1 Introduction

Coaching psychology in elite sport has been mainly relying on three mature but incompletely connected bodies of research. One examines the behaviors and leadership practices through which coaches create motivational climates, provide feedback, and support or frustrate basic psychological needs (Mageau and Vallerand, 2003; Côté and Gilbert, 2009). A second focuses on the relationship between coach and athlete, commonly described through closeness, commitment, complementarity, and co-orientation (Jowett, 2007; Jowett and Shanmugam, 2016). A third addresses competitive psychological processes, including anxiety, appraisal, coping, emotion regulation, confidence, and readiness under pressure (Hanton et al., 2008; Mellalieu et al., 2009). These literatures overlap in practice but are often reviewed and measured as parallel domains.

Elite sport provides a demanding setting in which their interaction may be especially consequential. Selection deadlines, compressed performance cycles, public scrutiny, organizational pressure, and unequal decision-making authority can alter both the meaning of coaching behavior and the athlete’s appraisal of it (Nesti, 2024; Henriksen et al., 2025). For example, in weight-class sports, competition-phase demands such as rapid weight loss may intensify physiological and psychological strain, including anxiety responses, and thereby shape how athletes appraise coach communication and competitive pressure (Sarıakçalı et al., 2025). A supportive action early in a season may contribute to trust and autonomous motivation, whereas a similar degree of direction immediately before a championship may be experienced as necessary structure. Controlling behavior or unclear communication, by contrast, can accumulate across repeated training and selection decisions. Single-time-point designs cannot establish how such patterns originate, change, or carry forward.

Recent work in sport and exercise psychology has also widened from a predominantly performance-centered orientation toward mental health, well-being, and development (Hidayat et al., 2026). A temporal lens does not require these priorities to be treated as competing. Need satisfaction, relational security, and sustainable motivation may be especially visible during foundation and maintenance periods; coping, confidence, appraisal, and emotional control become more prominent around major competition. What remains uncertain is whether the available evidence supports a linked seasonal sequence or only a set of phase-compatible associations.

To our knowledge, previous reviews have not systematically organized these three domains around a shared seasonal timeline while separating direct temporal evidence from conceptual phase assignment in elite sport. The present review therefore addressed three questions:

1. How are coach behaviors, coach–athlete relationship quality, and competitive psychological processes represented across distinct phases of an elite competitive cycle?
2. Which theoretical frameworks have direct empirical support within each phase, and where does phase mapping remain interpretive?
3. How do sport structure, organizational context, culture, gender, and relational disruption modify the proposed temporal pattern?

## 2 Method

### 2.1 Review design

The review combined a systematic database search and transparent two-stage screening process with a structured narrative synthesis. It was designed to integrate heterogeneous quantitative, qualitative, mixed-methods, and intervention evidence rather than estimate a pooled effect. PRISMA 2020 items were used to report record identification and selection (Page et al., 2021), while the interpretive synthesis followed the logic of a narrative review (Grant and Booth, 2009). No review protocol was prospectively registered for this study.

### 2.2 Search strategy and deduplication

Web of Science (WoS), SPORTDiscus via EBSCOhost, and Scopus were searched on 22 June 2026. The search was restricted to publications from January 2016 onward. Foundational theoretical contributions predate that boundary and were incorporated through citation chaining rather than the database search. The 10-year window was selected because repeated-measures, diary, and prospective designs in elite coaching research became common enough during this period to support a phase-specific synthesis; earlier literature, while valuable for conceptual grounding, offered fewer studies with the temporal resolution the review required. Three search strings targeted (S1) coaching behavior and relationship processes in elite sport, (S2) competitive psychological processes in coaching contexts, and (S3) temporal or seasonal dynamics. Full Boolean expressions, field codes, filters, and database-specific yields are reported in Supplementary Table S2.

The searches returned 645 records: 407 from Web of Science, 75 from SPORTDiscus, and 163 from Scopus. The Web of Science and SPORTDiscus exports were merged and deduplicated, yielding 417 unique records. Within Scopus, five records were duplicated across the three search strings, leaving 158 unique Scopus records. Ninety-four of these matched the Web of Science/SPORTDiscus master: 91 by exact normalized title and three after manual comparison of punctuation and typographic variants. The remaining 64 Scopus-only records were added, producing 481 unique records. The complete match log and final master are supplied in Supplementary Data File 1 (Table 1).

**Table 1:** Database yields and study flow.

Stage	WoS	SPORTDiscus	Scopus	Combined	Notes
Records retrieved	407	75	163	645	Final filtered yields
Unique Scopus records			158		Five internal Scopus duplicates
Unique WoS/SPORTDiscus master				417	Existing deduplicated master
Scopus overlap with master			94		91 exact; 3 manually verified fuzzy matches
Scopus-only records			64		Added to screening master
Unique records screened				481	
Excluded at title/abstract				276	173 construct; 71 population; 32 off-topic
Eligibility assessments				205	Full text or extended abstract
Excluded at eligibility				175	Article-level reasons in Supplementary Data File 1
Included in synthesis				30	21 core; 9 contextual

### 2.3 Eligibility criteria and evidence tiers

Eligible reports were peer-reviewed English-language articles published from January 2016 through 22 June 2026. Samples had to include elite, professional, national-level, international-level, or clearly defined high-performance athletes or coaches. Elite adolescents were retained when participation at national level or above was explicit. Original quantitative, qualitative, mixed-methods, and intervention studies were eligible. Reviews were used for background or citation chaining but were not counted among the 30 empirical studies.

The synthesis used two evidence tiers. Core studies examined at least two of three pillars: (P1) coach behavior or leadership, (P2) coach–athlete relationship or directly relevant interpersonal processes, and (P3) competitive psychological processes. Contextual studies could address one pillar when they supplied direct temporal evidence for that track or informed an organizational, cultural, gender, or sport-structure moderator. Studies were excluded when elite status could not be confirmed, fewer than two pillars were represented without a clear contextual role, temporal or phase relevance was absent, or the focus was predominantly physiological, technical, epidemiological, or technological.

Injury studies that did not measure coaching or relational processes were not counted in the empirical synthesis. Injury is retained in the model as a cross-cutting proposition that requires direct testing rather than as an evidence-supported seasonal phase.

### 2.4 Screening and verification

Title and abstract screening and eligibility assessment were completed by the review team using the predefined criteria; Figure 1 presents the complete flow of records from identification through inclusion. Dr. Oğuzhan Gül and Dr. Turhan Toros then reviewed the completed eligibility decisions and the article-level exclusion reasons for consistency with those criteria. Discrepancies were flagged when an eligibility classification or an exclusion reason differed between the initial coders and the reviewers; flagged cases were discussed among three team members and resolved through consensus before the final inclusion list was confirmed. Because this external check took the form of a review of completed decisions rather than duplicate screening of every record, an inter-rater agreement statistic was not calculated. Supplementary Data File 1 supplies all 481 screened records and the 175 eligibility exclusions with article-level reasons.

**Figure 1:**
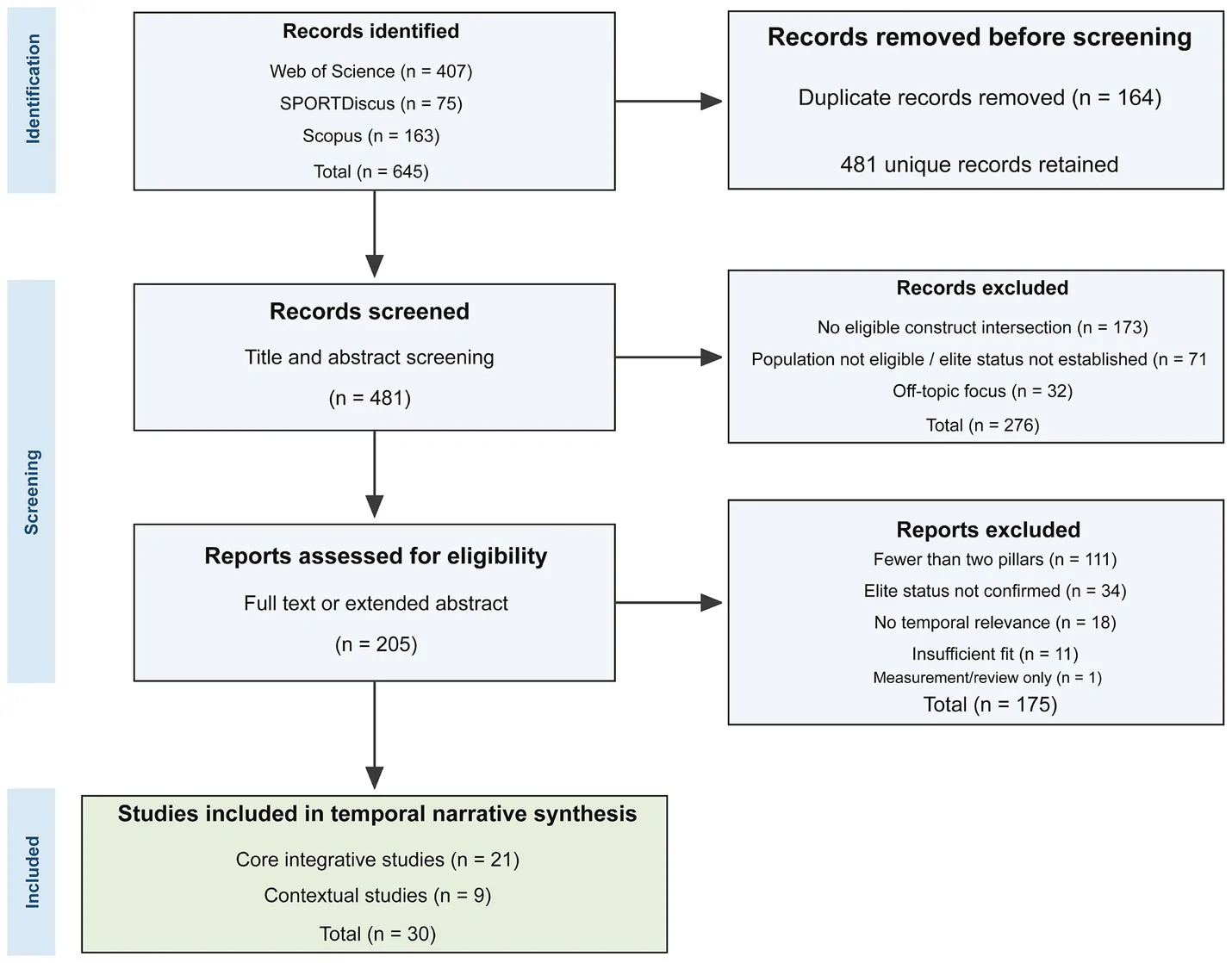
Flow of records through identification, title/abstract screening, eligibility assessment, and inclusion, following PRISMA 2020 reporting guidance (Page et al., 2021).

### 2.5 Data charting, phase coding, and evidence appraisal

For each included study, we charted authorship, year, sport and population, design, construct pillars, temporal structure, primary phase, theoretical framework, and findings relevant to the synthesis. Instruments relevant to each pillar are summarized in Supplementary Table S1. Phase I comprised pre-season or early-season climate setting, selection, role definition, and initial relationship formation. Phase II comprised regular-season maintenance, including feedback, autonomy calibration, conflict, selection uncertainty, fatigue, and motivational continuity. Phase III comprised immediate precompetition, competition, tournaments, championships, and major events. Phase IV was restricted to evidence explicitly collected after the final event or during the off-season and concerning evaluation, recovery, relationship continuation or dissolution, or preparation for the next cycle.

Two additional codes prevented temporal overreach. Relational aftermath captured long-term rupture, abuse, deselection, and career carryover not tied to a defined post-season window. Cross-cutting captured organizational, cultural, gender, sport-structure, and person-level moderators. Phase evidence was coded as direct when timing was measured or explicitly described in the design and as indirect when placement was based on conceptual compatibility. OG and TT completed the initial phase coding using the operational guide; MG and MEE reviewed the assignments and evidence classifications. Differences identified during review were discussed and resolved by consensus. The full coding guide is supplied in Supplementary Table S3 and Supplementary Data File 1.

Studies were also assigned an evidence-weight rating developed specifically for this review. Because the included studies span quantitative, qualitative, mixed-methods, and intervention designs, no single validated risk-of-bias instrument covers the full set. A bespoke five-domain scheme was therefore constructed to appraise each study’s contribution to the temporal synthesis rather than against a generic quality hierarchy. The five domains were selected because each captures a dimension along which a study’s relevance to a phase-specific coaching synthesis varies: population relevance (how closely the sample matched elite or high-performance criteria), construct integration (whether the study addressed more than one of the review’s three pillars), temporal alignment (whether timing was measured, designed into the study, or assigned by interpretive fit), design strength (prospective, repeated-measures, or intervention designs scored higher than cross-sectional or retrospective designs), and reporting adequacy (completeness of methodological and statistical detail). Each domain was scored on a three-point scale (0, 1, 2), yielding totals from 0 to 10. Scores of 8–10 were classified as high evidence weight and scores of 5–7 as moderate. The thresholds were set at the scale midpoint with a buffer favoring the moderate category, so that studies with strong population and design credentials but limited temporal alignment were not excluded from the interpretive synthesis. The appraisal informed the weight given to individual findings in the narrative but was not used as an automatic exclusion rule, and it should not be interpreted as a conventional risk-of-bias instrument. Study-level ratings are reported in Supplementary Table S4, and the full scoring definitions are available in Supplementary Data File 1.

## 3 Results

### 3.1 Study selection and evidence profile

Thirty empirical studies were retained: 21 core studies integrating at least two construct pillars and nine contextual studies informing one temporal track or a cross-cutting moderator. Seventeen studies provided direct phase-relevant evidence through prospective, repeated-measures, diary, intervention, event-based, or explicitly phase-focused qualitative designs; 13 supported phase placements indirectly. The evidence-weight appraisal classified 17 studies as high and 13 as moderate (Table 2). Evidence was concentrated in Phases II and III. Three studies were assigned primarily to Phase I, of which two supplied direct prospective evidence and one was interpretive. No included study directly followed coach–athlete dyads from the end of a season through the off-season into the next preparatory cycle.

**Table 2:** Included empirical studies, evidence tier, temporal placement, and principal contribution.

Study	Sport/population	Design	Tier	Pillars	Primary placement	Evidence	Principal finding
Fryer et al., 2026	Multiple Olympic/Paralympic sports; elite coaches	Qualitative interviews	Core	P1; P3	Phase III	Direct	Elite coaches described deliberate mental preparation, attentional control, and emotional regulation before and during major competitions.
Ferguson and Mason, 2026	Olympic swimming	Qualitative interviews	Core	P1; P2	Cross-cutting	Indirect	Olympic swimmers emphasized trust, psychological safety, explanation of decisions, and collaborative leadership within an individual-performance/team-training structure.
Braga-Pereira et al., 2026	National-team coaches; major international competitions	Qualitative interviews	Core	P2; P3	Phase III	Direct	National-team coaches described relationship management as an active task during major competitions, with practices adjusted to athletes and event demands.
Davis et al., 2026	Multiple team sports	Nonrandomized cluster-controlled mixed methods	Core	P1; P2	Phase II	Direct	A relationship intervention produced no significant improvements in the principal quantitative outcomes, although participants viewed parts of the program as relevant and feasible.
Henriksen et al., 2025	Danish Olympic/Paralympic programs	Multiple-case qualitative study	Core	P1; P2	Phase I	Indirect	High-performance coaches were described as architects who balance clear structures and values with individual consideration, athlete responsibility, and sustainable development.
Ulaş et al., 2025	Turkish top-league football	Qualitative thematic analysis	Core	P1; P2; P3	Phase II / relational aftermath	Indirect	Players linked non-selection to coach preferences and unclear communication; these attributions were associated with exclusion, reduced motivation, diminished self-worth, and strained relationships.
González-García et al., 2024	Multiple competitive sports	Three-point prospective design	Core	P2; P3	Phase III	Direct	Pre-competition relationship quality was associated with coping, affective states, satisfaction, and goal attainment through statistically indirect pathways.
Karayel et al., 2024	Elite Turkish football	Cross-sectional correlational study	Core	P1; P2; P3	Phase II	Indirect	Coach–athlete relationship quality was associated with team resilience, with transformational leadership functioning as a statistical mediator in a cross-sectional model.
Slade et al., 2024	United Kingdom high-performance sport	Delphi study	Core	P1; P2	Selection/transition	Indirect	A Delphi panel generated consensus guidelines for athletes, coaches, and organizations to improve selection and deselection processes.
Cece et al., 2022	Elite table tennis centers	Longitudinal field intervention	Core	P1; P3	Phase II	Direct	A need-supportive intervention during the competitive season was accompanied by improved trajectories in autonomy-related needs and several emotional outcomes.
Robazza et al., 2022	Individual and team sports	Cross-sectional SEM	Core	P1; P3	Phase II	Indirect	Perceived mastery climates were associated with cognitive reappraisal and more pleasant psychobiosocial experiences; performance climates were associated with suppression and unpleasant emotions.
da Silva et al., 2022	Under-20 football; transition to professional	Prospective beginning-to-end season study	Core	P2; P3	Phase II	Direct	Across a season, dimensions of relationship quality and coping were associated with later stress, anxiety, self-confidence, burnout, and depressive symptoms in under-20 players transitioning toward professional football.
Kim and Park, 2020	Korean Olympic archery	Qualitative interviews	Core	P1; P2; P3	Phase III	Direct	Olympic archers described communication that varied by context: instructional during training, concise and task-focused during competition, and emotionally supportive during crises.
Moreira da Silva et al., 2021	Olympic shooting	Cross-sectional elite-athlete self-report	Core	P1; P3	Phase III	Indirect	Elite shooters identified confidence, anxiety regulation, psychological preparation, and coach support as important components of Olympic shooting performance.
Miller et al., 2020	Amateur and professional team/individual sports	Cross-sectional plus start/end-season prospective study	Core	P1; P2; P3	Phase III	Direct	Coach identity leadership was associated with adaptive resource appraisals; in the prospective component, early-season identity leadership was linked to later self-efficacy through relational identification.
Balk et al., 2019	Multiple elite sports	Daily diary study	Core	P1; P3	Phase II	Direct	Day-level variation in coaches’ recovery was associated with athletes’ perceptions of autonomy support and performance satisfaction.
Cheval et al., 2017	Elite youth football academy	Three-wave repeated-measures study	Core	P1; P3	Phase II	Direct	Within-person changes in perceived autonomy support and controlling behavior covaried with changes in vitality, self-esteem, and burnout over the final months of the season.
Nicholls et al., 2017	Premier League academy football	Two-wave six-month longitudinal study	Core	P2; P3	Phase I	Direct	Relationship quality at the first assessment was prospectively associated with stronger mastery-goal attainment 6 months later.
Ntoumanis et al., 2017	Multiple competitive sports	Prospective study	Core	P1; P3	Phase I	Direct	Coach interpersonal style was prospectively associated with athletes’ doping-related cognitions and behavior, with moral disengagement implicated in the pathway.
Kavanagh et al., 2017	Multiple elite sports	Retrospective qualitative study	Core	P2; P3	Relational aftermath	Indirect	Elite athletes described coping and sense-making processes following emotional abuse in coach–athlete relationships; the evidence concerns relational aftermath rather than a defined post-season period.
De Backer et al., 2018	Elite handball and volleyball	Six-week repeated-game longitudinal study	Core	P1; P2	Phase II/III	Direct	Perceived justice fluctuated from game to game; need-supportive coaching and decision justifications were associated with greater justice, whereas substitute status and losses were associated with lower justice.
Schlawe et al., 2026	High-performance sport environments	Qualitative stakeholder study	Contextual	P1; P2	Cross-cutting	Indirect	Stakeholders identified environmental features that support performance and welfare, emphasizing organizational conditions beyond the dyad.
Tjønndal and Haugen, 2024	China-Norway winter-sport collaboration	Qualitative interviews	Contextual	P1; P2	Cross-cutting	Indirect	Norwegian coaches described care as a way to navigate cultural, bureaucratic, and political tensions in a China-Norway Olympic collaboration.
Levillain et al., 2025	Multiple sports and competitive levels	Five-competition longitudinal study	Contextual	P3	Phase III	Direct	Appraisals, coping, emotions, and perceived performance varied across five competitions; emotional intelligence moderated several within-season associations.
Levillain et al., 2022	Multiple competitive sports	Three-wave seasonal longitudinal study	Contextual	P3	Phases II–III	Direct	Athletes followed heterogeneous emotional trajectories across the beginning, middle, and end of a competitive season.
Verner-Filion and Vallerand, 2018	Elite youth football	Multi-season longitudinal study	Contextual	P3	Phase II	Direct	Passion and basic-need satisfaction showed longitudinal associations with preparation, well-being, and performance-related functioning in elite youth football.
Dehghansai et al., 2020	Olympic and Paralympic coaching	Qualitative coach-perspective study	Contextual	P1	Cross-cutting	Indirect	Olympic and Paralympic coaches emphasized adaptable skill-acquisition support, specialist collaboration, and continuing coach development.
Poucher et al., 2018	Female Olympians and support providers	Paired qualitative interviews	Contextual	P2	Cross-cutting	Indirect	Female Olympians and their main support providers described support as necessary but also demanding, with high levels of interpersonal dependence.
Kegelaers and Wylleman, 2019	Multiple elite sports	Qualitative study	Contextual	P1; P3	Cross-cutting	Indirect	Elite coaches described proactive and reactive strategies for developing athlete resilience.
Carleton et al., 2016	National Basketball Association	Longitudinal archival multilevel study	Contextual	P1; P3	Relational aftermath	Direct	Archival longitudinal analyses associated exposure to abusive leadership with career trajectories of player aggression and task performance.

### 3.2 Theoretical frameworks: supported uses and propositions

No single theory reviewed here spans the full arc from early-season climate formation to post-season relationship evaluation. The five retained frameworks instead illuminate different parts of the evidence. Their placement in the temporal framework does not establish that a particular theory governs a particular phase; it identifies where its constructs have been measured most directly and where their use remains interpretive.

Self-Determination Theory (SDT; Deci and Ryan, 2000) addresses how interpersonal environments satisfy or frustrate athletes’ needs for autonomy, competence, and relatedness. In the included evidence, SDT had its clearest direct support during competitive maintenance. Repeated-measures, diary, and intervention studies linked autonomy support, controlling behavior, need satisfaction, and motivation with changing athlete outcomes across the active season (Cheval et al., 2017; Balk et al., 2019; Cece et al., 2022). Achievement Goal Theory (AGT; Nicholls, 1989) directs attention to the evaluative climate created around mastery, improvement, and normative comparison. Its temporal placement was less direct: Nicholls et al. (2017) supplied prospective evidence concerning relationship quality and later mastery-goal attainment, whereas Robazza et al. (2022) linked motivational climate with emotion regulation in a cross-sectional design.

Jowett’s 3 + 1Cs model (Jowett, 2007) places the dyadic relationship at the center of analysis through closeness, commitment, complementarity, and co-orientation. It was represented most clearly in studies that measured relationship quality before or around competition or examined relationship processes directly (Davis et al., 2026; González-García et al., 2024; Braga-Pereira et al., 2026). Smith and Smoll’s Mediational Model (Smith et al., 1978; Smoll and Smith, 1989) offers a conceptual sequence in which coach behavior is interpreted by the athlete and followed by evaluative or behavioral responses. Recent evidence from athlete samples also supports this behavior–perception–response pathway, indicating that positive coach behaviors may contribute to athletes’ self-efficacy (Öztürk et al., 2026). The sequence is useful for reading event-specific communication and later sense-making, but contemporary elite studies rarely tested the full sequence directly.

Transformational and identity leadership theories add group-level processes, including collective identification, resilience, efficacy, and resource appraisal (Miller et al., 2020; Karayel et al., 2024). Their relevance is strongest in team settings and is less certain in individual sports or off-season transitions. Table 3 therefore distinguishes the best-supported use of each framework from interpretive extensions and boundary conditions rather than assigning one definitive theory to each phase.

**Table 3:** Theory-to-phase mapping with evidence status and boundaries.

Framework	Best-supported phase or use	Representative evidence	Interpretive extension or boundary
Self-Determination Theory	Phase II	Cheval et al. (2017); Balk et al. (2019); Cece et al. (2022)	Phase I climate formation is plausible but less often measured prospectively.
Achievement Goal Theory	Phases I–II, mainly indirect	Nicholls et al. (2017); Robazza et al. (2022)	Competition-appraisal effects are proposed more often than directly tested.
3 + 1Cs model	Phases II–III	Davis et al. (2026); González-García et al. (2024); Braga-Pereira et al. (2026)	Phase IV relationship evaluation, continuation, and dissolution remain largely untested.
Mediational Model	Phase III as an interpretive lens	Kim and Park (2020); Kavanagh et al. (2017)	The full behavior–perception–response sequence was not directly tested in most elite studies.
Transformational/identity leadership	Phases II–III in team contexts	Miller et al. (2020); Karayel et al. (2024)	Application to individual sports and off-season transitions remains uncertain.

### 3.3 Phase I: foundation building

Before competitive fixtures begin, coaches often face formative tasks: establishing a motivational climate, setting behavioral expectations, selecting or confirming squad members, and developing the trust needed to sustain a working relationship. Evidence from elite settings suggests that relationship quality and interpersonal style measured early in a competitive cycle may be associated with motivational and behavioral outcomes observed later. Direct evidence for the distinct pre-season period, however, was limited.

Nicholls et al. (2017), in a two-wave longitudinal study of Premier League academy footballers, found that perceptions of the coach–athlete relationship at the initial assessment were prospectively associated with mastery-goal attainment 6 months later. The temporal ordering is consistent with the possibility that early relationship quality contributes to the standards through which athletes interpret effort and improvement. The study did not isolate pre-season as the period in which this climate was formed. Ntoumanis et al. (2017) similarly showed that coach interpersonal style measured earlier in the competitive cycle was prospectively associated with later doping-related cognitions and behavior, with moral disengagement implicated in the statistical pathway. The design supports temporal ordering but not a causal effect of an early-season climate.

Henriksen et al. (2025) provided an interpretive account of foundation building in Danish Olympic and Paralympic programs. Coaches described organizing high-performance environments around clear values, stable structures, whole-person leadership, athlete responsibility, and the balance between competitive demands and sustainable development. These findings support the importance of environmental design, but they do not establish that such principles are created only before a season or that their effects differ by phase. The practical form of this work may also vary by sport structure: individual-sport coaches work more directly within the dyad, whereas team-sport coaches must coordinate collective climate and individual relationship quality.

Selection and deselection are further relational events that may occur during foundation building or at other transition points. Slade et al. (2024) used a Delphi process to identify communicative, procedural, educational, and supportive practices for managing these decisions in high-performance sport. The study generated consensus guidance rather than direct evidence of athletes’ lived relational rupture or its timing. It nevertheless indicates that clarity, explanation, and support may matter when squad membership and competitive opportunity are decided, and that these processes involve organizations as well as the coach–athlete dyad.

Theoretical links from Phase I to later competitive psychological processes remain plausible but only partly tested. AGT suggests that mastery-oriented climates may direct attention toward effort, learning, and controllable standards, whereas performance-oriented climates may heighten social comparison. Robazza et al. (2022) found cross-sectional associations between mastery climate, cognitive reappraisal, and more pleasant psychobiosocial experiences, while performance climate was associated with suppression and unpleasant experiences. Because timing was not tested, the study cannot show that a climate formed in Phase I produced later emotional responses. SDT provides a parallel proposition in which need-supportive coaching may strengthen autonomous motivation and resources for later demands. Overall, early relationship quality and interpersonal style can precede later outcomes, but the specific effects of pre-season camps, squad formation, and expectation setting remain underexamined.

### 3.4 Phase II: competitive maintenance

Once the competitive season is underway, coaching tasks shift from construction to continuing calibration. Coaches must sustain motivational climates under result pressure, adjust feedback and autonomy support, explain decisions, and maintain relationship quality through conflict, selection uncertainty, fatigue, and changing athlete needs. Phase II contained the strongest direct temporal evidence in the review.

Cheval et al. (2017) provided within-person evidence across three assessments during the final months of an elite football season. Changes in perceived autonomy support and controlling behavior covaried with changes in vitality, self-esteem, and burnout. Balk et al. (2019) extended this temporal resolution to the daily level: variation in coaches’ off-job recovery was associated with athletes’ perceptions of autonomy support and performance satisfaction. Coaching behavior and athlete experience can vary within the same people across relatively short periods, not only between coaches or athletes.

Intervention findings were less uniform. Cece et al. (2022) reported improved trajectories in autonomy-related needs and several motivational and emotional outcomes during a need-supportive intervention delivered in elite table tennis centers; the absence of a separate control condition limits causal interpretation. Davis et al. (2026), using a nonrandomized cluster-controlled mixed-methods design, found no significant improvement in the principal quantitative outcomes of a coach–athlete relationship program, although participants considered parts of the intervention useful and feasible. Motivational and relationship outcomes may respond differently to brief programs, but the studies do not establish that established relationships are generally resistant to change.

Relationship-related judgments also shifted within active competition. Across 6 weeks of repeated games, De Backer et al. (2018) found that perceived justice varied from game to game among elite handball and volleyball players. Need-supportive coaching and explanations for decisions were associated with greater perceived justice, whereas substitute status and losses were associated with lower ratings. Da Silva et al. (2022) followed under-20 footballers from the beginning to the end of a season and found that dimensions of relationship quality and coping were associated with later stress, anxiety, self-confidence, burnout, and depressive symptoms. Relationship maintenance is not a fixed background condition; athletes repeatedly interpret decisions under changing competitive and psychological demands.

Cross-sectional and qualitative findings were compatible with this account but could not show temporal change. Robazza et al. (2022) associated mastery climate with cognitive reappraisal and more pleasant psychobiosocial experiences, and performance climate with suppression and unpleasant emotions. Karayel et al. (2024) reported cross-sectional indirect associations among relationship quality, transformational leadership, and team resilience. Ulaş et al. (2025) found that footballers linked non-selection to coach preferences and unclear communication, with reported consequences for motivation, self-worth, and the relationship. The findings identify processes that may matter during maintenance, but their timing and direction cannot be inferred from the designs alone.

Contextual evidence also shows that athlete functioning changes over longer periods. Verner-Filion and Vallerand (2018) reported longitudinal associations among passion, basic-need satisfaction, preparation, well-being, and performance-related functioning across multiple seasons in elite youth football. The study did not measure coach behavior directly, so it supports the motivational track rather than the full three-pillar framework. Phase II is therefore the best-supported part of the temporal synthesis, although even here only a subset of studies measured coach behavior, relationship quality, and competitive psychological processes together.

### 3.5 Phase III: peak competition

As major events approach, the evidence shifts toward appraisal, coping, communication, confidence, and coach self-regulation. Coaching behavior becomes more event-specific, while relationship quality may serve as a resource or an additional source of strain. The relevant timescale also narrows from months and weeks to defined competitions, rounds, and moments within an event. González-García et al. (2024) offered multi-time-point evidence for this phase. Relationship quality assessed before competition was associated with coping, affective states, satisfaction, and goal attainment through statistical indirect pathways. The ordering of assessments is compatible with a sequential association from precompetition relationship quality to later outcomes. Mediation analysis, however, does not remove residual confounding or establish that the relationship caused the intervening psychological processes.

Qualitative studies supplied event-specific detail from the coaching perspective. Fryer et al. (2026) found that Australian high-performance coaches used structured mental preparation routines to manage their own psychological states before and during major competitions. Braga-Pereira et al. (2026) studied national team coaches at major international championships and found that relational management shifted under championship conditions. Coaches reported consciously adjusting their closeness and complementarity with athletes depending on the stage of the competition and the emotional demands of specific events. During early rounds, coach’s maintained familiar relational patterns; as stakes increased in later rounds and medal events, coaches described moving toward either greater emotional closeness or more directive complementarity, depending on their assessment of what each athlete needed.

Communication also varied by competitive context. Kim and Park (2020) documented that Korean Olympic archers described exchanges as more instructional during training, concise and task-focused during competition, and emotionally supportive during periods of difficulty. Moreira da Silva et al. (2021) reported a similar pattern with Olympic shooters, for whom coach-mediated confidence building in the hours before competition was intertwined with structured anxiety management routines.

Team-level and within-competition evidence further complicates a single account of peak competition. Miller et al. (2020) associated coach identity leadership with adaptive resource appraisals and relational identification; its prospective component linked early-season identity leadership with later self-efficacy. Levillain et al. (2022) identified heterogeneous emotional trajectories across the beginning, middle, and end of a season, while Levillain et al. (2025) documented variation in appraisal, coping, emotion, and perceived performance across five competitions. De Backer et al. (2018) similarly showed game-to-game variation in justice. Phase III should therefore not be treated as one stable psychological state. It contains event-level and within-event variation, and several studies describe only the competitive psychological track rather than directly testing coaching and relationship processes together.

### 3.6 Phase IV: post-competition and off-season transition

No included study directly followed elite coach–athlete dyads from the final competitive event through off-season evaluation and into the next preparatory cycle. Phase IV is therefore an empirical gap, not a phase supported by evidence comparable in volume or quality to that available for Phases II and III. Studies of abuse, deselection, coaching careers, and injury rehabilitation were not used as substitutes for direct post-season evidence. The missing questions are specific. It remains unclear how coaches and athletes review a completed season together, renegotiate trust and role expectations, decide whether the relationship will continue, or adapt when coaching personnel change. Research has also not established how unresolved experiences from one season affect the next cycle. These processes are central to the temporal framework, but they remain propositions requiring prospective cross-cycle research.

### 3.7 Relational aftermath and career carryover

Adjacent evidence indicates that coaching experiences can continue to shape athletes after an immediate interaction or relationship has ended, but these studies should not be labeled post-season research. Kavanagh et al. (2017) documented coping and evolving sense-making following emotional abuse in elite coach–athlete relationships. The retrospective accounts concerned longer-term aftermath rather than a defined off-season window. Carleton et al. (2016) associated exposure to abusive leadership with career trajectories of aggression and task performance in professional basketball. Ulaş et al. (2025) likewise showed that non-selection and inadequate communication could leave motivational and relational residue. These findings justify a separate category of relational aftermath and career carryover. They support the possibility that experiences persist, but they do not provide direct evidence about how ordinary season-end evaluation or off-season relationship renewal unfolds.

### 3.8 Cross-cutting moderators

Sport structure changes how the proposed temporal pattern may operate. In team sports, coaching behavior is interpreted within a collective climate that includes team identification, role clarity, cohesion, and group efficacy (Miller et al., 2020; Karayel et al., 2024). Individual sports place greater weight on the dyad, although training may still occur in a group. Ferguson and Mason (2026) illustrated this hybrid structure in Olympic swimming, where individual performance was embedded in a team training environment and athletes emphasized both individualized trust and group-based psychological safety. Organizational conditions also distribute authority beyond the dyad. Henriksen et al. (2025) and Schlawe et al. (2026) described high-performance systems in which coaches balance performance expectations, values, resources, and responsibility for athlete welfare. Slade et al. (2024) similarly located selection and deselection within a coach–athlete–organization triad. The studies indicate that relational events cannot always be attributed to coach behavior alone.

Cultural and gender norms may alter how the same behavior is interpreted, although the available studies characterize single settings rather than compare phases between contexts. Kim and Park (2020) described context-dependent communication within Korean Olympic archery, while Tjønndal and Haugen (2024) showed how care was used to navigate cultural, bureaucratic, and political tensions in a China–Norway winter-sport collaboration. Poucher et al. (2018) found that support for female Olympians could be both indispensable and demanding, and Kegelaers and Wylleman (2019) described proactive and reactive coaching strategies for resilience. Phase-by-culture and phase-by-gender comparisons are among the more tractable extensions of the framework, since the phase definitions in Supplementary Table S3 apply without modification to these settings.

Athletes and coaches also enter each phase with different psychological and recovery states. Balk et al. (2019) linked day-level coach recovery with athletes’ perceptions of autonomy support. Recent evidence in elite combat athletes also indicates that rapid weight loss before competition may alter psychological resilience and pain sensitivity, suggesting that athlete state variables may moderate phase-specific coach–athlete and performance-related processes (Çağlar et al., 2025). Two injury studies fell outside the empirical synthesis because neither measured coaching or relationship processes, yet both sharpen the person-level proposition: Purnomo et al. (2026) identified distinct psychological profiles among injured combat-sport athletes, and Yung et al. (2025) modeled return-to-sport timelines. Injury and person-level variation therefore enter the framework as testable propositions, and person-centered designs offer a direct route to testing them. Figure 2 summarizes the empirical support by phase, the separate category of relational aftermath, and the moderators that may operate across phases.

**Figure 2:**
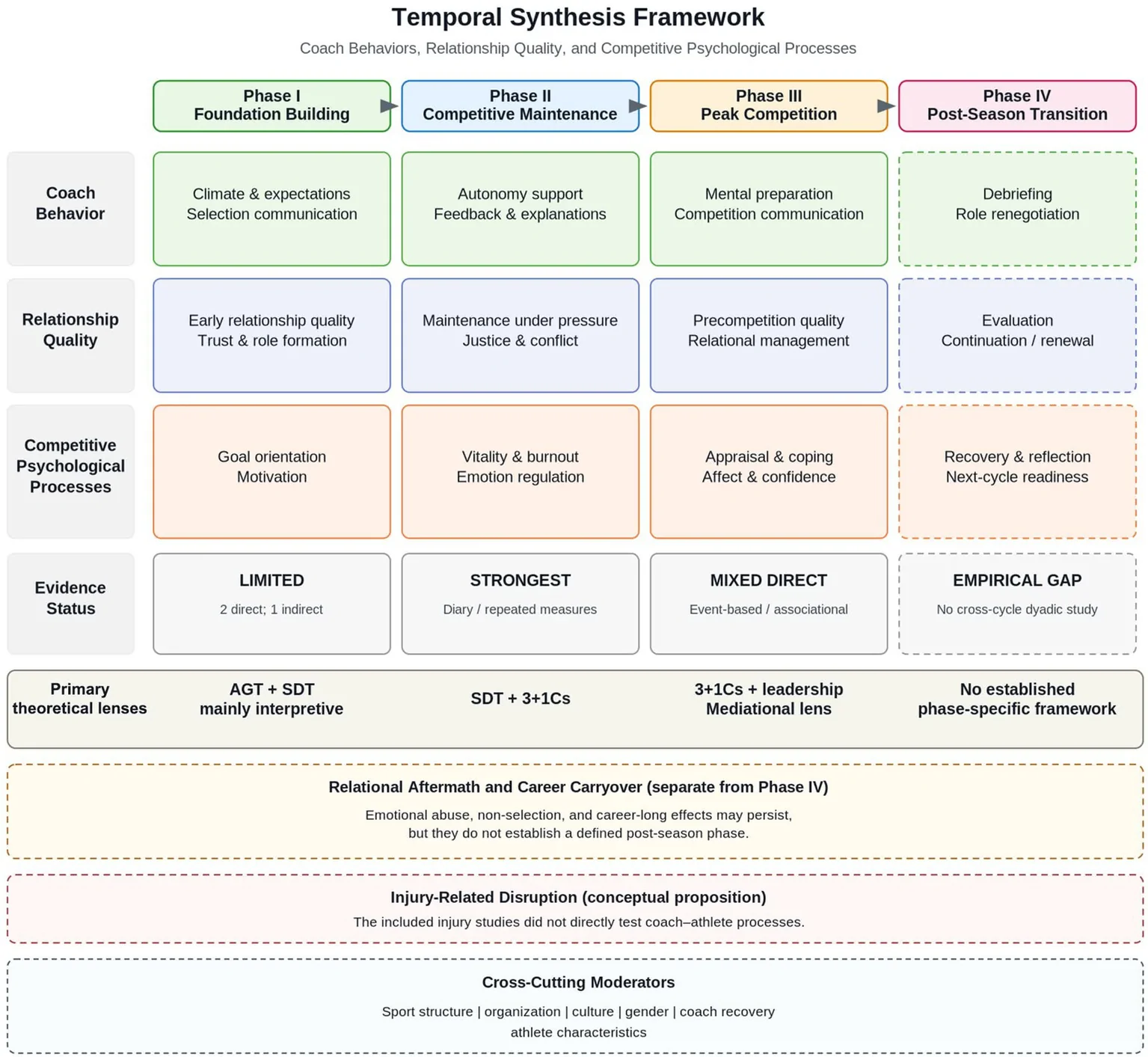
Temporal synthesis framework for coach behaviors, relationship quality, and competitive psychological processes across an elite competitive cycle. The columns organize observed findings and testable propositions by phase; horizontal arrows indicate temporal progression, not causal carryover. Direct empirical support is strongest during competitive maintenance and is considerable but mixed around peak competition. Foundation-building evidence is limited, and post-season transition remains an empirical gap. Relational aftermath and injury-related disruption are shown separately because they do not constitute direct evidence for Phase IV.

## 4 Discussion

### 4.1 Summary of the evidence

The synthesis yielded three defensible findings. Prospective, diary, and repeated-measures studies show that coaching behavior and relationship-related judgments can precede or covary with later motivation, vitality, well-being, perceived justice, and burnout-related outcomes. Competition-focused studies associate relationship quality, identity leadership, communication, and coach self-regulation with appraisal, coping, affect, confidence, and perceived performance. Once the final competitive event has passed, direct evidence becomes sparse. Asymmetric support of this kind is itself informative: it locates where the framework rests on measurement and where it rests on proposition.

The distinction between direct and indirect evidence carries most of that interpretive weight. Phase II contains the strongest within-season designs, including daily diaries, repeated measures, prospective studies, and interventions. Phase III includes event-based and multi-wave evidence, though many links remain statistical associations or qualitative accounts. Phase I contains two direct prospective studies alongside interpretive placements, while Phase IV contains no direct cross-cycle dyadic study. What the review delivers, accordingly, is an organizing framework with a set of testable hypotheses attached to each phase. Such asymmetry also limits what single-time-point studies can establish. A measurement taken in mid-season or immediately before a championship may reflect earlier relationship history, selection decisions, and accumulated fatigue without identifying when those influences emerged. The within-person findings of Cheval et al. (2017), Balk et al. (2019), and De Backer et al. (2018) show why event-aligned measurement is needed: meaningful variation occurs within the same athlete, coach, or team across time. Reading these studies together rather than in isolation can bring out three patterns that would be difficult to see from any one investigation alone. Cheval et al. (2017), Balk et al. (2019), and De Backer et al. (2018) all used within-person or repeated-event designs, and their findings converge on a similar conclusion. During the active season, changes within the same athlete over time were more closely tied to coaching behavior than stable differences between athletes. That distinction matters because between-person designs still dominate much of the literature, and such designs address a different question and may make these associations appear more stable than they are in practice.

A second pattern emerges from the two intervention studies conducted during the season. Cece et al. (2022) found more favorable motivational and emotional trajectories after a need-supportive coaching intervention. Davis et al. (2026), by contrast, reported little change in relationship quality, thriving, or collective efficacy after a workshop-based relationship program. One explanation is that motivational processes may respond more readily to structured input delivered during the season, whereas relationship quality develops through months of repeated interaction and may be less susceptible to brief attempts at change. Another possibility is more straightforward: the interventions differed enough in format, dose, and fidelity of implementation to produce different results. Either way, the contrast suggests that outcomes relevant to the same phase of the season should not be assumed to be equally modifiable within a single intervention period.

The third pattern concerns the structure of the evidence itself. Studies were concentrated where repeated measurement could be fitted most easily into existing routines, such as daily diaries and questionnaires during training weeks or assessments conducted immediately before and after competition. None of the studies in this synthesis followed the same coach–athlete dyads from the final competition of one season, through the off-season, and into the next preparatory period. As a result, the framework’s most consequential proposition that seasonal dynamics carry over into later cycles, is supported only indirectly. The relevant evidence comes from studies of relational consequences or aftermath (Kavanagh et al., 2017; Carleton et al., 2016; Ulaş et al., 2025), not from prospective observations across successive seasons. This is therefore more than a limitation of the present review; it marks a broader gap in the literature. The temporal sequence will remain incomplete until researchers have observed the transition from one season to the next directly.

### 4.2 Interpretation and theoretical contribution

The multi-theory approach is useful when treated as calibrated rather than deterministic. SDT has the strongest direct evidence for maintenance processes involving autonomy support, controlling behavior, need satisfaction, and motivation. AGT helps interpret motivational climate and goal orientation but is less often tested with phase-specific measurement. The 3 + 1Cs model is well suited to relationship quality before and during competition, while the Mediational Model offers a plausible behavior–perception–response sequence that contemporary elite studies seldom test in full. Transformational and identity leadership theories add collective mechanisms in team settings.

The theoretical contribution is therefore not the assignment of one theory to each calendar segment. It is the identification of changing analytical priorities: formation of climate and relationships, maintenance through repeated decisions, event-specific appraisal and coping, and post-season evaluation or carryover. Making each theory’s limits visible is part of that contribution. No framework in the present set was developed to explain how coach–athlete relationships are reviewed after a season, how they dissolve or renew, or how relational residue enters the next cycle; the temporal structure exposes that absence and defines the theoretical work the next generation of studies would need to do.

The separate category of relational aftermath sharpens that gap. Kavanagh et al. (2017), Carleton et al. (2016), and Ulaş et al. (2025) indicate that harmful or poorly explained experiences may continue beyond an immediate interaction. Their findings concern aftermath rather than an ordinary post-season process, yet they suggest that retrospective appraisal and carryover follow a temporal logic of their own, one that theories built for active coaching contexts approximate but do not capture.

A phase-sensitive account may also reduce the apparent opposition between well-being and performance. Hidayat et al. (2026) documented the growing prominence of mental health and well-being in the field, while Nesti (2024) emphasized the continuing performance demands of professional practice. The temporal framework allows these concerns to be examined as connected but differently emphasized across the cycle: motivational satisfaction, trust, and sustainable engagement may be especially salient during preparation and maintenance, whereas appraisal, coping, confidence, and emotional control become more visible around major events. Testing whether that emphasis actually shifts by phase would require the event-aligned designs described above, and the proposition stands or falls with them.

### 4.3 Conceptual propositions for testing

- Early-season perceptions of need-supportive coaching and relationship quality will account for within-person trajectories in motivation and burnout beyond what a single mid-season assessment explains.
- Game-to-game changes in decision explanations and perceived justice will help explain the association between selection status and relational trust during the regular season.
- Precompetition relationship quality will be associated with appraisal and coping; the magnitude and direction of these associations will vary by sport structure and by the coach’s access to the athlete during competition.
- Post-season relational evaluation will be associated with relationship continuation, role renegotiation, and early-season trust in the subsequent cycle.
- Organizational pressure, cultural norms of authority, gender, coach recovery, and athlete characteristics will moderate the phase-specific associations above.

### 4.4 Applied implications

The framework suggests phase-sensitive hypotheses for practice rather than established prescriptions. Coach education could give greater attention to how the function of communication, autonomy support, selection explanations, and relationship repair changes over time. Early preparation may require explicit expectation setting and trust formation; active competition may require repeated explanation, monitoring, and recalibration. Prospective evaluation of these proposals would test the framework’s central practical claim: that when a coaching skill is deployed matters alongside how well it is executed.

Psychological support may likewise be organized around likely phase demands: climate and trust during preparation, maintenance of motivation and recovery during the season, appraisal and coping around major events, and reflective evaluation after competition. The contrasting findings from Cece et al. (2022) and Davis et al. (2026) caution against treating all outcomes as equally modifiable; a program associated with motivational change in one setting does not establish durable change in relationship quality. Matching the target outcome to its likely window of modifiability is the practical lesson those two studies offer together.

Mid-season monitoring may be especially useful because daily and repeated-game evidence indicates considerable short-term variation. Brief assessments of coach recovery, athlete perceptions of autonomy support, perceived justice, and communication could help practitioners identify changes that one-off surveys obscure. Trials comparing monitored against standard support would show whether that information gain reaches athlete outcomes.

Coach welfare should also be treated as part of the athlete environment. Balk et al. (2019) associated coaches’ recovery with athlete perceptions at the daily level, and Fryer et al. (2026) showed that coaches regard their own mental preparation as salient under major-event pressure. Supporting this coach-centered perspective, evidence from wrestling coaches suggests that burnout may negatively affect coaching performance, whereas psychological capital may function as a protective resource in this relationship (Koç, 2023). Direct intervention evidence is thinner here than for athlete-focused support, which makes coach welfare a leading candidate for the prospective designs proposed above: organizational investment in workload, recovery, and psychological preparation plausibly reaches athletes through the daily pathway Balk et al. documented.

### 4.5 Strengths and limitations

The review’s principal strength is its separation of core integrative evidence, contextual evidence, direct temporal support, and interpretive placement. The database master was rebuilt to incorporate 64 Scopus-only records, and all 30 included studies are reported in the evidence table. Operational definitions for the phases, article-level exclusion reasons, and study-level appraisal ratings are provided in the Supplementary materials.

As for limitations, the evidence is heterogeneous and does not support pooled effect estimation. The five-domain appraisal is specific to this review and should not be read as a validated risk-of-bias instrument. Elite status and seasonal timing were sometimes reported imprecisely in the primary studies. Many studies used self-report, small samples, cross-sectional models, or retrospective interviews, and statistical mediation, though common, does not remove confounding or establish causal mechanisms.

The search was restricted to English-language publications in three databases. The core and contextual tiers improved coverage but required judgment about when a one-pillar study materially informed the temporal question. Finally, the four-phase structure may not fit sports with multiple peaks, tournament circuits, overlapping seasons, irregular selection cycles, or frequent changes in coaching personnel. In those settings the framework should be adapted rather than imposed unchanged.

### 4.6 Future research

The strongest test of the framework would follow coach–athlete dyads from late-season competition through post-season evaluation, the off-season, and the next preparatory period. Measurement points should be aligned to meaningful events rather than imposed at equal calendar intervals: selection decisions, competition blocks, debriefs, coaching changes, and return to training.

Daily diary and experience-sampling methods can capture short-term fluctuations in coach behavior, perceived justice, communication, affect, and recovery. Multi-wave dyadic models could estimate actor and partner effects across phases, while observational coding would reduce reliance on athlete or coach self-report alone. Qualitative interviews retain a distinct role here: explaining how the same behavior acquires different meanings across a season is interpretive work that measurement schedules cannot do.

Latent growth, profile, and transition approaches may identify distinct trajectories rather than assume one average pathway. Research should also extend to individual and team sports, hybrid training structures, women’s elite sport, Paralympic sport, and cultures with different norms of authority. Direct tests of phase-by-context moderation would then determine how far the framework generalizes and whether a standard form of psychological periodization can responsibly be recommended.

## 5 Conclusion

Elite coaching relationships unfold in time, but the evidence does not support every segment of the proposed sequence equally. Direct temporal support is strongest during competitive maintenance, where coaching behavior, relationship-related judgments, motivation, well-being, perceived justice, and burnout vary across repeated observations. Around peak competition, relationship quality, communication, identity leadership, and coach self-regulation are associated with appraisal, coping, affect, confidence, and perceived performance; establishing the mechanisms behind those associations is the task the event-aligned designs above are built for. Foundation-building evidence is limited, and direct evidence concerning post-season evaluation and cross-cycle carryover is largely absent. The temporal framework is therefore offered as an evidence-informed research architecture. Its value lies in distinguishing what has been observed from what remains to be tested, while integrating coaching behavior, relationship quality, and competitive psychological processes without forcing one theory or one causal sequence across the entire competitive cycle.

## Data Availability

The original contributions presented in the study are included in the article/Supplementary material, further inquiries can be directed to the corresponding authors.
