# Supplementary material for: Coach behaviors, coach–athlete relationships, and competitive psychological processes in elite sport: a temporal narrative synthesis
Source: Front Psychol. 2026 Sep 29;17:1969318. doi: 10.3389/fpsyg.2026.1969318 (PMC13658106; doi:10.3389/fpsyg.2026.1969318)
Supplement: Supplementary file 1 [file Data_Sheet_1.PDF]

Search, Deduplication, and Screening Audit

Stage	Web of Science	SPORTDiscus	Scopus	Combined	Audit note
Records retrieved	407	75	163	645	Searches completed on 22 June 2026.
Unique before cross-database matching			158		Scopus contained five internal duplicates across S1-S3.
Unique WoS + SPORTDiscus master				417	Existing screening master contained 417 deduplicated WoS/SPORTDiscus records.
Scopus overlaps with WoS/SPORTDiscus			94		91 exact normalized-title matches and 3 manually verified fuzzy matches.
New Scopus-only records			64		Added to the master after cross-database deduplication.
Unique records screened				481	417 + 64.
Duplicates removed				164	
Excluded at title/abstract				276	173 no eligible construct intersection; 71 population not eligible; 32 off-topic.
Advanced to eligibility assessment				205	Full text assessed against the revised criteria.
Excluded at eligibility				175	See Eligibility_Exclusions sheet for article-level reasons.
Included in synthesis				30	21 core integrative studies and 9 contextual studies.

Screening exclusion breakdown

Title/abstract exclusion reason	n
No eligible construct intersection	173
Population not eligible / elite status no	71
Off-topic physiological, technical, tech	32

Eligibility exclusion reason	n
Fewer than two construct pillars	111
Elite/professional status not confirmed	34
No direct or phase-relevant temporal content	18
Insufficient fit with temporal cascade	11
Measurement/review paper not retained	1
Total	175

ID	Title	Authors	Journal	Year	DOI	Search strings	Databases	Abstract
1	Coaching under pressure in Australian high-performance sport: A qualitative study of coach mental performance	Fryer, RP; Mallett, CJ; Kirby, JN; Kashdan, TB	INTERNATIONAL JOURNAL OF SPORTS SCIENCE & COACHING	2026	10.1177/17479541261430313	S1	WoS	Pressure in sport comes from a range of perceived factors including uncontrollability, motivation to succeed, and social evaluation. Psychological flexibility is a concept that has been suggested to help performers navigate challenging situations (such as competition pressure) and maintain effective goal-pursuit. In this study, we investigated sport coaches' experiences during elite-level competitions to deepen the understanding of coach performance under pressure. Ten Australian sport coaches (5 men, 5 women) with an average 22.3 years of experience at Olympic Games, Paralympic Games and World Championships across eight sports (athletics [2], cycling, diving, field hockey [2], netball, rowing, swimming, and water polo) participated in semi-structured interviews. Through a process of reflexive thematic analysis, two main themes were generated: (1) coach mental preparation to perform, and (2) coach mental performance in action. These themes underscored the significance of cognitive, emotional, and behavioural factors in coaching performance at the highest level and emphasised the benefits of articulating specific observable coaching behaviours. The findings are discussed, along with recommendations for more research centred around direct observation of coaching in action.
2	Olympic swimmers' perspectives on how they want to be led	Ferguson, C; Mason, C	INTERNATIONAL JOURNAL OF SPORTS SCIENCE & COACHING	2026	10.1177/17479541261446432	S1	WoS	This study examines Olympic swimmers' leadership preferences within high performance sport environments, addressing a notable gap in literature, as leadership research has predominantly focused on team sports contexts. Recent public and institutional scrutiny of coaching behaviours, safeguarding failures, and athlete welfare within elite swimming further highlight the importance of understanding athletes' perspectives on leadership. Employing semi structured interviews with 15 Olympic swimmers and thematic analysis guided by Chelladurai's Multidimensional Model of Leadership, the research identified four central themes: (1) the critical role of trust and psychological safety within coach-athlete relationships; (2) athletes' preference for collaborative leadership styles over autocratic approaches; (3) the significance of cohesive team cultures and effective interdisciplinary collaboration; and (4) the necessity for comprehensive feedback that clearly communicates the rationale behind training methods. The findings reveal that Olympic swimmers value emotionally intelligent and adaptable leadership, sensitive to their developmental stages and personal experiences. These insights contribute to ongoing debates on effective leadership practices within elite sport by foregrounding athletes' lived experiences of leadership within Olympic swimming. Collectively, these themes offer an interpretive account of how Olympic swimmers make sense of leadership through relational, emotional, and cognitive experiences, highlighting aspects of leadership that athletes themselves consider central within contemporary high-performance swimming environments. Practically, coach education should embed clear rationale-sharing and emotionally intelligent, collaborative practices to protect athlete welfare while sustaining performance. Carrying out this study in the period surrounding Paris 2024 situates these preferences within contemporary high-performance systems.
3	Positive Experiences Among Elite Athletes in Finland: Associations With Basic Psychological Needs, Sport Conditions, and Background Factors	Kaski, SS; Kinnunen, U	JOURNAL OF CLINICAL SPORT PSYCHOLOGY	2026	10.1123/jcsp.2024-0004	S1	WoS	This research aimed to study the prevalence of PERMA experiences (Positive emotions, Engagement, (positive) Relationships, Meaning, and Accomplishment) among elite athletes in Finland (n = 2,045) and factors that are potentially connected to these experiences. The results indicated that, depending on the PERMA component, 40%-52% of the athletes had positive experiences in sports. A total of 42% of the athletes could be classified as fl ourishing. Feeling competent in sports was positively related to all PERMA experiences. The safe atmosphere of the team seemed to be of greater importance for PERMA components than coach behavior. Belonging to a minority group was negatively related to all positive experiences except for Engagement. Our results suggest that strengthening of athletes' feeling of competence is essential in promoting positive experiences in sports, which are important for athletes' functioning and success. In addition, sports organizations should pay more attention to how to strengthen equality and equity in sports.
4	Understanding coach burnout in high-performance sport: the sequential effects of knowledge, psychological safety, and coach-athlete relationships	Tao, RC; Zhang, NY; Guo, C	FRONTIERS IN PSYCHOLOGY	2026	10.3389/fpsyg.2026.1785482	S1	SPORTDiscus+WoS	Background Coach burnout is a persistent concern in high-performance sport, yet limited research has examined how upstream cognitive resources contribute to coaches' wellbeing through psychological and relational processes. Drawing on coaching effectiveness and relational models, this study examined how coach knowledge relates to coach burnout via psychological safety and coach-athlete relationship quality.Methods A sample of elite football coaches completed validated measures of coach knowledge (CKQ), sport psychological safety (SPSI), coach-athlete relationship quality (CART-Q), and coach burnout (CBQ). Structural equation modelling (SEM) was used to test a sequential mediation model.Results Coach knowledge was positively associated with psychological safety and, both directly and indirectly, with coach-athlete relationship quality. Psychological safety did not independently mediate the association between coach knowledge and burnout. However, a significant sequential indirect effect emerged, indicating that coach knowledge was associated with lower burnout through psychological safety and, subsequently, higher-quality coach-athlete relationships. Coach-athlete relationship quality was directly and negatively associated with burnout.Conclusion The findings indicate that coach knowledge becomes consequential for wellbeing primarily through relational pathways, with psychological safety shaping the conditions under which knowledge is enacted within coach-athlete relationships. These results underscore the importance of fostering psychologically safe environments and sustaining high-quality relationships, alongside knowledge development, in efforts to reduce coach burnout in high-performance sport.

5	Exploring stressors in elite rugby league (NRL/NRLW) players	Humphreys, C; Goddard, SG; Poulus, D	JOURNAL OF APPLIED SPORT PSYCHOLOGY	2026	10.1080/10413200.2025.2500963	S1	WoS	Elite athletes in the National Rugby League (NRL) in Australia experience mental health challenges at rates higher than the general population. Stress plays a crucial role in triggering symptoms of mental health, yet knowledge gaps remain about the stressors these athletes face. Additionally, sports psychology research shows a gender disparity, with females being underrepresented. This study aimed to understand stressors experienced by NRL (male) and NRLW (women) players. Semi-structured interviews were conducted with 12 elite players (M age = 27.92, SD = 3.90) contracted across eight clubs in the NRL (n = 7, male) and NRLW (n = 5, female). Data were analyzed using directed content analysis, revealing three primary stressor categories: organizational, competitive and personal. Under-explored themes were identified, such as coach turnover and recurring losses. A significant contribution of this study is the identification of the interconnected nature of stressors, with organizational stressors amplifying competitive and personal stressors, leading to a lack of psychological safety. The analytic process elucidates how cultural background shapes experiences with themes like coaching style and how coaching style and teammates can influence themes such as off-field incidents. The theme ingrained culture of toughness also interacts with themes such as social media discrimination. NRLW players highlighted additional unique themes, including a lack of credibility and navigating gender dynamics within a male-dominated sport. The findings underscore the need for further research into the organizational environment to understand and foster culturally appropriate psychological safety and provide tailored support for NRL and NRLW players.
6	Exploring the relationship between burnout and mental health in elite athletes: the role of moderators	de Subijana, CL; Wergin, VV; Mallett, CJ; Haslam, A	INTERNATIONAL JOURNAL OF SPORT AND EXERCISE PSYCHOLOGY	2026	10.1080/1612197X.2025.2479715	S1+S3	WoS	The strain of intense competition at the very highest performance levels can sometimes compromise athletes' health. Understanding the mechanisms underlying the associations between burnout and mental health in elite athletes is an important endeavor in sport psychology. The goal of the present research was to examine how selected moderators are associated with burnout and mental health in elite athletes. One hundred and eighty-one elite athletes completed a survey assessing potential burnout, coach identity leadership, and mental health. The results showed that if athletes perceived their coach to be engaging in identity leadership and they were performing in a team sport, this buffered the impact of burnout on their mental health. At the same time, competing at a higher competitive level and being trained by a coach for a longer period (more than 2.8 seasons) increased the effect of burnout on athletes' mental health. This suggests that elite athletes who are involved in individual sports and who have long relationships with their coaches may be particularly vulnerable to mental health problems.
7	Why do elite athletes consider leaving their sports? Longitudinal evidence on the development of dropout considerations	Blessing, J; Hilpisch, C; Krüger, K; Raab, M; Wiese, L; Zentgraf, K; Mutz, M	INTERNATIONAL JOURNAL OF SPORTS SCIENCE & COACHING	2026	10.1177/17479541261438700	S1+S3	WoS	Dropout from elite sport poses a critical challenge to both athletes and sport systems, particularly when it occurs despite ongoing competitive potential. While performance development remains the system's central selection criterion, personal and environmental factors may also influence athletes' dropout considerations. This study investigated how dropout considerations evolve over time and which factors may contribute to these changes directly or indirectly via perceived performance development. Using a longitudinal design with two measurement points (T1 and T2), we analyzed survey responses from N = 235 German elite athletes (M = 17.5 years; 46.4% female). Change-score analyses indicated that dropout considerations were dynamic, with individual patterns reflecting both intensification and decline. Regression models predicted dropout considerations at T2 controlled for baseline dropout considerations at T1. Results showed that mental health problems, dissatisfaction with training conditions, and time and role conflicts were directly associated with increased dropout considerations, whereas burden of injury and social support showed indirect associations via perceived performance development. Motivation played a dual role, predicting dropout considerations both directly and indirectly via perceived performance development. These findings highlight key targets for preventive intervention and underscore the importance of addressing psychosocial and environmental factors alongside athletic performance.
8	I even called him coach at home: A qualitative study on role conflict and coping in parent-coached elite athletes	Alp, AF; Karagün, E	INTERNATIONAL JOURNAL OF SPORTS SCIENCE & COACHING	2026	10.1177/17479541251393447	S1	WoS	This study explores the dual role of parent-coaches and its impact on elite athletes' professional and emotional experiences, with a particular focus on role conflict. Drawing on a qualitative phenomenological approach, in-depth semi-structured interviews were conducted with 11 elite athletes (Mage = 26.8, SD = 5.28) competing in Taekwondo, Judo, and Karate-all of whom were coached by a parent. Participants were recruited through purposive and snowball sampling strategies. Thematic analysis identified role conflict as a central and multifaceted theme, comprising four subdimensions: (1) Dual Role Conflicts, (2) Athletes' Coping Strategies, (3) Parent-Coaches' Role Conflicts, and (4) Parent-Coaches' Coping Strategies.The findings illustrate that the parent-coach relationship is often described as a complex and paradoxical dynamic, creating tension between emotional intimacy and professional expectations. Across participants' accounts, success as a filial duty emerged as the central organizing concept, shaping how athletes interpreted their struggles and coping mechanisms. Athletes reported struggles with blurred boundaries, feelings of entrapment, and a perceived obligation to achieve as a form of filial duty. According to athletes, parent-coaches experienced internal conflicts and fairness-related concerns, often managing these tensions through compensatory behaviours. Despite these tensions, both athletes and parent-coaches developed various strategies to manage conflicting roles. This study contributes to a nuanced understanding of the psychological and relational complexities of dual-role dynamics in elite individual sports.

9	Unveiling the coach-athlete relationship in Major international competitions: A qualitative study of national team coaches	Braga-Pereira, R; Bernardo-Ramos, J; Castro-Vigário, P; Duarte, D; Teques, P	INTERNATIONAL JOURNAL OF SPORTS SCIENCE & COACHING	2026	10.1177/17479541261431543	S1	WoS	Although the coach-athlete relationship has been widely examined across competitive levels, limited research has focused on its dynamics within elite national team settings. This study explored the coach-athlete relationship in the context of national team coaches participating in major international competitions (e.g., European Championships, World Championships, and Olympic Games). A semi-structured protocol was used to interview 13 elite national team coaches from team and individual sports. A reflexive thematic analysis revealed three higher-order themes: (1) Coaching identities are shaped through experience and transition, highlighting how national team coaches build their profiles through personal pathways, lifelong learning, and relational competence; (2) Constructing performance beyond skill, showing that athlete selection transcends technical-tactical criteria, integrating psychosocial fit and team dynamics; and (3) Navigating connections in high-stakes contexts, illustrating how relational management, closeness, and complementarity influence sports success across competition phases. These findings expand existing knowledge by offering an in-depth understanding of coach-athlete relationship in elite national team settings, emphasising the interplay between individual profiles and relational processes. This study reinforces the importance of relational expertise as a core coaching skill and underlines the value of adaptive leadership in managing interpersonal dynamics. The practical implications include guiding the design of professional development programmes to strengthen interpersonal coaching skills and informing selection and communication strategies within national teams. By addressing a gap in the literature, this study contributes to enhancing the quality of coach-athlete interactions in high-performance environments and offers practical insights for federations, coaching organisations, and sport governing bodies aiming to optimise professional development and team performance.
10	Shame following performance failure as a potential mediator between sport contingent self-esteem and both competitive anxiety and burnout among elite athletes	Funke, A; Gustafsson, H	ACTA PSYCHOLOGICA	2026	10.1016/j.actpsy.2026.106694	S1	WoS	Previous research has demonstrated that basing self-esteem on performance in a specific context can yield both favorable and adverse outcomes. To assist high achievers whose performance is strongly connected to their selfworth, an important initial step is to identify when this becomes problematic. From a theoretical standpoint, we hypothesize that shame following performance failure may help explain the association between basing selfesteem on performance and negative outcomes related to performance and health. That is, it may be when basing self-esteem on performance develops into shame following performance failure that such consequences emerge. To explore this hypothesis, we conducted a cross-sectional study with 176 elite athletes to examine whether shame following performance failure statistically mediates the associations between basing self-esteem on performance and competitive anxiety and athlete burnout. The findings in the present study were consistent with the idea that shame following performance failure could play a key explanatory role. Initial analyses showed significant direct associations between basing self-esteem on performance and five out of six dimensions of competitive anxiety and athlete burnout, with correlation coefficients ranging from 0.42** to -0.22**. However, when shame following performance failure was statistically tested as a potential mediator, these direct effects were substantially reduced and became non-significant (ranging from 0.08 to -0.02). This pattern is statistically consistent with mediation, although it should not be interpreted causally given the cross-sectional nature of the data. Despite limitations, this study may offer an important step toward a better understanding of when, and under what circumstances, basing self-esteem on performance leads to negative outcomes.
11	Leadership micro-behaviors and performance stability under competitive pressure: a stepped-wedge field study in professional football	Paasch, R; Mau, G	FRONTIERS IN PSYCHOLOGY	2026	10.3389/fpsyg.2026.1833589	S2+S3	SPORTDiscus+WoS	Leadership in elite sport is often assumed to directly enhance performance outcomes, yet empirical evidence remains inconsistent, particularly under conditions of high competitive variability. The present study examined whether a brief, behavior-focused PERMA-based leadership intervention was associated with changes in psychological team resources and performance stability in competitive football teams. Using a stepped-wedge field design, 10 teams were observed across up to 24 competitive weeks, combining longitudinal team-level panel data with intensive daily diary assessments (1,567 player-day observations). Fixed-effects and event-study models were used to estimate within-team changes following intervention onset. Perceived leadership micro-behaviors increased following intervention onset, alongside higher levels of psychological safety and collective efficacy. At the performance level, no consistent changes in match points were observed, whereas performance instability decreased, reflecting lower week-to-week fluctuation across matches. Additional robustness analyses were consistent with the interpretation that observed patterns were more closely aligned with reduced performance variability than with systematic changes in average performance levels. Moderation analyses suggested that these associations were attenuated under conditions of elevated competitive pressure. Daily diary results provided converging evidence for changes in leadership perception and psychological safety, but not for load-dependent experiential states such as flow or sleep. These findings suggest that leadership micro-behaviors may be associated with context-sensitive patterns of team functioning, particularly in relation to performance stability rather than immediate competitive success. By distinguishing between performance stability and outcome performance, the present study contributes to a more differentiated understanding of leadership in elite sport and highlights the potential relevance of behavior-focused interventions in applied competitive settings.

12	Point-ending outcome patterns in break-point situations in professional tennis: Effects of player position and court surface	Mourtzios, C; Gouvelis, N; Mourtziou, MA	INTERNATIONAL JOURNAL OF SPORTS SCIENCE & COACHING	2026	10.1177/17479541261442085	S2	WoS	This study examined the distribution of point-ending outcomes during break-point situations in professional men's tennis, with particular attention to differences between players in the serving and receiving positions and the potential influence of court surface. A total of 1475 break points were analyzed from 80 matches across the four Grand Slam tournaments. Each point was categorized according to its observable outcome, including aces, winners, forced errors, and opponent unforced errors. The analysis revealed significant differences in outcome distribution based on player position. Points won by serving players more frequently concluded with outcomes classified as aces, winners, or forced errors, whereas points won by receivers more commonly ended with unforced errors committed by the opponent. In contrast, statistical analysis did not reveal significant differences in the distribution of these point-ending outcomes across clay, grass, and hard court surfaces. These findings provide descriptive insights into how break points are resolved during high-pressure moments in elite tennis competition. However, it should be emphasized that the outcome classification reflects the final observable event of the rally and does not necessarily indicate the tactical intention or decision-making processes of the players. The results may assist coaches and performance analysts in understanding outcome patterns associated with critical points in professional tennis.
13	Acute weight cycling and hydration status in elite kickboxers: a repeated-measures field study	Ceylan, B; Taskin, HB; Sarikaya, U; Pekel, HA; Sabirli, TN	BMC SPORTS SCIENCE MEDICINE AND REHABILITATION	2026	10.1186/s13102-026-01669-x	S2	Scopus+WoS	Objective This study aimed to monitor acute changes in body weight and hydration status-measured via urine-specific gravity (USG) and urine color (UC)-in elite-level kickboxers during a real-world competitive environment. The research sought to provide critical insights into current weight-management practices and the prevalence of hypohydration during the pre-competition period. Methods Using a cohort repeated-measures design, ten elite male kickboxers (age: 20.5 +/- 2.8 years) were monitored at four distinct time points: one week before the competition (T1), one day before the official weigh-in (T2), at the official weigh-in (T3), and on the competition day (T4). Body weight was measured using a calibrated scale, and body composition was assessed via bioelectrical impedance. Hydration status was evaluated through midstream urine samples analyzed for USG and UC. Hypohydration was defined as a USG >= 1.020. Results Athletes underwent significant rapid weight loss (RWL), losing an average of 3.1 +/- 1.7 kg (4.2%) in the week leading up to the weigh-in. This was followed by a rapid weight regain (RWG) of 3.3 +/- 1.4 kg (4.8%) between the weigh-in and the start of the competition. Hydration markers worsened significantly as weight decreased, with USG and UC values peaking at the official weigh-in (1.030 +/- 0.006 and 7.3 +/- 1.3, respectively), indicating peak hypohydration. Notably, while 50% of athletes were hypohydrated at T1, all remained hypohydrated on competition day (T4) despite the substantial weight regain. Conclusion The results indicate that elite kickboxers engage in significant RWL followed by RWG that returned athletes to their starting body weight. However, this weight gain does not equate to recovery of a euhydrated state, as the vast majority of athletes remain hypohydrated at the start of competition. Coaches and athletes should implement personalized, professional rehydration plans rather than relying on acute weight gain as a proxy for hydration recovery.
14	Workload and travel demands of professional basketball staff: A season-long case study across the National Basketball Association and G League	Mercer, RAJ; Russell, JL; Strack, DS; Coutts, AJ; McLean, BD	INTERNATIONAL JOURNAL OF SPORTS SCIENCE & COACHING	2026	10.1177/17479541261449029	S3	WoS	Staff in high-performance sport face considerable workload challenges, including long and irregular hours, frequent travel, and demanding responsibilities. These demands reflect substantial professional time commitments within increasingly professionalized and resource-intensive environments, highlighting the need to understand how staff work and cope with demanding schedules. This study examined work hours, travel requirements, and schedule characteristics of staff from one National Basketball Association (NBA) team and one NBA G League team across a full year. A longitudinal, observational design captured preseason, competitive season, and off-season workloads across departments (e.g., coaching, medical, performance). Work hour estimates were derived from team schedules and structured interviews with 10 senior staff representing departments across both teams (7 NBA and 3 G League staff; age = 40.0 +/- 8.0 [29-60] years, experience in NBA = 13.3 +/- 7.2 [6-28] years). Travel data included number of trips, time zones crossed, hours traveled, and days away from home. Unconventional working hours and recovery time between consecutive days were also calculated. The NBA and G League preseason and competitive season phases spanned 28.6 and 25.5 weeks, respectively. During the competitive season, NBA games occurred every 2.1 +/- 0.9 days and G League games every 2.8 +/- 1.6 days, with an average of 15.1 +/- 6.8 and 11.2 +/- 6.0 days between 'building closed' days (i.e., non-work/travel days), respectively. NBA staff worked 44.2-66.7 h per week during the season and 37.1-49.1 in the off-season, with 41-49% of work occurring outside 09:00-17:00 in-season. G League staff worked 38.9-56.3 h weekly during the season. Staff spent 46% (NBA) and 42% (G League) of the competitive season away from home, averaging 9.7 +/- 2.7 and 15.7 +/- 6.4 travel hours per week away from home, respectively. NBA travel occurred mainly overnight on charter flights, while G League staff traveled mostly during the day via commercial flights or bus. These findings highlight prolonged hours, frequent travel, and limited recovery, underscoring the need for organizational strategies to optimize scheduling and support staff well-being.

15	Features of successful high-performance sport environments: Stakeholder insights	Schlawe, A; Christiansen, AV; Henriksen, K	JOURNAL OF APPLIED SPORT PSYCHOLOGY	2026	10.1080/10413200.2026.2683956	S3	WoS	This study explores features of high-performance sport environments (HPSEs) that support athletes to thrive. Situated in the Danish elite sport context and informed by a holistic ecological approach, the study draws on semi-structured interviews with 13 stakeholders, including coaches, performance directors, sport managers, and sport psychologists, representing a range of Olympic and non-Olympic sports. The study is situated within a qualitative interpretivist paradigm,and draws on reflexive thematic analysis to identify features that stakeholders perceived to be important for supporting the joint pursuit of performance and well-being. Seven interconnected features were identified across micro- and macro-level levels of the environment. At the micro level, stakeholders highlighted competitive cohesion, structure and flexibility, psychological safety and compassion, and a whole-person focus as central to supporting athlete thriving At the macro level, thriving was perceived to be shaped by transparent communication and collaboration, integration of short- and long-term vision, and strategic resource use. . Together, these findings demonstrate that thriving may be supported by factors distributed across the broader sport environment rather than solely within the immediate training context. The study extends understanding of thriving in HPSEs by identifying features that may facilitate the sustained pursuit of both high performance and well-being and encourages practitioners to adopt a holistic perspective when designing and evaluating high-performance sport environments, with attention to the environmental features that shape athletes' opportunities to thrive over time
16	Applying the 5Cs Framework to Elite Youth Tennis: Impact Factors in a Talent Development Environment	Harwood, C; Porter, K	BEHAVIORAL SCIENCES	2026	10.3390/bs16020166	S3	Scopus+WoS	With the growing demands and expectations associated with professionalised youth sport environments, there is an increasing need for psychosocial development initiatives to support young athletes and their healthy progression. The aim of this study was to extend and investigate the application of the 5Cs framework, a prominent psychoeducational approach in sport psychology, to a youth tennis Talent Development Environment (TDE). Using a collective case study design, five athletes, their parents and two coaches (n = 12) participated in a season-long multimodal 5Cs intervention programme at a British Regional Player Development Centre (RPDC). The 30-week programme was delivered by an embedded sport psychology practitioner (SPP) and incorporated a blocked educational curriculum supported by a range of athletes, coach and parent development strategies. Post-intervention semi-structured interviews were conducted with all participants, with reflexive thematic analysis leading to three overarching themes. Findings highlighted the positive influence of the programme, with perceptions of the framework's effectiveness associated with its specificity to tennis and individual athlete needs, the collaboration of all stakeholders across the TDE and the use of developmentally accessible and innovative strategies enabling evidence of athlete improvements. Researchers, practitioners and sport organisations are encouraged to consider these impact factors in terms of supporting the development, performance and well-being of athletes and their families in competitive youth sport contexts.
17	Understanding the motivators and barriers of elite athletes as role models	Praet, L; De Bosscher, V; Smismans, S; Derom, I	SPORT IN SOCIETY	2025	10.1080/17430437.2025.2563822	S1	Scopus+WoS	Elite athletes are often leveraged as role models, regardless of their willingness to fulfil this role. Yet, little is known about how athletes perceive themselves as role models, what motivates them to be a role model or what barriers they encounter. Using Self-Determination Theory, this study explored these questions through semi-structured interviews with 20 Belgian elite athletes. Athletes' perceptions are more nuanced and cannot be reduced to 'athletes are role models' or 'athletes are not role models'. Athletes experienced intrinsic (e.g. satisfaction) and extrinsic (e.g. rewards) motivators, with feelings of competence, autonomy and social relatedness increasing their intrinsic motivation. Athletic, organisational, initiative-specific, personal and social barriers were identified. Findings highlight the importance of including an athlete-centred approach when developing role model theories within the sport's context. Sport stakeholders are advised to foster an adequate motivational climate that supports athletes' autonomy, competence and social relatedness, while tackling barriers at different levels.
18	Exploring the barriers to athlete personal development within UK Olympic and Paralympic sport	Holden, J; Wagstaff, CRD; Wadey, R; Brown, P	PSYCHOLOGY OF SPORT AND EXERCISE	2025	10.1016/j.psychsport.2025.102966	S1	WoS	Elite athletes face a range of challenges throughout their careers including injury, selection, funding status, and retirement. To support athletes to plan for and overcome these challenges, Career Assistance Programs (CAPs) have been developed to support personal and professional development, yet engagement with these services remains low. The aim of this study was to explore the perceived barriers to athletes' engagement with personal development within the UK High-Performance sport system. Underpinned by ontological relativism and epistemological constructivism, 15 elite athletes who were on a funded UK Sport World Class Olympic or Paralympic Program participated in semi-structured interviews. The dataset was analyzed using reflexive thematic analysis. Three themes were developed: The Athlete Bubble, outlines the barrier of the performance-driven culture, Head in the Sand reflects the fear of retirement, and Bridging the Employment Gap highlights the tension of meeting training demands with work opportunities. Findings reveal how cultural expectations, elite sport environments, coach-athlete relationships and scheduling conflicts inhibit athlete engagement with personal development. This study contributes to a deeper understanding of the cultural and systemic barriers that shape how athletes interpret and navigate their development journeys and offers direction for developing more integrated, athlete-centered CAPs.

19	Evaluating a behaviour change intervention to enhance athlete monitoring system engagement: Insights from elite sport	Neupert, EC; Holder, T; Jobson, SA	INTERNATIONAL JOURNAL OF SPORTS SCIENCE & COACHING	2025	10.1177/17479541251338804	S1	WoS	Athlete engagement with monitoring remains challenging, despite athlete monitoring systems (AMS) having the potential to enhance performance. Behaviour change frameworks may offer strategies to improve AMS engagement, but are underutilised in elite sport. This study assessed the practicality and utility of a behaviour change intervention (BCI) to improve AMS engagement. Three national team coaches (43.6 +/- 10.0 years) and eight athletes (20.1 +/- 2.0 years) participated in a six-month BCI to promote AMS adherence to daily monitoring using the Behaviour Change Wheel (BCW). All participants completed pre and post intervention semi-structured interviews, which were thematically analysed. Adherence to monitoring declined during the intervention, but the coaches reported improved athlete awareness of monitoring. Reduced adherence could be attributed to the partial implementation of the intervention caused by coaching personnel changes. In the post intervention interviews athletes indicated there was considerable intercoach variability in AMS use and feedback to athletes, frustrating both athletes and coaches. The coaches noted athletes lacked motivation and understanding of the AMS and its purpose, further hindering engagement. Overall, the use of the BCW enabled a feasible intervention to be devised, but the BCW proved cumbersome to adapt to the swift organisational changes often experienced in elite sport. Future interventions should consider ensuring a consistent and shared framework for AMS use between staff. Behaviour change targets should have contingencies for organisational changes, and focus on key interactions such as the coach/athlete relationship, its inter-relation with AMS data, and the feedback of data between the coach, athlete and practitioner.
20	Disciplinary strategies or reactive measures? An analysis of coach sanctions in elite Danish handball leagues	Gençoglu, C; Gümüs, H	INTERNATIONAL JOURNAL OF SPORTS SCIENCE & COACHING	2025	10.1177/17479541251360847	S2	WoS	This study analyzed coach sanctions in elite Danish handball matches across seven seasons (2017-2024), focusing on differences by league, gender, match period, and situational context. A total of 2978 matches and 398,054 events were collected from official match analyses. Sanctions were categorized by type (yellow card, 2-min suspension, blue card) and compared using chi-square tests and Spearman correlations. A two-minute retrospective event analysis was performed to identify triggers preceding coach sanctions using a custom Python code. The results showed that sanctions were more frequent in the Men's League than in the Women's League and occurred predominantly in the second half of matches. Positive correlations between coach and player sanctions were consistently stronger in the Women's League than in the Men's League (variable across seasons). Significant events such as goals, saved shots, technical mistakes, and turnovers were commonly recorded immediately before the coach's sanctions. Findings indicate that coach sanctions serve regulatory and strategic functions, with gender differences suggesting divergent disciplinary and management styles. These results highlight the role of situational pressure in triggering coach sanctions and suggest that coaching strategies should consider managing disciplinary responses under competitive stress.
21	The epidemiology of injuries in professional sitting volleyball athletes	Mahmoudkhani, M; Alizadeh, F; Khodsiyani, E; Norouzi, M	PHYSICIAN AND SPORTSMEDICINE	2025	10.1080/00913847.2025.2498317	S2	Scopus+WoS	Objectives This study investigated the epidemiology of injury among players at the Iran Sitting Volleyball Super League 2023-2024. Methods A total of 56 athletes from 6 teams, representing 47% of all athletes in the Super League, took part in this study. Injury data were obtained retrospectively through one-by-one interview sessions and a questionnaire. Results A total of 80 injuries were reported in 30 athletes at the Iran Sitting Volleyball (SV) Professional League. The injury proportion (IP) (%) of all athletes who sustained an injury during the League was 53.6%, with an overall incidence of 16.2 injuries per 1000 athlete days (95% CI 12.7 to 19.8). There was a significantly higher incidence of injury during the competition period (33.5 (95% CI 16.5 to 50.4)) compared with the pre-competition period (14.5 (95% CI 11.0 to 18.0); IR:2.31 (95% CI 1.3 to 4.0); p < 0.01). There were no significant differences between post and age groups concerning injury rate in the overall period. The injuries incurred are mostly re-injuries, and the anatomical areas of the fingers, wrist, and shoulder are at the most risk for injury. Conclusion This study provides important baseline data for medical staff and coaches, aiding in better preparation for long-term competitions and improving injury prevention programs for this sport.
22	Burnout in medical and performance professionals working in elite football in the United Kingdom: a pilot study	Mifsud, D; Acampora, N; Rao, NL; Shafik, A; Carmody, S; Chandran, A	SCIENCE AND MEDICINE IN FOOTBALL	2025	10.1080/24733938.2025.2476486	S3	WoS	BackgroundDespite the focus on the effect of burnout on athlete health and performance, understanding its impact on their support teams remains limited. Our primary aim was to investigate the prevalence of burnout amongst medical and performance professionals working in professional football in the UK.MethodsIn this cross-sectional design study burnout risk was assessed using the Burnout Assessment Tool-23 (BAT-23).ResultsA total of 162 participants completed the questionnaire. The overall average burnout score indicated a low risk of burnout; however, half (42%) of participants had individual burnout scores indicative of being at risk or very high risk of burnout. There was no statistically significant relationship between profession and burnout risk. There was also no statistically significant relationship between burnout risk and number of years working in professional football.ConclusionThis study, conducted during pre-season, suggests that medical and performance professionals working in professional football in the UK are not at risk of burnout. Measures to continue to safeguard practitioners against the effects of burnout are proposed.

23	A Longitudinal Study of Organizational Stressors and Mental Health in the Irish Olympic Team Before and After the Tokyo 2020 (2021) Olympic Games	Browne, JA; Paradis, KF; Kirby, K; Arnold, R	SPORT PSYCHOLOGIST	2025	10.1123/tsp.2024-0007	S3	WoS	Aims: The primary aim of the present study was to assess the relationship between organizational stressors and mental health and well-being indicators of the Irish Olympic Team before and after the Tokyo 2020 Olympic Games. A secondary aim was to examine the differences in mental health and well-being between the athletes and staff of Team Ireland pre- and post-Games. Method: The Irish Olympic Team comprised 271 members (116 athletes and 155 team staff). Participants were sent an online survey package 2 weeks pregames (Time 1) and 4 weeks postgames (Time 2). A total of 98 participants (36% response rate) responded at Time 1, and 70 participants (26% response rate) responded at Time 2. Measures included the Organizational Stressor Indicator for Sport Performers, the Mental Health Continuum-Short Form, and the Sport Mental Health Assessment Tool 1. Results: There was a significant inverse association between organizational stressors and mental well-being at both Time 1 (beta = -0.46) and Time 2 (beta = -0.35) and from pre- to post-Games (beta = -0.48). There was a significant positive association between organizational stressors and risk of mental health symptoms at both Time 1 (beta = 0.69) and Time 2 (beta = 0.67) and from pre- to post-Games (beta = 0.34). At Time 1, team staff (75%) reported significantly greater risk of mental health symptoms than athletes (50%); however, at Time 2, athletes (80%) reported significantly greater risk of mental health symptoms than staff (50%). The overall number of participants flourishing decreased from pre- (43%) to post-Games (31%). Conclusion: Findings have implications for mental health support provision, which tends to be athlete focused but might forget about team staff, and to consider preevent provision but to also not forget about postevent care
24	Optimizing recovery: the impact of training load in elite volleyball players	Rebelo, A; Pereira, JR; Vande Broek, G; Coelho-e-Silva, MJ; Elferink-Gemser, MT; Valente-dos-Santos, J	INTERNATIONAL JOURNAL OF SPORTS MEDICINE	2025	10.1055/a-2519-9330	S3	WoS	This study aimed to analyze the relationships between training load metrics and various dimensions of recovery over the course of 105 days during the competitive season. This study included 14 elite male volleyball players who participated in 95 training sessions and 18 matches. The session rating of perceived exertion and inertial measurement units were utilized to measure internal and external training loads, respectively. Biological recovery was assessed using the countermovement jump test, while social and psychological recoveries were measured through the RESTQ-Sport. Relationships were examined using linear mixed-effect models to account for repeated measures across players and time points. The results revealed that chronic external training loads negatively influenced the biological recovery, with increases in chronic external training loads associated with a longer time to take-off (beta =- 0.008, p =0.03) and a reduced modified reactive strength index (beta =- 0.008, p =0.047). Social recovery was influenced by internal training load metrics, with acute internal training loads increasing social stress (beta =- 3.512, p =0.034). For psychological recovery, chronic internal training loads and the acute:chronic internal training load ratio were linked to higher emotional exhaustion (beta =- 0.013, p =0.029; beta =0.021, p <0.001, respectively). These findings highlight the critical role of external load management in the biological recovery and the relevance of subjective internal load measures in monitoring social and psychological recovery dimensions. Coaches are encouraged to integrate time-based neuromuscular metrics and perceptual load assessments into their recovery monitoring protocols to optimize athletes' well-being and performance.
25	Examining the relationship of training load and menstrual cycle on a web application-based load and recovery score: A longitudinal observational study in elite women's soccer	Brefin, SL; Anderegg, J; Nigg, CR; Koschnick, D; Paul, C; Ketelhut, S	INTERNATIONAL JOURNAL OF SPORTS SCIENCE & COACHING	2025	10.1177/17479541251349332	S3	WoS	Monitoring training load and recovery is crucial in team sports. Despite the increasing prevalence of professional women's sports, most monitoring methods fail to account for the menstrual cycle (MC). Given the potential influence of the MC on sports performance, training responses, and recovery, incorporating it into monitoring practices is essential. This study assessed the relationship between training load and a newly developed web application-based Load and Recovery Score (LRS) among elite female soccer players. Furthermore, it explored the potential influence of the MC on the individual training responses. Forty-nine female elite soccer players were recruited for this 6-week observational study. The LRS was recorded daily using a web application, and a subgroup of 34 players recorded their basal temperature every morning, using a digital thermometer. Training load was assessed based on trainer-intended ratings of perceived exertion. A significant negative relationship was found between training load and players' LRS (Est. = -0.009, 95% CI -0.011, -0.007, p < .001). When controlling for training load, no significant effect of the MC on players' LRS was found (F = 1.274, p = .283). The explained variance of both models was 46.59% and 50.07%, respectively, with a high proportion of variance attributed to random effects (43.67%, and 47.43% respectively). The LRS represents a multifactorial tool that depicts training load in terms of Trainer Session Rating of Perceived Exertion, helping coaches identify athletes' responses to training stimuli. While the MC did not show a significant effect on the LRS, systematic monitoring should still be considered, as its irregularity may indicate serious health problems.

26	Evidence for the reversal of the relative age effect in European professional basketball	Akinci, Y; Yapar, A	INTERNATIONAL JOURNAL OF PERFORMANCE ANALYSIS IN SPORT	2025	10.1080/24748668.2025.2532271	S3	WoS	The Relative Age Effect (RAE) refers to how the selection process based on birthdates can influence athletic success and development. This study investigates the RAE in European professional basketball, focusing on its effects on roster composition, player performance, and team efficiency. Data from 2,571 male players across the top 10 European leagues in the 2022/23 season were analysed. The analysis utilised chi-square tests to evaluate birth quartile distribution, ANOVA for assessing playing time and efficiency scores, and correlation analyses to explore the relationship between birth quartile distribution and team success in league level. The findings confirm the existence of RAE, revealing an over-representation of players born in the first quarter of the year. However, contrary to expectations, players born in the last quarter received more playing time and had higher efficiency scores, particularly in the Turkish and Italian leagues. Additionally, a weak negative correlation was found between team success and RAE, suggesting that teams with a higher proportion of first quarter-born players performed worse. The study highlights the limitations of age-based selection and calls for alternative talent identification methods that could be beneficial for long-term career outcomes in professional basketball.
27	Influence of Mental Skills on Competitive Anxiety in Professional Futsal Players: Difference by Successful and Unsuccessful Players.	Kemarat, Supatcharin ; Theanthong, Apiluk ; Yeemin, Wichai ; Suwankan, Sutima ; Makaje, Niromlee	Trends in Psychology	2025	10.1007/s43076-023-00330-7	S2	SPORTDiscus	This study investigated the influence of mental skills on competitive anxiety in professional futsal players and measured the differences between successful and unsuccessful players. Participants involved four top and four bottom ranking teams (10 players each) qualified in the national futsal league. All participants completed the questionnaires concerning their mental skills and competitive anxiety using the athletic coping skills inventory-28 and the sport competitive anxiety tests. Comparisons between the successful and the unsuccessful players were analyzed by using multivariate analysis of variance. The results showed that the successful players demonstrated significantly greater scores in the goal-setting and mental preparation than the unsuccessful players (2.12 ± 0.61 and 1.84 ± 0.57, $p < 0.05$). However, no difference in the competitive anxiety was observed. Moreover, stepwise regression analysis found that for the successful players, peaking under pressure ($\beta = -0.68$) and concentration ($\beta = 0.48$) explained 22% of the variances in the competitive anxiety, whereas for the unsuccessful players, approximately 20% of the variances in the competitive anxiety were explained by freedom from worry ($\beta = -0.36$) and coachability ($\beta = -0.32$). With greater ability in the goal setting and mental preparation in the successful players, these could help focusing on the game and encounter critical situations. However, they should rely on optimal concentration since excessive focus may cause stress and performance anxiety. To promote the unsuccessful players, coaches should provide a variety of strategies for delivering constructive criticism to help athletes adjusting movements and improving overall performance. Also, simulation training is recommended in order to prepare athletes to effectively handle the pressures of playing.
28	Developing guidelines for selection-deselection in high performance sport for athletes, coaches, and organisations: A delphi study	Slade, K; Jowett, S; Rhind, D	JOURNAL OF SPORTS SCIENCES	2024	10.1080/02640414.2024.2387968	S1	Scopus+WoS	The process of athlete selection and deselection in sport involves not only athletes but also coaches, managers, performance directors, talent pathway coordinators and the wider organisation. Athlete selection and deselection can be viewed as the opposite sides of the same coin in that the process may be the same for all athletes but the outcome is very different. The outcome of this process can evoke extremely powerful emotions ranging from elevation to devastation. While selection and deselection are part of competitive sport regardless of type, level, gender or age, research is scarce. Employing the Delphi method, a total of 20 participants comprised the expert panel (coaches, athletes and other key personnel in high performance) from various sports, and ages ranged from 21 to 59 years old. Following three rounds, 60 items reached the pre-determined consensus level of 75%. The 60 items were then further content analysed and grouped with respect to the three key stakeholders: athlete (14), coach (21) and organisation (25). Within each of these categories, subcategories emerged: personal, interpersonal, procedural, educational, supportive, communicative and reviewing behaviours and actions that athletes, coaches and organisations can take to ease the navigation, apply consistency and establish a common ground during this challenging situation.
29	Resilience profiles of elite athletes and their associations with health-related behaviors, well-being, and performance: A latent profile analysis	Chretien, A; Hayotte, M; Vuillemin, A; Longueville, FD	PSYCHOLOGY OF SPORT AND EXERCISE	2024	10.1016/j.psychsport.2024.102689	S1	Scopus+WoS	Resilience is an important factor in the performances of elite athletes as it helps them overcome the adversity they face on a daily basis. It is a dynamic process with resilience qualities evolving throughout athletes' sporting careers. Recent research has established links between resilience, sports performance, lifestyle, and healthrelated factors. However, no study has yet focused on the resilience profiles of athletes (i.e., sub-groups of individuals with similar levels of different resilience qualities). The aims of this study were to: (a) explore the resilience profiles of elite athletes, (b) determine the characteristics of these resilience profiles (i.e., healthrelated behaviors, well-being, perceived social support), and (c) determine the correlates of these profiles at intrapersonal and interpersonal levels. Latent profile analysis was performed on 307 French elite athletes. Results revealed high resilience and moderate resilience profiles. Athletes with high resilience profiles were more likely to be men, exhibit better health-related behaviors, and have higher levels of psychological well-being and perceived performance. High levels of conscientiousness, extraversion, the dimensions of the coach-athlete relationship and the motivational climate were also related to the high resilience profile. This study is the first to explore the resilience profiles of elite athletes. The results suggest that a high level of resilience is related to the adoption of health-related behaviors favorable to elite sport. Based on these findings, health promotion programs targeting the concept of resilience should be developed in elite sport centers for athletes, coaches, and parents.

30	How is coaches' social identity leadership related to mental health in elite athletes? The mediating role of satisfaction with performance	de Subijana, CL; Pons, J; Mallett, CJ	JOURNAL OF SPORTS SCIENCES	2024	10.1080/02640414.2024.2425908	S1	Scopus+WoS	It has been found that the way high-performance coaches lead influences both athletes' performance and mental health. However, limited research exists on the Social Identity Approach to leadership in elite sports, despite growing interest in coaching science regarding interpersonal behaviours and leadership. This study investigates the empirical links between coaches' leadership, sport performance and mental health from a social identity approach. One hundred and forty-five elite athletes (Mage = 20.6 years; SD = 4.1; 62% women, 38% men) answered questionnaires measuring coaches' social identity leadership, sport performance satisfaction and mental health. The overall structural equation model (SEM) accounted for 36% and 19% of the variances in athletes' mental health and mental illness dimensions, respectively. There was a positive association between perceived coaches' social identity leadership and sport performance satisfaction, explaining 10% of its variance. Coaches' social identity leadership predicted athletes' mental health but not mental illness. Sport performance satisfaction positively influenced mental health and negatively impacted mental illness. Fostering a sense of we and us within elite sport training groups is instrumental in promoting sport performance satisfaction and, consequently, enhancing the mental health of athletes. Coaching educational courses may benefit from social identity leadership in interpersonal behaviours.
31	The successful experience of gymnastics world champion coach: an interview analysis	Liu, XX; Weng, XH; Qin, HH; Ma, S; Wang, GQ	FRONTIERS IN PSYCHOLOGY	2024	10.3389/fpsyg.2024.1405589	S1+S3	WoS	Objectives The role of a coach in enhancing athletes' performance and achieving success is well-documented across numerous studies. However, the strategies employed by Chinese coaches in developing world champion gymnasts remain under explored.Methods This research involved a single case study focusing on a coach from the Chinese National Men's Gymnastics Team, credited with nurturing eight world champion gymnasts.Results The inductive content analysis leads to that 6 subthemes, international perspective and collaborative ability, ability to control and regulate training loads, identifying athletes' needs and transforming them into motivation, goal setting aligned with athletes' abilities, adopting authoritative democratic coaching style, and establishing hierarchical-style friendship, and 3 themes, training management and planning, motivation and goal setting, and interpersonal communication are manifested. An overarching theme the successful experience of gymnastics world champion coach, is derived from the analysis.Conclusion This research bridges the gap between theoretical knowledge and practical application, offering valuable insights into the successful experiences of gymnastics world champion coaches. The findings have the potential to influence coaching methodologies globally, fostering the development of resilient, motivated, and high-performing athletes. Future research should focus on sport-specific studies, longitudinal analyses, and cross-cultural comparisons to further advance the field of sports coaching and validate the effectiveness of these innovative coaching strategies.
32	Determinants of motivation in world-class musicians and Olympic athletes: Exploring the front and the back side of the medallion	Hatfield, JL	PSYCHOLOGY OF MUSIC	2024	10.1177/03057356231155973	S1	WoS	The aim of the present case study was to obtain an in-depth understanding of the long-term motivation in four world-class musicians and two Olympic athletes. One part of the sample (Case 1) included three world famous musicians and one Olympic champion who all currently enjoy busy successful careers. These individuals are referred to as High Performing Stayers (HPS). The other part of the sample (Case 2) included one highly gifted musician and one Olympic athlete who both decided to put an end to their highly successful careers. These individuals are referred to as High Performing Quitters (HPQ). The performers' perceptions of underlying driving forces and motivational impediments on their journey toward excellence were investigated through the lens of Self-Determination Theory. Semi-structured interviews revealed conspicuous and contrasting motivational patterns throughout the development of the six performers. All the four HPS were largely driven by autonomous forms of motivation such a self-initiative, passion, curiosity and a desire for endless discovery and aspiration. In contrast, despite reaching the highest international level, the two HPQ turned out to be driven by controlled coercive forms of motivation throughout their journey toward excellence. Educational implications are discussed in relation to the main findings.
33	Factor analysis on the performance of elite male fencing athletes	Kim, HC; Park, KJ	JOURNAL OF SPORTS MEDICINE AND PHYSICAL FITNESS	2024	10.23736/S0022-4707.24.16381-5	S2	Scopus+WoS	BACKGROUND: We analyzed how stress and heart rate variability affect the performance of male fencing athletes expected to compete in various international events representing Korea. METHODS: This study included 72 fencing athletes who participated in training and competition from January 2020 to December 2023. The stress was analyzed using the Sports Competition Anxiety Test (SCAT). Heart rate variability (HRV) was measured using an autonomous neurodiagnostic device (SA-6000). Heart rate variability measurement items were time domain analysis (heart rate, standard deviation of NN interval, and root mean square of the successful differences) and frequency domain analysis (total power, low frequency, and low frequency/high-frequency ratio). All athletes were classified into two categories: victory (high performance) and defeat (low performance) groups in the recent national championships. RESULTS: In SCAT, there was no significant difference between high and low performance. After comparing HRV, it was found that there is a significant difference in heart rates (P=0.030) and low frequency/high-frequency ratio (P=0.006) between high and low performance. heart rates and low-frequency/high-frequency ratio during the high performance were observed to be higher than during low performance. CONCLUSIONS: In our study, we found that SCAT does not affect performance, while HRV has been shown to have an impact on performance. These findings can be valuable for coaches and athletes in developing strategies to improve performance results, particularly for elite male fencing athletes.

34	Orienteering Is More than Just Running! Acute Effect of Competitive Pressure on Autonomic Cardiac Activity among Elite Orienteering Athletes	Gorgulu, R; Oruç, H; Vasile, C; Corlaci, I; Voinea, F	MEDICINA-LITHUANIA	2024	10.3390/medicina60091547	S2	SPORTDiscus+Scopus+WoS	Background and Objectives: Orienteering is a sport characterized by high physical exertion and intense mental demands, which increase susceptibility to errors. Understanding the impact of such errors on psychophysiological responses, particularly on heart rate variability (HRV), is essential. This study aimed to investigate the relationship between psychophysiological indicators and checkpoint errors made by elite orienteers during official competition. Materials and Methods: Fifty-three orienteers participated in this study, and their performance was continuously monitored and recorded by using a global positioning system (GPS) and HRV data. Errors made during the orienteering events were identified and analyzed. HRV data were examined in three temporal segments: before, during, and after the identified and standardized errors. Results: The analyses indicated that errors significantly impacted HRV indices across multiple domains: the time domain, frequency domain, and nonlinear domain. Additionally, a significant effect of sex on the normalized the root mean square of successive differences (r-MSSD) before and after the error was observed. Conclusions: The findings of this study underscore the significant impact of errors made by orienteers on cardiovascular responses, as evidenced by measurable alterations in HRV metrics. Cardiovascular activity, represented by the HRV, can provide useful information for coaches and sport psychologists to adopt effective training programs for athletes.
35	The role of transformational leadership in the associations between coach-athlete relationship and team resilience: A study on elite football players	Karayel, E; Adilogullari, I; Senel, E	BMC PSYCHOLOGY	2024	10.1186/s40359-024-02043-7	S3	SPORTDiscus+Scopus+WoS	This study explores the relationship between the quality of coach-athlete relationships, transformational leadership style, and team resilience in elite football players. The coach-athlete relationship is an essential factor affecting many performance-related properties of athletes, such as mental health, psychological needs, motivation, and resilience. The study examines how the quality of coach-athlete relationships explains the team resilience feature through transformational leadership. The research group includes 210 elite players from the Super League, First League, Second League, and Third Leagues of the Turkish Football Federation (TFF) in the 2020-2021 football season. The study used descriptive and relational models to explore the current state of the dependent and independent variables and examine their relationships. The findings revealed insights into the mediating role of the coach's transformational leadership qualities, highlighting how the quality of coach-athlete relationships impacts team resilience.
36	The use of technology in the assessment and monitorization of a season in a professional padel player: A case study	Carnero-Diaz, A; Pecci, J; Reverte-Pagola, G; Da Silva-Grigoletto, ME	PROCEEDINGS OF THE INSTITUTION OF MECHANICAL ENGINEERS PART P-JOURNAL OF SPORTS ENGINEERING AND TECHNOLOGY	2024	10.1177/17543371231199812	S3	WoS	Training process in elite padel players is influenced by travels and competitions density. The irregularity in the workloads, as well as demands of the season, could affect the musculoskeletal structures. Strength training has a protective role against the injury incidence, but the competitive context does not always allow adequate periodization of training and thus achieve adaptations. The aim of this study is to analyze, using technological tools, if improvements in player's fitness are accompanied by improvements in sport performance through a case study. An elite padel player was analyzed during the 2021 season. Physical fitness was evaluated using different technological tools. Athlete monitoring was carried out using self-reported forms and sport performance was assessed through the results obtained in the World Padel Tour ranking at the end of the season. During the training process, multidimensional training was carried out in order to achieve the maximum availability of specific loads through coadjuvant training. Results of the assessment show slight improvements in all fitness tests. Assessment of sport performance reports an increased number of victories and a better position in the professional ranking. Musculoskeletal improvements helped the athlete's workload tolerance, allowing overall improvement in padel performance. The training approach from this study has shown to be effective in maintaining or even improving force-producing capacity in lower and upper limbs, force-velocity relationship, agility and sport performance, despite the high competitive density. This work provides coaches with a practical approach to assess, monitor and design a competitive season for an elite padel player.
37	The Role of Athlete Leadership Quality in the Characteristics of Team Resilience in Elite Soccer Teams: A Cross-Sectional and Longitudinal Mediation of Team Identification	López-Gajardo, MA; González-Ponce, I; García-Calvo, T; Enrich-Alturo, E; Leo, FM	JOURNAL OF CLINICAL SPORT PSYCHOLOGY	2024	10.1123/jcsp.2022-0022	S3	WoS	We present two studies examining the relationship between athlete leadership quality and team resilience and explored the mediating effect of team identification. In Study 1, 194 soccer players (Mage = 18.50, SD = 4.49) from eight national teams partici-pated. Structural equation modeling showed cross-sectionally that the four types of athlete leadership qualities were positively related to the characteristics of resilience and negatively to vulnerability under pressure. Team identification was shown to be a mediator of these relationships. Study 2, with four different time-points, involved 208 young soccer players (Mage =16.05, SD = 3.39) from two professional clubs (i.e., La Liga). Cross-lagged panel models revealed that task leadership quality (Times 1-2) was positively related to the characteristics of resilience (Times 3-4) and negatively to vulnerability under pressure (Times 3-4). However, team identification did not mediate these relationships. Therefore, practitioners should consider the perceptions of leader quality to achieve benefits during competition.

38	The Inducers of an Elite Male Table Tennis Player's Emotional Experience Throughout His Career: A Single Case Study Based on the Critical-Incident Method	Petiot, O; Kermarrec, G; Visioli, J; Martin, G	SPORT PSYCHOLOGIST	2024	10.1123/tsp.2023-0094	S3	WoS	Despite a growing interest in emotions in sport psychology, little has been written about the contextual elements triggering athletes' emotional experience. This single case study aimed to analyze the inducers of an elite table tennis player's emotional experience throughout his career. He was ranked among the 15 best players in the world, and his career spanned more than 20 years. Inspired by the critical-incident method, we conducted a lengthy interview with the player, during which he related the most significant moments of his career. The categorization of the 96 critical incidents collected highlighted four inducers of positive emotions and five inducers of negative emotions, emerging over five periods (exponential progression, first difficulties, major difficulties, second career, and end of career). These findings contribute to the development of an innovative single-casestudy design, allowing an understanding of the contextual origin of athletes' emotions over the long term. Finally, highlights are discussed and recommendations for the practice of sport psychology are formulated.
39	Integration of golf practise and strength and conditioning in golf: Insights from professional golf coaches	Bliss, A; Langdown, B	INTERNATIONAL JOURNAL OF SPORTS SCIENCE & COACHING	2024	10.1177/17479541231199657	S3	WoS	Strength and conditioning (S&C) interventions for golfers consistently demonstrate improvements in performance metrics such as clubhead speed. Golfers typically employ Professional Golfers' Association (PGA) coaches to support technical and tactical development. These coaches need to ensure golfers balance S&C training alongside competitions and technical golf practise, although no empirical data exist that demonstrates how this integration occurs practically. This study aimed to investigate the perceptions and practises of PGA Professional golf coaches and how S&C is integrated into the golf year. Forty-three (male = 35; female = 8) PGA coaches completed a mixed-methods survey with questions on themes such as coaching approach, perceptions of S&C, annual planning and goal setting. Results show that formal planning processes are highly variable or absent. The majority (n = 29; 70.7%) of PGA coaches stated they worked with an S&C coach and see the role of the S&C coach to support golfers with their input. However, when planning the season, 63.4% (n = 26) of coaches do so without input from the S&C coach. These and other related disconnects between desire for S&C intervention and inclusion of the S&C coach in planning processes were evident throughout survey responses. There may be subsequent risks of conflict or misunderstanding between PGA coach, S&C coach and golfer. It is recommended golf coaches, golfers and S&C coaches work collaboratively as a multidisciplinary support team to ensure coordinated golfer support is obtained.
40	PRE-COMPETITION TAPERING IN HIGH PERFORMANCE SPORTS -- A THEORETICAL APPROACH.	NEGRU, Ioan Niculaie ; NUŢ, Ramona Ancuţa	Studia Universitatis Babes-Bolyai, Educatio Artis Gymnasticae	2024	10.24193/subbeag.69(4).31	S2	SPORTDiscus	Aim: The aim of this study is to synthesize the literature on the 'tapering' process, i.e. reduction in training load, which takes place two to four weeks prior to a major competition. Methods: The main method used in this material is the literature review method; a total of six studies on this topic have been reviewed. Results: A synthesis of the 'tapering' process that should be implemented by every coach before a major competition in the annual competition calendar. Conclusions: In order to increase the chances of achieving high performance in major competitions, it is preferable that the training intensity during the taper be high or similar to that of the pre-taper training.
41	Coach-athlete relationship and burnout symptoms among young elite athletes and the role of mental toughness as a moderator.	Gerber, Markus ; Gygax, Basil ; Cody, Robyn	Sports Psychiatry	2024	10.1024/2674-0052/a000071	S3	SPORTDiscus	Abstract:Introduction: Social and contextual factors play an important role for elite athletes' mental health. In the present study, we examined how young elite athletes' coach-athlete relationship appraisals are associated with and longitudinally predict general and athlete-specific burnout symptoms and whether mental toughness moderates the association between the coach-athlete relationship and burnout symptoms. Methods: In this prospective study, data were collected twice within a 6 to 10-month interval. The sample consisted of 295 athletes attending Swiss Olympic Partner Schools (186 boys, 109 girls), with a mean age of 16.73±1.38 years. The following instruments were applied: Coach-Athlete Relationship Questionnaire (CART-Q), Athlete Burnout Questionnaire (ABQ), Shirom-Melamed Burnout Measure (SMBM), and Mental Toughness Questionnaire (MTQ). Cross-lagged panel analyses were performed to examine reciprocal relationships across time. Interaction effects were tested via hierarchical regression analyses. Results: Cross-sectionally, athletes who perceived their coach-athlete relationship as negative reported more general and athlete-specific burnout symptoms. Burnout symptoms were relatively stable across time, which made it difficult to explain further variance via coach-athlete relationship appraisals in the prospective analyses. While higher mental toughness scores were associated with fewer burnout symptoms in the cross-sectional analyses, higher mental toughness did not moderate the association between the coach-athlete relationship and burnout symptoms. Conclusion: The coach-athlete relationship plays an important role in athletes' mental health. Therefore, coaches need the ability to develop effective relationships with their athletes, including thoughtful and respectful communication about issues specific to sport and life generally. ABSTRACT FROM AUTHOR

42	The under representation of women coaches in high-performance sport	Serpell, BG; Harrison, D; Dower, R; Cook, CJ	INTERNATIONAL JOURNAL OF SPORTS SCIENCE & COACHING	2023	10.1177/17479541231160229	S1	WoS	High-performance sport is still not appropriately addressing issues that perpetuate women's underrepresentation in elite sport coaching and leadership. Therefore, it is also unlikely representation for other minority groups in high-performance sport will be achieved any time soon. For high-performance sport to progress, there is an opportunity to create environments that foster a variety of coaching styles with diverse racial, ethnic and gender identities. This paper points to statistics from elite football and Olympic sports to highlight the issue of the underrepresentation of women coaches in high-performance sport and draws upon a range of literature to inform an evidence-based and strategic approach to designing development in the context of increasing coach and leader gender diversity in these settings. It explores leadership in high-performance sport, learning and development, and high-performance sport environments. We argue future focused development should prepare high-performance sport leaders to intervene at an environmental level where they provide leadership, facilitate performance enablers, and engage athletes and performance staff to shape cultural change
43	Interpersonal Behaviors Questionnaire in Sport: Psychometric Analysis With Romanian Professional Athletes	Alexe, DI; Cîrtita, BC; Tohanean, DI; Larion, A; Alexe, CI; Dragos, P; Burgueño, R	PERCEPTUAL AND MOTOR SKILLS	2023	10.1177/00315125221135669	S1	Scopus+WoS	Building upon self-determination theory, our objective in this research was to adapt and analyze psychometrically the Interpersonal Behaviors Questionnaire (IBQ) in sport. Our participants were 642 professional athletes (55.14% men, M- age = 22.81) who completed an online survey measuring their perception of coaching behaviors, need satisfaction, need frustration, and motivation. The results showed a good fit for the 24-item IBQ with a six-factor correlated model (chi(2)/df = 4.178; CFI = .925; TLI = .912; SRMR = .048; RMSEA = .070), and we obtained evidence supporting its convergent and discriminant validity. The analyses also underpinned measurement invariance across gender, age, and sport, and confirmed good reliability. Criterion validity was met by positive associations of autonomy-supportive, competence-supportive, and relatedness-supportive behaviors with need satisfaction and autonomous motivation; and of autonomy-thwarting, competence-thwarting and relatedness-thwarting behaviors with need frustration, controlled motivation and amotivation. The adapted IBQ can be applied to the assessment of professional Romanian athletes' perceptions of need-supportive and need-thwarting coaching behaviors.
44	The impact of training environment on Tokyo Olympic athletes' wellbeing: a qualitative multiple case study	Paoletti, V; Mayglothling, T; Mills, JP	INTERNATIONAL JOURNAL OF SPORT PSYCHOLOGY	2023	10.7352/IJSP.2023.54.348	S1	WoS	This study explores the influence that the training environments have on the wellbeing of Tokyo Olympic athletes. The staff/athlete relationships and the Olympic Organizations can influence the sport performance and the athletes' wellbeing. A qualitative multiple case study was conducted on an anonymous sample of six Olympic level female athletes through the web software Chatplat. Reflexive thematic analysis was employed to analyze the data. Results were categorized into the following three general dimensions: training environments, relationships' influence on well-being, and organizational environments. The results are presented to demonstrate the negative and positive factors that affect the performance and the psychological well-be-ing of the athletes. The current study offers an original and significant contribution to the sport psychological literature by providing a unique methodology in addition to real time insight into the dynamic nature of well-being in Olympic athletes. Limitations and recommendations of the study are discussed for future research.
45	Inescapable tensions: performance and/or psychological well-being in Olympic and Paralympic athletes during sport disruption	Trainor, LR; Bennett, EV; Bundon, AM; Tremblay, M; Mannella, S; Crocker, PRE	QUALITATIVE RESEARCH IN SPORT EXERCISE AND HEALTH	2023	10.1080/2159676X.2023.2175899	S1	WoS	
46	Lifestyle challenges and mental health of professional tennis players: an exploratory case study	Shrom, SJ; Cumming, J; Fenton, SJ	INTERNATIONAL JOURNAL OF SPORT AND EXERCISE PSYCHOLOGY	2023	10.1080/1612197X.2022.2099947	S1	WoS	The professional tennis tour lifestyle has become increasingly difficult over time. Although the financial strain of tour life has been identified [International Tennis Federation. (2017, March 30). ITF pro circuit review. https://www.itftennis.com/procircuit/aboutpro-circuit/player-pathway.aspx], there is a gap in our understanding of the additional lifestyle challenges that professional players face and the potential impact these have on their progression and mental health. Adopting an exploratory case study methodology [Yin, R. K. (2017). Case study research and applications: Design and methods. Sage.], with Behind the Racquet, a social media platform aimed at raising awareness of professional players' challenges as its case, and theoretically underpinned by [Maslow, A. H. (1943). A theory of human motivation. Psychological Review, 50(4), 370-396.] hierarchy of needs, this study aimed to gain a contextual understanding of these challenges. Using Behind the Racquet as the data source provided a unique opportunity to gain authentic, self-reported experiences from a hard-to-reach population. The sample consisted of 65 professional players (33 males, 32 females; age range = 18-46 years; mean age = 27.34 years) from 28 different countries. Players achieved varying levels of success, including being Grand Slam and Olympic champions, ATP, and WTA Top 10 players, and within and outside of the world's Top 100. Findings illustrated physical and mental fatigue, financial imbalance of the professional system, the social and psychological impact of living a nomadic existence, the weight of expectation, structural-caused instability, and mental illness as key challenges that players experience on the professional tour. These challenges, which inhibit players' ability to meet basic needs on each level of [Maslow, A. H. (1943). A theory of human motivation. Psychological Review, 50(4), 370-396.] hierarchy of needs, pose concerns to both players' progression on tour and their mental health.

47	Heart Rate Variability in Elite International ITF Junior Davis Cup Tennis Players	Villafaina, S; Crespo, M; Martinez-Gallego, R; Fuentes-Garcia, JP	BIOLOGY-BASEL	2023	10.3390/biology12010017	S2	WoS	Simple Summary The precompetitive anxiety should be controlled in athletes to optimize their performance. In this regard, heart rate variability (HRV) is a non-invasive tool to assess the autonomic nervous system balance. Therefore, this study aims to investigate the HRV response of elite international junior tennis players during an international tournament. Eleven players participate in this study, with a mean age of 15.36 (0.67) years old. The HRV of tennis players are assessed 24 h before the event, and immediately before the first and the second match of the ITF Junior Davis Cup event. Each of the registers lasted 5 min and the time domain, frequency domain, and non-linear variables were extracted. Results show that elite tennis players did not decrease the HRV between the baseline and the first match. However, the HRV response significantly differed between the baseline and the second match. Nevertheless, anxiety was higher before the first match than before the second match. Coaches and physical trainers could use HRV to control and manage the precompetitive anxiety of junior tennis players. The experience in international tournaments and the familiarization with the environment in the second match could explain these results. The aim of the present study was to investigate the evolution of the HRV during an international team tennis tournament in elite junior tennis players. A total of 11 tennis players, with a mean age of 15.36 (0.67) from six nationalities, participated in this cross-sectional study. Participants were measured one day before the event, before the first match, and before the second match of the ITF Junior Davis Cup event. Each of the registers lasted 5 min and the time domain, frequency domain and non-linear variables were extracted. The tournament took place in Valencia (Spain). Results showed that there was not a significant decrease in the heart rate variability (HRV) induced by precompetitive stress. However, significant differences (p-value < 0.05) were found between baseline and second match pre-competition HRV values in low frequency (LFNu) (p-value = 0.008) and high frequency (HFNu) power (p-value = 0.008), LF/HF ratio (p-value = 0.008) and Sample Entropy (SampEn) (p-value = 0.033) variables. Furthermore, cognitive anxiety (measured by CSAI-2r) (p-value = 0.017) and anxiety (measured by STAI-S) (p-value = 0.042) were higher before the first match than before the second match. Coaches and physical trainers could use HRV to control and manage the precompetitive anxiety of junior tennis players. The
48	Balancing risk-taking and self-care: The ecology of athlete health behaviour during the Olympic qualification phase	Schubring, A; Hallten, M; Barker-Ruchti, N; Post, A	INTERNATIONAL REVIEW FOR THE SOCIOLOGY OF SPORT	2023	10.1177/10126902231168098	S3	WoS	Athletes who aim to qualify for the Olympic Games need to stay healthy. Research demonstrates, however, that many elite athletes take health risks to achieve sporting success. Drawing on social ecological thinking, the purpose of this study is to understand change in athlete health behaviour during the Olympic Games qualification phase. We draw on data from a six-month-long case study on four athletes who aimed to qualify for the 2016 Olympic Games in Rio. We used semi-structured interviews and weekly-online surveys to collect data. Olympic hopefuls engaged in both risky health behaviour, such as 'competing while injured', and in self-caring health behaviour, such as 'balanced dietary management'. Risk-taking was encouraged by a habit of taking pain medication, insufficient medical support, and time pressure to qualify. Awareness for self-care, a trusting coach-athlete relationship, and a well-rounded athlete support programme were factors that promoted self-care. Conceptually, we found that athlete health behaviour is dynamic and ecological, that is, it is relational to personal, contextual, and temporal factors. Based on the findings, we advise Olympic hopefuls to surround themselves with people who support caring health behaviour, and that sport organisations and stakeholders adopt long-term planning and implement policies of care.
49	Thinking About Elite Performance: The Experience and Impact of Mental Fatigue in Elite Sport Coaching	Russell, S; Halson, SL; Jenkins, DG; Rynne, SB; Roelands, B; Kelly, VG	INTERNATIONAL JOURNAL OF SPORTS PHYSIOLOGY AND PERFORMANCE	2023	10.1123/ijspp.2023-0033	S3	WoS	Purpose: Mental fatigue causes decreases in aspects of athletes' performance. Elite coaches commonly undertake cognitively demanding tasks and are seemingly at similar risk of subsequent performance impairment. However, elite sport coaches' experiences of mental fatigue, alongside other markers of psychobiological stress, have yet to be quantified. Methods: Three elite coaching and performance staff (2 women and 1 man) provided 100-mm visual analog scale ratings of mental fatigue, physical fatigue, readiness to perform, and salivary samples for later cortisol (sCort) and alpha-amylase (sAA) analysis. Data were obtained on the same morning each week across a 16-week preseason. Data were subset by individual coach for descriptive and repeated-measures correlational analyses. Results: Fluctuating mental fatigue was observed over the 16 weeks (min-max; coach 1 = 25-86 AU; coach 2 = 0-51 AU; and coach 3 = 15 - 76 AU). Elevated levels of mental fatigue were reported at multiple time points, with individual variability observed. sCort (in nanomoles per liter), sAA (in micromoles per liter), and sAA:sCort indicated that coaches experienced psychophysiological stress (min-max; coach 1 sCort = 8.42-17.31, sAA = 52.40-113.06, sAA:sCort = 3.20-12.80; coach 2 sCort = 4.20-9.70, sAA = 158.80-307.20, sAA:sCort = 21.10-61.70; and coach 3 sCort = 6.81-19.66, sAA = 86.55-495.85, sAA:sCort = 4.90-35.50). A significant inverse relationship between mental fatigue and readiness to perform (r = -.44 [-.64 to -.17], P = .002) was identified. Conclusions: Elite sport coaches report elevated instances of mental fatigue during a preseason training period. Those involved in elite sports should act to understand the presence and potential subsequent impacts of staff mental fatigue and consider management or mitigation strategies. Optimization of the cognitive performance of coaches and performance staff presents as a potential source of competitive advantage.

50	Are Metabolic Power Distribution and Accelerometer-Based Global Positioning System Variables Associated With Odds Ratios of Noncontact Injuries in Professional Soccer Players?	Nobari, H; Alves, AR; Abbasi, H; Khezri, D; Zamorano, AD; Bowman, TG	JOURNAL OF STRENGTH AND CONDITIONING RESEARCH	2023	10.1519/JSC.0000000000004475	S3	WoS	The present study was intended to i) investigate the relationship between metabolic power average (MPA), acceleration (AcZ) and deceleration (DcZ) zones, and their differences (Delta) on 3 load levels with noncontact injuries in professional players throughout a full soccer season and ii) to analyze the injury risk associated between high-load versus low-load levels for each of the aforementioned parameters with odds ratios (OR) and relative risk (RR), respectively. Twenty-one professional soccer players (age = 28.3 +/- 3.9 years) were monitored during a full season (48 weeks) through global positioning system (GPS). A relationship between MPA and accelerometer-based GPS, mainly in explosive actions (i.e., AcZs and DcZs), was found. A higher incidence of injuries in the high-load weeks compared with the low-load weeks were reported (mainly in MPA, AcZ1, AcZ2, and DcZ3 variables). Moreover, significant means of OR (mean = 4.3) and RR (mean = 2.6) of noncontact injuries were established in intense periods with higher metabolic load (i.e., power accelerations, AcZ1, x(2) = 0.022). Our results may be useful for coaches, sports scientists, and researchers regarding the optimization of the athletes' performance, as well as providing insights about the impact of intense exercise.
51	Multilevel Analysis of Coach Leadership, Group Cohesion and Collective Efficacy in Semiprofessional Football Teams	Leo, FM; González-Ponce, ; Pulido, JJ; López-Gajardo, MA; García-Calvo, T	INTERNATIONAL JOURNAL OF SPORT PSYCHOLOGY	2022	10.7352/IJSP.2022.53.378	S1	WoS	This study examined how perceptions of leadership behavior in semi-professional sport predicted group cohesion and collective efficacy in a sample of Spanish football players. We adopted a longitudinal perspective, taking measures at the beginning, the middle, and the end of a sport season, and three levels of analyses were specified: intra-individual, inter-individual, and inter-team level. Multilevel modeling analysis showed that perceptions of positive leadership behaviors, such as training instruction, social support, positive feedback, and democratic behaviors, were a stronger predictor of a high level of cohesion and collective efficacy than were perceptions of coaches' autocratic behavior. Predictive effects differed as a function of time, level of analyses and the outcome variable under investigation. The results indicate the importance of considering the coaches' leadership styles when examining group cohesion and collective efficacy at different levels of analyses in semi-professional sports.
52	Desired coaching behaviours of elite divers during competition	Henderson, S; Bloom, GA; Alexander, D	INTERNATIONAL JOURNAL OF SPORT AND EXERCISE PSYCHOLOGY	2022	10.1080/1612197X.2021.2003417	S1	WoS	A great deal of research on coach-athlete interactions during competition has focused on team sports, something which does not consider the many contextual differences between team and individual sports. Diving is an individual sport where recurrent intermittent breaks between dives in competition provide athletes and coaches with opportunities to communicate and interact during an event. The purpose of this study was to understand elite divers' perspectives of their coaches' behaviours during competition. Data were collected through semi-structured interviews and documents with six national team springboard and platform divers. A reflexive thematic analysis of the data revealed that athletes and coaches carefully developed and implemented individualised routines to deal with the pressures of competition. Athletes discussed the importance of trust and understanding with their coaches as crucial elements to facilitate performance in a high-risk sport. Athletes also found it particularly challenging when they felt their coaches were frustrated, disappointed, or giving up on them. From a practical perspective, our findings are useful for coaches and athletes in similar sports with intermittent breaks throughout the long day of competition, such as track and field, snowboarding, freestyle skiing, and weightlifting.
53	An Evidence-Informed Framework to Promote Mental Wellbeing in Elite Sport	Purcell, R; Pilkington, V; Carberry, S; Reid, D; Gwyther, K; Hall, K; Deacon, A; Manon, R; Walton, CC; Rice, S	FRONTIERS IN PSYCHOLOGY	2022	10.3389/fpsyg.2022.780359	S1	WoS	Elite athletes, coaches and high-performance staff are exposed to a range of stressors that have been shown to increase their susceptibility to experiencing mental ill-health. Despite this, athletes may be less inclined than the general population to seek support for their mental health due to stigma, perceptions of limited psychological safety within sport to disclose mental health difficulties (e.g., selection concerns) and/or fears of help-seeking signifying weakness in the context of high performance sport. Guidance on the best ways to promote mental health within sporting environments is increasing, though current frameworks and position statements require greater focus on a whole of system approach, in which the needs of athlete, coaches and high-performance staff are considered within the context of the broader ecological system in which they operate and perform. This paper synthesizes existing research, reviewed for translatability by mental health professionals working in elite sport, to provide an evidence-informed framework with real world utility to promote mentally healthy environments for all stakeholders in elite sporting organizations, from athletes through to administrators. Recommendations are provided to positively impact the mental wellbeing of athletes and support staff, which may in turn influence athletic performance. This framework is intended to provide sporting organizations with evidence-informed or best practice principles on which they can develop or progress their policies to support mental health promotion and prevent the onset of mental health difficulties. It is intended that the framework can be adapted or tailored by elite sporting organizations based upon their unique cultural, contextual and resourcing circumstances.

54	Well-being in elite sport: The specific role of sport-related demands, resources, and recovery from sport	Balk, YA; de Jonge, J; Geurts, SAE	GEDRAG & ORGANISATIE	2022	10.5117/Go2022.3.001.BALK	S1	WoS	Similar to employees, elite athletes are exposed to different job demands. In this article we discuss the role of both situational determinants (sport -related demands and resources) and individual determinants (detachment) of well-being in elite sport. The research was conducted while taking into account physical as well as cognitive and emotional dimensions of sport-related demands and resources, detachment, and well-being. Four sub-studies of a doctoral dissertation are discussed (a psychometric study, a cross-sectional study, and two diary studies), which together show that it is both useful and necessary to differentiate between the physical, cognitive, and emotional nature of demands, resources, and detachment from sport. Moreover, the relations of sport-related demands and resources with elite athletes' well-being are highly similar to the relations of work-related demands and resources with employee well-being. As such, theories and models in the domain of W/O psychology have the potential to assist elite athletes in their quest for healthy and optimal performance.
55	Predicting sports performance from well-being: A mapping of professional athletes', amateur athletes' and non-athletes' positions	Fruchart, E; Rulence-Pâques, P	EUROPEAN REVIEW OF APPLIED PSYCHOLOGY-REVUE EUROPEENNE DE PSYCHOLOGIE APPLIQUEE	2022	10.1016/j.erap.2022.100793	S1	WoS	Introduction. - Prediction of performance and well-being are two essential keys in sports performance.Objective. - The study aimed to map different positions regarding the way in which individuals integrated mentally five elements of eudaimonic well-being (relatedness, autonomy, competence, mental vitality and physical vitality) for predicting the degree of performance at the beginning of a match.Method. - Twenty-four non-athletes, 44 amateur athletes and 26 professional athletes indicated their prediction of performance in 32 scenarios constructed from the combination of these elements of well-being. Cluster analysis, ANOVAs, and chi-square test have been done.Results. - Two positions on prediction of performance were found. The first cluster was called Sometimes Performance Prediction and the second cluster was named Seldom Performance Prediction. The five elements had a positive effect on the prediction of performance. The composition of both clusters was linked to the level of involvement in sport.Conclusion. - Offering athletes a well-being environment may lead to predict the sports performance.(c) 2022 Elsevier Masson SAS. All rights reserved.
56	Orientation and motivational climate in elite handball players: Multivariate modeling of performance	Kristjánsdóttir, H; Matthíasdóttir, A; Saavedra, JM	NORDIC PSYCHOLOGY	2022	10.1080/19012276.2021.1922304	S1	SPORTDiscus+WoS	This study's objectives were to analyse goal orientation and motivational climate in elite handball players as functions of age and gender; and construct a multivariate model explaining handball performance from a motivational perspective (orientation and climate). The participants were 174 national team handball players. They were categorized in accordance with the official age groups. Two questionnaires were used: Task and Ego Orientation in Sport Questionnaire and the Perceived Motivational Climate in Sport Questionnaire-2. A one-way ANOVA (Bonferroni post-hoc correction) was used to examine differences between teams for each gender. Discriminant analyses were performed to classify the participants of each team into two groups according to their performance level (starters and non-starters). There were no age or gender differences found in goal orientation, and very few differences in motivational climate. Except for the women's U19, U17, and U15 teams, it was possible to predict the performance level (starters and non-starters) in the different age and gender groups, achieving correct classification (55% to 93%). The two variables that were repeated most often (thrice) in the models were ego orientation and task cooperative learning. Coaches might focus on looking for handball players with ego orientation, while a climate of task-oriented motivation should predominate in training sessions.
57	Creating an environment for thriving: An ethnographic exploration of a British decentralised Olympic and Paralympic Sport Organisation	Passaportis, MJR; Brown, DJ; Wagstaff, CRD; Arnold, R; Hays, K	PSYCHOLOGY OF SPORT AND EXERCISE	2022	10.1016/j.psychsport.2022.102247	S1	WoS	Objectives: The aims of this study were to explore the features of the athletic environment that influence thriving within a British Olympic and Paralympic sport organisation and to understand the interconnectedness of these factors across a range of individuals and contexts. These aims were pursued within a decentralised organisation that was undergoing a leader-led cultural change strategy. Method: To develop an understanding of the environmental factors that facilitate athlete thriving, a 16-month ethnography was conducted. Data analysis consisted of reflexive thematic analysis of observational notes, reflexive diaries, and interview transcripts. The findings are presented in an ethnographic tale. Results: Alongside the complexities of implementing a culture change strategy across a decentralised organisation, the ethnographic tale details three key features of the athletic environment targeted by senior leadership to successfully influence the athletes' ability to thrive within their silos. Underpinning these factors are three interconnected themes of understanding, openness, and trust. Conclusions: This study demonstrates how empowering devolved leadership was impactful for organisational culture by reducing the homogeneity of leader-centric change initiatives. Further, harnessing an organisationwide commitment to promoting relationships founded on understanding, openness, and trust can create athletic environments that facilitate thriving. Therefore, while a decentralised structure may present challenges for promoting a duty of care, it is possible to create an environment that supports athletes to thrive.

58	Satisfaction with life in Special Olympic athletes: the role of autonomy support and basic need fulfilment	Komenda, S; Springstein, T; Zrnic, I; Zeilinger, E; Franken, F; Weber, G	INTERNATIONAL JOURNAL OF DEVELOPMENTAL DISABILITIES	2022	10.1080/20473869.2021.1917110	S1	Scopus+WoS	Aims: Athletes in the general population report higher satisfaction of basic needs when coaches are providing an autonomy supportive sport climate (ASSC). Our study aims at investigating whether ASSC is associated with satisfaction with life in athletes with intellectual disabilities (ID) and whether this association is mediated by basic need satisfaction. Method: During the Special Olympics World Winter Games 2017, 168 athletes with ID (M = 33.86 years; SD = 10.47) completed questionnaires measuring ASSC, basic need satisfaction (autonomy, competence, and relatedness), and satisfaction with life. Multiple linear regression analyses and mediation analyses were performed. The mediation model was controlled for the potential impact of participating in team vs. individual sports. Additionally, gender effects were explored. Results: ASSC was significantly associated with satisfaction with life (beta = .38, p < .001). This association was mediated by competence (indirect effect: ab(1) = .15; CI [.05; .32]) but not by autonomy or relatedness. No effects were found related to participating in team vs. individual sports or gender. Conclusions: Our study provides evidence that an ASSC is associated with athletes perceiving themselves as more competent and reporting more satisfaction with life.
59	Stakeholder insights into athlete attrition in the high-performance pathway	Guevara, SA; Donaldson, A; Keegan, RJ; Appaneal, RN; Smyth, EA; Waddington, G; Mahony, K; Drew, MK	JOURNAL OF SCIENCE AND MEDICINE IN SPORT	2022	10.1016/j.jsams.2022.05.011	S2	Scopus+WoS	Objectives: Primary: To gain a system-wide perspective on factors leading to athlete attrition from a highperformance sport system (HPSS).Secondary: To identify what a sample of system-wide stakeholders and past athletes value as the most important and feasible attrition factors to address to retain talented athletes.Design: Mixed-methods.Methods: Concept mapping was used for qualitative data collection and quantitative data analysis. Sixty-one participants including: (i) past athletes from an Australian state sporting institute; (ii) their families; and (iii) internal and external stakeholders to a HPSS who supported past athletes.Results: Participants brainstormed 83 unique statements (i.e. attrition factors) that were mapped into 13 clusters of attrition factors following multidimensional scaling and hierarchical cluster analysis performed on the participants sorting data: 'abuse and mismanagement of health'; 'athlete health'; 'limited support/resourcing'; 'coaching'; 'inconsistent processes'; 'financial and career support'; 'pathway structure'; 'organisational dynamics'; 'competitive stress'; 'performance potential'; 'challenges with selection and transition'; 'psychological state'; and 'competing non-sport priorities'. 'Abuse and mismanagement of health' had the highest mean importance (3.76 out of 5) and feasibility (3.31) rating. The 13 clusters were further grouped into four overarching domains: 'sport system policy, structure and processes'; 'pathway structure, transition and support'; 'individual athlete health and capability'; and 'whole-of-life demands and priorities'. The domain 'sport system policy, structure and processes' contained the most important and feasible clusters.Conclusions: Macro (system-level) and micro (intrapersonal and interpersonal) level athlete attrition factors should be considered together. Athlete health was considered the most important athlete retention issue to address. (c) 2022 Published by Elsevier Ltd on behalf of Sports Medicine Australia.
60	Rapid weight loss among elite-level judo athletes: methods and nutrition in relation to competition performance	Stangar, M; Stangar, A; Shtyrba, V; Cigic, B; Benedik, E	JOURNAL OF THE INTERNATIONAL SOCIETY OF SPORTS NUTRITION	2022	10.1080/15502783.2022.2099231	S2	Scopus+WoS	Background Rapid weight loss (RWL) followed by rapid weight gain (RWG) is a regular pre-competition routine in combat sports and weightlifting. With the prevalence of these sports exceeding 20% at the 2020 Tokyo Olympics, there are limited data on RWL and RWG practices and their impact on well-being and competitive success in elite-level athletes. Methods A total of 138 elite-level female and male judokas, 7.7% of the athletes ranked as top 150 on the International Judo Federation Senior World Ranking List (WRL), completed a survey on RWL, RWG, and the consequences of these practices. Results Our findings showed that 96% of the respondents practice RWL. The average reduced body mass percentage was 5.8 +/- 2.3%. Respondents who used either of the dehydration methods - fluid restriction, sauna suit, and/or sauna/hot bath - to reduce weight were 88%, 85%, and 76%, respectively. Furthermore, 91% of the respondents reported reduced energy as a negative consequence of RWL and 21% experienced a collapse episode during the RWL period. Respondents ranked 1-20 on the WRL experienced fewer negative consequences of RWL and RWG (p = 0.002) and had more dietitian and/or medical doctor support (p = 0.040) than lower-ranked respondents. Those who started with RWL practices before the age of 16 (38%) were ranked lower on the WRL (p = 0.004) and reported more negative consequences of RWL and RWG (p = 0.014). Conclusions This study is the first to provide insight into the RWL practices of worldwide elite-level judokas and provides valuable information for the combat sports society, especially coaches. Proper weight management and optimal timed initiation of RWL practices in a judoka's career may contribute to success at the elite level.

61	The prevalence of pre-conditioning and recovery strategies in senior elite and non-elite amateur boxing	Finlay, MJ; Page, RM; Greig, M; Bridge, CA	PHYSICIAN AND SPORTSMEDICINE	2022	10.1080/00913847.2021.1931525	S2	Scopus+WoS	Objectives A variety of acute strategies around competition are used to prepare and promote physical and mental restoration in athletes. However, to date, no research exists on the prevalence of such methods in amateur boxing. Therefore, this study aimed to 1) examine the prevalence of pre-conditioning and recovery strategies in amateur boxing; 2) ascertain whether this was discriminated against at competitor level. Methods This study surveyed 101 senior amateur boxers (Senior Elite SEB n = 59; Senior Development SDB n = 42), on their application and perceptions of pre-conditioning and recovery strategies. Results The reported findings determined a significantly greater number of SEB performed resistance priming activity up to 48 hours prior to competition (11, 19% vs 2, 5%; P = 0.040), and post-activation performance enhancement (PAPE) activity in the pre-competition warm-up (18, 31% vs 1, 2%; P < 0.001), compared to SDB. Likewise, SEB reported they were also significantly more likely to utilize massage (SEB 35, 59%, SDB 11, 29%, P = 0.001) and cold-water immersion (CWI) (SEB 28, 47%, SDB 10, 29%, P = 0.016) as recovery modes, compared to their SDB counterparts. Conclusions This study was the first to provide data on the use of PAPE, priming and recovery methods around amateur boxing bouts. Increased access to multi-disciplinary staff could be expected in SEB, possibly explaining the greater prevalence of evidence-based methods around competition. Once athlete responsiveness to PAPEand longer-term priming methods are initially assessed, these strategies could be implemented to improve punch-specific performance, though more research is needed on their efficacy. Likewise, boxers could utilize evidence-based recovery modes where possible, with increased importance during repeat-bout scenarios, such as domestic tournaments. Coaches and practitioners may use this data to implement pre-conditioning and recovery strategies, to optimize performance and reduce the risk of injury of amateur boxers.
62	An exploration of reciprocity between female athletes and their coach in elite junior swimming: a shared reality theory perspective	Solstad, BE; Granerud, M; Haraldsen, HM; Gustafsson, H; Knight, CJ	QUALITATIVE RESEARCH IN SPORT EXERCISE AND HEALTH	2022	10.1080/2159676X.2021.1941211	S3	WoS	Based on the proposition that the relationship between a coach and athlete is at its best when both parties contribute to enhancing its quality, the present study sought to investigate if Shared Reality Theory could provide new insights on the topic. Specifically, the purpose of the present study was to explore: (a) how a shared reality is established, or fails to be established, over the course of the sporting partnership between athletes and their coach; and (b) how the presence of a shared reality (or not) in the coach-athlete relationship is related to the experienced quality of the relationship between athletes and their coach, as recorded over time. Narrative inquiry, embedded within a longitudinal qualitative approach, was adopted. Six female elite junior swimmers and their head coach completing three interviews each over a 9-month period. Data were analysed using narrative thematic analysis, leading to the development of two narratives: A prevention-oriented narrative and a promotion-oriented narrative. Overall, the findings suggests that athletes who experience a shared reality with their coach are more motivated and report a higher sense of psychological well-being. To establish the experience of a shared reality, reciprocal and honest communication motivated by trust in the other is needed. Due to power imbalance, it is deemed important for coaches to be supportive and trustworthy to encourage athletes to communicate with them, so the coach and his/her athletes can work together in a synergistic manner.
63	Mindful self-reflection to support sustainable high-performance coaching: A process evaluation of a novel method development in elite sport	Hägglund, K; Kenttä, G; Thelwell, R; Wagstaff, CRD	JOURNAL OF APPLIED SPORT PSYCHOLOGY	2022	10.1080/10413200.2021.1925782	S3	Scopus+WoS	High-performance coaches (HPCs) operate in complex and unpredictable environments wherein sustainable performance and well-being are regularly challenged. The purpose of this study was to develop, through an iterative process of data collection, evaluation and action, a brief SMS-based mindful self-reflection intervention to support sustainable high-performance coaching. We present this work through the form of a process evaluation. After an initial pilot intervention, three subsequent phases of development took place with 18 HPCs from athletics and figure skating. In each phase, HPCs completed a daily or weekly brief mindful self-reflection SMS-intervention for 8 weeks prior to taking part in a focus group interview and 6-month or 12-month follow-up. The results of the process evaluation demonstrate exceptionally high fidelity, reach and perceived value of the intervention for the HPCs. The HPCs also perceived the intervention to influence key mechanisms for a sustainable profession such as greater engagement in their well-being (e.g., self-awareness, helpful perspective on vulnerability and self-compassion). Moreover, lasting behavior changes associated with sustainability were reported at 6- and 12-month follow-ups. This process evaluation reflects a rigorously developed and novel procedure for the delivery of a brief mindful self-reflection intervention and appears easy to use by HPCs in their demanding roles. Lay summary: We present a process evaluation of a mindful self- reflection intervention developed to foster sustainable high-performance coaching. Over four development phases we refine the SMS-based intervention and report exceptionally high fidelity, reach and perceived value among the participants. At 6- and 12-month follow-ups participants reported lasting behavior changes associated with sustainability.IMPLICATIONS FOR PRACTICE This brief SMS-based mindful self-reflection intervention facilitated healthy perspectives on vulnerability and help-seeking behavior and prompted behavior change aligned with self-compassion and well-being at 6- and 12-months follow-ups. Hence, the SMS intervention may provide practitioners with a tool for supporting sustainability among high-performance coaches. The process evaluation presented here demonstrated exceptionally high fidelity with the simplicity of the SMS intervention seemingly key to this outcome. Moreover, receiving positive reinforcement throughout the intervention and having opportunities to share their experiences in focus group interviews influenced the

64	Stroke Kinematics, Temporal Patterns, Neuromuscular Activity, Pacing and Kinetics in Elite Breaststroke Swimming: A Systematic Review	Nicol, E; Pearson, S; Saxby, D; Minahan, C; Tor, E	SPORTS MEDICINE-OPEN	2022	10.1186/s40798-022-00467-2	S3	WoS	Background Breaststroke is a technically complex stroke characterised by discontinuous propulsive phases, large intracyclic velocity variation and low mean velocity. The performance of this stroke at an elite level is influenced by a number of biomechanical, physiological and psychological factors. The present systematic review aimed to synthesise the biomechanical factors influencing elite breaststroke swimming performance. This review aims to provide elite coaches and performance scientists with a breadth of knowledge from which training and racing interventions can be developed. Methods Electronic searches of Medline, Scopus and SPORTDiscus databases were conducted in May 2020 and March 2022. Search results that were peer-reviewed, published in English and published during or after the year 2000 were considered for review. The methodological rigour of studies was assessed using a risk of bias scale previously used for the evaluation of sports science research. Results Thirty-eight articles were included in the present review. Articles investigated elite breaststroke performance in relation to one of the following areas: stroke kinematics, temporal patterns, neuromuscular activity, pacing and kinetics. Discussion Kinematic, temporal and neuromuscular activity comparisons between groups of various race distance, performance or experience level, and athlete sex were common in the literature. These analyses demonstrated differences in stroke rate, stroke length, propulsive time, recovery time, glide time, sum of total integrated EMG and triceps brachii activation patterns between groups. The evaluation of various pacing strategies, and the relationship between kinetics and breaststroke performance was comparatively rare within the literature. Further research into the relationship between kinetics and breaststroke performance, and the manipulation of pacing strategy would increase the breadth of knowledge from which coaches and performance scientists can develop evidence-based training and racing interventions.
65	The quest for professional self-understanding: Sense making and the interpersonal nature of applied sport psychology practice	Haluch, P; Radcliffe, J; Rowley, C	JOURNAL OF APPLIED SPORT PSYCHOLOGY	2022	10.1080/10413200.2021.1914772	S3	Scopus+WoS	Several authors within the sport psychology literature have acknowledged an interpersonal dimension of applied practice, with several publications highlighting the significance of developing and maintaining effective working relationships with a range of key stakeholders (i.e., athletes, managers, coaches, sports physicians, parents). Through the use of a longitudinal series of semi-structured interviews across an eighteen month period, this article explores the experiences of four trainee practitioners and two qualified sport psychologists, and their perceptions of working with multiple stakeholders, examining how such encounters serve to shape and inform their professional self-understanding in a contextual manner. Key themes constructed through Interpretative Phenomenological Analysis accounted for finding one's place, picking your battles and being seen to do a good job. Upon acknowledgement of self-perception and recognition from others as being fundamental factors in shaping a practitioner's professional self-understanding, the present article encourages early career practitioners, and the professional bodies who train them, to increase their engagement with other fields and professions, with a view to better preparing sport psychologists for the everyday interpersonal demands of successful applied practice. Lay summary: This article explores trainee and qualified practitioners' experiences and reflections of working with multiple stakeholders to better prepare sport psychologists for everyday interpersonal demands of successful applied practice. IMPLICATIONS FOR PRACTICE The current article Furthermore highlights the inherently micropolitical nature of applied sport psychology contexts, illuminating the significant influence that interpersonal relationships with key contextual stakeholders can have upon a practitioner's professional self-understanding. The current article serves to support practitioners' efforts in seeking to better manage their interpersonal relationships with key stakeholders, enhancing their ability to survive and thrive as applied practitioners. The novel theoretical lens adopted within this study, provides a basis to help scaffold developmental activities relating to professional self-understanding, micropolitical activity, and contextual understanding.
66	Implementing Skill Acquisition Research in High-Performance Sport: Reflecting on the Importance of Autonomy-Support for Successful Collaboration.	Morris-Binelli, Khaya ; Müller, Sean ; van Rens, Fleur E. C. A. ; Staniforth, Dave ; Appleby, Brendyn ; Rosalie, Simon M.	Journal of Sport Psychology in Action	2022	10.1080/21520704.2021.1964661	S1	SPORTDiscus	Perceptual-cognitive-motor skills, such as visual anticipation, are pivotal for superior performance in sport. However, there are only a limited number of skill acquisition specialists working with coaches to develop these skills in athletes. The purpose of this paper is to present a brief reflection on the use of psychological strategies to create an autonomy-supportive environment to embed a skill acquisition research project in high-performance sport. The research project was conducted with the Australian national field hockey high-performance unit and investigated individual differences in expert goalkeepers' visual anticipation. The paper first discusses the role of a skill acquisition specialist, how they collaborate with coaches and athletes, and barriers to collaboration. The paper then outlines how psychological strategies can be used to create an autonomy-supportive environment to build a relationship and establish a research collaboration with a team. Further, the paper discusses the importance of continually involving coaches and athletes in the research process to facilitate their engagement and self-determined motivation to complete the project. By applying psychological strategies to create an autonomy-supportive environment, sports scientists may have greater success in overcoming the many barriers to conduct research in an elite sport setting, with the outcomes highly valuable for athlete development. ABSTRACT FROM AUTHOR

67	A cluster analysis of high-performance female team players' perceived motivational climate: Implications on perceived motor competence and autonomous behaviour.	Abraldes, J Arturo ; Conte Marín, Luis ; Manzano-Sánchez, David ; Gómez-López, Manuel ; Sánchez-Alcaraz, Bernardino J	PloS one	2022	10.1371/journal.pone.0278572	S1	SPORTDiscus+Scopus	High performance sport for females is an area which is gaining more and more relevance today, but which hasn't received the same research interest as sport for males. The aim of the present study was to analyse the motivational climate perceived by high performance female athletes and the implications on perceived motor competence and autonomous behaviour and check the differences according category, sport experience and training hours in performance and master climate. The participants were 615 female athletes who practice top level team sports, with ages comprised of 16 to 38 (M = 22,10; SD = 4,91). The sample consisted of two different categories: junior (n = 242) and senior (n = 373). These players participated in different team sports, specifically football, handball, basketball and volleyball, training between 6 and 24 hours a week (M = 8,34; DT = 4,33). The variables measured were: perceived motivational climate in sport, autonomous behaviour and perceived motor competence. A cluster analysis was carried out using, as a variable, the perceived motivational climate, and showing the existence of two profiles, one related to ego and the other to task. The multivariate analysis showed that the profile orientated towards the task had significant differences compared to the autonomous behaviour (M = 4.66 vs M = 3.41). At the same time an analysis was carried out looking at different social demographic variables, revealing how there were differences in the sports experience (those participants with more than ten years' experience were more orientated towards ego, compared to those with less than ten years' experience) and the category (junior athletes were more orientated towards the task, compared to senior athletes). It was concluded that a greater orientation towards the task can lead to an improvement in the perception of motor competence, with the youngest and least experienced athletes being the most prominent in this category.
68	Dark traits as a potential feature of leadership in the high-performance sports coach	Serpell, BG; Harrison, D; Lyons, M; Cook, CJ	INTERNATIONAL JOURNAL OF SPORTS SCIENCE & COACHING	2021	10.1177/1747954120964059	S1	WoS	High-performance sport coaches are leaders in their field; therefore, they sometimes operate under 'regular' workplace leadership role requirements. However, they are also subject to highly uncertain and pressured environments. Thus, most coaching/leadership positions in high-performance sport may be both 'normal' and 'unique' in leadership role requirement. Consequently, understanding what high-performance sport leaders 'look like', behaviorally, would be valuable. This study aimed to 1) describe personality traits of coaches in high-performance sport, and 2) describe differences between high-performance sport coaches and other leaders. Hogan's personality profile data was collected from fulltime coaches working in high-performance sport. Experienced and developing high-performance sport coaches were compared, and high-performance sport coaches were compared to other sector leaders. No differences exist between coaching groups for any personality traits. However, while high-performance sport coaches shared similar 'light', or 'socially desirable', traits to leaders in 'regular' workplaces they differed for some 'dark'/maladaptive traits. That is, as is the case for leaders in sectors including business, finance and healthcare high-performance coaches may be experienced as being balanced, stable, calm under pressure, approachable, friendly, accessible, planful, responsible, and mindful of details. However, unlike leaders in 'regular' workplace environments, high-performance coaches may also be experienced as moody, hard to please, creative but unusual, risk-taking, and limit-testing; which is more similar to scientists and artists. In conclusion, coach personality traits were both similar and unique to leaders from 'regular' workplaces. Thus, leadership is context specific both within sectors and between sectors.
69	The podium illusion: a phenomenological study of the influence of social support on well-being and performance in elite para swimmers	Aitchison, B; Rushton, AB; Martin, P; Soundy, A; Heneghan, NR	BMC SPORTS SCIENCE MEDICINE AND REHABILITATION	2021	10.1186/s13102-021-00269-1	S1	WoS	Background The value of social support in enhancing performance is well established in non-impaired and sub-international level athletes with impairments. Despite this, no research to date has explored the experiences of social support in elite para-athletes. The aim of this study was to explore the experiences of social support in elite British para-swimmers and the influence on their wellbeing and performance. Methods A hermeneutic phenomenological study involving semi-structured interviews was undertaken with 8 elite British para-swimmers (3 male, 5 female, mean age 24.9 years). Participants represented 5 para-swimming classes and all 10 of the International Paralympic Committee impairment categories. Data were analysed following a modified version of the Framework Method. Research quality and trustworthiness were ensured through employing techniques including data triangulation, member checking and reflexivity. Results Five themes and 11 sub-themes were generated. The five themes were: 'the coach-athlete relationship', 'team bond', 'tangible aid', 'The Podium Illusion' and 'British para-swimming'. The overall findings and the magnitude of support mentioned in the fourth theme led to the development of a new model called 'The Podium Illusion' which reflects the magnitude of support that is available to elite para-swimmers to help maximise their performance and wellbeing. Conclusion Social support is essential for athlete wellbeing and performance. Findings underpin a new model, 'The Podium Illusion'.

70	Psychosocial functioning of Olympic coaches and its perceived effect on athlete performance: a systematic review	Cook, GM; Fletcher, D; Carroll, C	INTERNATIONAL REVIEW OF SPORT AND EXERCISE PSYCHOLOGY	2021	10.1080/1750984X.2020.1802769	S1	WoS	Effective coaching facilitates athletes' success in reaching their potential in sport. Coaches possess a range of knowledge, skills and attributes that influence athletes' performance, including various intrapersonal and interpersonal qualities. The purpose of this study is to systematically review the research investigating the psychosocial functioning of Olympic coaches and its perceived effect on athlete performance. The review was conducted and reported in accordance with the PRISMA guidelines. The following databases were searched: SPORTDiscus, PsycINFO, PsycARTICLES, ScienceDirect, Scopus, Web of Knowledge, and Medline. The literature search identified 2873 studies which were screened and assessed for eligibility, with the resultant 25 eligible studies being assessed for quality of evidence using the Mixed Methods Appraisal Tool. Convergent meta-integration with thematic analysis was performed by converting quantitative and qualitative data from 207 Olympic coaches and 925 Olympic athletes into relevant themes and patterns. Three core themes of traits, states, and behaviors were identified. Within these themes, 18 traits, 28 states, and 38 behaviors were identified that were perceived to have either a facilitative, debilitative, or non-categorized effect on athlete performance. Future research will help national governing bodies and practitioners develop coach education to enhance Olympic coaching effectiveness.
71	A repeated-measures examination of organizational stressors, perceived psychological and physical health, and perceived performance in semi-elite athletes	Simms, M; Arnold, R; Turner, JE; Hays, K	JOURNAL OF SPORTS SCIENCES	2021	10.1080/02640414.2020.1804801	S3	WoS	Organizational stressors can undermine the psychological well-being and performance of athletes. Less is known, however, about how these relationships unfold over time and whether organizational stressors can impact upon perceived physical health. The current study, therefore, used a repeated-measures design to examine relationships between organizational stressors with components of perceived psychological (anxiety and depression) and physical (illness symptoms and missed training days via illness) ill-health, and perceived performance at the within-person level. Twenty-three semi-elite female rowers completed monthly measures of study variables for six-months. Multilevel models indicated that selection-related stressors positively predicted symptoms of perceived psychological and physical ill-health, and negatively predicted perceived performance. Conversely, coaching stressors negatively predicted symptoms of perceived psychological ill-health. Logistics and operations stressors positively predicted perceived performance, whereas goals and development stressors negatively predicted perceived performance. These findings demonstrate for the first time that, with a repeated-measures design, organizational stressors can predict components of perceived physical and psychological ill-health, and perceived performance at the within-person level in athletes. From a practical perspective, practitioners should incorporate these findings when diagnosing the need for, developing, and optimally implementing primary and secondary stress management interventions.
72	A Field-Based Approach to Determine Soft Tissue Injury Risk in Elite Futsal Using Novel Machine Learning Techniques	Ruiz-Pérez, I; López-Valenciano, A; Hernández-Sánchez, S; Puerta-Callejón, JM; Croix, MD; de Baranda, PS; Ayala, F	FRONTIERS IN PSYCHOLOGY	2021	10.3389/fpsyg.2021.610210	S3	WoS	Lower extremity non-contact soft tissue (LE-ST) injuries are prevalent in elite futsal. The purpose of this study was to develop robust screening models based on pre-season measures obtained from questionnaires and field-based tests to prospectively predict LE-ST injuries after having applied a range of supervised Machine Learning techniques. One hundred and thirty-nine elite futsal players underwent a pre-season screening evaluation that included individual characteristics; measures related to sleep quality, athlete burnout, psychological characteristics related to sport performance and self-reported perception of chronic ankle instability. A number of neuromuscular performance measures obtained through three field-based tests [isometric hip strength, dynamic postural control (Y-Balance) and lower extremity joints range of motion (ROM-Sport battery)] were also recorded. Injury incidence was monitored over one competitive season. There were 25 LE-ST injuries. Only those groups of measures from two of the field-based tests (ROM-Sport battery and Y-Balance), as independent data sets, were able to build robust models [area under the receiver operating characteristic curve (AUC) score >= 0.7] to identify elite futsal players at risk of sustaining a LE-ST injury. Unlike the measures obtained from the five questionnaires selected, the neuromuscular performance measures did build robust prediction models (AUC score >= 0.7). The inclusion in the same data set of the measures recorded from all the questionnaires and field-based tests did not result in models with significantly higher performance scores. The model generated by the UnderBagging technique with a cost-sensitive SMO as the base classifier and using only four ROM measures reported the best prediction performance scores (AUC = 0.767, true positive rate = 65.9% and true negative rate = 62%). The models developed might help coaches, physical trainers and medical practitioners in the decision-making process for injury prevention in futsal.

73	Longitudinal Physiological and Fitness Evaluations in Elite Ice Hockey: A Systematic Review	Chiarlitti, NA; Crozier, M; Insogna, JA; Reid, RER; Delisle-Houde, P	JOURNAL OF STRENGTH AND CONDITIONING RESEARCH	2021	10.1519/JSC.0000000000004115	S3	WoS	Chiarlitti, NA, Crozier, M, Insogna, JA, Reid, RER, and Delisle-Houde, P. Longitudinal physiological and fitness evaluations in elite ice hockey: A systematic review. J Strength Cond Res 35(10): 2963-2979, 2021-Ice hockey has greatly evolved since the last review article was published more than 25 years ago. Although players still combine anaerobic and aerobic conditioning, the pace of the game has greatly increased. Players are faster, stronger, and more agile than their predecessors; however, an important emphasis is now placed on maximizing player performance for the play-offs. For the coaching staff, strength and conditioning coaches, and players, an emphasis on mitigating fitness and physiologic losses throughout the season would be beneficial, given the intimate relationship they share with on-ice performance. This systematic review of the literature outlines the current knowledge concerning longitudinal changes in relation to fitness, body composition, and physiologic parameters across an elite hockey season. The search of 4 large scientific databases (i.e., Embase, PubMed, SPORTDiscus, and Web of Science) yielded 4,049 items, which, after removing duplicates and applying inclusion and exclusion criteria, resulted in 23 published scientific articles to be included in this review. The wide span of literature (1956-2020) made inferences difficult giving the degree to which the game of ice hockey has changed; however, more recent research points to an aerobic deconditioning pattern and increased fatigue throughout the season in a specific group of elite hockey players (i.e., university athletes) while showing that ice hockey can lead to many possible histological adaptations. Ultimately, tracking, identifying, and developing methods to mitigate potential negative longitudinal changes will be imperative to influencing individual and team performance in the later parts of the season.
74	The influence of macrocultural change on national governing bodies in British olympic sports	Feddersen, NB; Morris, R; Abrahamsen, FE; Littlewood, MA; Richardson, DJ	SPORT IN SOCIETY	2021	10.1080/17430437.2020.1771306	S3	WoS	The study objective was to examine the temporal macrocultural changes in Olympic sports in the United Kingdom and what regulates these changes. We carried out this study integrating grounded theory and action research in a 16-month longitudinal design. We collected data from eighteen interviews with participants from governing sports organisations (n = 6; GSOs) and NGBs (n = 3).came from ethnography with one national governing body; ten focus-groups with athletes, coaches, parents, and NGB personnel; and eight interviews with stakeholders. We found that political will had shielded Olympic sports from societal changes. However, macrocultural changes to social standards and the power of athletes highlighted that the organisational culture was increasingly deficient and required radical changes. GSOs used their systemic power to dictate appropriate avenues for change. Athletes used their reinforced position by speaking out about aspects that challenged the welfare of athletes and others working in Olympic sports.
75	A Longitudinal Study of Power Relations in a British Olympic Sport Organization	Feddersen, NB; Morris, R; Storm, LK; Littlewood, MA; Richardson, DJ	JOURNAL OF SPORT MANAGEMENT	2021	10.1123/jsm.2020-0119	S3	WoS	The purpose was to examine the power relations during a change of culture in an Olympic sports organization in the United Kingdom. The authors conducted a 16-month longitudinal study combining action research and grounded theory. The data collection included ethnography and a focus group discussion (n = 10) with athletes, coaches, parents, and the national governing body. The authors supplemented these with 26 interviews with stakeholders, and we analyzed the data using grounded theory. The core concept found was that power relations were further divided into systemic power and informational power. Systemic power (e.g., formal authority to reward or punish) denotes how the national governing bodies sought to implement change from the top-down and impose new strategies on the organization. The informational power (e.g., tacit feeling of oneness and belonging) represented how individuals and subunits mobilized coalitions to support or obstruct the sports organization's agenda. Olympic sports organizations should consider the influence of power when undertaking a change of culture.

76	Athlete perspectives on the enablers and barriers to nutritional adherence in high-performance sport	Bentley, MRN; Patterson, LB; Mitchell, N; Backhouse, SH	PSYCHOLOGY OF SPORT AND EXERCISE	2021	10.1016/j.psychsport.2020.101831	S3	Scopus+WoS	Objectives: Poor adherence to nutritional guidance by athletes may compromise their health and performance. Enhancing adherence is therefore an important performance and welfare strategy. The aim of this study was to qualitatively explore the barriers and enablers of elite athletes' adherence to nutritional guidelines. Design: Underpinned by our constructionist epistemological position and our relativist ontology, we conducted a qualitative study using focus groups. Methods: We used the Capability, Opportunity, and Motivation Behaviour (COM-B) model and the Theoretical Domains Framework (TDF) to conduct focus group discussions with a purposive sample of 39 UK-based funded athletes (mean age = 23 +/- 3.81), participating in either Olympic and Paralympic sport (n = 30) or professional sport (n = 9), who had access to a nutritionist. Data were analysed using reflexive thematic analysis. Results: Athlete adherence to nutritional guidance was seasonal and included inadequate energy intakes and episodes of binge eating. Underpinning these behaviours, athletes' emotional barriers (motivation) are reinforced through their social interactions within the high-performance environment (opportunity) and athletes' training environment limits developmental opportunities for food planning (capability). However, a holistic developmental approach by the sports nutritionists (opportunity) supports athlete wellbeing and nutritional adherence. Conclusion: These findings advance theoretical understanding of the barriers and enablers of nutritional adherence amongst elite-level athletes in high-performance sport and present a number of significant implications for athlete support personnel seeking to enhance performance in demanding sporting contexts. Drawing on the Behaviour Change Wheel (BCW), recommendations include the need to 1) train and educate sports nutritionists in human behaviour, 2) update regulations for sports nutrition profession practice to acknowledge the skills required to support athletes' emotional wellbeing, 3), educate coaches on the sensitivity of body weight and composition and develop guidelines for monitoring athletes' body weight and composition in sport, 4) persuade influential leaders to develop culture guidelines that shift the performance-narrative of high-performance (i.e., environmental restructuring).
77	More than just another bib: group dynamics in an elite Nordic ski team	Cascagnette, JCW; Benson, AJ; Cruwys, T; Haslam, SA; Bruner, MW	JOURNAL OF SPORTS SCIENCES	2021	10.1080/02640414.2020.1840040	S3	WoS	The social environment within individual sport teams can have a significant influence on the success, development, and well-being of athletes. We explored elite individual sport athletes' group experiences through the lens of social identity theorising. Participants were six members (4 male, 2 female, M-age = 21.5 years) of a National Development Nordic Ski team. At two time points in the competitive season, we used Social Identity Mapping (Bentley et al., 2020) in combination with semi-structured interviews to explore athletes' experiences as a member of the ski team. Social Identity Mapping provided a visual representation of each participant's social identities and was used to facilitate athletes' views of their group experiences in the semi-structured interviews. Interview data were thematically analysed to explicate participants' perceptions of social identity and cohesion, and their perceived relevance to success and development in elite individual sport. Major themes included social group memberships and identities, the presence of subgroups, the ebb and flow of cohesion and conflict, and teammate and coach influence on the group. On this basis, we argue that social identity mapping can be a valuable resource for athletes and coaches seeking to create a positive and cohesive team environment within an elite individual sport team.
78	Positive youth development and gender differences in high performance sport	O'Connor, D; Gardner, L; Larkin, P; Pope, A; Williams, AM	JOURNAL OF SPORTS SCIENCES	2020	10.1080/02640414.2019.1698001	S1	Scopus+WoS	We examined positive youth development within a high performance sport environment. Youth football players (N = 455; Males = 315; Females = 140) completed a range of questionnaires including: the Youth Experiences Survey for Sport; Self-Confidence subscale of the Competitive State Anxiety Inventory-2 Revised; Sport Competence Inventory; Prosocial and Antisocial Behaviour in Sport Scale; and the modified Coach-Athlete Relationship questionnaire. The players reported a relatively high level of self-confidence, competence and positive youth experiences. They felt a strong coach-athlete relationship and displayed higher levels of prosocial than antisocial behaviour. Males scored significantly higher than females on self-confidence, perceived self-competence, antisocial behaviour to teammates and opponents, relationship with their coach, and cognitive skills. Findings suggest a relationship between high performance sport environments and positive youth development.
79	Olympic and Paralympic coach perspectives on effective skill acquisition support and coach development	Dehghansai, N; Headrick, J; Renshaw, ; Pinder, RA; Barris, S	SPORT EDUCATION AND SOCIETY	2020	10.1080/13573322.2019.1631784	S1+S3	Scopus+WoS	The role of skill acquisition specialists within sport systems has become more prominent and imbedded in daily training environments with coaches; however, literature pertaining to their role and contributions to effective coach development is very scant. The objective was to extend our understanding of the coaches' perception of the role of, and relationship with, a skill acquisition specialist to identify key factors of effective support that shape coach behavior and ultimately enhance athlete performance. Semi-structured interviews with two National coaches with experience and podium success in multiple Olympic/Paralympic Games, Commonwealth Games, and World Championships was conducted. Three distinct narratives were identified: representing various experiences of the coaches in their sport ('the unplanned journey'), their relationship with the skill acquisition specialist ('more than just a skill expert'), and how this impacted athletes' development ('keys to success'). As part of the relationship development process, aspects of coaches' philosophy were challenged. In addition, the skill acquisition specialists had to display a wide range of skills in the pursuit of shaping coaching behaviors that could further enhance athletes' performance. Required skills included, but were not limited to, bridging the gap between scientific literature and practical application, ensuring knowledge was logical and aligned with the specific needs of the coach and cultural context, demonstrating trust and accountability, displaying personal and social skills and an ability to engage athletes and obtain their approval. Crucially, while overlapping themes occurred, the skill specialists needed to be adaptable to each unique working relationship and this emerged over time.

80	Enhancement of gratitude in the context of elite athletes: outcomes and challenges	Howells, K; Fitzallen, N	QUALITATIVE RESEARCH IN SPORT EXERCISE AND HEALTH	2020	10.1080/2159676X.2019.1679868	S1	WoS	Gratitude has been heralded as important to emotional, social and societal wellbeing. A burgeoning body of recent research on the positive impact of gratitude in sport calls for an investigation into its relevance to the field of elite sport, particularly for building resilience and enhancing relationships. An interpretivist/constructivist research approach was adopted to explore the practices, outcomes and challenges of gratitude of 10 Australian elite athletes - Olympians and Para-Olympians - who participated in a workshop on gratitude. Sports represented in this cohort included cycling, sailing, track and field, beach volleyball and diving. Semi-structured interviews were used to generate data, which were explored using content analysis. Enhancement of gratitude with the elite athletes resulted in the adoption of more meaningful and authentic gratitude towards others. Positive impact of gratitude expressed in this way was reported in the areas of improved attitude; greater awareness; better capacity to deal with stress; increased confidence to express gratitude; and enhanced performance. The paper also discusses the perceived challenges of expressing gratitude within this context.
81	The 5C's Model of Successful Partnerships in Elite Beach Volleyball Dyads	Poczwardowski, A; Lamphere, B; Allen, K; Marican, R; Haberl, P	JOURNAL OF APPLIED SPORT PSYCHOLOGY	2020	10.1080/10413200.2019.1573205	S1	Scopus+WoS	An athlete-athlete partnership is a form of athletic dyad in which both members equally share power and responsibility. Although research on the coach-athlete relationship is prevalent, research on the unique interpersonal dynamics of athlete-athlete partnerships in elite sport is sparse, particularly in beach volleyball. The purpose of the present study was to explore the interpersonal components of successful, elite athlete-athlete beach volleyball partnerships through the lens of interdependence theory and Jowett and colleagues' 3 + 1C's theory of coach-athlete relationships. Interpretative Phenomenological Analysis was applied to semistructured interviews with four Olympic-level beach volleyball players (3 male, 1 female). Results revealed 5 higher order themes: (a) compatibility, (b) commitment, (c) complementarity, (d) coorientation, and (e) closeness, which became the key constructs in the proposed 5C's model of the successful athlete-athlete partnership. Interpersonal awareness, interpersonal maturation, and context were identified as 3 overarching meta-themes, whereas interdependence connected all interpersonal components. For example, in consultation, increasing athlete individual and interpersonal awareness (e.g., in the areas of personal and dyadic philosophy, personal and shared values, and individual and dyadic coping) requires careful contextualization and thoughtful implementation. Future studies need to examine diverse samples of athlete-athlete dyads to advance interpersonal theory in sport and add to emerging theories of performance behavior and expertise in sport. Lay Summary: Successful beach volleyball partnerships share a philosophy and commitment to their sport. Desired partners are supportive and adaptive, are compatible, depend on one another, and continually appraise and reflect on their relationship to grow as individuals and as teammates.
82	Interplay of motivation and self-regulation throughout the development of elite athletes	Jordalen, G; Lemyre, PN; Durand-Bush, N	QUALITATIVE RESEARCH IN SPORT EXERCISE AND HEALTH	2020	10.1080/2159676X.2019.1585388	S3	WoS	Recent development in the understanding of human motivation has highlighted the crucial and reciprocal role of motivation on cognitive processes. In elite sport settings, athletes are subject to external forces that do not necessarily correspond with their inherent drives. However, they seem to develop cognitive competencies to cope with external forces, when planning, monitoring, and reflecting on their high-level achievements. The current study aims to explore likely interactions between motivation and cognitive processes as athletes develop from novice to elite levels. Five female Olympic and World Championship medallists were interviewed. A thematic analysis revealed how motivation and self-regulation competencies interchangeably influenced athletes' career trajectories asynchronously. Chronologically, four themes emerged: 1) Motivational shifts evoked planning and self-control competencies, 2) The external control constrained athletes' self-regulation, 3) Self-control and reflection in extrinsically driven athletes, and 4) Elite athletes' multidimensional motivation and self-regulation profile. Initially, intrinsic motivation prompted athletes' participation, but the competitive nature of sport activities led to a shift toward more external forms of motivation. This motivational shift was accompanied by changes in self-regulation competencies, particularly planning and self-control, rather than self-reflection. Over time, athletes' increased sport-specific self-confidence contributed to further refinement of self-regulation competencies and integrated motivational regulations. Rather than exploring motivation and cognitive competencies individually, current study findings highlight dynamic interactions between these concepts that influences athletes' ongoing development to elite level performances.

83	Is the Acute: Chronic Workload Ratio (ACWR) Associated with Risk of Time-Loss Injury in Professional Team Sports? A Systematic Review of Methodology, Variables and Injury Risk in Practical Situations	Andrade, R; Wik, EH; Rebelo-Marques, A; Blanch, P; Whiteley, R; Espregueira-Mendes, J; Gabbett, TJ	SPORTS MEDICINE	2020	10.1007/s40279-020-01308-6	S3	WoS	Background The acute: chronic workload ratio (ACWR) is an index of the acute workload relative to the cumulative chronic workloads. The monitoring of physical workloads using the ACWR has emerged and been hypothesized as a useful tool for coaches and athletes to optimize performance while aiming to reduce the risk of potentially preventable load-driven injuries. Objectives Our goal was to describe characteristics of the ACWR and investigate the association of the ACWR with the risk of time-loss injuries in adult elite team sport athletes. Data sources PubMed, EMBASE and grey literature databases; inception to May 2019. Eligibility criteria Longitudinal studies that assess the relationship of the ACWR and time-loss injury risk in adult professional or elite team sports. Methods We summarized the population characteristics, workload metrics and ACWR calculation methods. For each workload metric, we plotted the risk estimates for the ACWR in isolation, or when combined with chronic workloads. Methodological quality was assessed using a modified version of the Downs and Black scale. Results Twenty studies comprising 2375 injuries from 1234 athletes (all males and mean age of 24 years) from different sports were included. Internal (65%) and external loads (70%) were collected in more than half of the studies and the session-rating of perceived exertion and total distance were the most commonly collected metrics. The ACWR was commonly calculated using the coupled method (95%), 1:4 weekly blocks (95%) and subsequent week injury lag (80%). There were 14 different binning methods with almost none of the studies using the same binning categories. Conclusion The majority of studies suggest that athletes are at greater risk of sustaining a time-loss injury when the ACWR is higher relative to a lower or moderate ACWR. The heterogenous methodological approaches not only reflect the wide range of sports studied and the differing demands of these activities, but also limit the strength of recommendations. PROSPERO registration number CRD42017067585.
84	Does the longitudinal development of physical and anthropometric characteristics associate with professional career attainment in adolescent Australian footballers?	Cripps, AJ; Banyard, HG; Woods, CT; Joyce, C; Hopper, LS	INTERNATIONAL JOURNAL OF SPORTS SCIENCE & COACHING	2020	10.1177/1747954120927117	S3	WoS	This study sought to longitudinally and retrospectively determine the relationship between professional career attainment and the development of anthropometric and physical qualities in junior Australian footballers. Eighty adolescent male Australian footballers from a single state academy previously selected onto an under 16 s talent development squad were classified by career attainment (professional team selection; n = 17 and non-selected; n = 63). Physical and anthropometric tests were conducted at the end of preseason during participation in under 16 and under 18 competitions. Tests included standing height, mass, stationary countermovement jumps, dynamic vertical jumps, 20-m sprints, agility and 20 m multistage fitness test. Both groups significantly improved all performance measures between the under 16 and under 18 levels. Athletes selected onto a professional team possessed significantly quicker 20-m sprint outcomes than non-selected athletes at both under 16 and under 18 levels, highlighting the importance of this physical capacity within talent development programmes. Binary logistic regression was unable to predict an effect of any measures on career attainment. An inability of the binary logistic regression to identify qualities predictive of long-term career success likely highlights limitations associated with utilising unidimensional models of assessment in talent identification practices. As such development coaches and sport scientists should be aware that while physical capacities play a role in career attainment outcomes, other factors, such as tactical understanding and technical skill are also likely to be impactful.
85	Development, Implementation, and Evaluation of anAthlete-Informed Mental Skills Training Program for EliteYouth Tennis Players	Dohme, LC; Bloom, GA; Piggott, D; Backhouse, S	JOURNAL OF APPLIED SPORT PSYCHOLOGY	2020	10.1080/10413200.2019.1573204	S3	Scopus+WoS	Informed by athletes' psychological needs, the current qualitative study developed, implemented, and evaluated a 15-month mental skills training (MST) program for elite youth athletes. The MST was divided into 3 phases that included a 9-month preintervention, a 2-month intervention, and a 4-month evaluation phase. The intervention consisted of 3 interactive workshops that were delivered to 11 competitive British youth tennis players (8-15 years of age) and their coach (age 34). The intervention was informed by data that were collected throughout a 9-month preintervention phase including longitudinal observations, field notes, and semistructured interviews. The intervention was evaluated over 4 months through observations, field notes, athlete-workshop data, and a semistructured interview with the coach. Results reinforced the value of the longitudinal preintervention phase by highlighting that the establishment of rapport between the researcher and athletes enhanced the meaningfulness and content of the MST program. In addition, an increase in athletes' use and regulation of psychological skills and characteristics (PSCs) was identified as a result of athletes' improved understanding of psychological skills (i.e., self-talk, imagery, performance routines) and characteristics (i.e., focus, emotional control, PSCs). Finally, the MST program fostered a shared subject-specific language between athletes and their coach, enhancing the openness and frequency with which PSCs were talked about. Practical guidelines for future sport psychology interventions with youth athletes and their coaches are provided. Lay Summary:A 15-month mental skills training program was conducted with youth tennis players to enhance their ability to regulate their focus and emotional control. Practical guidelines for future interventions with youth athletes and their coaches are provided.

86	Salivary IgA as a Predictor of Upper Respiratory Tract Infections and Relationship to Training Load in Elite Rugby Union Players	Tiernan, C; Lyons, M; Comyns, T; Nevill, AM; Warrington, G	JOURNAL OF STRENGTH AND CONDITIONING RESEARCH	2020	10.1519/JSC.0000000000003019	S3	WoS	Tiernan, C, Lyons, M, Comyns, T, Nevill, AM, and Warrington, G. Salivary IgA as a predictor of upper respiratory tract infections and relationship to training load in elite rugby union players. J Strength Cond Res 34(3): 782-790, 2020-Upper Respiratory Tract Infections (URTI) are among the most common illnesses reported in athletes. An URTI can result in missed training days, which in turn may lead to performance decrements. The purpose of this study was to investigate the use of salivary immunoglobulin A (sIgA) as a predictor of URTI, while also exploring the relationship to weekly training load in elite rugby union players. Nineteen male elite rugby union players provided morning saliva swabs, biweekly (Monday and Friday), over a 10-week training period. Participants completed an illness log documenting symptoms of URTI. Session Rate of Perceived Exertion (sRPE) was collected to determine training load (sRPE x session duration). Weekly training load was also calculated. Logistic regression was used to analyze the relationship between incidences of URTI with sIgA and training load. Multilevel regression was conducted to compare associations between sIgA and training load. The results found that the likelihood of suffering from an URTI increased when sIgA significantly decreased ($p = 0.046$). Where sIgA decreased by 65% or more, a player was at a greater risk of contracting an URTI within the following 2 weeks. No association was found between sIgA and training load. In conclusion, sIgA may be a useful predictor for determining the likelihood of players contracting an URTI. This will allow the coach to make informed decisions on training status, helping reduce the risk of players missing training, which may have performance decrements. Coaches will benefit from the fast, easy, and instant results available, to analyze a player's immune function.
87	The emergence and perpetuation of a destructive culture in an elite sport in the United Kingdom	Feddersen, NB; Morris, R; Littlewood, MA; Richardson, DJ	SPORT IN SOCIETY	2020	10.1080/17430437.2019.1680639	S3	Scopus+WoS	Recent inquiries into elite sports in the United Kingdom have unearthed examples of destructive cultures. Yet, earlier research left destructive cultures overlooked. The purpose of this article is to: (1) outline the process of how a destructive organizational culture emerges and perpetuates in one Olympic sport in the United Kingdom, and (2) the features that regulate the process. We combined Action Research and Grounded Theory in a 16-month longitudinal study. The primary data collection strategies were ethnography and 10 focus groups, with athletes, coaches, parents and the national governing body (NGB). Twenty-six individual interviews with stakeholders supplemented these. A destructive culture emerged during radical changes, and antagonism in the power relations between the NGB and stakeholders characterised this process. Denial of responsibility and social weighting neutralised the stigma of perpetuating antagonism. In conclusion, sports organizations should be vigilant of how ignoring and denying antagonism could lead to a destructive culture.
88	Measurement Characteristics of Athlete Monitoring Tools in Professional Australian Football	Ryan, S; Pacecca, E; Tebble, J; Hocking, J; Kempton, T; Coutts, AJ	INTERNATIONAL JOURNAL OF SPORTS PHYSIOLOGY AND PERFORMANCE	2020	10.1123/ijspp.2019-0060	S3	WoS	Purpose: To examine the measurement reliability and sensitivity of common athlete monitoring tools in professional Australian Football players. Methods: Test-retest reliability (noise) and weekly variation (signal) data were collected from 42 professional Australian footballers from 1 club during a competition season. Perceptual wellness was measured via questionnaires completed before main training sessions (48, 72. and 96 h postmatch), with players providing a rating (1-5 Likert scale) regarding their muscle soreness, sleep quality, fatigue level, stress, and motivation. Eccentric hamstring force and countermovement jumps were assessed via proprietary systems once per week. Heart rate recovery was assessed via a standard submaximal run test on a grass-covered field with players wearing a heart rate monitor. The heart rate recovery was calculated by subtracting average heart rate during final 10 seconds of rest from average heart rate during final 30 seconds of exercise. Typical test error was reported as coefficient of variation percentage (CV%) and intraclass coefficients. Sensitivity was calculated by dividing weekly CV% by test CV% to produce a signal to noise ratio. Results: All measures displayed acceptable sensitivity. Signal to noise ratio ranged from 1.3 to 11.1. Intraclass coefficients ranged from .30 to .97 for all measures. Conclusions: The heart rate recovery test, countermovement jump test, eccentric hamstring force test, and perceptual wellness all possess acceptable measurement sensitivity. Signal to noise ratio analysis is a novel method of assessing measurement characteristics of monitoring tools. These data can be used by coaches and scientists to identify meaningful changes in common measures of fitness and fatigue in professional Australian football.
89	A Pre-Match Video Self-Modeling Intervention in Elite Youth Football	Middlemas, S; Harwood, C	JOURNAL OF APPLIED SPORT PSYCHOLOGY	2020	10.1080/10413200.2019.1590481	S3	WoS	In this study, a self-modelling video intervention was delivered to four elite youth soccer players over 13 weeks of the competitive soccer season. A multiple-baseline, repeated measures single-subject design was used to explore the impact of the intervention on subcomponents of soccer performance and psychological variables, including self-efficacy and positive/negative affect. Statistical analysis indicated that there were significant changes between pre- and post-intervention scores for two players (1 & 3) on three subcomponents of performance (turns, headers and tackles). The findings of this study highlight the potential benefits of designing individualised pre-match video interventions with elite youth athletes.Lay Summary: Video self-modelling (VSM) is a behavioural intervention in which an athlete views a video of themselves engaged in adaptive behaviour, in order to learn to reproduce it effectively under pressure. In this study, the impact of a self-modelling video intervention is examined within a youth football soccer environment.

90	Tapering practices of elite CrossFit athletes	Pritchard, HJ; Keogh, JW; Winwood, PW	INTERNATIONAL JOURNAL OF SPORTS SCIENCE & COACHING	2020	10.1177/1747954120934924	S3	WoS	This is the first study to document the training and tapering practices of elite CrossFit athletes. Seventy-two CrossFit athletes (39 females, 33 males) (mean +/- SD; 26.5 +/- 3.6 y, 167.1 +/- 9.5 cm, 74.5 +/- 12.7 kg, 12.8 +/- 6.5 y general training, 5.4 +/- 1.7 y CrossFit training) who competed at the Regionals level or higher in the 2018 CrossFit Games season completed a self-reported 5-page online survey. Almost all athletes (98.6%) tapered before important competitions. Taper length was 5.4 +/- 2.7 days, with the step and linear tapering styles being most commonly utilised. Strength training volume peaked 5.1 +/- 4.6 weeks before competition, whereas conditioning training volume peaked 4.0 +/- 4.4 weeks before competition. Strength training intensity peaked 3.1 +/- 2.4 weeks before competition, whereas conditioning training intensity peaked 2.8 +/- 2.2 weeks before competition. Almost all athletes (90.0%) reduced training duration during tapering, but changes to frequency and intensity were mixed. Training volume decreased by 41.2 +/- 15.5% during the taper, all training ceased 2.0 +/- 1.1 days before competition. Tapering was performed to achieve peak performance, recover (physically and psychologically), and reduce feelings of fatigue. Poor results from tapering were experienced when athletes tapered for too long or insufficiently. This observational data may be valuable for coaches and athletes engaged in CrossFit as well as other sports with concurrent training and competition demands.
91	The Cognitive Component of Elite High Jumpers' Preperformance Routines	Gretton, T; Blom, L; Hankemeier, D; Judge, L	SPORT PSYCHOLOGIST	2020	10.1123/tsp.2019-0093	S3	WoS	Preperformance routines are microlevel performance processes utilized by athletes to facilitate the attainment of an optimal state and enhance the chance for successful performance. Despite continued examination of these routines, only a small proportion of research has been directed toward the cognitive component of these routines. This study explored the cognitive component of elite high jumpers' preperformance routines, and specifically the consistency of the cognitive content (i.e., psychological skills and strategies). Data were acquired over an 8-week high-jump season and subjected to inductive thematic analysis. Results revealed the consistent implementation of the cognitive content (e.g., visualization) but an inconsistent design of this content (i.e., the content of the visualization). Furthermore, results underline the critical role of high-jump coaches and an athlete's need to be adaptable and competent in utilizing various types of preperformance routine. This study offers valuable insight into the complexities and inconsistencies of the cognitive component of high jumpers' preperformance routines.
92	Sports performers' perspectives on facilitating thriving in professional rugby contexts	Brown, DJ; Arnold, R	PSYCHOLOGY OF SPORT AND EXERCISE	2019	10.1016/j.psychsport.2018.09.008	S1	WoS	Objectives: The desire and apparent 'need' to succeed can drive a win-at-all-costs mentality in individuals who operate in sport. This approach has given rise to environments where the pursuit of high-level performance has been at the expense of athlete welfare. To redress this balance and to inform the creation and maintenance of sporting environments that promote both high performance and athlete well-being, we sought to (i) provide an exploration of the environmental factors that may promote player thriving in professional sport, and (ii) offer suggestions for how key stakeholders can support these mechanisms. Design: A qualitative research methodology underpinned by ontological relativism and epistemological constructionism. Method: Semi-structured interviews were conducted with 18 players from an English professional rugby union club. Results: Thematic analysis generated thirteen factors that players perceived to promote thriving. These factors were underpinned by two general ideas: (i) Establishing Bonds between Teammates and (ii) Establishing a Connection to the Coaching Staff and the Club. Conclusions: The findings provide the first insight into the type of environment professional rugby players believe can facilitate their development and sporting success. The factors generated in the analysis highlight the players' desire to operate within an integrated, inclusive, and trusting environment, and a variety of suggestions are forwarded for how this can be achieved. To promote thriving in a professional sport that requires emotion and passion to succeed on the pitch, it appears that rugby union organizations must appeal to the sensitivities and welfare of players off it.
93	A heuristic for the relationship between caring coaching and elite athlete performance	Fisher, LA; Larsen, LK; Bejar, MP; Shigeno, TC	INTERNATIONAL JOURNAL OF SPORTS SCIENCE & COACHING	2019	10.1177/1747954119827192	S1	WoS	While the topic of caring coaching and its relationship to performance has been explored by researchers mainly in the UK, it has been neglected in the US with the exception of three studies by Fisher and colleagues. The core problem is a lack of understanding regarding the construct of care and whether coaches can care about athletes in pressure-filled, high-stakes, win-at-all-cost sport contexts. Thus, in this paper, we draw upon the ethic of care first proposed by Gilligan and later developed by Noddings and on results from the aforementioned studies by Fisher and colleagues as well as insights from scholarship in the UK to propose a heuristic of the potential relationship between caring coaching and elite athlete performance. Such a heuristic could be used in future coach development programs.
94	Exploring the Coach's Role in Fostering Resilience in Elite Athletes	Kegelaers, J; Wylleman, P	SPORT EXERCISE AND PERFORMANCE PSYCHOLOGY	2019	10.1037/spy0000151	S1	WoS	Although resilience in sports has become a growing topic of research, little is known about how this capacity should he fostered or developed. Furthermore, as strategies to develop resilience are generally formulated in function of sport psychologists, less attention has been paid to coaches, who might play an important role in developing and sustaining athletes' resilience through their day-to-day interactions. The purpose of the present study was. therefore, to explore how coaches can foster resilience. Semistructured interviews were conducted with 4 elite athletes, 4 elite-level coaches, and 2 sport psychologists. Thematic analysis revealed 6 major themes reflecting resilience training strategies. Three themes reflected proactive (i.e., before the occurrence of a stressor) strategies (i.e., fostering motivation. mental preparation. and promoting life balance) and 3 themes reflected reactive (i.e., after the occurrence of a stressor) strategies (i.e., evaluating setbacks, promoting a positive mindset, and implementing lessons). Furthermore, the use and effectiveness of these strategies were believed to be influenced by coaches' interpersonal skills (i.e., quality of coach-athlete relationship and an individualized approach).

95	Antecedents and consequences of perceived autonomy support in elite sport: A diary study linking coaches' off-job recovery and athletes' performance satisfaction	Balk, YA; de Jonge, J; Geurts, SAE; Oerlemans, WGM	PSYCHOLOGY OF SPORT AND EXERCISE	2019	10.1016/j.psychsport.2019.04.020	S1	SPORTDiscus+WoS	Objectives: To prevent poor health and well-being resulting from the high demands of coaching in elite sport, coaches need to recover during time away from work. This can benefit coaches' own work experiences as well as their interpersonal behaviour towards athletes. Therefore, the first aim of this study was to investigate within-person associations between elite coaches' off-job recovery (i.e., physical, cognitive, and emotional detachment from work), physical fatigue, positive affect, work engagement, and perceived autonomy support. The second aim was to investigate within-person associations between elite athletes' daily perceptions of autonomy support, athlete engagement, and performance satisfaction. Design: A one-week daily diary study was conducted. Method: Thirty-one elite coaches (30 males, 1 female) and 96 elite athletes (67 males, 29 females) completed online daily surveys across eight consecutive days. Results: Multilevel structural equation modelling showed that coaches' daily off-job physical detachment was negatively related to physical fatigue the next morning, whereas daily off-job emotional detachment was positively related to positive affect the next morning. Physical fatigue and positive affect were positively and negatively related to daily work engagement, respectively, which in turn was positively related to athletes' perceptions of autonomy support. For athletes, daily athlete engagement fully mediated the relation between daily perceived autonomy support and daily performance satisfaction. Conclusions: This study shows that off-job recovery, in terms of physical and emotional detachment from work, is not only important for elite coaches' health, well-being, and work engagement but also benefits elite athletes' daily sport experiences.
96	MOTIVATION IN THE PRACTICE OF JUDO IN NON-PROFESSIONAL ATHLETES	Castro-Sánchez, M; Zurita-Ortega, F; Zafra-Santos, E; Rodríguez-Fernández, S; Chacón-Cuberos, R; Valdivia-Moral, P	REVISTA INTERNACIONAL DE MEDICINA Y CIENCIAS DE LA ACTIVIDAD FISICA Y DEL DEPORTE	2019	10.15366/rimcafd2019.74.005	S1	WoS	The aim of this study is to determine and analyse the relationships and differences in the motivational climate, based on the competitive level in non-professional judokas. A sample of 121 non-professional Chilean judokas (70 men and 51 women) aged between 18 and 40, coming from seven different judo clubs took part. They completed a self-registration sheet of sociodemographic variables and a motivational climate questionnaire (PMCSQ-2). The results indicate that the task goal orientation prevails in non-professional judokas and as the level rises, in amateur athletes the ego goal orientation takes on greater importance. The main deduction is that amateur judokas distinguish in a greater proportion between personal improvement and effort (task goal orientation) and show a greater physical ability than others.This is opposed to what happens with non-professionals, where the correlation between both is lower, although still negative.
97	Burnout symptoms and recovery processes in eight elite soccer coaches over 10 years	Hassmén, P; Kenttä, G; Hjälm, S; Lundkvist, E; Gustafsson, H	INTERNATIONAL JOURNAL OF SPORTS SCIENCE & COACHING	2019	10.1177/1747954119851246	S3	WoS	Elite sport can be stressful, which increases the risk for burnout symptoms to develop. Especially when not balanced with sufficient recovery. To study the burnout-recovery process, eight elite soccer coaches were followed for 10 years. All eight were active elite coaches at the inception of this study and reported elevated emotional exhaustion scores on Maslach's Burnout Inventory Educators Survey (MBI-ES). The coaches completed MBI-ES three additional times (year 3, 7, and 10), and they were also interviewed on the same occasions. At the 3-year follow-up, seven of the eight coaches reduced their exhaustion scores. The coach presenting with unchanged scores both at the 3 - and 7-year follow-up was the only one still coaching at the elite level. All coaches revealed during the interviews that they struggled to manage their work-life balance well; some worked too many hours, some experienced difficulty in managing conflicting role-demands, and some wrestled with external pressures. Their approach to recovery was, however, similar. Apart from moving away from coaching at the elite level, they unanimously mentioned that they changed their approach to coaching to make recovery possible. They achieved the latter by, for example, increasing control and delegating responsibility. According to our longitudinal results, burnout frequently regarded as an end-state can decrease over time. Provided that decisive action is taken to change situational factors and personal demands. This frequently meant withdrawing from coaching, which in turn explains why coach retention remains a serious challenge for most organizations with teams/athletes competing at the elite level.
98	It's not about sport, it's about you: An interpretative phenomenological analysis of mentoring elite athletes	Sandardos, SS; Chambers, TP	PSYCHOLOGY OF SPORT AND EXERCISE	2019	10.1016/j.psychsport.2019.02.003	S3	WoS	Objective: The need to support the well-being of elite athletes in a holistic manner is becoming increasingly evident with the rise of reported mental health issues in this context. Surprisingly, mentoring as a support mechanism for athlete well-being has yet to be explored. The purpose of this exploratory study was to develop a contextualized perspective of the experience of mentoring within sport, and investigate whether athletes perceived benefits to their well-being as a consequence of engaging in mentoring. Design and methodology: An interpretative phenomenological analysis was adopted (IPA; Smith, Flowers, & Larkin, 2009). Semi-structured interviews were conducted with eight mentors and three athlete-mentees, who had been mentored for at least 6 months. Results: Data analysis of participant narratives were conceptualized into five high-order themes: (1) role; (2) attributes; (3) experience and training; (4) relationship structure; and (5) agenda. Higher-order themes were embedded into three superordinate themes: critical elements of mentoring, effective relationships, and perceived benefits to psychological well-being. Conclusion: Identified themes paralleled the dimensions in Ryffs (1989) model of psychological well-being (PWB). Collectively, these findings advance the limited literature on the experience of athlete mentoring and highlight it as a potential mechanism for the development of holistic athlete well-being.

99	Motivational processes affecting the perception of organizational and media stressors among professional football players: A longitudinal mixed methods research study	Kristiansen, E; Ivarsson, A; Solstad, BE; Roberts, GC	PSYCHOLOGY OF SPORT AND EXERCISE	2019	10.1016/j.psychsport.2019.02.009	S3	WoS	Objectives: The purpose of this study was to examine professional football players' perceptions of organizational and media stressors over a season using Achievement Goal Theory (AGT) as the overarching motivational framework. We elaborated upon the experience of the extant motivational climate and how players perceived and coped with a number of organizational and media stressors during the season. Design: A mixed-methods approach was used in this longitudinal investigation with the quantitative testing of hypotheses (strand 1) complemented with interviews (strand II) exploring the perceptions of players of the target variables within their role in the team (captains, starters, and marginal players). Method: 27 players from one men's team in a Scandinavian Premier Division completed a series of questionnaires at ten occasions during the season. We used the Bayesian dynamic p-technique analysis to investigate the relationships between AGT constructs and stressors. At the end of the season, 11 players were interviewed. The qualitative data were coded and narratives were used when presenting the findings. Results: The quantitative results showed that a perceived mastery climate created by the coach was associated with low perceived magnitude of organizational stressors. The qualitative results revealed the main categories of organizational stressors were the selected line-up for games, injuries, and losing games. The results for media stressors revealed that task orientation was associated with low perceived magnitude of media stressors the following month, while ego orientation had the opposite relationship. The qualitative findings revealed that injured and marginal players were more sensitive to media questioning in a season where the team was acknowledged for their team performance. Conclusions: There are benefits of being task-involved through task orientation and perception of a mastery climate to combat the quantity, frequency, and/or intensity of the perception of organizational and media stressors in professional football.
100	The relationship of team and individual athlete performances on match quarter outcome in elite women's Australian Rules football	Cust, EE; Sweeting, AJ; Ball, K; Anderson, H; Robertson, S	JOURNAL OF SCIENCE AND MEDICINE IN SPORT	2019	10.1016/j.jsams.2019.05.004	S3	WoS	Objectives: To evaluate the relationships between the athlete distribution of team performance indicators and quarter outcome in elite women's Australian Rules football matches. Design: Retrospective longitudinal cohort analysis. Methods: Thirteen performance indicators were obtained from 56 matches across the 2017 and 2018 Australian Football League Women's (AFLW) seasons. Absolute and relative values of 13 performance indicators were obtained for each athlete, in each quarter of all matches. Eleven features were further extracted for each performance indicator, resulting in a total of 169 features. Generalised estimating equations (GEE) and regression decision trees were run across the different feature sets and dependent variables, resulting in 22 separate models. Results: The GEE algorithm produced slightly lower mean absolute errors across all dependent variables and feature sets comparative to the regression decision tree models. Quarter outcome was more accurately explained when considered as total points scored comparative to quarter score margin. Team differential and the 75th percentile of individual athlete Inside 50s were the strongest features included in the models. Conclusions: Modelling performance statistics by quarter outcomes provides specific practical information for in-game tactics and coaching in relation to athlete performances each quarter. Within the current elite women's Australian Rules football competition, key high performing individual athletes' skilled performances within matches contribute more to success rather than a collective team effort. (C) 2019 Sports Medicine Australia. Published by Elsevier Ltd. All rights reserved.
101	Training, match and non-rugby activities in elite male youth rugby union players in England	Hendricks, S; Till, K; Weaving, D; Powell, A; Kemp, S; Stokes, K; Jones, B	INTERNATIONAL JOURNAL OF SPORTS SCIENCE & COACHING	2019	10.1177/1747954119829289	S3	WoS	Rugby union is a late specialisation sport. As a consequence, youth players may still be engaged in other activities and sports throughout the year as they transition to rugby specialisation. Limited research exists quantifying rugby union training and matches as well as engagement in other activities and sports. Therefore, the aim of this study was to quantify and compare rugby union training, matches and other activities of elite youth U15 and U16 rugby union players at different stages of the season. Four-hundred and ninety-two youth (Under-15 and 16-year-old) rugby union players self-reported the frequency, intensity and duration of their participation in rugby union matches, rugby union training, gym, physical education and other sports during three different stages - September to December (Sept-Dec), January to April (Jan-Apr), May to August (May-Aug) - of the year. When all activities were combined, the frequency and volume of rugby matches and training was the greatest during the Sept-Dec stage. The frequency and volume of participating in other sports increased in the May-Aug stage of the season. Gym training frequency, intensity, and duration were stable across the year for both U15 and U16 players. Coaches and practitioners working with elite youth rugby union players should be aware that players are still participating in other activities outside of rugby training and competition. Coaches and practitioners should put structures in place to plan and monitor players' activities in order to optimise the positive outcomes of specialisation (e.g., expertise) while reducing the potential negative outcomes (e.g., injury, burnout).

102	Coaching Efficacy, Player Perceptions of Coaches' Leadership Styles, and Team Performance in Premier League Soccer	Keatlholetswe, L; Malete, L	RESEARCH QUARTERLY FOR EXERCISE AND SPORT	2019	10.1080/02701367.2018.1563277	S3	WoS	The coaching competency research has demonstrated the role of coaching efficacy and coaching behaviors on various athlete outcomes. However, athlete perceptions of these relationships and how they affect performance are less understood. Purpose: This study examined if coaching efficacy is predictive of player perceptions of coaches' leadership styles, team atmosphere, and team performance in a soccer season. Method: Fifteen male premier league soccer coaches (M-age=45.27, SD=6.07) and 226 players (M-age=25.66, SD=3.96) from Botswana participated in the study. All participants completed a background information questionnaire. Coaches completed the Coaching Efficacy Scale. Players rated their coaches' leadership styles using the Revised Leadership Scale for Sports as well as team atmosphere. Team performance was based on position in the league log and player ratings of the teams' performance. Results: Findings showed that coaches' self-ratings on technique efficacy predicted player perceptions of the coaches' use of all six leadership styles. Game strategy efficacy predicted higher team atmosphere and team performance. Motivation efficacy was not significantly associated with player perceptions of the coaches' use of any of the leadership styles, while character building efficacy was negatively associated with the various leadership styles. Conclusion: Findings provide support to previous research evidence linking higher coaching efficacy, leadership styles, and team outcomes. The study expands the emergent research within the coaching competency literature that examines player perceptions of coaches' behaviors and leadership styles.
103	Elite female footballers' stories of sociocultural factors, emotions, and behaviours prior to anterior cruciate ligament injury	Ivarsson, A; Johnson, U; Karlsson, J; Börjesson, M; Hägglund, M; Andersen, MB; Waldén, M	INTERNATIONAL JOURNAL OF SPORT AND EXERCISE PSYCHOLOGY	2019	10.1080/1612197X.2018.1462227	S3	WoS	The aim of the study was to examine how players' perceptions of sociocultural factors and intra- and interpersonal aspects of sporting experiences may have influenced the emotions, cognitions, and behaviours of elite female soccer players prior to the occurrence of ACL injuries. The research questions guiding the study were: (a) how did female elite soccer players perceive that their psychosocial experiences were related to their cognitive, physiological, and emotional states prior to their ACL injuries, and (b) how did the players feel their perceived states influenced their behaviours prior to injury occurrence. The participants consisted of the total population of female players (N = 18) competing in the Swedish women's elite league, who incurred a total ACL tear during the 2012 season. Using a semi-structured interview guide, all players were interviewed post-season. We represented the data using a storytelling approach of aggregated creative nonfiction. The aggregated stories showed sociocultural rules and expectations of overtraining and placing pressure on athletes to play even if they were not physically or psychologically fit. Responding to pressures with potentially risk-increasing behaviours might raise the probability of becoming injured through a number of pathways. Team managers, coaches, and members of the medical team are recommended to develop environments that stimulate the players to engage in adaptive stress-recovery and risk-decreasing behaviours.
104	Effects of Self-Talk Training on Competitive Anxiety, Self-Efficacy, Volitional Skills, and Performance: An Intervention Study with Junior Sub-Elite Athletes.	Walter, Nadja ; Nikoleizig, Lucie ; Alfermann, Dorothee	Sports (2075-4663)	2019	10.3390/sports7060148	S2	SPORTDiscus	(1) Background: Self-talk (ST) is used to influence athletes' thoughts, feelings, and behaviors. Samples of squad and competitive athletes are underrepresented, although research has proven the positive effects of ST in the context of sports. Thus, the present study focused on the impact of ST on psychological and performance outcomes of junior sub-elite athletes. (2) Methods: N = 117 athletes (55 females, 62 males; M = 16.0 years) were randomly assigned to either one of two experimental groups or to a control group (n = 30). The experimental groups received an ST intervention for either one week (n = 36) or eight weeks (n = 38), and the control group received no ST training. The dependent variables (competitive anxiety, volitional skills, self-efficacy, and coaches' performance ratings) were assessed three times before and after the intervention. It was expected that (a) an ST intervention would reduce the competitive anxiety and increase volitional skills, self-efficacy, and performance; and, (b) long-term training would lead to higher effects than short-term training. (3) Results: As expected, ST training led to (less) somatic state anxiety and (higher) state self-confidence, self-optimization, self-efficacy, and performance. Additionally, long-term training was more effective than short-term training. (4) Conclusions: Targeted ST interventions may help to improve junior athletes' psychological states and performance. ABSTRACT FROM AUTHOR
105	Need-supportiveness and athlete well-being: Coaches' competence-support at risk in the elite sport context throughout the season.	Berntsen, Hedda ; Ivarsson, Andreas ; Kristiansen, Elsa	Current Issues in Sport Science	2019	10.15203/CISS_2019.010	S3	SPORTDiscus	The aim of this study was to explore how perceptions of coaches' behaviour affected athletes' wellbeing, by examining: (a) the potential change in perceptions of need-support from the coach (over an academic year), and (b) the within-person relationship between the three aspects of need-supportiveness and subjective vitality at the end of the winter sport season. Elite student athletes (N = 102, M age = 17.04) completed a questionnaire three times. Bayesian growth curve analyses showed that perceptions of relatedness and autonomy support were stable and high throughout the year. In contrast, perceptions of competence support decreased during the season. In addition, the results showed a credible positive within-person relationship between changes in all three facets of needsupportiveness from the coach and vitality measured at the end of the season, which supports SDT tenets. These findings reveal the importance of need-support for athletes' well-being, and show that competence support needs extra attention in the elite sport context where competence satisfaction is constantly challenged. ABSTRACT FROM AUTHOR

106	Optimal Development of Youth Athletes Toward Elite Athletic Performance: How to Coach Their Motivation, Plan Exercise Training, and Pace the Race.	Menting, Stein G P ; Hendry, David T ; Schiphof-Godart, Lieke ; Elferink-Gemser, Marije T ; Hettinga, Florentina J	Frontiers in sports and active living	2019	10.3389/fspor.2019.00014	S3	SPORTDiscus	Elite athletes have invested many years in training and competition to reach the elite level. One very important factor on the road to elite performance is the decision-making process regarding the regulation of effort over time, termed as pacing behavior. The regulation of effort is vital for optimal athletic performance during a single race and over a longer period of time (e.g., a competitive season) as an inadequate regulation could result in a higher risk of injuries, overtraining, and drop-out. Despite this, there is limited knowledge on how young athletes learn and develop the abilities related to pacing. Pacing behavior of athletes develops from childhood throughout adolescence and is thought to be closely connected to physical maturation, the development of pre-frontal cortical related (meta-) cognitive functions, as well as the gathering of experience with exercise tasks. The motivation of an athlete can critically influence how an athlete paces a single race, but also how they distribute their effort over a longer period of time. Coaches are advised to closely monitor the development of pacing behavior during adolescence (e.g., by gathering split times, and related physiological measurement, during training and competition), as well as the underlying factors including physical maturation (meta-) cognitive development and the motivation of young athletes. Furthermore, pacing behavior development could be aided by providing training in which the task, individual, and environment are manipulated. Hereby, presenting athletes with the opportunity to gain experience in situations which closely resemble the perceptual-motor conditions of upcoming competitions.
107	A longitudinal examination of elite youth soccer players: The role of passion and basic need satisfaction in athletes' optimal functioning	Verner-Filion, J; Vallerand, RJ	PSYCHOLOGY OF SPORT AND EXERCISE	2018	10.1016/j.psychsport.2018.07.005	S3	Scopus+WoS	Objectives: Grounded in the basic psychological needs theory (BPNT; Ryan & Deci, 2017) and dualistic model of passion (DMP; Vallerand et al., 2003), the aim of the present study was to examine within-person variations in athletes' optimal functioning (i.e., positive and negative affect, athletic satisfaction, and quality of preparation and performance) as a function of passion types and need satisfaction over the course of three competitive seasons. Method: Elite youth soccer players (n = 91) completed multi-section questionnaires on up to five occasions over the course of three competitive seasons. Results: Results of Hierarchical Linear Modeling analyses showed that between-person variations in harmonious passion (HP) were positively related to optimal functioning, whereas it was only partially the case with obsessive passion (OP). Moreover, within-person variations in the satisfaction of autonomy, relatedness, and competence were also associated with increases in athletes' psychological well-being (Le., positive and negative affect, and athletic satisfaction). Additionally, results from a multilevel indirect effects model revealed that HP and increases in competence were both positively related to increases in the quality of athletes' preparation, which in turn led to increases in performance, as rated by coaches, over the span of three competitive seasons. Conclusions: Overall, the results offer support for the effects of needs and passion on optimal functioning and are discussed in line with their implications for athletes in elite youth sports settings.
108	Champions are racers, not pacers: an analysis of qualification patterns of Olympic and IAAF World Championship middle distance runners	Hanley, B; Hettinga, FJ	JOURNAL OF SPORTS SCIENCES	2018	10.1080/02640414.2018.1472200	S3	WoS	The aim of this study was to analyse qualification patterns in middle distance running and identify whether athletes adopt theoretically optimal tactics, or whether the will to win overrides these. The performances of 295 men and 258 women finalists in the Olympic and IAAF World Championship 800m and 1500m events from 1999 to 2017 were analysed across all three rounds of competition. Finishing position, time and ranking amongst all competitors were found for each athlete. Position in the final was correlated with finishing position in the heats and semi-finals (all $P<0.001$), but not with finishing times in those rounds. Of the 57 champions, 40 won both their heat and semi-final, even though a lower automatic qualification position would have been sufficient, and only 18 achieved a season's best time in the final. The will to win amongst the eventual champions (and other medallists) suggests predominantly ego oriented behaviour that is encouraged by a performance climate, and which did not appear to differ between men and women. Coaches and athletes are recommended to note that championship-specific physiological and psychological factors are important to develop in training and prior competition to improve both short- and long-term championship strategies.
109	Perceptions of the Coach-Athlete Relationship Predict the Attainment of Mastery Achievement Goals Six Months Later: A Two-Wave Longitudinal Study among F. A. Premier League Academy Soccer Players	Nicholls, AR; Earle, K; Earle, F; Madigan, DJ	FRONTIERS IN PSYCHOLOGY	2017	10.3389/fpsyg.2017.00684	S3	SPORTDiscus+WoS	All football teams that compete within the F. A. Premier League possess an academy, whose objective is to produce more and better home-grown players that are capable of playing professionally. These young players spend a large amount of time with their coach, but little is known about player's perception of the coach-athlete relationship within F. A. Premier League Academies. The objectives of this study were to examine whether perceptions of the coach-athlete relationship changed over six months and if the coach-athlete relationship predicted self-reported goal achievement among F. A. Premier League academy players. This study included cross-sectional (n = 104) and longitudinal (n = 52) assessments, in which academy soccer players completed a measure of the coach-athlete relationship and goal achievement across either one or two time periods. The cross-sectional data were subjected to bivariate correlations, whereas the longitudinal data were analyzed using multiple regressions. Perceptions of the coach-athlete relationship remained stable over time. The coach-athlete relationship predicted the achievement of mastery goals six months later. Enhancing the quality of the coach-athlete relationship among elite adolescent athletes appears to be a suitable way of maximizing mastery achievement goals, particularly among developmental athletes who participate in team sports.

110	How perceived autonomy support and controlling coach behaviors are related to well- and ill-being in elite soccer players: A within-person changes and between-person differences analysis	Cheval, B; Chalabaev, A; Quested, E; Courvoisier, DS; Sarrazin, P	PSYCHOLOGY OF SPORT AND EXERCISE	2017	10.1016/j.psychsport.2016.10.006	S3	Scopus+WoS	Objectives: Grounded in Basic Psychological Needs Theory (Ryan & Deci, 2002), this study examined the temporal relationships between perception of coaches' autonomy support and different facets of controlling behaviors, the satisfaction-frustration of athletes' basic needs, and subjective vitality, self-esteem and burnout in elite sportsmen. Methods: Participants (N = 110 males) from three elite youth soccer academies in northwest France completed a questionnaire on three occasions during the last three months of the competitive season. Results: Linear mixed models revealed that perceptions of coach-autonomy support and only two facets of controlling coach behaviors (excessive personal control and negative conditional regard) were related to basic need satisfaction-frustration, which in turn were related to the indices of well- and ill-being. In most cases, the relationships were observed both at the within- and between-person levels, but some were observed only at one level. Conclusions: The findings highlight the importance of considering the different facets of controlling coach behaviors separately and disaggregating the between-person and within-person effects. (C) 2016 Elsevier Ltd. All rights reserved.
111	Elite Athletes' Experience of Coping With Emotional Abuse in the Coach-Athlete Relationship	Kavanagh, E; Brown, L; Jones, I	JOURNAL OF APPLIED SPORT PSYCHOLOGY	2017	10.1080/10413200.2017.1298165	S1+S3	SPORTDiscus+Scopus+WoS	In this article, we explore the coping strategies used by elite athletes in response to emotional abuse experienced within the coach-athlete relationship. The athletes in this study adopted emotion- and avoidance-focused coping strategies to manage their feelings in the moment that emotional abuse occurred. Over time, athletes accessed support networks and engaged in sense making to rationalize their experiences. The potential of coping-level intervention to develop individual resources and to break the cycle of emotional abuse in sport is highlighted. We suggest that as primary agents of ensuring athlete's protection, sport psychologists need appropriate safeguarding training.
112	Managing player load in professional rugby union: a review of current knowledge and practices	Quarrie, KL; Raftery, M; Blackie, J; Cook, CJ; Fuller, CW; Gabbett, TJ; Gray, AJ; Gill, N; Hennessy, L; Kemp, S; Lambert, M; Nichol, R; Mellalieu, SD; Piscione, J; Stadelmann, J; Tucker, R	BRITISH JOURNAL OF SPORTS MEDICINE	2017	10.1136/bjsports-2016-096191	S3	WoS	Background The loads to which professional rugby players are subjected has been identified as a concern by coaches, players and administrators. In November 2014, World Rugby commissioned an expert group to identify the physical demands and non-physical load issues associated with participation in professional rugby. Objective To describe the current state of knowledge about the loads encountered by professional rugby players and the implications for their physical and mental health. Findings The group defined 'load' as it relates to professional rugby players as the total stressors and demands applied to the players. In the 2013-2014 seasons, 40% of professional players appeared in 20 matches or more, and 5% of players appeared in 30 matches or more. Matches account for similar to 5-11% of exposure to rugby-related activities (matches, team and individual training sessions) during professional competitions. The match injury rate is about 27 times higher than that in training. The working group surmised that players entering a new level of play, players with unresolved previous injuries, players who are relatively older and players who are subjected to rapid increases in load are probably at increased risk of injury. A mix of 'objective' and 'subjective' measures in conjunction with effective communication among team staff and between staff and players was held to be the best approach to monitoring and managing player loads. While comprehensive monitoring holds promise for individually addressing player loads, it brings with it ethical and legal responsibilities that rugby organisations need to address to ensure that players' personal information is adequately protected. Conclusions Administrators, broadcasters, team owners, team staff and the players themselves have important roles in balancing the desire to have the 'best players' on the field with the ongoing health of players. In contrast, the coaching, fitness and medical staff exert significant control over the activities, duration and intensity of training sessions. If load is a major risk factor for injury, then managing training loads should be an important element in enabling players to perform in a fit state as often as possible.
113	The Relationship Between Training Load and Injury in Men's Professional Basketball	Weiss, KJ; Allen, SV; McGuigan, MR; Whatman, CS	INTERNATIONAL JOURNAL OF SPORTS PHYSIOLOGY AND PERFORMANCE	2017	10.1123/ijspp.2016-0726	S3	WoS	Purpose: To establish the relationship between the acute: chronic workload ratio and lower-extremity overuse injuries in professional basketball players over the course of a competitive season. Methods: The acute: chronic workload ratio was determined by calculating the sum of the current week's session rating of perceived exertion of training load (acute load) and dividing it by the average weekly training load over the previous 4 wk (chronic load). All injuries were recorded weekly using a self-report injury questionnaire (Oslo Sports Trauma Research Center Injury Questionnaire(20)). Workload ratios were modeled against injury data using a logistic-regression model with unique intercepts for each player. Results: Substantially fewer team members were injured after workload ratios of 1 to 1.49 (36%) than with very low (<= 0.5; 54%), low (0.5-0.99; 51%), or high (>= 1.5; 59%) workload ratios. The regression model provided unique workload-injury trends for each player, but all mean differences in likelihood of being injured between workload ratios were unclear. Conclusions: Maintaining workload ratios of 1 to 1.5 may be optimal for athlete preparation in professional basketball. An individualized approach to modeling and monitoring the training load-injury relationship, along with a symptom-based injury-surveillance method, should help coaches and performance staff with individualized training-load planning and prescription and with developing athlete-specific recovery and rehabilitation strategies.

114	Scarred for the Rest of My Career? Career-Long Effects of Abusive Leadership on Professional Athlete Aggression and Task Performance	Carleton, EL; Barling, J; Christie, AM; Trivisonno, M; Tulloch, K; Beauchamp, MR	JOURNAL OF SPORT & EXERCISE PSYCHOLOGY	2016	10.1123/jsep.2015-0333	S1+S3	Scopus+WoS	Based on the contention that leadership has sustained effects on followers even after the leader follower relationship has ended, we investigated the career-long effects of abusive coach leadership on athlete aggression and task performance. Abusive leadership scores were derived from ratings by two independent raters' evaluations of coaches' biographies, and athlete aggression and task performance data were derived from objective sources. Data were obtained from players (N= 693) and coaches (N= 57) involved in the National Basketball Association (NBA) between the 2000-2001 and 2005-2006 seasons. Controlling for tenure, salary, team winning percentage, and absence due to injuries, multilevel modeling showed that exposure to abusive leadership influenced both the trajectory of psychological aggression and task performance over players' careers. These findings suggest that the effects of abusive leadership extend far longer than currently acknowledged, thus furthering our understanding of the nature and effects of abusive leadership.
115	The Mental Health of Elite Athletes: A Narrative Systematic Review	Rice, SM; Purcell, R; De Silva, S; Mawren, D; McGorry, PD; Parker, AG	SPORTS MEDICINE	2016	10.1007/s40279-016-0492-2	S1	WoS	Background The physical impacts of elite sport participation have been well documented; however, there is comparatively less research on the mental health and psychological wellbeing of elite athletes. Objective This review appraises the evidence base regarding the mental health and wellbeing of elite-level athletes, including the incidence and/or nature of mental ill-health and substance use. Methods A systematic search of the PubMed, EMBASE, SPORTDiscus, PsycINFO, Cochrane and Google Scholar databases, up to and including May 2015, was conducted. Results The search yielded a total of 2279 records. Following double screening, 60 studies were included. The findings suggested that elite athletes experience a broadly comparable risk of high-prevalence mental disorders (i.e. anxiety, depression) relative to the general population. Evidence regarding other mental health domains (i.e. eating disorders, substance use, stress and coping) is less consistent. These results are prefaced, however, by the outcome of the quality assessment of the included studies, which demonstrated that relatively few studies (25%) were well reported or methodologically rigorous. Furthermore, there is a lack of intervention-based research on this topic. Conclusion The evidence base regarding the mental health and wellbeing of elite athletes is limited by a paucity of high-quality, systematic studies. Nonetheless, the research demonstrates that this population is vulnerable to a range of mental health problems (including substance misuse), which may be related to both sporting factors (e.g. injury, overtraining and burnout) and non-sporting factors. More high-quality epidemiological and intervention studies are needed to inform optimal strategies to identify and respond to player mental health needs.
116	Predicting Self-Reported Illness for Professional Team-Sport Athletes	Thornton, HR; Delaney, JA; Duthie, GM; Scott, BR; Chivers, WJ; Sanctuary, CE; Dascombe, BJ	INTERNATIONAL JOURNAL OF SPORTS PHYSIOLOGY AND PERFORMANCE	2016	10.1123/ijspp.2015-0330	S3	WoS	Purpose: To identify contributing factors to the incidence of illness for professional team-sport athletes, using training load (TL), self-reported illness, and well-being data. Methods: Thirty-two professional rugby league players (26.0 +/- 4.8 y, 99.1 +/- 9.6 kg, 1.84 +/- 0.06 m) were recruited from the same club. Players participated in prescribed training and responded to a series of questionnaires to determine the presence of self-reported illness and markers of well-being. Internal TL was determined using the session rating of perceived exertion. These data were collected over 29 wk, across the preparatory and competition macrocycles. Results: The predictive models developed recognized increases in internal TL (strain values of >2282 AU, weekly TL >2786 AU, and monotony >0.78 AU) to best predict when athletes are at increased risk of self-reported illness. In addition, a reduction in overall well-being (<7.25 AU) in the presence of increased internal TL, as previously stated, was highlighted as a contributor to self-reported-illness occurrence. Conclusions: These results indicate that self-report data can be successfully used to provide a novel understanding of the interactions between competition-associated stressors experienced by professional team-sport athletes and their susceptibility to illness. This may help coaching staff more effectively monitor players during the season and potentially implement preventive measures to reduce the likelihood of illnesses occurring.
117	Emotion-centered and action-centered coping in elite sport: Task execution design approach	Hanin, Y; Hanina, M; Sasek, H; Kobilsek, A	INTERNATIONAL JOURNAL OF SPORTS SCIENCE & COACHING	2016	10.1177/1747954116654782	S3	WoS	Task execution design approach to optimization of athletic performance aims to help elite athletes and coaches to prevent underperformance in competitions. This individual-specific coping framework includes: (i) constraints-led competition environment; (ii) appraisals, (iii) decision making, and (iv) emergent performance patterns. Practical utility of the Task execution design approach is illustrated by a five-year participant observation case study involving an elite female shooter, her coach, and two consultants. This longitudinal study describes a systematic cooperation that identified several practical problems in competition, with a special reference to the athlete's transition from juniors' to seniors' category. The athlete developed three effective coping strategies: emotion-centered, action-centered, and combined (plans A, B, and C). These strategies were used depending on performer-environment relationship in the framework of ecological dynamics. The study shows how the athlete's needs in self-control changed during five years and were satisfied using the proposed Task execution design framework. The main emphasis was on the qualitative analysis of the experiential knowledge based on the athlete's and coach's self-perceptions and self-reflections. The case also illustrates how the athlete and her coach used the RLD and ALD principles in their preparation for competition. The validity of the Task execution design approach was tested by the practitioners across 14 different sports.

118	A Longitudinal Case Study of a Sport Psychology Intervention Designed to Facilitate Team Culture Change	Glaude, M; Alexander-Urquhart, D; Henderson, S; Sheppard, MB; Loughead, TM; Bloom, GA	SPORT EXERCISE AND PERFORMANCE PSYCHOLOGY	2026	10.1037/spy0000414	S1	WoS	The purpose of this mixed methods study was to design, implement, and evaluate a 13-week sport psychology intervention designed to facilitate team culture change of a poor-performing university football team. Participants were 74 male athletes who participated in a season-long intervention, including four culture workshops, two team-building sessions, and biweekly psychological skills training sessions. Data were collected and analyzed using a convergent parallel mixed methods design, incorporating quantitative measures of team cohesion at three time points and qualitative individual semistructured interviews at the end of the season. Quantitative results showed significant increases in team cohesion from pre- to midseason, with these gains maintained through the end of the season. Furthermore, between-group differences based on athlete tenure and position were explored, revealing that first-year athletes showed sharp increases in group integration (particularly Group Integration-Social), whereas players with four or more years of tenure reported lower baseline Group Integration-Social and more gradual change. Postseason qualitative interviews with six athletes highlighted how the intervention supported a player-driven cultural shift, with early workshops fostering buy-in and accountability and ongoing sessions reinforcing values, psychological safety, and team unity. Players described the season as a meaningful cultural rebuild marked by greater clarity, connection, and shared leadership. Overall, this study advances the empirical, conceptual, and methodological understanding of culture change in sport by addressing calls for theory-driven approaches and providing practical strategies to foster cohesive, value-based cultures while tracking their integration over time.
119	Beyond the podium: A Delphi approach aimed at empowering coaches with practical strategies to identify and support athlete mental health	Sankey, C; Brockett, C; Slattery, K; Lederman, O; Warmenhoven, J; Menzies-Stegbauer, T; Langan, E; Caperchione, CM	JOURNAL OF SPORTS SCIENCES	2026	10.1080/02640414.2025.2576311	S1	Scopus+WoS	This study aimed to establish expert consensus on approaches to assist coaches in both identifying mental health symptoms and facilitating appropriate referral of athletes to mental health professionals. A modified Delphi approach was adopted using three rounds of expert consultation through individual and anonymous survey responses, thereby reducing potential influences or biases from group opinions. An interdisciplinary expert panel of coaches (n = 15), athletes (n = 9), psychologists (n = 16), and performance support staff (n = 26) was recruited through Australian sports networks, inclusive of sport science, medicine, and coaching to participate. Iterative surveys involved open-ended and multiple-choice questions (Survey 1), as well as statement agreement ratings (Surveys 2 and 3). The threshold for agreement was set at 80%. Fifty-three items achieved consensus across four main domains, including the coach role and responsibilities, the referral process, athlete mental health plans, and mental health training and upskilling. Consensus outcomes highlighted the nuanced and dynamic approach required to effectively support athlete mental health. The strategies and guiding principles outlined can be used to inform the development of resources that can build the confidence, skills, and knowledge of coaches in identifying, supporting, and referring athletes who require mental health assistance.
120	Authentic leadership and well-being in sport: the mediating role of psychological safety and the moderating role of interpersonal violence	Hurst, P; Kavussanu, M	INTERNATIONAL JOURNAL OF SPORT AND EXERCISE PSYCHOLOGY	2026	10.1080/1612197X.2025.2477163	S1	WoS	Sport leaders, such as coaches, are critical in promoting psychological safety with subsequent effects on athlete well-being. However, many athletes experience interpersonal violence (e.g., psychological abuse), which can negatively influence their well-being and may attenuate the beneficial effects of positive coaching styles such as authentic leadership. Understanding the relationship between authentic leadership, psychological safety and athlete well-being after experiencing interpersonal violence in sport is important in designing effective safeguarding interventions and policy. In this study, we were the first to examine the indirect relationship between authentic leadership and well-being via psychological safety, and if the direct relationship between authentic leadership and well-being is moderated by interpersonal violence. Competitive athletes (N = 305; 52% female, 29.20 +/- 12.62 years old) completed an anonymous questionnaire measuring authentic leadership of their current coach, psychological safety, interpersonal violence, and well-being. Authentic leadership was indirectly related to athletes' well-being via psychological safety (effect size = 0.13, 95% CI = 0.07, 0.19), and the direct effect of authentic leadership on athletes' well-being was significant only when interpersonal violence scores were low or moderate. These findings suggest that when athletes perceive their coaches to be authentic leaders, they are more likely to feel psychologically safe, and in turn have a greater sense of well-being. However, authentic leaders are less likely to positively influence well-being when athletes have experienced interpersonal violence. A need exists to prevent interpersonal violence so that coaches displaying authentic leadership qualities can positively influence their athletes' well-being.

121	Effects of a six-week basketball-specific individual skills development program on mental toughness and sport competition anxiety	Yazici, A; Tasçioglu, R; Solmaz, S; Sari, I; Kan, EMR; Bayköse, N	FRONTIERS IN PSYCHOLOGY	2026	10.3389/fpsyg.2026.1794140	S2+S3	WoS	Introduction This quasi-experimental study aimed to investigate the impact of a six-week basketball-specific individual skills development program on mental toughness and competitive anxiety among young professional male basketball players competing in organized leagues. Mental toughness and anxiety are critical psychological factors that influence athletic performance, particularly in high-pressure sports like basketball. The study sought to bridge the gap in existing research by examining how individualized training programs can simultaneously enhance mental resilience and reduce anxiety levels during the competitive season. Methods Sixty-three male basketball players (M = 17.93, SD = 0.75), all of whom were officially registered players competing under club contracts and receiving financial compensation, were divided into experimental and control groups. The experimental group underwent a six-week tailored training program designed to enhance position-specific skills, while the control group participated in regular team training. Psychological assessments, including the Sports Mental Toughness Questionnaire (SMTQ-14) and the Sport Competition Anxiety Test (SCAT-A), were conducted before and after the intervention to measure changes in mental toughness and anxiety levels. Statistical analyses, including two-factor ANOVA and Mann-Whitney U tests, were used to evaluate the data. Results The experimental group showed significant improvements in mental toughness subscales (confidence, consistency, and control) and a significant reduction in sport competition anxiety compared to the control group ($p < 0.05$). Effect sizes indicated moderate to large improvements in psychological outcomes, suggesting that the individualized skills development program effectively enhanced mental resilience and reduced anxiety among participants. Discussion The findings highlight the importance of tailored training programs in fostering psychological resilience and reducing anxiety in basketball players. The results align with previous research emphasizing the role of structured, individualized training in improving mental toughness and managing competitive anxiety. The study underscores the need for coaches to integrate psychological skill development into training regimens, particularly during the competitive season, to optimize both performance and mental wellbeing. Conclusion This study demonstrates that a six-week basketball-specific individual skills development program enhances players' mental toughness and reduces sport
122	Digital twin for Taekwondo athletes: integrating sports nutrition and psychological readiness using artificial intelligence	Amawi, AT; Grivas, GV; Alkasasbeh, WJ	FRONTIERS IN PUBLIC HEALTH	2026	10.3389/fpubh.2026.1822194	S2	Scopus+WoS	Background Taekwondo is a high-intensity Olympic combat sport that requires the integration of physical performance, tactical decision-making, and psychological resilience. Athletes face unique challenges such as rapid weight management, fatigue accumulation, injury risk, and competitive anxiety. While sports nutrition and psychological readiness are critical determinants of performance, they are often addressed separately, creating a gap in holistic, individualized athlete monitoring systems.Methods This narrative review synthesizes interdisciplinary evidence from sport science, nutrition, psychology, and artificial intelligence. A structured literature search was conducted across PubMed, Scopus, Web of Science, and Google Scholar, focusing on studies related to Taekwondo performance, weight-category nutrition strategies, psychological readiness, and AI-driven technologies including wearable systems, machine learning, and digital twin frameworks.Results The findings indicate that AI-driven digital twin technology enables the integration of multidimensional athlete data, including nutritional intake, psychological state, training load, and physiological biomarkers (e.g., HRV and cortisol). These systems can generate actionable outputs such as readiness scoring, personalized nutrition strategies, early detection of fatigue and stress dysregulation, and prediction of injury or overtraining risk.Conclusion Digital twin technology represents a promising framework for transforming Taekwondo athlete management from fragmented monitoring to a holistic, data-driven approach. Practically, this may support coaches in making real-time decisions regarding training load, weight management, recovery, and psychological interventions. However, further empirical validation, ethical considerations, and applied research are required to support real-world implementation in elite combat sport environments.
123	Perilous games: Gendered moral disengagement, winning culture, and COVID-19 safety in NCAA division I athletics	Hollis, LP; Oaxaca, GC	INTERNATIONAL JOURNAL OF SPORTS SCIENCE & COACHING	2026	10.1177/17479541261445526	S2	WoS	Building on international research, which applies moral disengagement theory in sport, this study examined NCAA Division I coaches' adherence to COVID-19 safety recommendations in the University of Pittsburgh Medical Center (UPMC) Medical Sport Playbook. These protocols included social distancing, sanitation, personal protective equipment (PPE), pre-participation exams, and daily screening. Coaches (n = 179) across 29 sports completed a survey regarding compliance with this guidance. The following research questions guided the analysis: RQ1: What is the relationship between win/loss records and compliance with COVID-19 safety protocols? H1: Teams with sustained winning records (>= 80% win rate) were expected to demonstrate higher compliance with COVID-19 safety protocols. RQ2: What is the relationship between team gender and compliance with these protocols? H2: Women's teams were expected to demonstrate greater compliance with COVID-19 protocols. Guided by Bandura's moral disengagement theory, we explored how competitive pressure and coaching identity may rationalize noncompliance with public health mandates. Regression analyses revealed that teams with higher win rates were significantly less likely to comply with sanitation and daily screening protocols, while women's teams demonstrated significantly greater adherence to social distancing. Compliance with PPE use and pre-participation exams was inconsistent across all teams. Overall, high-performance programs were less likely to follow COVID-19 safety protocols, highlighting the influence of moral disengagement, gendered expectations, and performance culture on health-related decision-making. These findings extend understanding of how performance culture, moral disengagement, and gendered leadership norms influence health compliance in elite sport environments worldwide.

124	Sudden cardiac arrest preparedness in African football: evidence from Cameroon and implications for global sport	Tankeng, LT; Akwa, GM; Ossaih, AULS; Bilong, VAN; Tasong, LAM; Ghassi, HT; Ndom, MS; Douryang, M	BRITISH JOURNAL OF SPORTS MEDICINE	2026	10.1136/bjsports-2025-110869	S2	WoS	Objective Sudden cardiac arrest (SCA) remains the leading cause of sport-related death worldwide, yet survival outcomes in Africa are critically poor. We conducted a formative assessment of the knowledge, attitudes and practices of professional football stakeholders in Cameroon as a model for identifying system-level gaps in SCA preparedness in low-resource sport settings.Methods We conducted a cross-sectional survey during the 2024/2025 season across five pre-competition medical centres in Cameroon. Eligible participants were players, referees, coaches and physiotherapists from Elite 1, Elite 2 and the Female Super League who had two or more seasons of professional experience. A validated questionnaire, adapted from international guidelines, was used to assess sociodemographic data, knowledge, attitudes and practices. Data were analysed descriptively with subgroup comparisons using SPSS V.20.Results A total of 745 participants were enrolled: 536 players, 90 referees, 84 coaches and 35 physiotherapists. Most (66.4%) demonstrated poor knowledge of SCA, only 12% reported confidence in cardiopulmonary resuscitation (CPR) or automated external defibrillator (AED) use and referees showed the lowest preparedness scores despite their critical first-responder role. While one-third expressed positive attitudes, self-reported barriers to providing CPR included fear of harm (66.3%) and legal concerns (78.3%). Only 33.4% reported good practices, with significant gaps in AED access, CPR training and emergency action plan awareness.Conclusion This assessment identified critical gaps in SCA preparedness in Cameroonian football, exposing gaps that likely extend across Africa and similar environments. Compulsory CPR/AED training, AED availability and enforceable emergency action plans are urgently needed to align African football with international best practice and strengthen the survival chain.
125	The role of leaders in the communal coping process in sports teams: A social network analysis perspective	Mahot, K; Lienhart, N; Doron, J	JOURNAL OF APPLIED SPORT PSYCHOLOGY	2026	10.1080/10413200.2026.2613793	S2	WoS	Elite ice hockey players experience intense competitive pressure requiring team coordination to manage these challenges. Understanding how communal coping occurs within teams and its impact on team performance in high-pressure sports environments is crucial. This study first explores the role of identity leaders in initiating communal coping dimensions within their team. Second, it aims to evaluate how communal coping relates to subjective team performance under pressure. Data were collected from 15 elite ice hockey teams across France, Switzerland, and Canada, comprising 395 male athletes (Mage = 21.85, SD = 6.38). Social network analysis was used to examine relationships between identity leaders and communal coping dimensions including problem-focused communal efforts, communal management of emotions, and communal-goal withdrawal. Perceived team performance under pressure was assessed. QAP analyses indicate that identity leaders are significantly and positively associated with problem-focused communal efforts and communal management of emotions. In contrast, the relationship between identity leaders and communal-goal withdrawal varied across teams, indicating contextual variability. The results regarding correlations between communal coping dimensions and subjective team performance under pressure indicate that problem-focused communal efforts and communal management of emotions are both significantly and positively associated with subjective team performance under pressure. Conversely, communal-goal withdrawal was negatively associated with subjective team performance under pressure. These findings underscore the critical role of identity leaders in implementing communal coping. Moreover, this study highlights the relationships between communal coping dimensions and subjective team performance under pressure, advancing theoretical insights into social dynamics in elite team sports under pressure.Lay SummaryElite ice hockey players cope with intense pressure which requires team stress management. Results highlight the critical role of leaders in the collective management of high-pressure sports. They also provide preliminary support for the relationship between communal coping dimensions and subjective team performance.
126	Implications of perceived empowering motivational climate on athletes' goal-regulatory processes: A longitudinal analysis	Martínez-González, N; Atienza, FL; Balaguer, I	JOURNAL OF SPORTS SCIENCES	2026	10.1080/02640414.2025.2598159	S3	WoS	This study explores how athletes' perceptions of empowering climates can influence athletes' goal regulatory processes over the competitive season. The purpose of this research was to test, using a longitudinal design, the relationship between athletes' perceptions of an empowering climate, goal motives, and goal-directed effort. A total of 112 university athletes (M = 20.90; SD = 2.79) completed a multi-section questionnaire at two time points during the season. Changes in perceptions of an empowering motivational climate positively predicted changes in autonomous goal motives (but not controlled ones), which in turn led to positive changes in goal-directed effort. These results suggest that athletes who perceive a climate of empowerment on the part of their coach tend to pursue goals autonomously, leading to greater effort directed towards those goals. Overall, the findings provide information on the implications of athletes' perception of the empowering climates created by their coaches for the autonomous motivation underlying their goals, as well as for enhancing their effort sustainability and facilitating their goal attainment in high-level sporting contexts. In addition, the study supports the positive implications of perceived empowering climates in relation to athletes' pursuit of adaptive goals when examined over time.

127	Burnout Syndrome in Sport-Related Jobs: A Meta-analysis of Recent Evidence	Viego, V; Camilo, JAD; Santos, BIA; Lima-Sena, I; Hamodi, C	SPORTS MEDICINE	2026	10.1007/s40279-026-02463-y	S3	WoS	BackgroundBurnout syndrome in sports is receiving increasing attention in the empirical literature applying psychometric tools. Since 2019, the number of scientific publications has doubled. This growth has increasingly dissociated athletes from their status as workers and burnout is rarely conceived as a phenomenon emerging from a working relationship. This study aims to meta-analyze the empirical measurements of burnout using scales in athletes and occupations related to professional sports during 2014-2023.ResultsThe initial search detected 996 studies. After a screening guided by PRISMA principles, we meta-analyzed 113 independent studies comprising 133 burnout measurements from 35,059 athletes, coaches, and referees across 29 countries. The results show a generalized use of the Athlete Burnout Questionnaire (ABQ), a notable heterogeneity in the estimates, signs of publication biases in some specific subscales, higher mean scores in personal accomplishment than other burnout dimensions, a decreasing trend in global scores over time, a higher burnout prevalence in developed countries, and different mean scores according to the scale applied.ConclusionsThe results highlight the need to continue improving the existing psychometric tools and focus interventions on the perception of accomplishment to reduce burnout incidence.
128	Development and evaluation of a coach-athlete relationship intervention program	Davis, L; Sommer, M; Brown, DJ; Jowett, S; Ekelund, R; Mårs, T; Wachsmuth, S	PSYCHOLOGY OF SPORT AND EXERCISE	2026	10.1016/j.psychsport.2026.103141	S3	Scopus+WoS	This study aimed to develop, implement and evaluate the effectiveness and feasibility of a coach-athlete relationship intervention program. The intervention targeted improvements in relationship quality and individual and team outcomes over time (i.e., thriving, basic psychological need satisfaction, and collective efficacy). Using a nonrandomized cluster-controlled trial design and mixed methods evaluation, 16 sports teams (45 coaches, 328 athletes) were assigned to an intervention or a no-training control condition. Following baseline assessments, intervention teams participated in two workshops, four weeks apart and coaches and athletes were encouraged to apply strategies taught during practice and complete bi-weekly diary logs. Follow-up assessments were completed at 4, 8 and 16 weeks post baseline. Focus groups were held after follow up to evaluate feasibility. Intervention effects were assessed using two-level growth models, with athletes nested within teams. Overall, no significant intervention effects were found over time for coaches or athletes' perceptions of relationship quality, or for athlete outcomes of thriving and collective efficacy. However, a significant decrease in relatedness was observed in athletes' basic psychological need satisfaction. Content analysis of focus group interviews highlighted participants' experiences of the intervention's relevance, delivery and implementation into sport practice. While participants emphasized the importance of the intervention's content, they also forwarded suggestions to improve delivery and transfer of skills into their daily sport practice. These insights shed light on potential reasons for the limited quantitative effects. While the current intervention did not produce significant effects, the study lays important groundwork for future research aiming to strengthen the coach-athlete relationship.
129	How does need satisfaction mediate the relation between harmonious passion and positive affect in coaches and athletes? A dyadic longitudinal study in coach-athlete dyads	Denul, A; Haerens, L; Loeys, T	INTERNATIONAL JOURNAL OF SPORTS SCIENCE & COACHING	2026	10.1177/17479541251366906	S3	WoS	Previous research has linked harmonious passion to positive affect in both coaches and athletes. Additionally, need satisfaction is suggested to mediate this relationship. However, these studies often relied on cross-sectional designs or failed to account for the inherent dyadic relationship between coaches and athletes. Addressing these limitations, our study combines a longitudinal design with a dyadic perspective to provide a more comprehensive understanding of the processes underlying positive affect in coaches and athletes. Using the longitudinal actor-partner interdependence mediation model, we investigated actor and partner effects for coaches and athletes at both the between- and within-person level. Our findings reveal that, for both coaches and athletes, their own harmonious passion is positively associated with their own positive affect at both the between- and within-person level. Notably, for coaches, this relationship is partly mediated by their own need satisfaction. Moreover, we found preliminary evidence for partner effects through coaches' need satisfaction at both the between- and within-person level. In summary, the longitudinal design suggests that harmonious passion is important for both coaches and athletes, not only in terms of between-person differences but also in terms of changes within an individual over a season. Additionally, the dyadic perspective yields valuable insights into potential partner effects via coaches' need satisfaction.

130	The relationship between China university basketball association athletes' mindfulness, self-efficacy in sports training, and training competition satisfaction: a cross-sectional and longitudinal study	Zhao, YL; Liu, JW; Yang, XB; Wu, SM	BMC PSYCHOLOGY	2026	10.1186/s40359-026-04069-5	S3	WoS	Background This study investigates the relationship and underlying mechanisms among mindfulness, self-efficacy in sports training, and training competition satisfaction among China University Basketball Association (CUBA) athletes through cross-sectional surveys and longitudinal experimental research. These findings provide valuable insights into the healthy development of college athletes. Methods Study I was a cross-sectional study. A total of 684 CUBA athletes were surveyed using the Mindful Attention Awareness Scale, the College Student Physical Education Learning Self-Efficacy Scale, and the Training Competition Satisfaction Scale. Correlation and regression analyses were performed on the variables, and the bootstrap method was used to test the mediating effect. Study 2 adopted a 2 (experimental conditions) x 2 (time points) mixed experimental design, randomly assigning 32 CUBA athletes to either a mindfulness training group or a control group (16 people in each group). The experimental group underwent an eight-week mindfulness intervention. In contrast, the control group simultaneously received standard technical training and underwent psychological assessments before and after the training. Results In Study 1, the mindfulness level of CUBA athletes had a significant positive predictive effect on their self-efficacy in sports training and training competition satisfaction ($p < 0.001$) The self-efficacy in sports training partially mediated the relationship between mindfulness and training competition satisfaction, with an indirect effect value of 0.210, accounting for 45.3% of the total effect. In Study 2, compared to the control group, the mindfulness level, self-efficacy in sports training, and training competition satisfaction of athletes in the mindfulness training group significantly improved over time ($p < 0.001$). Conclusions Mindfulness can have a direct effect on training competition satisfaction, as well as an indirect effect through self-efficacy in sports training, making it an effective intervention method to enhance motor psychological skills.
131	Psychometric properties of the French athlete version of the coach-athlete relationship questionnaire (Fr-CART-Q)	Roux, V; Trouilloud, D	INTERNATIONAL JOURNAL OF SPORTS SCIENCE & COACHING	2026	10.1177/17479541251399639	S3	SPORTDiscus+WoS	Two studies were conducted to examine the psychometric properties of the French athlete version of the Coach-Athlete Relationship Questionnaire (Fr-CART-Q) among two independent samples of French athletes. Study 1 evaluated the reliability, factorial structure, and convergent, discriminant, and criterion validities of this scale among 442 French athletes (65.8% men; Mage = 21.66, SD = 4.82) involved in various sports. Study 2 investigated the same psychometric properties, as well as temporal invariance, among a specific sample of 286 adolescent handball players (58.4% men; Mage =15.39 years, SD = 1.01) involved in intensive training centers. Both studies reveal that the psychometric properties of the Fr-CART-Q are satisfactory. Reliability indices and factorial structures confirm the multidimensional and hierarchical structure of the Fr-CART-Q. Temporial invariance analysis reveals that the factorial structure of this scale remains consistent over time. Criterion validity is satisfactory and the quality of the coach-athlete relationship is positively associated with athletes' perceived competence. Overall, these two studies indicate that the Fr-CART-Q can be used with French athletes.
132	Are winning teams more attentive to coach feedback and correction? A longitudinal study across a national-level volleyball season	Malisse, F; Van Puyenbroeck, S; Hofmans, J; Vande Broek, G	INTERNATIONAL JOURNAL OF SPORT AND EXERCISE PSYCHOLOGY	2026	10.1080/1612197X.2026.2652876	S3	WoS	In performance-oriented team sports, coaches rely on their power to shape athlete behaviour and optimise team performance. Yet, frequent coach dismissals suggest this influence may be closely tied to team results. However, the empirical relationship between team performance and coach power has received limited attention. Furthermore, existing work on coach power has mainly examined why, rather than whether athletes actually attend to the coach's input and how this evolves over time. Therefore, this study examined how team attentiveness to coach feedback and correction relates to team performance across a season. All 13 male second-division volleyball teams in Belgium (N = 160 athletes) were assessed at four timepoints across a 9-month season: preseason start, competition start, mid-season, and season end. Athletes rated their team's subjective performance and attentiveness to coach feedback and correction. An expectations-adjusted objective performance (EAOP) score was calculated by comparing game results to teams' ranking expectations. Using three-level linear mixed-effects models, we tested our hypotheses. Only subjective performance predicted higher attentiveness (beta = 0.25, $p < .001$). Team attentiveness declined throughout the season (beta = -0.79, $p < .001$), even when subjective performance remained high. However, high perceived goal achievement buffered the negative impact of poor EAOP on attentiveness (beta = -0.04, $p = .024$). These findings suggest that, although team attentiveness to coach feedback and correction is positively tied to subjective performance, it declines towards season end regardless of performance. However, fostering high perceived goal achievement may help coaches sustain their teams' attentiveness despite objective underperformance.

133	Early Sport Specialization and Intense Training in Junior Tennis Players: A Sport-Specific Review	Thurber, L; Kantrowitz, DE; Wang, KC; Jayanthi, N; Colvin, A	SPORTS HEALTH-A MULTIDISCIPLINARY APPROACH	2026	10.1177/19417381251393642	S3	WoS	Context: Early sports participation can provide significant physical, psychosocial, and mental benefits for young athletes. Sports engagement can be via sport sampling or sport specialization. Sport specialization is often encouraged by parents and coaches as it is perceived as a mechanism for achieving elite level play, particularly in tennis. Evidence Acquisition: A search on PubMed, Google Scholar, and Science Direct was conducted for pertinent peer-reviewed studies on sport specialization and overuse injuries in youth athletics and junior tennis using key terms including junior tennis, youth sports, sport specialization, and overuse injury. Study Design: Clinical review. Level of Evidence: Level 5. Results: Early sport specialization leads to increased injury risk, decreased athletic career longevity, and higher incidence of burnout without demonstrating significant benefits in terms of improving peak sports performance or the likelihood of reaching elite play. Conclusion: Youth athletes should avoid specializing in a single sport before age 12, avoid training more hours per week than their age in years, and limit training to 16 hours per week maximum. Specific to tennis, junior players should be at least 12 years old for tournament play, compete in no more than 2 tournaments per month or 12 per year, engage in a maximum of 12 hours per week of organized tennis, a minimum of 2 hours per week of injury prevention training, and consider another off-season sport. Injury may be more likely with early sport specialization, early intense training (before middle or late adolescence) and annual match volume (>40 matches annually). Finally, we provide novel recommendations for safe volumes of training, matches, and tournaments for junior tennis players based on their age and level of play. Strength-of-Recommendation Taxonomy (SORT): B - recommendations based on inconsistent or limited-quality patient-oriented evidence.
134	Guiding growth: the influence of coach-created climate on passion, athletic identity, and perfectionism in teen female athletes	Martin, EM; Horn, TS	INTERNATIONAL JOURNAL OF SPORT AND EXERCISE PSYCHOLOGY	2026	10.1080/1612197X.2025.2481420	S3	WoS	Coaches who create a predominantly mastery-oriented motivational climate where self-improvement and individual mastery are emphasised might see different outcomes in relation to their athletes' psychosocial well-being than would coaches who create a predominantly performance-oriented motivational climate that emphasises intra-team competition and peer comparison. Three psychosocial variables that might be impacted by the coach-created motivational climate include passion, athletic identity, and perfectionism. This study was conducted to examine potential links between the coach-initiated motivational climate and these three dispositional variables in a sample of older adolescent athletes. Self-report questionnaires were administered to 224 female athletes ranging in age from 16 to 18 years. Data were analysed using both variable-centred (canonical correlation) and person-centred (cluster analyses with follow-up multivariate comparison) procedures. Obtained results revealed that a task-oriented team climate was positively associated with high levels of harmonious passion and perfectionistic strivings while a performance-oriented climate was positively linked to perfectionistic concerns. However, a mixture of the two climates did show some positive effects, especially regarding athletes' levels of perfectionistic strivings. These results provide support for the idea that the type of team climate, as initiated by coaches, is related to the psychosocial well-being of their adolescent athletes. However, additional research is needed to investigate these relationships in athletes at different developmental and competitive levels of play.
135	From social identity to team performance: Linking transactive memory systems and teamwork	Ozlu, C; Sezer, U; Durdubas, D; Koruc, Z	PSYCHOLOGY OF SPORT AND EXERCISE	2026	10.1016/j.psychsport.2025.103007	S3	Scopus+WoS	Cultivating a shared team identity fosters individuals' sense of belonging and strengthens their connection to the team. Previous studies have demonstrated that having a shared team identity and strongly identified individuals within the group are associated with various positive outcomes. Among these positive outcomes, increasing evidence from the organizational field suggests that a shared identity may serve as a foundational mechanism in the emergence of transactive memory systems (TMS). TMS refers to shared cognitive structures that enable team members to encode, store, and retrieve information collectively. The main objective of this study was to examine the relationship between team identification and transactive memory systems, as well as to investigate their effects on teamwork behaviors and overall performance of sport teams in unique tournament settings. A 3-wave longitudinal research design was employed to examine these associations, involving 167 athletes across 15 teams. Structural equation modelling indicated a significant relationship between team identification and transactive memory systems in the pre-tournament phase. Furthermore, pre-tournament transactive memory systems predicted teamwork behaviors during the tournament, positively influencing overall team performance. Our exploratory findings underscore how the short duration and high-pressure nature of the tournament differentially shaped teams' adaptive responses. Specifically, only mid-ranking teams showed significant improvements in adjustment behaviours from pre-tournament to mid-tournament, whereas both lower-and higherranking teams exhibited non-significant change. Based on these findings, to optimize a team's performance in a time-limited context such as tournaments, sport psychologists and coaches are encouraged to cultivate a shared team identity through social identity focused workshops or identity leadership interventions, and social activities with a heightened sense of psychological safety (e.g. open discussions for role clarity), which can develop transactive memory systems and, in turn, enhance teamwork behaviors and overall team performance. Consequently, the present study aims to adopt a collective-cognitive approach to the link between social identity and team performance in a sporting context, and to shed light on how team functioning unfolds under time-constrained conditions.

136	Perceived Coach and Teammate Weight/Shape-Related Beliefs and Disordered Eating in NCAA Swimmers	Pinson, CK; Fox, KR; Frank, GKW; Hooley, JM; Javaras, KN	INTERNATIONAL JOURNAL OF EATING DISORDERS	2026	10.1002/eat.70040	S3	WoS	Objective The distinct associations of coaches' and teammates' beliefs about ideal body weight/shape with athletes' disordered eating remain unclear. This study examined how athletes' perceptions of their coaches' and teammates' weight- and shape-related beliefs were associated with eating disorder symptoms and body dissatisfaction among National Collegiate Athletic Association (NCAA) swimmers. We hypothesized that both perceived coaches' and teammates' beliefs would be significantly associated with eating disorder symptoms and body dissatisfaction.Method Participants were 190 in-season NCAA swimmers (81% female) who completed a newly developed brief questionnaire designed to assess perceived coach and teammate weight/shape-related beliefs (Coach and Teammate Weight/Shape Beliefs Questionnaire; CTWSBQ), as well as scales assessing eating disorder symptoms (Eating Disorder Examination-Questionnaire, EDE-Q) and thinness- and muscularity-related body dissatisfaction (ATHLETE Questionnaire Your Body and Sports subscale and Drive for Muscularity Scale, respectively). Multiple linear regression was used to model disordered eating as a function of CTWSBQ Teammate and Coach scores and covariates.Results Only the CTWSBQ Teammate score was significantly associated with EDE-Q Global (Delta R2 = 0.04) and ATHLETE (Delta R2 = 0.04) scores after adjustment for both demographic and psychological covariates. CTWSBQ Coach, but not Teammate, scores were significantly associated with Drive for Muscularity scores (Delta R2 = 0.02) after adjustment for covariates.Discussion Findings from this cross-sectional study suggest distinct patterns of association for perceived teammate versus coach weight/shape-related beliefs across eating disorder symptoms, thinness-related concerns, and muscularity-related concerns in NCAA swimmers. Future work should test these patterns prospectively and determine whether they can inform prevention and education content for athletes and coaches.
137	The Relationship Between the Somatosensory System and Growth and Maturation in Pathway Triathletes; a Pilot Study	Fitch, AS; Mara, J; Waddington, G	PERCEPTUAL AND MOTOR SKILLS	2026	10.1177/00315125261459743	S3	WoS	Introduction Growth and maturation during adolescence can affect the somatosensory system, potentially impacting coordination and proprioception. Optimal proprioception is important for performance and preventing lower limb injury. This study investigates the relationship between the somatosensory function, biological maturation and growth tempo in youth pathway triathletes to enable coaches and healthcare professionals to optimise training programs for long term injury prevention and enhance subsequent performance through key stages of development.Methods Fifty three pathway triathletes (aged 12-18 years) were assessed over two separate time points during the triathlon season. At each time point data was collected on growth (height and mass) and maturation (bone age and maturity offset), and somatosensory function using the Active movement extent discrimination assessment (AMEDA) assessment.Results AMEDA scores improved with increasing maturity offset in both males and females. Females had reduced symmetry in earlier adolescence and a decline in somatosensory function mid-adolescence compared to male triathletes. There was a weak negative association observed between faster growth tempo and lower somatosensory performance.Conclusion This study supports the need for youth triathletes training programs to account for athletes sex, stage and tempo of biological maturation. Incorporating lower limb focused proprioception training starting pre PHV may support injury prevention and enhance long term performance outcomes as triathletes progress through the athletic pathway.
138	Artificial intelligence in sport psychology: Implications for the identification and development of talent	Sartor, F; Rosito, SA; Raimo, M; Beato, M; Bertollo, M	PSYCHOLOGY OF SPORT AND EXERCISE	2026	10.1016/j.psychsport.2025.102977	S3	WoS	Artificial Intelligence (AI), defined as computational systems capable of executing tasks requiring human-like intelligence, such as reasoning, planning, learning, perception, and interaction, represents a transformative paradigm within sport psychology. Machine Learning, a key subdomain of AI that enables pattern recognition and predictive modeling from data, has demonstrated significant efficacy in performance prediction and talent identification across different sports, as evidenced by Baker et al. (2025) in their review. However, the implementation of these technologies faces significant hurdles, critically concerning data availability, quality, ownership, and the inherent challenges of labeling for supervised learning. Longitudinal studies are further complicated by missing data and dataset imbalance, particularly concerning outcomes such as the rare development of talent into top-tier athletes, which can lead to biased models with poor generalizability. Standardized development frameworks, such as the Software Development Life Cycle, are advocated to mitigate these risks. Future directions emphasize human-machine interaction, such as conversational AI for decision support and digital twins for athlete modeling. Nevertheless, psychological and subjective factors, like coach judgment, athlete attitudes are critical yet currently underrepresented. AI will be playing a crucial role in enhancing communication between domain experts (coaches, scouts) and complex black-box systems, building trust for collaborative talent development. Future advancements should prioritize the integration of psychological constructs through idiosyncratic and idiographic approaches, while exploring deep learning for safeguarding applications.

139	Developing Equestrian Athlete Confidence After Experiencing the Setback of a Fall	Collins, A; Smith, MJ	SPORT EXERCISE AND PERFORMANCE PSYCHOLOGY	2026	10.1037/spy0000411	S3	WoS	Confidence is a key psychological quality for athletes' performance success. Researchers have previously examined sources from which athletes gain their confidence; however, limited research has considered the coach's role in rebuilding athlete confidence when returning to sport after dealing with setbacks. Thus, the purpose of the present study was to understand how coaches can work effectively with athletes to build confidence after a setback, considering the specific setback experienced by a rider from falling off a horse. To gain multiple perspectives, participants were six riders and six coaches (10 females and two males). Initially, participants wrote reflective letters to their younger selves on their experiences around the fall and consequent issues with confidence, with the content from these letters used to stimulate follow-up interviews. Three main themes were developed to represent the process of working effectively with riders to rebuild confidence: providing the foundation for building confidence, facilitating an understanding and awareness, and creating change in rider approach. These results informed the development of a temporal framework that suggests three stages that practitioners could implement into their practice. Findings were presented back to a further selection of coaches (n = 9) and sport psychologists (n = 3) as part of a member/external reflection process, to consider how these results might inform applied practice when working with riders after experiencing setbacks. The results are discussed regarding the practical implications for informing practitioners on how to work with riders who have lost their confidence in this context.
140	Crossing the line: Players perspectives on the leap from junior to senior football	Tomás, J; Sousa, H; Araújo, D; Field, A; Ribeiro, J; Sarmento, H	INTERNATIONAL JOURNAL OF SPORTS SCIENCE & COACHING	2026	10.1177/17479541261441668	S3	WoS	The transition from junior to senior is widely regarded as one of the most critical and challenging stages in the long-term talent development pathway. Despite its career-defining nature, this phase remains underexplored, particularly regarding the challenges faced by promising young players. This study investigated players' perceptions of the junior-to-senior transition through semi-structured interviews with 20 Portuguese football players: (1) 10 classified as successful players (39.3 +/- 11.96 years; 13.4 +/- 7.02 professional seasons; 5.8 +/- 3.48 career clubs), and (2) 10 classified as unsuccessful (35.6 +/- 12.22 years; 2.5 +/- 2.59 professional seasons; 2.2 +/- 2.14 career clubs). Success was defined as completing at least one full season in a top-tier European professional league. Interview questions addressed priorities in sporting experiences, psychosocial factors, and physical attributes. Interviews were audio-recorded, transcribed, and thematically analysed. Findings suggest that in a performance-driven sporting context, successful transition requires ambition, discipline, resilience, and self-reflection. Outcomes are strongly shaped by coach-player relationships, competitive environment, and the perceived importance of academic education. Persistent barriers include unrealistic expectations, external pressures, limited playing time, and the overemphasis on biological maturation and relative age effects. The influence of close social networks was found to be ambivalent, highlighting the need for gradual, personalized transition approaches supported by conscious and adaptive leadership.
141	Active success drives momentum, but late-stage errors destroy it: a real-time analysis of volleyball	Asai, Y; Okuda, T; Shide, N; Kida, N	FRONTIERS IN PSYCHOLOGY	2026	10.3389/fpsyg.2026.1839311	S3	WoS	Introduction Psychological momentum is a dynamic system that characterizes the psychological states of individuals progressing toward or regressing from goal attainment. As research has largely relied on controlled laboratory settings or artificial scenarios, empirical evidence from actual athletic competitions remains limited. Therefore, this study aimed to comprehensively elucidate how play content, game stages, and player expertise interact to dictate the micro-dynamics of momentum among actors during a real match.Methods Real-time measurements were conducted with 25 male high-school volleyball players during practice matches to ensure high ecological validity. Participants used voice recorders during natural intervals between rallies to verbally rate their momentum fluctuations. A dataset comprising 2,103 observations was analyzed using a linear mixed model to evaluate the fixed effects of expertise, game stage, play content (active success versus opponent error), and rally outcomes. Because the model did not include the lagged dependent variable, the first-order autoregressive [AR(1)] residual covariance structure was used only to address potential serial correlation in the residuals across adjacent rallies, rather than to model temporal carryover in psychological momentum itself.Results Scoring through a team's active success yielded significantly higher momentum improvements than scoring through an opponent's error across all game stages. In the late stages of a set, conceding a point because of a self-induced error resulted in a significantly more severe deterioration of momentum than conceding a point through an opponent's active success. Furthermore, advanced players exhibited a significantly greater decrease in momentum following lost points than intermediate players.Discussion These findings suggest that momentum fluctuations are highly sensitive to players' internal attributions of events, particularly the high mutability and regret associated with late-stage errors. Advanced players' acute sensitivity to conceded points highlights the phenomenon of proxy agency and the risk of proxy dilemmas among less experienced teammates. Practically, coaches can mitigate this overreliance and enhance self-agency by assigning specific, specialized roles, such as defensive pillars or high-set providers, to every player, ensuring that all participants can exercise personal agency and earn mutual trust within the team.

142	An evaluation of the hot-hand hypothesis in the NBA when accounting for shot difficulty	Kondur, A; Shen, WN	INTERNATIONAL JOURNAL OF SPORTS SCIENCE & COACHING	2026	10.1177/17479541251374788	S3	WoS	The hot-hand hypothesis suggests that athletes can experience periods of exceptional performance where the success of their next action is influenced by previous successes. This study examines the hot-hand phenomenon in the context of the NBA, focusing on shot difficulty as a critical factor. Previous research has yielded conflicting results, often ignoring the varying difficulty of shots and the role of defensive pressure. Utilizing shot log data from the 2014-2015 NBA season, this paper introduces new models that account for shot difficulty with defender proximity. By employing XGBoost classifiers and difficulty-weighted metrics, we find that while traditional hot-hand effects are minimal, adjusting for shot difficulty reveals a reverse hot-hand effect: players tend to perform worse following streaks of successful but difficult shots. This research provides a nuanced understanding of the hot-hand phenomenon, highlighting the importance of considering shot difficulty in sports performance analysis. These findings have implications for coaching strategies and the psychological interpretation of performance streaks in competitive basketball.
143	Motivational Climate Collaboration Between a Collegiate Native American Volleyball Program and Sport Psychology Researchers	Claunch, JL; Fry, MD; Brown, TC	SPORT PSYCHOLOGIST	2026	10.1123/tsp.2025-0028	S3	WoS	This study examined the outcomes of a caring, task-involving climate collaboration in a Native American women's collegiate volleyball team. Grounded in achievement goal perspective theory and a caring framework, the collaboration was shaped by a needs assessment completed the prior spring semester. In the subsequent fall season, the lead researcher provided continuous education and support to the head coach and athletes to enhance the team's environment. Interviews with the coach and athletes were conducted at three points, along with a survey on motivational climate perceptions. Results revealed improved perceptions of a caring, task-involving climate, contributing to positive athlete experiences despite external challenges such as academic pressures and time constraints. This research highlights the potential of motivational climate collaborations to positively influence the experiences of Native American collegiate athletes and underscores the importance of addressing contextual challenges to maintain these efforts, offering practical insights for coaches, sport psychology practitioners, and researchers.
144	The relationship between coaching leadership practices and athlete burnout among college athletes in China: the mediating role of psychological resilience and emotion regulation.	Chen, Xiaoying ; Yue, Yuan ; Xiang, Jun ; Zhang, Fang	BMC psychology	2026	10.1186/s40359-026-04891-x	S3	SPORTDiscus	As a key indicator in sports psychology reflecting athletes' complex physiological and psychological responses to challenges or threats, athlete burnout has become a focal issue in research on athlete-coach interactions and athletic performance.Objective To investigate the impact of coaches' leadership behaviors on athlete burnout among Chinese collegiate student-athletes and to validate the mediating roles of psychological resilience and emotion regulation in this relationship.Method Using convenience sampling combined with cluster sampling, 519 student athletes from multiple Chinese universities were selected as participants. The study employed the Coach Leadership Behavior Scale, the Burnout Scale, the Psychological Resilience Scale, and the emotion regulation Difficulty Scale for measurement. Data analysis utilized SPSS 21.0 and the Hayes Process macro for correlation analysis and mediation effect testing.Results (1) Significant gender differences emerged: Male athletes exhibited lower levels of psychological resilience and emotion regulation difficulties (i.e., stronger regulation abilities), with significantly lower sports burnout scores than female athletes ($P < 0.05$). No significant differences were found based on age or athletic level ($P > 0.05$). (2) Coach leadership behaviors showed a significant negative correlation with athletic burnout ($R = -0.543$, $P < 0.01$) and exerted a direct negative predictive effect on burnout ($\beta = -0.276$, $t = -7.726$, $P < 0.001$). Positive leadership behavior significantly enhanced psychological resilience ($\beta = 0.615$, $t = 12.956$, $P < 0.001$) and reduced emotion regulation difficulties ($\beta =-0.472$, $t=-7.929$, $P < 0.001$). Psychological resilience positively predicted emotion regulation levels ($\beta = -0.513$, $t = 10.696$, $P < 0.001$) and indirectly reduced burnout ($\beta = -0.116$, $t = -3.857$, $P < 0.001$); Reduced emotion regulation difficulties also significantly decreased burnout ($\beta = -0.160$, $t = -6.394$, $P < 0.001$); (3) Mediational analysis revealed that psychological resilience and emotion regulation difficulties jointly mediated the relationship between leadership behaviors and athlete burnout. Direct effects accounted for 58.35% of the total effect, while indirect effects accounted for 41.65%. Examining individual pathways: psychological resilience alone mediated 15.01%, emotion regulation difficulties alone mediated 15.86%, and their chained mediation effect accounted for 10.78%. This indicates that positive leadership behaviors alleviate sports burnout through dual pathways: enhancing psychological resilience and reducing emotion regulation

145	The coach as an architect of Danish high- performance sport environments	Henriksen, K; Dideriksen, S; Kuettel, A; Schlawe, A; Storm, LK	PSYCHOLOGY OF SPORT AND EXERCISE	2025	10.1016/j.psychsport.2025.102877	S1	Scopus+WoS	Elite athletes train and compete within high-performance sport environments that significantly influence their performance, development, and well-being. Within an enormous portfolio of tasks, a key role of a coach, therefore, is that of an 'architect' who designs, builds, and maintains successful high-performance environments. To understand this role in more depth, we conducted four case studies in which we interviewed one high-level coach in Danish Olympic and Paralympic sport, their manager, and two of their athletes. Each case was first analyzed using reflexive thematic analysis to identify key themes. Second, in a cross-case analysis focusing on how these four coaches develop and maintain environments conducive to top performance, we crafted two overarching themes. Non-negotiables refer to ways of leading that coaches described as so essential that they were never up for discussion, and included leading through values and clear structures, leading whole persons, and leading the whole staff. Delicate balances refer to dilemmas that coaches had to navigate when leading their HPSE, including balancing intensity with sustainable development, individual consideration with the team, a focus on the best with a focus on the rest, and athlete responsibility with coach guidance. The study illustrates that high-performance coaches play a critical role as architects of their athletes' environments and thus contributes to the understanding of the complexity of modern coaching, highlighting a need for ongoing professional development for coaches to enhance their leadership competencies in high-performance settings.
146	My biggest learning curve: an interpretive phenomenological analysis of coaches' experiences of working with athletes who experience a menstrual cycle	Jorgensen, H; Davenport, MH; Kavic, K; McHugh, TL	QUALITATIVE RESEARCH IN SPORT EXERCISE AND HEALTH	2025	10.1080/2159676X.2025.2495012	S1	WoS	The purpose of this interpretative phenomenological analysis was to describe and interpret coaches' experiences working with athletes who experience a menstrual cycle (MC). Participants included 15 high-performance coaches (11 women, 4 men) involved at the national or international level in a variety of winter and summer sports. Coaches participated in online, semi-structured one-on-one interviews. Data were analysed using an interpretative phenomenological analysis approach, and coaches' experiences are represented by four main themes: (a) 'Make them feel safe' - facilitating a culture of trust, empathy, and support; (b) 'It will be different' - recognising athletes' unique and personal experiences; (c) 'Find strategies that allow them to participate' - managing training and performance; and (d) 'Make it more normalised' - reducing stigma, barriers, and awkwardness. Our study advances MC-research in sport by highlighting the need to take athlete-centred approaches to understand athletes' unique MC experiences, as well as coaching-education and practices for progressing and supporting women athletes. In addition, we provide recommendations for future research and evidence-informed practices that can support athletes who experience a MC.
147	Impact of competitive anxiety and coach leadership style on track and field athlete performance: moderating role of coping strategies and emotional intelligence	Yue, Y; Zubing, X; Yasmin, F; Poulova, P	CURRENT PSYCHOLOGY	2025	10.1007/s12144-024-07125-8	S1+S2	SPORTDiscus+WoS	The current study aimed to investigate the effect of competitive anxiety and coach leadership style on track and field athlete performance through the moderating role of coping strategies and emotional intelligence. The purposive sampling technique was used, and data was collected from 297 athlete students from 15 public universities in China. The Partial Least Squares-structural equation modeling (PLS-SEM) on Smart PLS software version 4 was used to test the hypotheses. It was hypothesized that competitive anxiety and coach leadership style would significantly affect track and field athlete's performance. The result revealed that coach leadership style has a positive and significant impact on athlete's performance but competitive anxiety negatively affects athlete's performance. Furthermore, the moderators used athlete coping strategies and emotional intelligence. The athlete coping strategies have a negative effect on the relationship between competitive anxiety and athlete performance and a positive effect on coach leadership style and athlete performance. Lastly, athlete emotional intelligence also negatively affects the relationship between competitive anxiety and athlete performance. Therefore, research findings propose that coaches ought to establish a nurturing team environment and embrace a constructive mindset to assist athletes in managing the stress of competition caused by their high performance standards. When coaches demonstrate a positive attitude and confidence, athletes may internalize this perspective and utilize it as a coping mechanism. Besides that, athletes need to understand the worth of different coping mechanisms and how much their anxiety levels affect their performance. Moreover, professional sports psychologists and trainers should work together to guide athletes toward reaching their full potential.

148	Achieving international podium success with a positive sport experience	Willson, E; Kerr, G	INTERNATIONAL JOURNAL OF SPORT AND EXERCISE PSYCHOLOGY	2025	10.1080/1612197X.2025.2510252	S1	WoS	Positive coaching strategies (e.g., athlete - centred, autonomy - supportive, caring, transformational) have been highly regarded for their holistic approaches in creating supportive sport environments, enhancing motivation, and supporting athlete well - being. Despite calls for more positive approaches to athlete development, there is mounting evidence that high - performance coaches do not embrace these coaching styles, opting for controlling or emotionally abusive coaching styles. While various researchers have argued that a positive sports environment can lead to high - performance outcomes, coaches remain hesitant to change due to a perceived lack of solid evidence directly linking positive coaching methods to improved performance. This study addresses this gap by exploring international medal - winning athletes' and coaches' perspectives of achieving performance results in positive sport environments. Using a constructivist approach, semi - structured interviews were conducted with 24 Olympic and Paralympic athletes (n = 13) and coaches (n = 11) who had won a medal at an Olympic, Paralympic, Pan American Games, or World Championship. Results suggest that medals can be obtained in positive sport environments. Participants reported positive sport experiences also increased positive emotions, motivation, resiliency, feelings of being cared for and valued, personal development, sport - life balance, and staying in sport longer. All of these outcomes further contributed to increased performance results. These findings provide empirical evidence to support previous assertions that positive sport experiences can contribute to high - performance success.
149	Relationship between growth mindset and competitive motivation: a moderated parallel mediation model and feature importance analysis	Deng, JC; Wang, ZP; Chen, HW; Song, B	FRONTIERS IN PSYCHOLOGY	2025	10.3389/fpsyg.2025.1576649	S1	WoS	While growth mindset theory has been extensively studied in education, its influence on competitive motivation in sports contexts remains less understood. This study investigates how growth mindset (GM) affects competitive motivation (CM) among university athletes through stress response (SR) and basic psychological need satisfaction (BNS), with elite athlete status as a moderator. Analysis of data from 490 university athletes (250 elite, 240 non-elite) in Guangzhou revealed that GM positively relates to CM, partially mediated by reduced SR and increased BNS. Remarkably, elite athlete status demonstrated substantially stronger effects on these mediating pathways than GM itself, with elite athletes showing enhanced benefits from GM compared to non-elite peers. Feature importance analysis further identified dimension-specific predictors across different motivational aspects: autonomy most strongly predicted social recognition motivation, GM primarily influenced athletic ability improvement, while environmental factors and competition losses differentially affected entertainment and effort orientations. These findings expand GM applications in competitive sports and suggest that psychological interventions might yield stronger effects for elite athletes, highlighting the critical interplay between athletic development level and psychological factors in CM.
150	Mental well-being in young Taekwondo athletes: psychological-related parameters, associated factors and cluster analysis	Tozetto, WR; Kons, RL; Bussato, E; Felix, G; Ishibashi, M; Milistetd, M	INTERNATIONAL JOURNAL OF SPORT AND EXERCISE PSYCHOLOGY	2025	10.1080/1612197X.2025.2547362	S1	WoS	Taekwondo is a martial art that promotes physical, mental, and social development, making it a relevant practice for investigating factors influencing the mental well-being of athletes in Brazil. This study aimed to analyse the effect of sociodemographic factors, training characteristics, and sources of enjoyment on the mental well-being of Brazilian Taekwondo athletes. A cross-sectional observational design was employed, with data collected through standardised questionnaires completed remotely. The instruments gathered information on sociodemographics (age, gender, region), training (years of practice, weekly session duration, selection to the national team, and position), coach-athlete relationship (CART-Q), sources of enjoyment (SEYSQ), and sport well-being (S-MHC). The sample comprised 149 athletes (male = 77; female = 72) from all five geographic regions of Brazil, with a mean age of 15.4 +/- 1.58 years. Multiple linear regression revealed significant associations between CART-Q variables and sources of enjoyment with mental well-being, explaining 44.9% of its variance. Hierarchical cluster analysis grouped athletes based on the three well-being dimensions - emotional, social, and psychological - into three clusters: Cluster 1 (n = 15) with the lowest well-being levels, Cluster 2 (n = 58) with moderate levels, and Cluster 3 (n = 76) with the highest levels. ANOVA showed significant differences between clusters for all CART-Q domains and sources of enjoyment. It is concluded that harmonious coach-athlete relationships, combined with enjoyment sources such as effort exertion, peer affiliation, and self-referenced skills, are essential for enhancing the mental well-being of Taekwondo athletes.

151	Do coaches' and athletes' perceptions of relative energy deficiency in sport align? A narrative review	Delaney, D; McGuire, A; Warrington, G; Doyle, L	INTERNATIONAL JOURNAL OF SPORTS SCIENCE & COACHING	2025	10.1177/17479541251362409	S1	WoS	Relative energy deficiency in sport (REDs) is a syndrome that negatively impacts the health and performance of athletes, due to low energy availability (LEA), resulting in metabolic, cardiovascular, and endocrine function impairments, and an increased risk of injury. An increased emphasis on the coach-athlete relationship may facilitate the knowledge transfer of LEA's negative effects, mitigating the potential of an athlete developing REDs. Yet building on the International Olympic Committee's 2023 REDs statement of articles published between 2018-2022, to also include articles from 2023-2024, it is noted 38,424 participants were recruited to 246 REDs-related studies, with coaching staff only represented similar to 3% of the total cohort. This review aims to assess coaches' and athletes' perceptions of their respective, as well as each other's roles and experiences in the presence of LEA and REDs. A summary of athlete and coach first-person experiences of REDs-related issues is provided. However first-person REDs experiences are often that of the athlete, while the coach will more often 'experience' REDs from a second-person perspective, thereby highlighting the need for the development and provision of REDs education targeted to coaching staff which maybe sports-specific, taking into account topics such as appropriate language to use and how/when to discuss an athlete's body, health and/or performance. A better understanding of how athletes experience REDs-related issues may facilitate individual coaching staffs' evaluation of their role in preventing and/or addressing a potential REDs diagnosis.
152	Motivation, Sportspersonship, and Gender Equality in a Quidditch Sport Season: Analysis With Mixed Methodology	Méndez-Giménez, A; Collado-Martínez, M	JOURNAL OF TEACHING IN PHYSICAL EDUCATION	2025	10.1123/jtpe.2024-0250	S1	WoS	Purpose: This study examined the effect of a quidditch Sport Education season on motivational regulations, basic psychological needs, sportspersonship, and gender equality in physical education. Sixty students (M =12.62; SD = 0.60) from the first year of Secondary Education participated, divided into two groups (experimental group, N= 36 and control group, N= 24). Methods: A quasi-experimental design was carried out with pre- and posttest measurements, and a mixed methodology was used. Results: The intergroup multivariate analyses of variance, one at each measurement time, showed differences in intrinsic motivation, basic psychological needs, and commitment in favor of the experimental group in the posttest. Repeated-measures analyses of variance taking time as a within-subject factor and group and gender as between-subject factors revealed group significant effects over time on relatedness, social conventions, and rules and referees, as well as group interactions on autonomy and relatedness. From the qualitative analyses of 30 semistructured interviews, six categories emerged: satisfaction, perceived effort, basic psychological needs, equal and gender participation, classroom climate and sportspersonship, and facilitators and obstacles. Conclusion: Data triangulation supported the positive impact of the Sport Education Model on motivation, sportspersonship, and gender equality among students during the quidditch season.
153	The impact of low energy availability risk on pre-competition physiological function in Chinese female combat athletes	Liang, YH; Li, YX; Chen, Y; Meng, K; Zhou, FY; Pei, YR; Liu, Y; Qiu, JQ	JOURNAL OF THE INTERNATIONAL SOCIETY OF SPORTS NUTRITION	2025	10.1080/15502783.2025.2490170	S2	Scopus+WoS	Background: Low energy availability (LEA) can negatively impact athletes' physiological function and performance. This study aims to examine the prevalence of LEA in Chinese female combat athletes and monitor changes in physiological function and performance during the pre-competition period. Method: We assessed the incidence of low energy availability (LEA) and eating disorder (ED) risks in 84 female combat athletes (judo, freestyle wrestling, and sanda) from Beijing using the Low Energy Availability in Females Questionnaire (LEAF-Q) and the Eating Disorder Examination Questionnaire (EDE-Q). From this group, 11 judo athletes who were preparing for competition were selected and divided into a low energy availability (LEA) group and a non-LEA group based on their energy availability levels. Dietary intake, training energy expenditure, body composition, resting metabolic rate, blood markers, and special judo fitness tests were monitored at 4 weeks, 2 weeks, and 0 weeks before the competition. Results: Among the 84 athletes, 45.2% of athletes (n = 38) were at increased risk of LEA, and 21.4% of athletes (n = 18) were classified as high in eating disorder risk. There were no significant differences in LEA and ED risk between elite and recreational athletes. Among the 11 athletes preparing for competition, 6 athletes (45.5%) were in a state of LEA at the initial stage (4 weeks before the competition), and by the competition week, all 11 athletes exhibited LEA. Additionally, athletes in the LEA group experienced significant reductions in VO2 and resting metabolic rate at 0 week of the competition compared to 4 weeks prior ($p < 0.05$). Thyroid function indicators and IGF-1 levels of LEA group also significantly decreased ($p < 0.05$). After completing the four-week pre-competition weight loss, heart rate recovery during the special judo fitness test improved significantly in both the LEA and non-LEA groups ($p < 0.05$). Conclusion: The current study identified a risk of LEA among Chinese female combat sport athletes, with no significant difference in the prevalence of LEA between elite and recreational athletes. It is essential for Chinese coaches and sports medicine staff to implement LEA-related nutritional education across all performance levels. Moreover, preventive measures during training are recommended to mitigate the impact of LEA on physiological function during the pre-competition weight loss phase.

154	Differences in the relationship among grit, self-regulation, competition preparation, and sport confidence across performance levels: a multi-group analysis	Shen, YQ; Sim, YK; Bae, J; Bang, IJ; Seo, JP	SCIENTIFIC REPORTS	2025	10.1038/s41598-025-16448-w	S2	Scopus+WoS	In the literature on high-competition sports, some evidence has been suggested regarding the relationships between athletes' grit, self-regulation, competition preparation, and confidence. However, there has been a lack of integrated approaches to examine the relationships among these variables, and the performance level of athletes have not been considered. Therefore, this study (a) analyzed the structural relationships between grit, self-regulation, competition preparation, and confidence, and (b) investigated how these relationships vary according to athletes' performance levels. Participants were 270 student-athletes (209 males, 61 females) from various sports, all of whom were involved in highly competitive sports in South Korea. Athletes who had won at least one national-level competition were classified into the medal winner group (n = 112), while those without such experience were classified into the non-medal winner group (n = 158). Multi-group analysis showed that the structural relationships among grit, self-regulation, competition preparation, and confidence varied according to the athletes' performance levels. In the medal winner group, self-regulation fully mediated the effect of competition preparation on confidence, whereas in the non-medal winner group, the mediation was partial. Furthermore, the path estimates for self-regulation, competition preparation, and confidence were found to be greater in the medal winner group. This study highlights that coaches should understand athletes' performance levels and apply appropriate strategies accordingly.
155	Weight loss practices in Chinese national and international-level Sanda athletes	Zhong, YM; Tang, WW; Gee, T; Li, MF; Jiang, HD; Yin, MY; Xu, K; Lakicevic, N; Drid, P; Kirk, C; Langan-Evans, C; Artioli, GG; Reale, R; Weldon, A; Li, YM	JOURNAL OF THE INTERNATIONAL SOCIETY OF SPORTS NUTRITION	2025	10.1080/15502783.2025.2551216	S2	Scopus+WoS	BackgroundSanda is an unarmed combat sport (CS) comprises punching, kicking, throwing, wrestling, and defensive techniques that has increased in popularity. To ensure athletes compete against opponents of similar body size and weight for fairness, Sanda competition is classified according to weight classes. Previous studies revealed that CS athletes tend to intentionally lose weight to gain a competitive advantage over their opponents. However, little is known about weight loss (WL) practices in Sanda athletes. Therefore, the aim of this study is to investigate the WL practices of Chinese Sanda athletes.MethodsIn total, 169 (96 males, 73 females) national and international-level Sanda athletes completed the Rapid WL Questionnaire. Participants were categorized by sex (male, female), age (junior, senior), competition weight categories, and competitive levels (national, international).ResultsOver half (56%) of athletes purposefully implemented WL practices pre-competition. Their mean habitual WL was 7.6 +/- 3.7%, and mean highest WL was 10.8 +/- 4.2%. Participants usually allocated 15+ days pre-competition for WL (64%). Their rapid WL score (RWLS) was 30.4 +/- 6.4. There were no significant differences across Sanda athletes concerning habitual WL% (p = 0.386, p = 0.542, p = 0.207), highest WL% (p = 0.631, p = 0.232, p = 0.091), RWLS (p = 0.055, p = 0.395, p = 0.736) between junior and senior athletes, across weight categories, or between competitive levels. There were no significant differences in habitual WL% across WL time (p = 0.135). However, male athletes habitual WL% (p = 0.009), highest WL% (p = 0.002), number of WL in the last year between sexes (p = 0.048), and RWLS (p = 0.011) were significantly higher than female athletes. International athletes more frequently conducted WL per year than national athletes (p = 0.005). There were no significant differences in age groups (p = 0.447) and weight categories (p = 0.585). Participants usually allocated 15+ days before the competition for WL (64%), followed by 11-14 days (21%), 8-10 days (5%), 6-7 days (4%), 4-5 days (4%), and 1-3 days (1%). Training in plastic suits (87.4%), increasing exercise (85.2%), skipping meals (56.8%), limiting fluid intake (75.8%), gradually dieting (60.0%), and training in a heated room (54.7%) were the most frequently used WL practices for Sanda athletes. Sports coach (68.5%), strength and conditioning coaches/physical trainers (42.1%), other athletes (same sport) (39.0%), and parents (32.6%) were considered to have the highest impact
156	Using a Bayesian network to classify time to return to sport based on football injury epidemiological data	Yung, KKY; Wu, PPY; der Fünten, KAD; Hecksteden, A; Meyer, T	PLOS ONE	2025	10.1371/journal.pone.0314184	S2+S3	Scopus+WoS	The return-to-sport (RTS) process is multifaceted and complex, as multiple variables may interact and influence the time to RTS. These variables include intrinsic factors related the player, such as anthropometrics and playing position, or extrinsic factors, such as competitive pressure. Providing an individualised estimation of time to return to play is often challenging, and clinical decision support tools are not common in sports medicine. This study uses epidemiological data to demonstrate a Bayesian Network (BN). We applied a BN that integrated clinical, non-clinical factors, and expert knowledge to classify time day to RTS and injury severity (minimal, mild, moderate and severe) for individual players. Retrospective injury data of 3374 player seasons and 6143 time-loss injuries from seven seasons of the professional German football league (Bundesliga, 2014/2015 through 2020/2021) were collected from public databases and media resources. A total of twelve variables from three categories (player's characteristics and anthropometrics, match information and injury information) were included. The response variables were 1) days to RTS (1-3, 4-7, 8-14, 15-28, 29-60, > 60, and 2) injury severity (minimal, mild, moderate, and severe). The sensitivity of the model for days to RTS was 0.24-0.97, while for severity categories it was 0.73-1.00. The user's accuracy of the model for days to RTS was 0.52-0.83, while for severity categories, it was 0.67-1.00. The BN can help to integrate different data types to model the probability of an outcome, such as days to return to sport. In our study, the BN may support coaches and players in 1) predicting days to RTS given an injury, 2) team planning via assessment of scenarios based on players' characteristics and injury risk, and 3) understanding the relationships between injury risk factors and RTS. This study demonstrates the how a Bayesian network may aid clinical decision making for RTS.

157	Relative age effect, morphological status and physical performance of U-15 Croatian football players	Rada, A; Marasovic, J; Erceg, M; Krustrup, P; Randers, MB; Ardigò, LP	PLOS ONE	2025	10.1371/journal.pone.0327878	S2	Scopus+WoS	The relative age effect (RAE) refers to the unequal quartal-to-quartal birth distribution in competitive sports, leading to more athletes being born earlier in the selection period. This effect results in relatively older players, who are often physically more developed, being overrepresented in youth academies and professional levels, as they receive more attention and opportunities due to their advanced biological maturation and superior physical performance compared to their younger peers. This study involved 177 U-15 football players from Dalmatia, Croatia, averaging 14.1 +/- 0.9 yrs of age and 6.6 +/- 1.9 yrs of training experience. Anthropometric characteristics, motor-functional abilities, and specific abilities with the ball were measured over two days, using standardised equipment and procedures, and the data were analysed with descriptive statistics, one-way analysis of variance, and post hoc least significant difference tests to identify differences across quartiles. The study found statistically significant differences in the distribution and characteristics of players born in different quartiles of the year, with 63.3% born in the first two quartiles. RAE influenced young football players' birth distribution and physical characteristics, with a higher proportion born in the first quartile of the year (Q1) and showing superior morphological development and physical performance. Q1 players were significantly taller and heavier and performed better in explosiveness tests (medium effect sizes) compared to other quartiles. Furthermore, small to medium effect differences occurred in circumferences, 20-m, 30-m sprints and ball-kicking velocities in favour of Q1. As these abilities and characteristics are key to success in football, players born in the first quartile have an advantage in the selection process. This highlights the need for coaches to focus on potential talent rather than current performance despite the competitive pressure to win.
158	From adversity to adaptation: the struggle between resilience and athlete burnout in stressful situations	Cai, CY; Mei, ZY; Yang, Y; Luo, S	FRONTIERS IN PSYCHOLOGY	2025	10.3389/fpsyg.2025.1578198	S3	WoS	Athlete burnout is considered a negative psychological consequence of stressors in sports, with the potential to adversely affect both the physical and mental well-being of athletes, as well as their overall performance. Resilience serves as a protective factor against stressors, enabling athletes to effectively manage the unique challenges they encounter in the sports environment, ultimately helping protect them from burnout. This narrative review aimed to summarize the direct evidence regarding the relationship between resilience and athlete burnout. By examining existing theories and empirical evidence, the relationship between resilience and athlete burnout was explored and discussed in terms of individual and environmental factors, theoretical models, and neurobiological mechanisms to construct effective intervention programs to prevent and control the occurrence of athlete burnout. At present, resilience can positively influence athlete burnout through individual factors (perceived stress, coping strategies, and motivation) as well as environmental factors (social support, motivational climate, family cohesion, and coach-athlete relationship). The theoretical models mainly include the stress coping process model of athlete resilience and the systematic self-reflection model. These models elucidate the relationship between resilience and athlete burnout from the viewpoints of stress coping and self-reflection, respectively. The neurobiological mechanisms through which resilience influences athlete burnout are explored primarily through the hypothalamic-pituitary-adrenal (HPA) axis and the mesolimbic dopamine system. These mechanisms suggest that resilience has an effect on athlete burnout primarily through stress hormone levels and brain region activity related to reward and motivation. However, the pathways and mechanisms through which resilience influences athlete burnout require further in-depth investigation. Future research should combine the longitudinal perspective to track the dynamic impact relationship between resilience and athlete burnout and focus on examining the relevant theoretical framework from a multidisciplinary research perspective to provide a theoretical basis for coaches and sports managers to formulate scientific training programs. This will help enhance athlete resilience, effectively prevent and mitigate the risk of burnout, and promote the development of athletes' physical and mental well-being.
159	The relationship between self-oriented perfectionism and athlete burnout: a longitudinal study	Fu, YT; Li, XY; Sun, JJ; Li, CX; Peng, Y; Hong, FX; Pan, JH	FRONTIERS IN PSYCHOLOGY	2025	10.3389/fpsyg.2025.1656816	S3	WoS	Background: Athlete burnout is a critical psychological concern, and perfectionism-characterized by excessively high standards and self-critical tendencies-may play a role in its onset. However, prior findings on the link between self-oriented perfectionism (SOP) and burnout remain inconsistent, and the mechanisms underlying this relationship are not fully understood. Methods: This longitudinal study employed a Random-Intercept Cross-Lagged Panel Model (RI-CLPM) to examine reciprocal associations between SOP and athlete burnout, as well as the mediating role of loneliness. A total of 422 athletes (M-age = 19.74, SD = 1.77) completed three waves of assessments between June 2024 and June 2025. Results: SOP significantly predicted subsequent athlete burnout (SOP_T1 -> AB_T2: beta = 0.355, p < 0.001; SOP_T2 -> AB_T3: beta = 0.374, p < 0.001). Loneliness emerged as a significant mediator: higher SOP predicted increased loneliness (beta = 0.376, p < 0.001), which in turn was associated with greater burnout (beta = 0.265, p < 0.01). The indirect effect was significant (beta = 0.099, 95% CI [0.033, 0.209]). Notably, no evidence supported a reverse pathway from burnout to subsequent SOP. Discussion: SOP is a longitudinal risk factor for athlete burnout, partly through its contribution to heightened loneliness. Preventive interventions that enhance social and coach support, alongside resilience-building strategies such as mindfulness, may help reduce vulnerability to burnout in athletes.

160	A systematic review of the factors associated with athlete burnout in team sports	Woods, S; Dunne, S; Gallagher, P; McNicholl, A	INTERNATIONAL REVIEW OF SPORT AND EXERCISE PSYCHOLOGY	2025	10.1080/1750984X.2022.2148225	S3	WoS	Athlete burnout is a psychological syndrome with substantial negative consequences, including depression and sport dropout. It has been linked to a range of factors, some of which may vary across different sport-types. This review is the first to synthesise variables examined in relation to burnout in team-sports specifically. An online search of seven relevant databases yielded 59 papers examining 123 burnout correlates. Eligible papers were peer-reviewed, quantitative empirical studies, which assessed burnout using the Athlete Burnout Questionnaire. Results are reported in-line with PRISMA guidelines. Weighted meta-analysis (WMA) assessed the strength of the relationship between burnout dimensions and 18 variables examined across >= 3 samples. A narrative synthesis of the remaining cross-sectional, longitudinal and mediating/moderating relationships provides a comprehensive overview of the literature. Burnout displayed a negative relationship with autonomy, competence, relatedness, self-determined motivation, positive affect, autonomy supportive coach, harmonious passion, self-oriented perfectionism and social support, and a positive relationship with amotivation, negative affect, obsessive passion, socially-prescribed perfectionism, ego-involving climate, playing experience and controlling coach style in the WMA. Some variability in relationships was identified across the dimensions of burnout. The results highlight the key factors associated with the dimension of burnout in team sports, which may inform targeted intervention strategies.
161	Coach-athlete attachment and athlete burnout: testing longitudinal mediation via cognitive reappraisal and expressive suppression	Xin, ZY; Li, TE; Li, QL; Chen, YA; Wang, MH	INTERNATIONAL JOURNAL OF SPORT AND EXERCISE PSYCHOLOGY	2025	10.1080/1612197X.2024.2389207	S3	SPORTDiscus+WoS	Athlete burnout is a psychological syndrome that leads to emotional and physical exhaustion, a reduced sense of achievement, and sport devaluation among athletes. Drawing on attachment theory and the emotion regulation model of attachment, this study aimed to explore the potential mechanism of the relationship between coach-athlete attachment and athlete burnout. We employed a two-wave longitudinal design method to track 352 adult athletes in China, from which 258 completed the entire data collection (Mage = 22.2; SD = 3.2 years; 68.2% male). Results of the cross-lagged panel model for a half-longitudinal design showed that secure attachment was negatively linked to athlete burnout through cognitive reappraisal and expressive suppression, and avoidant attachment was positively linked to athlete burnout through expressive suppression. Our study applies attachment theory to the literature on athlete burnout and emphasises the importance of emotion regulation. The findings encourage coaches and sports-related therapists to develop appropriate interventions targeting attachment style and cognitive reappraisal training to reduce the risk of athlete burnout.
162	Longitudinal associations between coach-athlete relationship quality and individual injury rate among young handball players: A variable and person-centered approach in intensive training centers	Roux, V; Trouilloud, D	INTERNATIONAL JOURNAL OF SPORTS SCIENCE & COACHING	2025	10.1177/17479541251334632	S3	WoS	This study explores the potential associations between the evolution of coach-athlete relationship quality (in terms of closeness, commitment, and complementarity) and the athletes' individual injury rate over a sport season. 203 young French handball players (Mage = 15.38, 47.3% women) involved in intensive training centers completed a French version of the Coach-Athlete Relationship Questionnaire at the beginning, middle, and end of the season. The athlete follow-up allowed their individual injury rate over the season to be captured. Both variable and person-centered approaches were used to explore the temporal associations between these variables. A linear growth model and a multiple regression analysis indicated that the evolution of coach-athlete relationship quality was negatively related to athletes' individual injury rate over the season. In addition, latent class growth analyses and ANOVA revealed inter-individual differences in the evolution of this association, indicating that athletes who perceived a deterioration in the relationship with their coach were more often injured during the season. This study is the first to highlight a temporal association between coach-athlete relationship quality and athletes' individual injury rate over time, and thus provides stimulating perspectives for future research.
163	Canadian University Sport Coaches' Experiences Participating in a Social Learning Space on Topics Related to and Impacting Coach Mental Health	Henderson, S; Bloom, GA; Durand-Bush, N; Kenttä, G	SPORT EXERCISE AND PERFORMANCE PSYCHOLOGY	2025	10.1037/spy0000394	S3	WoS	Although high-performance coaching can be rewarding, it can also cause considerable performance, organizational, and personal stress, which over time, can diminish coaches' mental health. There is a growing recognition that collective and community-based interventions that extend beyond the individual level are needed to support mental health, such as a social learning space. Therefore, the purpose of this study was to understand university coaches' experiences participating in a season-long social learning space on topics related to and impacting coach mental health. Specifically, 10 Canadian University coaches engaged in six monthly sessions throughout one competitive season on topics related to mental health. Data collection consisted of pre- and postseason individual, semistructured interviews. Data were analyzed abductively using a reflexive thematic analysis. Results revealed that coaches reported positive experiences from participating in the social learning space, including the sense of community it created and the quality of the knowledge provided by the invited speakers. The environment encouraged sharing and provided insights into mental health, leadership, and the coach-athlete relationship. Coaches also acquired new resources, improved personal well-being, and enhanced coaching effectiveness. Overall, this initiative empowered coaches to become advocates for mental health-both for themselves and for their athletes and networks.

164	The relation between perceived need-supportive coaching behaviours, winning games, and team resilience: a longitudinal examination among basketball and soccer teams	Van Puyenbroeck, S; Hermans, F; Boen, F; Fransen, K; Vande Broek, G	INTERNATIONAL JOURNAL OF SPORT AND EXERCISE PSYCHOLOGY	2025	10.1080/1612197X.2024.2366421	S3	WoS		In team resilience literature, team performance is consistently regarded as a primary outcome of resilient teams. However, empirical evidence linking team resilience to objective performance indicators such as effectively winning a game remains scarce. Drawing upon team resilience literature and Self-Determination Theory, this study firstly investigated whether team resilience predict the odds of winning. In addition, as sports teams inevitably experience common setbacks, this study examined whether athletes' perceived need-supportive and controlling coaching behaviours predict team resilience over time. This research therefore contributes to coaching literature by providing insights into how coaches could arm teams against adversities and increase their resilience. We conducted a three-wave longitudinal study with a sample of 397 basketball and soccer players, belonging to 39 distinct teams. They completed questionnaires at the beginning, middle and end of the season. Multilevel binomial logistic regressions indicated that team resilience significantly increased the odds of winning on top of athletes' perceived need-supportive and psychologically controlling coaching behaviours, which emerged as non-significant predictors. Random intercept cross-lagged panel models further revealed that perceived need-supportive coaching behaviours predicted higher levels of team resilience over time, while perceived psychologically controlling behaviours was associated with lower team resilience in the following waves. We conclude that team resilience is a clear predictor of actually winning a game. Although coaches' need-supportive or controlling behaviours do not directly predict their teams' odds of winning, it might enable them to create a resilient team capable of handling collective adversities.
165	The threshold moderating effect of self-efficacy: a nonlinear transformation mechanism of work passion on job satisfaction among sports practitioners	Wang, KY; Tan, M	FRONTIERS IN PSYCHOLOGY	2025	10.3389/fpsyg.2025.1667520	S3	WoS		Despite the global sports industry's growth, practitioners face a systemic satisfaction crisis inadequately explained by traditional linear models (e.g., JD-R model) or existing nonlinear frameworks (e.g., COR theory's resource caravans), which fail to capture sport-specific dynamics and threshold effects of key cognitive resources. Focusing on China's unique high-pressure context (e.g., policy shifts like the Double Reduction), this study proposes and validates the Passion-Efficacy Synergistic Gain (PESG) framework, positioning self-efficacy (SE) as a critical nonlinear cognitive transformer moderating the passion-satisfaction link. Using multi-wave longitudinal data from 1,384 Chinese sports practitioners (coaches, athletes, technical staff), we employed threshold regression and 3D response surface analysis (RSA). Work passion was measured as a composite score representing total affective investment (mean of Harmonious Passion, HP, and Obsessive Passion, OP, subscale totals), capturing overall motivational resource magnitude.
166	The motivational climate perceived by young soccer players regarding their coaches, parents, and peers on sport optimal functioning: a cluster analysis	Martínez-González, N; Atienza-González, FL; González-García, L; Balaguer, I	FRONTIERS IN PSYCHOLOGY	2025	10.3389/fpsyg.2025.1564391	S3	WoS		The aim of this study was (a) to describe the perceived motivational climate profiles created by coaches, parents, and peers of young football players, and (b) to analyse the implications of these profiles on goal orientations, motivation, psychological needs, and indicators of well- and ill-being. The participants were 876 football players (M = 13.57; SD = 1.17) who completed a multi-section questionnaire at the beginning of the sports season. Cluster analyses identified four profiles (empowering, disempowering, high mixed, low mixed) based on youth perceptions of empowering and disempowering climates created by coaches, parents, and peers. Multivariate analyses, controlling for gender and age, revealed that those young players categorized in the most adaptive profiles (high empowering climates and low disempowering climates) demonstrated optimal functioning, including task orientation, higher autonomous motivation, need satisfaction, and vitality. Conversely, those in the most maladaptive profile (low empowering climates and high disempowering climates) experienced the worst consequences (high levels of ego orientation, controlled motivation, no motivation, need frustration, physical and emotional exhaustion). Regarding mixed profiles, although the analyses reveal that combining empowering and disempowering behaviors limits the benefits of empowering climates, and low involvement hinders athletes' development, future explorations are necessary to better interpret these profiles. As a whole, the results highlight the importance of considering the combined roles of coaches, parents, and peers, and suggest that interventions aimed at fostering empowering behaviors and reducing disempowering ones across these social agents may help support youth athletes' optimal functioning.

167	Resilience and Well-Being Over the Course of an National Collegiate Athletic Association Division I Gymnastics Season	Williams, SM; Baez, SE; Derrico, D; Robinson, H; Coyne, K; Settle, H; Jochimsen, KN	JOURNAL OF SPORT REHABILITATION	2025	10.1123/jsr.2023-0379	S3	WoS	Context: Awareness regarding the importance of mental health in athletes has been increasing in recent years. Mental health has been investigated in relation to injury recovery and performance; however, less evidence has examined changes in mental health throughout a competitive season. Objectives: This study aimed to: (1) evaluate the psychological factors of resilience and well-being over a National Collegiate Athletic Association Division I gymnastics season and examine changes over 4 time points: preseason, start of the competitive season, midseason, and postseason and (2) examine the relationships between these psychological measures at these 4 time points. We hypothesized that resilience and well-being would remain stable, and higher resilience would be related to a greater sense of well-being at all time points. Design: This study utilized a retrospective chart review. Setting: This study is conducted in a college setting. Patients or Other Participants: Study participants were 17 Division I female gymnasts. Methods: A retrospective chart review was conducted to evaluate the Brief Resilience Scale and 5-item World Health Organization Well-Being Index, which gymnasts had completed biweekly from preseason to postseason. Descriptive statistics were reported, a 1-way repeated measures analysis of variance was used to examine changes across 4 time points, and Pearson product-moment correlations were used to examine the relationships between Brief Resilience Scale and 5-item World Health Organization Well-Being Index scores. Results: Brief Resilience Scale and 5-item World Health Organization Well-Being Index scores did not change throughout the season (P = .62, .52). No relationship between the variables was identified at the start of preseason or midseason (P > .17). However, at the start of the competitive season and postseason, resilience was positively correlated with well-being (r = .65, P = .006; r = .82, P = .006). Conclusions: Higher resilience and well-being are related in Division I female gymnasts but do not fluctuate throughout the season. Sports medicine staff and coaches should be aware that there is room to improve resilience and well-being, making additional supports warranted, including coping skills and resilience training interventions.
168	Smartphone use and mental health - findings from a 30-day longitudinal study among young talented volleyball players	Hilpisch, C; Zentgraf, K; Mutz, M	INTERNATIONAL JOURNAL OF SPORT AND EXERCISE PSYCHOLOGY	2025	10.1080/1612197X.2025.2563317	S3	WoS	This longitudinal study examines the impact of smartphone use on mental health-related outcomes in a sample of N = 47 of Germany's most talented young male volleyball players. Accounting for both inter- and intra-individual variation in smartphone use, we investigate the coexistence and impact of three temporally distinct effects: habitual, short-term, and late-night smartphone use. Results highlight the consequences of habitual effects, with higher levels of regular smartphone use being associated with increased symptoms of anxiety, depression, and sleep complaints. In addition, we find negative non-linear relationships between habitual smartphone use and athletes' emotional well-being and concentration during training. In terms of short-term effects, athletes report more mind-wandering during training sessions following days of higher smartphone use, while no evidence is found for the hypothesised late-night effect. We conclude that habitual smartphone use exceeding approximately 4 hours a day is problematic for young athletes' performance and mental health. Athletes, coaches, and sport psychologists should be made aware of the importance of managing screen time.
169	Fostering self-efficacy in Chinese university athletes: the mediating roles of psychological resilience and emotional regulation and the moderating role of autonomy-supportive coaching	Zhang, X; Khan, SU	FRONTIERS IN PSYCHOLOGY	2025	10.3389/fpsyg.2025.1664339	S3	SPORTDiscus+WoS	This study explores how mental toughness contributes to the psychological sustainability of athletes by enhancing emotional regulation and psychological resilience, ultimately fostering self-efficacy, particularly in environments supported by autonomy-supportive coaching. The study examines mental toughness (MT), a dispositional resource for goal-focused persistence under pressure, and its effects on performance self-efficacy (PSE) through the mediating roles of emotional regulation (ER) and psychological resilience (PR), with autonomy-supportive coaching (ASC) as a contextual moderator. University athletes in China face intense dual academic-athletic demands within a collectivist culture that may suppress autonomy and undermine self-efficacy, yet little research addresses how psychological and coaching factors jointly shape their confidence. Using purposive sampling, we recruited 581 active university athletes across China who met the eligibility criteria: (1) currently enrolled as a university student, and (2) actively participating in organized sports for at least one year. Participants completed validated scales measuring the target constructs. We analyzed the data through structural equation modeling (SEM) in Smart PLS. Results reveal that MT positively affects ER and PR, which in turn significantly enhance PSE. Both ER and PR mediate the relationship between MT and PSE, while ASC strengthens the positive effects of MT on ER and PR. Theoretically, the study highlights the role of internal psychological resources and supportive environments in sustaining athlete development over time, offering a framework that bridges sport psychology with broader concepts of personal and developmental sustainability. The study provides practical guidance for coaches aiming to foster mental toughness and psychological skills to improve athletes' confidence and psychological resilience.

170	Structural modelling of student volleyball athletes' intimacy, social adjustment, perceived stress, and learning-related anxiety: mediating role of psychological safety	Li, YS	BMC PSYCHOLOGY	2025	10.1186/s40359-024-02299-z	S3	Scopus+WoS	BackgroundPsychological safety is crucial for student-athletes, impacting their stress levels and academic anxiety. Understanding how interpersonal factors like intimacy and social adjustment impact psychological safety can guide interventions to enhance student-athlete well-being.ObjectivesThis study investigates the relationships between intimacy, social adjustment, psychological safety, perceived stress, and learning-related anxiety among student-athletes. It tests explicitly whether psychological safety mediates the effects of intimacy and social adjustment on perceived stress and learning-related anxiety.MethodologyA cross-sectional design was employed, involving 300 student-athletes from various universities in Wuhan, China. Participants completed measures of intimacy (RCI), social adjustment (SAS-SR), perceived stress (PSS), learning-related anxiety (LAS), and psychological safety (PSS). Structural Equation Modeling (SEM) was used to analyze direct and indirect relationships among the variables.Results/DiscussionThe results revealed that intimacy and social adjustment positively impact psychological safety. In turn, psychological safety negatively affects perceived stress and learning-related anxiety. The mediation analysis showed that psychological safety significantly mediates the relationships between both intimacy and social adjustment with perceived stress and learning-related anxiety. Specifically, the study found that higher intimacy and social adjustment levels increased psychological safety, subsequently reducing perceived stress and anxiety levels. These findings underscore the importance of fostering a psychologically safe environment to mitigate stress and anxiety among student-athletes. The study's implications suggest that coaches and educators should build supportive relationships and enhance social integration to improve psychological safety and overall athlete well-being.ConclusionThis study highlights the critical role of psychological safety in student athletes' mental health. By promoting intimacy and social adjustment, institutions can enhance psychological safety, reduce stress and anxiety, and improve athletes' academic and personal experiences. Future research should explore longitudinal effects and potential moderators to understand these relationships further.
171	Ethnographic research on coaching behaviour to enhance athlete skill learning: A scoping review	Taylor, S; Renshaw, I; Pinder, R; Polman, R; Russell, S; Gorman, AD	INTERNATIONAL JOURNAL OF SPORTS SCIENCE & COACHING	2025	10.1177/17479541251350035	S3	WoS	Numerous calls have been made throughout coaching science literature for consideration of different methodological and theoretical approaches to underpin research design. Recommendations include the need for more 'naturalistic' 1 and 'longitudinal' 2, 3 approaches. One such approach is ethnography. The purpose of this scoping review was to provide an overview of peer-reviewed studies that used ethnography to investigate coaching behaviours that aim to enhance skill learning. The Preferred Reporting Items for Systematic Reviews and Meta-analysis extension for scoping reviews (PRISMA-ScR) 4 was used to guide this review. Electronic databases including APA PsycINFO, SPORTDiscus, SCOPUS, and PubMed were searched to identify eligible studies. From 1661 results, 9 studies were determined to meet the eligibility criteria. Findings indicated that the use of ethnography is increasing, however, the low number of studies suggest its uptake remains limited in coaching science. The embedded nature of ethnography creates an opportunity for researchers to gain in-depth and nuanced insights on coaching behaviours in situ. The included studies suggest that different methodological and theoretical approaches underpinning ethnography can help enhance our understanding of the coaching process. Specifically, the use of ethnography allows us to see how behaviours inform coaching practice, influence task design and oppurtunities for learning, and the responding behaviour and actions exhibited by athletes.
172	The Effect of an Educational Session to Improve Knowledge About the Menstrual Cycle and Hormonal Contraceptives in Junior Endurance Athletes and Their Coaches : An Exploratory Investigation	Solli, GS; Topranin, VD; Svantorp-Tveiten, KME; Noordhof, DA	INTERNATIONAL JOURNAL OF SPORTS PHYSIOLOGY AND PERFORMANCE	2025	10.1123/ijspp.2025-0068	S3	WoS	Purpose: To investigate the effects of a tailored educational session about the menstrual cycle (MC) and hormonal contraceptives (HCs) on perceived knowledge, knowledge satisfaction, communication, and the coach-athlete relationship among junior endurance athletes and their coaches. Methods: Junior female cross-country skiers and biathletes (n = 36) and their coaches (n =12) completed 2 questionnaires within an 8-week interval (PRE-POST). After 2 weeks, the intervention group (20 athletes and 6 coaches) participated in a tailored educational session designed to enhance their knowledge about the MC and HCs. The control group (16 athletes and 6 coaches) received no education on this topic. Results: Athletes and coaches in the intervention group experienced significant PRE-POST increases in perceived knowledge (estimate = 1.28, P = .002) and satisfaction (estimate =1.20, P < .001) with their knowledge of the MC and HCs compared to the control group. Coaches had higher perceived knowledge (estimate = 1.45, P = .003) and satisfaction (estimate = 0.83, P = .041) with their knowledge of the MC than athletes. The intervention improved the degree of communication with peers (estimate =1.02, P = .013) but not with the other role (ie, athletes' communication with coaches and vice versa; estimate = 0.12, P = .833). In addition, it improved perceived ease of communication (estimate = 0.93, P = .003). The intervention did not seem to affect the coach-athlete relationship (P> .005). Conclusion: The educational session resulted in promising effects on perceived knowledge and satisfaction with athletes' and coaches' knowledge of the MC/HCs, while also facilitating communication among athletes. Future studies should focus on implementing and evaluating more longitudinal interventions with larger sample sizes.

173	Proactive personality and positional competition: exploring the impact on soccer players' performance and gender differences	Çar, B; Kurtoglu, A; Eken, Ö; Sufaru, C; Ciaccioni, S; Baskaya, G; Panaet, EA; Dragomir, L; Alexe, CI; Bulzan, CO	BMC SPORTS SCIENCE MEDICINE AND REHABILITATION	2025	10.1186/s13102-025-01086-6	S3	WoS	Aim The study aims to explore the relationship between proactive personality and positional competition in soccer players, considering factors like gender, age, position, and years of active participation in the sport. Method Quantitative data collection methods were employed, including the Proactive Personality Scale and Positional Competition in Sports Scale, along with a custom personal information form. The study involved 242 soccer players (average age: 20.60 years, 39% female) from semi-amateur soccer clubs in Ankara province, surveyed during the 2022-2023 season. Results The study found no significant differences in proactive personality and positional competition among soccer players based on their age and position (p > .05). However, gender differences were significant, with male players scoring higher than females in receiving and providing teammate support, communication, and overall positional competition in sports. Conclusion Proactive personality can be a determinant of positional competition in sports. Moreover, these findings may provide important contributions to the development of talent identification, athlete development programs and coaching strategies that aim to increase competitiveness and success in positional contexts. In addition, in the present study, it can be concluded that following athletes over time is an important phenomenon for proactive personality and athletic development.
174	Implementing pressure on athletes during training: a scoping review	Bouthiller, E; Lautenbach, F	INTERNATIONAL REVIEW OF SPORT AND EXERCISE PSYCHOLOGY	2025	10.1080/1750984X.2025.2578781	S3	WoS	The strategic use of pressure in sport training has gained increasing attention. This scoping review aimed to synthesize research on pressure implementation in sport by examining: (a) theoretical frameworks underpinning pressure-based practices, (b) strategies used to enhance pressure in training, (c) associated outcomes and measurement methods, and (d) key knowledge gaps. A total of 22 peer-reviewed studies were identified from PsycINFO, PsycARTICLES, CINAHL Complete, and SPORTDiscus. From nine theoretical frameworks, two perspectives emerged: systematic exposure to pressure as a means to develop resilience, adaptive coping, and effective stress responses (66.7%); and the simulation of competition-like conditions to enhance skill acquisition and transfer (33.3%). Study populations included athletes (84.5%), coaches (13.4%), and sport psychologists (2.1%). Methodologically, 40.9% of studies employed qualitative, 31.8% quantitative, and 27.3% mixed-methods designs. Across studies, 92 distinct pressure-inducing strategies were identified. Interviews (77.8%) were the most common qualitative method, while interventions (57.1%) dominated quantitative designs. Overall, findings support the benefits of structured pressure training, though potential drawbacks, such as negative impacts on coach-athlete relationship, were noted. The review underscores the need for an integrative theoretical framework, multi-method research designs, longitudinal investigations, and the development of a validated instrument to assess the type and frequency of pressure-based strategies.
175	The Relationship Between Preseason Upper Extremity Function, Pain, and Training and Normalized Division III Collegiate Swimming Performance	Tate, AR; Woznicki, L; Strouse, G; Wisseman, D; Thomas, S	JOURNAL OF ATHLETIC TRAINING	2025	10.4085/1062-6050-0080.24	S3	WoS	Context: Shoulder injuries comprise the largest proportion of swimming injuries, and a large percentage of swimmers participate with pain. Therefore, it is assumed that shoulder pain decreases performance, but researchers have not compared collegiate swimmers' performance with and without pain. Objectives: (1) To determine if individual swimmers' shoulder pain and function are associated with a change in normalized swimming performance over a season, (2) to determine if differences in normalized swimming performance exist among 3 collegiate teams, and (3) to qualitatively describe and compare each team's training regimes. Design: Cross-sectional study. Setting: Swimmers completed preseason (T1) and post- season (T2) surveys including pain ratings and shoulder function using the Kerlan-Jobe Orthopaedic Clinic (KJOC) shoulder and elbow questionnaire. Swimming times were obtained from published meet results. Coaches reported training programs through interviews and tracking logs. Patients or Other Participants: Fifty-two National Collegiate Athletic Association Division III swimmers from 3 teams. Main Outcome Measure(s): Stepwise linear regression was used to determine if pain and function related to performance. Team demographics and normalized swimming performance (reduction in time per lap from season beginning to end) were compared with analyses of variance with post hoc tests. Results: Initial KJOC scores, but not pain, related to individual swimming performance. Differences in team performance were found (P = .006), with Team 3 having the greatest reduction in time (1.01 s/lap), a lower percentage of females, a more experienced coach, and a periodization schedule with large increases and decreases in yardage. A main effect (P = .043) was found for baseline demographics, with Team 3's swimmers being taller and having longer competitive experience. Conclusions: The initial KJOC score predicting swimming performance improvement demonstrates the need for athletic trainers to prioritize enhancement of preseason function. Endurance training-induced hypoalgesia and motivation may explain the lack of effect of shoulder pain on performance. Further research is needed to elucidate optimal periodization and dry-land training.

176	A model of relationships of teammate relations, body image, and eating behaviors among athletes	Runquist, ER III; Cox, M; Meyer, A; Rodgers, RF	BODY IMAGE	2025	10.1016/j.bodyim.2024.101818	S3	WoS	Recent studies have identified athletes to be at elevated risk for developing disordered eating habits. However, few studies have investigated the association between teammate interactions and athletes' relationships with food and their body. To address this gap, the current study examined a model in which teammate appearance pressures were associated with disordered eating behaviors and body image concerns among varsity and club athletes who competed in a variety of sports at a US university. A sample of 156 university athletes, mean age = 20.7 years (SD = 1.2) (19.2 % varsity, 80.8 % club, 63.5 % female-identifying), completed self-report measures that assessed teammate pressures, depth of teammate relationships, internalization of athletic body ideals, teammate body comparisons, weight and shape concerns, and muscularity oriented disordered eating behaviors. Path analysis was used to test the hypothetical model, which after modification revealed a good fit to the data. The findings suggest that social frameworks are useful for conceptualizing relationships between teammate pressures and associated body image concerns and disordered eating behaviors. Further, the findings provide insight into how team environments may play an important role in shaping athletes' body image and relationship with food and support the development of prevention interventions that can be implemented at team levels.
177	Evaluating external load responses to cumulative playing time and position in the European Handball Federation Women's Euro 2022 through an IoT and Big Data architecture approach	Karcher, C; Font, R; Marcos-Jorquera, D; Gilart-Iglesias, V; Manchado, C	BIOLOGY OF SPORT	2025	10.5114/biolsport.2025.144409	S3	WoS	The quantification of physical demands placed upon handball players, segmented by their specific roles and duration of play, is crucial for sustaining high performance and minimizing the risk of injury. Leveraging advanced inertial measurement units, this investigation captured and analyzed the external load data of athletes participating in the EHF Women's EURO 2022. The aim of this study was to provide coaching staff with information on fatigue development during periods of high match density. The study evaluated the effects of playing position and cumulative playing time on external load metrics, using linear mixed models that treated individual players as random effects. The study employed a cutting-edge computational framework integrating sensor network technologies, Local Positioning Systems (LPS), and Big Data Analytics within a descriptive analytics methodology. From over half a billion raw records, we distilled 1,013 data entries from 47 matches for analysis. The findings reveal that the wings demonstrated the highest levels of total and high-speed running distances, though they sustained lower PlayerLoad relative to backs. Interestingly, cumulative playing time did not markedly alter load profiles, which may be attributed to strategic substitution decisions by coaches and the players' own pacing strategies. Notable discrepancies within positional demands were observed over time, such as centers displaying increased distance coverage within the 2-3 hour play interval. This study underscores the efficacy of strategic load management and tailored pacing in sustaining player performance throughout high-stakes tournaments. It elucidates the relationship between managerial tactics and player-specific characteristics in the context of external load distribution.
178	An Examination of the Relationship Between Single Leg Jump and Balance Performance in Female Collegiate Basketball Players	Zahn, D; Willardson, JM	JOURNAL OF STRENGTH AND CONDITIONING RESEARCH	2025	10.1519/JSC.0000000000005112	S3	WoS	Zahn, D and Willardson, JM. An examination of the relationship between single leg jump and balance performance in female collegiate basketball players. J Strength Cond Res 39(6): 705-710, 2025-The purpose of this study was to examine relationships between different types of single leg jumps and components of the Y-Balance Test (YBT) in a convenience sample of female collegiate basketball players. Data collection involved assessment of players in the single leg vertical jump, single leg broad jump, and the YBT. These 3 tests were performed in random order and with the order of legs tested also randomized. Independent t-tests were conducted to assess symmetry in balance and jump performance between legs and Pearson correlations to assess relationships between tests (p <= 0.05). For all tests, there were no significant differences between legs, but multiple significant correlations were found between tests. Key findings included a significant (p = 0.046) and positive relationship (r = 0.584, r(2) = 0.341) between the left leg vertical jump and YBT right leg posteromedial percent reach, while supported on the left leg, and reciprocally, a significant (p = 0.020) and positive relationship (r = 0.659, r(2) = 0.434) between the right leg vertical jump and YBT left leg posteromedial percent reach, while supported on the right leg. Other key findings included significant and positive correlations between the right leg broad jump and right leg vertical jump (p = 0.034, r = 0.612, r(2) = 0.375) and between the left leg broad jump and left leg vertical jump (p = 0.008, r = 0.719, r(2) = 0.517). When considering that none of the players suffered a significant time-loss injury during the course of the season, the above results may suggest a benefit in seeking for symmetry in single leg vertical and broad jump and YBT performance as part of the strength and conditioning process. The mean values for the single leg vertical jump (left, 30.29 cm; right, 31.16 cm) and single leg broad jump (left, 168.60 cm; right, 163.85 cm) and the YBT percent reach values for the anterior (left, 67.17%; right 68.86%), posteromedial (left, 130.46%; right 128.23%), and posterolateral (left, 124.49%; right 127.35%) directions from the present study can be used by strength and conditioning coaches for comparative purposes in healthy samples of female collegiate basketball players.

179	The story just brings the science to life: exploring the use of stories to translate knowledge about self-regulatory processes during goal striving in running	Jackman, PC; Whitehead, AE; Brick, NE; Wadey, R	INTERNATIONAL JOURNAL OF SPORT AND EXERCISE PSYCHOLOGY	2025	10.1080/1612197X.2024.2372404	S3	WoS	Storied representations of research have been recommended and identified as an effective pedagogical tool to facilitate knowledge transfer to end users. However, storied forms of representation have not yet been used to communicate knowledge of self-regulation during goal striving in running. Therefore, building upon a previous programme of research represented via a traditional realist tale (Jackman et al., 2024), the study aims were twofold: (1) to construct stories that communicate runners' lived experiences of self-regulation during goal striving in distance-running; and (2) to share these stories with relevant knowledge users to explore what they do on and for them in the short-term (1-2 weeks) and long-term (six months). Stories were constructed and represented through vignettes. To accommodate diverse views and appreciate different knowledge bases, we invited athletes, coaches, and sport psychologists to witness the vignettes and explored their responses via qualitative surveys and multiple interviews over time. Data analysis identified how the stories were useful for translating knowledge into practice, the reasons why, and how they acted on and for the participants. The accessibility of the stories enabled participants to connect meaningfully with the research. This expanded their knowledge, which some used in performances and to support other runners. Overall, our findings support an evolving body of research illustrating the potential of storied forms of representation to facilitate knowledge transfer, and extend understanding by providing a subject-specific (i.e., self-regulation and goal striving) and population-specific (i.e., running) example of how stories can work on and for athletes and practitioners.
180	Athletic identity and sport injury: a systematic review and meta-aggregation	Liu, SQ; Noh, YE; Kim, J	BMC PSYCHOLOGY	2025	10.1186/s40359-025-03902-7	S3	Scopus+WoS	ObjectiveThis review aimed to systematically review qualitative research examining changes in athletic identity following sports injuries.MethodsThe review was conducted in accordance with the Preferred Reporting Items for Systematic Reviews and Meta-Analyses (PRISMA 2020) guidelines. Electronic databases searched included Web of Science, Psychology & Behavioral Sciences Collection, PubMed, SPORTDiscus, and Scopus. Manual searches were conducted using forward and backward citation tracking via Google Scholar. Study quality was assessed using the Mixed Methods Appraisal Tool. Data extraction and synthesis followed the Joanna Briggs Institute guidelines, and the ConQual approach was used to evaluate the confidence level of findings based on dependability and credibility.ResultsA total of 24 qualitative studies were included. Using a meta-aggregative approach, findings were synthesized into two overarching themes: athletic identity disruption (n = 17) and athletic identity reconstruction (n = 7). The theme of athletic identity disruption highlighted that sports injuries often lead to a diminished sense of athletic identity due to factors such as physical limitations, withdrawal from competition, and psychological distress. In contrast, the athletic identity reconstruction theme showed that some athletes rebuild their athletic identity by engaging in alternative roles, such as coaching, volunteering, or participating in new sports.ConclusionSports injuries can have profound and varied effects on athletic identity. While some athletes experience disruption and identity loss, others successfully reconstruct their sense of self within the sporting context. Future research should consider more diverse study designs, such as longitudinal and mixed-methods studies, and further investigate how cultural, social, and contextual factors influence athletes' experiences of identity disruption and reconstruction.
181	A Novel Approach for Monitoring Training Load and Wellness in Women's College Gymnastics	Leupold, O; Cheng, J; Wimberly, A; Nguyen, J; Tilley, D; Gabbett, TJ; Casey, E	SPORTS HEALTH-A MULTIDISCIPLINARY APPROACH	2025	10.1177/19417381241296855	S3	WoS	Background: Monitoring training load has the potential to improve sport performance and reduce injuries in athletes. This study examined training load and its association with wellness in artistic gymnastics. Hypotheses: Training load and changes in training load (acute:chronic workload ratio [ACWR]) vary throughout 1 season; wellness is inversely correlated with training load and ACWR. Study Design: Prospective case series. Level of Evidence: Level 3. Methods: A total of 30 female collegiate gymnasts from 4 Division I National Collegiate Athletic Association teams participated (mean age, 20 +/- 2 years). During 4 months, before daily training, wellness surveys assessed sleep, energy, soreness, and mood (1-10; higher = better). After daily training, training load surveys assessed training duration per event (warm-up, vault, bars, beam, floor, strength and conditioning) and session rating of perceived exertion (RPE; 1-10; 10 = hardest) per event. Coaches reported technical complexity of training per event (1-4; 4 = hardest). Training load was calculated as [duration] x [RPE] x [technical complexity]. ACWR represented a ratio between acute [1-week] and chronic [4-week rolling average] training loads. Results: ACWR and weekly training load fluctuated throughout the season (ACWR mean weekly range: 0.68-1.11; training load mean weekly range: 2073-6193 arbitrary units). ACWR and weekly training loads were trichotomized into low, medium, and high groups; positive correlations were observed between each wellness variable and ACWR (P < 0.01) and between each wellness variable and weekly training load (P < 0.01). Conclusion: Our novel training load monitoring framework for women's college gymnastics enabled us to characterize training load and its relationship with wellness throughout 1 season. This method should be explored in gymnasts across various ages and competitive levels.

182	Examining the Role of Needs Satisfaction in the Coach-Athlete Relationship and in Athletes' Well-Being.	Caling, Jester Vann Y. ; Longakit, Jet C. ; Jariol, Kiana Polline Z. ; Lobo, Joseph ; Rodriguez, Deljun R.	Physical Culture & Sport. Studies & Research	2025	10.2478/pcssr-2025-0010	S1+S3	SPORTDiscus	Although various studies have explored the effects of the coach-athlete relationship and athletes' well-being across a number of contexts, there is scant literature indicating how satisfaction of needs (autonomy, competence and relatedness) mediates the relationship. This study aimed to examine the mediating role of needs-satisfaction, particularly autonomy, competence, and relatedness, on the coach-athlete relationship and the well-being of athletes. Using a cross-sectional design, a total of 248 competitive athletes (142 males and 106 females, M= 20.24, SD = 2.02) from various sports participated in this study. The results indicate that needs satisfaction (autonomy, competence and relatedness) partially mediated the coach-athlete relationship and the athlete's well-being. This suggests that a strong relationship between coaches and athletes creates a positive social environment that fulfills athletes' basic psychological needs. This encourages athletes to stay involved in sports and promotes overall well-being. The findings indicate that individuals thrive and experience physical and mental well-being proactively and healthy development when their basic psychological needs are met. Conversely, when these needs are unmet, individuals experience poor functioning, stunted growth, fatigue, and physical and mental distress. Future research should incorporate longitudinal forms of investigations into athletic populations and pay considerable attention to concrete methods of promoting the psychological needs outlined above in the specified athletic populations.
183	Burnout, Help Seeking, and Perceptions of Psychological Safety and Stigma Among National Collegiate Athletic Association Coaches.	Slowiak, Julie M. ; Osborne, Rebecca R. ; Thomas, Jordyn ; Haasan, Adna	International Sport Coaching Journal	2025	10.1123/iscj.2023-0107	S3	SPORTDiscus	Sport coaches face unique work-related demands that, over time, can lead to negative well-being outcomes, such as burnout. The link between burnout and mental health is supported in the literature, and public stigma around mental health has been identified as a prominent barrier to seeking help. The aim of this study was to investigate how burnout and help-seeking attitudes of National Collegiate Athletic Association coaches are impacted by psychological safety and public stigma associated with seeking help. A sample of 187 National Collegiate Athletic Association coaches participated in a cross-sectional online survey and reported sociodemographic and job-related characteristics, public stigma, self and team psychological safety, burnout, and attitudes toward help seeking. Regression-based mediation analyses revealed that greater psychological safety predicted lower levels of exhaustion and disengagement as well as more positive help-seeking attitudes. In addition, public stigma partially mediated the influence of psychological safety on exhaustion and disengagement and fully mediated the relationship between psychological safety and help-seeking attitudes. No differences in exhaustion, disengagement, and help-seeking attitudes among National Collegiate Athletic Association coaches across Divisions I, II, and III were observed. Findings lend support for intervention development to increase psychological safety as a burnout management strategy as well as to reduce public stigma associated with help seeking. ABSTRACT FROM AUTHOR
184	The processes of developing the coach-athlete relationship: a case study in women's university sport.	Corkery, Erin ; Fletcher, Tim	Sports Coaching Review	2025	10.1080/21640629.2023.2256139	S3	SPORTDiscus	The purpose of this research was to describe and interpret the processes of developing the coach-athlete relationship in a women's university sports team across one competitive season. Data were generated from observations of 19 team practices and 11 games, and two individual interviews with the coach and three athletes. Participants' unique perspectives of developing the coach-athlete relationship were examined through the use of Jowett's (2007) 3 + 1Cs model. Findings highlight the ambiguous and often contradicting ways that participants understood and interpreted the processes of their coach-athlete relationship, due in part by the performance and social agendas at play. This research makes a contribution to knowledge about the processes of the coach-athlete relationship in a team environment and application of the 3 + 1 Cs model by primarily attending to its processes rather than outcomes. In doing so, we showed some of the complexities in these processes and the challenges this offers to coaches and athletes. ABSTRACT FROM AUTHOR
185	Coaching Psychology in Athletic Bilbao: The Story From Its Beginnings.	O'Neill, John ; Campbell, Mark J. ; Sherwin, Ian	International Sport Coaching Journal	2025	10.1123/iscj.2023-0096	S3	SPORTDiscus	Athletic Bilbao, also known by its more correct name of Athletic Club, is a professional football club based in Bilbao, in the Basque Country of Spain. Since 1912, it has adhered to its policy of only allowing players from the Basque Country to play for them. This system is known as cantera, from the word for quarry, meaning treating players from the surrounding area as a valuable resource to be extracted and moulded. Despite the self-imposed limitation of this unwritten rule, Athletic Bilbao is one of only three clubs to have never been relegated from LaLiga, along with Real Madrid and Barcelona. Here, we look at how psychology was developed in the club toward developing home-grown players, which became known as La Mirada ("The Gaze" in Spanish). A key perspective of how La Mirada developed over time was to address coaches' mindsets before those of the players, especially because coaches often felt that their learning was going to be an upward trajectory by relying on what had given them results in other clubs. This Practical Advance paper explores this distinctive journey of psychology with examples from what was themed the lights and shadows of coaches' and players' learning development in Athletic Bilbao. ABSTRACT FROM AUTHOR

186	Systematic review and meta-analysis of Chinese coach leadership and athlete satisfaction and cohesion	Zhu, J; Wang, M; Cruz, AB; Kim, HD	FRONTIERS IN PSYCHOLOGY	2024	10.3389/fpsyg.2024.1385178	S1	WoS	Introduction This meta-analysis investigates the relationship between coach leadership behaviors and athlete satisfaction and group cohesion within the realm of Chinese sports. The study also explores player sex and player classification as potential moderating variables. The primary focus is on evaluating the impact of coaching behaviors, as measured by the Leadership Scale for Sports, on athlete satisfaction and group cohesion. Methods Standard literature searches from China National Knowledge Infrastructure and Wanfang academic databases produced 26 studies encompassing a total of 319 effect sizes and a participant pool of 7,121 athletes across various sports. Results Using the Comprehensive Meta-Analysis (CMA) to examine relevant data, results reveal a moderate and positive association between coach leadership and athlete satisfaction (ES = 0.412). Specifically, training and instruction (ES = 0.531), positive feedback (ES = 0.526), social support, and democratic decision-making exhibit positive effects, while autocratic behavior demonstrates a marginal positive effect. Similarly, a moderate positive relationship is identified between coach leadership and overall group cohesion (ES = 0.275), with training and instruction (ES = 0.396), social support (ES = 0.356), positive feedback, and democratic behavior positively influencing cohesion. Conversely, autocratic behavior has a small negative impact on cohesion. Furthermore, female athletes (ES = 0.603) and professional players (ES = 0.544) display stronger positive associations between coach leadership and satisfaction. Conclusion These findings highlight the significance of diverse coaching behaviors aligned with player characteristics for fostering positive athlete satisfaction and group cohesion within the Chinese sports context, offering valuable guidance to Chinese coaches aiming to enhance their coaching strategies.
187	The coach-athlete relationship within a cross-boundary team of experts: a conceptual analysis	Jowett, S	INTERNATIONAL REVIEW OF SPORT AND EXERCISE PSYCHOLOGY	2024	10.1080/1750984X.2024.2416968	S1	WoS	The coach-athlete relationship phenomenon two and a half decades ago was thought of as an uncharted territory, though today the coach-athlete relationship has a body of knowledge that grows by the day. Conceptual and empirical advancements have helped coaches and athletes to work with each other more intelligently within their sport teams, groups and organisations. While acknowledging that the coach and the athlete as a unit relationship is pivotal, this significant relationship especially in high-performance sport often operates within a complex web of other interpersonal relationships. These relationships include individuals of diverse disciplines (e.g. physiologists, psychologists, nutritionists, physiotherapists, physicians, performance analysts) and take place in various locations (i.e. within and outside an athlete's and a coach's organisation). The article aims to address: (a) the quality of the coach-athlete relationship and its role in athletes' and coaches' performance and wellbeing and (b) the diverse expertise and perspective coaches and athletes within their unit relationships leverage to accomplish challenging performance goals while ensuring positive wellbeing. The objective of this article is to open a dialogue around cross-boundary discipline performance teams and provide an impetus for concerted conceptual and empirical research activities that have significant practical impact in high-performance sport.
188	The influence of athletes' gratitude on burnout: the sequential mediating roles of the coach-athlete relationship and hope	Dong, LS; Zou, SS; Fan, R; Wang, B; Ye, L	FRONTIERS IN PSYCHOLOGY	2024	10.3389/fpsyg.2024.1358799	S1	WoS	Background Athlete burnout is a widespread psychological syndrome in competitive sports, negatively impacts athletes' competitive state and hampers the healthy development of sports organizations. With the rise of positive psychology, exploring the mechanisms of athlete psychological fatigue through the lens of psychological capital has become a focal point of recent research. This study introduces gratitude, a key element of psychological capital in positive psychology, to examine its effect on athlete burnout and its mechanism of action, with a particular focus on the sequential mediating roles of the coach-athlete relationship (CAR) and hope.Method A cross-sectional study design was utilized, involving 483 active Chinese athletes from national training teams and professional sports teams. The sample comprised both male (n=251) and female (n=232) athletes, with an average age of 19.24 +/- 3.99 years. Participants were asked to complete self-administered questionnaires, including the Gratitude Questionnaire, CAR Questionnaire, Hope Questionnaire, and Athlete Burnout Questionnaire. Structural equation modeling in AMOS 24.0 and descriptive statistics and correlation analyses in SPSS 20.0 were employed for data analysis.Results The study revealed significant associations between athlete gratitude, CAR, hope, and athlete burnout. Notably, gratitude was found to both directly and indirectly (via CAR and hope) influence burnout levels among athletes, suggesting a sequential mediation effect.Conclusion The findings highlight the importance of positive psychological constructs in buffering against athlete burnout. Specifically, gratitude, alongside a supportive CAR and elevated levels of hope, may play crucial roles in mitigating burnout symptoms. These insights offer promising directions for the development of targeted intervention strategies aimed at fostering athlete well-being and performance, advocating for the integration of positive psychology principles in the management and prevention of athlete burnout.

189	NCAA coaches' perceptions of the inclusion of transgender athletes: a qualitative analysis	Arnold, ML; Chambers, K; Petrie, TA	SPORT EDUCATION AND SOCIETY	2024	10.1080/13573322.2023.2283078	S1	Scopus+WoS	The National Collegiate Athletic Association (NCAA) established policies for the inclusion of transgender athletes to compete in collegiate sport that align with their gender identity. Despite this access, the extent to which collegiate sport environments are welcoming, inclusive and supportive of transgender athletes is unclear. Understanding coaches' perceptions and attitudes is particularly salient given the critical, and often personal, nature of coach-athlete relationships and the impact coaches have on sport environments [Kuhlin, F., Barker-Ruchti, N., & Stewart, C. (2020). Long-term impact of the coach-athlete relationship on development, health and wellbeing: stories from a figure skater. Sports Coaching Review, 9(2), 208-230; Stirling, A. E., & Kerr, G. A. (2013). The perceived effects of elite athletes' experiences of emotional abuse in the coach-athlete relationship. International Journal of Sport and Exercise Psychology, 11(1), 87-100]. NCAA coaches' perceptions of, and attitudes towards the inclusion of transgender athletes is unknown, and thus the degree to which coaches are creating safe, or harmful, environments for transgender athletes is unknown. The present study aims investigate coaches' perceptions to be able to inform policy and education for promoting inclusive, safe environments for transgender athletes. We asked NCAA coaches (N = 1358) to describe the reasoning for their beliefs regarding the inclusion of transgender athletes in collegiate sports. Through reflexive thematic analysis, we determined coaches' reasoning for their perceptions, within the context of five higher-order themes: (a) It's not right: Transgender identities as invalid, (b) A need to protect (cis) women's sports, (c) Transgender rights are human rights, (d) Sport's role in society and coaches' role in sport and (e) I am not sure what I believe. Analyses illuminate avenues for transgender athlete inclusion training for coaches.
190	Care as a strategy to navigate political tensions within the China-Norway sports coaching collaboration	Tjonndal, A; Haugen, HO	INTERNATIONAL REVIEW FOR THE SOCIOLOGY OF SPORT	2024	10.1177/10126902241247579	S1	Scopus+WoS	This study investigates the experiences of Norwegian coaches involved in a winter sports collaboration between Norway and China leading up to the 2022 Winter Olympic Games. Our analysis delves into the complexities of coach-athlete relationships in collaborations between nations with vastly different sports systems and cultures. Methodologically, the study is based on qualitative interviews with Norwegian coaches involved in the collaboration. Emphasizing the concept of care, we examine how Norwegian coaches navigated the cultural, bureaucratic, and political tensions that encompassed their relations with the Chinese winter sports athletes. The findings reveal that prioritizing care and athlete welfare serves not only as a coaching philosophy for the Norwegian coaches, but also as both a shield and a justification for their involvement, effectively sidelining broader ethical, and political considerations in the Norway-China winter sports collaboration. This research contributes to understanding the dynamics of international sports collaborations and coaches' strategies for maintaining integrity while engaging in international partnerships marked by different values and traditions.
191	Adverse competition-related cognitions questionnaire (ACCQ): Development and preliminary validation of a measure identifying different factors of athletes' cognitions related to competitions	Michel-Kröhler, A; Wessa, M; Berti, S	PSYCHOLOGY OF SPORT AND EXERCISE	2024	10.1016/j.psychsport.2023.102582	S1	WoS	This research project presents the development and preliminary validation of a German version of the Adverse Competition-related Cognitions Questionnaire (ACCQ) and comprises four studies. In Study 1, 101 athletes and 124 coaches from different team and individual sports generated a large pool of cognitions, which was reduced to an initial item pool of 54 cognitions through a multi-step procedure with different experts. In Study 2, the un-derlying factor structure of the questionnaire was examined by an exploratory factor analysis (N2 = 348), which revealed six factors (athletic comparison, coach devaluation, devaluation of one's own performance, apprecia-tion by coach and family, inner resistance against competitions, and general exhaustion) with 26 items retained. Subsequently, in Study 3, the results were re-examined with a confirmatory factor analysis (CFA, N3 = 419; CFI = 0.91, RMSEA = 0.06, SRMR = 0.06). In addition, preliminary support for the construct validity (i.e., convergent, divergent, and concurrent validity) of the ACCQ was obtained by examining associations among the ACCQ subscales and theoretical correlates such as social comparison, cognitive interference, irrational perfor-mance beliefs, and fear of negative evaluation. In Study 4, a second CFA (N4 = 153; CFI = 0.89, RMSEA = 0.06, SRMR = 0.07) was conducted to test the identified 6-factor solution in a sample of high-performance competitive athletes. With its broad range of factors, the ACCQ provides a useful and valid measure for assessing different adverse competition-related cognitions, offering a wide range of potential applications in research and sport psychology practice.
192	Exploring strength and conditioning practitioners' perceptions about using priming exercise as a pre-competition strategy to improve performance	Holmberg, PM; Russell, S; O'Brien, KA; James, LP; Kelly, VG	INTERNATIONAL JOURNAL OF SPORTS SCIENCE & COACHING	2024	10.1177/17479541231207951	S2	WoS	The term 'priming exercise' has recently been introduced to describe exercise stimuli prescribed to enhance performance within 1-48 hours of that stimulus. Despite evidence of these activities being utilised in competitive settings, the reasoning underpinning the application of priming exercise is not yet clear. Therefore, this study aimed to explore practitioners' perceptions about using priming exercise as a pre-competition strategy to improve performance. Semi-structured interviews were conducted with 28 practitioners involved in prescribing training programs to elite Australian athletes. Reflexive thematic analysis revealed six organising themes (i.e. 'limited and inapplicable research findings', 'consideration of athletes' training age', risk-return of priming session outcomes', 'logistical concerns', 'buy-in' and 'priming exercise prescription') later grouped into a global theme, representing the 'challenges of applying priming exercise'. The findings highlight contextual factors influencing practitioners' use of priming exercise and, based on these considerations, draw attention to the apparent uncertainty about whether these stimuli can be prescribed effectively to improve competition performance in sports. In addition to gaining insight into applied priming strategies, the present findings may be used to inform externally valid and relevant study designs that subsequently guide practice.

193	The effect of post-activation enhancement on the performance of Chinese national skeleton athletes in the ice push sled-a first cross-sectional study	Tian, G; Li, HJ; Zhu, H; Gao, BH	PEERJ	2024	10.7717/peerj.18271	S2	Scopus+WoS	Objective: To investigate the impact of post-activation potentiation (PAP) induced by resisted sled sprint at different loads on the subsequent 30 m ice push sled performance of Chinese skeleton athletes, and to identify the resisted sled sprint load that most effectively enhances PAP for Chinese skeleton athletes. Methods: Seven elite athletes from the Chinese skeleton team participated in four tests with more than 48 h intervals. During the tests, on the first test, athletes completed a 40 min standard warm-up, rested for 6 min, and then performed a 30 m test. On the second, third, and fourth test, athletes completed the standard warm-up, then performed 20 m sprints with resisted sled (RS) at 75%, 50%, and 25% of body mass (BM), respectively, rested for 6 min, and then performed the 30 m test. Results: No significant differences were found in morning pulse, blood urea, and creatine kinase levels among four tests. The percentage of maximum heart rate (%HRmax) within different intensity ranges showed no significant differences among four tests. However, significant differences were observed in ice push sled performance among four tests (No BMRS: 5.08 +/- 0.27; 25% BMRS: 5.05 +/- 0.29; 50% BMRS: 5.02 +/- 0.27; 75% BMRS: 5.04 +/- 0.28). Post hoc analyses revealed that the 50% BMRS test had faster speed compared to the no resistance ($p < 0.05$), the 25% BMRS ($p < 0.05$), and the 75% BMRS ($p < 0.05$) tests. Additionally, the 75% BMRS test had faster speed than the no resistance test ($p < 0.05$). Conclusion: A 20 m sprint with 50% BMRS effectively enhances the PAP effect in skeleton athletes, improving their ice push sled performance. Coaches can incorporate this resisted sled sprint in athletes' training routines for performance enhancement in both daily training and pre-competition preparations.
194	Psychological stress and psychological well-being among sports coaches: A close proximity longitudinal daily diary study	Potts, AJ; Didymus, FF; Kaiseler, M	JOURNAL OF APPLIED SPORT PSYCHOLOGY	2024	10.1080/10413200.2024.2344847	S3	Scopus+WoS	It is essential to understand sports coaches' experiences of psychological stress and psychological well-being (PWB) on a daily basis to better equip coaches to manage stress and improve well-being. Coaches make a vital contribution to sport and, given the potentially stressful nature of coaches' roles, are required to manage their own PWB and performance alongside that of the athletes with whom they work. To better understand how to support coaches, we explored coaches' experiences of stressors, primary appraisals, coping, and PWB using a close proximity longitudinal daily diary approach. Five sports coaches (one woman and four men) each completed an interval-contingent, daily diary over a 28-day period. The diary facilitated understanding of the coaches' experiences of their worlds and captured descriptions of the coaches' daily lives. Underpinned by our constructivist paradigm and following an abductive approach to data analysis, four experiences are presented (preparing for major events and championships, competition, admin and national governing body-related tasks, and work-life balance) using a trajectory approach. This method of data presentation allows understanding of each coach's journey through their experiences as they occurred over the 28-day period. The experiences are categorized into themes representing stressors, primary appraisals, coping, and PWB. Collectively, the findings highlight that coaches experienced certain stressors (e.g., athlete-related) on a regular basis, and that they appraised (e.g., as a challenge) and coped with these experiences in different ways (e.g., via information seeking). The findings also demonstrate that stress experiences influenced different elements of the coaches' PWB (e.g., relationships with others, personal growth) and that, occasionally, coaches may experience delayed or inaccessible appraisals of a stressor. Lay summary: Five sports coaches' experiences of psychological stress and psychological well-being are explored using close proximity daily diaries over a 28-day period. Four experiences (preparing for major events and championships, competition, admin and national governing body-related tasks, and work-life balance) are discussed to highlight how psychological stress influences coaches' psychological well-being.
195	Reciprocal relationships between teamwork execution, emergent states, and perceived team performance: a longitudinal study	López-Gajardo, MA; Leo, FM; Díaz-García, J; McEwan, D	INTERNATIONAL JOURNAL OF SPORT AND EXERCISE PSYCHOLOGY	2024	10.1080/1612197X.2023.2263863	S3	WoS	The purpose of this study was to analyze the reciprocal relationships between teamwork execution (i.e., communication, cooperation, and coordination), emergent states (i.e., group cohesion and collective efficacy), and perceived team performance among sport teams over the course of a competitive season. We also examined whether (a) emergent states mediated the relationship between teamwork execution and perceived team performance, and (b) teamwork execution mediated the relationship between emergent states and perceived team performance. We adopted a longitudinal design, taking measures of each variable near the beginning, middle, and end of teams' seasons. Participants included 657 athletes (104 teams), aged 16-43 years ($M = 23.56$, $SD = 5.72$; 71% men), and from a range of team sports and competitive levels. Structural equation models showed teamwork execution was positively and reciprocally related to collective efficacy as well as task cohesion (but not social cohesion). Teamwork execution also predicted perceived team performance over time but a reciprocal effect was not evident. Finally, task cohesion and collective efficacy mediated the longitudinal association between teamwork execution and perceived team performance; however, teamwork execution did not mediate the relationship between those emergent states and perceived team performance. These findings provide initial evidence of longitudinal relationships between teamwork execution, two emergent states, and perceived performance. It appears particularly important that coaches and practitioners focus on developing teamwork execution from the outset of their team's season as this appears to translate over time into greater task cohesion and collective efficacy and, in turn, team performance.

196	Changes in Sports Participation, Specialization, and Burnout From 7th to 12th Grade: Final Results From a 6-Year Longitudinal Study	Valenzuela-Moss, J; Sini, M; Wren, TAL; Edison, BR	SPORTS HEALTH-A MULTIDISCIPLINARY APPROACH	2024	10.1177/19417381231224792	S3	WoS	Background: Previous studies show alarming rates of burnout and dropout from sports participation and physical activity in the youth population. Early sports specialization may increase the risk of injury, burnout, and eventual dropout from sports. Hypothesis: Sports participation will decrease, specialization will increase, and burnout will increase from junior high to high school. Study Design: Prospective longitudinal study. Level of Evidence: Level 2b. Methods: The graduating class of 2023 was surveyed longitudinally in the fall of each school year from 7th to 12th grade (n = 35-77 from middle to high school based on new enrollment participants and attrition). Survey questions asked about sports participation, specialization, and burnout. Responses were analyzed by grade level using mixed effects linear and logistic regression accounting for repeated measures. Results: The number of days per week in which students engaged in at least 60 minutes of strenuous exercise progressively decreased from 7th through 12th grade from 4.0 to 2.3 days per week (P < 0.01). The percentage of students participating in sports also decreased from 7th to 12th grade from 82% to 39% (P < 0.01). More students stopped participating in a sport (38% vs 22% to 29%, P = 0.04), quit a sport to focus on a single sport (60% vs 46% to 49%, P = 0.01), or reported that 1 sport was most important to them (71% vs 54% to 67%, P = 0.06) in 9th grade. Burnout in sports did not differ significantly by grade level (19% to 23%, P > 0.99). In contrast, burnout in school was significantly more common in high school (54% to 69%) compared with middle school (36%, P < 0.01). Burnout in school was higher in female students compared with male students (68% vs 38%, P < 0.01). Conclusion: Organized sports participation and general physical activity decreased from middle to high school, with the entry into high school at 9th grade being a time at which a significant number of students dropped other sports to specialize in a single sport or stopped participating. Burnout in sports did not appear to increase with age; however, there was an increase in school-related burnout as students transitioned from middle to high school. Clinical Relevance: Our results highlight the need for continued research into sports participation, specialization, and burnout in adolescents. In addition, health professionals and the community (parents, coaches, teachers) supporting youth athletes may consider positive interventions during the periods of transition from middle to high school to include facilitating different tracks of sports participation
197	Organizational stress and well-being in competitive sport: a systematic review	Simpson, RAC; Didymus, FF; Williams, TL	INTERNATIONAL REVIEW OF SPORT AND EXERCISE PSYCHOLOGY	2024	10.1080/1750984X.2021.1975305	S3	WoS	Research on organizational stress in sport has expanded within recent decades. Despite voluminous literature, no systematic reviews exist to congregate findings in a single, rigorous point of reference. Filling this void helps researchers and practitioners to understand organizational stress and its implications for health, well-being, and performance. The objectives of this study were to search for, appraise, and critically synthesize the literature on organizational stress (i.e. stressors, appraisals, coping, emotions) and or well-being within the context of organizational stress in competitive sport, with the intention of progressing conceptual, methodological, and applied understanding. A systematic review was conducted using PRISMA guidelines and robust searches of PsycArticles, PsycInfo, and SPORTDiscus databases. The final sample comprised 55 methodologically sound studies with athletes, coaches, and or support staff. Findings revealed a myriad of organizational stressors, underpinned by a range of situational properties, and managed using various coping options (e.g. problem solving). Appraisals, emotions, and well-being received scant attention in the context of organizational stress. Experiences of organizational stress among athletes, coaches, and support staff have highlighted implications for health - and performance-related outcomes. Future research should consider longitudinal, diary, integrative designs, and analytical pluralism within under-represented populations (e.g. coaches, support staff) to deepen our understanding.
198	Factors influencing sport persistence still represent a knowledge gap - the experience of a systematic review	Kovács, KE; Szakál, Z	BMC PSYCHOLOGY	2024	10.1186/s40359-024-02098-6	S3	Scopus+WoS	Investigating sport persistence can make a significant contribution to a thorough examination and understanding of the factors underlying an athlete's activity, integrating physical activity, sports motivation and commitment. This systematic review aimed to detect the papers focusing on the factors influencing sport persistence following the levels of Bronfenbrenner's and Bauman's ecological models. Following the PRISMA guidelines, by the EBSCO Discovery Service Search Engine, 512 records were detected, of which 21 met the criteria. Factors influencing sport persistence show a huge variety. Individual factors and micro-system level were overrepresented. Meso- and macro-level factors are underrepresented and measured only with other (individual or micro-system level) factors. Most factors detected at the individual (e.g. gender, age, career-related variables and positive psychological factors), micro- (e.g. family, peers and coaches), meso- (e.g. climate), and macro-level (cultural, societal, and institutional influences) were positively correlated with sport persistence, i.e. supporting the persistent behaviour of the athletes regarding participating in one's sporting activity. In conclusion, there is still room for further research concerning sport persistence. Including variables belonging to the meso- and macro-system and conducting longitudinal research should be considered which would allow us to better understand the nature of sport persistence.

199	The mediating roles of pre-competitive coping and affective states in the relationships between coach-athlete relationship, satisfaction and attainment of achievement goals	González-García, H; Martinent, G; Nicolas, M	INTERNATIONAL JOURNAL OF SPORT AND EXERCISE PSYCHOLOGY	2024	10.1080/1612197X.2023.2190346	S3	SPORTDiscus+WoS	The study is aimed to explore the relationship between coach-athlete relationship, precompetitive coping and affective states, satisfaction and attainment of achievement goals. A sample of 567 French athletes (Mage = 22.10; SD = 5.66; 377 men and 190 women) involved in 75 training groups participated in the study and completed a series of self-report measures in a temporal design with three measure points. Results of multilevel analyses revealed that the coach-athlete dyad (level 2; between-person level of analysis or training groups) and task-oriented coping (level 1; within person level of analysis or individuals) significantly predicted goal attainment (level 1). Moreover, the results of the Sobel test revealed that task-oriented coping (level 1) marginally mediated the relationship between coach-athlete dyad (level 2) and sport satisfaction (level 1). Besides, the coach-athlete dyad (level 1) and Intensity of positive affect (PA) mediated the relationship between coach-athlete dyad and goal attainment. Finally, the coach-athlete dyad (level 1) and Intensity of PA mediated the relationship between coach-athlete dyad and satisfaction (level 1). In conclusion, the display of Coach-Athlete relationship strategies may enhance higher levels of precompetitive task-oriented coping, intensity of PA, goal attainment and satisfaction. Subsequently, coaches and practitioners should focus their training on enhancing coach-athlete relationship as a crucial part of training in order to perform in competitions.
200	Validation of the Coach Behavior in Sports Questionnaire: Towards dynamic assessments using the circumplex model for coach behavior	Van Meervelt, K; Reynders, B; Van Puyenbroeck, S; De Backer, M; Hofmans, J; Vande Broek, G	PSYCHOLOGY OF SPORT AND EXERCISE	2024	10.1016/j.psychsport.2024.10269 1	S3	WoS	The development of the circumplex model for coach behavior recently provided researchers an integrative model to assess coach behavior. While the circumplex model has currently only been deployed in cross-sectional research designs using the Situations in Sports questionnaire (SISQ), it has clear potential within research on dynamics of coach behavior. However, the SISQ consists of vignette-based situations and is too extensive for such frequent administrations. Within the present study we therefore developed the Coach Behavior in Sports Questionnaire (CBSQ), a 32-item alternative for the SISQ which consists of general items rather than vignettebased situations. First, content validity of 54 initial pilot CBSQ items was tested in a sample of 34 expert coaches. Second, construct validity, predictive validity, and reliability of the 32-item CBSQ was tested in a sample of 689 athletes and 420 coaches. Third, within-person variability of coach behavior was longitudinally assessed using the 32-item CBSQ during a five-week training and game period in a sample of 31 coach-athlete dyads. The 32-item CBSQ showed to be a valid and reliable alternative for the SISQ. Also, coach behavior showed considerable within-person variability over the five-week period in all coaching styles and approaches. Additionally, a 24, 16, and 12-item version were tested to provide even shorter alternatives. The CBSQ opens new horizons for future longitudinal research and coach reflection programs based on the circumplex model for coach behavior.
201	A Longitudinal Multicase Study About the Board-Game Format of an Educational Self-Talk Intervention	Mathas, V; Solomon-Turay, P; Hatzigeorgiadis, A; Hardy, J; Latinjak, AT	SPORT PSYCHOLOGIST	2024	10.1123/tsp.2023-0150	S3	Scopus+WoS	This study explored goal-directed self-talk and its optimization through educational self-talk interventions, focusing on a novel sports-oriented board-game intervention. Examining a practitioner's experiences and diverse participant profiles, including an elite tennis player, a recreational athlete, a football referee, and a tennis coach, the research employed four in-depth case studies. A unique aspect of this study involved intentional control transfer from practitioner to client, documented through a practitioner diary and postintervention interviews. Participants generally expressed appreciation for the intervention; however, the coach's case necessitated bespoke adjustments, underscoring the importance of contextual sensitivity in applied practice. The educational self-talk intervention reportedly facilitated the analytical deconstruction of challenges, enhancing self-regulation through goaldirected self-talk. The study underscored the pedagogical merit of the board game and observed a substantive transfer of control from practitioner to participant over the intervention's longitudinal trajectory. Detailed recommendations have been crafted for practitioners to implement the educational self-talk intervention effectively in their practice.
202	Initiatives to facilitate the social inclusion of people with intellectual disability in physical activities with others in community-based sporting, recreation and leisure activities: A scoping review	Ristevski, E; McCook, F; Thompson, S; Duncan, Z; Lawler, B; Broxham, C	JOURNAL OF APPLIED RESEARCH IN INTELLECTUAL DISABILITIES	2024	10.1111/jar.13238	S3	Scopus+WoS	BackgroundWhile barriers to participation in physical activity are well documented, there is no consolidated evidence on strategies promoting participation of people with intellectual disability in physical activities.MethodsWe conducted a scoping review to examine initiatives used to facilitate the inclusion of people with intellectual disability in community-based sporting and recreation activities. We searched Medline, Embase, CINAHL+ and PsycINFO for articles published in English between 2000 and 2022.ResultsEight articles were included. Initiatives included: (1) building knowledge, awareness and attitudinal change (2) organisational alliances/partnerships (3) relationship-building (4) modifications/adaptions to activities/environment (5) organisational policy (6) engagement with people with disability. Initiatives were implemented through international collaborations, in local fitness/recreational facilities and public spaces, and targeted athletes, coaches, volunteers and organisational leaders.ConclusionWhile some increase in awareness and inclusion of people with intellectual disability was reported, no longitudinal data reported on the impact of initiatives on sustained social inclusion for people with intellectual disability.

203	Changes in Body Composition and Athletic Performance in National Collegiate Athletic Association Division I Female Field Hockey Athletes Throughout a Competitive Season	Summer, LC; Cheng, R; Moran, JT; Lee, M; Belanger, AJ; Taylor, WL IV; Gardner, EC	JOURNAL OF STRENGTH AND CONDITIONING RESEARCH	2024	10.1519/JSC.0000000000004591	S3	WoS	The purposes of this study were (a) to analyze the changes in total and regional body composition measurements in a National Collegiate Athletic Association (NCAA) Division I female field hockey team throughout a 17-game competitive season using dual X-ray absorptiometry (DXA); (b) to examine improvements, if any, in athletic performance measures after a season; and (c) to report on the relationship between these body composition changes and changes in athletic performance. Preseason and postseason dual-energy DXA and performance data from the 2019-2020 season were retrospectively identified for 20 field players (forwards, midfielders, and defenders). Body composition data included total and regional fat mass, lean mass, and body fat percentage, whereas athletic performance measures included the vertical jump, 10-yard dash, and pro-agility (5-10-5) shuttle run. All variables were quantitative and analyzed using paired t-tests or its nonparametric equivalent and an alpha level of $p < 0.05$ was used to determine significance. After a competitive season, athletes had significant decreases in fat mass and increases in lean mass in their arms, legs, trunks, gynoids, and total body measurements. Android fat mass and body fat percentage also decreased. Athletes performed significantly better on the pro-agility shuttle run at the end of the season, but no significant differences were observed in other performance metrics. Moderate correlations were observed between changes in body composition (total fat mass and total lean mass) and changes in athletic performance. Our study provides a novel, longitudinal assessment of body composition and athletic performance for elite female field hockey athletes that will help trainers and coaches better understand how these variables change throughout a season and allow them to better prepare their players for competitive success.
204	Association between concussion understanding and stakeholder knowledge translation in collegiate sports	Giguere, D; Barnes, M; Faught, BE	INTERNATIONAL JOURNAL OF SPORTS SCIENCE & COACHING	2024	10.1177/17479541231224273	S3	WoS	The purpose of this study was to determine if there was an association between concussion understanding and stakeholder knowledge translation in collegiate sports following the mandate of Rowan's Law in Ontario, Canada. To our knowledge, this is the first study to examine concussion knowledge translation within a sport network using social network analysis. A cross-sectional design was used to evaluate 76 collegiate athletes (54 females, 21 males, 1 not identified), aged 20.55 years (SD = 3.4) who completed a survey on sport demographics, concussion knowledge and stakeholders who provided concussion information during the sport season. Athlete concussion knowledge scores and reported stakeholders were examined. An average of three key stakeholders provided concussion information to 82% of the varsity athletes in our study. Athletes reported that a coach or athletic trainer most often provided concussion knowledge. Overall, athlete concussion knowledge scores were the same for athletes who sought concussion knowledge from stakeholders and those who did not. Over 95% of athletes in the study did not access the Rowan's Law website for mandated concussion education. These findings suggest that Rowan's Law is hugely neglected resulting in stakeholder knowledge translation having minimal influence on an athletes' understanding of concussions. Future recommendations include verified review of mandated concussion education resources and testing of concussion knowledge for all persons associated with sport in Ontario. Due to the number of athletes seeking concussion knowledge in their varsity athlete network, accurate sport specific resources should be provided to support stakeholders who are in direct contact with athletes.
205	The effects of mobile phone dependence on athletic performance and its mechanisms	Mei, ZY; Zhang, YZ; Fan, Q; Luo, SL; Luo, S	FRONTIERS IN PSYCHOLOGY	2024	10.3389/fpsyg.2024.1391258	S3	WoS	Mobile phone dependence (also known as internet dependence, MPD), defined as a problematic behavior characterized by excessive use or intermittent craving to use a mobile phone, results in various social, behavioral, and affective problems in daily life. In sports, MPD is directly related to the physical and mental health and sports performance of athletes. The individual and environmental factors, neurobiological mechanisms and theoretical models of MPD affecting athletic performance were analyzed by reviewing previous studies, aiming to construct effective training and development protocols to prevent and control the occurrence of MPD in athletes. At present, athletic performance can be affected by MPD through individual factors and environmental factors. The neurobiological mechanisms between the two are based on the brain reward system and microwave radiation from mobile phones, with athletic performance being restricted by alterations in the corresponding brain regions. Relevant theoretical models mainly include the social cognitive model of self-regulation and the integrative model of self-control, which explain the interrelationship between MPD and athletic performance from the perspectives of athletes' self-regulation and self-control, respectively. As an emerging phenomenon, the influence pathways and mechanisms by which MPD affects athletic performance need to be further investigated. A longitudinal perspective should be adopted to trace the dynamic impact relationship between the two, and developing relevant theoretical frameworks from an interdisciplinary research perspective should be valuable for providing theoretical support for coaches and sports administrators to formulate scientific training protocols and thus improve the mental health of athletes.
206	College Football Kids: Infantilizing Language in Football Bowl Subdivision Bowl Game Broadcasts	Corr, C; Southall, C; Hawkins, B; Southall, RM	SOCIOLOGY OF SPORT JOURNAL	2024	10.1123/ssj.2022-0185	S3	WoS	Paternalistic institutional structures are strategically arranged to maintain locus of control and preserve male-centric patriarchal authority. A confluence of cultural, social, and legal structures perpetuates paternalism within National Collegiate Athletic Association college sport and specifically in Football Bowl Subdivision (FBS) football. This study examined FBS bowl game broadcasts to determine the prevalence of paternalistic and infantilizing commentary. An analysis of in-game commentary from a sample of 18 FBS bowl games from the 2019 to 2020 season revealed that commentators frequently infantilize FBS football players, normalizing a paternalistic and exploitative coach-athlete relationship.

207	A framework for mental training in sport: Enhancing mental skills, wellbeing, and performance	Vealey, RS	JOURNAL OF APPLIED SPORT PSYCHOLOGY	2024	10.1080/10413200.2023.2274459	S3	Scopus+WoS	Frameworks that illustrate the comprehensive process of mental training have provided a big picture of the provision of applied sport psychology services. The purpose of this article is to propose an updated framework for mental training in sport. The revised framework includes four categories of mental skills including personal foundation, performance, personal development, and team skills. Mental health, as represented by emotional, psychological, and social wellbeing, is also included in the framework as a major focus for applied work by mental performance consultants, not just clinical mental health providers. The flow of the framework suggests that the consultant's philosophy and skills in using various models, strategies, and techniques, coupled with athlete commitment, can enhance athletes' mental skills and mental health to enhance performance and other significant outcomes. Suggestions for future work in mental training in sport include research examining the role of mental health in mental skills development, implementation of mental health enhancement programs, periodized approaches to sport psychology service delivery, and a renewed focus on intentionality in athletes to build mental skillfulness over time and situations for an adaptive, agile mindset.Lay summary: Mental training is a process in which athletes, usually with the help of consultants or coaches, systematically use strategies and techniques to build mental skills and wellbeing to enhance their performance, development, and experiences in sport. Enhancing mental health in athletes is an important objective for mental performance consultants. Consultants base their service delivery on a foundational philosophy and model of practice, from which they utilize and integrate various mental training strategies and techniques.Mental skills are learnable psychological capabilities that facilitate individuals' success in life pursuits.Mental health focuses on athletes' wellbeing and how much they are flourishing; it is not merely the presence or absence of mental illness.
208	A review of the interpersonal experience, expression, and regulation of emotions in sport	Tamminen, K; Wolf, SA; Dunn, R; Bissett, JE	INTERNATIONAL REVIEW OF SPORT AND EXERCISE PSYCHOLOGY	2024	10.1080/1750984X.2022.2132526	S3	WoS	There has recently been a surge in sport psychology research examining various aspects of the interpersonal and social processes related to emotions and emotion regulation. The purpose of this study was to review the literature related to the interpersonal experience, expression, and regulation of emotions in sport, in order to provide a comprehensive overview of the studies that have been conducted to date. A scoping review of the literature (Grant, M. J., & Booth, A. [2009]. A typology of reviews: An analysis of 14 review types and associated methodologies. Health Information and Libraries Journal, 26(2), 91-108) using a systematic search process returned 7,769 entries that were screened for inclusion; the final sample of studies included in the review consisted of 79 relevant articles and 8 dissertations. The results describe the interconnected findings on athletes' self-regulation of emotions in social contexts, interpersonal emotion regulation, collective emotions (group-based emotions, emotional contagion, and effervescence), emotional expressions, and individual and contextual moderators (e.g. personality, culture, norms, gender, roles, and situational/temporal aspects). We identify key issues to advance theory and research, including: the need for programmatic research to investigate these processes, their effects, and underlying mechanisms; greater theoretical and conceptual clarity; more research among diverse populations (e.g. female athletes, youth athletes); the need to consider interconnected emotional phenomena in future research; and the need for applied intervention research.
209	The Relationship Between Mental Fatigue and Shooting Performance Over the Course of a National Collegiate Athletic Association Division I Basketball Season	Daub, BD; Mclean, BD; Heishman, AD; Peak, KM; Coutts, AJ	JOURNAL OF STRENGTH AND CONDITIONING RESEARCH	2024	10.1519/JSC.0000000000004624	S3	WoS	Daub, BD, McLean, BD, Heishman, AD, Peak, KM, and Coutts, AJ. The relationship between mental fatigue and shooting performance over the course of a National Collegiate Athletic Association Division I basketball season. J Strength Cond Res 38(2): 334-341, 2024-The aim of this investigation was to examine the presence of mental fatigue and concurrent changes in shooting performance across various experimental weeks throughout a National Collegiate Athletic Association (NCAA) basketball season. Fifteen elite male NCAA Division I collegiate basketball players (age 20.2 +/- 1.2 years, height 199.3 +/- 7.1 cm, and body mass 93.1 +/- 8.6 kg) volunteered for this study. Mental fatigue and basketball shooting performance was evaluated at 4 timepoints with varying seasonal demands: high game volume (GAME), high academic load (ACADEMIC), no games and no academic load (PRACTICE), and standard number of games and academic requirements (TYPICAL). Subjective mental fatigue increased significantly ($p <= 0.05$) from Pre to Post brief psychomotor vigilance test (PVT-B) measurements at the end of the ACADEMIC week ($p = 0.002$, $d = 1.51$) and from beginning to end of the ACADEMIC week ($p < 0.001$, $d = 2.21$). Ratings of mental effort were significantly increased during the ACADEMIC week ($p < 0.001$, $d = 1.67$). Recovery stress questionnaire (REST-Q) showed significant differences between week GAME and ACADEMIC with an increase in Social Stress ($p = 0.001$, $d = 0.84$), Fatigue ($p = 0.021$, $d = 1.12$), Disturbed Breaks ($p = 0.024$, $d = 0.57$), and Emotional Exhaustion ($p = 0.035$, $d = 0.75$). Lower shooting performance was observed during the ACADEMIC week from Pre to Post ($p = 0.009$, $d = 0.35$) and higher scores Pre to Post in the TYPICAL week ($p = 0.008$, $d = 0.25$). Basketball shooting performance was significantly reduced after increased levels of mental fatigue stemming from added academic stress. In addition, an increase in sport-specific training or games had no effect on subsequent basketball shooting performance. Special consideration should be given by coaches around examination periods because the existence of academic stressors can influence basketball shooting performance.

210	Tracking relations between development of tactical knowledge and tactical behaviour: a season-long action research study	Ramos, A; Davids, K; Coutinho, P; Mesquita, I	PHYSICAL EDUCATION AND SPORT PEDAGOGY	2024	10.1080/17408989.2022.2083099	S3	WoS	Background Specific learning experiences are vital for athletes to continuously interact with developing knowledge of the performance environment, through the refined design of representative learning contexts (ecological paradigm), and appropriate didactical interventions (constructivist paradigm). Although there is a well-established relationship between tactical knowledge (TK) and tactical behaviours (TB), research has investigated the development of both separately. Thus, the inter-dependency between knowledge and behaviour has been inferred, but not contextually interpreted in a process-oriented analysis of performance. Purpose Through an insider action-research (AR) design, and adopting a mix-methods approach, this study aimed to: (i) investigate in-depth the influence of a constructivist-ecological pedagogical intervention on developing players' TK and TB; (ii) explore effects of integrated tactical knowledge and behaviour on competitive performance. Participants and settings Fifteen female volleyballers participated in this study. An insider-AR (first author assumed the dual role of coach and researcher) was conducted from September 2017 to June 2018. In total, four AR-cycles were completed. A mixed-method approach was used to obtain distinct, but complementary, data on the impact of a constructivist-ecological pedagogical intervention on development of players' TK and TB. Qualitative data were used to explore the development of TK, while TB was analysed through quantitative methods. Data collection Qualitative data on the coach's perceptions were collected using a reflexive diary and field notes, while players' perceptions were recorded through four semi-structured focus-group interviews, one per AR-cycle. Quantitative data from four official competitive matches, one per AR-cycle, were analysed. Players' positional coordinates were used to calculate the synchronisation tendencies of lateral blocking and defensive lines through the cluster-phase method. Data analysis Qualitative data were analysed using thematic analysis, in which inductive procedures deepened understanding of the development of the players' TK. A 4 (matches) x 1 (court direction) repeated-measures ANOVA was used to analyse quantitatively the differences in the mean cluster-amplitude values of lateral blocking and defensive lines between matches. Main findings The unique integration of constructivist and ecological principles scaffolded the development of players' TK and TB. Players evolved from an initial stage in which they verbally described competitive game
211	Making the Invisible, Visible: An Exploration of Track-and-Field Coaches' Perspectives of Their Planning Processes	Johns, KL; Potrac, P; Hayes, PR	INTERNATIONAL JOURNAL OF SPORTS PHYSIOLOGY AND PERFORMANCE	2024	10.1123/ijspp.2023-0467	S3	WoS	Purpose : The traditional understanding of how coaches plan for sporting performance is rooted in the assumption that coaches create periodized plans underpinned by physiological principles, thereby providing scienti fi c credibility to their work. In contrast, there remains a paucity of literature exploring how coaches understand or think about their planning practices. The purpose of this study was to generate new knowledge regarding what information coaches actually consider within their planning processes and how they actually approach the task of planning. Method : Using rigorous, in-depth interviews, this study examined athletics coaches ' understandings of their everyday planning practices, in an attempt to contribute to narrowing the gap between academic research and real-world application. Twenty-eight highly experienced, high-performance track-and- fi eld coaches based in England (female, n = 1; male, n = 27) were recruited. The coaches were from the throwing disciplines (n = 10) and endurance running (n = 18). Coaches were interviewed about their planning process, using a maximum of 3 semistructured interviews per coach, spaced across a full athletics season. In total, this generated 68 hours of data. Results : The analysis demonstrated that, while the participant coaches utilized the principles of periodization, their planning activities were not limited to this issue. The fi ndings highlighted how the coaches conceptualized successful athletic performance in a holistic way; that is, planning is multifaceted in nature. Conclusion : This study presents a holistic picture of the complexity of coaches ' planning, detailing the considerable time and attention given to planning for athletes ' psychological, personal, and social development, to enhance athlete performance and development.
212	Optimization of Sprint Training Among European Coaches: Quality Over Quantity	Agudo-Ortega, A; Sandbakk, O; Salinero, JJ; Johansen, B; González-Rave, JM	INTERNATIONAL JOURNAL OF SPORTS PHYSIOLOGY AND PERFORMANCE	2024	10.1123/ijspp.2024-0042	S3	WoS	Purpose : To describe how high-level European sprint coaches (from 100 to 400 m) work to improve important factors associated with the quality of the holistic training process and the quality of the speci fi c training session. Methods : A descriptive analysis was conducted using questionnaires from 31 European elite sprint coaches (ie, training athletes de fi ned as tiers 3, 4, and 5) who participated voluntarily. Results : The coaches used traditional periodization (45%) with a 10- to 15-day tapering phase (48%) that includes a reduction in volume, maintenance of intensity, and focus on correct technical execution. In the 3 mesophases, coaches prioritized the basic development of strength and sprint work in the fi rst phases of the season and emphasized more sprint-speci fi c work in the competitive phase. Before sessions, adjustments were made based on factors such as psychological (77%), technical (48%), and physical (39%) parameters. In-session load management relies on a combination of objective and subjective measures (55%), in which the dialogue with athletes (65%) was regarded as the main resource. Feedback during and after sessions covers technical (54%), psychological (48%), and physical (35%) aspects. Recovery protocols after sessions mainly involve rest and professional guidance (42%). For performance assessment and testing, coaches utilize countermovement jump (52%), force - velocity pro fi le (45%), and 30-m fl ying (61%) as main tools. Conclusions : European sprint coaches demonstrated a comprehensive approach to planning and management, shedding light on the multifaceted nature of their training methodologies and the diverse tools employed for athlete testing and monitoring.

213	THE INFLUENCE OF CLIMATE ON OUTDOOR ATHLETIC TRAINING REGIMES IN NORWAY	Ostgaard, N	REVISTA INTERNACIONAL DE MEDICINA Y CIENCIAS DE LA ACTIVIDAD FISICA Y DEL DEPORTE	2024	10.15366/rimcafd2024.95.022	S3	WoS	This study investigates how the country's environment impacts Norway's outdoor athletic training regimens. Athletes and coaches in various sports have special chances and problems due to Norway's environment, which is marked by frigid temperatures, protracted winters, and varied topography. The research was based on primary data analysis to determine whether the research used smart PLS software and generated informative results, including descriptive statistics, correlation coefficient analysis, also the smart PLS Algorithm model between them. The main topics covered are the prevalence of winter sports, adjusting to low temperatures, changing training regimens according to the season, using various terrain, and psychological toughness. The overall research found climate's positive and significant influence on the outdoor athletic training regimes in Norway. Furthermore, the impact of technology developments on weather - related risk mitigation and training regimen optimization is explored. Athletes and coaches in Norway manage and succeed in outdoor sports endeavors via resilience, adaptability, and a profound grasp of how climate affects training, demonstrating the complex interactions between sport and the natural environment.
214	Research note: Evaluating the efficacy of an online post event reflection tool in competitive sport	Bruner, MW; Mclaren, CD; Turcotte, D; Marshall, C	JOURNAL OF APPLIED SPORT PSYCHOLOGY	2024	10.1080/10413200.2023.2296902	S3	WoS	In competitive sport environments, coaches and sport psychology practitioners work to optimize individual and team performance. One strategy identified as a key to optimal sport performance is athlete self-awareness. This study evaluated the efficacy of a novel, online post-event reflection (PER) tool. Using a mixed methods design, 22 members of a competitive women's ice hockey team completed the PER across one season. The PER demonstrated efficacy through tailored insight used as a basis for athlete discussions, complementing other performance-related feedback. Follow-up interviews with three team members and the head coach provided additional support for the efficacy of the PER.Lay summary: In competitive sport environments, coaches and sport psychology practitioners work to optimize individual and team performance. This study provides evidence to support the use a novel, online post-event reflection tool by athletes, coaches, and sport psychology practitioners to enhance athlete self-awareness of mental skills and performance. IMPLICATIONS FOR PRACTICE center dot When striving to optimize performance, coaches and sport psychology practitioners should consider strategies to enhance athlete self-awareness center dot The post event reflection (PER) tool is an effective online tool for athletes, coaches, and sport psychology practitioners to enhance athlete self-awareness of mental skills and performance center dot The PER is also a valuable diagnostic tool for coaches and sport psychology practitioners to identify individual athlete and team patterns in mental skills and performance
215	A Systematic Review with a Meta-Analysis of the Motivational Climate and Hedonic Well-Being Constructs: The Importance of the Athlete Level.	Lochbaum, Marc ; Sisneros, Cassandra	European Journal of Investigation in Health, Psychology & Education (EJIHPE)	2024	10.3390/ejihpe14040064	S1	SPORTDiscus	Motivational climate is known to relate to individual behaviors, emotions, and thoughts. Hedonic or subjective well-being includes self-assessed positive affect (i.e., pleasant affect, moods, and emotions), negative affect (i.e., unpleasant affect, moods, and emotions), and life or domain-specific satisfaction. The aim of this review was to quantify the relationships between task and ego motivational climate scales and measures representing hedonic well-being with sports participants. Potential moderators of the motivational climate and hedonic well-being were examined. This review followed the PRISMA guidelines (PROSPERO ID CRD42023470462, registered 28 October 2023). From five relevant databases, one relevant review, and hand searching, 82 articles totaling 26,378 participants (46.3% female) met the inclusion criteria. The articles spanned publication dates from 1993 to 2023, representing 18 countries, various team and individual sports, and athletes competing in elite (e.g., Olympic) to grassroot (e.g., club sport) competitions. To meta-analyze the motivational climate and hedonic well-being relationships, the random-effects model was used. For the moderation analyses, the mixed-effects model was used. The task or mastery climate relationships were medium in magnitude with positive affect and satisfaction and small with negative affect. The ego or performance climate relationships were small in magnitude for positive affect, negative affect, and satisfaction. Evidence of bias existed in the motivational climate and hedonic well-being relationships. For moderation analyses, athlete level (i.e., elite vs. non-elite) moderated ($p < 0.05$) the task (elite, $r = 0.23$; non-elite, $r = 0.34$) and ego motivational climate (elite, $r = −0.02$; non-elite, $r = −0.13$) and positive affect and satisfaction combined relationships. In conclusion, the motivational climate and hedonic well-being relationships were stronger for the task climate than for the ego climate. The finding that elite athlete correlations appeared dampened is important for future research. Even with the damped relationships, practitioners, from the Olympics to local clubs, should ensure the promotion of the task climate to maximize positive affect and satisfactions in and around the sport experience.

216	Examining the Impact of Coach Leadership Styles on Athlete Motivation in Collegiate Athletics.	Awan, Shah Hussain ; Khan, Awais Alam ; Zulkifal, Syed ; Ullah, Abrar	International Journal of Social Science Archives (IJSSA)	2024		S3	SPORTDiscus	This research delves into the complex interplay of the leadership styles of the college athletic coaches and players' motivation. Athletes' intrinsic drive is critical for peak performance in the fast-paced world of collegiate athletics however, gaps exist in the literature regarding the interaction of leadership styles of the coach and athletes' motivation. To address this gap, this research examines the effects of democratic, autocratic, transformational, and transactional leadership styles on players motivation. Using cross sectional design, data was obtained from athletes of the sports department of a university through questionnaires. Analysis suggest that, there is a positive association between transformational leadership and the motivation of athletes, but a negative association between transactional leadership and athletes' motivation. Leadership styles, whether autocratic or democratic, have different effects on what drives athletes. This research helps in understanding the leadership dynamics in the collegiate sports which should help coaches, administrators, and researchers find ways to motivate and inspire their athletes to achieve at their best. Further longitudinal study and balanced leadership styles are suggested.
217	The Gold Medal Profile for Sport Psychology (GMP-SP)	Durand-Bush, N; Baker, J; Van den Berg, F; Richard, V; Bloom, GA	JOURNAL OF APPLIED SPORT PSYCHOLOGY	2023	10.1080/10413200.2022.2055224	S1	WoS	The Gold Medal Profile for Sport Psychology (GMP-SP) is a comprehensive, evidence-informed framework integrating mental performance competencies underpinning the athletic performances of Canadian athletes capable of stepping onto the Para/Olympic Podium. The GMP-SP was established to guide Mental Performance Consultants (MPCs) and National Sport Organizations (NSOs) in their design, delivery, tracking, and evaluation of mental skills programs in the Canadian high performance sport system. A Participatory Action Research approach involving a 2-year cyclical process of planning, action, reflection, and evaluation informed the collaborative work of six experienced Canadian MPCs (four men, two women). The group, whose lived experience was integral to the relevance and impact of the inquiry collectively had over 125 years of experience conducting research and consulting in high performance sport. A review of the scientific literature combined with the experts' professional practice led to the creation of the GMP-SP, which includes 11 mental performance competencies grouped under three broad categories: (a) fundamental competencies (motivation, confidence, resilience), (b) self-regulation competencies (self-awareness, stress management, emotion, and arousal regulation, attentional control), and (c) interpersonal competencies (athlete-coach relationship, leadership, teamwork, communication). Mental health was also included as an overarching construct influencing the achievement of mental and athletic performance. The GMP-SP fulfills an important gap given the current lack of models, methods, and tools to guide the operationalization of mental performance systems in high performance sport that include both intrapersonal and interpersonal competencies. Lay summary: The Gold Medal Profile for Sport Psychology (GMP-SP) is a framework integrating 11 mental performance competencies underpinning podium success in Canadian high performance sport, with attention to mental health. The GMP-SP is intended to guide practitioners and sport leaders in their sport psychology programming and resource allocation to support athletes in their quest for excellence.IMPLICATIONS FOR PRACTICE Mental Performance Consultants (MPCs) can use the Gold Medal Profile for Sport Psychology (GMP-SP) for the assessment, periodization, and implementation of mental skills training programs in high performance sport. The GMP-SP can be used to educate athletes, coaches, and staff about the importance of mental performance skills to achieve success
218	THE COACH-ATHLETE RELATIONSHIP: PSYCHOMETRIC PROPERTIES OF THE SPANISH VERSION OF THE CART-Q	Sanz-Fernández, C; Alonso-Arbiol, I; Pérez-Albéniz, A	REVISTA INTERNACIONAL DE MEDICINA Y CIENCIAS DE LA ACTIVIDAD FISICA Y DEL DEPORTE	2023	10.15366/rimcafd2022.89.013	S1	WoS	The aims of the present study were to adapt into Spanish and examine the construct validity of the Coach-Sport Relationship Questionnaire (CART-Q). A total of 162 sports(wo)men (43.2% women), players of differente sports, were between 16 and 59 years old (M = 26.41, SD = 8.98), made up the sample. A confirmatory factorial analysis, revealed a trifactorial structure. Indexes of goodness-of-fit (GFI: .951; CFI: .890; TLI: .952 and error (RMSEA: .078; RMR: .067) were adequate. Regarding gender and level differences, professional sportswomen were significantly closer to their coaches than the rest of the participants. Results support the use of the instrument in the context of Spanish-speaking sport relationships. Gender and level differences need to be taken into account for the improvement of athletes' relational satisfaction.
219	Can't jump, won't jump: Affordances of the horse-rider dyad underpin skill adaptation in showjumping using a constraints-led approach	Davies, M; Stone, JA; Davids, K; Williams, J; O'Sullivan, M	INTERNATIONAL JOURNAL OF SPORTS SCIENCE & COACHING	2023	10.1177/17479541221107379	S1	WoS	Equestrianism is part of a global industry influenced by a rich history of over 4000 years of culture and tradition. As the only interspecies Olympic event, equestrianism is facing negative public perceptions of competition performance and traditional coaching practices. In this position paper, we propose a constraints-led approach as a framework for contemporising equestrian coaching practice. Ecological dynamics is the theoretical framework that underpins a constraints-led approach methodology, providing guiding principles that inform a nonlinear pedagogy in sport and physical education. A constraints-led approach focuses on the individual (organism/s), task and environmental constraints acting over multiple nested timescales and what this means for how behaviour emerges. Using examples from the equestrian discipline of showjumping, we outline how a constraints-led approach can inform coaching behaviour and practice design to support skill acquisition through co-adaptations in the horse-rider dyad system. By focussing on the horse-rider dyad as a complex system, there is a move away from a human-centric perspective of compliance and control of the horse, toward system agency and intentionality in problem solving. Practice design principles of intention, representativeness, constraints manipulation and functional variability support the dyad to co-adapt and interact effectively through practice to achieve performance goals. Skilful performance is developed through attunement to perceptual information that invites opportunities for action (affordances). Understanding the development of affordance perception in the horse-rider dyad could guide the application of a constraints-led approach to equestrian coaching practice.

220	A Qualitative Exploration of Coaches' Perceptions of Performance Under Pressure in Sport	Hufton, JR; Vella, SA; Schweickle, MJ	SPORT EXERCISE AND PERFORMANCE PSYCHOLOGY	2023	10.1037/spy0000324	S2+S3	WoS	The concept of clutch performance (improved or successful performance under pressure) has gained traction in both research and the media in recent years, with notable, high-profile sporting moments occurring more frequently. As athletes inherently experience pressure when competing in sporting events, researchers and sport practitioners aim to work with athletes to help facilitate performance under these pressure circumstances. While research in the field has aimed to explore how clutch performances occur in sport, there remains some conceptual confusion regarding the definition of clutch performance itself. In light of this, 12 semielite/elite sporting coaches took part in a career-based, semistructured interview, to offer insight into when, and how, clutch performances occur. Interviews were recorded and transcribed verbatim, and data were analyzed using reflexive thematic analysis (Braun & Clarke, 2006, 2019). Two themes were generated: (a) the performance level required for clutch performance is context-dependent and (b) clutch performances may occur at multiple temporal levels. These themes suggest that the performance requirements to achieve a clutch performance vary depending on the context of the event, and further, clutch performances were considered to occur as either specific clutch moments throughout an event or as an extended performance over the course of an entire event. These findings provide support for a refined definition of clutch performance, wherein the context of the situation and duration of the performance are considered. Implications are discussed, and recommendations are made for future research.
221	Narcissistic coaches and athletes' individual rowing performance	Nevicka, B; van Gerven, EJG; Sedikides, C	SCIENTIFIC REPORTS	2023	10.1038/s41598-023-48239-6	S2	Scopus+WoS	Narcissism, a personality trait marked by an excessively self-aggrandizing, entitled, and dominant orientation, has been associated with high performance under competitive pressure, as these contexts afford the opportunity to self-enhance. Narcissism is often characteristic of organizational and political leaders, yet little is known about narcissism in sports coaches. We propose that in a competitive context narcissistic coaches could inspire and motivate their athletes to raise their performance. We investigated the association between coach narcissism and athletes' performance, and the role of athletes' perceived self-enhancement opportunity as a potential mediating mechanism. We examined coach narcissism, athletes' individual end times (i.e., performance), and athletes' perceptions of self-enhancement opportunity during annual national indoor rowing competitions in 266 national level competitive rowers from 52 rowing clubs. Results of multilevel analyses showed that coach narcissism positively predicted athlete performance, and this was explained by athletes' perceived opportunity to self-enhance during the competition. Thus, narcissistic coaches seem to reinforce athletes' perceptions that competition provides them with an opportunity to show off their skills, which in turn accounts for athletes' better performance in comparison to athletes who do not train with narcissistic coaches. The findings point to a potentially functional side of narcissism in coaching.
222	A randomized controlled study of mindful sport performance enhancement and psychological skills training with collegiate track and field athletes	Hut, M; Minkler, TO; Glass, CR; Weppner, CH; Thomas, HM; Flannery, CB	JOURNAL OF APPLIED SPORT PSYCHOLOGY	2023	10.1080/10413200.2021.1989521	S2	Scopus+WoS	Although both traditional cognitive behavioral psychological skills training (PST) interventions and mindfulness-based alternatives can lead to improvements in sport performance and athlete well-being, recent reviews and meta-analyses support the need for more outcome research with randomized designs. The present randomized controlled trial with members of an NCAA Division III Track and Field team was the first to compare a 6-week Mindful Sport Performance Enhancement (MSPE; K. A. Kaufman et al., 2018) training to a PST active control group, using a mixed-method approach with both quantitative and qualitative assessment and a train-the-trainer model of intervention delivery. The 24 student-athletes who completed post-test measures showed significant improvements in sport anxiety, and reported greater sport enjoyment and less influence of negative internal states on current sport performance; the MSPE group reported greater improvements in satisfaction with sport performance compared to PST. Qualitative responses suggested that both programs were helpful both for sport and everyday life, while some differential effects for interventions were also noted. The program evaluation measure additionally revealed a moderate degree of program acceptability in both groups, and the trainings were seen as moderately to very helpful for sport (e.g., being in the zone, less anxious, more focused). Although future research is needed with larger sample sizes and interview methods, results suggest that both interventions may be appropriate for sport psychology consultants to utilize and provide further evidence for a train-the-trainer approach for intervention delivery. IMPLICATIONS FOR PRACTICE Based on both quantitative and qualitative data analysis, results suggest that both psychological skills training (PST) and mindfulness-based interventions (MBIs) may positively influence factors related to sport experience and contribute to enhanced well-being, including anxiety reduction, attention, and emotion regulation. Moreover, both interventions included exercises that participants enjoyed, providing support for the acceptability of both programs. Short-term MBIs may contribute to greater satisfaction with sport performance and improve attention (present-moment focus and awareness) in both sport and everyday life, while PST may lead to more improvements in mindset during sport. Those who wish to see changes in these specific areas might want to consider these interventions. Coaches may be able to provide sport psychology services feasibly

223	Moderators of the coach leadership and athlete motivation relationship	de Subijana, CL; Martin, LJ; McGuire, CS; Côté, J	EUROPEAN JOURNAL OF SPORT SCIENCE	2023	10.1080/17461391.2022.2041101	S3	SPORTDiscus+WoS	The purpose of this study was to determine whether the relationship between coach leadership and athlete motivation was moderated by age, gender, competition level, and seasons spent with a coach. This study involved data from two previous studies that explored this relationship yet provides a novel perspective through the lens of important moderators. Three-hundred and three athletes (M-age = 17.6 years; SD = 3.20; 49.7% women and 50.3% men) responded to questionnaires pertaining to their coaches' leadership behaviours and their own sport motivation. Multiple regression analyses using moderators were conducted. Age, competition level, and seasons spent with the coach significantly moderated the relationships of interest. Coach transformational leadership predicted intrinsic and extrinsic motivation to a greater extent when athletes were younger than 20.8 and 18.2 years of age, respectively. Further, coach transactional leadership predicted intrinsic and extrinsic motivation to a greater extent when athletes had trained for more than two seasons with their coach. Results emphasize the need to consider athlete characteristics from both research and practitioner perspectives. Herein, we advocate for increased awareness amongst key sport stakeholders on the influence that a coach can have on younger athletes' motivation and the importance of developing coach-athlete relationships over time.
224	A longitudinal investigation of the role of perceived autonomy support from coaches in reducing athletes' experiential avoidance: The mediating role of subjective vitality	Wang, Y; Wu, CH; Chen, LH	PSYCHOLOGY OF SPORT AND EXERCISE	2023	10.1016/j.psychsport.2022.102304	S3	Scopus+WoS	Experiential avoidance, a personality trait that refers to individuals' tendency to avoid negative experiences, can have a negative impact on athletes' goal achievement. For this reason, it is crucial to identify the factors that can mitigate such a tendency. Drawing on self-determination theory and referring specifically to the function of subjective vitality, we first hypothesize that perceived autonomy support from coaches is positively associated with athletes' subjective vitality, which in turn is negatively associated with athletes' experiential avoidance. Data were collected from one hundred eighty-five high school athletes in Taiwan using a three-wave, time-lagged survey design spanning a period of seven months. These athletes were drawn from ten senior high schools and were in their second year of high school. The results of regression analysis showed that perceived autonomy support from coaches at Time 1 was associated with higher vitality among athletes at Time 2, which was, in turn, associated with lower levels of experiential avoidance at Time 3, conditional on the athletes' experiential avoidance at Time 2. While perceived autonomy support from coaches at Time 1 was also associated with lower experiential avoidance at Time 2, experiential avoidance at Time 2 was not associated with vitality at Time 3 after controlling for vitality at Time 2. The results of mediation analysis further supported the claim that vitality is a critical mediator of the relationship between perceived autonomy support from coaches and athletes' experiential avoidance. Implications concerning the identification of this mediator are discussed.
225	The mediator role of communication skill in the relationship between empathy, team cohesion, and competition performance in curlers	Bedir, D; Agduman, F; Bedir, F; Erhan, SE	FRONTIERS IN PSYCHOLOGY	2023	10.3389/fpsyg.2023.1115402	S3	WoS	Understanding the psycho-social factors such as communication, empathy, cohesion, etc., that affect successful athletic performance is a high priority and primary focus for applied sports psychology. Detailed examination of the athletes' psycho-social characteristics is essential in revealing which processes play an active role in achieving optimum performance. Developing these features of the athlete can contribute to coordinating the team, sharing tasks, increasing motivation, preparing team members for a change, and improving performance. For this purpose, the mediating role of communication skills in the relationship between empathy, team cohesion, and competition performance was examined in a sample of 241 curlers competing in 69 teams in the Turkish Curling League in the 2021-2022 season. During the data collection process, Personal Information Form, Empathic Tendency Scale, Scale for Effective Communication in Team Sport, and Group Environment Questionnaire were used. Competition performance was calculated by giving 1 point for each match won by the teams in the competitions in which the single-circuit round-robin system is applied. Structural equation modeling was used in data analysis to determine the direct and indirect predictive effects between variables. The study showed that empathy and team cohesion predict competition performance through communication skills, and communication skills fully mediate this relationship. Based on the research results, it was evaluated that communication skills have a substantial effect on the competitive performance of athletes, and this finding was discussed in the context of the literature.
226	Application of a nutrition support protocol to encourage optimisation of nutrient intake in provincial academy rugby union athletes in New Zealand: Practical considerations and challenges from a team-based case study	Roberts, CJ; Gill, ND; Beaven, CM; Posthumus, LR; Sims, ST	INTERNATIONAL JOURNAL OF SPORTS SCIENCE & COACHING	2023	10.1177/17479541221124119	S3	WoS	Provincial academies represent an important bridge between amateur and professional level rugby union in New Zealand. Athletes are provided with professional-level coaching; however, limited direct nutrition support is available. Congested training schedules and the requirement to work or study due to a lack of financial support may present a challenge towards athletes meeting nutrition requirements. The aim of the study was to facilitate improvement in nutrient intake, body composition and subjective well-being in provincial academy athletes via the implementation of a nutrition-support protocol based around behaviour change techniques. Significant increases in total energy (pre: 2492 +/- 762 kcal; post: 2614 +/- 625 kcal), relative energy (pre: 24.4 +/- 7.5 kcal center dot kg; 25.5 +/- 6.0 kcal center dot kg), total protein (pre: 131.1 +/- 41.8 g; 153.8 +/- 37.1 g) and relative protein (pre: 1.3 +/- 0.4 g center dot kg; post: 1.5 +/- 0.3 g center dot kg) were observed. Furthermore, changes in subjective sleep quality, stress, mood and upper body soreness were observed following the intervention. No changes were observed in body composition, carbohydrate or fat intake. Significant variability in nutrition and body composition changes highlights the importance of applying an individualised approach to nutrition support provision in developmental athletes. Practitioners working within these environments should be aware of the challenges and influences contributing to athletes' nutrition choices and habits.

227	Understanding the Role of Self-Efficacy in Sports Performance: A Longitudinal Study	Yang, XK; Mojtahe, K	REVISTA DE PSICOLOGIA DEL DEPORTE	2023		S3	WoS	The primary goal of this research project is to evaluate the influence of self-efficacy in athletic performance. This longitudinal study gives primary research data analysis to identify the research study employed useful research questions connected to self-efficacy and sports performance. This question is dependent on whether it is open-ended or closed-ended. The research study employed SPSS software for this aim, and the outcomes were useful. The descriptive statistic analysis, one-way ANOVA test analysis, variance component analysis, chi-square analysis, and the initial main test between dependent and independent variables are all performed. Self-efficacy considers independent, and sports performance is the dependent variable. An athlete's self-efficacy beliefs are greatly influenced by their coaches, teammates, and the broader sports environment. Coaches, trainers, and sports psychologists can improve performance results by creating effective techniques to boost players' self-belief and confidence by better understanding the components that affect self-efficacy. The overall research study found that there are negative links between self-efficacy and sports performance.
228	Noncontact Injury Distribution and Relationship With Preseason Training Load and Nonmodifiable Risk Factors in Rugby Union Players Across Multiple Seasons	Evans, SL; Whittaker, G; Davis, OE; Jones, ES; Hardy, J; Owen, JA	JOURNAL OF STRENGTH AND CONDITIONING RESEARCH	2023	10.1519/JSC.0000000000004402	S3	WoS	Evans, SL, Whittaker, G, Elphinstone Davis, E, Jones, ES, Hardy, J, and Owen, JA. Noncontact injury distribution and relationship with preseason training load and non-modifiable risk factors in Rugby Union players across multiple seasons. J Strength Cond Res 37(7): 1456-1462, 2023-This study examined the distribution of noncontact injury during phases of the competitive season and the association between preseason training load (TL) and nonmodifiable risk factors on injury risk during these phases. Injury data were recorded from 1 senior academy team over 3 seasons (2017-2020) and analyzed across early-season, midseason, and late-season phases. A generalized estimating equation was used to model risk factors with noncontact injury for selected phases. The highest noncontact injury incidence occurred in the late-season phase (22.2 per 1,000 hours) compared with early (13.7 per 1,000 hours, $p < 0.001$) and midseason phases (15.5 per 1,000 hours, $p = 0.001$). Low preseason TL (8,949-12,589 arbitrary units; odds ratio [OR], 95% confidence interval [CI] = 4.7, 1.0-21.6; $p = 0.04$) and low preseason TL combined with high early-season TL and injury in the early-season phase (OR, 95% CI = 6.5, 1.1-35.5; $p = 0.03$) were associated with greater midseason noncontact injury risk. In addition, low preseason TL combined with previous injury was associated with increased risk of noncontact injury risk in the late season (OR, 95% CI = 12.2, 0.9-15.6, $p = 0.05$). Our results suggest players are at a greater injury risk during the late-season phase, with low preseason cumulative loads combined with a history of previous injury associated with increased in-season injury risk. Strength and conditioning coaches should therefore monitor cumulative preseason TL alongside screening for previous injury history to identify athletes at greater risk of noncontact injury risk during the competitive season.
229	Assessing the Use of Heart-Rate Monitoring for Competitive Swimmers	Sixsmith, H; Crowcroft, S; Slattery, K	INTERNATIONAL JOURNAL OF SPORTS PHYSIOLOGY AND PERFORMANCE	2023	10.1123/ijspp.2023-0009	S3	WoS	Purpose: Quantifying training intensity provides a comprehensive understanding of the training stimulus. Recent technological advances may have improved the feasibility of using heart-rate (HR) monitoring in swimming. However, the implementation of HR monitoring is yet to be assessed longitudinally in the daily training environment of swimmers. This study aimed to assess the implementation of HR by comparing the training-intensity distribution from an external measure, planned volume at set intensities (PVSI), with the internal training-intensity distribution measured using time in HR zones. Methods: Using a longitudinal observational design, 10 competitive swimmers (8 male and 2 female, age: 22.0 [2.3] y, Federation Internationale de Natation point score: 842.9 [58.5], mean [SD]) were monitored daily for 6 months. Each session, HR data, and coached-planned and athlete-reported session rating of perceived exertion (Modified Category Ratio 10 scale) were recorded. Based on previously determined training zones from an incremental step test, PVSI was calculated using the planned distance and planned intensity of each swim bout. Training-intensity distributions were analyzed using a linear mixed model (lme4). Results: The model revealed a small to moderate relationship between PVSI and time in HR zone, based on the Nakagawa R-squared value (range .14-.42). Conclusions: Training-intensity distribution differed between the internal measure (ie, HR) and the external measure of intensity (ie, PVSI). This demonstrates that internal and planned external measures of intensity cannot be used interchangeably to monitor training. Further research should explore how to best integrate these measures to better understand training in swimming.

230	Monitoring mood state to improve performance in soccer players: A brief review	Selmi, O; Ouergui, I; Muscella, A; Levitt, DE; Suzuki, K; Bouassida, A	FRONTIERS IN PSYCHOLOGY	2023	10.3389/fpsyg.2023.1095238	S3	WoS	IntroductionPsychological aspects of sport are key in maintaining athlete motivation and make a difference in competitive outcomes. Adjustments to training may be necessary according to athletes' emotional state. Therefore, it is important to assess and quantify mood states throughout the season in team sports, including among soccer players. The Profile of Mood States (POMS) is a widely used questionnaire that assesses emotional states characterized by positive or negative feelings and can be administered repeatedly to assess changes in mood state. This review aims to assess and summarize the current literature on mood state variation in soccer players with a specific focus on training loads, training modalities, and competitive performance. MethodsA literature search was systematically conducted and resulted in 156 records. After removing duplicates, items with irrelevant titles and abstracts were screened out, and full texts were then screened for relevance and compared with inclusion and exclusion criteria. The remaining 37 articles were included in the final qualitative synthesis. ResultsPOMS scores were related to variability in training load, intensity of the training period, modality of training exercises, competitive performance and time of day in soccer players. Common recommendations include monitoring the mood state of soccer players during training sessions, matches, and throughout training periods to detect early signs of psychological disturbance and aid in optimizing high-level training performance. ConclusionThe POMS allows for monitoring of players' psychological state, providing coaches with data to aid in adjusting acute program variables according to players' psychological states and improve performance. Results offer practical support for the use of a simple POMS measurement as part of an overall program to monitor the players' psychological states. Results also highlight how training choices (i.e., load and exercise modality) and competitive performance are related to mood states (i.e., tension, anger, confusion, depression, fatigue, and vigor).
231	Relationship Between Athlete-Reported Outcome Measures and Subsequent Match Performance in Team Sports: A Systematic Review	Sansone, P; Rago, V; Kellmann, M; Alcaraz, PE	JOURNAL OF STRENGTH AND CONDITIONING RESEARCH	2023	10.1519/JSC.0000000000004605	S3	WoS	Sansone, P, Rago, V, Kellmann, M, and Alcaraz, PE. Relationship between athlete-reported outcome measures and subsequent match performance in team sports: A systematic review. J Strength Cond Res 37(11): 2302-2313, 2023-Athlete-reported outcome measures (AROMs; e.g., fatigue, stress, readiness, recovery, and sleep quality) are commonly implemented in team sports to monitor the athlete status. However, the relationship between AROMs and match performance indicators is unclear and warrants further investigation. This systematic review examined the relationship between precompetitive AROMs and subsequent match performances of team sport athletes. Following the Preferred Reporting Items for Systematic Reviews and Meta-Analyses (PRISMA) guidelines, 3 (PubMed, Scopus, and Web of Science) databases were systematically searched to retrieve studies investigating the effects or association of AROMs and match: (a) technical-tactical performance (match-related statistics), (b) physical performance, (c) physiological and (d) perceptual demands, and (e) other measures of performance in adult team sport athletes. Quality assessment of included studies was performed using a modified Black and Downs checklist. Fifteen articles representing 289 team sport athletes were included. Mean quality of included studies was 7.6 +/- 1.0 (of 11). Across the included studies, 22 AROMs parameters were used, and 16 different statistical approaches were identified. Approximately 11 of 15 studies used nonvalidated AROMs. Overall, associations or effects of AROMs were found consistently for match-related statistics (7/9 studies), whereas results were unclear for physical performances (3/7 studies), perceptual demands (1/2 studies), or other measures of performance (2/4 studies). Considering the importance of key match-related statistics for success in team sports, this review suggests that monitoring precompetitive AROMs has potential to provide valuable information to coaches. However, it is indispensable to validate AROMs questionnaires and to uniform data collection and statistical procedures before substantiated indications to practitioners can be made.
232	Research on Optimization of Post-Competition Training Methods for Competitive Sports Athletes	Xia, CY; Lei, XM	REVISTA DE PSICOLOGIA DEL DEPORTE	2023		S3	WoS	Athletes enter a competitive sport for various reasons and go on to compete for years without any significant modifications that may affect the success of their sporting career. However, there is a cost to this approach of no changes in training strategy. The purpose of this study was to identify successful post-competition training methods by studying both international and Olympic athletes compete in multiple endurance sports during the World Championships event. Results showed several factors including personal characteristics, coaching knowledge, skill level, competition phase and season can have a significant impact on the success rates of athletes following competition performance. In this study, five factors were employed, and the results were published. The information was gathered using a questionnaire from 50 kids who participated in sports. However, the gathered data was subjected to SEM PLS 3 analysis. The findings revealed a favorable relationship between the five variables that were studied.

233	Precompetition Strategies to Improve Performance in Endurance or Repeated Intermittent Activity: Evidence and Practical Suggestions	Valcarce-Merayo, E; Latella, C	STRENGTH AND CONDITIONING JOURNAL	2023	10.1519/SSC.0000000000000753	S3	WoS	Athletes engage in training programs that develop physical and physiological attributes to improve performance over time. However, ensuring athletes are optimally prepared just before competition is also of utmost importance. Indeed, such approaches may often adopt 1 or more strategies in the days, hours, and minutes preceding competition, including exercise priming, active warm-up, and/or postactivation performance-enhancing (PAPE) activities. Despite a plethora of discrete research, such information seems poorly synthesized, making it difficult for coaches and athletes to implement optimal evidence-based physical preparation approaches in many sporting contexts. This article aims to provide a scoping overview of evidence, facilitate discussion, and where possible, propose practical exercise-based recommendations that may be useful leading up to (& SIM;48 hours-& SIM;1 minute) tasks with a short or prolonged endurance component or requiring repeated intermittent efforts (e.g., team-based field sports). Well-timed and -designed activities can facilitate several aspects of physical performance, potentially improve athletes' psychological state(s) and mental preparedness. The benefits of a warm-up are most well-documented, but priming and PAPE strategies also warrant consideration. It is intended that the evidence and suggestions presented will be useful for strength and conditioning professionals preparing athletes for competition.
234	Using Physiological Laboratory Tests and Neuromuscular Functions to Predict Extreme Ultratriathlon Performance	Abad, CCC; Carmo, EC; Do Prado, DML; Reis, V; Pereira, L; Loturco, I; Koehle, M	RESEARCH QUARTERLY FOR EXERCISE AND SPORT	2023	10.1080/02701367.2022.2094307	S3	WoS	Purpose: This study aims to investigate the relationship between split disciplines and overall extreme ultra-triathlon (EUT) performance and verify the relationship among physiological and neuromuscular measurements with both fractional and total EUT performance while checking which variables could predict partial and overall EUT race time. Methods: Eleven volunteers (37 +/- 6 years; 176.9 +/- 6.1 cm; 77.9 +/- 10.9 kg) performed two maximal graded tests (cycling and running) for physiological measurements and muscle strength/power tests to assess neuromuscular functions. Results: The correlation of swimming split times to predict overall EUT race times was lower than for cycling and running split times (r(2) = 0.005; p > .05; r(2) = 0.949; p r(2) = 0.925; p < .001, respectively). VO2peak obtained during running test (VO2peakrun) and VT power output assessed during cycling test (VTPO) were the highest predictors of cycling performance (r(2) = 0.92; p = .017), whereas VO2peakrun and peakpower output in the cycling test (PPO) were the highest predictors of running performance (r(2) = 0.94; p = .008). Conclusion: VO2peakrun and VTPO, associated to jump height assessed during countermovement jump (CMJ) test were the highest correlated variables to predict total EUT performance (r(2) = 0.99; p = .007). In practical terms, coaches should include the assessment of VO2peakrun, VTPO, and CMJ to evaluate the athletes' status and monitor their performance throughout the season. Future studies should test how the improvement of these variables would affect EUT performance during official races.
235	The Psychology of Athletic Tapering in Sport: A Scoping Review	Stone, MJ; Knight, CJ; Hall, R; Shearer, C; Nicholas, R; Shearer, DA	SPORTS MEDICINE	2023	10.1007/s40279-022-01798-6	S3	WoS	Taper is a common training strategy used to reduce fatigue and enhance athletic performance. However, currently, no review has summarised what psychological research has been conducted examining taper, what this research shows and what future research needs to be undertaken to extend the field. Consequently, a scoping review was conducted with three aims: (a) to determine the characteristics of psychological research examining taper, (b) to summarise psychological research collected during taper with adult athletes and coaches, and (c) to identify gaps in psychological research examining taper. Forty-eight articles were identified following an exhaustive search strategy and charted following scoping review guidelines. Results showed most research was quantitative, used a longitudinal design, was conducted in swimming, triathlon, cycling or across multiple sports, and used a university-, regional- or national-level male athlete sample. Eight themes were developed to summarise the research: Mood, Perception of Effort, Perceived Fatigue and Wellness, Recovery-Stress, Taper as a Stressor, Stress Tolerance, Psychological Preparation and Cognitive Functioning. Additionally, four research recommendations were identified: (a) conducting exploratory research that examines the impact taper has on athletes' and coaches' competition preparation and stress experience, (b) asking more advanced psychological questions and conducting multi-disciplinary research, (c) including a more diverse participant sample in studies and (d) examining the impact of psychological interventions during taper. Overall, this scoping review has highlighted the limited research examining the psychology of taper and the need for focused research that asks more complex questions across diverse populations.

236	Secondary School Athletic Coaches' Perceptions and Knowledge of the Athletic Training Profession	Lacy, AMP; Stearns, RL; Eason, CM; Casa, DJ	JOURNAL OF ATHLETIC TRAINING	2023	10.4085/1062-6050-0369.21	S3	WoS	Context: Coaches play a role in streamlining care, espe-cially by directing student-athletes in need of further medical attention to the athletic trainer (AT). The AT-coach relationship holds great potential for incorporating collaborative care, and yet, little is known about coaches' perceptions and knowledge of ATs.Objective: To investigate coaches' perceptions of athletic training and their knowledge regarding the roles and responsi-bilities of ATs in secondary schools.Design: Concurrent mixed-methods study.Setting: Cross-sectional online questionnaire. Patients or Other Participants: Secondary school athletic coaches from 10 sports with the highest participation rates during the 2017-2018 season (n = 1097). Most respondents were male (n = 795, 72.4%), and their average age was 44.7 6 11.4 years. Main Outcome Measure(s): Participants completed a web -based questionnaire containing demographics and quantitative measures assessing their perceived value and knowledge of ATs, as well as open-ended questions. Descriptive statistics summarized the demographic data. Counts and percentage responses for quantitative measures were reported. Open-ended responses were analyzed using the general inductive approach.Results: Approximately 93% of respondents considered an AT a trusted source of medical information and a key member of the sports medicine team. Most respondents selected injury prevention (98.9%), first aid and wound care (97%), therapeutic interventions (89.9%), and emergency care (85.8%) as skills ATs are qualified to perform. Forty-six percent of respondents were willing to coach without an AT employed. Coaches trusted ATs as part of the athletic teamand as gatekeepers, referring student-athletes for advanced care when warranted. Regarding the AT role, coaches emphasized the treatment of minor injuries and the idea of coverage versus care.Conclusions: Secondary school coaches valued the ath-letic training profession and were knowledgeable regarding various roles and responsibilities ATs frequently perform. However, they may view ATs as luxuries instead of necessities, as evidenced by the fact that just under half of responding coaches were willing to coach without an AT employed at the school.
237	Understanding mental toughness at a Women?s Australian rules football club	Clark, JD; Mallett, CJ; Moyle, GM; Coulter, TJ	PSYCHOLOGY OF SPORT AND EXERCISE	2023	10.1016/j.psychsport.2023.102432	S3	Scopus+WoS	Scholars have argued that mental toughness is a term that represents hypermasculine ideals. This hyper -masculinity ideal could be considered relevant in the sport of Australian rules football, which has been played, at the professional level, by male athletes for the majority of its existence. Given the rising popularity of the Australian Football League Women's (AFLW), the present research sought to explore how the term mental toughness was understood at an AFLW club through a cultural sport psychology lens. Interviews were conducted with players (n = 4) and coaches (n = 6) from an AFLW club over the course of a competitive season. Data were transcribed verbatim and thematically analysed, with themes constructed to correspond with different levels of contextual influence. From this analysis, the club values and underlying assumptions, the social truths, and the role models/archetypes that provided a collective understanding of the term were identified. Mental toughness was defined by high-performance values of the men's game, which had implications for female athletes in this environment who are not afforded the same opportunities to fully embody these values. Mental toughness is positioned, in this environment, as an ideal with different meanings for female athletes due to structural factors associated with elite-level competition (e.g., full-time versus part-time professionalism).
238	The effects of coach autonomy support, sports mental training, experiential avoidance, and sports anxiety among badminton college athletes.	CHUAN-CHEN HUANG	Journal of Physical Education & Sport	2023	10.7752/jpes.2023.09289	S1	SPORTDiscus	Introduction: College athletes not only have to deal with their academic responsibilities but also undergo rigorous training processes and face the pressures of winning and losing in competitions. This is particularly challenging for badminton players, as the sport demands quick reaction times and involves varying speeds during play. Therefore, in addition to mastering the necessary skills, badminton players undergo significant psychological testing. Purpose: The purpose of this study is to investigate the relationships between coach autonomy support, sports mental training, experiential avoidance, and sports anxiety among badminton college athletes. Methods: A purposive sampling method was employed to distribute questionnaires to representative players from various college badminton teams in Taiwan. This study used the Learning Climate Questionnaire (LCQ) was used to measure the perception of coach autonomy support. The Sport Anxiety Scale (SAS) was employed to assess sports anxiety. The Acceptance and Action Questionnaire (AAQ) was utilized to gauge the level of experiential avoidance. Lastly, the Sport Mental Training Questionnaire (SMTQ) was used to evaluate sports mental skills. A total of 251 questionnaires were distributed, out of which 201 valid responses were received, resulting in a response rate of 80.08%. The collected data were processed using a linear structural equation model. Results: Coach autonomy support had a positive impact on sports mental training among badminton college athletes ($p < 0.05$). Conversely, sport mental training had a negative effect on experience avoidance and sports anxiety ($p< 0.05$). This implies that higher levels of coach autonomy support lead to better sports mental training, which, in turn, reduce the levels of experience avoidance and sports anxiety. Conclusion: Creating a positive sports atmosphere, nurturing the psychological skills of elite athletes, and mitigating the negative psychological impact during sports training is crucial. It is recommended to plan in place to enhance athletes' positive psychological skills and provide them with timely psychological care. Understanding the sources of pressure that athletes face beyond their daily training and competitions is crucial. Through psychological skills training, athletes can benefit more from these experiences and demonstrate superior performance in sports competitions.

239	Effect of boxers' social support on mental fatigue: Chain mediating effects of coach leadership behaviors and psychological resilience.	Zhang, Defa ; Lyu, Bei ; Wu, Jingtao ; Li, Wangze ; Zhang, Kaiyuan	Work	2023	10.3233/WOR-220478	S1	SPORTDiscus+Scopus	BACKGROUND: Athletic fatigue is an inescapable issue in competitive sports. It belongs to a physiological response that is triggered when competitive athletes are trained to a critical point. OBJECTIVE: The study aims to explore the relationships involving boxers' social support, mental fatigue, coach leadership behaviors and psychological resilience. METHODS: 1050 boxers were selected in several provinces across China and investigated on the basis of the Social Support Questionnaire for Athletes, Mental Fatigue Scale, Psychological Resilience Scale, and Leadership Scale for Sport. RESULTS: Boxers' social support was negatively correlated with mental fatigue and psychological resilience, while it was positively correlated with coach leadership behaviors. Apart from direct effects on mental fatigue, other impacts are imposed by boxers' social support via mediating effects such as coach leadership behaviors and psychological resilience. The total effect value was -0.18, the direct effect value was -0.08, and the indirect effect value was -0.12; furthermore, coach leadership behaviors and psychological resilience play a mediating role, accounting for 65.57% of the total. CONCLUSION: In order to alleviate the stress from intense competitive training and abate mental fatigue, competitive athletes may be encouraged in subsequent training to seek all-sided social support for social interpersonal relationships. While clarifying the mechanism how the external environment affects individuals, this paper explains the principle of social support on athletes' psychological fatigue and identifies mutual influences between coaches and athletes.
240	Features and Effects of Athlete Burnout Among Top Amateur Female Rugby Union Players	Martin, K; Hong, HJ	SPORT PSYCHOLOGIST	2022	10.1123/tsp.2022-0034	S1	WoS	This paper addresses the contributing factors and effects of athlete burnout in women's rugby. Current and former top amateur female rugby union players (N=10, age 19-21 yr) were interviewed regarding their experiences of high-performance rugby and of athlete burnout. Thematic analysis was applied to analyze the data. The findings show that seven players exhibited the three dimensions of burnout: emotional and physical exhaustion, reduced sense of accomplishment, and sport devaluation. Significant antecedents included role conflict, high physical demands, and coaching behaviors that resulted in maladaptive outcomes. The findings were in line with self-determination theory and showed consistencies with previous literature regarding athlete burnout. Meaningful and original findings related to the female rugby setting were also presented and discussed. Therefore, the study provides new insights into female rugby players' experience of athlete burnout and contributes to the understanding of athlete burnout in the context of women's rugby, which has been underresearched.
241	Relationships between coach's leadership, group cohesion, affective states, sport satisfaction and goal attainment in competitive settings	González-García, H; Martinent, G; Nicolas, M	INTERNATIONAL JOURNAL OF SPORTS SCIENCE & COACHING	2022	10.1177/17479541211053229	S1	WoS	We examined whether a perceived coach's leadership predicted group cohesion and whether group cohesion predicted intensity and direction of positive affect and negative affect experienced during competition, sport satisfaction and goal attainment following the completion of a sports competition. A sample of 296 competitive athletes (Mage = 21.61; standard deviation = 6.32) participated in the study and completed a series of self-report questionnaires assessing the aforementioned constructs. A partial least square path modelling approach was used to examine the relationships between the study variables. Results of the structural partial least square path modelling model revealed that: (a) democratic coach behaviour negatively predicted Group Integration Task, (b) authoritarian coach positively predicted Individual Attractions to the Group-Social, and (c) reward positively predicted Group Integration Task. Moreover, partial least square path modelling results also revealed that (a) Individual Attraction to the Group-Task positively predicted the direction of negative affect during competition, (b) authoritarian coach marginally positively predicted Group Social Integration during competition, and (c) Group Social Integration marginally negatively predicted the direction of Negative Affects during competition. Thus, contradicting previous studies, results did not provide evidence of the empowering effect of democratic coach leadership on Group Integration Task. Finally, results provided evidence for the significant relationships between group cohesion and athletes' competitive outcomes such as affective states experienced during competition.
242	Understanding Sport Expertise through a Cultural Perspective: An Exploratory Study	Castillo, EA; Lebeau, JC; Cho, S; Sáenz-Moncaleano, C; Chow, GM	INTERNATIONAL JOURNAL OF SPORT PSYCHOLOGY	2022	10.7352/IJSP.2021.52.099	S1	WoS	Cultural sport psychology has emerged as an important area within the field of applied sport psychology, but little research has been done on the cultural factors impacting sport expertise development. Employing Bronfenbrenner's ecological system theory as a guiding framework, the present study examined the development of sport expertise through a cultural lens. In-depth semi-structured interviews were conducted with ten elite athletes who had represented their respective countries in major international events. Our findings permeated three layers of Bronfenbrenner's ecological model of development: microsystem (family and coach-athlete relationship), mesosystem (sport culture), and macrosystem (country culture), and indicate that culture is a salient factor that influences the development of elite athletic performance. Culture was found to be a multifaceted phenomenon ranging from broad cultural layers such as values and norms within a country to more specific layers such as the sport culture within a family, the community atmosphere within the sport, and customs in the coach-athlete relationship. These findings contribute to the growing body of research in cultural sport psychology and enhance cross-cultural understandings of talent development in sport.

243	Body shaming and associated practices as abuse: athlete entourage as perpetrators of abuse	McMahon, J; McGannon, KR; Palmer, C	SPORT EDUCATION AND SOCIETY	2022	10.1080/13573322.2021.1890571	S1	WoS	In the 2016 International Olympic Committee Consensus Statement on harassment and abuse, it was outlined that psychological abuse in sport research has been heavily focused on the coach-athlete relationship resulting in a lack of research on other members of the athletes' support system such as their 'entourage.' Researchers of abuse have further noted that psychological abuse remains relatively underexplored in comparison to other types of athlete abuses (e.g. sexual abuse). As psychological abuse is one of the most common types of abuse occurring in sporting contexts, it has been flagged as an urgent safeguarding concern. Psychological abuse can be enacted in different ways with many associated behaviours. The present study explored one under-researched issue shown to be entrenched in sport culture - 'body shaming' - and how it constitutes psychological abuse. We also focused on the role of the athlete entourage (i.e. people associated with the athlete) in relation to psychological abuse through the body shaming of athletes. Using thematic analysis, three female athletes' stories showed how they were subjected to psychological abuse from members of their entourage when their bodies failed to meet socio-cultural expectations (i.e. too fat, not 'slim to win'). While it was not the central focus of our research, the athletes also explained how they were subjected to physical abuse and physical neglect from entourage members when they were perceived to be overweight or too fat. The athlete entourage members found to be perpetrators of abuse and physical neglect included the coach, the parent, the partner, and the manager. This research provides novel insight into how abuse is circulating through sporting contexts, and in so doing, generates knowledge for prevention and intervention initiatives in sport.
244	Perceived motivational climate influences athletes' emotion regulation strategies, emotions, and psychobiosocial experiences	Robazza, C; Moran, M; Bortoli, L; Ruiz, MC	PSYCHOLOGY OF SPORT AND EXERCISE	2022	10.1016/j.psychsport.2021.102110	S1	Scopus+WoS	The purpose of the study was to investigate whether athletes' perceptions about the motivational climate created by their coach influence emotion regulation strategies (i.e., cognitive reappraisal and expressive suppression), emotions, and psychobiosocial experiences of athletes. A sample of 459 competitive athletes (201 women, 258 men), aged 16-35 years, drawn from individual and team sports, completed self-assessment measures of perceived motivational climate, emotion regulation, sport emotions, and psychobiosocial experiences. Main results from structural equation modeling showed that perceived mastery climate was positively related to cognitive reappraisal, pleasant emotions, and psychobiosocial experiences, while perceived performance climate was positively related to expressive suppression and unpleasant emotions. Moreover, mediation analysis showed perceived mastery climate to have positive indirect effects on pleasant emotions and psychobiosocial experiences via cognitive reappraisal, while performance climate had indirect effects on unpleasant emotions via expressive suppression. Overall findings suggest that the type of motivational climate created by the coach has consequences in terms of athletes' emotion regulation strategies, emotions, and psychobiosocial experiences.
245	Don't think, just shoot - The paradox of shooting three-point shots in basketball	Lidor, R; Lipshits, L; Arnon, M; Bar-Eli, M	INTERNATIONAL JOURNAL OF SPORT AND EXERCISE PSYCHOLOGY	2022	10.1080/1612197X.2021.1987957	S2	WoS	A two-phase study was conducted on shooting three-point shots in basketball in pressure/no-pressure game situations. In Phase 1 - a subjective approach, our aim was to discover what the players and coaches thought about shooting beyond the three-point arc when the shooters were performing under defensive pressure or when they were free of such pressure. In Phase 2 - an analysis of shooting success, we examined the actual success (i.e., percentage of successful shots) of shooting three-point shots under these two situations. In Phase 1, 97 Division 1 male basketball players and 12 elite coaches were asked how they perceived shooting three-point shots in various game situations. In Phase 2, the success of 382 three-point shots taken in actual Division 1 games was analyzed. The shots were classified by four expert coaches into two categories - shots taken in free-of-defense and shots taken in under-pressure game situations. The success of the two classified shots was analyzed under 10 conditions. Expected results were found in Phase 1 - both players and coaches believed that the success of three-point shots is higher when the shots are taken when the shooter is free of defense. In Phase 2, a surprising finding was revealed: shooting success was higher when the players shot under defensive pressure. We discuss the data in line with Kahneman's (2011), Thinking, fast and slow, model, and propose a number of practical implications for coaches who are preparing their three-point shooters to deal with real game situations.

246	Upper-Body Post-activation Performance Enhancement for Athletic Performance: A Systematic Review with Meta-analysis and Recommendations for Future Research	Finlay, MJ; Bridge, CA; Greig, M; Page, RM	SPORTS MEDICINE	2022	10.1007/s40279-021-01598-4	S2	WoS	Background Research on post-activation performance enhancement (PAPE) is dominated by lower-body conditioning activities/performance test complexes. Despite the contribution of the upper body to many sporting actions, no review on upperbody PAPE currently exists. Objectives The aim of this systematic review with meta-analysis was to provide a synthesis of the available research on the inclusion of upper-body PAPE conditioning activities to improve athletic performance. Methods A review of the literature was conducted according to the Preferred Reporting Items for Systematic Review and Meta-analyses guidelines, including a literature search of EBSCOhost, SPORTDiscus, PubMed and Google Scholar databases. A total of 127 studies were identified through database searches, and were assessed against the following criteria: (1) randomised controlled trial or pre-and-post study design; (2) studies explored the effects of prior voluntary muscle activity, and not electrically induced contractions, (3) evidence, or lack thereof, of PAPE was quantified by the monitoring of individual performance to commonly applied physical tests or sport-specific tasks; (4) conditioning activities and performance tests were primarily upper-body; (5) detailed description of a standardised warm-up; and (6) full-text versions of studies could be accessed in English language peer-reviewed journals. Studies were quality assessed for methodological quality via the PEDro scale and ranked accordingly. Results Thirty-one studies met the inclusion criteria. Studies were classified into different conditioning activity modes: bench press variations, sport-specific (modified implement throws, swing-specific, cable pulley, elastic resistance, combination) and bodyweight activity. Acute performance enhancement in several movement-specific combinations was found. A metaanalysis revealed that bench press at >= 80% one repetition maximum significantly (p = 0.03; ES = 0.31) improves subsequent power output in the ballistic bench throw at 30-40% one repetition maximum, following 8-12 min recovery. Additionally, sport-specific overweight implement throws improved subsequent throwing distance at competition weight by -1.7-8.5%; ES = 0.14-0.33, following 3 min recovery. Sport-specific lighter weighted bat swings and swing-specific isometrics resulted in improved subsequent competition weight bat swing velocities, ranging from -1.3-3.3%; ES =0.16-0.57. Conclusions This review presents several upper-body movement-specific conditioning activities that could be considered by coaches and
247	Autonomy Support in Sport Contexts: The Role of Parents and Coaches in the Promotion of Athlete Well-Being and Performance	Lemelin, E; Verner-Filion, J; Carpentier, J; Carbonneau, N; Mageau, GA	SPORT EXERCISE AND PERFORMANCE PSYCHOLOGY	2022	10.1037/spy0000287	S3	WoS	The purpose of the present research was to investigate the independent and joint role of parent and coach autonomy support in the prediction of athlete well-being and performance. Two studies were conducted: Study 1 used a cross-sectional design and targeted athletes from different sports and competitive levels, while Study 2 was based on longitudinal data and targeted youth elite soccer players. Results from both studies showed that parent and coach autonomy support have additive relations with athlete well-being, but that only coach autonomy support is associated with sport performance. This research suggests that parent and coach play an important but distinct role in athlete well-being and that coach autonomy support could help athletes reach high levels of performance.
248	Unlocking the power of 'us': Longitudinal evidence that identity leadership predicts team functioning and athlete well-being	Fransen, K; Boen, F; Haslam, SA; McLaren, CD; Mertens, N; Steffens, NK; Bruner, MW	JOURNAL OF SPORTS SCIENCES	2022	10.1080/02640414.2023.2193005	S3	WoS	The social identity approach has become an important framework for understanding effective leadership. The present study is the first to longitudinally examine the relative impact of coaches' and athlete leaders' identity leadership on athletes' identification with their team, as well as the subsequent relationships with key team and individual outcomes. To investigate these research questions, 18 sport teams (N = 279) completed a questionnaire early and late in their season competition. To analyse these data, we conducted structural equation modelling and controlled both for baseline values and the nested structure of our data. Results revealed that it was mainly the identity leadership of athlete leaders (and not of the coach) early in the season that predicted athletes' team identification later in the season. This increased team identification in turn fed into both team outcomes (i.e., task climate, team resilience, team performance) and individual outcomes (i.e., well-being, burnout, and individual performance). The mediating role of team identification suggests that by building a shared sense of 'we', athlete leaders can improve the team's effectiveness and enhance athletes' well-being. Accordingly, we conclude that empowering athlete leaders and strengthening their identity leadership skills is an important way to unlock sport teams' full potential.
249	Six-month stability of individual differences in sports coaches' burnout, self-compassion and social support	Ackeret, N; Röthlin, P; Allemand, M; Krieger, T; Berger, T; Znoj, H; Kenttä, G; Birrer, D; Horvath, S	PSYCHOLOGY OF SPORT AND EXERCISE	2022	10.1016/j.psychsport.2022.102207	S3	WoS	Using a three-wave prospective cross-lagged panel design, the study examined six-month stability of burnout, self-compassion and social support among sports coaches in terms of measurement invariance, mean-level change, rank-order stability, and structural stability. The participating coaches (N = 422; M-age = 44.48, SD = 11.03) completed an online questionnaire measuring self-compassion, social support, coach burnout and demographics at baseline and two follow-ups at three months and six months. The various forms of stability were assessed using structural equation modeling. There was no significant mean-level change in burnout, self-compassion, or social support, and all three constructs exhibited measurement invariance. Rank-order stability remained relatively high, ranging from 0.78 to 0.94 across the three time points. For all three constructs, covariances between latent factors were invariant over time, indicating high structural stability. While self-compassion and social support were positively related, both were negatively related to coach burnout. These results confirm the importance of preventing and addressing symptoms of burnout, low self-compassion and poor social support in sports settings.

250	A Prospective Study of Teammate Factors on Athletes' Well-Being, Disordered Eating, and Compulsive Exercise	Scott, CL; Haycraft, E; Plateau, CR	SPORT EXERCISE AND PERFORMANCE PSYCHOLOGY	2022	10.1037/spy0000293	S3	WoS	Cross-sectional research has demonstrated the strong influence teammates have on athletes' eating attitudes/behaviors, but less is known about the enduring nature of such influence or the stability of eating and exercise psychopathology over time. This study aimed to (a) examine whether eating and exercise psychopathology and psychological well-being (anxiety, depression, self-esteem) in athletes remain stable over time, (b) examine which teammate factors predict athletes' eating and exercise attitudes/behaviors longitudinally, and (c) explore whether such predictive relationships differed as a function of gender/sport type/age group. Athletes (N = 311, mean age 18.74 years, n = 171 female, n = 134 lean sport athletes) completed a survey at the beginning (T1) and middle (T2; 4 months later) of their athletic season exploring teammate factors, psychological well-being, and eating/exercise psychopathology. Wilcoxon T-Tests assessed stability in variables over time (Aim 1), structural equation modeling tested the cross-lagged relationships among variables (Aim 2), and tests of invariance explored group differences (Aim 3). Levels of eating psychopathology significantly decreased from T1-T2 while levels of anxiety significantly increased. Notably, the cross-lagged model found higher levels of self-esteem at T1 predicted lower T2 bulimia modeling, and higher levels of bulimia modeling at T1 predicted higher T2 disordered eating. Males, those participating in nonlean sports and adolescent athletes are at increased risk of modeling disordered eating. Given the importance of understanding factors that can increase/reduce athletes' susceptibility to teammate influence, as well as the negative impact of teammate influence, these findings will inform the targeted development of team-based eating and exercise psychopathology prevention strategies.
251	A longitudinal study on the psychological and physiological predictors of burnout in NCAA collegiate swimmers	Martin, J; Byrd, B; Hew-Butler, T; Moore, EWG	JOURNAL OF APPLIED SPORT PSYCHOLOGY	2022	10.1080/10413200.2021.1974603	S3	WoS	The purpose of the current study was to examine the ability of psychosocial constructs and biomarker measures of overtraining and stress to predict athlete burnout, as mediated by training distress, during a 6-week intense training period. Twenty-three females and 17 male NCAA swimmers completed measures, before training (baseline), of athlete identity, academic identity, mental toughness, pain attitude, and controlling coaching behaviors. Training distress was assessed at 3 weeks. Serum measurement of creatine kinase, myoglobin, testosterone, and cortisol were obtained at baseline and after 6 weeks of intense training. Athlete burnout was also measured at 6 weeks. The full mediation model was non-significant, F(9,30) = 1.23, p = .32. However, the direct effects only model was significant, F(10, 29) = 5.22, p = .001, and accounted for 64% of the variance in burnout symptoms. Controlling coaching behaviors, athletic identity, academic identity, and testosterone/cortisol ratio change were all significant predictors. Our findings provide support for the value of an athletic identity, the deleterious effects of controlling coaches, and the detrimental effects of an academic identity (when narrowly considered in terms of burnout symptoms). The ability of biomarkers of muscle damage as indicators of perceptions of burnout symptoms is questionable. Lay summary: Burnout in swimming is characterized by mental and physical exhaustion, a devaluation of swimming, and successes often becomes less meaningful. Swimmers who train hard, resulting in soreness and fatigue, may not experience such a state as stressful or indicative of burnout. However, controlling coaches and athletes who strongly value academic success may be prone to experiencing burnout symptoms. Strong athletic identities may protect swimmers from burnout symptoms.IMPLICATIONS FOR PRACTICE Coaches should allow their swimmers input into their training. Coaches should be aware that academic demands may contribute to burnout. Coaches should be aware that swimmers with weak athletic identities may be susceptible to burnout.
252	Performance Profiling in Strength and Conditioning	Johnson, DL; Bird, MD	STRENGTH AND CONDITIONING JOURNAL	2022	10.1519/SSC.0000000000000684	S3	WoS	Performance profiling is a client-led assessment tool that enables athletes to uncover strengths and weaknesses associated with their sporting performance. Initially designed to increase client commitment to a training program, the performance profiling technique presents an opportunity for the athlete to play an active role and contribute to the initial needs analysis process. This evidenced-based method of assessment has been shown to help clients increase their self-awareness, identify areas they can improve, and serve as a basis for goal setting. The tool also permits both client and practitioner to monitor and track progress over time. Although predominantly adopted for those providing psychological support to athletes, the performance profile may be beneficial for strength and conditioning coaches. This article aims to highlight the potential benefits of performance profiling within the field of strength and conditioning, provides guidelines with completed examples for strength and conditioning practitioners to integrate into their work with clients, and presents a brief overview of the theory underpinning the technique.

253	Athletes' motivational and emotional outcomes related to a Need-Supportive intervention in intensive training centers	Cece, V; Guillet-Descas, E; Tessier, D; Martinent, G	JOURNAL OF APPLIED SPORT PSYCHOLOGY	2022	10.1080/10413200.2021.1941425	S3	Scopus+WoS	The aim of this study was to set up an intervention program promoting a need-supportive style for elite coaches, and to evaluate its effects on athletes' emotional and motivational outcomes during the competitive season. Seven coaches of 16 elite table tennis players from two intensive training centers received a need-supportive intervention, comprising an initial theoretical session and video analyses of coaching sessions. The athletes reported their self-determined motivation (i.e., intrinsic motivation, integrated regulation, identified regulation, introjected regulation, external regulation, and amotivation), basic psychological need satisfaction (BPNS, for autonomy, competence, and relatedness) and thwarting (BPNT, for autonomy, competence, and relatedness), and positive and negative emotions (i.e., anxiety, anger, excitement, dejection, and happiness) in a logbook twice a month during the intervention period. Multilevel growth curve analyses revealed a decrease in the BPNT levels for autonomy and competence and an increase in BPNS for autonomy. Moreover, the results also showed improved trajectories for emotional outcomes including anxiety, anger, excitement, and happiness. Altogether, these findings complement the body of knowledge on Self-Determination Theory (SDT) in intensive training settings. Moreover, the motivational and emotional trajectories support the possibility of promoting elite athletes' outcomes with targeted sessions that are suitable for the competitive environment. Despite the absence of a control group, the program provides additional knowledge about the effects of longitudinal and practical need-supportive interventions comprising reflective practice analysis of coaching in intensive training centers. Lay summary: This study explored the effects of a need-supportive intervention on athletes' psychological experiences in intensive training centers. The intervention was targeted at the coaches and comprised theoretical knowledge and practical analyses. The results revealed an improvement in some motivational and emotional experiences of the athletes.IMPLICATIONS FOR PRACTICE Motivational and emotional experiences of young athletes can be improved with a need-supportive intervention. Practical analyses of coaches, based on the Self-Determination Theory, represent an opportunity to foment a need-supportive climate. A need-supportive climate can be promoted in intensive training centers with short targeted interventions.
254	Implementation and evaluation of a standardized performance profile intervention with collegiate athletes: A comparison of the original and revised techniques	Castillo, EA; Bird, MD; Chow, GM	JOURNAL OF APPLIED SPORT PSYCHOLOGY	2022	10.1080/10413200.2020.1862358	S3	WoS	Performance profiling is a theory-driven technique commonly employed by sport psychology professionals to enhance various psychological outcomes within athletes and teams. Over time, empirical and anecdotal evidence have advocated the applied use of this technique and several adaptations of the original profiling procedure have emerged. The purpose of this study was to examine the impact of the original and revised performance profile (PP) techniques compared to a didactic active control condition on self-awareness, intrinsic motivation, basic psychological need satisfaction, and commitment. A standardized intervention protocol was developed for each performance profiling procedure and utilized to guide implementation of the interventions. Using a randomized design, 117 collegiate athletes from different sports were allocated to a(n) original PP (n = 41), revised PP (n = 42), or didactic PP (n = 34) condition to examine the pre-post impacts of a single session delivered in a team setting. Two-way mixed ANOVAs and MANOVAs were used to analyze the pre to post changes across intervention conditions for the outcome variables. Paired comparisons using two-way mixed ANOVAs were used to determine which intervention was most effective. Results revealed that self-awareness significantly increased for the original and revised condition, however, no differences in improvement were seen between the groups. Furthermore, those in the original group reported significantly higher scores on numerous intrinsic motivation subscales from pre to post intervention, while significantly elevated scores for commitment were reported in the revised condition. Lay summary: Performance profiling is an assessment tool used by athletes, coaches, and sport psychology professionals to help identify athletes' strengths and weaknesses. The original and revised versions of the technique may influence different psychological constructs. Implications for practice Sport psychology professionals should carefully consider which performance profile they implement in practice as the original and revised versions influence different psychological constructs. Practitioners might contemplate, from their perspective, the usefulness of data derived from each performance profiling method, before implementing a version in practice.
255	Exploring the Relationship Between Athletes' Perceptions of Their Team Motivational Climate and Their Sport Shame	Fontana, MS; Fry, MD; Moore, EWG	SPORT PSYCHOLOGIST	2022	10.1123/tsp.2021-0145	S3	Scopus+WoS	Athletes have reported that they would experience shame while playing sport, both for their lack of preparation (process shame) and for their poor outcomes (result shame) during competition. The purpose of this study was to explore how motivational climate is related to athletes' process and result shame. A survey was administered to 259 high-school track and field athletes before a practice 3 weeks into the season. Structural equation modeling showed that a perceived caring and task-involving motivational climate was positively related to athletes' process shame and negatively related to their result shame. Perceptions of an ego-involving motivational climate were negatively related to athletes' process shame and positively related to athletes' result shame. The results highlight that caring and task-involving behaviors in coaches may help mitigate proneness to shame in athletes.

256	A Coach Development Program: A Guided Online Reflective Practice Intervention Study	Da Silva, EJ; Mallett, CJ; Sánchez-Oliva, D; Dias, A; Palmeira, A	JOURNAL OF SPORTS SCIENCES	2022	10.1080/02640414.2022.2045795	S3	WoS	Coaches can influence athlete outcomes, such as performance and personal development; yet, coaches themselves are learners in their own right, who seek to develop their coaching craft. Reflective practice is essential for coaches' development; however, coaches might engage and benefit from reflective practice in myriad ways. This study aimed to evaluate if online reflective journaling (ORJ) enhances the depth of reflection of sports coaches in a 4-week coach development programme (CDP). Participants were a convenience sample of 83 sports coaches from several sports, divided into an intervention group (N = 42) and a control group (N = 41). Data collection used a mixed-methods approach, examining a CDP focused on the coaches' reflective practice. The results revealed that reflection was the only dependent variable that showed significant differences over time. Participation in ORJ showed positive effects on reflection in both groups; however, only the experimental group was statistically significant. For this sample, ORJ was found to help enhance coaches' reflection towards critical reflection. All texts included in pre-, post-, and follow-up tests were coded for trustworthiness purposes. This finding supports the potential of ORJ in nurturing reflective practice, which is considered a core competency in becoming a successful sports coach.
257	Longitudinal trajectories of emotions among athletes in sports competitions: Does emotional intelligence matter?	Levillain, G; Martinent, G; Vacher, P; Nicolas, M	PSYCHOLOGY OF SPORT AND EXERCISE	2022	10.1016/j.psychsport.2021.102012	S3	Scopus+WoS	Objectives: This research explored whether several subgroups of athletes representing distinct emotional trajectories could be shown to exist within the latent class growth analysis (LCGA) of pleasant and unpleasant sport emotions (anger, anxiety, dejection, excitement, happiness). A secondary aim was to explore whether athletes belonging to distinct emotional trajectories reported distinct scores of trait-emotional intelligence (EI) at time 1 (T1). Design: A longitudinal three-wave measurement design (beginning, middle, and end of a competitive season) was used in the present study. Method: A sample of 460 athletes completed the sport emotion questionnaire across three measurement times and the brief emotional intelligence scale at T1. Results: Results of LCGAs showed five trajectories for anger, anxiety and happiness, and six trajectories for dejection and excitement. Furthermore, athletes belonging to distinct (adaptive or maladaptive) trajectories reported significantly different scores of EI. Conclusions: LCGA results highlighted the athlete heterogeneity in longitudinal sport emotions. Higher scores of EI were reported by athletes belonging to adaptive emotional trajectories. These results could be used to help coaches and sport psychologists to identify athletes at risk of developing dysfunctional emotional trajectories across the competitive season. Moreover, this study suggested potential benefits of EI-centred interventions to help athletes to experience adaptive emotional trajectories. Finally, these results highlighted that heterogeneity in sport emotions must be accounted for in future research.
258	The effects of self-compassion on daily emotion regulation and performance rebound among college athletes: Comparisons with confidence, grit, and hope	Doorley, JD; Kashdan, TB; Weppner, CH; Glass, CR	PSYCHOLOGY OF SPORT AND EXERCISE	2022	10.1016/j.psychsport.2021.102081	S3	Scopus+WoS	Self-compassion (SC) facilitates healthy responses to stressful events among athletes. Existing findings are pre-dominantly retrospective, however, and questions remain about the role of SC in college athletes' daily emotion regulation and sport performance. Sixty-seven college athletes completed daily diaries for 14 consecutive days during their competitive seasons. We tested whether SC predicted 1) emotion regulation strategy use and effectiveness following negative events and 2) sport performance rebound following subjectively poor perfor-mances the previous day. We also compared the effects of SC with grit, sport-specific hope, and sport-related self-confidence, each in separate models. Results showed that SC and sport-specific hope predicted greater use of cognitive reappraisal in response to negative events. Interestingly, social support seeking was associated with higher negative emotions on average. However, higher levels of SC, grit, and sport-specific hope (but not sport self-confidence) mitigated this association. When athletes' subjective performance during practice fell 1 SD below their two-week mean, SC, grit, and sport-specific hope predicted improved subjective performance during the next day's practice by 7.8-10.9%. Sport confidence had no effect, suggesting that coaches and sport psy-chology consultants might consider shifting their focus toward cultivating SC instead.

259	A shame of inches: Are teams with black head coaches more heavily penalized in Division 1 college football?	Davis, AP; Leppard, TR; Kinney, AB	SOCIAL SCIENCE QUARTERLY	2022	10.1111/ssqu.13117	S3	WoS	Background A popular line of media discourse has sought to provide explanations for college football's lack of diversity in the coaching ranks. A related effort has recently sought to explain why black coaches are faced with institutional barriers to success in athletic fields. While research on race and the sociology of sport has frequently focused on racial characteristics and expectations of players for on-field penalization, this literature neglected to examine racialized leadership positions such as coaching. Further, the vast majority of this line of literature that has examined racial leadership has explored the effects of race on hiring and promotion decisions off the field rather than on-field dynamics. Objective This article fills this gap by exploring the relationship between the race of college football coaches and the level of on-field penalization their teams incur. Are teams coached by black coaches more heavily penalized than those coaches by non-black coaches? Methods Using data from a merged unique data set and a two-step approach comprising two statistical procedures, we explore this question empirically. We first examine the mean levels of penalties at the game level using a comparison of means, followed by a panel analysis of penalties at the yearly-school level. Results In our comparison of means across all games played between white and black coaches in the 2019/2020 college football season, we find that black coaches are more heavily penalized than white coaches in terms of both penalties per game as well as penalty yardage. Findings of our longitudinal analysis covering all Division 1 college football teams from the college football playoff era (2014/2015 season until the 2019/2020 season) reveal that teams coached by black coaches receive more penalties per game than do teams coached by non-black coaches. Conclusion To conclude, we discuss implications for these findings and suggest ameliorative prescriptions to combat bias in college sports. We contextualize our findings within the context of racialized organizational processes in college sports.
260	Understanding the sleep of ultra-marathon swimmers: Guidance for coaches and swimmers	Dunican, IC; Perry, EL; Gemma, M; Nesci, E; Roberts, S	INTERNATIONAL JOURNAL OF SPORTS SCIENCE & COACHING	2022	10.1177/17479541221089385	S3	WoS	Open water swimming ultra-marathon events >= 10 km have become increasingly popular amongst master athletes. However, very little is known about the timing of training sessions and the impact on sleep. This study aimed to examine sleep behaviours, sleep problems and disorders and the relationship with training timings. This study used a longitudinal observational design for 42 nights with 24 masters' swimmers (n = 13 females), aged 39 +/- 11 years, body mass index of 26 +/- 3 kg/m(2) during a training squad for an ocean ultra-swim (19.7 km) in Western Australia. Objective measures of sleep were obtained from a wrist-activity monitor, the Readiband (TM) (Fatigue Science Inc., Canada). Swimmers completed a survey instrument related to sleep problems, disorders, chronotype, anthropometric and demographic information. Generalised linear mixed models were fitted to examine relationships between predictor variables and sleep responses. Body mass index was associated with a decline in Total Sleep Time (TST), each one-unit increase in BMI was associated with 5 min less TST (p = 0.04). Swimmers with a high risk of sleep apnea had 21 min more wake time (p = 0.04) and 5% lower Sleep Efficiency (p = 0.04). Sleep Offset on the morning of a morning training session was earlier by 48 min (p < 0.001) resulting in less TST by 39 min (p < 0.001). This study provides evidence that coaches need to consider sleep behaviours and problems before designing training schedules. Swimmers need to plan and allocate an adequate sleep opportunity and those who have a suspected sleep disorder or problem should seek the support of a sleep physician.
261	Transcultural Adaptation and Psychometric Support for a Brazilian Portuguese Version of the Flow State Scale (FSS-2)	Garcia, WF; Nascimento, JRA Jr; Mizoguchi, MV; Brandao, MRF; Fiorese, L	PERCEPTUAL AND MOTOR SKILLS	2022	10.1177/00315125221093917	S3	Scopus+WoS	The flow experience in sports is a construct of great interest to recreational and competitive athletes, coaches, and psychologists in pursuit of optimal performance. As there are no validated instruments for evaluating flow in the Brazilian Portuguese language, we evaluated the psychometric properties of a Brazilian version of the Flow State Scale (FSS-2) through three steps. Initially, four translators and five sports psychology specialists adapted the FSS-2 content for the Brazilian Portuguese language. Second, 371 athletes of both sexes who were engaged in group and individual sport modalities and who participated in national university sports competitions from 24 states responded to the adapted version of the FSS-2. Third, an independent sample of 34 athletes from Parana responded to both the adapted FSS-2 and the dispositional flow scale (DFS-2) to permit analysis of the external validity and temporal stability of the adapted FSS-2. We found that the Brazilian version of the FSS-2 contains clear and pertinent items with a good content validity coefficient (CVC = 0.94) and satisfactory internal consistency (alpha > 0.88/CC > 0.80). Confirmatory factor analysis revealed that the adapted 36-item model presented adequate fit [X-2 (558) = 1258.85; X-2/df = 2.256; comparative fit index (CFI) = 0.92; non-normed fit index (NNFI) = 0.90; Tucker-Lewis Index (TLI) = 0.91; root mean square error of approximation (RMSEA) = 0.06 (0.05-0.06); (RMSEA <0.05) =.001] with all first order factors (challenge-skills balance, action-attention fusion, clear goals, feedback, intense concentration, control, loss of self-awareness, time transformation, and autotelic experience). External validity (r > 0.344) and temporal stability (0.53 < intraclass correlation coefficient (ICC) < 0.86) were satisfactory. We conclude that the Brazilian version of FSS-2 is adequate to evaluate flow states experienced by Brazilian athletes following a sports competition.

262	The impact of verbal and physical abuse on distress, mental health, and intentions to quit in sports officials	Brick, NE; Breslin, G; Shevlin, M; Shannon, S	PSYCHOLOGY OF SPORT AND EXERCISE	2022	10.1016/j.psychsport.2022.102274	S3	WoS	Sports officials (e.g., referees, umpires, judges) can experience distress from a multitude of sources, including episodes of verbal and physical abuse from spectators, coaches, and athletes. Little is known about the impact of this abuse on mental health (MH) outcomes and intentions to quit, however. As such, the primary aims of this study were to survey the prevalence and frequency of abuse in sports officials and to examine relationships between abuse, distress, and subsequent MH and intentions to quit outcomes. Survey data were collected from 438 Gaelic Games match officials. Of these, 94.29% and 23.06% had experienced verbal and physical abuse respectively during their career. Verbal abuse was mostly experienced a couple of times a season (reported by 43.83% of officials) or every couple of games (31.48%), whereas physical abuse was predominantly experienced once or twice in a career (85.15%). Structural equation modelling was used to assess three alternative models that proposed the relationship between experiences of abuse, and MH and intentions to quit outcomes to be either 1) direct, 2) indirect, fully mediated by distress, or 3) both direct and indirect, partially mediated by distress. For verbal abuse, only the direct and indirect effects model achieved acceptable fit and significantly explained variance in mental wellbeing (9.4%), anxiety (15.2%), depression (15.6%), and intentions to quit (19.1%). For physical abuse, though higher distress was associated with poorer MH and greater intentions to quit, none of the models fully explained the relationships between all variables. These findings demonstrate, for the first time, relationships between abuse, subsequent distress, and MH outcomes. We highlight the urgent need to develop evidence-based psychological interventions to tackle abuse, manage conflict, and support the MH needs of sports officials.
263	A Coach-Delivered Dating Violence Prevention Program: Coach Perspectives on Implementation	Fields, AD; Fenn, M; Ripper, L; Hill, A; Mulbah, P; Kass, NM; Miller, E	JOURNAL OF INTERPERSONAL VIOLENCE	2022	10.1177/08862605221092073	S3	Scopus+WoS	Coaching Boys into Men (CBIM), an evidence-based violence prevention program, utilizes the coach-athlete relationship to deliver information about healthy, respectful relationships to adolescent boys involved in athletics. Although CBIM has demonstrated effectiveness in clinical research trials, its implementation outside of an effectiveness study has not been explored. The purpose of this current study was to explore facilitators and barriers of effective implementation in community settings using experiences from one regional implementation in Southwestern Pennsylvania. Head coaches who implemented CBIM from 2017-2018 seasons (n = 35) were contacted and 22 (63%) agreed to participate. Coaches were asked how they delivered CBIM to their team, what aided the process, barriers to facilitation, and their overall thoughts on CBIM implementation. Interviews were structured to uncover specific information regarding implementation while also providing time and space for coaches to voice their suggestions and opinions. Interviews were coded using descriptive content analysis. Key facilitators to implementation were involvement of violence prevention advocates, athletic directors, assistant coaches, and players and having flexibility to integrate current events to emphasize topics covered in CBIM. Observed improvements in bystander intervention behaviors and better attitudes towards women and girls among athletes were key motivators for coaches to continue the program. Main barriers were inconsistencies in time available to discuss topics with players and need to refresh curriculum content over multiple seasons. Coaches identified components necessary for broader and sustained implementation of this evidence-based violence prevention intervention in community settings. Coaches' confidence in delivering program content can be bolstered with appropriate strategies in place to address barriers. As coaches witness positive impacts among their athletes, these successes contribute to coaches recognizing their role in transforming sports into a platform for community-wide violence prevention.
264	The role of the anaerobic speed reserve in female middle-distance running	Hallam, LC; Ducharme, JB; Mang, ZA; Amorim, FT	SCIENCE & SPORTS	2022	10.1016/j.scispo.2021.07.006	S3	WoS	Objectives. - Middle-distance running represents a complex interplay of metabolic and mechanical factors. A better understanding of the requirements of male 800 m running has been proposed using the anaerobic speed reserve construct. However, the anaerobic speed reserve is yet to be investigated within female middle-distance running. Methods. - The anaerobic speed reserve, defined as the difference between maximal sprint speed and maximal aerobic speed, was assessed in 12 sub-elite female middle-distance runners using fastest 15 m sprint times and a maximal incremental treadmill test, respectively. Participants were allocated to either 400-800 m or 800 m-Mile subgroups. Comparisons between groups were made for anaerobic speed reserve, maximal sprint speed, maximal aerobic speed and the speed reserve ratio, defined as maximal sprint speed divided by maximal aerobic speed. The relationships between the anaerobic speed reserve components and 800 m season's best race times were assessed. Results. - Female 400-800 m middle-distance runners had a significantly larger anaerobic speed reserve (P = 0.013), faster maximal sprint speed (P = 0.001) and greater speed reserve ratio (P = 0.042) than runners in the 800 m-Mile group. There was a significant negative correlation between maximal aerobic speed and 800 m time (P = 0.012), but no statistically significant relationship was observed for anaerobic speed reserve (P = 0.900), speed reserve ratio (P = 0.558) or maximal sprint speed (P = 0.057). Conclusions. - Female middle-distance subgroups can be distinguished using the speed reserve ratio, with implications for coaches and physiologists to use the speed reserve ratio as a tool tocharacterize athletes and advise individualized training prescription. Aerobic power appears tounderpin female 800 m performance as opposed to anaerobic or sprint abilities in these sub-eliteathletes. (c) 2022 Elsevier Masson SAS. All rights reserved.

265	Effectiveness of the Activate injury prevention exercise programme to prevent injury in schoolboy rugby union	Barden, C; Hancock, M; Stokes, KA; Roberts, SP; McKay, CD	BRITISH JOURNAL OF SPORTS MEDICINE	2022	10.1136/bjsports-2021-105170	S3	WoS	Objective The efficacious Activate injury prevention exercise programme has been shown to prevent injuries in English schoolboy rugby union. There is now a need to assess the implementation and effectiveness of Activate in the applie setting. Methods This quasi-experimental study used a 24-hour time-loss injury definition to calculate incidence (/1000 hours) and burden (days lost/1000 hours) for individuals whose teams adopted Activate (used Activate during season) versus non-adopters. The dose-response relationship of varying levels of Activate adherence (median Activate sessions per week) was also assessed. Player-level rugby exposure, sessional Activate adoption and injury reports were recorded by school gatekeepers. Rate ratios (RR), adjusted by cluster (team), were calculated using backwards stepwise Poisson regression to compare rates between adoption and adherence groups. Results Individuals in teams adopting Activate had a 23% lower match injury incidence (RR 0.77, 95% CI 0.55 to 1.07), 59% lower training injury incidence (RR 0.41, 95% CI 0.17 to 0.97) and 26% lower match injury burden (95% CI 0.46 to 1.20) than individuals on non-adopting teams. Individuals with high Activate adherence (>= 3 sessions per week) had a 67% lower training injury incidence (RR 0.33, 95% CI 0.12 to 0.91) and a 32% lower match injury incidence (RR 0.68, 95% CI 0.50 to 0.92) than individuals with low adherence (<1 session per week). While 65% of teams adopted Activate during the season, only one team used Activate three times per week, using whole phases and programme progressions. Conclusion Activate is effective at preventing injury in English schoolboy rugby. Attention should focus on factors influencing programme uptake and implementation, ensuring Activate can have maximal benefit.
266	The Effects of an Exploratory Mindfulness Intervention on Collegiate Performance	Trajkovski, M; Newl, A	SPORT PSYCHOLOGIST	2022	10.1123/tsp.2021-0088	S3	Scopus+WoS	Although mindfulness has been suggested as a means to improve athletes' performance, few studies have connected changes in mindfulness with improved performance. The purpose of this study was to explore the effect of an exploratory mindfulness intervention (MI) on performance using a mixed methods design. Thirty-four female National Collegiate Athletic Association (NCAA) soccer athletes participated in a 12-week MI. Using the Mindfulness Inventory for Sport, athletic performance and self-perceived ability during the first three games of the season compared to the final three games of the season (August-November 2019) were analyzed using paired symbolscript tests. Postintervention focus groups explored athletes' perceptions of the MI on performance. Mindfulness, shots per game, and self-perceived athletic ability increased after the MI. Six themes emerged from the focus groups: awareness, increased focus, letting-go mentality, skill acquisition, self-compassion, and team cohesion. Coaches and sport psychology practitioners may consider implementing similar MI to improve performance and overall athletic experiences.
267	Mental training in the opinion of the Polish national karate kyokushin team	Piepiora, P; Baginska, J; Jaskowska, K; Witkowski, K	ARCHIVES OF BUDO	2022	10.2154/matarts.8754	S3	WoS	Background and Study Aim: The widely understood mental training was adopted as the background of the work as it has a sound ground-ing in numerous scientific papers as well as in the experience of trainers and coaches. The aim of the paper was to verify the opinions of kyokushin karatekas on mental training. Material and Methods: The Polish representation of juniors and seniors in karate kyokushin (n = 48) from the 2021/2022 season took part in the study. The average training experience in the entire sample of respondents was 12.7 years. Three people are kata competitors and 45 people are kumite competitors. A diagnostic survey method was used, and for this purpose the authors' own questionnaire was used. Results: The investigated athletes pay a significant role to mental preparation: they cooperate with sports psychologists and mental trainers; some of them also carry out their own mental training. Standard methods of mental prep-aration as well as meditation and wakefulness are used by the surveyed karatekas. These methods help to re- duce the level of stress, which some of them experience before the competition day, while others experience stress on the day of the competition. The training level of their opponents does not affect their stress level. Conclusions: For the studied athletes, mental training is very important. Karatekas cooperate with specialists, and also in- dependently undertake mental exercises from the karate culture array. Practicing mental training by karatekas reduces their stress level. Most of them experience stress only on the competition day and this stress is re- lated only to the starting situation.
268	Winning the second half: The perceived and actual impact of the coach's half-time speech on basketball players' performance	Zach, S; Avugos, S; Bakalo-Kuffler, L; Bar-Eli, M	INTERNATIONAL JOURNAL OF SPORTS SCIENCE & COACHING	2022	10.1177/17479541221089743	S3	WoS	This study was designed to explore coaches' half-time speeches and whether players perceive these speeches as having an impact on their performance later in the game. A mixed-methods convergent design was used. Participants were nine male basketball players aged 19-34 (M = 27, SD = 4.76) from a team playing in the Israeli Basketball Premier League, as well as their head coach (aged 41) and two assistant coaches (aged 51 and 34). Data were collected on the coach's speeches at half-time in games during the regular season. In addition, face-to-face semi-structured interviews with the players and the two assistant coaches were conducted at midseason. The average speech duration was approximately 3 min, and messages were delivered at an average pace of one theme every 13 s. The most frequent theme was psychological, with more negative themes delivered compared to positive ones. Psychological themes appeared 50% more than informational. We discuss the differences in the features of the talks between games in which the team was trailing at half-time, and games in which they reached half-time with a leading score. A surprising gap exists between the participants' perception regarding the contribution of the half-time speech to players' performance, and the actual score at the end of the game.

269	Why Does Teamwork Execution Breakdown? Experiences of University Team Sport Athletes	McEwan, D; Crawford, KL	SPORT EXERCISE AND PERFORMANCE PSYCHOLOGY	2022	10.1037/spy0000290	S3	WoS	Teamwork is a dynamic process that can fluctuate over a team's time together, including within a competition. The purpose of the present study was to better understand why this process breaks down, whereby teams do not demonstrate effective teamwork execution. To do so, 18 British university athletes (11 men, 7 women; M (age) = 21.4 years) from interdependent team sports were interviewed on two occasions and asked to describe experiences in which their team did not communicate, coordinate, or cooperate effectively during gameplay. Underpinned by a critical realist approach and through abductive thematic analysis, we developed seven themes (comprised of 16 subthemes) which reflected precursors to teamwork breakdowns. These included: (a) ineffective team preparation during training and during the pre-competition warmup; (b) ineffective team monitoring, problem solving, action planning, and conflict management during in-competition transition periods; (c) changes to the team's roster composition over the season and during games; (d) unhelpful leadership from coaches and athlete leaders during gameplay; (e) poor unity among team members regarding the team's instrumental objectives and social relationships; (f) problematic levels of confidence between teammates and among the team as a whole; and (g) poor performance of one's team and successful performances of one's opponent during the competition. The novel findings from this study extend current knowledge of teamwork and group dysfunction in sport and provide directions for future research on teamwork breakdowns. The potential applied implications for coaches and other team leaders (e.g., sport psychology consultants, and athlete leaders) related to these findings are also highlighted.
270	Athletes' perspectives of preparation strategies in open-skill sports	Bonk, D; Tamminen, KA	JOURNAL OF APPLIED SPORT PSYCHOLOGY	2022	10.1080/10413200.2021.1875517	S3	Scopus+WoS	Preparation strategies (i.e., pre-game rituals, pre-performance routines) have long been a topic of study in sport psychology. However, previous research has focused on closed-skill tasks and employed methodologies that make it difficult to capture athletes' perspectives of these behaviors. The purpose of this study was to explore the perceptions of open-skill sport athletes regarding their preparation strategies. Nineteen high-performance athletes participated in semi-structured interviews. Participants described their perceptions of the structures and functions of the behaviors they consider to be part of their preparation strategies. These findings contribute to a more nuanced understanding of how athletes perceive what they do to prepare for a performance. Exploring athletes' perspectives of this phenomenon allowed for existing theoretical representations of strategies to be compared to the lived experiences of the athletes who engage in them having implications for coaches and sport psychology consultants. Lay Summary: Many athletes have strategies they use to help prepare themselves to perform at their best. We asked 19 high-performance open-skill sport athletes to describe what their preparation strategies are and why they engage in them. Coaches and practitioners should consider their athlete's perspective when working to establish an effective preparation strategy. Implications for practice Sport psychology practitioners should strive to understand what components an athlete perceives to be part of their preparation strategy and why they engage in them. Preparation strategies can help athletes achieve optimal physical, mental, and emotional states, which differ between athletes and contexts. Preparation strategies are tied to athletes' individual identities, and in team sports, can help to build and maintain a team's collective identity over the course of a season.
271	Examining links between coaching behaviors and collegiate athletes' burnout levels using a longitudinal approach.	Mellano, Kathleen T. ; Horn, Thelma S. ; Mann, Mallory	Psychology of Sport & Exercise	2022	10.1016/j.psychsport.2022.102189	S3	SPORTDiscus+Scopus	Research on burnout in athletes reveals a range of potential negative effects including decreased performance, compromised physical and psychological well-being, and possible sport withdrawal. The current study was designed to: (a) examine if and how collegiate athletes' levels of burnout change from the beginning to the end of a competitive season; and (b) determine if there is a predictive link between athletes' perceptions of their coaches' interpersonal styles and feedback behavior and changes in athletes' level of burnout. Self-report measures were completed by 126 collegiate female athletes at two time-points. A repeated measures MANOVA revealed a significant time main effect showing increases over the season in two of the three burnout dimensions (reduced accomplishment and sport devaluation). Hierarchical regression analyses indicated that perceived coaching behaviors did explain a significant amount of the variability between athletes in their end of season burnout scores above and beyond that explained by their early season levels and their perceptions of their performance success. These results add to the growing body of literature on the utility of self-determination theory as a framework for examining psychosocial health and well-being in collegiate athletes. In addition, the longitudinal nature of this study provides information regarding potential changes in the dimensions of burnout as a function of time in the season and in response to perceptions of coaches' behaviors. • Reduced accomplishment and sport devaluation increased across a competitive season • Exhaustion perceptions did not change but over 40% of athletes classified as high • More athletes report relatively high scores in all burnout dimensions at the end of the season • Autonomy-supportive style negatively predicted all late season burnout dimensions • Punishment-oriented feedback was a positive predictor of late season exhaustion

272	Resolving uncertainties of the factor structures of the Coach-Athlete Relationship Questionnaire (CART-Q)	Woolliams, D; Spencer, K; Walters, S; Krägeloh, C	AUSTRALIAN JOURNAL OF PSYCHOLOGY	2021	10.1080/00049530.2021.1882275	S1	WoS	Objective: The Coach-Athlete Relationship Questionnaire (CART-Q) measures the nature of the coach-athlete relationship, yet the psychometric properties of the instrument still remain unclear. The purpose of this study was to resolve uncertainty about a suitable factor solution by applying previously used analytical methods, asymptotic distribution-free confirmatory factor analysis (CFA) and Rasch analysis. Method: The 11-item direct and meta-perspective versions of the CART-Q were examined utilising AMOS, LISREL and Rach analysis using a sample of 251 national football and futsal coaches and athletes. A model comparison strategy with AMOS provided a direct comparison with previous studies. LISREL and Rasch analysis was conducted to provide additional information about the CART-Q psychometric properties. Results: CFA with maximum likelihood and diagonally-weighted least squares estimation methods supported the factorial validity of the three-factor first-order model of both CART-Q versions, although a unidimensional solution was also tenable. Rasch analysis determined that the unidimensional model possessed higher reliability and provided improved coverage of participants' scores, minimising ceiling effects. Conclusion: Research designs that require assessment of coach-athlete relationships may prefer the unidimensional model due to its superior psychometric properties. When more detailed profile scores are required, the subscale scores can still be interpreted with adequate validity and reliability What is already known about the topic: The coach-athlete relationship is essential to enhancing performance and psychosocial well-being. The 3+1Cs model and Coach-Athlete Relationship Questionnaire (CART-Q), represents the most popular approach to understanding coach-athlete relationships. Previous studies investigating the CAR factorial validity have identified the 3+1Cs model as a multidimensional structure with universal applicability across cultures. What this study adds: This is the first study to perform CTT and IRT, to minimise the likelihood of psychometric findings materialising as an artefact of using a particular analytic tool. Contrary to previous research, the results of this study suggest that a unidimensional structure obtained greater robustness in increased reliability, item coverage and reduced ceiling effects than the multidimensionality of the model. These results suggest that a unidimensional factor solution is a more appropriate representation of the model than previously reported and
273	The Personal and Interpersonal Components of Perfectionism: The Italian Validation of Multidimensional Inventory of Perfectionism in Sport	De Maria, A; Mallia, L; Lombardo, C; Vacca, M; Zelli, A	INTERNATIONAL JOURNAL OF ENVIRONMENTAL RESEARCH AND PUBLIC HEALTH	2021	10.3390/ijerph18052657	S2	Scopus+WoS	The present research focused on the general theme of perfectionism in the sport domain, and it provided the first empirical validation of the original 72-item Multidimensional Inventory of Perfectionism in Sport (MIPS) among Italian athletes. The study, specifically, also focused on the relations linking personal and interpersonal components of perfectionism to athletes' competitive anxiety. The research overall relied on data from 644 Italian sport science students and professional athletes and included both cross-sectional and longitudinal designs. Data analyses primarily focused on structural equation modeling, and the findings overall supported the psychometric and construct validity of the Italian version of the MIPS, also highlighting the key role of the personal components of perfectionism.
274	Stressors in Indoor and Field Brazilian Soccer: Are They Perceived as a Distress or Eustress?	Brandao, MRF; Polito, LF; Hernandes, V; Correa, M; Mastrocola, AP; Oliveira, D; Oliveira, A; Moura, L; Boas, MV Jr; Angelo, D	FRONTIERS IN PSYCHOLOGY	2021	10.3389/fpsyg.2021.623719	S2	WoS	Soccer players inescapably live under stress during the sportive career, and many real-life aspects of soccer situations operate in the ongoing performance. This study's main objective was to elaborate the List of Stressors in Professional Indoor and Field Soccer, a self-report instrument designed to measure the impact of 77 soccer situations upon the sport performance. Participants were 138 indoor and field soccer players from the Brazilian Premier League. Each situation was evaluated on a 7-point scale, ranging from the most negative (-3) to the most positive (+3). Data were analyzed according to the players' perception of the items: distress or eustress and its intensity, and after that, situations perceived as plus -1 and +1 were compared by time in which they were experienced and distributed among five categories established by the literature: Expectations about the Performance, Personal Factors, Competition Aspects, Training Demands, and Relationship with Significant People. Narratives of athletes' experiences were also used to discuss the results. An Exploratory Structural Equation Modeling using Bi-factorial (BI-ESEM) was employed to assess the factor structure. For the total participants, 49 situations were perceived as distress and 28 as eustress. Using the criteria established a priori, the distribution was among the five categories in the remaining 32 situations. Differences in perception between less and more experienced players were found in 11 situations. The results revealed that Brazilian professional soccer players experience various stressful situations. These events are important representations of environmental demands and could predict the performance as they are perceived as eustress or distress. Some of these stressful situations are inherent in sport and others adjacent to the sports system or environment. Coach pressure to win and conflicts with teammates are examples of stressors in-sport, family problems and disputes with press or fans are examples of stressors external to the team, also called peripheral opponents, and showed the relative social influence of significant others in soccer performance. We can conclude that the knowledge of the direction of a given stress situation has important practical implications in preparing athletes and helping them face the performance stressors that are part of soccer daily life.

275	Mindfulness training for a college team: Feasibility, acceptability, and effectiveness from within an athletic department	Minkler, TO; Glass, CR; Hut, M	JOURNAL OF APPLIED SPORT PSYCHOLOGY	2021	10.1080/10413200.2020.1739169	S2	Scopus+WoS	Mindfulness-based interventions for athletes-including Mindful Sport Performance Enhancement (MSPE)-have demonstrated promising results. While initial studies of MSPE have found improvements in many areas of athlete functioning (e.g. flow, anxiety, sport performance), questions regarding the feasibility, acceptability, and effectiveness of the protocol when implemented in real-world settings remain. The present pilot study was the first in which a college coach was supervised in using the MSPE protocol to learn mindfulness and develop a consistent practice, and later delivered a 6-week MSPE intervention to all 30 athletes on a Division III NCAA women's lacrosse team. With a 100% completion rate, players rated the program as highly successful, engaged in mindfulness practice outside of weekly sessions, and strongly recommended MSPE training for other athletes; qualitative data also suggested several perceived benefits. After the intervention, participants showed significant improvements in questionnaire measures of sport anxiety, mindfulness, difficulties in emotion regulation, and sport performance satisfaction, with medium to large effect sizes. Furthermore, improvements in sport mindfulness and flow were associated with several dimensions of lacrosse performance. As many universities lack resources to support sport psychology services for athletes, the present study-with its focus on generalizability and feasibility-suggests that MSPE can be delivered by a trained coach to potentially benefit athletes' well-being and performance satisfaction. Ultimately, this train-the-trainer model could create a foundation for sustainable integration of mindfulness and/or mental training in general into college athletic departments. Lay summary: The feasibility and success of a mindfulness intervention with a collegiate women's lacrosse team, when led by a coach trained in mindfulness rather than a professional clinician, sport psychologist, or mindfulness expert, suggests that this train-the-trainer model could promote feasible and sustainable integration of mindfulness programs into university athletic departments.
276	Research on Pre-Competition Mood State of Sports Athletes and Their Individual Differences	Wang, ML	REVISTA DE PSICOLOGIA DEL DEPORTE	2021		S2	WoS	With the development of a country's economy and the progressive improvements in quality of life, sports and fitness are increasingly paid more attention. Regardless of the general sports and fitness-related needs, or the professional athletes who need a substantial amount of exercise, the scientific arrangement of exercise, training intensity and frequency, and monitoring of the athlete's mood state is an important topic. This paper uses bioelectric signals to study the mood state of male athletes during aerobic exercise, and analyzes the changes of the three signals of ECG, EMG and EEG in different physical mood states and the recovery period after exercise. This is done by constructing an objective recognition of the degree of mood state and the degree of recovery after exercise, while tracking and monitoring the changes of the athletes' red blood cell index, blood oxygen saturation, and heart rate indicators during the training process, to objectively evaluate the impact of the long-term training on the athlete's physical function, and provide suggestions for coaches to conduct scientific training. It is also hoped that further summarization of the variation rules of various physiological state evaluation indicators during long-term training of athletes will provide a certain technical support for athletes to avoid overtraining and damaging the body, and in doing so, scientifically restore their sports mood state.
277	A longitudinal examination of thriving in sport performers	Brown, DJ; Arnold, R; Standage, M; Fletcher, D	PSYCHOLOGY OF SPORT AND EXERCISE	2021	10.1016/j.psychsport.2021.101934	S3	Scopus+WoS	To sustain success in sport, athletes need to function effectively in their competitive encounters and maintain this level over repeated events. Yet, to date, little is known about how athletes can continue to fully function (i.e., thrive) in their sporting encounters. Equally, there is a lack of research in relation to the factors that predict thriving. Testing the premise that basic psychological needs (i.e., for autonomy, competence, and relatedness) predict optimal functioning, the aim of this study was to provide the first longitudinal examination of thriving in sport. Sport performers (N = 268) completed questionnaires assessing thriving and basic psychological need satisfaction on three occasions across 28 days. Longitudinal structural equation modeling showed thriving to be highly predicted by both the experience of recent thriving and the perceived satisfaction of basic psychological needs. These findings highlight an important mechanism through which coaches and practitioners can initiate and maintain thriving in the athletes that they work with across a series of sporting encounters.
278	Inhibitory Control Across Athletic Expertise and Its Relationship With Sport Performance	Hagyard, J; Brimmell, J; Edwards, EJ; Vaughan, RS	JOURNAL OF SPORT & EXERCISE PSYCHOLOGY	2021	10.1123/jsep.2020-0043	S3	WoS	Inhibitory control may be vital in elite sport. The authors examined the link between athletic expertise, inhibitory control, and sport performance in a two-part quasi experiment. Inhibitory control was indexed using the Stop-Signal Task, athlete expertise was categorized on literary recommendations, and sport performance was assessed using athlete and coach ratings. Study 1 examined cross-sectional and longitudinal patterns of inhibitory control across athletic expertise. Study 2 investigated whether the inhibitory control-sport performance relationship was moderated by expertise. Study 1 showed that expertise was linked to greater inhibitory control cross-sectionally and longitudinally. Study 2 revealed that expertise was related to superior performance on the Stop-Signal Task and athlete and coach performance ratings, and this relationship was moderated by athletic expertise. Inhibitory control relates to sport performance, increases with greater athlete expertise, and develops longitudinally. Long-term participation in sport may bring about changes in inhibitory control, which may lead to improved sport performance.

279	Extraversion in sport: a scoping review	Allen, MS; Mison, EA; Robson, DA; Laborde, S	INTERNATIONAL REVIEW OF SPORT AND EXERCISE PSYCHOLOGY	2021	10.1080/1750984X.2020.1790024	S3	WoS	This scoping review sought to identify every published study on extraversion in sport. A narrative synthesis was used to interpret findings across research themes. The evidence was graded with validated measures that use quantitative criteria to establish the quality of report writing and confidence in the findings reported. A comprehensive electronic and manual literature search identified 151 published articles (155 independent studies). The research was deductively coded into eight research themes within four causal hypotheses. Study quality varied according to publication date (r = -.52) and the evidence supporting major research questions was graded as 'low' or 'very low' in most instances. The most convincing evidence indicated that athletes are more extraverted than non-athletes (k = 58), team-sport athletes are more extraverted than individual-sport athletes (k = 18), female athletes are more extraverted than male athletes (k = 24), athletes scoring higher on extraversion use more adaptive coping strategies (k = 9), have stronger coach-athlete relationships (k = 6), and tend to be more successful (k = 33). Insufficient evidence was available to draw conclusions regarding playing position, group processes, or team success. Further research on coaches and officials, and using longitudinal and experimental research designs, are recommended.
280	A pilot study investigating cortical haemodynamic and physiological correlates of exercise cognition in trained and untrained cyclists over an incremental self-paced performance test, while thinking aloud	Robinson, NJ; Montgomery, C; Swettenham, L; Whitehead, A	PSYCHOLOGY OF SPORT AND EXERCISE	2021	10.1016/j.psychsport.2021.101912	S3	WoS	Objectives: Few studies have directly investigated changes in cortical haemodynamics during a self-paced interval endurance activity, while collecting conscious cognition and physiological performance data. This pilot study used functional Near Infrared Spectroscopy (fNIRS), while capturing conscious cognition using Think Aloud (TA) during an incremental paced cycling exercise. Methods: A mixed design was implemented with cycling expertise (untrained vs. trained) as the between groups variable and incremental self-paced stage (5 stages of increasing effort) and site (12 optodes across the PFC) as the within groups variables. Dependent measures were the changes in cortical O(2)Hb, and physiological indicators (% heart rate max (%HRmax), average power output (APO), peak power output (PPO), rate of perceived exertion (RPE) and blood lactate (Bla)) over time. Participants used TA throughout their second interval trial. Results: Trained cyclists had higher APO and maximum power output (MPO) from stages 2-5, in addition to a greater increase in PPO over the whole trial. There were significant main effects of stage on %HRmax, Bla and RPE. Differences in cortical haemodynamics were found specifically in areas in the mid left and right PFC. TA data demonstrated that untrained participants verbalised more irrelevant information and feelings of pain and fatigue, in addition to both groups verbalising significantly more motivation-related thoughts during the final stage. Conclusion: This pilot is the first to capture changes in Cox, physiological measures and conscious cognition through the use of TA. We demonstrate the potential role of mid- PFC, and how conscious cognition may change over time. This study has implications for coaches and sport psychologists who may want to understand the cognitions of their athlete during an event and support low level athletes in developing a better understanding of the own cognitions.
281	Role of psychological ownership and authenticity strength in the relationship between authentic leadership and sport team performance: A multilevel analysis	Grégoire, B; Laure, G; Sarah, M; Séverine, C; Evelyne, F	PSYCHOLOGY OF SPORT AND EXERCISE	2021	10.1016/j.psychsport.2021.101965	S3	Scopus+WoS	Objectives: Understanding how and when authentic leadership (AL) is related to teams' performance is an important issue. The objective of the present study was to investigate the potential indirect role of psychological ownership (PO) in the relationship between AL and team sport performance for both athletes and coaches. We also hypothesized that AL perceived strength could moderate this relationship. Design and methods: 337 athletes (243 females) and their 44 coaches (3 females) completed questionnaires assessing AL and PO. Objective performance was assessed at the end of the season. Results: There was a link between players' perception of AL as exogenous variables and team performance as dependent variable via player's perception of PO. Coach's perception of PO did not affect the relationship between coach's perception of AL and team performance. Results did not suggest a moderating effect of AL perception strength. Conclusions: The current study provides the first evidence of relationship between AL and team performance through PO in team sports. However, this study did not support our hypothesis at the coach level. Theoretical and practical implications are discussed.
282	Methodological intervention with soccer coaches to improve athlete-perceived coaching competency, satisfaction with the coach, enjoyment and intention to persist	Pulido, JJ; Leo, FM; González-Ponce, I; López-Gajardo, MA; Sánchez-Oliva, D	INTERNATIONAL JOURNAL OF SPORTS SCIENCE & COACHING	2021	10.1177/1747954120952069	S3	WoS	Objectives The purpose of the study was to assess the effect of a coaching intervention on soccer athletes' perceptions of the coaches' competencies, enjoyment, satisfaction, and intention to persist in the sport. Equipment and methods:The sample consisted of 117 young male soccer players and eight of their coaches who were divided into two groups: group receiving the coaching effectiveness intervention (EG) (n = 4;M-age = 32.50 years,SD = 14.24) with an average of 3.00 years of training experience (SD = 2.44); and control group (CG) (n = 4;M-age = 28.50 years,SD = 13.67) with an average of 6.25 years of training experience (SD = 2.87), and their 117 young male soccer players from 45 teams, aged between 10 and 16 years (M-age = 13.78,SD = 1.38). Focused on coach-effectiveness, coaches from EG received a 12-hour training program. Self-report questionnaires were administered to the athletes both at the beginning and the end of the intervention. A repeated-measures ANOVA was carried out for each dependent variable. Results The results showed significant changes in coaches' game strategy competency, technique competency, and character-building competency as perceived by the players from the EG compared with the players from the CG. Also, both the CG and the EG athletes decreased their satisfaction with the coach. Thus, these types of interventions are effective for improving and maintaining over time variables related to athletes' perception of their coaches' competency, their own enjoyment, and intention to persist practicing sports.

283	It's not just about football all the time either: transnational athletes' stories about the choice to migrate	Ely, G; Ronkainen, NJ	INTERNATIONAL JOURNAL OF SPORT AND EXERCISE PSYCHOLOGY	2021	10.1080/1612197X.2019.1637364	S3	WoS	Due to the growing mobility of athletes and coaches, cultural transitions have recently emerged as a distinct area of interest within athletic career development literature. The purpose of the present study was to extend understanding of cultural transitions by investigating the experiences of transnational athlete migrants within European American Football. Nine male athletes who had crossed national borders to play at least one full season of American Football in Europe were interviewed twice with a focus on discerning narrative meaning-making associated with the choice to migrate and the consequences of these narratives for cultural adaptation and engagement with the host site. Through thematic narrative analysis, we identified three narrative types, a step across the pond: fleeing America, a step up: proving myself, and vehicle for travel: it's a paid vacation, each representing different life themes that had implications for adaptation and well-being. The findings challenge dominant discourses regarding the reasons why athletes migrate while tracing different ways in which adaptation and belonging can be achieved.
284	Follow-up evaluation of the Coaching for Life Skills online training program	Turgeon, S; Camiré, M; Rathwell, S	INTERNATIONAL JOURNAL OF SPORTS SCIENCE & COACHING	2021	10.1177/1747954120964075	S3	WoS	Coach education has been positioned as an important catalyst in enabling coaches to maximise the positive influence of high school sport on student-athlete development. The purpose of the study was to conduct a subsequent season follow-up evaluation, examining longer-term changes in coach-athlete relationship, coach interpersonal behaviours, and life skills teaching in coaches who completed the Coaching for Life Skills (CLS) online training program. A 2 x 2 prospective causal comparative design was used. Coaches (n = 285) were asked to complete follow-up measures during the high school sport season that followed their completion of the trial. The response rate was 36.84%, with the final sample consisting of 64 participants. Data were analysed using independent samplet-tests. From baseline to subsequent season follow-up, scores for coach-athlete relationship and coach interpersonal behaviours significantly increased for the CLS group and significantly decreased for the comparison group. No significant differences in scores were found on life skills teaching from baseline to subsequent season follow-up. The results suggest that the intervention may have helped CLS group coaches remain consistent in their use of relational coaching behaviours.
285	Consent, capacity and compliance in concussion management: cave ergo medicus (let the doctor beware)	Turner, M; Maddocks, D; Hassan, M; Anderson, A; McCrory, P	BRITISH JOURNAL OF SPORTS MEDICINE	2021	10.1136/bjsports-2020-102108	S3	WoS	While the acute effects of concussion and mild traumatic brain injury (TBI) are well understood, the certainty in the medical literature regarding the long-term outcomes of sports-related concussion is limited. Long-term deficits that may result from single, repeated concussions, and possibly subconcussive impacts, include cognitive dysfunction, depression and executive dysfunction. Perhaps most troublingly, repetitive head impacts have been linked to neurodegenerative diseases, including chronic traumatic encephalopathy (CTE), although the precise risk of long-term consequences remains unknown. CTE represents a distinct tauopathy with an unknown incidence in athletic populations; however, a cause and effect relationship has not yet been demonstrated between CTE and concussions or between CTE and exposure to contact sports, as no prospective longitudinal studies have been performed to address that question. Studies of high-school sports exposure and long-term outcomes have not demonstrated consistent findings. Medical advice regarding return to play and the risk of acute and/or long-term consequences is therefore problematic. It is important that the individual's right to make their own choices regarding their health is respected. Team, coach, parental, peer or financial pressures should not influence this decision. The choice to return to play after a concussion or mild TBI injury is the athlete's decision once they have (1) recovered from their injury and have the legal capacity to make an informed decision; (2) been medically assessed and (3) been informed of any possible long-term risks in a language that they can understand. Given the current lack of certainty in relation to long-term outcomes from concussion, is it possible to provide a framework to inform players of current evidence, as part of a consent process, even if the information upon which the decision to return to sport is based remains uncertain and evolving?
286	Acceptance and Commitment Training for Ice Hockey Players: A Randomized Controlled Trial	Lundgren, T; Reinebo, G; Fröjmark, MJ; Jäder, E; Näslund, M; Svartvadet, P; Samuelsson, U; Parling, T	FRONTIERS IN PSYCHOLOGY	2021	10.3389/fpsyg.2021.685260	S3	WoS	Recent systematic reviews on the topic of mindfulness- and acceptance-based approaches in sport psychology conclude that there is a need for further trials using a more robust research methodology with direct performance as outcome. Acceptance and Commitment Training (ACT) is a contextual behavioral change method that focuses on facilitating psychological processes such as values, committed action, acceptance and mindfulness. In the present study designed as a randomized controlled trial, 34 junior elite ice hockey players were allocated into either an ACT group intervention or a wait list control group. Results showed significant effects on both objective performance outcomes (goals, assists, and taken shots) and blinded coach ratings of players' performance, focus and commitment to their development in favor of the ACT group. Effects lasted at 3-month follow-up for the coach ratings, but not for the objective performance measures. All ACT trained players recommended ACT to other players and considered the training as important for their development as ice hockey players. The results add to the growing body of evidence on ACT interventions for athletes and its effect on performance. Future studies should investigate the maintenance of effects from the psychological training over time, using robust research methodology and investigate theoretical coherent potential mediating variables.

287	Harnessing the power of 'us': A randomized wait-list controlled trial of the 5R shared leadership development program (5Rs) in basketball teams	Mertens, N; Boen, F; Steffens, NK; Haslam, SA; Bruner, M; Barker, JB; Slater, MJ; Fransen, K	PSYCHOLOGY OF SPORT AND EXERCISE	2021	10.1016/j.psychsport.2021.101936	S3	WoS	High-quality leadership has been established as a key factor driving a team's competitive advantage. Besides the role of the coach, recent research has emphasized the importance of leadership provided by athletes within a team (i.e., athlete leaders). To unlock the potential benefits of athlete leadership, the development of leaders is therefore essential. The 5R Shared Leadership Program (5R(s)) aims to identify promising leaders within a team, on different athlete leadership roles, both on and off the field. After the appointment of the leaders, their identity leadership skills to build and strengthen a sense of 'we' and `us' are further developed. The design of the present research consisted of a randomized wait-list controlled trial to test the effectiveness of a train-the-trainer approach to develop shared leadership within teams (i.e., 5R(s)). We tracked 16 competitive basketball teams throughout a competitive season. While eight teams (four female and four male teams) received 5R(s) during the first half of the season (i.e., experimental condition), the other eight teams received 5R(s) during the second half of the season (i.e., wait-list control condition). Our findings highlight 5R(s) 's capacity to develop athlete leaders' ability to create a shared sense of 'us', build a stronger team identification, enhance the available social support in the team, help players to remain motivated and confident in their team's abilities, and nurture players' health. Moreover, 5R(s) appeared to achieve this impact by using a train-the-trainer approach, regardless of whether the intervention was delivered during the first or second half of the season, and with generally consistent findings amongst male and female teams. The present study both advances the current field on in-group leadership development, and provides practitioners with guidance on how and when to apply 5R(s) with the aim of improving team functioning and athletes' health.
288	Perceived Coach-Created Motivational Climates as Predictors of Athletes' Goal Reengagement: The Mediational Role of Goal Motives	Martinez-Gonzalez, N; Atienza, FL; Tomas, I; Balaguer, I	FRONTIERS IN PSYCHOLOGY	2021	10.3389/fpsyg.2021.740060	S3	WoS	Athletes have to face several challenges during the sport season, and one of them could involve dealing with unattainable goals. In these situations, being able to reengage in other goals as a form of goal adjustment and in response to contextual demands is adaptive. According to previous literature, some aspects of the athletes' social context, such as coach-created motivational climates, could encourage more adaptive responses in athletes, and so it is possible that these climates would also promote athletes' goal regulation and goal reengagement. The purpose of this study was twofold: to analyze whether athletes' perception of empowering and disempowering climates were related to their goal reengagement through the mediation of goal motives; and to examine the interaction between the two climates when they predict reengagement through athletes' goal motives. Participants were 414 Spanish university athletes (49.5% male, 50.5% female) who belonged to different university teams, with ages ranging from 17 to 33 years old (M = 20.61, SD = 2.58). In the sport facilities, all of them completed questionnaires that evaluated their perception of empowering and disempowering climates, their goal motives, and their goal reengagement. Structural equation modeling (SEM) results showed that perceived empowering climate positively predicted autonomous goal motives, which in turn had a positive relationship with goal reengagement. Conversely, perceived disempowering climate positively predicted controlled goal motives, which were not related to goal reengagement. Thus, we only found support for the indirect relationship between perceived empowering climate and goal reengagement through autonomous goal motives. Moderated mediation analyses revealed that interaction effects between perceived empowering and disempowering climates were not significant in the prediction of goal reengagement through goal motives. Findings revealed that the perception of empowering climates promotes athletes' goal reengagement when goals become unattainable via the increase in their autonomous goal motives. Conversely, when athletes perceive disempowering climates, they have more controlled goal motives, which are not related to goal reengagement. In addition, the study supports the need to educate coaches to create more empowering and less disempowering climates.

289	Health Conditions, Substance Use, Physical Activity, and Quality of Life in Current and Former Baseball Players	Bullock, GS; Nicholson, KF; Waterman, BR; Niesen, E; Salamh, P; Thigpen, CA; Shanley, E; Devaney, L; Collins, GS; Arden, NK; Filbay, SR	ORTHOPAEDIC JOURNAL OF SPORTS MEDICINE	2021	10.1177/23259671211056645	S3	WoS	Background: A comprehensive understanding of lifestyle (health conditions and substance use), health-related quality of life (HRQoL), flourishing (holistic representation of health), and physical activity can inform stakeholders (players, coaches, and clinicians) and help improve long-term health across the life span. Purpose: To describe health conditions (comorbidities or diagnoses), substance use, physical activity, HRQoL, and flourishing in current and former collegiate and professional baseball players and to assess the relationship between playing position and HRQoL/flourishing in former baseball players. Study Design: Descriptive epidemiology study. Methods: Eligible participants were those >= 18 years old with >= 1 season of collegiate or professional baseball experience. Participants completed a survey on health conditions (asthma, diabetes, hypertension, hypercholesterolemia, and depression), substance use (tobacco, alcohol, and energy drinks), physical activity (International Physical Activity Questionnaire-Short Form), HRQoL (Veterans Rand 12-Item Health Survey [VR-12] physical and mental component scores), and flourishing (Flourishing Scale). Adjusted multivariable regressions were performed for HRQoL and flourishing. Results: Overall, 260 baseball players opened the survey, and 214 (current players, 97; former players, 117) participated for an 82% response rate. Of the former players, 32% had hypertension or hypercholesterolemia. In addition, 26% of current players had used smokeless tobacco (median, 3 years; interquartile range [IQR], 1-5 years) as compared with 34% of former players (median, 15 years; IQR, 5-25 years). In addition, 14% of current players had used electronic cigarettes (median, 2 years; IQR, 0-4 years) as opposed to 3% of former (median, 3 years; IQR, 2-4 years). Energy drinks were consumed by 31% and 14%, respectively, of current and former players on at least a weekly basis. Current baseball players performed 8667 metabolic equivalents per week of physical activity as opposed to 3931 in former players. Pitching was associated with worse VR-12 Mental Component Scores (-5.0; 95% confidence interval, -9.0 to -1.0). Playing position was not related to VR-12 Physical Component Scores or flourishing in former baseball players. Conclusion: The similar smokeless tobacco prevalence between current and former baseball players suggests that they may start using tobacco products during baseball participation and continue after retirement. Similar reported HRQoL as compared with the general US
290	An exploratory investigation into the reception of verbal and video feedback provided to players in an Australian Football League club	Mason, RJ; Farrow, D; Hattie, JAC	INTERNATIONAL JOURNAL OF SPORTS SCIENCE & COACHING	2021	10.1177/1747954120951080	S3	WoS	Coach observation studies commonly examine training and competition environments, with little attention paid to the ways in which coaches provide video feedback in a performance analysis setting. In addition, few studies have considered the reception of feedback by an athlete, or the characteristics of the athlete that may support or hinder feedback reception. The purposes of this study were threefold. First, to examine the characteristics of feedback provided by a coach during a typical video feedback meeting. Second, to measure the impact of this feedback on athlete learning. Third, to consider a range of learner characteristics that may impact feedback reception. Six coaches and six players affiliated with an Australian Football League (AFL) club were recruited. Coach-player dyads were observed in one-to-one video feedback meetings following a game played in the 2017 season. Players were interviewed to test feedback recall. Players also completed a series of tests designed to measure learner characteristics, with the intention of discovering moderating factors of the relationship between feedback and learning outcomes. Rates of feedback generally mirrored those found in previous studies. Coaches provided nearly 30 feedback messages during each meeting. Players recalled 50% of summarised feedback messages but just 6% of all feedback a week later. A ceiling effect on learner characteristics was observed. The paper presents a novel design for examining feedback effectiveness while considering learner characteristics. Given the findings on feedback quantity and recall, coaches are encouraged to adopt a 'less is more' approach to providing feedback.
291	Home Team (Dis)Advantage Patterns in the National Hockey League: Changes Through Increased Emphasis on Individual Performance With the 3-on-3 Overtime Rule	Hoffmann, MD; McEwan, D; Baumeister, RF; Barnes, JD; Guerrero, MD	PERCEPTUAL AND MOTOR SKILLS	2021	10.1177/0031512520966138	S3	Scopus+WoS	Past research examining National Hockey League (professional ice hockey; NHL) data from the 4-on-4 overtime era (seasons between 2005-06 and 2013-14) revealed an inconsistent home team (dis)advantage pattern such that home teams that were superior to their visiting counterparts had slightly greater odds of winning during regulation play compared to overtime (demonstrating home crowd advantages for team performance during regulation); in contrast, home teams experienced lower odds of winning in the shootout period than in overtime regardless of team quality (thereby demonstrating risks for individual choking from home crowd pressures). In this study, we explored the NHL home (dis)advantage pattern during four more recent seasons (2015-16 through 2018-19) in which the league instituted 3-on-3 play during overtime (perhaps increasing individual pressure for athletes competing in the 3-on-3 overtime period). We used archival data from the regular season (N=5,002 games) to compare home teams' odds of winning in regulation (with 5-on-5 skaters per team) to overtime (with 3-on-3) and in the shootout, adjusting for the quality of home and visiting teams. We conducted fixed-effects and multi-level logistic regression modeling. Evenly matched home teams were 1.66 times more likely to win than inferior home teams when games concluded in regulation versus overtime. Superior home teams were 4.24 times more likely to win than inferior home teams when games concluded in regulation rather than overtime. Thus, it is apparently more difficult for superior and evenly matched home teams to win in overtime than during regulation, suggesting that such home teams may be susceptible to choking in overtime. In contrast to the earlier 4-on-4 overtime era, home teams did not have lower odds of winning in the shootout compared to overtime. These results may have implications for NHL coaches' and players' tactical decision-making.

292	Dyadic coping in coach-athlete relationships: A grounded theory	Staff, HR; Didymus, FF; Backhouse, SH	PSYCHOLOGY OF SPORT AND EXERCISE	2020	10.1016/j.psychsport.2020.101741	S1	WoS	Objectives: The purpose of this study was to make an original contribution to sport psychology literature by offering a substantive grounded theory of dyadic coping in coach-athlete relationships. Specifically, this study aimed to capture the development and manifestation of dyadic coping for coaches and athletes operating in individual sports. Design: Using constructionist grounded theory methodology as a guide, a theory of dyadic coping was constructed by the authors, 13 coaches, 15 athletes, and five sport and exercise psychology practitioners. Method: Theoretical sampling procedures ensured that data collection was directed by the developing theoretical concepts, rather than a set of predefined criteria. We conducted individual interviews (n = 16) with coaches and athletes, and one 90-min workshop with coaches, athletes, and sport and exercise psychology practitioners. Methodological rigor was enhanced by focusing on credibility, originality, resonance, and usefulness. Results: The theory proposes that when coaches and athletes appraise a stressor communicated in their dyad as significant and meaningful, they use dyadic coping to protect themselves and their coach-athlete relationships. This process is moderated by a number of personal (e.g., personality), relationship (e.g., length), and organizational (e.g., leadership behaviors) characteristics. Conclusion: The theory presented here represents a notable shift in thinking away from coping as an individual process and toward coping as an important interpersonal phenomenon. This type of coping can have long-term effects on coaches' and athletes' relationship functioning, well-being, and performance.
293	'I was excited to train, so I didn't have problems with the coach': dual career athletes' experiences of (dis)empowering motivational climates	Saarinen, M; Ryba, T; Ronkainen, NJ; Rintala, H; Aunola, K	SPORT IN SOCIETY	2020	10.1080/17430437.2019.1669322	S1	Scopus+WoS	In addition to investing in athletic development, adolescent elite athletes are expected to complete their secondary education. As a result of this expectation and the demands of sport and education, they may struggle to sustain high levels of motivation for both domains. Grounded in theoretical tenets of empowering coaching, this study sought to explore student-athletes' perceptions of empowering and disempowering motivational climates and their possible implications for athletes' dual career experiences. Semi-structured interviews were conducted with 17 Finnish student-athletes, and the data were thematically analysed. The analysis indicated that a majority of the athletes had experiences of disempowering coaching climates due to coaches' exclusive emphasis on athletic performance. It is concluded that the perception that obtaining an education is less important than sport may potentially decrease athletes' motivation to pursue an academic track and thus challenge their exploration of future vocations outside the sporting context.
294	Effects of Motivational Climate on Fear of Failure and Anxiety in Teen Handball Players	Gómez-López, M; Chicau Borrego, C; da Silva, CM; Granero-Gallegos, A; González-Hernández, J	INTERNATIONAL JOURNAL OF ENVIRONMENTAL RESEARCH AND PUBLIC HEALTH	2020	10.3390/ijerph17020592	S2	WoS	The aim of this study was to examine the effects of the motivational climate created by the coach and perceived by a group of young, high-performance handball players on their fear of failure and precompetitive anxiety. The study participants were 479 young handball players. The age range was 16-17 years old. Players were administered a battery composed of a Perceived Motivational Climate in Sport Questionnaire, a Performance Failure Appraisal Inventory, and Competitive State Anxiety Inventory-2R, to measure the aforementioned theoretical constructs. Using structural equation modelling (SEM), the results showed that the handball players experienced high levels task-involving climate and moderate values of self-confidence. In contrast, players experienced lower levels of ego-involving climate, fear of failure, and cognitive and somatic anxiety. The obtained model shows that fear of failure positively mediates the association between an ego-involving climate with both cognitive and somatic anxiety, and negatively in terms of self-confidence. In contrast, fear of failure does not mediate the associations between a task-involving climate and both somatic and cognitive anxiety and self-confidence.
295	Heart rate variability and pre-competitive anxiety according to the demanding level of the match in female soccer athletes	Ayuso-Moreno, R; Fuentes-García, JP; Collado-Mateo, D; Villafaina, S	PHYSIOLOGY & BEHAVIOR	2020	10.1016/j.physbeh.2020.112926	S2	Scopus+WoS	The present study aimed to evaluate the effects of a highly-demanding match and a lowly-demanding match on pre-competitive heart rate variability (HRV) and anxiety in semi-professional female soccer athletes. A total of 14 players, with a mean age of 23.78 (4.93), from the Caceres Women Football Club of the Spanish Second National Division participated in our study. They were evaluated in two microcycles which correspond to a highly- and a lowly-demanding matches. For each microcycle a baseline and a pre-competitive measures were collected. Results indicated that HRV was significantly reduced before a highly demanding match whereas a lowly-demanding match did not lead to any change. Significant differences in HRV and cognitive anxiety were observed when compared the highly and the lowly demanding matches, which means an increase in the anxiety levels before the highly-demanding match. HRV could be an indicator of precompetitive anxiety in semi-professional female soccer players. This could be used by coaches or physical trainers as a tool to examine the precompetitive anxiety in athletes.

296	Methods and Approaches of College Counselors to Deal with Students' Psychological Crisis	Guan, RX; Zhang, JB	REVISTA DE PSICOLOGIA DEL DEPORTE	2020		S2	WoS	The transformation and development of higher education into elite education has led to a widespread knowledge and increase in students' numbers in colleges and universities. Simultaneously, with the increasing diversification of social values and the increasingly fierce social competition, psychological problems caused by college students' study pressure, employment pressure, and personal emotions continue to emerge, and college students have become a group with a high incidence of psychological crises. They also face problems in interpersonal communication as they lack self-psychological adjustment skills and have low ability to withstand setbacks, resulting in psychological issues. College counselors are university administrators who have the closest contact with students. They have the most direct influence on students. Therefore, in order to urgently bring some kind of psychological crisis intervention, it is important to establish an effective student psychological crisis intervention system, and explore the methods and ways of students' psychological crisis intervention. This paper examined counselors' important role in the identification and intervention of college students' psychological turmoil. The study found that the rational application of psychological principles can effectively improve the counselors' working ability in psychological crisis intervention, prevent and reduce the occurrence of college students' psychological crisis, and ensure college students' healthy development.
297	Psychometric Properties of the Psychological State Test for Athletes (TEP)	Díaz-Tendero, P; Pérez-Llantada, MC; de la Llave, AL	FRONTIERS IN PSYCHOLOGY	2020	10.3389/fpsyg.2020.566828	S2	WoS	This study has two objectives: to validate an adapted online version of the Psychological State Test (TEP, in its Spanish acronym); and to assess differences in pre-competitive psychological state profiles between amateur and professional athletes in team sports. The TEP questionnaire is an instrument which is used to assess, in a quick and simple fashion, the psychological state of athletes prior to competing. Its psychometric properties were evaluated by means of an analysis of internal consistency, an Exploratory Factor Analysis and a Confirmatory Factor Analysis. The EFA's results showed a factorial structure consisting of two principal factors and reliability coefficients, both globally and at the factor level, which can be considered acceptable (global alpha = 0.81; Factor 1 alpha = 0.85; and Factor 2 alpha = 0.73). The CFA confirmed the model proposed by the EFA so that the items were distributed around these factors, giving rise to one factor which includes variables that have a positive relationship with performance, and another with variables that negatively affect performance. Meanwhile, regarding the difference between the pre-competitive psychological state of amateurs and professionals, professional athletes presented higher levels of Motivation ($p = 0.5$ and $d = -0.23$). It is concluded that the TEP is a suitable tool for the evaluation of pre-competitive psychological states. However, in future research, this study should be complemented by analyzing the TEP's predictive validity in terms of the performance of athletes and/or teams, as well as its use as a tool available to athletes and coaches.
298	Performance Trajectories for Competitive Swimmers: The Role of Coach Interpersonal Behaviors and Athlete Motivation	Rocchi, MA; Guertin, C; Pelletier, LG; Sweet, SN	MOTIVATION SCIENCE	2020	10.1037/mot0000156	S3	WoS	This study investigated the longitudinal trajectories of athletes' competitive performance results, as well as the motivational predictors of the emerging trajectories. In 2010, a group of 413 competitive swimmers were asked to provide background characteristics (age, gender, years of experience) and complete self-determination theory-based measures of their coaches' interpersonal behaviors and sport motivation. The athletes' performances were then followed for seven swimming seasons (2011-2017) and their top score from each season was used to track their trajectories over time. The data were analyzed using latent class growth analysis to determine the best-fitting model of performance trajectories, while controlling for athletes' background characteristics. Athletes' perceptions of coaches' behaviors and their motivation were explored as predictors using a multinomial logistic regression. The analyses revealed 5 latent classes: Early Dropout (23%) Delayed Dropout (40%), Stable (9%), Improvement (18%), and High-Performance (11%). Multinomial logistic regression analysis indicated that, in 2010, members of the Improvement and High-Performance classes reported higher perceived autonomy support (ORs >1.58, CI95s > 1.00) and relatedness support (ORs > 1.58, CI95s > 1.00) than the Early Dropout and Delayed Dropout groups. The Stable, Improvement, and High-Performance groups reported less non-self-determined motivation in 2010 than the Early Dropout and Delayed Dropout Groups (ORs < .43, CI95s < 1.00). These results support that perceptions of coaches' interpersonal behaviors and athletes' motivation predict performance trajectories among swimmers over time.
299	Mental Blocks in Artistic Gymnastics and Cheerleading: Longitudinal Analysis of Flikikammo	Maaranen, A; Van Raalte, JL; Brewer, BW	JOURNAL OF CLINICAL SPORT PSYCHOLOGY	2020	10.1123/jcsp.2018-0101	S3	WoS	Flikikammo is a troubling phenomenon in which athletes lose the ability to perform previously automatic backward moving gymnastics skills as a normal part of a routine. To better understand the effects of flikikammo over time, the confidence, perceived pressure, physical well-being, energy, and stress levels of gymnasts ($n = 6$) and cheerleaders ($n = 4$) were assessed weekly over 10 weeks. Half of the participants reported experiencing flikikammo at the start of the study, and half served as age, skill level, and sport-matched controls. Athletes with flikikammo indicated that pressure from coaches and higher energy levels were related to more severe flikikammo. For participants under the age of 18, higher levels of life stress positively correlated with flikikammo, but for those over 18, higher life stress was negatively correlated with flikikammo. These findings highlight the complexity of flikikammo and suggest that complex solutions may be needed to address flikikammo issues.

300	Performance indicators during international rugby union matches are influenced by a combination of physiological and contextual variables	Crewther, BT; Potts, N; Kilduff, LP; Drawer, S; Cook, CJ	JOURNAL OF SCIENCE AND MEDICINE IN SPORT	2020	10.1016/j.jsams.2019.10.011	S3	WoS	Objectives: Research has linked physiological (e.g., hormonal, affective, fatigue) outcomes to performance indicators in rugby competition, but no work has integrated and contextualised these factors within a test-match environment. We addressed this gap by monitoring 29 athletes from a training squad across eight international rugby matches. Design: Longitudinal observational study. Methods: Pre-match (8-9 am) measures of salivary testosterone and cortisol concentrations, sleep duration, pulse rate, muscle soreness, stress, mood, and motivation were taken. Contextual factors were playing time, internal training load (ITL), test-match experience, opponent ranking, and crowd size. Performance was indexed by coach and player ratings of performance (CRP, PRP) and quantitative metrics; offloads, turnovers, runs with ball in hand (RWB), tackles, passes, and defenders beaten (DFB). Results: Morning cortisol, sleep and mood were positively related to CRP and PRP (standardised coefficient estimates from 0.17 to 0.22). Cortisol, sleep, stress, mood and motivation were associated with one (or more) of turnovers, RWB, tackles, passes and DFB (incidence rate ratio [IRR] from 0.74 to 1.40). Playing time was positively related to all quantitative performance indicators (IRR from 1.01 to 1.04) with ITL, opponent ranking, and crowd size predicting selected outputs (IRR from 0.89 to 1.15). The explanatory models varied (conditional R-2 = 0.15-0.83) but were generally stronger with both physiological and contextual inputs. Conclusions: Multiple physiological and contextual factors appear to contribute to player performance in international rugby competition. Measurement of these factors may guide training and management practices, a potential practical consequence but also advancing understanding from marker to causal link. (C) 2019 Sports Medicine Australia. Published by Elsevier Ltd. All rights reserved.
301	Psychological characteristics, sport engagement, and performance in alpine skiers	Fawver, B; Cowan, RL; DeCouto, BS; Lohse, KR; Podlog, L; Williams, AM	PSYCHOLOGY OF SPORT AND EXERCISE	2020	10.1016/j.psychsport.2019.101616	S3	Scopus+WoS	Previously, investigators have sought to clarify the role of psychological factors in the development of expertise across numerous sport domains; however, almost no empirical work exists on winter sport athletes. Using a retrospective design, we examined associations between psychological traits, engagement in practice-related activities, and performance among sub-elite level, youth alpine skiers. A total of 169 skiers (88 women) enrolled at professional development academies in the United States completed a battery of questionnaires assessing practice history profiles and performance milestones, as well as various psychological factors (e.g., mental toughness, grit, perfectionism). Performance was assessed using national ranking across both speed and technical disciplines. In addition to linear models and MANOVA/MANCOVA tests, linear mixed-effect regressions were utilized to assess relationships between psychological factors, practice hours, and ranking over time. Higher scores on perfectionistic strivings (personal standards) were associated with improved performance. Also, grit was associated with increased engagement in individual practice hours. Coach-led one-on-one practice hours were associated with increased perfectionistic concerns (e.g., parental pressure), while indirect exposure (e.g., attending events without competing) was associated with decreased mental toughness. Findings highlight potentially important associations between athletes' dispositional characteristics, prolonged ski engagement, and performance trajectory.
302	Understanding social networks and social support resources with sports coaches	Norris, LA; Didymus, FF; Kaiseler, M	PSYCHOLOGY OF SPORT AND EXERCISE	2020	10.1016/j.psychsport.2020.101665	S3	Scopus+WoS	Objectives: Research on social support with sports coaches is limited, yet the benefits of social support within other occupations have been widely reported. This study explored sports coaches' social network structures, the social support resources available to coaches, and the situations in which coaches use social support. Design: Cross-sectional. Method: Data were collected with male (n = 6) and female (n = 7) British coaches (M-age = 34.20, SD = 13.37; M-experience = 13.20, SD = 10.41) using semi-structured interviews and interviewee-aided sociograms. Interview data and sociograms were analyzed using abductive thematic analysis and social network analysis to create ego-network diagrams. The ego-network diagrams were created to provide information on the locality and influence of coaches' social network members. Results: The ego-network diagrams highlight that the structure of coaches' social networks encompasses support from peers, friends, family, and miscellaneous (e.g., media). The diagrams also demonstrate that support from friends tended to be perceived as most influential. The coaches called on their network for appraisal (e.g., affirmation), emotional (e.g., venting), informational (e.g., training), and or instrumental support (e.g., cooking dinner) for a variety of situations, such as training (e.g., drill ideas) and issues with athletes (e.g., venting about a misbehaving player). Conclusion: Given the pertinence of coaches' social networks and resources for performance and psychological well-being, coach education programs should include a focus on the importance of building relationships. Longitudinal research methods are warranted to, for example, explore the dynamic functions of coaches' social support. This will develop a more comprehensive base from which interventions can be developed.

303	The 2020 Coronavirus Pandemic as a Change-Event in Sport Performers' Careers: Conceptual and Applied Practice Considerations	Samuel, RD; Tenenbaum, G; Galily, Y	FRONTIERS IN PSYCHOLOGY	2020	10.3389/fpsyg.2020.567966	S3	WoS	The Coronavirus experience (CE) presents a highly challenging period for sport performers (e.g., athletes, coaches, referees), with potential effects on their lives and career trajectories. In this article, we initially conceptualize the CE using the scheme of change for sport psychology practice (Samuel and Tenenbaum, 2011a). Within this framework, the CE is understood as a longitudinal, multifaceted, unpredicted, non-controlled change-event, with four distinct stages: (a) a pre-Coronavirus stage with unique career contextual conditions (i.e., stable engagement or a transitional period), (b) Coronavirus stage-A accompanied by instability and confusion, emotional response, and cognitive appraisal, (c) Coronavirus stage-B characterized by active coping or regression, and (d) Coronavirus stage-C; instability endures or decreases, depending on career trajectory. The CE presents sport performers with modifications in various dimensions, including physical and physiological, motor skills, psycho-social and self-identity, relationships, performance and achievement, motivation and aspirations, organizational-occupational, and micro- and macro-cultural issues. Sport performers can exhibit several emotional responses (i.e., positive, negative, neutral), and consequential coping endeavors. The development of the change process is underlined by key decisions, manifested in sport performers' attempts to implement responsive change in these dimensions (e.g., adapt their diets, sleep routines, and exercise regimen). The second part of the article discusses applied practice considerations, presenting various techniques and methodologies which practitioners can apply while consulting from a change-based perspective. Ethical issues pertaining to the formation of effective therapeutic relationships during this period are also assessed. The conclusions offer future avenues for researchers and practitioners when attempting to evaluate and cope with this global phenomenon.
304	Individual Pathways Through the Junior-to-Senior Transition: Narratives of Two Swedish Team Sport Athletes	Franck, A; Stambulova, NB	JOURNAL OF APPLIED SPORT PSYCHOLOGY	2020	10.1080/10413200.2018.1525625	S3	Scopus+WoS	Athletes frequently describe the junior-to-senior transition (JST) as the most difficult within-career transition, and many athletes have acknowledged that they failed to cope well with it. Athletes' development in the JST is influenced by narratives existing within relevant contexts and settings. This study served as a follow-up to the quantitative longitudinal study to gain a deeper understanding of individual JST paths through a qualitative narrative approach. The aim was to explore 2 team sport athletes' (John, the football player, and Anna, the basketball player) JST pathways, emphasizing psychosocial factors that were perceived as facilitating and debilitating the process. Narrative type interviews were conducted, and the holistic-form structural analysis was used. Through their narratives, John and Anna reconstructed their JST paths, attaching meanings to certain events, recounting the people involved, and making personal reflections. John had a performance and family narrative and Anna had an enjoyment and relationship narrative. They perceived their key facilitating persons to be their family members and teammates. The debilitating factors were some coaches' behaviors. At the time of this study, John and Anna had already terminated their athletic careers and had refocused on getting an education. Although they did not reach elite senior levels in their sports, they found their athletic careers to be meaningful life experiences. Lay Summary: The aim was to explore two team sport athletes' junior-to-senior transition (JST) pathways, emphasizing psychosocial factors involved in the transition process. The findings revealed two narratives. John (football player) storied his JST as a performance and family narrative and Anna (basketball player) as an enjoyment and relationship narrative.
305	Psychosocial experiences of concussed collegiate athletes: The role of emotional support in the recovery process	Wayment, HA; Huffman, AH	JOURNAL OF AMERICAN COLLEGE HEALTH	2020	10.1080/07448481.2019.1577863	S3	WoS	Objective: Understand from whom concussed football players seek and receive emotional support, and whether this support is associated with injury perceptions. Participants: Football players (N = 26) from three NCAA Division I programs. Methods: With approval from the head athletic trainer, concussed athletes (2017 season) completed short surveys within 4-6 days of diagnosis and when cleared to return. Results: Concussed athletes perceived their injury as a normal consequence of playing football, not serious, and reported little, if any, depression and anxiety. Athletes reported the most support from athletic trainers; the least from coaches and teammates. Emotional support was associated with fewer adverse psychosocial reactions, more sport-injury related growth, and greater intentions to report future concussion symptoms. Conclusion: Results from this pilot study suggest that emotional support during the concussion recovery process should be understood and fostered by university officials charged with the health and well-being of collegiate football players.

306	Coach identity leadership behaviours are positively associated with athlete resource appraisals: The mediating roles of relational and group identification	Miller, AJ; Slater, MJ; Turner, MJ	PSYCHOLOGY OF SPORT AND EXERCISE	2020	10.1016/j.psychsport.2020.101755	S3	Scopus+WoS	Background: There is growing evidence identifying the positive effects of sport and exercise leaders engaging in identity leadership. Yet we have limited knowledge of how identity leadership is associated with athletes' resource appraisals (e.g., self-efficacy) and performance, the underpinning mechanisms that explain such relationships, and changes in relationships across a sporting season. Methods: In Study 1, 412 amateur and professional athletes completed seven questionnaires directly prior to athletic competition in a cross-sectional design. In Study 2, 136 athletes completed seven questionnaires directly before competition, and one questionnaire directly after competition both at the start and the end of the athletic season. Results: In Study 1, relational identification and group identification mediated the positive relationship between identity leadership and self-efficacy, control, approach goals and social support. In Study 2, identity leadership at the start of the season predicted self-efficacy at the end of the season through relational identification. Group identification did not significantly mediate the identity leadership-resource appraisal relationship. Perceived social support at the start of the season predicted greater performance satisfaction at the end of the competitive season. Conclusions: Findings provide evidence that sport coaches' engagement in identity leadership is key to forming a shared social identity, which in turn, is broadly adaptive for stress appraisals and performance satisfaction both cross sectionally and longitudinally.
307	Relationship Between Power Output and Speed-Related Performance in Brazilian Wheelchair Basketball Players	Loturco, I; McGuigan, MR; Reis, VP; Santos, S; Yanci, J; Pereira, LA; Winckler, C	ADAPTED PHYSICAL ACTIVITY QUARTERLY	2020	10.1123/apaq.2019-0158	S3	WoS	This study aimed to investigate the association between the optimum power load in the bench press (BP), shoulder press (SP), and prone bench pull (PBP) exercises and acceleration (ACC) and speed performances in 11 National Team wheelchair basketball (WB) players with similar levels of disability. All athletes were previously familiarized with the testing procedures that were performed on the same day during the competitive period of the season. First, athletes performed a wheelchair 20-m sprint assessment and, subsequently, a maximum power load test to determine the mean propulsive power (MPP) in the BP, SP, and PBP. A Pearson product-moment correlation was used to examine the relationships between sprint velocity (VEL), ACC, and the MPP in the three exercises. The significance level was set as p <.05. Large to very large significant associations were observed between VEL and ACC and the MPP in the BP, SP, and PBP exercises (r varying from.60 to.77; p <.05). The results reveal that WB players who produce more power in these three exercises are also able to accelerate faster and achieve higher speeds over short distances. Given the key importance of high and successive ACCs during wheelchair game-related maneuvers, it is recommended that coaches frequently assess the optimum power load in BP, SP, and PBP in WB players, even during their regular training sessions.
308	Perceived Coach Leadership Profiles and Relationship With Burnout, Coping, and Emotions	González-García, H; Martinent, G; Morales, AT	FRONTIERS IN PSYCHOLOGY	2019	10.3389/fpsyg.2019.01785	S1	WoS	The aims of the study were to identify coach profiles and examine whether participants from distinct profiles significantly differed on burnout, emotions, and coping or not. A sample of 268 athletes (M-age = 29.34; SD = 12.37) completed a series of self-reported questionnaires. Cluster analyses revealed two coach leadership profiles: (a) profile 1 with high scores of training and instruction, authoritarian behavior, social support, and positive feedback, and a low score of democratic behavior; and (b) profile 2 with low levels in training and instruction, authoritarian behavior, social support, and positive feedback and high levels in democratic behavior. Results of Multivariate analyses of variance (MANOVAs) indicated significant differences across coach profiles on reduced accomplishment, sport devaluation, happiness and seeking support and marginal differences on dejection, logical analysis, imagery/thought control, and excitement. Moreover, coach leadership profiles were not confounded by demographic variables (level of competition, gender, age, number of practice hours, professional versus no professional athletes). As a conclusion, the profile approach offered a holistic way to examine coach leadership in sport as two distinct coach profiles emerged from the cluster analyses with an unexpected combination of coach leadership dimensions.
309	Working alliance in the coach-athlete relationship and athlete burnout The mediating role of athlete resilience	Moen, F; Hrozanova, M; Stiles, T; Stenseng, F	INTERNATIONAL JOURNAL OF SPORT PSYCHOLOGY	2019	10.7352/IJSP.2019.50.338	S1	WoS	The quality of the coach-athlete relationship has been linked to athlete burnout in previous studies. However, few studies have examined individual factors among athletes that may affect this association, and in particular among young athletes with high activity involvement. Hence, in the present study, we studied the potential mediating role of athletes' resiliency in the effect of the working alliance of the coach and the athlete on the athletes' levels of burnout. Six-hundred and seventy junior athletes from 27 Norwegian high schools specialized for sports participated in a web-based syrvey study where 49% males and 51% were females, and 78% of the athletes had ambitions to become future elite athletes in their sports. Structural equation modeling analyses showed a strong positive association between the coach-athlete working alliance and resilience, and a strong negative association between resilience and burnout. A moderate association was found between the coach-athlete working alliance athlete burnout. Furthermore, a mediational relationship was detected, where the effect of working alliance on less burnout partly was explained by an indirect effect of working alliance through more resilience in the athlete. The current study highlights the importance of a strong working alliance between coaches and athletes to develop athlete resilience, and the importance of athlete resilience as a buffer to prevent occurences of athlete burnout.

310	Development of the Sport Mental Health Continuum-Short Form (Sport MHC-SF)	Foster, BJ; Chow, GM	JOURNAL OF CLINICAL SPORT PSYCHOLOGY	2019	10.1123/jcsp.2017-0057	S1	WoS	Well-being research conducted in competitive athletics has been marred by the lack of a context-specific measurement instrument. The purpose of this study was to adapt the Mental Health Continuum - Short Form (MHC-SF) to create a sport-specific well-being instrument, the Sport Mental Health Continuum Short Form (Sport MHC-SF), and test its initial psychometric properties. Participants were 287 collegiate athletes from a variety of sports. Confirmatory factor analysis (CFA) determined a three-factor structure of sport well-being, consisting of subjective, psychological, and social factors, as the model of best fit. Internal consistency reliabilities of the subscales exceeded .88. Moderate positive correlations were found between Sport MHC-SF subscales and quality of life indices, notably physical and emotional quality of life, demonstrating convergent validity. The Sport MHC-SF will facilitate empirical research by providing a more accurate and comprehensive measurement of well-being for an athletic population.
311	Emotional Intelligence (EI) Training Adapted to the International Preparation Constraints in Rugby: Influence of EI Trainer Status on EI Training Effectiveness	Campo, M; Laborde, S; Martinent, G; Louvet, B; Nicolas, M	FRONTIERS IN PSYCHOLOGY	2019	10.3389/fpsyg.2019.01939	S1+S3	WoS	Given the positive influence of emotional intelligence (EI) on sports performance, particular attention should be paid on how to improve it. Following promising results, previous research concluding that it was possible to improve EI via specific training programs also raised considerable debates. Indeed, previous EI training programs were very time-consuming for participants. This lessens consequently their suitability with the schedule constraints of elite sport. While, in the absence of sport psychologists, numerous coaches or physiologists try to work with players to improve their emotional competences, the aim of this study was to investigate the effectiveness of EI training programs fitting the schedule constraints of elite team sports, provided by three different EI trainers: the team's coach, the team's physiotherapist, and an expert in sport psychology. Young elite rugby union players (N = 96) participated in this study. Based on schedule constraints imposed by the head coach of the French u18 rugby union national team, the program consisted in three 1 h group-based EI training sessions occurring the last 3 days before a game (1 per day). Linear mixed-effects models showed that despite the constraining organizational challenge imposed by the coach, the intervention helped the players to increase some emotional competences at the trait level. Furthermore, a pairwise analysis showed that the type of emotional competencies developed depended on the status of the EI trainers. These findings highlight the suitability of a group-based approach in the training-week structure. They also point the way to EI improvement in a short period of time. Moreover, the specific influences of the EI trainer's status on players' EI development invite coaches and researchers to jointly combine their efforts in order to increase the EI training opportunities and to maximize the effects of their interventions. Together, these preliminary results provide first evidence facilitating the integration of such work in the preparation periods during international seasons.
312	A practitioner's perspective on psychological issues in football	Heidari, J; Pelka, M; Beckmann, J; Kellmann, M	SCIENCE AND MEDICINE IN FOOTBALL	2019	10.1080/24733938.2018.1526397	S2	WoS	The challenges for professional football players are becoming more and more complex due to the multifaceted physical, technical, and psychological demands of the modern game. Psychological components have especially attracted the attention of science and are finding their way into practice. This overview article aims to discuss five psychological issues in football which the authors consider to be among the most relevant for sport-psychological practitioners. Each topic is structured identically: (a) topic description, (b) empirical evidence highlighting its relevance in football and potential impacts on performance, and (c) practical recommendations. The first two topics focus on the environment in football by discussing (1) organisational issues and (2) player-coach-parent triads within football teams. Subsequently, the central theme performance is evaluated. (3) How do health and well-being impact long-term elite performance? (4) How can the training of psychological skills enhance performance? (5) How does choking under pressure affect performance? It can be stated that sport-psychological football research has provided several important and meaningful findings so far which can partly be adapted to practical settings. An even stronger focus of research in applied settings is warranted to gain further insights into the psychological underpinnings prevalent in the world of football. From a practical perspective, the article may provide both researchers and practitioners with valuable input on selected psychological topics in football. The summarised research can serve as a starting point for further research or interventions in practical settings.
313	The Coach-Athlete Relationship and Athlete Psychological Outcomes	McGee, V; Defreese, JD	JOURNAL OF CLINICAL SPORT PSYCHOLOGY	2019	10.1123/jcsp.2018-0010	S3	SPORTDiscus+WoS	This study examined associations among athlete perceptions of the coach-athlete relationship and psychological outcomes of burnout and engagement across a competitive season. We hypothesized that higher levels of the three markers of the coach-athlete relationship (closeness, commitment, and complementarity) would be negatively associated with burnout and positively associated with engagement across a sport season. American female collegiate rowers (N = 37; M-age = 19.3 years, SD = 1.18) completed online self-report assessments of study variables at four seasonal survey waves. Multilevel linear modeling analyses revealed closeness to be a significant predictor of seasonal global burnout and engagement across the season assessment period. Findings inform future research and intervention to promote engagement and deter burnout via improving coach-athlete closeness.

314	Communication Strategies: The Fuel for Quality Coach-Athlete Relationships and Athlete Satisfaction	Davis, L; Jowett, S; Tafvelin, S	FRONTIERS IN PSYCHOLOGY	2019	10.3389/fpsyg.2019.02156	S3	WoS	The present two-study paper examined the role of communication strategies that athletes use to develop their coach-athlete relationship. Study 1 examined the mediating role of motivation, support, and conflict management strategies between the quality of the coach-athlete relationship and athletes' perceptions of sport satisfaction. Study 2 examined the longitudinal and mediational associations of communication strategies and relationship quality across two time points, over a 6-week period. Within both studies, data were collected through multi-section questionnaires assessing the studies' variables. For study 1, structural equation modeling highlighted significant indirect effects for motivation and support strategies between the quality of the coach-athlete relationship and athletes' experiences of sport satisfaction. For study 2, significant indirect effects were found for the athletes' perceptions of the quality of the coach-athlete relationship at time 2 between athletes' use of communication strategies at time point 1 and time point 2. Together these findings provide support for the practical utility of communications strategies in enhancing the quality of the coach-athlete relationship and athlete's experiences of sport satisfaction. In addition, the findings provide evidence to highlight the potential cyclical relationship between communication and relationship quality across time.
315	Development of Perfectionism in Junior Athletes: A Three-Sample Study of Coach and Parental Pressure	Madigan, DJ; Curran, T; Stoeber, J; Hill, AP; Smith, MM; Passfield, L	JOURNAL OF SPORT & EXERCISE PSYCHOLOGY	2019	10.1123/jsep.2018-0287	S3	Scopus+WoS	Perfectionism predicts cognitions, emotions, and behaviors in sport. Nonetheless, our understanding of the factors that influence its development is limited. The authors sought to address this issue by examining the role of coach and parental pressure in the development of perfectionism in sport. Using 3 samples of junior athletes (16-19 years; cross-sectional n = 212, 3-month longitudinal n = 101, and 6-month longitudinal n = 110), the authors examined relations between coach pressure to be perfect, parental pressure to be perfect, perfectionistic strivings, and perfectionistic concerns. Mini meta-analysis of the combined cross-sectional data (N = 423) showed that both coach pressure and parental pressure were positively correlated with perfectionistic strivings and perfectionistic concerns. In contrast, longitudinal analyses showed that only coach pressure predicted increased perfectionistic strivings and perfectionistic concerns over time. Overall, our findings provide preliminary evidence that coaches may play a more important role in the development of junior athletes' perfectionism than parents.
316	Coach-Created Motivational Climate and Athletes' Adaptation to Psychological Stress: Temporal Motivation-Emotion Interplay	Ruiz, MC; Robazza, C; Tolvanen, A; Haapanen, S; Duda, JL	FRONTIERS IN PSYCHOLOGY	2019	10.3389/fpsyg.2019.00617	S3	SPORTDiscus+WoS	This two-wave study investigated the temporal interplay between motivation and the intensity and reported impact of athletes' emotions in training settings. In total, 217 athletes completed self-report measures of motivational climate, motivation regulations, emotional states (i.e., pleasant states, anger, and anxiety) experienced before practice at two time points during a 3-month period. Latent change score modeling revealed significantly negative paths from task-involving climate at time 1 to the latent change in the intensity of dysfunctional anxiety and anger, and significantly positive paths from ego-involving climate at time 1 to the latent change in dysfunctional anger (i.e., intensity and reported impact). The paths from controlled motivation at time 1 to the latent change in the intensity of dysfunctional anxiety and vice versa were significantly positive. The path from controlled motivation at time 1 to the latent change in the intensity of functional anger was significantly positive, but not vice versa. In addition, the paths from dysfunctional anger (i.e., intensity and reported impact) at time 1 to the latent change in motivation regulations were significant, but not vice versa. Overall, evidence provided suggested that the temporal interplay of motivation and emotions is contingent on the specific emotions. The findings highlight the role of coach-created motivational climate and the importance of identifying high levels of controlled motivation to help athletes better adapt to psychological stress.
317	Coaches' Emotional Intelligence and Reactive Behaviors in Soccer Matches: Mediating Effects of Coach Efficacy Beliefs	Teques, P; Duarte, D; Viana, J	FRONTIERS IN PSYCHOLOGY	2019	10.3389/fpsyg.2019.01629	S3	WoS	In the last 10 years, emotional intelligence (EI) has become a current issue of research in psychology, and there are indicators to consider that EI should be analyzed to help the coach to behave effectively during competitions. According to Boardley's (2018) revised model of coaching efficacy, coaches' EI is predictive of their efficacy beliefs, which, in turn, is predictive of coaching behavior. However, little is known about the mediating effects of coaching efficacy dimensions on the relationships between coach's EI and reactive behaviors in competitive settings. Thus, the purpose of this study is to examine mediating effects of coaching efficacy dimensions on the relationship between EI and coaches' reactive behaviors during a game using a multimethod approach. Participants were 258 coaches of youth football players aged 9 to 17 years old. Observations in situ using Coaching Behavior Assessment System (CBAS) were carried on 258 football games during two seasons. At the end of each game, coaches completed the Wong and Law Emotional Intelligence Scale (WLEIS) and the Coaching Efficacy Scale (CES). Structural equation modeling (SEM) analyses revealed that motivation efficacy and character building mediated the relationship between regulation of emotion and positive and negative coaches' reactions during game. Specifically, motivation efficacy mediated the association between regulation of emotion and positive coaches' reactions, and the relationship between regulation of emotion and negative coaches' reactions were mediated by motivation efficacy and character building. In addition, coaching level moderated the relationships between EI, self-efficacy and coaches' reactive behaviors. Findings of the present study showed that coaching efficacy dimensions (i.e., motivation efficacy and character building) that have the capacity to influence their confidence in ability to affect the psychological mood and positive attitude of athletes, transfer the effects of EI (i.e., regulation of emotion) on coaches' verbal reactions during a youth soccer game. Specifically, a coach who feels competent to regulate their own emotions would perceive high beliefs of efficacy to motivate and to build character of their athletes, and this insight has an impact on their positive verbal reactions in response to athletes' performances.

318	Dual Career of the U-23 Spanish Canoeing Team	Gavala-González, J; Castillo-Rodríguez, A; Fernández-Garcia, JC	FRONTIERS IN PSYCHOLOGY	2019	10.3389/fpsyg.2019.01783	S3	WoS	Canoeing is one of the sport disciplines that brings great success to Spain in international competitions and Olympic Games (of the 17 medals won in Rio in 2016, four were in this sport, including three gold). However, the journey to become an elite athlete coincides in time with the challenge of pursuing an academic education, which often involves making difficult choices in the training-university dichotomy. The aim of this research was to ascertain how the Spanish under-23 calm water canoeing team perceives their athletic and academic careers. The present study was carried out with the Spanish under-23 flat water canoeing team, the step prior to competition at the highest level. The study sample comprised the whole population, namely the entire national team, made up of 21 athletes (11 women and 10 men) with a mean age of 20.57 +/- 2.64 years and 10.00 +/- 3.49 years of experience. These athletes are usually based at La Cartuja High Performance Center (Seville) and combine their sports activity with studying toward a university degree. A double qualitative and quantitative methodology was used. For the first of these, an interview script was elaborated based on the theoretical model by Wylleman et al. (2013). Applicable consents were requested from the Spanish Royal Canoeing Federation, coaches and paddlers. Interviews were carried out with each athlete twice: at the beginning and at the end of the season/academic year, which were recorded and subsequently tabulated and analyzed. Concerning quantitative methodology, the ESTPORT dual career questionnaire validated by Sanchez-Pato et al. (2016) was used. The results show that for all the paddlers canoeing is very important. It is typically complicated for them to attend classes, and subject planning is usually based on their training schedules. In the second/last part of the course, it is stressful for these athletes to combine both activities and some drop out of school. During the course, though, they appreciate having another activity that allows them to escape from the training routine. In addition, they miss not having an academic tutor to guide and advise them. These student-athletes are aware that their sport causes them to miss out on many moments with family and friends due to training or competing. However, at present this is offset, largely because of their high level of performance, which makes it easier for them to obtain scholarships that provide economic support.
319	Psychometric properties of the Brazilian version of the Coach-Athlete Relationship Questionnaire for coaches	Contreira, AR; do Nascimento, JRA; Pizzo, GC; Sitoe, SA; Moreira, CR; Jowett, S; Fiorese, L	INTERNATIONAL JOURNAL OF SPORTS SCIENCE & COACHING	2019	10.1177/1747954119832715	S3	SPORTDiscus+WoS	The aim of this study was to assess the psychometric properties of the Brazilian version of the Coach-Athlete Relationship Questionnaire (CART-Q) for coaches. Subjects were 173 coaches (20-65 years old) from both sexes, from individual and team sports, ranging from amateur to national level; the instruments were the Coach version of CART-Q translated and adapted to Portuguese and the Athletic Satisfaction Scale adapted for coaches. Data analysis consisted of confirmatory factor analysis, internal consistency (Cronbach's alpha and composite reliability), concurrent validity (Spearman correlation), and temporal stability (intraclass correlation coefficient, ICC). Confirmatory factor analysis revealed a model with 11 items presenting adequate fit (chi(2)/df = 2.68; comparative fit index = 0.92; goodness-of-fit index = 0.91; Tucker-Lewis index = 0.90; root mean square error of approximation = 0.10) and the existence of a second-order factor (coach-athlete relationship). The scale has shown satisfactory internal consistency (composite reliability > 0.70 and alpha > 0.70). Concurrent validity assessment presented significant relationships (r > 0.40) with all coach satisfaction variables and temporal stability after 15 days was also adequate (0.65 < ICC < 0.88). It was concluded that the Brazilian version of the CART-Q Coach is adequate to assess coaches' perception regarding the relationship with their athletes.
320	The roles of coaches, peers, and parents in athletes' basic psychological needs: A mixed-studies review	Chu, TL; Zhang, T	INTERNATIONAL JOURNAL OF SPORTS SCIENCE & COACHING	2019	10.1177/1747954119858458	S3	SPORTDiscus+WoS	The purposes of this mixed-studies review were to summarize (a) the social environments created by coaches, peers, and parents concurrently, (b) the relative influence of social agents in youth athletes' psychological needs, and (c) the emerging research gaps for future research in and practical implications for youth sport. Literature was searched in six databases, resulting in 20 final studies with 2851 participants. These studies were reviewed and synthesized based on the theoretical frameworks, research design, participants and sports, associations between social environments and psychological needs, data analyses, results, and limitations. Results suggest that coaches, peers, and parents serve different roles in athletes' psychological needs. Coaches are the most important social agent in influencing autonomy, while peers are the most important social agent in influencing competence and relatedness. Parental influence is the least influential but also least studied in current literature. More research, particularly studies that use mixed methods or longitudinal design across developmental periods, is needed to examine the relative influence of all three social agents in youth sport contexts.

321	The junior to senior transition: a narrative analysis of the pathways of two Swedish athletes	Franck, A; Stambulova, NB	QUALITATIVE RESEARCH IN SPORT EXERCISE AND HEALTH	2019	10.1080/2159676X.2018.1479979	S3	WoS	There are as many careers with various pathways as there are athletes, and the interest in the junior-to-senior transition (JST) stems from its particular importance for athletes' lives when aiming for the senior elite levels of their sports. This study is a follow-up of the quantitative longitudinal study that investigated the JST process in Swedish sport club athletes. The aim of this study was to explore two athletes' (pseudonyms Erik, the swimmer, and Jessika, the tennis player) JST transition pathways, emphasising psychosocial factors that were perceived as facilitating and debilitating the transition process. Narrative-type interviews were conducted, and the stories were analysed using the holistic-form structural analysis. The analysis provided a central storyline (performance narrative) that is similar for both athletes, and two side storylines: Erik's effort and relationship narrative and Jessika's injury and reorientation narrative. They shared psychosocial factors that were perceived as facilitating the transition process, including family, coaches and sport club environment. For Erik, the debilitating factors were the negative changes in the group and a poor relationship with the new coach. The debilitating factors that influenced Jessika's JST were the decrease/ loss of financial support and the challenge of facing younger opponents against whom she felt she shouldn't lose when making a comeback after an injury. After the JST, both Erik and Jessika changed their tracks in life, terminated their athletic careers and focused on pursuing higher education qualifications.
322	The Temporal Relationship Between Exercise, Recovery Processes, and Changes in Performance	Skorski, S; Mujika, I; Bosquet, L; Meeusen, R; Coutts, AJ; Meyer, T	INTERNATIONAL JOURNAL OF SPORTS PHYSIOLOGY AND PERFORMANCE	2019	10.1123/ijspp.2018-0668	S3	WoS	Physiological and psychological demands during training and competition generate fatigue and reduce an athlete's sport-specific performance capacity. The magnitude of this decrement depends on several characteristics of the exercise stimulus (eg, type, duration, and intensity), as well as on individual characteristics (eg, fitness, profile, and fatigue resistance). As such, the time required to fully recover is proportional to the level of fatigue, and the consequences of exercise-induced fatigue are manifold. Whatever the purpose of the ensuing exercise session (ie, training or competition), it is crucial to understand the importance of optimizing the period between exercise bouts in order to speed up the regenerative processes and facilitate recovery or set the next stimulus at the optimal time point. This implies having a fairly precise understanding of the fatigue mechanisms that contribute to the performance decrement. Failing to respect an athlete's recovery needs may lead to an excessive accumulation of fatigue and potentially nonfunctional overreaching or to maladaptive training. Although research in this area recently increased, considerations regarding the specific time frames for different physiological mechanisms in relation to exercise-induced fatigue are still missing. Furthermore, recommendations on the timing and dosing of recovery based on these time frames are limited. Therefore, the aim of this article is to describe time courses of recovery in relation to the exercise type and on different physiological levels. This summary supports coaches, athletes, and scientists in their decision-making process by considering the relationship of exercise type, physiology, and recovery.
323	RELATIONSHIP BETWEEN RUNNING ECONOMY AND KINEMATIC PARAMETERS IN LONG-DISTANCE RUNNERS	Pizzuto, F; de Oliveira, CF; Soares, TSA; Rago, V; Silva, G; Oliveira, J	JOURNAL OF STRENGTH AND CONDITIONING RESEARCH	2019	10.1519/JSC.0000000000003040	S3	WoS	The purpose of this study was to explore the relationship between running economy (RE) and sagittal, frontal, and transverse plane kinematic parameters in long-distance runners. A secondary purpose was to identify the kinematic predictors of RE during running at the lowest RE value, representing an individual's most efficient running intensity. Twenty recreational long-distance runners ran 3 submaximal stages on a treadmill (65, 75, and 85% of velocity at maximum oxygen consumption). Respiratory data were collected using a portable gas analysis system. Kinematics were gathered using passive retroreflective markers and 8 high-resolution infrared cameras to collect the respective trajectories. Hip, knee, and ankle angles at foot strike and stance phase, as well as spatio-temporal parameters were calculated during each gait cycle. Knee flexion/extension range of motion (ROM), knee ab/adduction ROM, and hip ab/adduction ROM during the stance phase of the gait cycle showed positive moderate to large correlations with RE (r +/- 90% confidence intervals = 0.51 +/- 0.29; 0.49 +/- 0.30; 0.53 +/- 0.28, respectively). Knee and hip ab/adduction ROMs during the stance phase are predictors of RE, accounting for 44% of RE variance. Therefore, sagittal and frontal plane kinematics affect RE-inducing alterations in running performance. Coaches, athletic trainers, and anyone involved in running training prescription should consider a relationship between these parameters to ensure optimal technique and, consequently, to improve RE in recreational long-distance runners.
324	Identity salience and the football self: a critical ethnographic study of women and girls in football	Pielichaty, H	QUALITATIVE RESEARCH IN SPORT EXERCISE AND HEALTH	2019	10.1080/2159676X.2018.1549094	S3	WoS	The focus on identity has enjoyed a certain permanency within sports studies research, and the relationship between athlete identity and sport is complex and nuanced. Ordinarily literature on girls' and women's football (soccer) tends to focus on gender identities rather than identities per se. The purpose of the current paper is to address this issue and examine the way in which player identities are negotiated within the game. A season-long critical ethnographic exploration undertaken across three sites (football academy, school and family homes) underpins the research design for this exploration. It was found that the football self is managed and organised differently depending on the centrality of football within the lives of the women and girls who play. The development of the Football Self Continuum examines identity shifts vis-a-vis life transitions and football commitment. Identities are viewed in both hierarchical and fluid terms dependent on whether the player has a 'salient' or 'contingent' football self. This research has useful implications for coaches, family members and National Governing Bodies with regard to understanding and therefore supporting player identity changes in relation to life transitions.

325	When You Watch Your Team Fall Apart - Coaches' and Sport Psychologists' Perceptions on Causes of Collective Sport Team Collapse	Wergin, VV; Mallett, CJ; Mesagno, C; Zimanyi, Z; Beckmann, J	FRONTIERS IN PSYCHOLOGY	2019	10.3389/fpsyg.2019.01331	S3	WoS	Collective team collapse occurs when multiple players of a sport team experience a sudden and extreme underperformance within a game and are unable to return to their initial performance level. The occurrence of such a team collapse event commonly leads to the loss of the game or championship. A recent study investigated athletes' perceptions of the phenomenon and proposed a process model of causes of collective sport team collapse. The main goal of this study was to apply this process model to the data collected from coaches and sport psychologists. A further goal was to explore differences in perceptions of causes of team collapse among athletes, coaches, and sport psychologists of various professional German sport teams. Semi-structured interviews were conducted to investigate seven coaches' and four sport psychologists' perceptions. Following an abductive approach, a deductive content analysis was used to explore if the data supported the process model of collective sport team collapse. Perceived antecedents and critical events causing team collapse were similar among the three participant groups. Coaches and sport psychologists differed from athletes in their perception of emotional, cognitive, and behavioral outcomes of team collapse. Coaches tended to report behavioral factors, such as immobility or the blaming of other players, as critical factors maintaining team collapse. Sport psychologists reported cognitive factors, such as individualization or a lack of accountability between the players, to be relevant for team collapse maintenance. Overall, the data of this study supported the general structure of the process model of collective sport team collapse; however, minor amendments to the temporal cascade of causes of team collapse are introduced. Future research is encouraged to examine this model, to provide guidance to teams, coaches, and sport psychologists in dealing with collective sport team collapse.
326	Self-regulatory skills: Are they helpful in the prevention of overuse injuries in talented tennis players?	van der Sluis, A; Brink, MS; Pluim, BM; Verhagen, EALM; Elferink-Gemser, MT; Visscher, C	SCANDINAVIAN JOURNAL OF MEDICINE & SCIENCE IN SPORTS	2019	10.1111/sms.13420	S3	WoS	Talented athletes use metacognitive skills to improve their performance. Also, it is known that these skills are important for managing one's health. The goal of this study was to identify the relationship between metacognitive skills and overuse injuries in talented tennis players. Metacognitive skills were measured in 73 talented tennis players (45 boys and 28 girls, age 11-14) at the start of the season, using the Self-Regulation of Learning Self-Report Scale. Overuse injuries were monitored for one season using the Oslo Sports Trauma Research Centre Questionnaire on Health Problems. Ordinal regression indicated that moderate or low selfmonitoring skills (compared to high selfmonitoring) (OR 4.555, CI 1.096-18.927, P = 0.037) and exposure time (OR 1.380, CI 1.106-1.721, P = 0.004) were associated with more time loss overuse injuries. A second analysis showed that this was the case in girls (OR 10.757, CI 1.845-62.714, P = 0.008), but not in boys. Linear regression revealed that higher reflection scores and exposure time predicted overuse severity (F(5,58) = 2.921, P = 0.020, R-2 = 0.201). Possibly, selfmonitoring can help players to prevent themselves from time loss overuse injuries. Coaches should be aware that players can differ in selfmonitoring ability and thus in the ability to prevent overuse injuries. The role of reflection needs more research.
327	Relationship between total hemoglobin mass and competitive performance in endurance athletes	Zelenkova, IE; Zotkin, SV; Korneev, PV; Koprov, SV; Grushin, AA	JOURNAL OF SPORTS MEDICINE AND PHYSICAL FITNESS	2019	10.23736/S0022-4707.18.07865-9	S3	WoS	BACKGROUND: Determine the relationships between total hemoglobin mass (tHb-mass), h emoglobin (Hb), hematocrit (Hct) and competitive performance. METHODS: The team of 35 elite endurance athletes (biathlon men [BM, N.= 12] & women [BW, N.= 10], long track speed-skating men [SM, N.= 7] and women [SW, N.= 6]) were examined before participate in seven different competitions (C1 through C7) during the 2012-2013 and 2013-2014 seasons. Total hemoglobin mass measurement and biochemical analysis were performed using carbon monoxide rebreathing technique 24 hours before the competition. RESULTS: In BM correlations were found between results of two 10-km sprint competitions and tHb-mass between C1 and C2 (r=-0.94 and r=0.87). Correlations were also found between tHb-mass and performance in C3 individual races for BM (16.765 km, r=-0.88; and 10-km sprint, r= 0.80) and BW (7.2 km, r=-0.79; and 10-km sprint, r=-0.81). In SM, a correlation was found between tHb-mass and performance (r=-0.83) in C5. In SM and SW correlations were seen between tHb-mass and performance (SM, 5,000-m; r=-0.80; SW, 3,000-m; r=-0.77) in C6. CONCLUSIONS: No correlation was found between performance and Hb or Hct in any group. The demonstration of significant and direct correlation between tHb-mass and competitive performance highlights the importance of tHb-mass in this context and may be relevant for coaches providing one more piece of information for training.

328	Coaches' interpersonal emotion regulation and the coach-athlete relationship.	Campo, Mickaël ; Braun, Courtney ; Tamminen, Katherine A.	Movement & Sport Sciences / Science & Motricité	2019	10.1051/sm/2019011	S3	SPORTDiscus	Researchers have examined the impact of coaches' emotional expressions and emotional intelligence on athlete outcomes (Allan, V., & Côté, J. (2016). A cross-sectional analysis of coaches' observed emotion-behavior profiles and adolescent athletes' self-reported developmental outcomes. Journal of Applied Sport Psychology,28, 321–337; Thelwell, R.C., Lane, A.M., Weston, N.J., & Greenlees, I.A. (2008). Examining relationships between emotional intelligence and coaching efficacy. International Journal of Sport and Exercise Psychology,6, 224–235; van Kleef, G.A., Cheshin, A., Koning, L.F., & Wolf, S.A. (2018). Emotional games: How coaches' emotional expressions shape players' emotions, inferences, and team performance. Psychology of Sport & Exercise). However, there is little research examining coaches' use of specific strategies to regulate their athletes' emotions. The purpose of the present study was to explore the strategies coaches used to try and regulate their athletes' emotions, and to explore the relationship and contextual factors influencing coaches' IER strategy use. A longitudinal multiple case study approach was used (Stake, R.E. (2006). Multiple case study analysis. New York: The Guilford Press) with five cases, each consisting of one male coach and two individual varsity sport athletes (N = 15). Participants completed individual interviews, a two-week audio diary period, and a follow-up interview. Data were inductively and deductively analyzed and a conceptual model was developed outlining athletes' emotions and emotion regulation, coaches' IER, the coach-athlete relationship, and contextual factors. Participants described a bidirectional association between the coach-athlete relationship and coaches' IER. A number of factors influenced athletes' and coaches' use of emotion regulation strategies and contributed to the quality of the coach-athlete relationship. The IER strategies that coaches used may reflect instrumental, performance-related motives, and coaches' IER efforts may also contribute to coaches' emotional labour. ABSTRACT FROM AUTHOR
329	The development of a tournament preparation framework for competitive golf: A Delphi study	Pilgrim, J; Kremer, P; Robertson, S	EUROPEAN JOURNAL OF SPORT SCIENCE	2018	10.1080/17461391.2018.1469673	S2	Scopus+WoS	Tournament preparation in golf is used by players to increase course knowledge, develop strategy, optimise playing conditions and facilitate self-regulation. It is not known whether specific behaviours in tournament preparation should be given priority in education and practice at different stages of competition. This study aimed to achieve consensus on the importance of specific tournament preparation behaviours or items to players of five competitive levels. A two-round Delphi study was used, including an expert panel of 36 coaches, high-performance staff, players and academics. Participants were asked to score the relative importance of 48 items to players using a 5-point Likert-type scale. For an item to achieve consensus, 67% agreement was required in two adjacent score categories. Consensus was reached for 46 items and these were used to develop a ranked framework for each competitive level. The developed framework provides consensus-based guidelines of the behaviours that are perceived as important in tournament preparation. This framework could be used by national sport organisations to guide the development of more comprehensive learning environments for players and coaches. It could also direct future studies examining the critical behaviours for golfers across different competitive levels.
330	Organizational stressors, social support, and implications for subjective performance in high-level sport	Arnold, R; Edwards, T; Rees, T	PSYCHOLOGY OF SPORT AND EXERCISE	2018	10.1016/j.psychsport.2018.08.010	S2	WoS	Objectives: Although much is now known about the role of social support in the competitive stress process, scholars have yet to examine this moderator in relation to organizational stress. The purpose of this study was to examine the relationship between perceived organizational stressors and subjective performance in sport, with particular focus on the potential moderating role of social support. Design and methods: Talented athletes (N = 122; 60 male; M-age = 20.50) completed questionnaires of perceived organizational stressors, social support, and subjective athletic performance. Results: In addition to evidence of main effects, analyses revealed four significant interactions which demonstrated that social support did act as a significant moderator of the relationship between organizational stressors and subjective performance. Contrary to the extant literature, however, the findings illustrated reverse buffering. Associations suggest that some dimensions of social support exacerbated rather than mitigated athletes' stress reactions (i.e. impaired performance) when encountering greater frequencies of organizational stressors. Conclusion: These findings not only advance theoretical understanding of the organizational stress process, but also present a number of significant implications for athletes, coaches, and applied practitioners aiming to enhance performance in pressurized and demanding situations. Specifically, recommendations are forwarded for practitioners to address coaching stressors and provide effective social support that is matched to the stressors that he or she encounters.

331	Emotional Intelligence, Motivational Climate and Levels of Anxiety in Athletes from Different Categories of Sports: Analysis through Structural Equations	Castro-Sánchez, M; Zurita-Ortega, F; Chacón-Cuberos, R; López-Gutiérrez, CJ; Zafra-Santos, E	INTERNATIONAL JOURNAL OF ENVIRONMENTAL RESEARCH AND PUBLIC HEALTH	2018	10.3390/ijerph15050894	S2	Scopus+WoS	(1) Background: Psychological factors can strongly affect the athletes' performance. Therefore, currently the role of the sports psychologist is particularly relevant, being in charge of training the athlete's psychological factors. This study aims at analysing the connections between motivational climate in sport, anxiety and emotional intelligence depending on the type of sport practised (individual/team) by means of a multigroup structural equations analysis. (2) 372 semi-professional Spanish athletes took part in this investigation, analysing motivational climate (PMCSQ-2), emotional intelligence (SSRI) and levels of anxiety (STAI). A model of multigroup structural equations was carried out which fitted accordingly ((2) = 586.77; df = 6.37; p < 0.001; Comparative Fit Index (CFI) = 0.951; Normed Fit Index (NFI) = 0.938; Incremental Fit Index (IFI) = 0.947; Root Mean Square Error of Approximation (RMSEA) = 0.069). (3) Results: A negative and direct connection has been found between ego oriented climate and task oriented climate, which is stronger and more differentiated in team sports. The most influential indicator in ego oriented climate is intra-group rivalry, exerting greater influence in individual sports. For task-oriented climate the strongest indicator is having an important role in individual sports, while in team sports it is cooperative learning. Emotional intelligence dimensions correlate more strongly in team sports than in individual sports. In addition, there was a negative and indirect relation between task oriented climate and trait-anxiety in both categories of sports. (4) Conclusions: This study shows how the task-oriented motivational climate or certain levels of emotional intelligence can act preventively in the face of anxiety states in athletes. Therefore, the development of these psychological factors could prevent anxiety states and improve performance in athletes.
332	Athletes' perceptions of coaching competency and team conflict in sport teams: A multilevel analysis	González-Ponce, I; Leo, FM; Jiménez, R; Sánchez-Oliva, D; Sarmento, H; Figueiredo, A; García-Calvo, T	EUROPEAN JOURNAL OF SPORT SCIENCE	2018	10.1080/17461391.2018.1461245	S3	WoS	The purpose of this study was to examine the relationship between coaching competency and team conflict, at individual and team levels, over the season. The participants were professional female and male soccer players, who participated in the First and Second Division. A longitudinal study was performed. At Time 1, the sample of participants consisted of 581 soccer players aged between 15 and 39 years. At Time 2, 549 players were recruited from the original sample aged between 15 and 37 years. Finally, at Time 3, the sample comprised 576 players aged between 15 and 37 years. All participants completed a multi-section questionnaire assessing coaching competency (motivation, game strategy, technique competency, and character-building competency) and team conflict (task conflict and relationship conflict). Results showed that both task and relationship conflict increased significantly over time. Multilevel modelling analysis showed that game strategy and character-building competencies negatively predicted both task and relationship conflicts at the individual level, whereas motivation competency was also added as a significant predictor of task conflict at the team level. Moreover, technique competency positively predicted task conflict at the team level. The current study suggests the importance of coaching competency in group dynamics in sport.
333	Monitoring Training Load and Well-Being During the In-Season Phase in National Collegiate Athletic Association Division I Men's Basketball	Conte, D; Kolb, N; Scanlan, AT; Santolamazza, F	INTERNATIONAL JOURNAL OF SPORTS PHYSIOLOGY AND PERFORMANCE	2018	10.1123/ijspp.2017-0689	S3	WoS	Purpose: To characterize the weekly training load (TL) and well-being of college basketball players during the in-season phase. Methods: Ten (6 guards and 4 forwards) male basketball players (age 20.9 [0.9] y, stature 195.0 [8.2] cm, and body mass 91.3 [11.3] kg) from the same Division I National Collegiate Athletic Association team were recruited to participate in this study. Individualized training and game loads were assessed using the session rating of perceived exertion at the end of each training and game session, and well-being status was collected before each session. Weekly changes (%) in TL, acute-to-chronic workload ratio, and well-being were determined. Differences in TL and well-being between starting and bench players and between 1-game and 2-game weeks were calculated using magnitude-based statistics. Results: Total weekly TL and acute-to-chronic workload ratio demonstrated high week-to-week variation, with spikes up to 226% and 220%, respectively. Starting players experienced a higher (most likely negative) total weekly TL and similar (unclear) well-being status compared with bench players. Game scheduling influenced TL, with 1-game weeks demonstrating a higher (likely negative) total weekly TL and similar (most likely trivial) well-being status compared with 2-game weeks. Conclusions: These findings provide college basketball coaches information to optimize training strategies during the in-season phase. Basketball coaches should concurrently consider the number of weekly games and player status (starting vs bench player) when creating individualized periodization plans, with increases in TL potentially needed in bench players, especially in 2-game weeks.
334	Self-Talk and Competitive Balance Beam Performance	Van Dyke, ED; Van Raalte, JL; Mullin, EM; Brewer, BW	SPORT PSYCHOLOGIST	2018	10.1123/tsp.2016-0085	S3	Scopus+WoS	Little research has explored the relationship between highly skilled athletes' self-talk and their competitive performance over the course of a season. For the current study, positive, negative, motivational, instructional, and functional dimensions of collegiate gymnasts' (N = 141) self-talk were assessed. The gymnasts' competitive balance beam performances in intercollegiate meets were also recorded. Multiple regression analysis revealed that positive self-talk significantly predicted balance beam performance and performance consistency. Significant positive correlations were found among key self-talk variables, except negative self-talk. Significant negative correlations were found between negative self-talk and self-talk functions (i.e., attention, cognitive and emotional control, and confidence). The results highlight the interrelationships among various types and functions of self-talk in competitive settings, and provide evidence for the ways in which self-talk is related to the performance of highly skilled athletes. Suggestions for how these findings might be applied by athletes, coaches, and sport psychology practitioners are provided.

335	Neophyte experiences of football (soccer) match analysis: a multiple case study approach	McKenna, M; Cowan, DT; Stevenson, D; Baker, JS	RESEARCH IN SPORTS MEDICINE	2018	10.1080/15438627.2018.1447473	S3	WoS	Performance analysis is extensively used in sport, but its pedagogical application is little understood. Given its expanding role across football, this study explored the experiences of neophyte performance analysts. Experiences of six analysis interns, across three professional football clubs, were investigated as multiple cases of new match analysis. Each intern was interviewed after their first season, with archival data providing background information. Four themes emerged from qualitative analysis: (1) building of relationships was important, along with trust and role clarity; (2) establishing an analysis system was difficult due to tacit coach knowledge, but analysis was established; (3) the quality of the feedback process hinged on coaching styles, with balance of feedback and athlete engagement considered essential; (4) establishing effect was complex with no statistical effects reported; yet enhanced relationships, role clarity, and improved performances were reported. Other emic accounts are required to further understand occupational culture within performance analysis.
336	Talent Identification and Development in Male Football: A Systematic Review	Sarmento, H; Anguera, MT; Pereira, A; Araújo, D	SPORTS MEDICINE	2018	10.1007/s40279-017-0851-7	S3	WoS	Background Expertise has been extensively studied in several sports over recent years. The specificities of how excellence is achieved in Association Football, a sport practiced worldwide, are being repeatedly investigated by many researchers through a variety of approaches and scientific disciplines. Objective The aim of this review was to identify and synthesise the most significant literature addressing talent identification and development in football. We identified the most frequently researched topics and characterised their methodologies. Methods A systematic review of Web of Science (TM) Core Collection and Scopus databases was performed according to PRISMA (Preferred Reporting Items for Systematic Reviews and Meta-analyses) guidelines. The following keywords were used: football and soccer. Each word was associated with the terms talent, expert*, elite, elite athlete, identification, career transition or career progression. The selection was for the original articles in English containing relevant data about talent development/identification on male footballers. Results The search returned 2944 records. After screening against set criteria, a total of 70 manuscripts were fully reviewed. The quality of the evidence reviewed was generally excellent. The most common topics of analysis were (1) task constraints: (a) specificity and volume of practice; (2) performers' constraints: (a) psychological factors; (b) technical and tactical skills; (c) anthropometric and physiological factors; (3) environmental constraints: (a) relative age effect; (b) socio-cultural influences; and (4) multidimensional analysis. Results indicate that the most successful players present technical, tactical, anthropometric, physiological and psychological advantages that change non-linearly with age, maturational status and playing positions. These findings should be carefully considered by those involved in the identification and development of football players. Conclusion This review highlights the need for coaches and scouts to consider the players' technical and tactical skills combined with their anthropometric and physiological characteristics scaled to age. Moreover, research addressing the psychological and environmental aspects that influence talent identification and development in football is currently lacking. The limitations detected in the reviewed studies suggest that future research should include the best performers and adopt a longitudinal and multidimensional perspective.
337	When Suddenly Nothing Works Anymore Within a Team - Causes of Collective Sport Team Collapse	Wergin, VV; Zimanyi, Z; Mesagno, C; Beckmann, J	FRONTIERS IN PSYCHOLOGY	2018	10.3389/fpsyg.2018.02115	S3	WoS	Collective team collapse occurs when multiple players of a sport team experience a sudden and extreme underperformance within a game. To date, minimal research has been conducted on the causes of collective team collapse. Thus, goals of this study were to explore perceived causes of collective team collapse in different sports and to define team collapse in contrast to negative momentum. To investigate factors causing and maintaining collective sport team collapse, an inductive, exploratory qualitative analysis of individual interviews was conducted. Semi-structured interviews were carried out with 10 athletes of professional German teams of various sports playing in between first and fourth division. Participants were interviewed about a team collapse event they had experienced with their team during the past year. Data were collected and analyzed using a grounded theory methodology. Collective team collapse appeared to be induced by a temporal cascade of causes rather than by single triggers. This cascade included antecedents, which represent factors that make the occurrence of a team collapse more likely; critical events, which include specific events within the game that trigger a team collapse; as well as affective, cognitive, and behavioral outcomes that foster a maintenance of the collapse. Within this theoretical framework, social factors, such as decreased performance contagion or emotional contagion, played crucial roles in causing a team collapse. These results illustrate that collective team collapse is more than the sum of individual choking of multiple players at the same time. In conclusion, a new definition, differentiating team collapse from negative momentum, is introduced. Furthermore, a process model of causes of collective team collapse is proposed. The results provide first insights into causes of collective collapse in a variety of team sports. The developed model is supposed to help future research to better connect to practice and to support athletes, coaches, and sport psychologists.

338	An Integrated, Multifactorial Approach to Periodization for Optimal Performance in Individual and Team Sports	Mujika, I; Halson, S; Burke, LM; Balagué, G; Farrow, D	INTERNATIONAL JOURNAL OF SPORTS PHYSIOLOGY AND PERFORMANCE	2018	10.1123/ijspp.2018-0093	S3	WoS	Sports periodization has traditionally focused on the exercise aspect of athletic preparation, while neglecting the integration of other elements that can impact an athlete's readiness for peak competition performances. Integrated periodization allows the coordinated inclusion of multiple training components best suited for a given training phase into an athlete's program. The aim of this article is to review the available evidence underpinning integrated periodization, focusing on exercise training, recovery, nutrition, psychological skills, and skill acquisition as key factors by which athletic preparation can be periodized. The periodization of heat and altitude adaptation, body composition, and physical therapy is also considered. Despite recent criticism, various methods of exercise training periodization can contribute to performance enhancement in a variety of elite individual and team sports, such as soccer. In the latter, both physical and strategic periodization are useful tools for managing the heavy travel schedule, fatigue, and injuries that occur throughout a competitive season. Recovery interventions should be periodized (ie, withheld or emphasized) to influence acute and chronic training adaptation and performance. Nutrient intake and timing in relation to exercise and as part of the periodization of an athlete's training and competition calendar can also promote physiological adaptations and performance capacity. Psychological skills are a central component of athletic performance, and their periodization should cater to each athlete's individual needs and the needs of the team. Skill acquisition can also be integrated into an athlete's periodized training program to make a significant contribution to competition performance.
339	Leveling the playing field: can psychological coping resources reduce the influence of physical and technical skills on athletic performance?	Christensen, DS; Smith, RE	ANXIETY STRESS AND COPING	2018	10.1080/10615806.2018.1506646	S3	Scopus+WoS	Background and Objectives: The present study assessed the extent to which individual differences in psychological coping resources are related to athletic performance; whether they can attenuate the amount of performance variance accounted for by physical/technical skills; and whether coping resources remain significant predictors of performance when physical/technical skill level is statistically controlled. Methods: Twenty college golf coaches rated the physical/technical skills of 189 men and women varsity golfers on their teams. Athletes completed the Athletic Coping Skills Inventory (ACSI-28), with the total (Personal Coping Resources) score serving as a global measure of sport-relevant psychological coping resources. Subsequent performance (stroke average per round) of 105 golfers was assessed over a mean of 12.04 competitive rounds during the remainder of the season. Results: Physical/technical skills and psychological coping resource measures were minimally correlated and both were significant and similarly influential predictors of performance. With psychological resources controlled, performance variance accounted for by physical/technical skills was reduced from 21.2% to 10.6%. With physical/technical skills statistically controlled, psychological coping resources, though reduced from 18.2% to 7.5% of accountable variance, remained a significant predictor of performance. Conclusion: Results support the significant role played by psychological coping resources as predictors of athletic performance, together with their ability to level the field by reducing the influence of physical/technical talent.
340	A test of basic psychological needs theory in young soccer players: time-lagged design at the individual and team levels	González, L; Tomás, I; Castillo, I; Duda, JL; Balaguer, I	SCANDINAVIAN JOURNAL OF MEDICINE & SCIENCE IN SPORTS	2017	10.1111/sms.12778	S3	WoS	Within the framework of basic psychological needs theory (Deci & Ryan, 2000), multilevel structural equation modeling (MSEM) with a time-lagged design was used to test a mediation model examining the relationship between perceptions of coaches' interpersonal styles (autonomy supportive and controlling), athletes' basic psychological needs (satisfaction and thwarting), and indicators of well-being (subjective vitality) and ill-being (burnout), estimating separately between and within effects. The participants were 597 Spanish male soccer players aged between 11 and 14 years (M = 12.57, SD = 0.54) from 40 teams who completed a questionnaire package at two time points in a competitive season. Results revealed that at the individual level, athletes' perceptions of autonomy support positively predicted athletes' need satisfaction (autonomy, competence, and relatedness), whereas athletes' perceptions of controlling style positively predicted athletes' need thwarting (autonomy, competence, and relatedness). In turn, all three athletes' need satisfaction dimensions predicted athletes' subjective vitality and burnout (positively and negatively, respectively), whereas competence thwarting negatively predicted subjective vitality and competence and relatedness positively predicted burnout. At the team level, team perceptions of autonomy supportive style positively predicted team autonomy and relatedness satisfaction. Mediation effects only appeared at the individual level.

341	Stressors, coping, and well-being among sports coaches: A systematic review	Norris, LA; Didymus, FF; Kaiseler, M	PSYCHOLOGY OF SPORT AND EXERCISE	2017	10.1016/j.psychsport.2017.08.005	S3	Scopus+WoS	Objectives: Sports coaching can be an inherently stressful occupation because coaches must fulfill multiple roles and cope with various expectations. Further, stress and well-being have implications for coach performance. The objective of this study was, therefore, to conduct a systematic review of literature on stressors, coping, and well-being among sports coaches. Design: A systematic review using PRIMSA guidelines. Method: Thorough and systematic literature searches of PsycINFO, SPORTDiscus, and Web of Science were conducted. To be eligible for inclusion, papers had to be published in the English language between January 1994 and March 2016 and as full papers in peer-reviewed journals. Results: The final sample consisted of 38 studies that were conducted with 4188 sports coaches. This sample consisted of 19 qualitative, 17 quantitative, and two mixed methods studies. The findings demonstrate that coaches experience a variety of stressors relating to their performance and that of the athletes they work with in addition to organizational, contextual, interpersonal, and intrapersonal stressors. The findings also highlight that coaches use a variety of coping strategies (e.g., problem solving, social support, escaping the stressful environment) to reduce the negative outcomes of stress. Five studies that were included in this review focused on coaches' well-being and found that basic psychological needs satisfaction, lack of basic psychological needs thwarting, and self-determined motivation are needed for coaches to be psychologically well. Conclusion: Future research should address gaps in extant literature by using longitudinal study designs to explore coaches' appraisals of stressors, coping effectiveness, social support, and well-being among the unique sports coaching population. (C) 2017 Elsevier Ltd. All rights reserved.
342	A Self-Determination Theory Perspective on Attachment, Need Satisfaction, and Well-Being in a Sample of Athletes: A Longitudinal Study	Felton, L; Jowett, S	JOURNAL OF CLINICAL SPORT PSYCHOLOGY	2017	10.1123/jcsp.2016-0013	S3	WoS	The current study aimed to examine whether (a) mean differences and changes in athletes' attachment style predicted psychological need satisfaction within two diverse relational contexts (coach and parent) and well-being, and (b) mean differences and changes in need satisfaction within the two relational contexts predicted well-being. One hundred and ten athletes aged between 15 and 32 years old completed a multisection questionnaire at three time points over a span of 6 months to assess the main study variables. Multilevel modeling revealed that insecure attachment styles (anxious and avoidant) predicted well-being outcomes at the within-and between-person levels. Avoidant attachment predicted need satisfaction within the parent relational context at both levels, and need satisfaction within the coach relational context at the between-person level. Need satisfaction within both relational contexts predicted various well-being outcomes at the between-person level, while need satisfaction within the parent relational context predicted vitality at the within-person level.
343	Longitudinal associations between athletes' controlled motivation, ill being, and perceptions of controlling coach behaviors: A Bayesian latent growth curve approach	Stenling, A; Ivarsson, A; Hassmén, P; Lindwall, M	PSYCHOLOGY OF SPORT AND EXERCISE	2017	10.1016/j.psychsport.2017.03.002	S3	WoS	Although many scholars have argued that leadership is a dynamic process jointly produced by leaders and followers, leadership in sports is most often researched as a unidirectional process from coaches to athletes. Within self-determination theory (SDT), individual characteristics are suggested to influence how people perceive external events such as coaches' behaviors. In the present study, we examined this jointly produced leadership process by investigating longitudinal associations between athletes' controlled motivation, ill-being, and perceptions of coaches' controlling behaviors at the between- and within-person levels. The participants were 247 young elite skiers enrolled at Swedish sport high schools who responded to self-report questionnaires at three time points over the course of an athletic season. At the between-person level, increases in perceptions of coaches' controlling behaviors over the season positively predicted controlled motivation at the end of the season, and controlled motivation at the beginning of the season positively predicted ill-being at the end of the season. At the within-person level, athletes' controlled motivation positively predicted perceptions of coaches' controlling behaviors. The results at the between-person level support the unidirectional perspective and the tenets of SDT. The results at the within-person level suggest that individual characteristics such as motivation can influence how athletes perceive external events, which has been proposed theoretically but seldom examined empirically. Three plausible explanations for this reversed association are presented in the discussion. (C) 2017 Elsevier Ltd. All rights reserved.

344	The two roads from passion to sport performance and psychological well-being: The mediating role of need satisfaction, deliberate practice, and achievement goals	Verner-Filion, J; Vallerand, RJ; Amiot, CE; Mocanu, I	PSYCHOLOGY OF SPORT AND EXERCISE	2017	10.1016/j.psychsport.2017.01.009	S3	Scopus+WoS	Objectives: Research on passion has demonstrated the existence of two roads toward sports performance through the effects of deliberate practice (Vallerand et al., 2007, 2008). The first emanates from harmonious passion (HP) and contributes to both performance and psychological well-being. The second stems from obsessive passion (OP), and performance comes at the cost of well-being. The present research proposes that need satisfaction (Deci & Ryan, 2000) mediates the relation of HP, but not OP, with both outcomes. In Study 2, achievement goals were added to the model. Mastery goals were expected to mediate the positive relation between HP and outcomes, whereas performance-avoidance goals would be associated with OP and, thus be detrimental to athletes. Design: Two studies using correlational (Study 1) and longitudinal (Study 2) designs. Method: Study 1 (N = 172) was conducted with soccer players. Study 2 was conducted with hockey players (N = 598). Athletes completed measures of passion, need satisfaction, life satisfaction, deliberate practice, and achievement goals (Study 2 only). Coaches assessed performance in Study 1. Study 2 used games played in competitive leagues over 15 years to measure performance. Results: Analyses using SEM provided support for the mediating role of need satisfaction (Study 1 and 2) and achievement goals (Study 2) in the relation of HP with outcomes. In contrast, deliberate practice (Study 1 and 2) meditated the relation between OP and performance. Conclusions: This research supported the mediating role of need satisfaction in the 'two roads to performance (Vallerand et al., 2007, 2008). (C) 2017 Elsevier Ltd. All rights reserved.
345	Training Load and Fatigue Marker Associations with Injury and Illness: A Systematic Review of Longitudinal Studies	Jones, CM; Griffiths, PC; Mellalieu, SD	SPORTS MEDICINE	2017	10.1007/s40279-016-0619-5	S3	WoS	Background Coaches, sport scientists, clinicians and medical personnel face a constant challenge to prescribe sufficient training load to produce training adaption while minimising fatigue, performance inhibition and risk of injury/illness. Objective The aim of this review was to investigate the relationship between injury and illness and longitudinal training load and fatigue markers in sporting populations. Methods Systematic searches of the Web of Science and PubMed online databases to August 2015 were conducted for articles reporting relationships between training load/fatigue measures and injury/illness in athlete populations. Results From the initial 5943 articles identified, 2863 duplicates were removed, followed by a further 2833 articles from title and abstract selection. Manual searching of the reference lists of the remaining 247 articles, together with use of the Google Scholar 'cited by' tool, yielded 205 extra articles deemed worthy of assessment. Sixty-eight studies were subsequently selected for inclusion in this study, of which 45 investigated injury only, 17 investigated illness only, and 6 investigated both injury and illness. This systematic review highlighted a number of key findings, including disparity within the literature regarding the use of various terminologies such as training load, fatigue, injury and illness. Athletes are at an increased risk of injury/illness at key stages in their training and competition, including periods of training load intensification and periods of accumulated training loads. Conclusions Further investigation of individual athlete characteristics is required due to their impact on internal training load and, therefore, susceptibility to injury/illness.
346	Development and Validation of the Characteristics of Resilience in Sports Teams Inventory	Decroos, S; Morgan, PBC; Sarkar, M; Lines, RLJ; Fletcher, D; Fransen, K; Boen, F; Broek, GV	SPORT EXERCISE AND PERFORMANCE PSYCHOLOGY	2017	10.1037/spy0000089	S3	WoS	This multistudy paper reports the development and initial validation of an inventory for the Characteristics of Resilience in Sports Teams (CREST). In 4 related studies, 1,225 athletes from Belgium and the United Kingdom were sampled. The first study provided content validity for an initial item set. The second study explored the factor structure of the CREST, yielding initial evidence but no conclusive results. In contrast, the third and fourth study provided evidence for a 2-factor measure, reflecting (a) the team's ability to display resilient characteristics and (b) the vulnerabilities being displayed under pressure. Overall, the CREST was shown to be reliable at the between-players and the between-teams level, as well as over time. Moreover, its concurrent validity was verified by linking the characteristics of team resilience with various relevant team processes. Its discriminant validity was established by comparing the CREST measures with individual athletes' resilient traits. In conclusion, the CREST was argued to be a usable state-like measure of team-level resilient characteristics and vulnerabilities. To gain further understanding of team resilience as a process, this measurement could be used in future process-oriented research examining adverse events and sports team's pre- and postadversity functioning.
347	Linking Coach Interpersonal Style With Athlete Doping Intentions and Doping Use: A Prospective Study	Ntoumanis, N; Barkoukis, V; Gucciardi, DF; Chan, DKC	JOURNAL OF SPORT & EXERCISE PSYCHOLOGY	2017	10.1123/jsep.2016-0243	S3	Scopus+WoS	We brought together various lines of work on motivation, morality, and doping by testing a theory-based model prospectively linking contextual and personal motivational variables, moral attitudes, moral disengagement in doping, doping intentions, and doping use. Participants were 257 Greek athletes who completed a questionnaire pack at the beginning of a sport season. In the case of doping use, we also obtained data close to the end of the same season. The model showed that perceptions of controlling coach behaviors predicted athlete need frustration, which in turn predicted low moral functioning and doping intentions/doping use. The findings highlight pathways (direct and indirect) by which the social environment may impact on athletes' intentions and decisions to engage in doping and could pave the way for future antidoping interventions aimed at improving coaching interpersonal style.

348	The Internalisation of Sociocultural and Thin-Ideal Standards in Sports scale: Development and preliminary validation in female athletes participating in aesthetic sports	Scoffier-Mériaux, S; Ferrand, C; d'Arripe-Longueville, F	INTERNATIONAL JOURNAL OF SPORT AND EXERCISE PSYCHOLOGY	2017	10.1080/1612197X.2015.1122720	S3	WoS	Thinness is a part of today's sociocultural standards of beauty and thus is implicated in body-image dissatisfaction and eating disorders. In female aesthetic sports, thinness is a standard for aesthetics and performance. Measuring the internalisation of thin-ideal standards in these athletes may therefore help athletes, coaches, parents, administrators, sport psychologists, and so on to gain insight into the risks associated with body-image dissatisfaction and disordered eating. This study aimed to develop and validate the Internalisation of Sociocultural and Thin-Ideal Standards in Sports (ISTISS) scale. Four studies involving 357 female aesthetic athletes examined the exploratory factorial structure, temporal stability, and concurrent validity of the ISTISS, which gave rise to a 10-item scale with two subscales: internalisation of sociocultural standards and thin-ideal standards in sports. Internal consistencies were satisfactory and relationships with the self-regulation of eating attitude in sport were as expected. These findings provide preliminary evidence for the appropriateness of the ISTISS scale for female aesthetic sports athletes.
349	Long-Term Prognostic Validity of Talent Selections: Comparing National and Regional Coaches, Laypersons and Novices	Schorer, J; Rienhoff, R; Fischer, L; Baker, J	FRONTIERS IN PSYCHOLOGY	2017	10.3389/fpsyg.2017.01146	S3	WoS	In most sports, the development of elite athletes is a long-term process of talent identification and support. Typically, talent selection systems administer a multi-faceted strategy including national coach observations and varying physical and psychological tests when deciding who is chosen for talent development. The aim of this exploratory study was to evaluate the prognostic validity of talent selections by varying groups 10 years after they had been conducted. This study used a unique, multi-phased approach. Phase 1 involved players (n = 68) in 2001 completing a battery of general and sport-specific tests of handball ` talent' and performance. In Phase 2, national and regional coaches (n = 7) in 2001 who attended training camps identified the most talented players. In Phase 3, current novice and advanced handball players (n = 12 in each group) selected the most talented from short videos of matches played during the talent camp. Analyses compared predictions among all groups with a best modelfit derived from the motor tests. Results revealed little difference between regional and national coaches in the prediction of future performance and little difference in forecasting performance between novices and players. The best model-fit regression by the motor-tests outperformed all predictions. While several limitations are discussed, this study is a useful starting point for future investigations considering athlete selection decisions in talent identification in sport.
350	Phases in the junior-to-senior transition of Swedish ice hockey players: From a conceptual framework to an empirical model	Stambulova, NB; Pehrson, S; Olsson, K	INTERNATIONAL JOURNAL OF SPORTS SCIENCE & COACHING	2017	10.1177/1747954117694928	S3	WoS	The purpose of this study was to explore a temporal structure (i.e. sequence of phases and relevant psychological content) of the junior-to-senior transition in Swedish ice hockey players. The study was a qualitative exploration of the junior-to-senior transition process designed in three steps. First, the athletic career transition model coupled with a holistic developmental approach and analysis of the Swedish ice hockey context were used to create a conceptual four-phase (preparation, orientation, adaptation and stabilization) junior-to-senior transition framework and interview guides. Second, seven active semi-professional ice hockey players were interviewed about their junior-to-senior transition experiences and asked for their feedback on the conceptual framework. Finally, based on the empirical data and the participants' feedback, the conceptual framework was transformed into an empirical model of the junior-to-senior transition. In the empirical model Phases in the junior-to-senior transition of Swedish ice hockey players', the authors provided a summary of the players' transition experiences (i.e. perceived demands, resources, barriers, coping strategies and outcomes) within each of the four (i.e. the preparation, the orientation, the adaptation and the stabilization) junior-to-senior transition phases. Further, the authors discussed the empirical model in relation to theoretical frameworks and previous research and also provided methodology-, future research- and practice-oriented reflections addressed to researchers, coaches and sport psychology practitioners.
351	The Influence of Playing Experience and Position on Injury Risk in NCAA Division I College Football Players	McCunn, R; Fullagar, HHK; Williams, S; Halseth, TJ; Sampson, JA; Murray, A	INTERNATIONAL JOURNAL OF SPORTS PHYSIOLOGY AND PERFORMANCE	2017	10.1123/ijspp.2016-0803	S3	WoS	Purpose: American football is widely played by college student-athletes throughout the United States; however, the associated injury risk is greater than in other team sports. Numerous factors likely contribute to this risk, yet research identifying these risk factors is limited. The present study sought to explore the relationship between playing experience and position on injury risk in NCAA Division I college football players. Methods: Seventy-six male college student-athletes in the football program of an American NCAA Division I university participated. Injuries were recorded over 2 consecutive seasons. Players were characterized based on college year (freshman, sophomore, junior, or senior) and playing position. The effect of playing experience and position on injury incidence rates was analyzed using a generalized linear mixed-effects model, with a Poisson distribution, log-linear link function, and offset for hours of training exposure or number of in-game plays (for training and game injuries, respectively). Results: The overall rates of non-time-loss and time-loss game-related injuries were 2.1 (90% CI: 1.8-2.5) and 0.6 (90% CI: 0.4-0.8) per 1000 plays, respectively. The overall rates of non-time-loss and time-loss training-related injuries were 26.0 (90% CI: 22.6-29.9) and 7.1 (90% CI: 5.9-8.5) per 1000 h, respectively. During training, seniors and running backs displayed the greatest risk. During games, sophomores, juniors, and wide receivers were at greatest risk. Conclusions: Being aware of the elevated injury risk experienced by certain player groups may help coaches make considered decisions related to training design and player selection.

352	Updated Review of the Applied Physiology of American College Football: Physical Demands, Strength and Conditioning, Nutrition, and Injury Characteristics of America's Favorite Game	Fullagar, HHK; McCunn, R; Murray, A	INTERNATIONAL JOURNAL OF SPORTS PHYSIOLOGY AND PERFORMANCE	2017	10.1123/ijspp.2016-0783	S3	WoS	While there are various avenues for performance improvement in college American football (AF), there is no comprehensive evaluation of the collective array of resources around performance, physical conditioning, and injury and training/game characteristics to guide future research and inform practitioners. Accordingly, the aim of the present review was to provide a current examination of these areas in college AF. Recent studies show that there is a wide range of body compositions and strength characteristics between players, which appear to be influenced by playing position, level of play, training history/programming, and time of season. Collectively, game demands may require a combination of upper-and lower-body strength and power production, rapid acceleration (positive and negative), change of direction, high running speed, high-intensity and repetitive collisions, and muscle-strength endurance. These may be affected by the timing of and between-plays and/or coaching style. AF players appear to possess limited nutrition and hydration practices, which may be disadvantageous to performance. AF injuries appear due to a multitude of factors-strength, movement quality, and previous injury-while there is also potential for extrinsic factors such as playing surface type, travel, time of season, playing position, and training load. Future proof-of-concept studies are required to determine the quantification of game demands with regard to game style, type of opposition, and key performance indicators. Moreover, more research is required to understand the efficacy of recovery and nutrition interventions. Finally, the assessment of the relationship between external/internal-load constructs and injury risk is warranted.
353	Training loads and injury risk in Australian football-differing acute: chronic workload ratios influence match injury risk	Carey, DL; Blanch, P; Ong, KL; Crossley, KM; Crow, J; Morris, ME	BRITISH JOURNAL OF SPORTS MEDICINE	2017	10.1136/bjsports-2016-096309	S3	WoS	Aims (1) To investigate whether a daily acute: chronic workload ratio informs injury risk in Australian football players; (2) to identify which combination of workload variable, acute and chronic time window best explains injury likelihood. Methods Workload and injury data were collected from 53 athletes over 2 seasons in a professional Australian football club. Acute: chronic workload ratios were calculated daily for each athlete, and modelled against non-contact injury likelihood using a quadratic relationship. 6 workload variables, 8 acute time windows (2-9 days) and 7 chronic time windows (14-35 days) were considered (336 combinations). Each parameter combination was compared for injury likelihood fit (using R-2). Results The ratio of moderate speed running workload (18-24 km/h) in the previous 3 days (acute time window) compared with the previous 21 days (chronic time window) best explained the injury likelihood in matches (R-2= 0.79) and in the immediate 2 or 5 days following matches (R-2= 0.76-0.82). The 3: 21 acute: chronic workload ratio discriminated between high-risk and low-risk athletes (relative risk= 1.98-2.43). Using the previous 6 days to calculate the acute workload time window yielded similar results. The choice of acute time window significantly influenced model performance and appeared to reflect the competition and training schedule. Conclusions Daily workload ratios can inform injury risk in Australian football. Clinicians and conditioning coaches should consider the sport-specific schedule of competition and training when choosing acute and chronic time windows. For Australian football, the ratio of moderate speed running in a 3-day or 6-day acute time window and a 21-day chronic time window best explained injury risk.
354	Revisiting the empirical model Phases in the junior-to-senior transition of Swedish ice hockey players': External validation through focus groups and interviews	Pehrson, S; Stambulova, NB; Olsson, K	INTERNATIONAL JOURNAL OF SPORTS SCIENCE & COACHING	2017	10.1177/1747954117738897	S3	WoS	In this study, we continue to explore the junior-to-senior transition process in Swedish ice hockey by validating the empirical model Phases in the junior-to-senior transition of Swedish ice hockey players' created in our previous study.(1) More specifically, we collected opinions and critical reflections of 15 senior professional players and expert coaches about the empirical model as a whole and about the content of each junior-to-senior transition phase and then converted the original empirical model into its validated version. Professional ice hockey players and first league coaches formed three focus groups, while three senior coaches were interviewed individually. All were encouraged to critically reflect on the empirical model. The data from all sources were analysed following guidelines of the thematic (deductive) analysis complemented by quantitative measurements of the participants' agreement with the content of the empirical model. Results supported the sequence and designations of the junior-to-senior transition phases (i.e. preparation, orientation, adaptation and stabilization), as well as duration of the junior-to-senior transition (i.e. up to four seasons) outlined by the empirical model. We were also able to validate the psychological content (perceived demands, resources, barriers, coping strategies and outcomes) of each transitional phase. Accordingly, five themes were reformulated, 17 new themes were added and some themes were re-ordered during the conversion of the original empirical model into the validated model. To improve the readability of the validated model (from the bottom to the top), we also re-ordered sub-categories describing the psychological content of each junior-to-senior transition phase. We conclude by discussing the validated model in relation to theoretical frameworks and previous research and providing some methodology, future research and practice-oriented reflections addressed to researchers, coaches and sport psychology practitioners.

355	Longitudinal associations between athletes' controlled motivation, ill-being, and perceptions of controlling coach behaviors: A Bayesian latent growth curve approach.	Stenling, Andreas ; Ivarsson, Andreas ; Hassmén, Peter ; Lindwall, Magnus	Psychology of Sport & Exercise	2017	10.1016/j.psychsport.2017.03.002	S3	SPORTDiscus	Although many scholars have argued that leadership is a dynamic process jointly produced by leaders and followers, leadership in sports is most often researched as a unidirectional process from coaches to athletes. Within self-determination theory (SDT), individual characteristics are suggested to influence how people perceive external events such as coaches' behaviors. In the present study, we examined this jointly produced leadership process by investigating longitudinal associations between athletes' controlled motivation, ill-being, and perceptions of coaches' controlling behaviors at the between- and within-person levels. The participants were 247 young elite skiers enrolled at Swedish sport high schools who responded to self-report questionnaires at three time points over the course of an athletic season. At the between-person level, increases in perceptions of coaches' controlling behaviors over the season positively predicted controlled motivation at the end of the season, and controlled motivation at the beginning of the season positively predicted ill-being at the end of the season. At the within-person level, athletes' controlled motivation positively predicted perceptions of coaches' controlling behaviors. The results at the between-person level support the unidirectional perspective and the tenets of SDT. The results at the within-person level suggest that individual characteristics such as motivation can influence how athletes perceive external events, which has been proposed theoretically but seldom examined empirically. Three plausible explanations for this reversed association are presented in the discussion.
356	The effect of an additional substitution in association football. Evidence from the Italian Serie A	Varela-Quintana, C; Cuervo, JD; Prieto-Rodríguez, J	REVISTA DE PSICOLOGIA DEL DEPORTE	2016		S1	WoS	The substitution of players during a match has been one of the most significant changes in the Laws of the Game of the association football. FIFA regulated its application in the 1970 World Cup by establishing two replacements, which were expanded to three in 1995. The experimental introduction in 2016 of a fourth replacement during the extra time of several tournaments (the Olympic Games, the FIFA U-20 Women's World Cup and the FIFA Club World Cup) as a previous step to a possible application in next 2018 World Cup makes advisable to study how coaches reacted to an additional substitution in past situations. The present research examines coaches' behavior before and after the FIFA regulatory reform in 1995 that authorized the third replacement. For this, substitutions made in the Italian Serie A during the seasons 1994-95 and 1995-96 are analyzed. Our hypothesis is that teams made more physiological substitutions than tactical ones. which would reflect in a higher proportion of neutral substitutions (replacements of players who belong to the same positions). The results from the estimated discrete choice models found evidence of a change in the coaches' behavior giving preference to neutral replacements over tactical substitutions, probably to maintain the game intensity and prevent injuries.
357	A review of competitive sport motivation research	Clancy, RB; Herring, MP; MacIntyre, TE; Campbell, MJ	PSYCHOLOGY OF SPORT AND EXERCISE	2016	10.1016/j.psychsport.2016.09.003	S1	WoS	Objectives: The purpose of this review was to synthesise contemporary research on competitive sport motivation (1995-2016) with a view to identifying trends and gaps in sample characteristics, research designs and analytical strategies, and classifying associated topics. Design: Narrative review. Method: Four databases were searched using a focused search strategy, leading to 63 studies that met the inclusion criteria. These were then evaluated in five sections: Theoretical Frameworks, Sample Characteristics, Research Designs, Analytical Strategies and Topics. Results: A total of 63 studies comprising 12,440 participants were appraised. The average sample size and participant age were 197 and 22.6 years, respectively. Just over half of the studies described their sample as high-level. The majority of studies were quantitative and employed a cross-sectional design. A range of innovative statistical analyses were used, with structural equation modelling being increasingly adopted. In addition to studies focusing on motivation as a stand-alone topic, researchers in the area also investigated its relationship with motivational climate, burnout, doping, perfectionism, injury, and several other related variables. Conclusions: After reviewing the body of research on this specific topic area, promising directions for future research are presented. The focused approach particularly seeks to encourage researchers to employ different methodologies, and prioritise underused topics in future research on competitive athlete groups. Furthermore, the review points to several practical implications for competitive sport stakeholders. (C) 2016 Elsevier Ltd. All rights reserved.
358	Enhancing Collegiate Women's Soccer Psychosocial and Performance Outcomes by Promoting Intrinsic Sources of Sport Enjoyment	Barnicle, SP; Burton, D	JOURNAL OF SPORTS SCIENCE AND MEDICINE	2016		S3	WoS	This study examined the effectiveness of an applied mental skills training (MST) intervention utilizing mental skills to enhance intrinsic sources of enjoyment (ISOEs) as a means of promoting self-confidence, motivational style, and athletic performance, while also decreasing trait anxiety. The intervention project was designed to increase intrinsic SOE using a systematic and individualized mental training protocol, and then examine its relationships to mental skills and soccer performance. A Division 1 collegiate women's soccer team was randomly assigned to treatment (n = 8) and control (n = 11) groups, equally distributed by academic year, position, and pre-season coach-evaluated starters and non-starts. Results revealed that the MST intervention significantly increased intrinsic enjoyment targeted psychological and competitive outcomes, both in practice and competition within the treatment group as compared to the control group. This study's support for the impact mental skills training may have had on ISOEs, as well as other psychosocial outcomes and athletic performance can serve to highlight a mental skill often overlooked by consultants and coaches.

359	EFFECT OF PHYSICAL AND ACADEMIC STRESS ON ILLNESS AND INJURY IN DIVISION 1 COLLEGE FOOTBALL PLAYERS	Mann, JB; Bryant, KR; Johnstone, B; Ivey, PA; Sayers, SP	JOURNAL OF STRENGTH AND CONDITIONING RESEARCH	2016	10.1519/JSC.0000000000001055	S3	WoS	Mann, JB, Bryant, KR, Johnstone, B, Ivey, PA, and Sayers, SP. Effect of physical and academic stress on illness and injury in division 1 college football players. J Strength Cond Res 30(1): 20-25, 2016Stress-injury models of health suggest that athletes experience more physical injuries during times of high stress. The purpose of this study was to evaluate the effect of increased physical and academic stress on injury restrictions for athletes (n = 101) on a division I college football team. Weeks of the season were categorized into 3 levels: high physical stress (HPS) (i.e., preseason), high academic stress (HAS) (i.e., weeks with regularly scheduled examinations such as midterms, finals, and week before Thanksgiving break), and low academic stress (LAS) (i.e., regular season without regularly scheduled academic examinations). During each week, we recorded whether a player had an injury restriction, thereby creating a longitudinal binary outcome. The data were analyzed using a hierarchical logistic regression model to properly account for the dependency induced by the repeated observations over time within each subject. Significance for regression models was accepted at p 0.05. We found that the odds of an injury restriction during training camp (HPS) were the greatest compared with weeks of HAS (odds ratio [OR] = 2.05, p = 0.0003) and LAS (OR = 3.65, p < 0.001). However, the odds of an injury restriction during weeks of HAS were nearly twice as high as during weeks of LAS (OR = 1.78, p = 0.0088). Moreover, the difference in injury rates reported in all athletes during weeks of HPS and weeks of HAS disappeared when considering only athletes that regularly played in games (OR = 1.13, p = 0.75) suggesting that HAS may affect athletes that play to an even greater extent than HPS. Coaches should be aware of both types of stressors and consider carefully the types of training methods imposed during times of HAS when injuries are most likely.
360	The Coaching Behavior Scale for Sport (CBS-S): A psychometric evaluation of the Swedish version	Carlsson, A; Lundqvist, C	SCANDINAVIAN JOURNAL OF MEDICINE & SCIENCE IN SPORTS	2016	10.1111/sms.12359	S3	WoS	The present study validated a Swedish version of the 47-item Coaching Behavior Scale for Sport (CBS-S). Sample 1 consisted of 506 team sport athletes [262 men and 244 women; mean age: 22.20, standard deviation (SD)=3.90] distributed across 41 coaches at the two highest national levels of various sports. Athletes completed the CBS-S and established questionnaires of coaching behaviors (LSS), self-confidence (CSAI-2R), and coach-athlete relationship (CART-Q). An additional sample of 39 basketball players (21 men and 18 women; mean age=17.40, SD=2.39) completed the CBS-S twice, approximately 4 weeks apart. Confirmatory factor analysis showed an acceptable model fit for the seven-factor version of the CBS-S, although two items of the negative personal rapport subscale displayed insufficient factor loadings. Correlations between the subscales of the CBS-S and established instruments were in accordance with theoretical expectations, supporting the concurrent validity. Cronbach's alpha (>0.82) for all dimensions provided support for the reliability of the CBS-S, and test-retest correlations indicated moderate stability over time. Cultural differences in the assessment of coaching behaviors and the usability of the CBS-S by coaches for self-reflection and development are discussed.
361	Emotional intelligence in sport and exercise: A systematic review	Laborde, S; Dosseville, F; Allen, MS	SCANDINAVIAN JOURNAL OF MEDICINE & SCIENCE IN SPORTS	2016	10.1111/sms.12510	S3	WoS	This review targets emotional intelligence (EI) in sport and physical activity. We systematically review the available literature and offer a sound theoretical integration of differing EI perspectives (the tripartite model of EI) before considering applied practice in the form of EI training. Our review identified 36 studies assessing EI in an athletic or physical activity context. EI has most often been conceptualized as a trait. In the context of sport performance, we found that EI relates to emotions, physiological stress responses, successful psychological skill usage, and more successful athletic performance. In the context of physical activity, we found that trait EI relates to physical activity levels and positive attitudes toward physical activity. There was a shortage of research into the EI of coaches, officials, and spectators, non-adult samples, and longitudinal and experimental methods. The tripartite model proposes that EI operates on three levels - knowledge, ability, and trait - and predicts an interplay between the different levels of EI. We present this framework as a promising alternative to trait and ability EI conceptualizations that can guide applied research and professional practice. Further research into EI training, measurement validation and cultural diversity is recommended.
362	MOVEMENT PROFILES, MATCH EVENTS, AND PERFORMANCE IN AUSTRALIAN FOOTBALL	Johnston, RJ; Watsford, ML; Austin, DJ; Pine, MJ; Spurrs, RW	JOURNAL OF STRENGTH AND CONDITIONING RESEARCH	2016	10.1519/JSC.0000000000001333	S3	WoS	This study examined the relationship between movement demands, match events, and match performance in professional Australian football. Data were collected from 19 players using global positioning system units during 2 Australian Football League seasons. A range of movement demands and instantaneous power measures were collected. The players were divided into high-caliber (HC, >= 17/20) and low-caliber (LC, <= 8/20) groups based on the rating score by their coaches. A Mann-Whitney U-test, independent samples t-test, and effect sizes were used to determine whether any differences existed between the 2 groups. The HC group had a significantly higher match duration (7.2%), higher total distance (9.6%), and covered more distance and spent more time high-speed running per minute (12.7 and 11.9%). Although not significant, the effect sizes revealed that the HC group tended to have a higher mean metabolic power output (2.6%) and spent more time at the high power zone (7.9%). For the match event data, the HC group had significantly more involvements with the football. The results demonstrated the higher physical demands placed on the HC group. The findings suggest that analyzing instantaneous power measures can provide valuable information about the physical demands placed on team sport athletes to coaches and conditioning staff.

363	The Development, Implementation, and Evaluation of an Athlete Leadership Development Program With Female Varsity Athletes	Duguay, AM; Loughead, TM; Munroe-Chandler, KJ	SPORT PSYCHOLOGIST	2016	10.1123/tsp.2015-0050	S3	Scopus+WoS	The purpose of the current study was to develop, implement, and evaluate a season-long athlete leadership development program. Participants were 27 female varsity athletes who participated in four leadership workshops throughout the season, each 1 hr in duration. All of the participants completed inventories measuring leadership behaviors, cohesion, communication, athlete satisfaction, and peer motivational climate. Overall, the results showed significant differences in regards to leadership behaviors, athlete satisfaction, and peer motivational climate from pre- to postintervention. Further, follow-up focus groups were also conducted to assess the social validity of the leadership development program. These focus groups revealed important insight into program structure, influence of the program, leadership challenges, and suggestions for future improvements. These findings provide researchers, sport psychology consultants, and coaches with important information regarding the effectiveness of this athlete leadership development program in targeting human and social capital development.
364	Effects of basic psychological needs on Chinese youth athlete burnout under coach burnout: Using hierarchical linear modeling	Zhong, JL; Wang, QY; Bao, HS; Wang, Y; Guo, S	PSYCHOLOGY OF SPORT AND EXERCISE	2026	10.1016/j.psychsport.2026.103099	S1	Scopus+WoS	Under China's elite athlete development system, the result-oriented and high-intensity training often exposes young athletes to many risks, which can easily lead to athlete burnout (AB). While previous research has identified numerous individual-level antecedents of AB, limited attention has been paid to how coach burnout (CB), operating at the organizational level, may contribute to AB in individual level. Grounded in SelfDetermination Theory and employing Hierarchical Linear Modeling (HLM), this study examined the influence of basic psychological needs and CB on AB among 938 athletes (M-age = 17.47, SD = 1.11) and their 34 Chief coaches from provincial sports teams in China. At the individual level, autonomy was found to be a significant negative predictor of AB (gamma = -0.11, t = -2.34, p < .05), whereas competence and relatedness showed no significant predictive effect. At the organizational level, Cross-level analysis indicated that CB significantly and positively predicted AB (gamma = 0.53, t = 8.33, p < .001), and significantly moderated the relationship between relatedness and AB in a positive direction (gamma = 0.17, t = 2.57, p < .05). These findings underscore the critical role of autonomy in mitigating AB and highlight CB as both a direct and moderating factor in Chinese youth athlete psychological health. To effectively prevent burnout in youth athletes, sports organizations should address both coach well-being and athletes' basic psychological needs by implementing regular coach training programs and fostering stronger coach-athlete relationships.
365	Leveraging Student-Athlete Mental Health Through an AI-Augmented Mobile Platform: The ThriveNudge Study Protocol	Chakraborty, S; Mendro, N; Li, LX	BEHAVIORAL SCIENCES	2026	10.3390/bs16020268	S3	Scopus+WoS	Playing sports remains one of the most common avenues for youth engagement in physical activity. Yet mental health challenges, such as performance anxiety, depressive symptoms, reduced motivation, and burnout, place many young athletes at risk. As key mediators of sport participation, coaches' roles are often underscored in recognizing shifts in athlete motivation, behavior, or well-being. Gaining better insight into athlete mental health status may enable coaches to provide timely support and strengthen athlete and team well-being. In this study protocol, we employ a mixed-methods design, evaluating the effectiveness of an AI-augmented mobile application (i.e., ThriveNudge) in promoting the mental health of youth athletes. ThriveNudge helps coaches monitor athlete mental health, flag mood disruptions, and practice supportive communication via simulated chats. A target sample of four interscholastic teams (with athletes aged 14-18 years) and their head coaches will be recruited. Teams will be cluster-randomized to either the intervention condition (n = 2), receiving pre-season training to implement ThriveNudge, or to a waitlist control condition (n = 2). Primary outcomes, including athlete burnout, motivation, coach-athlete relationships, and sport enjoyment, will be measured using psychometric scales administered online. Semi-structured interviews will be conducted with coaches and athletes in the experimental group to collect qualitative data on user interface and user experience. We hypothesize that teams using ThriveNudge will report lower athlete anxiety and burnout, higher intrinsic motivation and enjoyment, and stronger coach-athlete relationships than athletes in control teams. We aim to provide a scalable and accessible digital platform that safeguards youth mental health.
366	Trajectories of sport exhaustion, cynicism and inadequacy among adolescent student-athletes: A three-year longitudinal study of social influences in the Finnish dual career context	Kuokkanen, J; Phipps, DJ; Saarinen, M; Korhonen, J; Romar, JE; Gustafsson, H	PSYCHOLOGY OF SPORT AND EXERCISE	2026	10.1016/j.psychsport.2025.103015	S3	WoS	Sport schools enhance adolescents' athletic development by offering extra training opportunities in addition to sport club training, but a rapid increase in the training load may elevate the risk of sport burnout. Thus, the aim of this study was twofold: (1) to explore the trajectories of three sport burnout dimensions (exhaustion, cynicism and inadequacy) among Finnish student-athletes (n = 268; 44 % female, 56 % male) across three academic years in lower secondary sport schools (ages 13-15 years), and to (2) examine how these trajectories were associated with student-athletes' perceived social support from parents, peers and coaches, as well as their gender, sport type and competition level. Student-athletes completed associated questionnaires at three time points at the end of three academic years-Grades 7, 8, and 9. Linear latent growth models revealed that feelings of inadequacy and cynicism increased during lower secondary school across the sample. At baseline, female student-athletes reported higher levels of inadequacy and cynicism, whereas males showed higher levels of exhaustion. Social support from peers and coaches reduced the initial levels and progression of burnout over time, respectively. Although paternal support initially minimized inadequacy, continued support subsequently tended to increase inadequacy. The findings highlight the importance of examining the three dimensions as interconnected aspects of sport burnout, and to support student-athletes in addressing emerging feelings of inadequacy and cynicism before these symptoms intensify. Educating coaches and strengthening peer support would be particularly beneficial while helping mothers to take a more active role in sports and fathers to provide support without becoming overly involved.

367	Caring coaching climates emerge as key factor in promoting high school athletes' well-being and motivation, complimented by a task-involving climate	Hogue, CM	INTERNATIONAL JOURNAL OF SPORT AND EXERCISE PSYCHOLOGY	2026	10.1080/1612197X.2024.2437481	S3	SPORTDiscus+WoS	When coaches utilise evidence-based practices, the benefits to athletes often extend far beyond the physical. Achievement goal theory has provided insight into which coaching practices facilitate and which hinder youth development. This investigation assessed whether the perceived motivational climate (caring, task-involving, and ego-involving climates; CCs, TICs, EICs) on high school teams are related to athlete well-being and motivation. Athletes (N = 181; M = 15.71 y.o.) completed a questionnaire mid-season. For both the girls' and boys' teams, CCs were negatively linked to shame, psychosocial stress, and depression while TICs were positively linked to state coping appraisals and motivation to continue, after controlling for the nested structure of the data. For athletes on girls' teams (n = 129), CCs were negatively associated with state stress appraisals and positively associated with feeling that their coach cares about what happens to them, state coping, enjoyment, and motivation to continue. For girls' teams, TICs were positively associated with enjoyment and negatively associated with state stress and life stress. For boys' teams, CCs were negatively associated with life stress, while EICs were negatively linked to feeling that their coach cares about what happens to them. These results suggest coaches should create CCs and TICs to elicit adaptive motivational responses and foster greater well-being in youth. More than a third (36.5%) of boys' teams athletes and 24.0% on girls' teams reported to some extent that their coach is one of the only adults who cares about them, underscoring the critical role coaches can play during adolescence.
368	Perceived parental autonomy support and moral behavior in youth athletes: applying the trans-contextual model	Choi, Y; Sim, Y; Hwang, K; Bae, J	FRONTIERS IN PSYCHOLOGY	2026	10.3389/fpsyg.2026.1780952	S3	WoS	Introduction This study empirically examined the role of perceived parental autonomy support in promoting moral behavior among student-athletes through the Trans-Contextual Model (TCM).Methods Using data from 355 Korean high school athletes across 16 sports, we investigated the effects of autonomy support on autonomous and controlled motivations in everyday and sport contexts, and their subsequent influence on moral attitudes, subjective norms, perceived behavioral control, and prosocial and antisocial behaviors.Results Structural equation modeling revealed that parental autonomy support positively influenced both autonomous and controlled motivations in daily life, which then transferred to sport contexts. Autonomous motivation significantly predicted moral beliefs and intentions, leading to increased prosocial and decreased antisocial behaviors. Interestingly, controlled motivations also positively impacted subjective norms, suggesting complex motivational processes underlying moral conduct.Conclusion These findings extend previous research by integrating Self-Determination Theory and the Theory of Planned Behavior, highlighting the importance of motivational quality and contextual transfer in shaping athletes' moral actions. Practical implications for coaching, parental education, and athlete development programs are discussed, along with directions for future longitudinal and cross-cultural research.
369	Changes in perceptions of coach- and peer-created motivational climates during the children-to-youth sport transition	Jorgensen, TM; Erickson, K; Abrahamsen, FE; Gjesdal, S	JOURNAL OF APPLIED SPORT PSYCHOLOGY	2026	10.1080/10413200.2026.2661050	S3	WoS	A mastery-oriented motivational climate focused on personal improvement, teamwork, and effort is linked to positive outcomes among young athletes, whereas a performance-oriented climate centered on social comparison and displaying superiority is associated with adverse outcomes. However, little is known about how these climates change during key developmental transitions in sport. This study examined changes in athletes' perceptions of coach- and peer-created motivational climates during the children-to-youth sport transition (CYT) in Norway, a period characterized by policy changes, increased competitiveness, and greater specialization. We followed athletes through this transition by collecting data on three separate occasions, each approximately six months apart. In total, 425 soccer and handball players aged 11-14 (Mage at time one = 12.16; 47.1% girls) participated in the study, and latent growth curve modeling was used to study changes over time. The results showed a small decline in coach-created mastery climate, along with large increases in coach-created performance climate and moderate increases in peer-created performance climate during the CYT. Notably, decreases in coach-created mastery climate corresponded with rises in coach-created performance climate. Gender differences emerged, with girls showing sharper increases in coach-created performance climate. These findings highlight the value of monitoring climate changes and suggest that targeted initiatives, such as coach training and the promotion of positive peer norms, may support the maintenance of mastery cues as athletes transition to youth sports.
370	Talent identification and development recommendations in youth sports: a real-time Delphi study	Bennett, KJM; Mccalman, W; Goddard, SG; Williams, AM; Fransen, J	JOURNAL OF APPLIED SPORT PSYCHOLOGY	2026	10.1080/10413200.2026.2636296	S3	WoS	Talent identification and development are critical components of athlete performance pathways, yet they remain heavily debated in the sports science and psychology literature. Although numerous best-practice recommendations have been proposed, there is a divide between their conceptual value and practicality. The current study attempted to address this gap, adopting a real-time Delphi methodology to capture the perspectives of expert academics and practitioners on literature-derived talent identification and development recommendations. Experts (n = 20) rated each of the 15 Delphi statements for agreement and feasibility using a five-point Likert scale and provided open-text justifications. Real-time feedback allowed the panel to view emerging group consensus and anonymous peer comments. Quantitative results indicated consensus for most recommendations; however, qualitative responses revealed practical barriers to implementation, including limited resources and time, insufficient coach and athlete education opportunities, a lack of meaningful data, and inter-sport competition for athletes. Accordingly, future research could benefit from a shift in focus toward evaluating the efficiency and effectiveness of talent identification and development initiatives. Methodologically, this may require longitudinal qualitative research and mixed-method experimental designs that better account for context-specific constraints.

371	The relationships among peer motivational climate, team preparation, and mental toughness in youth soccer players	Hwang, JH; Chung, KY; Cabral, M; Vazou, S; Song, YG	INTERNATIONAL JOURNAL OF SPORTS SCIENCE & COACHING	2026	10.1177/17479541261429879	S3	WoS	Teamwork is a crucial process for youth sport teams to accomplish a successful season. Despite that teamwork preparation is considered an influential bridge between environmental factors and individual outcomes, there is a paucity of research investigating team preparation as a mediator. Therefore, the purpose of the study was to examine how team preparation mediates the relationship between peer motivational climate and mental toughness of male youth athletes. A total of 526 male youth soccer players of elite middle and high school, and university soccer teams completed a survey at their sport facility. Multiple regression and mediation analysis were conducted with SPSS and AMOS to examine the associations among peer motivational climate, team preparation, and mental toughness. The results showed significant positive direct associations among peer task motivational climate, team preparation, and mental toughness except for the association between peer task climate and mental toughness, and negative direct association between peer ego motivational climate and team preparation. For mediation analysis, team preparation positively and negatively mediated the relationships between peer task and ego climate, and mental toughness, respectively. These results indicate that when peer groups form a task motivational climate, athletes are likely to prepare for their season collaboratively, and it can be positively related to individuals' mentality. This underscores the importance of team preparation, and it provides practitioners with prospective insight into how they can design their coaching strategies to benefit both teams and individuals.
372	The Influence of Parent-Initiated Motivational Climate on Adolescent Golfers' Flow Experiences: The Mediating Role of Basic Psychological Needs	Sim, Y; Kwon, S; Lee, D	PERCEPTUAL AND MOTOR SKILLS	2025	10.1177/00315125251336272	S1	SPORTDiscus+Scopus+WoS	The aim of this study was to investigate the roles of adolescent golf athletes' perceptions of (a) parent-initiated motivational climate and (b) their satisfaction of basic psychological needs in predicting their self-reported flow state. Participants were 429 adolescent golfers (Males = 203, Females = 226) who were registered with the Korean Sport and Olympic Committee and who completed surveys measuring these constructs. To verify the structural validity of the measurement tools, descriptive statistics, inference analysis, correlational analysis, structural equation modeling, and confirmatory factor analysis were employed. Also, in structural equation modeling, we examined the mediating effect of satisfying psychological needs in the relationship between parent-initiated motivational climate and flow state. Results showed that these participants' perceptions of their task-involved, parent-initiated motivational climate was positively related to their perceptions of their basic psychological needs. Basic psychological needs were significantly and positively related to flow. However, perceived parent-initiated motivational climate was not significantly related to flow. Therefore, athletes' perception of their basic psychological needs completely mediated the relationship between parent-initiated motivational climate and flow. The task-involved, parent-initiated motivational climate perceived by adolescent golf athletes predicted their increased self-report of flow through satisfaction of basic psychological needs. The analysis verified that adolescent golfers' perceptions of a task-involved, parent-initiated motivational climate may have contributed to achieving perceived flow and, presumably, peak performance. Consequently, these findings highlight the crucial role of parental involvement in creating an optimal environment for young athletes' performance.
373	Comparative effects of French Contrast Method vs. Complex Training on explosive power and its endurance in youth badminton athletes	Huang, RY; Gao, YH; Yang, K; Mo, Y; Lu, YR; Gao, Z	PLOS ONE	2025	10.1371/journal.pone.0338383	S2	Scopus+WoS	Badminton players normally prioritize technical and tactical training over physical conditioning before competition, presenting a challenge in enhancing physical fitness within a constrained timeframe. While evidence have indicated complex contrast training can enhance strength and power, it is still unclear whether the French Contrast Method Training (FCMT) can bring greater explosive power gains to young badminton players. This study evaluated the effects of French Contrast Method Training versus Complex Training on lower limb explosive strength and its endurance in elite adolescent male badminton players. In a single-blind randomized controlled trial, 20 athletes were allocated to either an FCMT group (n = 10) or a CT group (n = 10) and completed an 8-week intervention. Performance was assessed pre- and post-intervention using standardized tests, including countermovement jump (CMJ), squat jump (SJ), drop jump (DJ), 10-m sprint, 5-0-5 agility test, eccentric utilization ratio (EUR), one-repetition maximum (1RM) squat, and lower extremity explosive endurance (LEEE). Subjective fatigue was monitored using the rating of perceived exertion (RPE) scale. Results demonstrated that while both training modalities improved explosive strength, the French Contrast Method Training led to significantly greater improvements in CMJ, DJ, Reaction Strength Index (RSI), EUR, agility, and LEEE ($p < 0.05$). The French Contrast Method Training also induced a larger effect size across most performance indicators. In contrast, the Complex Training improved strength and speed-related measures but showed limited effects on stretch-contraction cycle (SSC) utilization and endurance. Perceived fatigue was consistently higher in the CT group than in the FCMT group. These findings suggest that adaptation occurred more rapidly in the CT group (after roughly 3 weeks), whereas the FCMT group showed a more prolonged adaptation period (similar to 1 month). The findings suggest that the French Contrast Method Training is more effective than the Complex Training in developing reactive strength, agility, SSC efficiency, and explosive endurance in youth badminton athletes within a short-term training period. Given its neuromechanical advantages across the force-velocity spectrum, the French Contrast Method Training is recommended for use in the pre-competition phase of training, while Complex Training may be more suitable for foundational strength development. These results provide practical insights for coaches and support the strategic integration of

374	Advancements in research on psychological and emotional aspects of student-athletes	Mao, ZJ	FRONTIERS IN PSYCHOLOGY	2025	10.3389/fpsyg.2025.1645177	S3	WoS	Purpose: This systematic review synthesizes evidence on the psychological and emotional experiences of collegiate student-athletes, identifying key stressors, coping strategies, and intervention effectiveness. Methods: Literature was searched in PubMed, PsycINFO, Scopus, and Web of Science for studies published between 2010 and 2024. A total of 41 empirical studies met inclusion criteria (27 cross-sectional, 7 longitudinal/observational, 4 quasi-experimental/experimental, and 3 mixed-methods). The studies were conducted primarily in the USA (n = 19), Europe (n = 11), and East Asia (n = 8), with sample sizes ranging from 30 to >1,000 participants. Results: Across studies, the most frequently reported stressors were training load (68% of studies), academic pressure (61%), and coach-athlete or team dynamics (42%). Emotional outcomes included anxiety (reported in 70% of studies), depression (52%), and burnout/fatigue (39%). Quantitative data indicated that depressive symptoms affected up to 35% of female student-athletes and COVID-related disruptions increased distress levels in over 40% of participants. Coping strategies with strongest empirical support included Psychological Skills Training (PST) (9 studies), Cognitive-Behavioral Therapy (CBT) (3 studies), and self-care practices such as sleep hygiene and mindfulness (11 studies). Conclusion: Student-athletes' well-being is shaped by the dual demands of sport and academics, moderated by gender and cultural context. Evidence supports PST and CBT as effective interventions, complemented by self-care practices. To translate these findings into practice, universities and sports organizations should integrate structured mental skills programs into athletic training, provide flexible academic support during peak competition periods, and invest in accessible counseling services. Policy efforts should focus on reducing stigma, training coaches and staff to recognize early signs of distress, and expanding digital or mobile-based mental health solutions to increase accessibility.
375	Effects of Cohesion With Teammates on Division-I Student-Athletes' Mental Health: An Application of the Human Need to Belong and Transactional Stress Frameworks	Cranmer, GA; Cox, EG; Rey, RT; Browning, B; Holbert, LG	COMMUNICATION & SPORT	2025	10.1177/21674795241267197	S3	WoS	Collegiate student-athletes are experiencing an ongoing mental health epidemic, especially concerning anxiety and depression. This study builds upon the meta-theory of the human need to belong and the transactional theory of stress and coping by framing stress as a mediator for the benefits of cohesion between teammates on mental health. Data from 121 Division-I student-athletes demonstrated a fully mediated model whereby task cohesion indirectly benefited anxiety and depression through lower stress levels. Social cohesion, however, was not significantly associated with either stress or anxiety and depression. These findings underscore teammate relationships are appraised as social stimuli and that the lack of shared task approaches and understandings among teammates can be distressing and detract from general well-being. Coaches, athletic departments, and other academic stakeholders may use this information to guide their efforts to lower student-athlete stress through their social relationships with others. This aim can become an emphasis on pre-season training, team meetings, and athlete check-ins.
376	The predictive role of coach-athlete relationship quality in training engagement and skill development among adolescent basketball players	Luo, YY; Li, SR; Cao, YY; Luo, ZJ	FRONTIERS IN PSYCHOLOGY	2025	10.3389/fpsyg.2025.1648082	S3	SPORTDiscus+WoS	Objective This study aimed to examine the predictive effect of coach-athlete relationship (CAR) quality on training engagement (TE) and shooting skill improvement (SI) among adolescent basketball players, as well as the potential mediating role of TE in this relationship. Methods A total of 128 basketball players aged 16-18 years (including 83 males) were recruited. The Coach-Athlete Relationship Questionnaire (CART-Q) was used to assess CAR, the Task and Ego Orientation in Sport Questionnaire (TEOSQ) measured TE, and a 100-shot stationary shooting test was used to evaluate SI at both pre- and post-season. Statistical analyses included Pearson correlation, hierarchical regression, and structural equation modeling (SEM). The significance level was set at alpha = 0.05. Results CAR was significantly positively correlated with both TE (r = 0.52, p < 0.001) and SI (r = 0.38, p < 0.001). After controlling for gender and competition experience, CAR remained a significant predictor of TE (beta = 0.48, p < 0.001) and SI (beta = 0.31, p = 0.002). TE partially mediated the relationship between CAR and SI (beta = 0.14, 95% CI [0.06, 0.24]), accounting for 46% of the total effect. The SEM showed a good model fit (chi 2/df = 1.86, CFI = 0.95, RMSEA = 0.072). Conclusion A high-quality coach-athlete relationship not only directly enhances training engagement in adolescent basketball players but also indirectly facilitates shooting skill improvement over the course of a season by increasing training engagement.
377	Shorter follow-through is associated with greater accuracy: a kinematic analysis of the tennis forehand in youth athletes	Bottura, RM; Ferrando, V; Placidilli, F; Ruggeri, P; Faelli, E; Bisio, A	INTERNATIONAL JOURNAL OF PERFORMANCE ANALYSIS IN SPORT	2025	10.1080/24748668.2025.2577551	S3	WoS	In tennis, athletes' technique, influenced by biomechanical characteristics, plays a key role in success. Forehand groundstroke is the most used technique during a match. This study analysed the relationship between forehand stroke duration and shot accuracy. Sixteen skilled young tennis players (National Class: 3.11 +/- 0.43) were evaluated before and after 6-week off-season training. The test involved 15 forehands with maximal acceleration from a standing position. Each stroke was video recorded for movement duration analysis, while accuracy was based on whether the ball landed in or out of the designed area. Total duration was divided into two phases: backswing to impact (initial) and impact to follow-through (final). A significant reduction in total duration suggested improved efficiency. However, no significant changes were observed in accuracy. A significant negative correlation emerged between accuracy and the duration of the final phase. This study showed that, in youth players, improved forehand accuracy is significantly associated with shorter follow-through times. Findings suggest efficiency and control of the follow-through phase play a key role in shot precision, even without explicit changes in accuracy. From a practical perspective, coaches may benefit from emphasising post-impact control and efficient deceleration, as these elements appear linked to greater accuracy in young athletes.

378	Coping profiles of adolescent football players and association with interpersonal coping: Do emotional competence and psychological need satisfaction matter?	Doron, J; Hayotte, M; d'Arripe-Longueville, F; Leprince, C	SCANDINAVIAN JOURNAL OF MEDICINE & SCIENCE IN SPORTS	2024	10.1111/sms.14550	S2	Scopus+WoS	Using a person-centered approach, the present study aimed to investigate the coping profiles of adolescent football players involved in elite football training centers. The purposes were to (1) identify coping profiles based on the reported use of multiple coping strategies in response to competitive stress, (2) explore whether emotional competencies and psychological need satisfaction would predict coping profile membership, and (3) examine the extent to which coping profiles were differently associated with individual and team perceived stress, interpersonal coping, and subjective team performance, as well as demographic characteristics. A sample of 416 young French football players (males = 282; females = 134; Mage = 16.2; SDage = 1.2) from 12 elite football training centers participated in this study. Latent profile analysis results yielded three coping profiles allowing players to be grouped according to their preferences for a combined use of certain strategies (i.e., low copers, high disengaged copers, and high task copers). Results provided further insight into each coping profile membership by indicating the role played by intrapersonal emotional competence and psychological need satisfaction. Finally, differences between coping profiles have been shown in terms of individual perceived stress intensity, interpersonal coping approach, and gender. These findings provide a deeper understanding of adaptive coping profiles within a population of adolescent football players involved in elite training centers. Implications for developing and tailoring psychoeducational interventions for adolescent football players exhibiting a maladaptive coping profile (i.e., disengagement-oriented coping profile) are considered.
379	Exploring the Dynamics of Athletes' Enjoyment and Self-Determined Motivation, and of the Motivational Climate in Youth Football: A Longitudinal Perspective	Rodrigues, F; Monteiro, D; Matos, R; Jacinto, M; Antunes, R; Amaro, N	PERCEPTUAL AND MOTOR SKILLS	2024	10.1177/00315125231222152	S3	Scopus+WoS	We aimed to explore a short period longitudinal interplay between athletes' enjoyment and their self-determined motivation and motivational climate in youth football. We recruited 109 youth football athletes (79 males, 30 females) through a convenient sampling method. We included individuals within the 12-17-year-old age range, with a mean age of 14.31 (SD = 1.46) years. To examine these proposed associations, we performed hierarchical multiple regression analyses and found that enjoyment at pre-season assessment (T1) and self-determined motivation and a task-involving sport climate at mid-season (T2) were significant predictors of mid-season enjoyment (at T2). However, self-determined motivation and task-involving climate at T1 did not significantly contribute to the model. These findings emphasize the importance of initial enjoyment and an evolved self-determined motivation, and task-involving climate in understanding later enjoyment in sport. Coaches, practitioners, and policymakers should prioritize strategies that enhance intrinsic motivation, provide opportunities for autonomy, and cultivate a supportive and growth-oriented environment.
380	Motivational climate dimensions predict youth soccer players' psychosocial well-being over time	Kipp, LE; Bolter, ND	PSYCHOLOGY OF SPORT AND EXERCISE	2024	10.1016/j.psychsport.2023.102518	S3	Scopus+WoS	Objectives: This study tested a longitudinal model of relationships, based on self-determination theory, to determine whether motivational climate dimensions predicted young athletes' psychological need satisfaction and, in turn, personal and social responsibility. Design: We used a longitudinal design. Method: Youth soccer players (N = 161; M = 10.8 years-old, SD = 1.0 year) completed a survey at two time points, spaced 4 months apart, on average. Results: Several significant direct effects emerged. First, greater perceptions that coaches punished for mistakes predicted decreases in relatedness with coaches and teammates. Second, greater perceptions of relatedness with coaches and teammates predicted increases in personal and social responsibility. Indirect effects also emerged: (a) punishment for mistakes predicted decreases in personal responsibility and social responsibility, and (b) cooperative learning predicted increases in social responsibility, through effects on coach and teammate relatedness. Conclusions: Results suggest that coaches who provide opportunities for collaborative learning and minimize mistake-contingent punishment will foster athletes' sense of connection and enhance their psychosocial wellbeing.

381	A Longitudinal Mixed Methods Case Study Investigation of the Academic, Athletic, Psychosocial and Psychological Impacts of Being of a Sport School Student Athlete	Thompson, F; Rongen, F; Cowburn, I; Till, K	SPORTS MEDICINE	2024	10.1007/s40279-024-02021-4	S3	WoS	Background Sport schools are popular environments for simultaneously delivering education and sport to young people. Previous research suggests sport school involvement to have impact (i.e. the positive/negative, intended/unintended and long/short-term outcomes, results and effects) on student athlete's holistic (i.e. academic, athletic, psychosocial and psychological) development. However, previous research is limited by (1) cross-sectional methods, (2) limited multidimensional assessments, (3) lack of consideration for athlete characteristics (e.g. sex) and (4) failure to evaluate how sport school features affect student-athlete impacts.Objectives The study, using a mixed methods case study approach, aims to (1) longitudinally evaluate the impact of sport school involvement on the holistic development of student athletes, (2) evaluate the impact on holistic development by student-athlete characteristics and (3) explore the features and processes of the sport-school programme that drive/facilitate holistic impacts.Methods A longitudinal mixed methods design was employed across one full academic school year (33 weeks). Six data-collection methods (i.e. online questionnaire, physical fitness testing battery, academic assessment grades, log diaries, field notes/observation and timeline diagram/illustration) were used to assess the academic, athletic, psychosocial and psychological impacts for 72 student athletes from one sport school in the United Kingdom (UK).Results Student athletes developed positive long-term holistic overall impacts (i.e. academically, athletically and personally), including maintaining stable and relatively high levels of sport confidence, academic motivation, general recovery, life skills, resilience and friends, family and free time scores. Despite positive impacts, juggling academic and sport workload posed challenges for student athletes, having the potential to lead to negative holistic impacts (e.g. fatigue, stress and injury). Positive and negative impacts were linked to many potential features and processes of the sport school (e.g. academic and athletic support services versus insufficient training load build-up, communication, coordination, flexibility and planning). Furthermore, when considering student-athlete characteristics, females had lower sport confidence, higher general stress and body image concerns and less general recovery than males and student athletes who played sport outside the school had lower general recovery.Conclusions This mixed method, longitudinal study demonstrated sport school
382	Supporting my daughter, the best way I can: Parental support in youth golf	McIlmoyle, A; Sharp, LA; Woods, D; Paradis, KF	JOURNAL OF APPLIED SPORT PSYCHOLOGY	2024	10.1080/10413200.2024.2344841	S3	WoS	Burgess et al. (2016) emphasized the importance of parental support in youth sports as they provide financial, informational, and emotional support to the youth-athlete. Parents also play a significant role in shaping youth sport experiences (Sheridan et al., 2014, p. 198). Research around parental support has been conducted in sports such as tennis, gymnastics, and football. The aim of the present study was to examine the parents' perspective of parental support in female youth golf, exploring how they support their female youth golfers, and if the support changes through their child's development. Twenty-two semi structured interviews were conducted with parents (14 fathers, 10 mothers) of high-performance female golfers in the specializing or investment stages of C & ocirc;t & eacute;'s (1999) DMSP. Participants were recruited from six countries (England, Ireland, Scotland, New Zealand, Australia, Canada). Using thematic analysis (Braun & Clarke, 2012) six higher order themes were identified, namely, parental support: emotional, practical, technical, financial, organizational, and reflective support. The results align with the grounded theory of parental support (Burke et al., 2023a) providing an insight into the parents' perspective of providing support aligning with informational, emotional, and instrumental support of the theory. Furthermore, the current research presented novel findings regarding reflective support that parents provide. Findings highlighted that parental support changed depending on temporal differences (place in the golf season) and their daughter's development. The present research reinforces the need to provide support programs for parents based on their needs, rather than programs designed from a governing body or coaches perspective. Parents perspective of parental support and associated temporal changes were explored in female youth golf. Six themes of support were identified: emotional, practical, technical, financial, organizational, and reflective support. Changes in parental support were dependent on their daughter's development (level of maturity or level of play) and the time of year differences. Sport psychologists can build upon the current findings when assisting the development of youth athletes, by involving the parent in managing emotions for their child-athlete but also providing some help for how parents may manage their own emotions to avoid having a negative impact on their youth athlete.Findings can be applied by practitioners, governing bodies, and sporting organizations to develop and implement suitable

383	Incivility and psychological safety in youth sport: the reciprocal effects and its impact on well-being and social outcomes	Kinoshita, K; Sato, S	SPORT MANAGEMENT REVIEW	2023	10.1080/14413523.2022.2138110	S3	WoS	Youth can often be the subject of rude and discourteous behaviors in their sport setting, given their susceptibility to the social environments. Incivility refers to insensitive behavior that exhibits a lack of respect for others, namely, disrespectful and rude behaviors. Incivility is a significant issue in youth sport since it negatively influences teams and individuals. The present study aims to investigate 1) how incivility and psychological safety are associated with each other over time and 2) the influence of the initial value of psychological safety and the change in psychological safety on youth athletes' well-being and social outcomes. Three-wave time-lagged data collection was employed, and the present study included 283 youth athletes who completed the survey three times. The hypotheses were tested in SEM with cross-lagged panel and growth latent curve modeling. The results showed that coach and teammate incivility were significantly associated with the change in psychological safety. In contrast, the initial value of psychological safety was a significant antecedent of the subsequent coach and teammate incivility, well-being, and social outcomes. Lastly, the change in psychological safety was significantly associated with youth athletes' well-being and social outcomes. The findings suggest that incivility and psychological safety were reciprocally associated, and psychological safety rather than incivility was a significant predictor of youths' well-being and social outcomes. The present study found a mechanism underlying the relationship between incivility, psychological safety, and essential outcomes (i.e., well-being and social outcomes) in youth sport.
384	Temporal dynamics of the quality of the coach-athlete relationship over one season among adolescent handball players: a latent class analysis approach	Roux, V; Martinent, G; Trouilloud, D	INTERNATIONAL JOURNAL OF SPORT AND EXERCISE PSYCHOLOGY	2023	10.1080/1612197X.2022.2109185	S3	SPORTDiscus+WoS	Objective. The purpose of this study was to examine the temporal evolution of the quality of the coach-athlete relationship (CAR), in terms of athletes' perceptions of commitment, closeness, and complementarity, over a 1-season period. More precisely, the intra-individual temporal dynamic of the quality of the CAR was explored in three complementary ways: (1) mean change in the whole sample, (2) inter-individual differences between athletes, and (3) intra-individual stability and/or change of CAR profiles. Method. Eighty handball players (Mage = 16.15; 78% boys) involved in intensive training centres completed a measure of the quality of the CAR across three time points (beginning, middle, and ending of the season). Data were analysed using latent class growth and latent profile transition analyses. Results. Latent class growth analyses indicated that athletes' perceptions of closeness and complementarity linearly decrease - on average - over the season, whereas those of commitment decreases in a quadratic way (U-shape). In addition, results revealed three distinctive athletes' trajectories for closeness and complementarity, and four for commitment, suggesting inter-individual differences in the intra-individual evolution of the perceived quality of the CAR. Finally, latent profile transition analyses highlighted the existence of four distinct CAR profiles, and revealed that most athletes displayed the same profile over the season, but some of them changed their profile over time. Conclusion. As a whole, these results highlighted the dynamic aspect of athletes' perceived quality of the CAR, suggesting that this relationship is likely to evolve in different ways over a season in intensive training context.
385	Perceptions, facilitators, and barriers regarding use of the injury prevention exercise programme Knee Control among players and coaches in youth floorball: a cross-sectional survey study	Åkerlund, I; Sonesson, S; Lindblom, H; Waldén, M; Hägglund, M	BMC SPORTS SCIENCE MEDICINE AND REHABILITATION	2023	10.1186/s13102-023-00660-0	S3	WoS	BackgroundYouth participation in team ball sports is associated with a risk of both acute and gradual onset injuries but today there are several efficacious injury prevention exercise programmes (IPEPs). However, there is limited research about how to implement those programmes and the perceived barriers and facilitators among end-users.ObjectiveTo investigate perceptions of the IPEP Knee Control and facilitators and barriers to programme use among coaches and youth floorball players, and explore factors associated with planned maintenance of Knee Control.MethodsThis cross-sectional study is a sub-analysis of data from the intervention group of a cluster randomised controlled trial. Perceptions about Knee Control and facilitators and barriers to programme use were evaluated with surveys pre-intervention and post-season. 246 youth floorball players aged 12-17 years, and 35 coaches that reported no use of IPEPs during the preceding year were included. Descriptive statistics and univariate and multivariate ordinal logistic regression models were undertaken with the dependent variables: coaches' planned maintenance and players' opinions of maintenance of Knee Control. Independent variables were perceptions, facilitators and barriers regarding use of Knee Control and other potential influencing factors.Results88% of the players believed that Knee Control can reduce injury risk. Common facilitators to Knee Control use among coaches were support, education and high player motivation, and common barriers were that injury prevention training was time-consuming, lack of space to execute the exercises and lack of player motivation. Players who planned to maintain use of Knee Control had higher outcome expectancies and belief in one's ability to use Knee Control (action self-efficacy). Coaches who planned to maintain Knee Control had higher action self-efficacy and to a lesser extent considered that Knee Control takes too much time.ConclusionsSupport, education, and high player motivation are key facilitators, while lack of time and space for injury prevention training and boring exercises are key barriers for coaches and players to use Knee Control. High action self-efficacy among coaches and players seems to be a prerequisite for maintained use of IPEPs.

386	Changes in Quality of Life, Sleep, and Physical Activity During COVID-19: A Longitudinal Study of Adolescent Athletes	Wingerson, MJ; Baugh, CM; Provance, AJ; Armento, A; Walker, GA; Howell, DR	JOURNAL OF ATHLETIC TRAINING	2023	10.4085/1062-6050-0529.22	S3	WoS	Context: The abrupt cessation of school and sport participation during the COVID-19 pandemic may have negative implications for adolescent mental health.Objectives: To (1) compare mental, physical, and social health and behaviors during pandemic-related stay-at-home mandates with the same measures collected 1 to 2 years earlier and (2) evaluate the relationships between physical activity and sleep during the pandemic and changes in anxiety, fatigue, and peer relationships between assessment times.Design: Cohort study.Setting: Pediatric sports medicine center.Patients or other participants: A total of 39 high school athletes (25 adolescent girls, 14 adolescent boys; age = 16.2 +/- 0.9 years).Main outcome measure(s): Patient-Reported Outcome Measurement System anxiety, fatigue, and peer relationships short forms and the Pittsburgh Sleep Quality Index were completed twice (initial assessment in May 2018 or 2019, follow-up assessment in May or June 2020). Frequency and duration of physical activity and frequency of interaction with other individuals (family, peers, sport coaches, etc) were self-reported at follow-up assessment for the 2 weeks before school or sport closure and the 2 weeks before questionnaire completion.Results: Higher levels of anxiety (5.5 +/- 4.0 versus 3.6 +/- 3.4 points; P = .003) and fatigue (5.4 +/- 3.7 versus 2.3 +/- 2.5 points; P < .001) and worse sleep quality (6.6 +/- 2.9 versus 4.3 +/- 2.3 points; P < .001) were observed during the pandemic compared with previous assessments. Reductions in physical activity were noted between assessments (exercise duration: 86.4 +/- 41.0 versus 53.8 +/- 30.0 minutes; P < .001). Sleep quality but not physical activity during the pandemic predicted changes in fatigue (P = .03, beta = 0.44 [95% CI = 0.06, 0.83]) and peer relationships (P = .01, beta = -0.65 [95% CI = -1.16, -0.15]) from initial to follow-up assessment.Conclusions: Mental and physical health declined during stay-at-home mandates compared with assessments 1 to 2 years earlier. Physical activity behaviors and sources of social interaction underwent changes after school and sport cessation. Sleep quality may have provided some protection against declining adolescent mental health during the pandemic, although this relationship requires further investigation.
387	Effect of coaches' interpersonal style on young athletes' individual resilience and team adherence intention: a season-long investigation	Llanos-Muñoz, R; Pulido, JJ; Nobari, H; Raya-González, J; López-Gajardo, MA	BMC PSYCHOLOGY	2023	10.1186/s40359-023-01445-3	S3	Scopus+WoS	Background In the sports context, coaches must be able to improve their players physically, psychologically, and socially. Hence, a fundamental part of this process is the athlete's individual resilience (IR).Methods Three hundred and fifteen youth team-sport players (boys: n = 283; M-age = 16.02, SD = 0.56; and girls: n = 32; M-age = 15.92, SD = 0.62) completed the measures of coach's interpersonal style, individual resilience, perceived performance, and team adherence intention (intention to remain on the same team the following year) twice (Time 1: mid-season; Time 2: end-season). Structural equation modeling was used to test the relationships between variables.Results The results showed that coach support was positively related to IR (p < 0.001) and, in turn, IR to individual (p < 0.01) and team performance (p < 0.05) at Time 1, and to individual performance (p < 0.001) and team adherence intention at Time 2 (p < 0.01). In addition, team performance at Time 2 was positively related to team adherence intention (p < 0.001). Finally, a mediating effect of IR was observed between interpersonal coaching style, individual and team performance, and team adherence intention.Conclusions These results show the importance of a supportive interpersonal coaching style to foster athletes' levels of resilience, which could have positive consequences in performance (individual and team) and team adherence intention.
388	Putting the Puzzle Back Together-A Narrative Case Study of an Athlete Who Survived Child Sexual Abuse in Sport	Gillard, A; St-Pierre, E; Radziszewski, S; Parent, S	FRONTIERS IN PSYCHOLOGY	2022	10.3389/fpsyg.2022.856957	S3	WoS	Denunciations of child sexual abuse (CSA) in the sport context have been increasing in the last decades. Studies estimate that between 14 and 29% of athletes have been victim of at least one form of sexual violence in sport before the age of 18. However, studies suggest that many do not disclose their experience of CSA during childhood. This finding is alarming since studies have shown that the healing process usually starts with disclosure. Moreover, little is known about the healing process of CSA experienced in the sport context. The aim of the study is to present a single case study of a CSA in sport to better understand the global experience over time from the perspective of the athlete. A narrative inquiry approach was adopted. Three non-structured interviews were conducted with the participant. Three pathways in the survivor journey have been identified through inductive thematic analysis: (a) pathway to understanding, (b) pathway to disclosure, and (c) pathway to healing. These pathways represent distinct processes but are intertwined as they are dynamic and iterative. Indeed, the survivor explained how she had been, and is still, going back and forth between them. Results are consistent with those found in the literature on CSA in the general population. It suggests that theoretical models of CSA in the general population could be applied to CSA in sport. Practical implications include a need for education and clearer boundaries in the coach-athlete relationship. Sport stakeholders also need to be better equipped to recognize the signs of sexual violence in sport. Our results indicate that qualitative research could be a potential avenue to help victims heal from CSA. It gives them the chance to talk about and make sense of their abuse in a safe space. Finally, our results demonstrate the importance of reviewing the current justice system for victims. It should be based on a trauma-informed approach that places the victim at the center of the judicial process.

389	High compliance with the injury prevention exercise programme Knee Control is associated with a greater injury preventive effect in male, but not in female, youth floorball players	Åkerlund, I; Waldén, M; Sonesson, S; Lindblom, H; Hägglund, M	KNEE SURGERY SPORTS TRAUMATOLOGY ARTHROSCOPY	2022	10.1007/s00167-021-06644-2	S3	WoS	Purpose Evaluate team and player compliance with the Knee Control injury prevention exercise programme, study the association between player compliance and injury rates, and compare coach demographics, baseline prevention expectancies, and programme utilisation between teams with high and low compliance. Methods Prospective one-season cohort study based on a cluster randomised controlled trial on 301 (107 female) floorball players aged 12-17 years. Floorball exposure and injuries were self-reported weekly by players using the Oslo Sports Trauma Research Center questionnaire. Team and player compliance to Knee Control was reported monthly by coaches. Additionally, coaches answered pre- and post-season surveys. Teams were divided into a high (>= 80%) or low (< 80%) compliance group based on their use of Knee Control during the season. Players were divided into three compliance groups based on their average weekly number of Knee Control sessions; high (>= 2 sessions), intermediate (>= 1 to < 2 sessions), and low dose (< 1 session). Results Mean team compliance for the high and low compliance groups were 95% (range 82-100) and 50% (range 13-66), respectively. Mean +/- SD weekly Knee Control dose in the three player compliance groups were 2.4 +/- 0.3, 1.4 +/- 0.3, and 0.7 +/- 0.3 sessions, respectively. There were no differences in total injury incidence between the player compliance groups, but players in the high-dose group had a 35% lower prevalence of injuries overall [adjusted prevalence rate ratio (PRR) 0.65, 95% CI 0.48-0.89] and 60% lower prevalence of substantial injuries (adjusted PRR 0.40, 95% CI 0.26-0.61) compared with the low-dose group. Male players in the high-dose group had consistently lower injury incidence and prevalence, while no between compliance group differences were seen in female players. There were no differences in sex, years of coaching experience, or baseline prevention expectancies in general between coaches for teams in the high vs. low compliance groups, but teams in the high compliance group had a better utilisation fidelity. Conclusion There was a clear dose-response relationship between more frequent Knee Control use and lower injury rates in male floorball players, but not in female players. Teams with higher compliance also showed a better utilisation fidelity with the programme.
390	The implementation and evaluation of an athlete leadership development program with male youth ice hockey players	Boisvert, MM; Loughead, TM; Munroe-Chandler, KJ	FRONTIERS IN PSYCHOLOGY	2022	10.3389/fpsyg.2022.648039	S3	WoS	The purpose of the current study was to implement and evaluate an athlete leadership development program in youth boys ice hockey. The sample consisted of 14 male U17 hockey players (M = 16.46, SD = 0.78) from one team playing in a competitive hockey league. The players participated in six leadership intervention workshops over the course of the season, and completed inventories measuring athlete leadership behaviours, cohesion, and collective efficacy pre-and post-intervention. In addition, a focus group was conducted to assess the impact of the athlete leadership development program at the end of the season. Bayesian t tests showed that the leadership program generally helped to maintain levels of athlete leadership behaviours, cohesion, and collective efficacy pre- and post-intervention. The results of the focus group following the intervention revealed the players believed the leadership development program helped buffer against the negative effects of their on-ice performances.
391	Mental health protective factors among flourishing Canadian women university student-athletes	Pankow, K; McHugh, TLF; Mosewich, AD; Holt, NL	PSYCHOLOGY OF SPORT AND EXERCISE	2021	10.1016/j.psychsport.2020.101847	S3	WoS	Mental health is an important resource for student-athletes. Flourishing (Keyes, 2002) may be a useful concept for examining aspects of mental health. Therefore, the purpose of this study was to investigate mental health protective factors among flourishing Canadian university student-athletes over the course of their sport season. Initially, 36 Canadian student-athletes completed the Mental Health Continuum - Short Form (Keyes, 2009) and Sport Mental Health Continuum - Short Form (Foster & Chow, 2019). Based on the results from the questionnaires, six women athletes who were identified as flourishing at global and sport levels then participated in the study. Data were generated through individual preand post-season semi-structured interviews, and in-season weekly written diaries. Analysis followed guidelines outlined by Smith et al. (2009) for interpretative phenomenological analysis. Results are presented over three stages of the seasons (pre-, in-, and post-season). In the pre-season, participants invested in their flourishing by making positive connections and planning their schedule. In-season, participants discussed facing challenges and tried to maintain flourishing by managing their commitments, communicating with coaches, and looking for positives. In the post-season, the participants attempted to re-invest in their flourishing by reflecting on their season and taking a break from sport. It appeared that the athletes were able to identify potential challenges to their flourishing in sport and use global resources to buffer the impact. These results reveal strategies that may help promote and protect mental health among studentathletes.
392	A united front: Coach and teammate motivational climate and team cohesion among female adolescent athletes	Weiss, MR; Moehnke, HJ; Kipp, LE	INTERNATIONAL JOURNAL OF SPORTS SCIENCE & COACHING	2021	10.1177/17479541211006905	S3	WoS	Based on theory and research on athlete motivation and sport cohesion, this study examined the concurrent association of perceived coach and peer motivational climates with team cohesion among female adolescent athletes. Participants (N = 235) included 14- to 18-year-old competitive volleyball players who completed self-report measures of motivational climates and team cohesion in the latter part of the season. Canonical correlation analysis revealed that all coach and peer task-involving dimensions were positively related, and peer intra-team conflict (ego-involving dimension) was negatively related, to team cohesion. Task-involving dimensions of coach cooperative learning and peer effort and relatedness support contributed most strongly to the relationship as did task cohesion. Multiple regression analyses showed that elements of both coach and peer climates were related to task and social cohesion. Findings extend past research by simultaneously examining coaches and peers as social sources of team cohesion among female adolescent athletes. Specific strategies are offered for how coaches can enhance team cohesiveness by directly shaping a task-involving climate, minimizing an ego-involving climate, and fostering a teammate task-involving climate that promotes positive peer interactions and relationships.

393	Perceived coach-created environment directly predicts high school athletes' physical activity during sport	Chu, TL; Zhang, XX; Lee, J; Zhang, T	INTERNATIONAL JOURNAL OF SPORTS SCIENCE & COACHING	2021	10.1177/1747954120959733	S3	WoS	Sport participation is an important means for adolescents to achieve moderate-to-vigorous physical activity (MVPA), yet most high school students including athletes do not achieve the 60-minute daily MVPA guideline. As psychosocial factors influence athlete engagement and physical activity during sport, the perceived environment created by coaches could play a role in this influence. Guided by self-determination and achievement goal theories, this four-month prospective study examined the direct and indirect effects of perceived coach-created environment on high school athletes' MVPA and sedentary behavior (SB) during sport. During the third to fourth week of a sport season, 225 high school athletes (M-age = 15.24 years) completed a survey assessing perceptions of coach-created empowering and disempowering climates as well as psychological need satisfaction and frustration. Four months later, their MVPA and SB percentage times (%) during sport were measured using accelerometers. Path analyses partially supported our hypothesis, indicating significant direct effects of a perceived empowering climate on need satisfaction (beta = .41) and need frustration (beta = -.29), and direct effects of a perceived disempowering climate on need frustration (beta = .38) and MVPA% (beta = -.28). No significant indirect effects on MVPA% or SB% were found. Findings support and provide new insights into the important role of disempowering beyond empowering climates in predicting high school athletes' PA. Specifically, when coaches display ego-involving and controlling behaviors, high school athletes may disengage during sport and achieve less overall MVPA. Further examination of these relationships using a longitudinal design across more diverse samples is warranted.
394	The role of growth, maturation and sporting environment on the development of performance and technical and tactical skills in youth basketball players: The INEX study	Guimaraes, E; Baxter-Jones, ADG; Williams, AM; Tavares, F; Janeira, MA; Maia, J	JOURNAL OF SPORTS SCIENCES	2021	10.1080/02640414.2020.1853334	S3	WoS	In this paper, we outline a systematic testing programme developed to help identify excellence in youth basketball players. We examine the links between biological maturation and training experience with anthropometry, body composition, physical performance, technical and tactical skills from five age-cohorts, and characterize, in detail, facets of their environment. In total, 238 young basketball players aged 11-15 years, clustered into five age-cohorts (11, 12, 13, 14, 15 years) were recruited. We assessed measures across three domains: (1) biological [anthropometry, body composition, biological maturation and physical performance]; (2) skill/game proficiency [technical skills and tactical skills]; and (3) contextual [family support, coach knowledge and competence and club context]. The data were analysed using one-way ANOVAs and multivariate analysis of covariance adjusting for biological maturation and training experience. We report significant differences favouring older basketball players on most biological and skill/game proficiency variables. However, differences between age-cohorts in physical performance and technical skills were mitigated after controlling for the effects of both covariates. In conclusion, our findings highlight the important role of both biological maturation and training experience on youth basketball players' performance and development. We discuss the implications of these findings for research as well as for athletes, parents, coaches and clubs.
395	Evaluation of the NFHS Online Captains Leadership Course: Student Athletes' Views of Effectiveness	Walker, LF; Gould, DR	FRONTIERS IN PSYCHOLOGY	2021	10.3389/fpsyg.2021.648559	S3	WoS	Sport is viewed as an arena for positive life skill development, including leadership development. In 2015, the NFHS launched an online Captain's Leadership Training Course. The main purpose of this study was to examine the effectiveness of the course in improving leadership knowledge and ability. An electronic survey was sent to a sample of athletes (n = 202, 129 female), ages 13-19 (M = 17.01, SD = 0.10) in eight United States states who had completed the NFHS course within the last 3-18 months. Most athletes (92.6%) completed the course based upon their coach's recommendation. The course was viewed to be moderately to very useful (M = 2.49, SD = 1.00) in helping them in preparing to be a team captain. Participants believed the course to be very to extremely effective in building their knowledge on motivation (M = 1.96, SD = 0.89), communication (M = 1.90, SD = 0.80), decision making (M = 2.03, SD = 0.91), peer modeling (M = 1.91, SD = 0.86), team cohesion (M = 1.96, SD = 0.88) and problem solving strategies (M = 2.00, SD = 0.85). Canonical correlation analyses showed that athletes who felt they were more reflective tended to rate the effectiveness of the course lower than their peers. Additionally, analyses did not show any clear demographic characteristics that distinguished between perceptions of the effectiveness of the course, showing the value found in the course was high with all types of scholastic athletes. Athletes felt the course could be improved most in the area of learning how to manage conflict with their peers and coaches. Future research in scholastic leadership should seek to understand the impact of the course prospectively across a high school sport season.

396	Transformational parenting and coaching on mental toughness and physical performance in adolescent soccer players: The moderating effect of athlete age	Murray, RM; Dugdale, JH; Habeeb, CM; Arthur, CA	EUROPEAN JOURNAL OF SPORT SCIENCE	2021	10.1080/17461391.2020.1765027	S3	WoS	Both parent and coach leadership behaviours are instrumental to adolescent athlete development. Researchers, however, are yet to examine parent and coach leadership influences simultaneously, and at different stages of adolescents' psychological and physical development. Therefore, the purpose of this study was to understand if the effects of transformational parenting, and transformational coaching on mental toughness and performance varied at different ages during adolescence. Early adolescent (ages 10-14) and late adolescent (ages 15-18) soccer players (n = 334) completed questionnaires assessing their perceptions of their mother's, father's, and coach's transformational leadership, as well as a questionnaire assessing mental toughness. Participants also completed a comprehensive battery of physical fitness tests relevant to soccer. Results indicated that transformational fathering was more strongly associated with levels of mental toughness for early adolescent athletes than it was for later adolescent athletes. Results also indicated that transformational coaching was more strongly associated with physical performance for later adolescent athletes than it was for early adolescents. Overall, these results can inform development models and provide support for future longitudinal studies to assess the impact of parent and coach transformational leadership across different stages of athlete development.
397	Perceived parental behaviours and motivational processes among adolescent athletes in intensive training centres: A profile approach	Lienhart, N; Nicaise, V; Martinent, G; Guillet-Descas, E	PSYCHOLOGY OF SPORT AND EXERCISE	2020	10.1016/j.psychsport.2020.101708	S3	WoS	Parental behaviours influence athletes' psychological functioning in different ways. To date, research has typically explored parental behaviours one by one, and few papers have simultaneously considered sets of parental behaviours. The objectives of this study were to: (a) identify parental behaviours using a person-centred approach and differentiating mother's and father's behaviours; (b) explore the changes of parental behaviour profiles across the season; and (c) examine the prospective impact of parental behaviour profiles at the beginning of the season on athlete scores of motivation, satisfaction, and frustration of basic psychological needs at the end of the season. A longitudinal two-wave measurement design (beginning and end of a season) was used in the study. A sample of 226 French athletes completed self-reported questionnaires: (a) at the beginning and end of the season to measure the behaviours of their parents; and (b) at the end of the season to assess their motivation, satisfaction, and frustration of basic psychological needs. Latent profile transition analysis revealed three parental behaviour profiles: (a) moderate parental involvement; (b) moderate to high parental involvement; and (c) moderate mother's involvement and low father's involvement. The profiles were based on associated patterns of four mother's and father's behaviours: (a) directive behaviours; (b) active involvement; (c) praise and understanding; and (d) pressure. The three parental behaviour profiles remained stable across the season (i.e., interand intra-individual stability). Athletes who reported the moderate parental involvement profile at the beginning of the season had lower scores of controlled motivation and frustration of autonomy as well as higher scores of satisfaction of competence and relatedness at the end of the season than their counterparts from the two other profiles. This study contributes to enriching the complex picture of parental involvement in the sport to help sport organisations identify parents at risk of impeding their child's development.
398	Longitudinal associations between perceived programme quality, basic needs support and basic needs satisfaction within youth sport: A person-centred approach	Bean, C; Solstad, BE; Ivarsson, A; Forneris, T	INTERNATIONAL JOURNAL OF SPORT AND EXERCISE PSYCHOLOGY	2020	10.1080/1612197X.2018.1462234	S3	WoS	Acknowledging the importance of longitudinal data to test process-based psychological theories of motivation is critical. The purpose of this study was to use a person-centred approach to identify unique subgroups (i.e. profiles) of youth athletes based on their level of self-reported programme quality (PQ) and basic needs support mid-way through their sport season and investigate potential differences between the subgroups on their self-reported basic needs satisfaction at the end of the sport season. The current study involved 541 Canadian youth athletes (males n = 289; females n = 250; gender-fluid n = 2) within 52 sport programmes over the course of 18 months. Youth athletes ranged in age from 8 through 19 (M = 13.76, SD = 2.61). A latent profile analysis (LPA) in Mplus 8.0 was used to carry out the analyses. The LPA revealed three distinct profiles based on youth athletes' levels of self-reported PQ and basic needs support. Specifically, athletes who perceived their sport experience to be of higher quality and supported their basic psychological needs midway through the sport season also reported higher levels of basic needs satisfaction at season end. Results from this study contribute to the field of sport psychology through understanding how basic needs theory contributes to the dimensions of programme quality and by informing recommendations for future coach education on how to satisfy youth athletes' basic psychological needs for autonomy, competence, and relatedness through programme delivery.
399	Snapchat and child sexual abuse in sport: Protecting child athletes in the social media age	Sanderson, J; Weathers, MR	SPORT MANAGEMENT REVIEW	2020	10.1016/j.smr.2019.04.006	S3	WoS	Child sexual abuse is a pervasive problem that sport organizations must confront to protect the integrity of sport. While previous researchers have examined this topic, few have explored the role that technology plays. The authors address this gap by examining how coach perpetrators utilize Snapchat to facilitate sexual abuse, using Cense and Brackenridge's (2001) Temporal Model of Sexual Abuse with Children and Young Persons in Sport as a framework. Analysis of 99 media reports from 2013 to 2018 found that Snapchat provides motivation for coach perpetrators to sexually abuse that allowed them, in varied ways, to overcome internal inhibitions, external barriers, and break down victim resistance. The results indicate that youth sport organizations must develop clear policies that govern coach-athlete communication and relationships in the digital age. To further protect young athletes, youth sport organizations must develop programming to educate parents and athletes on these boundaries, to increase understanding about how sexual abuse and sexual maltreatment occur in virtual spaces. (C) 2019 Sport Management Association of Australia and New Zealand. Published by Elsevier Ltd. All rights reserved.

400	Positive youth development related athlete experiences and coach behaviors following a targeted coach education course	MacDonald, DJ; Camiré, M; Erickson, K; Santos, F	INTERNATIONAL JOURNAL OF SPORTS SCIENCE & COACHING	2020	10.1177/1747954120942017	S3	WoS	The purpose of this study was to examine if a positive youth development (PYD) coach education course would (i) result in more positive developmental experiences perceived by athletes of participant coaches and (ii) increase the frequency of PYD-oriented behaviors exhibited by participant coaches. A total of 14 coaches and 270 athletes participated in the study. Eight coaches (M-age = 38.0 years) overseeing 154 athletes (M-age = 13.8 years) were in the control group, while six coaches (M-age = 36.7 years) overseeing 116 athletes (M-age = 13.9 years) were in the experimental group. The 10-week intervention combined a group-based PYD coach education course with individualized coach meetings. Seven practices per coach were observed and recorded at baseline (n = 2), intervention (n = 3), and follow-up (n = 2). Athletes completed measures of coach-athlete relationships and sport experiences at the three time points. Results of the 2 x 3 (group x time) mixed ANOVAs demonstrated that coaches in the intervention group increased the frequency of their PYD-oriented behaviors during the intervention but returned to levels slightly above baseline at follow-up. Coaches in the control group did not change their behaviors over the same period. Athlete reports of their developmental experiences were high at all time points and did not change over time. Findings suggest PYD coach education has the potential to influence coach behavior, but further research is required to determine how to create sustained changes for coaches and increases in athletes' perceived positive developmental experiences.
401	The impact of humanistic coach training on youth athletes' development through sport	Falcao, WR; Bloom, GA; Sabiston, CM	INTERNATIONAL JOURNAL OF SPORTS SCIENCE & COACHING	2020	10.1177/1747954120933975	S3	WoS	The purpose of this study was to evaluate the impact of humanistic coach training on athletes' developmental outcomes. A sample of 148 high school student-athletes between 12 and 17 years old from low socio-economic communities completed questionnaires during their season. The student-athletes were divided into an experimental group composed of student-athletes of 11 head coaches who participated in a humanistic coach training session, and a comparison group composed of student-athletes of 8 untrained coaches. Four questionnaires were used to assess competence, confidence, connection to the coach, and character (prosocial and antisocial behaviours) of the participants. The effects of the humanistic coach training program were assessed using repeated measures analysis of variance models. Results showed that connection to the coach worsened for athletes of untrained coaches. Also, participants from both groups reported an increase in antisocial behaviours at the end of the season, but the athletes of trained coaches reported engaging less frequently in antisocial behaviours compared to athletes of untrained coaches. These findings suggest that teaching humanistic coaching may help practitioners foster positive developmental outcomes in youth sport participants and build positive coach-athlete relationships, while also raising awareness to the use of sport as a tool to promote personal growth and development.
402	Physical Performance Measures Correlate with Head Impact Exposure in Youth Football	Kelley, ME; Jones, DA; Espeland, MA; Rosenberg, ML; Miles, CM; Whitlow, CT; Maldjian, JA; Stitzel, JD; Urban, JE	MEDICINE & SCIENCE IN SPORTS & EXERCISE	2020	10.1249/MSS.0000000000002144	S3	WoS	Purpose Head impact exposure (HIE) (i.e., magnitude and frequency of impacts) can vary considerably among individuals within a single football team. To better understand individual-specific factors that may explain variation in head impact biomechanics, this study aimed to evaluate the relationship between physical performance measures and HIE metrics in youth football players. Methods Head impact data were collected from youth football players using the Head Impact Telemetry System. Head impact exposure was quantified in terms of impact frequency, linear and rotational head acceleration, and risk-weighted cumulative exposure metrics (RWELinear, RWERotational, and RWECP). Study participants completed four physical performance tests: vertical jump, shuttle run, three-cone, and 40-yard sprint. The relationships between performance measures, and HIE metrics were evaluated using linear regression analyses. Results A total of 51 youth football athletes (ages, 9-13 yr) completed performance testing and received combined 13,770 head impacts measured with the Head Impact Telemetry System for a full season. All performance measures were significantly correlated with total number of impacts in a season, RWELinear-Season, and all RWE-Game metrics. The strongest relationships were between 40-yard sprint speed and all RWE-Game metrics (all P <= 0.0001 and partial R-2 > 0.3). The only significant relationships among HIE metrics in practice were between shuttle run speed and total practice impacts and RWELinear-Practices, 40 yard sprint speed and total number of practice impacts, and three-cone speed and 95th percentile number of impacts/practice. Conclusions Generally, higher vertical jump height and faster times in speed and agility drills were associated with higher HIE, especially in games. Physical performance explained less variation in HIE in practices, where drills and other factors, such as coaching style, may have a larger influence on HIE.

403	The dynamic nature of connection and its relation to character in youth sport	Herbison, JD; Vierimaa, M; Côté, J; Martin, LJ	INTERNATIONAL JOURNAL OF SPORT AND EXERCISE PSYCHOLOGY	2019	10.1080/1612197X.2017.1423507	S3	WoS	Positive youth development (PYD) is an asset-oriented approach to adolescent development that has been adapted to sport. Interestingly, in sport, the PYD outcomes (i.e. competence, confidence, connection, and character) have largely been introduced as outcomes, with little attention to their dynamic nature or individual influences over time. In considering evidence from educational settings that indicates peer relationships fluctuate over time, the current project sought to explore the dynamicity of these relationships (i.e. connection) over the course of an athletic season. In addition, the potential for connection towards the beginning of a sport season to influence athlete character towards the end of the season was assessed. Athletes from five elite youth volleyball teams (N = 43 athletes; M-age = 15.86; SD = 1.17) completed peer nomination questionnaires (i.e. connection) at the beginning and end of an athletic season. At the end of the season, a practice session was videotaped and athlete behaviours were coded for frequency of prosocial and antisocial behaviours (i.e. character). Results indicate significant variability in connection over the course of a season, and those who were rated as popular by their team members at the beginning of the season were more likely to demonstrate prosocial behaviours at the end of the season. Discussions pertaining to the novel methodologies implemented, as well as the theoretical and practical implications of the results are advanced herein.
404	Longitudinal trajectories of emotions among young athletes involving in intense training centres: Do emotional intelligence and emotional regulation matter?	Cece, V; Guillet-Descas, E; Nicaise, V; Lienhart, N; Martinent, G	PSYCHOLOGY OF SPORT AND EXERCISE	2019	10.1016/j.psychsport.2019.01.011	S3	Scopus+WoS	Objectives: This study aimed to explore whether several subgroups of athletes representing distinct emotional trajectories emerged from the person-centred analyses for a wide variety of positive and negative sport emotions (anger, anxiety, sadness, confidence, happiness, harmony, love and vitality). The present study also focused on the determinants of the emotional dynamics exploring whether athletes reporting distinct scores of emotional intelligence (EI) and emotional regulation (ER) at time 1 (T1) belonged to distinct trajectories. Design: A longitudinal three-wave measurement design (beginning, middle, and end of a competitive season) was used in the present study. Method: A sample of 325 adolescent athletes evolving in intensive training centres completed three self-reported questionnaires. Results: Results of latent class growth analyses (LCGA) demonstrated four trajectories for love and confidence, three trajectories for anger, harmony, happiness, sadness, and vitality, and two trajectories for anxiety. Furthermore, the likelihood of belonging to emotional trajectories was significantly influenced by EI and ER scores for love, confidence, anger, harmony, happiness, sadness and vitality. Conclusions: LCGA results highlighted the young athlete heterogeneity in longitudinal sport emotions and the predictive effects of ER and EI on their emotional trajectories. These results could be used to help coaches and sport psychologists to identify athletes at risk of developing dysfunctional emotional trajectories across the competitive season. Moreover, this study suggested potential benefits of EI and ER-centred interventions to help young elite athletes to experience adaptive emotional trajectories. Finally, these results highlighted that the heterogeneity in sport emotions must be accounted for in future research.
405	Helping student-athletes learn to self-regulate to alleviate burnout: a multiple case study showcasing their challenging but altering experiences	Dubuc-Charbonneau, N; Durand-Bush, N	QUALITATIVE RESEARCH IN SPORT EXERCISE AND HEALTH	2018	10.1080/2159676X.2018.1433226	S3	WoS	A multiple case study approach was used to investigate how four university student-athletes learned to self-regulate by taking part in an individual season-long intervention designed to reduce stress and burnout symptoms. A male hockey player, female fencer, male swimmer and female basketball player met the initial selection criteria of scoring 3.0 or higher on two of the three subscales of the Athlete Burnout Questionnaire. Pre- and post-intervention interviews as well as seven to nine bi-weekly intervention sessions were conducted with each participant. All data were transcribed verbatim and subjected to a content analysis, subsequent to which narratives were created to depict each student-athlete's unique experiences throughout the intervention. Through a tailored educational approach, these student-athletes learned to develop and implement various self-regulation strategies in order to manage the multiple demands and challenges (e.g. injuries, autonomy-depleting coaching behaviours, amotivation toward school, excessive drinking, distracting social media) they faced throughout their season. The most commonly used strategies included goal-setting, time and task management, self-monitoring via daily 'check-ins' and self-reflection through journaling. Interestingly, prior to this study, two of the student-athletes had previously dropped out of their sport due to burnout but subsequently returned, and one was contemplating quitting at the time of the study. However, all four of them reported post-intervention that with reduced stress and burnout symptoms, and more quality performances and well-being, they intended to continue playing their sport the following year.

406	UNDERSTANDING THE RELATIONSHIP BETWEEN COACH AND ATHLETE PERCEPTIONS OF TRAINING INTENSITY IN YOUTH SPORT	Scantlebury, S; Till, K; Sawczuk, T; Weakley, J; Jones, B	JOURNAL OF STRENGTH AND CONDITIONING RESEARCH	2018	10.1519/JSC.0000000000002204	S3	SPORTDiscus+WoS	To alleviate issues arising from the over-/under-prescription of training load, coaches must ensure that desired athlete responses to training are being achieved. The present study aimed to assess the level of agreement between the coach-intended (presession) and observed (postsession) rating of perceived exertion (RPE), with athlete RPE during different training intensities (easy, moderate, hard). Coach-intended RPE was taken before all field-based training sessions over an 8-week in-season period. After training, all coaches and athletes, who were participants in hockey, netball, rugby, and soccer were asked to provide an RPE measure for the completed session. Sessions were then classified based on the coach's intended RPE, with a total of 28, 125, and 66 easy, moderate, and hard training sessions collected, respectively. A univariate analysis of variance was used to calculate within-participant correlations between coach-intended/-observed RPE and athlete RPE. Moderate correlations were found between coach-intended and athlete RPE for sessions intended to be moderate and hard whilst a small correlation was found for sessions intended to be easy. The level of agreement between coach and athlete RPE improved following training with coaches altering their RPE to align with those of the athlete. Despite this, moderate and small differences between coach-observed and athlete RPE persisted for sessions intended to be easy and moderate, respectively. Coaches should, therefore, incorporate strategies to monitor training load to increase the accuracy of training periodization and reduce potential over-/under-prescription of training.
407	An empirical examination comparing the Mindfulness-Acceptance-Commitment approach and Psychological Skills Training for the mental health and sport performance of female student athletes	Gross, M; Moore, ZE; Gardner, FL; Wolanin, AT; Pess, R; Marks, DR	INTERNATIONAL JOURNAL OF SPORT AND EXERCISE PSYCHOLOGY	2018	10.1080/1612197X.2016.1250802	S3	WoS	The present study was a randomised controlled trial investigating the effectiveness of the Mindfulness-Acceptance-Commitment (MAC) approach compared to traditional Psychological Skills Training (PST) for the mental health and sport performance of female collegiate athletes. Two hypotheses were proposed: (a) participants in the MAC group would demonstrate reduced behavioural issues, emotional distress, and psychological symptoms, and increased athletic performance when compared to those in the PST group; (b) MAC participants would exhibit reduced emotion dysregulation and increased psychological flexibility and dispositional mindfulness, compared to PST participants. Participants included 18 National Collegiate Athletic Association Division III female student athletes who were randomly assigned into either the MAC or PST group based upon pre-intervention levels of distress; and were assessed pre-intervention, post-intervention, and at 1-month follow-up. A mixed-model ANOVA analysis revealed that the MAC effectively reduced Substance Use, Hostility, and Emotion Dysregulation over time when compared to the PST group. Several within-group differences also emerged, as MAC participants demonstrated reduced Generalised Anxiety, Eating Concerns, and Psychological Distress, as well as increased psychological flexibility from post-intervention to one-month follow-up. As per coach ratings, MAC participants also evidenced improved sport performance from pre-intervention to post-intervention. Results suggest that the MAC is an effective intervention for the mental health and sport performance needs of female collegiate athletes.
408	Youth Sport Coaches' Well-Being Across the Season: The Psychological Costs and Benefits of Giving Empowering and Disempowering Sports Coaching to Athletes.	Solstad, Bård Erlend ; Ivarsson, Andreas ; Haug, Ellen Merethe ; Ommundsen, Yngvar	International Sport Coaching Journal	2018	10.1123/iscj.2017-0026	S3	SPORTDiscus	The purpose of this study was to investigate the associations between giving empowering and disempowering sports coaching to young athletes and coaches' well-being across the season. The sample comprised 169 Norwegian youth football (i.e., European soccer) coaches with a mean age of 41.99 (SD = 6.32). Moreover, we were interested in examining heterogeneous groups of coaches showing variability in their self-reporting of empowering and disempowering behaviors towards their athletes. Thus, a person-centered approach was used. The latent profile analysis revealed three distinct profiles and the association between these profiles and coaches' well-being was in line with the outlined hypotheses. Specifically, coaches who gave higher levels of empowering and lower levels of disempowering sports coaching to their athletes at the beginning of the season also reported higher levels of well-being at the end of the season. The results indicate that there exists an intrinsic value as to why coaches should give empowering sports coaching, as opposed to disempowering sports coaching, to their athletes; namely, these actions may be advantageous in terms of improving their own well-being. In practical terms, future coach education may take advantage of these findings by providing coaches another reason for coaching in an empowering manner. ABSTRACT FROM AUTHOR
409	Exploring relationships between the coach-initiated motivational climate and psychological coping skills over the high school American football season	Gano-Overway, LA; Steele, E; Boyce, BA; Whaley, D	INTERNATIONAL JOURNAL OF SPORTS SCIENCE & COACHING	2017	10.1177/1747954117738873	S3	WoS	This study investigated the relationship between the perceived coach-initiated motivational climate and psychological coping skills over a competitive high school football season as well as changes in perceptions of the climate over the season. Near the beginning (Time 1) and end of the season (Time 2), 101 players from five competitive high school American football programs completed the Perceived Motivational Climate in Sport Questionnaire-2 and the Athletic Coping Skill Inventory-28. A hierarchical linear regression revealed that Time 2 task-involving climate predicted Time 2 psychological coping skills when controlling for Time 1 psychological coping skills. Repeated measure multivariate analysis of variance results demonstrated that players perceived a decrease in the task-involving climate and an increase in the ego-involving climate over the course of the competitive season. These results add to the research on the positive role of a task-involving climate in the sport domain. Additionally, this research provides insight into how perceptions of the coach-initiated motivational climate can shift over the course of the competitive season.

410	Understanding psychological changes in adolescent wrestlers participating in an intensive training camp: A mixed-methods investigation	Driska, AP; Gould, DR; Pierce, S; Cowburn, IHJ	INTERNATIONAL JOURNAL OF SPORT PSYCHOLOGY	2017	10.7352/IJSP.2017.48.303	S3	Scopus+WoS	Research has indicated that adversity created in training environments can develop mental toughness in adolescent athletes (Bell, Hardy, & Beattie, 2013; Connaughton, Hanton, & Jones, 2010; Gucciardi, Gordon, & Dimmock, 2008). However, few studies (Bell et al., 2013) have explored this development prospectively. Employing a pragmatic, longitudinal, mixed methods design, this study assessed 70 adolescent wrestlers participating in an established intensive wrestling camp that systematically employed adversity. The Trait Sport Confidence Inventory, the State Hope Scale, and the Athletic Coping Skills Inventory 28 were administered at the outset, conclusion, and nine-months-following the camp. A repeated measures MANOVA with post-hoc measures showed increases in seven variables, maintained at the nine-month follow-up, with the strongest partial effect sizes in hope (eta = .242), sport confidence (eta = .151), and coping with adversity (eta = .142). Interviews with eight participants, conducted throughout the camp, one-week post-camp, and nine-months post-camp, yielded five high-order themes: enhanced confidence, work ethic, development of empowering attributes (e.g., responsibility, internal control), enhanced thought processes (e.g., self-awareness), and enhanced interpersonal skills. Results suggest that this highly-structured adverse training environment has the ability to develop a range of skills and dispositions related to mental toughness, and that some of these factors may transfer to other life contexts.
411	Just for the fun of it: coaches' perceptions of an exemplary community youth sport program	Vierimaa, M; Turnnidge, J; Bruner, M; Côté, J	PHYSICAL EDUCATION AND SPORT PEDAGOGY	2017	10.1080/17408989.2017.1341473	S3	WoS	Background: A growing body of research has studied sport as a vehicle for positive youth development (PYD). While much of this research has investigated the developmental outcomes associated with sport participation, less is known about the mechanisms through which PYD occurs in a sport context. Further, much of the research on PYD in sport has studied competitive sport settings, while comparatively less is known about recreational sport. Aims: This qualitative descriptive study explored coaches' perceptions of a successful recreational community youth basketball league. Specifically, this study aimed to describe coaches' perceptions of the structure of the league, the developmental outcomes associated with youth's participation in this sport program, as well as the mechanisms through which these outcomes are realized. Participants: A total of 12 volunteer basketball coaches (6 males and 6 females) were purposefully selected to participate in this study. The coaches were between 16 and 59 years old, had an average of 10.1 years of coaching experience, and 6 of the coaches previously played in the league in their youth. Data collection: Individual semi-structured interviews were conducted with each basketball coach. Data analysis: The interviews were transcribed verbatim and subjected to a thematic analysis that was driven by both the data and extant PYD conceptual frameworks. Findings: Identified themes were integrated into an existing PYD conceptual framework. These themes related to immediate (e.g. enjoyment), short-term (e.g. competence, confidence, connection, and character), and long-term developmental outcomes (e.g. contribution), as well as social and contextual processes (e.g. activities, social relationships, and settings) which underpin these outcomes. Conclusion: This study extended the current body of research on PYD in sport through emphasizing the importance of ensuring that youth have enjoyable, positive immediate experiences in sport, and that accumulated positive experiences result, over time, in lasting effects on athletes' development.
412	Young Athletes' Perceptions of Coach Behaviors and Their Implications on Their Well- and Ill-Being Over Time	González, L; García-Merita, M; Castillo, I; Balaguer, I	JOURNAL OF STRENGTH AND CONDITIONING RESEARCH	2016	10.1519/JSC.0000000000001170	S3	WoS	Gonzalez, L, Garcia-Merita, M, Castillo, I, and Balaguer, I. Young athletes' perceptions of coach behaviors and their implications on their well- and ill-being over time. J Strength Cond Res 30(4): 1147-1154, 2016-Grounded on basic psychological needs theory the purpose of this study was twofold: (a) to test the mediational role of basic psychological needs (satisfaction and thwarting), and (b) to test the model invariance over 2 consecutive seasons. Three hundred sixty young male athletes completed a questionnaire package tapping the variables of interest at 4 time points during 2 consecutive seasons. Results of the path analyses revealed that in both seasons, changes in perceived coach autonomy supportive style positively predicted changes in needs satisfaction which, in turn, positively predicted changes in self-esteem; changes in perceived coach autonomy supportive and controlling style negatively and positively, respectively, predicted changes in needs thwarting which, in turn, positively predicted changes in burnout and negatively in self-esteem. Only in the first season, changes in needs satisfaction emerged as a negative predictor of changes in burnout. The mediational role of basic psychological needs and the invariance of the aforementioned relationships over the 2 seasons were supported. Results emphasize the importance of having coaches promoting autonomy supportive atmospheres and avoiding controlling styles to facilitate athletes' well-being and to prevent their ill-being.
413	A Three-Wave Longitudinal Test of Self-Determination Theory's Mediation Model of Engagement and Disaffection in Youth Sport	Curran, T; Hill, AP; Ntoumanis, N; Hall, HK; Jowett, GE	JOURNAL OF SPORT & EXERCISE PSYCHOLOGY	2016	10.1123/jsep.2015-0016	S3	WoS	Research adopting self-determination theory (SDT) supports a mediation model whereby coach motivational styles (autonomy support and interpersonal control) predict athletes' engagement and disaffection in youth sport via the satisfaction and frustration of psychological needs (autonomy, competence, and relatedness). Our study extends this research by examining SDT's mediation model longitudinally with three waves of data. Two hundred fifty-two youth sports participants (M-age = 12.98; SD = 1.84; range = 11-17; female n = 67) completed measures of study variables at the start, middle, and end of a competitive soccer season. Cross-lagged path analyses revealed that associations between the two coach motivational styles and athletes' engagement were mediated by psychological need satisfaction. Furthermore, a positive reciprocal association between psychological need satisfaction and engagement emerged over time. This study therefore supports the temporal assumptions underpinning SDT's mediation model but, importantly, evidences a mutually reinforcing interplay between athletes' psychological needs and their engaged behavior.

414	High school athletes' self-determined motivation: The independent and interactive effects of coach, father, and mother autonomy support	Amorose, AJ; Anderson-Butcher, D; Newman, TJ; Fraina, M; Iachini, A	PSYCHOLOGY OF SPORT AND EXERCISE	2016	10.1016/j.psychsport.2016.05.005	S3	SPORTDiscus+Scopus+WoS	Objectives: The purpose of the study was to examine the independent and interactive influences of athletes' perceptions of autonomy support from their coaches, fathers, and mothers on the athletes' self determined motivation. Design: Cross-sectional survey. Method: High school athletes (N = 335; M age =15.75 years; 62.4% female; 84.2% Caucasian) completed surveys assessing the constructs of interest near the end of their season. Results: Hierarchical regression analysis results showed that autonomy support from all three social agents significantly and positively predicted self-determined motivation (R-2 = 0.32), and the two- and three-way interactions significantly added to the prediction (total R-2 = 0.35). Results showed that a relatively high level of self-determined motivation was associated with the perception that at least two of the three social agents provided high levels of autonomy support. Conclusions: The provision of autonomy support from coaches, mothers, and fathers relate to athletes' self-determined motivation both independently and interactively. (C) 2016 Elsevier Ltd. All rights reserved.
415	A season-long examination of the intervention tone of coach-athlete interactions and athlete development in youth sport	Erickson, K; Côté, J	PSYCHOLOGY OF SPORT AND EXERCISE	2016	10.1016/j.psychsport.2015.08.006	S3	WoS	Objectives: Coaches are a primary influence on athletes' development in youth sport (Horn, 2008). However, the intervention tone of coaches' behaviour has not been directly observed. The purpose of this study was to examine associations between the intervention tone exhibited by youth sport coaches and athletes' individual developmental trajectories over the course of a season. Design: Short-term longitudinal study with behavioural observation. Method: Fifty-five athletes and their coaches from five youth volleyball teams were observed at three time points, and the intervention tone of interactive behaviour was systematically coded and organized by coach athlete dyad. Athletes completed measures of the 4C's of athlete development (competence, confidence, connection, character) at each time point, which were used to create individualized developmental trajectories. Person-centred analyses were used to examine associations between athletes' developmental trajectories and their unique interactive experiences with their coach. Results: Cluster analysis revealed the presence of three distinct clusters based on athletes' developmental trajectories: 1) high and increasing, 2) low and decreasing, and 3) moderate and maintaining, with athletes from each team distributed across clusters. Analysis of dyadic interaction profiles revealed significant differences in interactive behaviour between clusters. Conclusions: Results suggest that differences in coach athlete interactive experiences are associated with different developmental trajectories over the course of a season, even for athletes working with the same coach, highlighting the individualized nature of coaches' influence on young athletes. Practical implications for coaches include a critical awareness of their unique interactive relationship with each athlete independently, as well as the importance of fostering these relationships with regard to young people as more than just athletes. (C) 2015 Elsevier Ltd. All rights reserved.
416	Sports Specialization, Part II: Alternative Solutions to Early Sport Specialization in Youth Athletes	Myer, GD; Jayanthi, N; DiFiori, JP; Faigenbaum, AD; Kiefer, AW; Logerstedt, D; Micheli, LJ	SPORTS HEALTH-A MULTIDISCIPLINARY APPROACH	2016	10.1177/1941738115614811	S3	WoS	Context: Many coaches, parents, and children believe that the best way to develop elite athletes is for them to participate in only 1 sport from an early age and to play it year-round. However, emerging evidence to the contrary indicates that efforts to specialize in 1 sport may reduce opportunities for all children to participate in a diverse year-round sports season and can lead to lost development of lifetime sports skills. Early sports specialization may also reduce motor skill development and ongoing participation in games and sports as a lifestyle choice. The purpose of this review is to employ the current literature to provide evidence-based alternative strategies that may help to optimize opportunities for all aspiring young athletes to maximize their health, fitness, and sports performance. Evidence Acquisition: Nonsystematic review with critical appraisal of existing literature. Study Design: Clinical review. Level of Evidence: Level 4. Conclusion: Based on the current evidence, parents and educators should help provide opportunities for free unstructured play to improve motor skill development and youth should be encouraged to participate in a variety of sports during their growing years to influence the development of diverse motor skills. For those children who do choose to specialize in a single sport, periods of intense training and specialized sport activities should be closely monitored for indicators of burnout, overuse injury, or potential decrements in performance due to overtraining. Last, the evidence indicates that all youth should be involved in periodized strength and conditioning (eg, integrative neuromuscular training) to help them prepare for the demands of competitive sport participation, and youth who specialize in a single sport should plan periods of isolated and focused integrative neuromuscular training to enhance diverse motor skill development and reduce injury risk factors. Strength of Recommendation Taxonomy (SORT): B.

417	Rapid Weight Loss Among Adolescents Participating In Competitive Judo	Berkovich, BE; Eliakim, A; Nemet, D; Stark, AH; Sinai, T	INTERNATIONAL JOURNAL OF SPORT NUTRITION AND EXERCISE METABOLISM	2016	10.1123/ijsnem.2015-0196	S3	WoS	Athletes competing in individual sports such as judo are categorized by weight. Before competitions, weight cutting is common. This cross-sectional study was designed to characterize and determine the prevalence of rapid weight loss (RWL) among adolescent judo competitors. Male athletes aged 12- to 17-years old (N = 108) were recruited from local judo teams. Each participant completed a validated questionnaire regarding RWL practices. Anthropometric measurements were also performed. Average age was 14.6 +/- 1.6 years and all participants were of normal body mass index (BMI). RWL was practiced by 80% of the athletes before competition, beginning at an average age of 12.5 +/- 2.2 years with the highest prevalence (similar to 94%) in the oldest group of judoka (16-17.9 years). Precompetition weight loss duration was 8 +/- 5.4 days, with an average weight reduction of 1.5 +/- 1.1 kg. The number of weight loss efforts per athlete in the past season was 2.8 +/- 2.2. RWL was achieved by increased physical activity (82.6%), skipped meals (56.3%), or fasting at least once (47%). Two-thirds of the athletes indicated that their coaches were the most influential figure in their decision to lose weight before competition. RWL is highly prevalent in adolescent judo competitors. The methods used by these athletes can potentially lead to significant health risks including compromised nutritional status, diminished physical performance and impaired growth and development. It is of great importance to insure that those who guide young adults in weight loss for competitive sports have the knowledge and understanding to make safe recommendations and appropriate decisions regarding achieving specific weight goals.
418	Assessing toxic leadership in high-performance sports: Psychometric validation of a Swedish Version of the Toxic Leadership Scale for Sports (TLS- S)	Lundqvist C.,Johansson S.	Plos One	2026		S1	Scopus	This study evaluated the internal consistency and factorial validity of a sport-adjusted 15-item version of the Toxic Leadership Scale (TLS-S). The sample consisted of 189 Swedish elite team sport athletes (56 females, 132 males, 1 preferred not to disclose; mean age=17.9 years, SD=3.3) from 21 different teams (ice hockey n=68; soccer n=60; American football n=26; basketball n=15; handball n=14; volleyball n=3; other team sports not specified or multiple sports n=3). Participants competed at different performance levels: sub-elite (n=15), national junior elite (n=105), international junior elite (n=26), national senior elite (n=30), international senior elite (n=12), and other not specified (n=1). Results showed adequate internal consistency (ωt>.70) for the total TLS-S score and all subscales except the authoritarian leadership subscale (ωt=.61). Confirmatory factor analysis indicated acceptable model fit for both a five-factor and a one- factor model. While the five-factor model (χ2(80)=135.87, p<.001; CFI=.98; RMSEA=.062 [90% CI: .044–.080]; SRMR=.044) was superior to the one-factor solution (difference test: χ2(10)=53.60, p<.001), intercorrelations across subscales were high (range: .73−.92). A bifactor model supported a unidimensional structure (explained common variance=.79; percentage of uncontaminated correlations=.86). The findings provide psychometric evidence for the TLS-S and support a unidimensional structure. This study constitutes an important initial step toward facilitating the quantitative assessment of toxic leadership within sport environments, with the TLS-S emerging as a promising instrument for this purpose.
419	Acceptable or Not: An In-depth Analysis of Adolescent Competitive Athletes' Perceptions on Abusive Coaching Behaviors	Marsollier E.,Hauw D.,von Roten F.C.	Journal of Interpersonal Violence	2025		S1	Scopus	The present study aimed to conduct an in-depth analysis of adolescent competitive athletes' perceptions on abusive coaching behaviors. Our aims were thus t (a) identify the acceptable abusive coaching behaviors and (b) characterize qualitatively the criteria for the acceptance of abusive coaching behaviors. Based o the study goal, an Abusive Coaching Behavior Grid was developed and completed by 356 French-speaking athletes, among which 10 were interviewed to justify where they draw the line between acceptable and unacceptable coaching behaviors. Quantitative analysis showed that shaking, shouting at, or asking athlete to perform until exhaustion were considered acceptable. Quebec and female athletes tended to accept fewer different abusive behaviors, but there were no differences by sport characteristics. The perception on abusive coaching behaviors was influenced by expectations about the coaching role, negative effects of coaching behaviors, circumstances in which the behaviors occur, and the nature of behaviors. The present study raises the importance of questioning cognitiv schemas shared by groups of athletes as well as the norms coaches convey and the behaviors they adopt.
420	Interpersonal climate and moral conduct in competitive sport: A dual-route model with serial mediation	Wang Z.,Liu T.,Hong S.,Wang W.,Wang F.	Plos One	2025		S1+S3	Scopus	Background To investigate how coaching leadership styles influence athletes' moral conduct, this study explored the direct and indirect effects of democratic and autocratic leadership behaviors on prosocial and antisocial behaviors in sport. Method A cross-sectional survey was conducted among 1,239 competitive athletes from multiple sports, measuring leadership perception, goal orientation, and moral disengagement. Key results Democratic leadership significantly predicted higher prosocial behavior and lower antisocial tendencies, while autocratic leadership showed the opposite pattern. Implications These effects were mediated by athletes' task and ego orientation, as well as their level of moral disengagement. The findings highlight the broader relevance of ethical, knowledge-based leadership in cultivating sustainable behavioral outcomes in sports. These results support social cognitive and moral disengagement theory by revealing how internal cognitive-motivational factors transmit leadership influence. The study contributes to both sport psychology and leadership literature by identifying key mediating mechanisms and clarifying the behavioral implications of different leadership approaches. Practically, within the context of Chinese elite sport, the findings suggest that fostering more democratic leadership styles may be associated with more ethical conduct and support athletes' longer-term development, although cross-cultural and longitudinal research is needed before drawing broader causal conclusions.

421	Qualitative analysis of elite football players' causal attribution to not playing: A perspective of attribution theory	Ulas M.,Adilogullari I.,Senel E.	Plos One	2025	S1	Scopus	This study explores the causal attributions of professional football players who experience reduced playing time or exclusion from the squad, focusing on the psychological and relational consequences of these experiences. Using a qualitative thematic analysis approach, semi-structured interviews were conducted with 16 elite football players from Turkey's top league, all of whom had represented the national team. The findings reveal that players predominantly attribut their exclusion to coach preferences and subjective decisions, rather than their performance. This attribution process often leads to negative emotional outcomes, including feelings of exclusion, loss of motivation, diminished self-worth, and identity confusion. The study also highlights that a lack of clear communication from coaches exacerbates these negative emotions and can strain both team dynamics and players' personal lives. Furthermore, the results indicate that prolonged periods of exclusion may result in decreased training effort and a decline in overall performance, creating a negative feedback loop. These findings underscore the importance of transparent communication and supportive coach-athlete relationships in mitigating the adverse effects of reduced playing time. The study contributes to the literature by providing new insights into the underexplored area of athletes' psychological responses to exclusion and offers practical recommendations for coaches to foster athlete motivation and well-being, even in challenging circumstances.
422	Using the Conceptual Framework for Examining Sport Teams to Understand Group Dynamics in Professional Soccer	Gonzalez-Ponce I.,Diaz-Garcia J.,Ponce-Bordon J.C.,Jimenez-Castuera R.,Lopez-Gajardo M.A.	International Journal of Environmental Research and Public Health	2022	S1	Scopus	(1) Background: The aim of this study is to propose a model of the interactions of group dynamics using the conceptual framework to examine sports teams; (2 Methods: The hypothesized model includes measures of group structure (authentic leadership, perceived justice, coaching competency, role clarity/ambiguity, and role conflict), group cohesion (cohesion and team conflict), and group processes (collective efficacy and transactive memory systems). Participants were 581 professional soccer players (M = 24.51, SD = 3.73; 356 males and 225 females) who completed a multisection questionnaire assessing group dynamics variables; (3) Results: The results show that coach leadership predicts coaching competency and perceived justice, and both competency and justice predict role ambiguity and role conflict. Furthermore, role ambiguity and role conflict predict group cohesion and team conflict, whereas group cohesion and team conflict both predict the transactive memory system. Finally, collective efficacy is predicted by the transactive memory system; (4) Conclusions: The results suggest the importance of coach behavior (leadership, justice, and competency) and group processes to improving team functioning in a professional sports context.
423	Examining the psychometric properties of the Controlling Coach Behaviors Scale in Chinese Elite Athletes	Zhao D.,Zhou Y.	Plos One	2022	S1	Scopus	Coaching style is key to athletes' performance and mental well-being. However, few attempts have examined the effects of coaching style on athletes in a Chinese cultural context. Based on previous literature and 23 expert interviews (11 with athletes and 12 with coaches), we rebuilt a 16-items on the Controlling Coach Behaviors Scale. In study 1, 130 provincial team athletes completed a 16-items questionnaire on controlling coach behaviors. The questionnaire items were then screened using exploratory factor analysis and transformed into a formal scale. In study 2, another 560 provincial athletes completed several measures related to coaching style, motivation, subjective vitality, and burnout, and systematic tests were carried out to validate the scale. Study 3 examined the test-retest reliability of the Controlling Coach Behaviors Scale over a 2-week interval. Finally, the present study yielded a nine-item Controlling Coach Behaviors Scale with three dimensions (controlling use of reward, negative conditional regard, and excessive personal control). It suggests that cultural differences played an important role in the communication between athletes and coaches. The new Controlling Coach Behaviors Scale shows good validity and can be used in future research.
424	Quality of coach-athlete relationship and coping as associated factors of stress, anxiety, burnout, and depression symptoms of soccer players in transition to professional: a prospective study	da Silva A.A.,de Sousa Fortes L.,da Cruz Carvalho L.P.,... do Nascimento Junior J.R.A.,Barbieri R.A.	Motriz Revista De Educacao Fisica	2022	S1+S3	Scopus	Aims: The present study investigated the quality of coach-athlete relationship (CAR) and coping as associated factors stress, anxiety, burnout, and depression symptoms of soccer players in the transition to professional. Methods: Twenty-three athletes from four teams of the 2019 under-20 Bahia State Championship participated in the study. The instruments used were the Coach-Athlete Relationship Questionnaire, the Coping Strategic Athletic Inventory, the Daily Analysis of Life Demands in Athletes, the Competitive State Anxiety Inventory-2R, the Burnout Questionnaire for Athletes, and the Major Depression Inventory. Data analysis was conducted using Generalized Estimation Equations, Pearson's Correlation, and Linear Multiple Regression ($p < 0.05$). Results: Our findings show that from the beginning to the end of the season, CAR and coping strategies were predictors of psychic occurrences. For CAR, the symptoms of anxiety associated with self-confidence and stress symptoms were predicted by Proximity and Complementarity, respectively. As for coping strategies, only the symptoms of stress were predicted by the dimension of facing adversity. Conclusion: From the beginning to the end of the season, the magnitude of the predicting factors over some psychopathologies is enhanced in youth soccer players going through a career transition stage.

425	Motivational beliefs and subjective effort in adolescent athletes	Mudrak J.,Slepicka P.,Slepickova I.,Zabrodska K.,Knoblochova M.	International Journal of Sport Psychology	2021	S1	Scopus	The study explored motivational antecedents of subjective effort in adolescent competitive athletes using an integrative approach inspired by the expectancy- value model of achievement motivation as the theoretical framework. In total, 1019 athletes (mean age of 16.5 years) who engaged in various sports from all regions of the Czech Republic participated in the large-scale questionnaire study. We hypothesized two groups of motivational variables as predictors of subjective effort: 1) sport motivation and achievement goal orientations, representing athletes' valuation of sport participation, and 2) self-efficacy and mindset, representing the athletes' expectancies of success. We analyzed the questionnaire data within a multiple regression model. The model was significan ($F(19, 990) = 19.788$, $p < .001$) and explained 27.5 % of the variance in subjective effort the participants reported exerting in their practice. In the model, subjective effort was significantly predicted by integrated regulation (β=.156), amotivation (β=-.143), task orientation (β=.117), and self-efficacy, both directly (β=.243) and in interaction with inborn talent beliefs (β=-.129). These results suggest that the subjective effort of adolescent athletes may be associated with sport-related identity rather than experiences of enjoyment or external rewards and with a focus on skills development rather than on competition. The result provide further evidence for a complex approach to athletes' motivation, suggesting that motivational variables related to task value as well as expectancies o success should be taken into account to create motivational climates supporting effortful practice and subsequent athletic outcomes of adolescent athletes.
426	Which are the most determinant psychological factors in olympic shooting performance? A self-perspective from elite shooters	Moreira da Silva F.,Malico Sousa P.,Pinheiro V.B.,... Refoyo Roman I.,Mon-Lopez D.	International Journal of Environmental Research and Public Health	2021	S1	Scopus	Psychological aspects like anxiety, injuries' effects, professional psychological support (PPS), psychological training (PT), or athlete-coach relationships could influence shooters' performance. This study tried to determine which aspects were critical from the shooters' perspective. Eight elite shooters were interviewe After using qualitative methods, the following categories were obtained: importance of PT; anxiety and competition relationship; mental preparation; PPS; stress during training; injuries' psychological effects and coach's influence. PT is important for shooter's performance, being PPS a key aspect. Moreover, anxiety levels are critical, raising during the pre-competitive period and oscillating during competition. Furthermore, shooters considered more effective the stress placed on training by the coach than by themselves. Accordingly, the coach plays a key role. Surprisingly, injuries did not affect shooters psychologically nor in their competitive performance. We conclude that the combination of PPS, shooter competitive experience and the optimal coach's work can promote a greater performance in Olympic shooting.
427	Perceptions of low back pain in elite gymnastics: A multi-disciplinary qualitative focus group study	Fawcett L.,Heneghan N.R.,James S.,Rushton A.	Physical Therapy in Sport	2020	S1	Scopus	Objectives: To explore the multidisciplinary team experience of Low Back Pain (LBP) in elite gymnastics. Design: A qualitative focus group. Setting: British Gymnastics. Participants: Ten coaching, sports science and medicine multidisciplinary team members working with British gymnasts. Main outcome measure A topic guide informed by literature/expert opinion enabled discussion that was recorded/transcribed verbatim. Initial inductive analytic process developed theoretical insights. Manual coding using constant comparative methods categorised meaningful themes and sub-themes. Results: Two key aspects were identified. Emerging themes for LBP presentation included: early identification LBP and influence of multidisciplinary team members on outcomes; factors influencing LBP reporting e.g. coach-athlete relationship; frequent presentations of LBP and accepted norms; athlete history and physical examination e.g. training load. Emerging themes for causation of LBP included: intrinsic risk factors e.g. growth and maturation; extrinsic risk factors e.g. equipment. Conclusions: Individual responses of a gymnast to experiencing LBP were important across all themes. Some LBP was perceived as normal. The coach-athlete relationship and support team are crucial decision-makers around training load and adaptation. Early detection will help minimise time loss from training/performance to expedite healing.
428	"Coach really knew what i needed and understood me well as a person": Effective communication acts in coach–athlete interactions among Korean olympic archers	Kim Y.,Park I.	International Journal of Environmental Research and Public Health	2020	S1	Scopus	The purpose of this study is to qualitatively explore situations in which athletes perceived communication with their coach to be important and determine the effect of this communication on the athletes. Literature on the communication process in sports emphasizes the distinct characteristics of each sports and its setting. However, previous research has not studied various settings in detail, and archery is yet to be explored. The qualitative process included an in-depth, semi-structured interview with eight Olympic archers. Thematic analysis was used to interpret the data. Athletes perceived communication with coaches to b important during their performance, while dealing with psychological crises, and during their training. Our analysis suggests that, depending on the communicative actions, a coach may positively or negatively impact an athlete's self-awareness, self-confidence, anxiety, autonomy, and motivation. A noteworthy finding of this study is that archers perceive communication with coaches about the selection and management of equipment as important. This study emphasizes the critical role of an athlete's communication with the coach in various situations and discusses the theoretical and practical implications in the context of sports performance.

429	Emotion, psychological well-being and their influence on resilience. A study with semi-professional athletes	Trigueros R.,Aguilar-Parra J.M.,Alvarez J.F.,Gonzalez-Bernal J.J.,Lopez-Liria R.	International Journal of Environmental Research and Public Health	2019	S1	Scopus	The objective of the present study is to analyze the influence of coaches on emotional intelligence and on levels of anxiety, motivation, self-esteem, and resilience among athletes. Five-hundred forty-seven semi-professional athletes between the ages of 16 and 19 participated in this study. Various statistical analyses were conducted which explain the causal relationships between the variables. The results, obtained using a structural equations model, find that while autonomy support positively predicts emotional intelligence, perceived control predicts it negatively. Moreover, emotional intelligence positively predict self-esteem and self-determined motivation, but negatively predicts anxiety. Other results show that self-esteem positively predicts self-determined motivation, whereas anxiety predicts it negatively. Finally, self-determined motivation positively predicts resilience. Indeed, the study demonstrates the influence and the importance of coaches in relation to the emotional intelligence, psychological well-being, and motivational processes of adolescent athletes when the latter engage in their respective sports. These results help to better understand how different behavioral, emotional, and social aspects belonging to the athlete interrelate with one another during competition.
430	Motivation in the practice of judo in non-professional athletes | Motivación en la práctica del judo en deportistas no profesionales	Castro-Sanchez M.,Zurita-Ortega F.,Zafra-Santos E.,... Chacon-Cuberos R.,Valdivia-Moral P.	Revista Internacional De Medicina Y Ciencias De La Actividad Fisica Y Del Deporte	2019	S1	Scopus	The aim of this study is to determine and analyse the relationships and differences in the motivational climate, based on the competitive level in non- professional judokas. A sample of 121 non-professional Chilean judokas (70 men and 51 women) aged between 18 and 40, coming from seven different judo clubs took part. They completed a self-registration sheet of sociodemographic variables and a motivational climate questionnaire (PMCSQ-2). The results indicate that the task goal orientation prevails in non-professional judokas and as the level rises, in amateur athletes the ego goal orientation takes on greate importance. The main deduction is that amateur judokas distinguish in a greater proportion between personal improvement and effort (task goal orientation) and show a greater physical ability than others.This is opposed to what happens with non-professionals, where the correlation between both is lower, althoug still negative.
431	Using vignettes to analyze potential influences of effective strength and conditioning coaching on athlete development	Szedlak C.,Smith M.,Day M.,Callary B.	Sport Psychologist	2018	S1	Scopus	This study explored which strength and conditioning (S&C) coaching behaviors and characteristics are perceived as effective by elite athletes and how these influence the athletes. A secondary aim was to consider the development and usefulness of vignettes to elicit new knowledge. Ten elite athletes reflected on scenarios presented in vignettes. Resulting themes were divided into the processes and factors influencing athletes and how the athletes are affected. The athletes considered these themes effective because the coach had built an environment of trust and respect. How coaches might influence athletes were divided into cognitive influences and behavioral influences. The results are discussed in light of current sport coaching literature, and the way vignettes enhance the richness of the data collection is reflected on. Practically, the results suggest that S&C coaches can build trust and respect to influence athletes' development through effective instruction, communication, and motivation.
432	Providing social support to female Olympic athletes	Poucher Z.A.,Tamminen K.A.,Kerr G.	Journal of Sport and Exercise Psychology	2018	S1	Scopus	Support providers may experience positive and negative outcomes associated with supporting others. However, there is a lack of research on support provision to elite athletes and the views of athletes' support providers. This study addressed this gap by exploring the experiences of providing and receiving support between female Olympians and their main support providers. Five female Olympians and their main support providers participated in separate semistructured interviews. It appeared that support provision was personally and professionally rewarding, as well as challenging, for support providers, and athletes were generally satisfied with the support they received. Athletes appeared highly dependent on their support providers, but both athletes and support providers felt that high levels of support were necessary for athletic success. Further research is needed to understand how support providers are able to foster their own personally supportive relationships and whether high levels of interpersonal dependence are required to achieve athletic success.
433	Influences of perfectionism and motivational climate on attitudes towards doping among Korean national athletes: A cross sectional study	Bae M.,Yoon J.,Kang H.,Kim T.	Substance Abuse Treatment Prevention and Policy	2017	S1	Scopus	Background: The motives for elite athletes to dope are related primarily to maintaining and improving their physical performance. Especially, elite athletes training to compete in the Olympics may feel unique situational pressure, which may in turn induce powerful motivation for doping and predict doping behavior. This study aimed to investigate possible factors associated with attitudes towards doping in Korean national athletes who competed in the Rio 2016 Olympic Games. Methods: A total of 198 athletes (95 female, 103 male) completed the questionnaire, which covered demographic information, doping-related experiences, Performance Enhancement Attitude Scale (PEAS), Perfectionism in Sports Scale (PSS; coach's criticism, concern over mistakes, and personal standards), and Perceived Motivational Climate in Sport Questionnaire-2 (PMCSQ-2; ego-involving and task-involving climates). Pearson's correlation coefficients were used to identify correlations among PEAS, PSS, and PMCSQ-2 scores, and stepwise multiple linear regression was performed to investigate possible factors significantly associated with attitudes towards doping. Results: The coach's criticism of PSS was slightly or weakly related to the concern over mistakes of PSS and the ego-involving climate of PMCSQ-2, respectively. And the concern over mistakes sub-scale of perfectionism was related to attitudes towards doping, but weakly. Conclusions: Effective anti-doping policy should meet athletes' perfectionism, and more studies that identify other factors that influence athletes' doping attitudes are needed.

434	Pathos and orchestration in elite sport: The experiences of NCAA DI student- athletes	Raabe J.,Readdy T.,Zakrajsek R.A.	Sport Psychologist	2017	S1	Scopus	Coaching is characterized by an inherent pathos between the goals coaches hope to accomplish and those that are realized (Jones & Wallace, 2005). Coaches can actively enhance the likelihood of optimal outcomes through orchestration, a process of incremental coping intended to create improvement in performance (Jones & Wallace, 2005). The current study explored to what extent pathos also manifests in the lives of elite athletes and whether they engage in processes consistent with orchestration. Semistructured interviews were conducted with 12 National Collegiate Athletic Association Division I student- athletes. Primarily deductive analysis of the qualitative data provided confirmation for four domains: (a) sources of ambiguity created by coaches, (b) other sources of ambiguity within student-athletes' experiences, (c) attempted strategies for orchestrating the pathos, and (d) relationships are crucial for navigating the pathos. The findings potentially offer an approach to understanding the challenges athletes face, which allows coaches to more accurately provide assistance.
435	‘This might be him; the guy I'm gonna marry’: Love and sexual relationships between female elite-athletes and male coaches	Johansson S.,Larsson H.	International Review for the Sociology of Sport	2017	S1	Scopus	Infatuation, love and sexual relationships exist virtually anywhere. Coach–athlete sexual relationships (CASR), however, are overlooked and under-researched. Within sport sociology, CASR have been framed predominantly by a sexual abuse discourse. Informed by Foucault's discourse analysis, this study explores how discourses regarding performance enhancement in elite-sport and coaching, and romantic love, frame female elite-athletes' experiences with CASR. Interview with four female elite-athletes aged 26–30 were conducted. The results indicate that CASR are potentially problematic because they intersect and challenge discourses comprising elite-sports, coach–athlete relationships, female sexual agency, and love. Moreover, discourses of power differ between the professiona and private contexts. While the athletes expect their coaches to exert dominance and control in the elite-sport context, love relationships are about equally and mutually giving away power and control. Although CASR can facilitate motivation and performance, framing CASR as inherently unequal and abusive can contribute to stigmatisation, secrecy and athlete disempowerment.
436	Offended by the algorithm: The hidden interpersonal costs of clients seeking AI second opinion	Spassova G.,Palmeira M.	Computers in Human Behavior	2026	S2	Scopus	Rapid advances in artificial intelligence have enabled the rise of AI-enabled advisory tools. While these tools benefit decision-makers, they also introduce new competitive pressures for human advisors whose expertise they may complement or replace. Prior research shows that advisors react negatively whe clients approach other advisors, feeling offended and becoming less willing to maintain the relationship. Yet little is known about how advisors respond when the other advisor is an AI system rather than a human. Across four studies, we examine how professionals perceive and react to clients who consult AI-enabled (vs. other human) advisors. We find that learning a client has also sought AI (vs. other human) advice decreases focal advisors' motivation to work with that client. This effect persists even when clients use AI only for background information or as a complementary resource. We propose that advisors view AI as substantially inferior to themselves; thus, being placed in the same category as an AI system feels insulting and signals disrespect, undermining advisors' willingness to engage. We also show that consulting AI may change perceptions of the client, making them appear less competent and warm. Our work contributes to emerging research on the advisor perspective and extends the literature on human responses to AI by shifting attention from AI users to service providers. Practically, the findings suggest that clients' seemingly innocuous use of AI tools may unintentionally erode their relationships with human advisors.
437	One Sprinter, Two Olympic Preparations: A Single-Athlete Longitudinal Observational Study of Training-Intensity Distribution and Implications for Future 50 m Events	Papadimitriou K.,Margaritelis N.V.,Tsalis G.	Sports	2026	S2	Scopus	Purpose: This single-athlete, longitudinal observational study describes training intensity distribution (TID) across two Olympic preparation cycles (Rio 2016 vs. Tokyo 2021) and explores whether differences in high-intensity exposure coincided with performance outcomes. Methods: An elite male 50 m freestyle specialist (personal best 21.27 s; height: 187 cm, weight: 80 kg, body mass index: 22.9 $kg \cdot m^{-2}$, fat-free mass: 75.2 kg, and fat mass: 4.8 kg) was monitored across four mesocycle periods. TID is expressed as % of total swim volume in three zones: Z1 (low intensity), Z2 (threshold), Z3 [high intensity/race-pace, including High Intensity Interval Training (HIIT) and Sprint Interval Training (SIT)]. Both the coach and swimmer signed a written informed consent for the use of their data. Results: For Rio 2016, TID (Z1/Z2/Z3) was as follows: General 80/0/20, Specific 60/0/40, Pre-competition 40/30/30 and Taper 50/20/30, indicating a polarized approach. For Tokyo 2021, TID shifted to: General 85/0/15, Specific 60/0/40, Pre-competition 30/30/40, and Taper 40/20/40. Discussion: In this single athlete, a greater proportion of work in Z3 during the Tokyo cycle, particularly in the Pre-competition and Taper phases probably coincided with improved performance (21.57 vs. 21.79 s). Conclusions: Although clear causal inference is not possible, these observations depict the probability that sprint-swim preparation for 50 m events needs a training volume oriented to Z3 and relatively less in Z1. However, the study's design, the methods by which the TID was recorded, etc., limit any generalization about the interpretation of the findings. Therefore, future studies should addres these limitations, providing more insights into improving the training on that kind of events.

438	Training Stress, Sleep Behaviour, and Recovery Self-Regulation in Elite Female Boxers: A Behavioural Pattern Analysis Using Data-Driven Methods	Rattanasateankij W.,Senakham T.,Punthipayanon S.,Puttasimma T.,Senakham N.	Journal of Mind and Behavior	2025	S2	Scopus	Elite boxing performance is significantly affected by training load, sleep habits, and the regulation of recovery; however, traditional monitoring approaches tend to assess these components separately and largely depend on subjective observations from coaches. Consequently, evaluating athlete readiness solely through movement analysis is insufficient, restricting both performance enhancement and the early detection of fatigue. This study investigates behavioural patterns indicative of readiness in elite female boxers and develops a data-driven classification framework that integrates physiological and behavioural metrics with boxing action execution. Data were obtained from competitive female boxers during the pre-competition phase. Training load was quantified, sleep was assessed using wearable-derived duration and efficiency measures, and recovery regulation was evaluated through standardised recovery questionnaires. Psychological dimensions, including competitive anxiety, self-confidence, team cohesion, and perception o opponents, were also recorded. A dataset containing 5,200 labelled boxing actions underwent pre-processing involving Gaussian noise filtering and feature normalisation. Principal Component Analysis (PCA) was employed to reduce data dimensionality while preserving critical structures. To capture behavioural patterns, a Dynamic Monarch Butterfly Optimized Attention-Vision AutoEncoder (DMBO-Att-VAE) was introduced, integrating behavioural an spatiotemporal motion features, followed by SoftMax classification with a train–test split. The DMBO-Att-VAE framework, implemented in Python, successfully fused behavioural and psychological metrics with spatiotemporal features, achieving a classification accuracy of 98.76% and an F1-score of 97.52%. This data-driven approach effectively identifies behavioural indicators of readiness, providing an interpretable tool to monitor performance and optimise training strategies in elite female boxing.
439	Strategies to reduce hyperglycemia-related anxiety in elite athletes with type 1 diabetes: A qualitative analysis	Katz A.,Shulkin A.,Fortier M.-A.,... Rabasa-Lhoret R.,Brazeau A.-S.	Plos One	2025	S2	Scopus	Objective Managing blood glucose levels is challenging for elite athletes with type 1 diabetes (T1D) as competition can cause unpredictable fluctuations. While fear of hypoglycemia during physical activity is well documented, research on hyperglycemia-related anxiety (HRA) is limited. HRA refers to the heightened fear that hyperglycemia-related symptoms will impair functioning. This study investigates current strategies employed to mitigate HRA during competition and the development of alternative approaches. Research design and methods Elite athletes with TID, aged >14 who self-reported HRA durin competition were recruited. Elite athletes were defined as individuals exercising >10 hours per week whose athletic performance has achieved the highest competition level. 60 to 90-minute virtual semi-structured interviews were analyzed using an Interpretative Phenomenological Analysis. Results Ten elite athletes with T1D (average age 25 ± 3 years; T1D duration 12 ± 8 years; number of competitions per year 27 ± 19; training time per week 12 ± 6 hours) reported the strategies they currently use to mitigate HRA. These strategies include managing insulin and nutrition intake, embracing social support networks, using technology, practicing relaxation techniques, establishing routines, performing pre-competition aerobic exercise, and maintaining adequate sleep hygiene. Several additional approaches that could be implemented were identified including establishing targeted support networks, developing peer-reviewed resources on HRA, ensuring support teams have sufficient tools, and improving existing technology. Conclusions Elite athletes with T1D use physiological and psychological strategies to mitigate HRA during competition. This finding highlights the need for increased support and education for these athletes, and advancements in technology. A multidisciplinary approach involving healthcare professionals, athletic staff, and peer mentors could help integrate personalized anxiety management and diabetes care strategies into training regimens, enhancing both mental resilience and performance outcomes for athletes with T1D.
440	Sport Anxiety, Fear of Negative Evaluation, Stress and Coping as Predictors of Athlete's Sensitivity to the Behavior of Supporters	Gabrys K.,Wontorczyk A.	International Journal of Environmental Research and Public Health	2023	S2	Scopus	The aim of the study was to find predictors of athlete sensitivity to the positive or negative behaviors of supporters and determine a relationship between athletes' sensitivity to the positive and negative behaviors of supporters and traits such as anxiety and stress or the strategies used to cope with stress. Th sample consisted of 171 professional athletes. The study determined three variables that are predictors of athlete sensitivity to the positive behavior of supporters (SPS), i.e., strategies of coping with stress, such as high levels of coachability, confidence and achievement motivation and low levels of freedom from worry (change R2 = 0.15, F of change = 9.78, p of change < 0.001). The predictors of sensitivity to the negative behavior of supporters (SNS) are as follows: a low level of freedom from worry and a high level of fear of negative evaluation (change R2 = 0.31, F of change = 38.56, p of change < 0.001). In the context of the athlete's position, the predictors of SPS for midfielders are as follows: high level of susceptibility to stress and two strategies of coping with stress, i.e., peaking under pressure and concentration. For forwarders they are as follows: high level of worry and coping with stress via goal-setting, while for defenders, the ways of coping with stress are confidence and achievement motivation. In terms of SNS, for defenders, the predictors are low levels of freedom from worry, coachability, and a high level of fear of negative evaluation. Forwarders, who are sensitive to the negative behavior of supporters, ar characterized by a fear of negative evaluation.

441	Depressive symptoms and burnout in football players: A systematic review	Sarmento H.,Frontini R.,Marques A.,... Campos M.J.,Clemente F.M.	Brain Sciences	2021	S2	Scopus	The purpose of this article was to systematically review and organise the available literature devoted to the topic of depressive symptoms and burnout in football players. A systematic search was conducted in Web of Science, Scopus, SPORTdiscus, PubMed, and Psychinfo for articles published up to June 2020 The searches yielded 1589 articles, and after the screening process, a total of 18 studies met the eligibility criteria and were included for review. Playing position and conflicts with coach/management seems to have a direct influence on the prevalence of depressive symptoms in current players as do the injuries and life events of former players. During the pre-competition phase, most of the athletes displayed reduced rates, indicating burnout. An exploration of the mental health of football players will help to create models of care and guide professionals so that they may help players achieve better performance while also having better wellbeing. Understanding how to prevent and cope with the emotional wellbeing of football players will be possible to guide players and coaches.
442	PoseShot: hybrid CNN–BiLSTM transformer model for free throw action recognition via pose analysis	Hsu W.-C.,Lee C.-C.,Lee Y.-H.,... Ho C.-C.,Chang Y.-C.	Scientific Reports	2026	S3	Scopus	The evaluation of basketball free throw techniques has traditionally relied on subjective assessments, which introduces inherent biases and inconsistencies in performance analysis. This study presents PoseShot, a novel dual channel hybrid CNN-BiLSTM-Transformer Model that facilitates the comprehensive analysis of free throw mechanics with data-driven insights. Unlike conventional human activity recognition that focuses on coarse activity labels, PoseShot is designed to analyze fine-grained, phase-dependent mechanics within a single basketball free throw motion. The proposed framework integrates training footage with precise body posture angle calculations via a dual-channel deep learning architecture to enable the capture of subtle technical variations. Our innovative approach synthesizes convolutional neural networks (CNN) for spatial feature extraction, bidirectional long short-term memory (BiLSTM) for temporal sequence processing, and a transformer encoder for enhanced contextual understanding of motion dynamics. The model demonstrates exceptional performance, achieving an F1-score of 95.76%, precision of 95.72%, and recall of 95.80%. These metrics surpass the performance of established architectures, including DenseNet, Swin Transformer, and Vision Transformer, particularly for the analysis of complex throwin motions. By incorporating both spatial features and postural dynamics, PoseShot provides accuracy in motion analysis. Empirical evaluation reveals PoseShot's capacity to identify crucial biomechanical determinants of successful free throws, thus offering quantifiable insights for performance enhancement. Since the model's analysis elucidates the intricate relationship between posture optimization and action consistency, it can provide actionable guidance for athletes and coaches. This research bridges the gap between subjective evaluation methods and advanced motion analytics, establishing PoseShot as a transformative tool in sports performance analysis. The findings demonstrate the potential for data-driven approaches to revolutionize basketball training methodologies through precise, objective assessment criteria.
443	Prediction of athlete performance based on a gradient regression model	Wei X.,Liang S.,Diao W.	Scientific Reports	2026	S3	Scopus	Accurate prediction of athlete performance is a challenges issue of significance in sports science and analytics and has application in training design, injury prevention, and talent management. Conventional statistical models usually cannot represent nonlinearities that are highly intricate in terms of physiological, lifestyle, and contextual characteristics. The main aim of the study is the prediction of performance (predictive validity/generalization) of th performance scores of the athletes based on the tabular data. An interpretability is a secondary objective that is tackled through SHAP-based explanations, and computational efficiency is considered as the practical aspect of the selected model class; deployment feasibility is only presented as a possible application, not as a proven real-world application. The main objective is to offer a dependable platform of determining the main determinants o athlete performance and data-driven decision making in sports coaching. Kaggle Athlete Performance Prediction Dataset was used, which included demographic, training, physiological, and/or lifestyle features. Imputation, normalization, encoding, and feature engineering then followed, and finally, partitioning into training, validation, and test sets. Gradient Regression Model was trained with tenfold cross-validation and compared against various baseline which included linear regression, Ridge Regression, Support Vector Regression (SVR), Random Forest, and Neural Networks. Measures of evaluation wereR^2, RMSE, and MAE. The proposed model had an R^2 of 0.923, which was higher than baselines, which included Neural Networks ($R^2 = 0.901$ and Random Forest ($R^2 = 0.887$). The presence of residual and error analysis ensured that bias and variance were reduced to a minimum, whereas learning dynamics showed that convergence and stability were achieved efficiently. The Gradient Regression Model is better at predicting and is also more interpretable, with applications that are applicable to individualized training and performance monitoring. This study is presented as an application and evaluation work with a publicly available dataset, with its contribution being a transparent end-to-end pipeline, strict validation, and analysis that relate predictive factors to the performance of athletes, as opposed to a new learning algorithm. The future research will cover larger and longitudinal studies along with the hybrid frameworks that incorporate both biomechanical and psychological aspects. Discover early research ideas View preprints published by authors to have an early idea of upcoming research documents.

444	The development of mental toughness: A journey into the perceptions and experiences of a world-class padel athlete	Barreiros A.,Maia M.,Fonseca A.M.,Dias C.	Performance Enhancement and Health	2026	S3	Scopus	This study retrospectively explored the developmental factors underlying mental toughness in a world-class female padel athlete, examining how it evolved throughout her elite sport career. Using a case study approach, data were collected through a semi-structured interview organised around four stages (early years, middle years, professional years, and maintenance years) and informed by established models of athlete development and mental toughness. Data were analysed using a combined deductive and inductive approach. The findings highlight the athlete's early adoption of mental skills and routines, the significant impact of sport psychology support, and the ongoing motivational influence of parents and coaches. Balancing academic and elite sport demands involved substantial sacrifices, which the athlete described as important for developing mental toughness. She also explained how ke moments, both positive and negative, contributed to strengthening resilience over time. Overall, the results suggest that mental toughness is dynamic an shaped by context, developing through ongoing interactions between individual, social, and environmental factors rather than a fixed trait. The findings also support the view that mental toughness can be trained and developed over time, and underline the value of structured psychological support, social influences, and how athletes cope with adversity in elite sport.
445	Performance inflation in junior tennis: Longitudinal analysis and Bayesian forecasting of ranking thresholds, efficiency, and access equity	Bozdech M.	Plos One	2026	S3	Scopus	This study examined long-term trends, systemic dynamics, and predictive trajectories of junior male tennis performance within the International Tennis Federation (ITF) World Tennis Tour Juniors between 2004 and 2024. Using a longitudinal dataset of 8,082 ranked players, percentile-based performance thresholds (P90, P75, P50) were first derived from annual Total Ranking Points (TRP) distributions to represent the 90th-, 75th-, and 50th-percentile cut-offs i the ITF ranking system. These empirically determined thresholds were then used as reference criteria in binary logistic regression models to estimate corresponding performance boundaries for both TRP and Points per Event (PPE)—the latter serving as an efficiency-adjusted indicator derived from the ratio of TRP to the number of tournaments played. Exponential trend fitting and Bayesian time-series forecasting (Prophet) were subsequently applied to model long-term trajectories and project performance standards for the 2025–2029 period. Findings revealed a sustained and predominantly exponential increase in both TRP and PPE percentile thresholds, indicating that competitive attainment in junior male tennis has followed an accelerating trajectory. However, growth patterns differed in magnitude: TRP thresholds exhibited consistently steeper gradients than PPE thresholds across all percentile tiers, indicating that performance inflation appears to be more strongly associated with cumulative ranking-point expansion than with uniform increases in per-event scoring efficiency, although these indicators remain strongly correlated and should therefore be interpreted as complementary rather than independent mechanisms. Although efficiency-adjusted thresholds also demonstrated upward drift—particularly at the elite level (P90)—the magnitude o this growth remained comparatively moderate. Geographic analysis further identified a persistent concentration of elite players and tournaments within limited group of nations, highlighting systemic inequities in access to ranking opportunities. Forecasts suggest that percentile cut-off thresholds will continue to rise over the next five years, reinforcing the need for adaptive performance strategies. For coaches, federations, parents, and other key stakeholders, sustainable athlete development will depend on balancing competition access and efficiency optimisation with adequate recovery, educational commitments, and psychosocial well-being. Collectively, these results position "performance inflation" in junior tennis as a multidimensional phenomenon
446	The Lions Sports Academy TackleTEK Tool: The intra-and inter-coach reliability of assessing tackling competency in rugby union	Owen R.,Knight C.J.,Page W.,... Kilduff L.,Waldron M.	Plos One	2026	S3	Scopus	Tackling skill tests in rugby aim to evaluate skill competency, but their reliability to detect meaningful changes in tackling competency over time remains uncertain. This study aimed to: 1) determine the intra- and inter-rater reliability of scoring composite tackle competency by three youth grassroots rugby coaches (tier one, two, and three), and 2) assess reliability in scoring across tackling subcomponents (pre-contact, contact, and post-contact) using the TackleTEK Tool. Pilot data was used to develop a-priori analytical goals to assess the reliability of composite and subcomponent scoring. Non-parametric 95% Limits of Agreement were used to assess reliability. Twenty-five participants were recorded performing the one vs. one tackling assessment. The thre coaches scored each tackle against pre-defined 18-point criteria. Systematic differences were assessed using Wilcoxon tests. Intra-coach composite tackling scores demonstrated agreement levels of 72% (95% CI 60% to 84%) for the tier three coach, and 100% (95% CI 100% to 100%) for both the tier two and one coaches, relative to the analytical goal. Inter-coach composite tackling agreement ranged from 78% (95% CI 67% to 89%) to 100% (95% CI 100% to 100%). Intra-coach subcomponent analysis showed the tier one and tier two coach to score all tackling subcomponents consistently (P> 0.05); however, the tier three coach only reliably scored the pre-contact phase. Subcomponent agreement ranged from 68% (95% CI 55% to 81%) to 82% (95% C 71% to 93%), between tier three and tier one coaches, to 72% (95% CI 60% to 84%) to 98% (95% CI 94% to 100%), between tier two and tier one coaches. Within the context of this study, agreement was strongest for tier one and tier two coaches, supporting the use of the TackleTEK Tool to assess changes in tackling competency over time, with further work required to extend its application across coaching levels.

447	An intelligent taekwondo coaching system based on augmented reality technology with real-time feedback mechanisms	Yang F.,Wang Z.	Scientific Reports	2025	S3	Scopus	Traditional taekwondo training methods face limitations in providing objective, real-time feedback for technique improvement, relying primarily on subjective instructor observations that may lack precision and consistency. This research presents an innovative intelligent taekwondo coaching framework that integrates augmented reality technology with advanced motion analysis algorithms to deliver comprehensive, real-time training feedback. The system employs a modular architecture incorporating multi-modal sensor data acquisition, deep learning-based pose estimation, biomechanical analysis, and immersive AR visualization to create an interactive training environment. The motion recognition module utilizes convolutional neural networks specifically adapted for taekwondo techniques, achieving recognition accuracies exceeding 95% across nine fundamental technique categories with processing latencies below 25 milliseconds. The comprehensive evaluation framework assesses movement quality across eight dimensions including geometric accuracy, temporal coordination, and force generation, providing personalized feedback through AR overlays. Experimental validation with 47 practitioners across novice, intermediate, and advanced skill levels demonstrates significant improvements in learning efficiency and technique standardization compared to conventional training methods. User experience evaluation reveals high satisfaction ratings (average 8.5/10) across interface usability, feedback clarity, and learning effectiveness. The system successfully addresses critical gaps in martial arts instruction by democratizing access to high-quality technical guidance while maintaining the engagement and motivation essential for skill development This research establishes important foundations for next-generation sports training technologies and provides valuable insights for developing similar intelligent coaching systems across diverse athletic disciplines.
448	Adverse competition-related cognitions and it's relation to satisfaction and subjective performance: a validation study in a sample of English-speaking athletes	Alena M.-K.,Michele W.,Stefan B.	Scientific Reports	2025	S3	Scopus	This study aimed at examining the reliability and validity of the Adverse Competition-related Cognitions Questionnaire (ACCQ) in an English-speaking sample of athletes. The ACCQ is a performance-focused measure that captures six different areas of adverse competition-related cognitions– athletic comparison, coach devaluation, devaluation of one's own performance, appreciation by coach and family, inner resistance against competitions, and general exhaustion. Data from 278 athletes (Mage = 27.64, age range = 16–68 years) from different sports were collected and subjected to confirmatory factor analysis, which confirmed the 6-factor solution of the translated ACCQ (CFI = 0.915; RMSEA = 0.056). In addition, the different subscales of the ACCQ showed sufficient internal consistency (Cronbach's alpha > 0.60). Furthermore, we examined the relationships with cognitive interference, satisfaction in three different domains (i.e., general in life, sporting development, and athletic performance) and athletes' subjective performance evaluations (i.e., performance and peak performance in the previous season and confidence in achieving their goals in the upcoming season). Results indicated positive correlations with athletes' cognitive interference (i.e., construct validity in terms of a nomological network), low negative relations with athletes' satisfaction in the three different domains and with the three parameters of subjective performance evaluation (i.e., concurrent validity). Implications of these findings and perspectives for future research are discussed.
449	Investigating sport persistence through the development of the Sport Persistence Questionnaire	Kovacs K.E.,Csukonyi C.	Scientific Reports	2025	S3	Scopus	Regarding sporting activity, one of the most important indicators of effectiveness may be sport persistence, a complex concept, a terminology referring to the mastery, form, level and efficacy of sporting activity. It encompasses our attempts concerning the performance plateau, failures, injuries, or even the resolution, processing and utilisation of stressful situations associated with success and positive events, combining the physical, mental and social aspect of sport. In our study, we aimed to describe the development of a tool to measure sport persistence. Accordingly, our research involved a multi-step process. Based on the experience of a preliminary research [N = 1165], a sport persistence principal component was created including four items, which was a reliable but not nuanced variable. To provide a nuanced picture of sport persistence, a 95-item long questionnaire was developed and tested during the pre-test [N = 1105], which was revised and analysed during the post-test [N = 212]). The results of both the pre-testing and post-testing phases indicated that the 13-item Sport Persistence Questionnaire (SPQ) instrument, which scores items on a five-point Likert scale, is reliable for measuring sport persistence (Cronbach's α = 0.943). The indicators of the model fit were also found to be adequate. The instrument measures the level of sport persistence in one dimension. In conclusion, the findings of this study may be useful concerning tailored interventions in fostering sport persistence, applicable for coaches, educators and policymakers as well. The instrument provides a sound basis for further research. In the future, the investigation of sport persistence of competitive and recreational youth athletes will be available to detect the characteristics of sport persistence, including the qualitative, quantitative and longitudinal analysis of individual, micro, meso-, exo- and macro-level factors influencing sport persistence and intervention programs supporting persistent sporting behaviour.

450	Analyzing the impact of prolonged pauses on soccer performance	Atici A.R.,Kamis O.,Edriss S.,... Bowman T.G.,Nobari H.	Scientific Reports	2025	S3	Scopus	During breaks or playing period changes, players' accumulated workload, physiological alterations, body composition factors, sleep, and health-related qualities can factor into soccer performance. This research examined the impact of prolonged pauses, including breaks due to the 2022 FIFA World Cup and an earthquake tragedy, on running displacements and ball in-play duration during the 2022/23 Turkish Super League season. The secondary purpose was to determine the relationship between performance metrics and competition outcomes (i.e., wins, losses, or draws). 550 players from 19 Turkish Super League teams were included. Performance metrics such as running speed, accelerations/number of speed actions, total field coverage, and match outcomes (i.e., win, lose, or draw) were analyzed before and after significant pauses in play. We also explored correlations between running speed, numbe of speed actions, match duration, and total distance covered to understand their influence on soccer performance. Significant differences existed in performance metrics between periods, with higher total displacement and running observed after the World Cup ($p < 0.05$). Additionally, teams exhibiting more static behavior during matches were likelier to lose points, while those running at higher speeds ($p = 0.007$) and acceleration ($p = 0.008$) had a higher winning probability. The findings provide valuable insights into the factors affecting success or failure in soccer matches and have implications for trainin and preparation strategies in professional soccer. In addition, findings illustrate how breaks influence performance metrics and highlight the challenges coaches face in maintaining player readiness during unplanned pauses. This suggests minimizing such disruptions to support optimal training and preparation strategies in professional soccer.
451	Enhancing team dynamics: Unveiling synergy effects through social network analysis in professional basketball	Wei B.,Li Z.,Zhou C.,Gomez M.-A.,Zhang M.	Acta Psychologica	2025	S3	Scopus	The objective of this study was to: (1) redefine player roles of Chinese Basketball Association (CBA) players using a clustering model, grounded in offensive and defensive Play Type data; (2) Utilize social network analysis (SNA) to examine the connections and synergy effect between player positions in lineups that demonstrate offensive excellence, defensive excellence, and net rating excellence. The sample gathered included 20 clubs throughout five seasons (2018–2019 to 2022–2023) of the CBA, which included 1813 players and 22,661 samples of lineup data. The clustering results of the study classified CBA players into 19 distinct player roles. Further analysis revealed that: (1) only ten player roles were highly correlated with excellent lineups; (2) the presence o the C6 Core Ball Handler enhanced the performance of most teammates; (3) the C1 Off-Ball Shooter's performance heavily depended on the interaction with the C6 Ball Handler and the C2 Versatile Big; and (4) the C3 Ball Dominant Scorer negatively impacted the performance of all other player roles. Thes results reveal new insights into players' fingerprints and offer coaches a fresh perspective on team composition.
452	Adaptation and psychometric properties of Psychological Skills Inventory for Sport (PSIS-R5) in Latvian athletes: Insights and implications for practice	Volgemute K.,Ulme G.,Perepjolkina V.,... Tihija A.,Grants E.	Plos One	2025	S3	Scopus	Background and objective Psychological skills are critical for high achievement and elite sports performance. However, there remains a lack of valid, reliable, and accessible instruments adapted to modern athletes. This highlights significant psychometric challenges in the existing tools currently available. While the Psychological Skills Inventory for Sport (PSIS-R5) is a commonly used tool in sports psychology worldwide, it has demonstrated various psychometric issues over time. A previous adaptation to the Latvian sports environment highlighted specific challenges in the Latvian context. It is important to note that these issues are not unique to the Latvian adaptation. A comprehensive review is necessary to address these broader psychometric concerns and ensure the instrument's suitability for contemporary athletes, including those in Latvia. Moreover, the adaptation process may offer insights and methodological guidance for similar efforts in other cultural contexts. The aims of this research were twofold: first, to review and adapt the PSIS-R5 for use in Latvia (PSIS-R5-L), ensuring its validity and reliability; and second, to assess the applicability of the PSIS-R5-L in elite sports settings by examinin relationships between athletes' achievement levels and psychological skills. Methods A total of 444 Latvian athletes aged between 18–43 ($M = 21.32$; $SD = 6.52$) completed the PSIS-R5 inventory to assess their psychological skills. The PSIS-R5 was re-translated into Latvian language (PSIS-R5-L) using forward- backward translation. Additionally, five sports psychologists participated in the study, providing evaluations of elite athletes' psychological skills using the PSIS-R5 inventory. Results Series of Principal Components Analyses (PCA) were conducted to explore and validate the structure of the Latvian revisited adaptation of the PSIS-R5-L. As a result, a stable four-factor structure was obtained, consisting of 17 items with satisfactory fit indices ($CFI=0.968$, $RMSEA=0.045$ [90% CI: 0.034, 0.057], $SRMR=0.026$). The findings indicate that the Latvian adapted PSIS-R5-L has appropriate psychometric properties, confirming its suitability for use in the Latvian sports environment. Comparisons between different athlete groups (elite, pre-elite, and amateur) revealed several correlations and statistically significant ($p<0.05$) differences in psychological skills. Conclusions The PSIS-R5-L is a psychometrically robust and practically valuable tool for assessing athletes' psychological skills in Latvia. It offers coaches, sports psychologists, and practitioners an

453	Longitudinal relationships between emotional processes and perceived performances among athletes: The moderating effect of emotional intelligence	Levillain G.,Martinent G.,Saby Y.,Nicolas M.	Performance Enhancement and Health	2025	S3	Scopus	Objectives: Based on emotional intelligence (EI) theory and cognitive motivational relational theory of emotions, this study aimed to explore longitudinal relationships between the core constructs of the emotional process (appraisals, coping and emotions) and perceived performances during competition an to examine the moderating role of EI on these relationships. Methods: A total of 453 athletes (Mage= 20.6; 117 females and 336 males) from different sports and different competitive levels participated in the study. Appraisals, coping, emotions, and perceived performances were assessed after five competition across the season and EI was assessed at the beginning of the competitive season. Hierarchical linear modelling analyses were conducted to: (a) Evaluate the mediational role of coping strategies on the appraisals – emotions relationships and on the appraisals – perceived performances relationships; (b) explore the mediational role of emotions on the appraisals – perceived performances relationships; and (c) test the moderating role of EI on these relationships. Results: The results showed that: (a) coping played a mediational role on the appraisals-emotions relationships; (b) emotions mediated the appraisals-perceived performances relationships; and (c) EI moderated the aforementioned relationships between the core constructs of emotional proces and perceived performances. Conclusions: As a whole, this study furthered knowledge about the ways the core emotional constructs are interrelated and impact perceived performances within competitive settings. Through coping strategies and cognitive appraisals, EI was associated with higher intensity o pleasant emotions and perceived performances. As an applied implication, we encourage sport psychologists and coaches to provide training programs aimed at enhancing EI.
454	Creatine kinase and neuromuscular fatigue responses following differing spells of simulated cricket fast bowling	Bray J.W.,Towlson C.,Hayes S.C.,Fogarty M.	Plos One	2025	S3	Scopus	The fast-bowling action demands repetitive high-intensity whole body movements, imposing complex physical and perceptual demands on players that vary significantly throughout the season. This study aimed to assess and establish practical methods and metrics for quantifying fatigue after four simulated fast bowling spells. Eleven senior club male fast bowlers (age 27.3 ± 7.0 y; body mass 83.7 ± 11.6 kg; height 1.80 ± 0.06 m) completed four differen bouts of the modified Cricket Australia-Australian Institute of Sport bowling skills test over consecutive weeks. Neuromuscular function (countermovemen jump [CMJ]) and creatine kinase (CK) levels were assessed at baseline, immediately post- (+0 h), and +24 h post-simulation. Perceptual measures (session rating of perceived exertion [sRPE]) and well-being were recorded pre and post fast-bowling simulations, with physical demands (PlayerLoad™) recorded throughout each simulation. Significant reductions in CMJ height were observed at +0 hours ($t = 9.789$, $P < 0.01$, $d = 0.50$) and +24 hours post-simulation ($t = 4.051$, $P < 0.01$, $d = 0.21$) compared to baseline. Moderate correlations were found between deliveries bowled ($r = 0.48$, $P < 0.01$), simulation duration ($r = 0.49$ $P < 0.01$), PlayerLoad™ ($r = 0.41$, $P < 0.01$), sRPE ($r = 0.48$, $P < 0.01$), and the change in CK concentration at +24 hours post-simulation. These findings suggest that lower-body neuromuscular function may be compromised following spells of fast bowing for up to 24 hours. Moreover, a 'dose-response' relationship was observed between the change in CK concentrations and PlayerLoad™, sRPE and number of deliveries bowled at +24 hours post-bowling spell. Coaches and support staff could use a combination of tools to monitor training and playing to enhance their ability to make informed decisions about a player's readiness to perform.
455	Effects of brief mindfulness intervention on mental fatigue and recovery in basketball tactical performance	Cao S.,Liu J.,Geok S.K.,Sun H.,Wang X.	Plos One	2024	S3	Scopus	Objective The detrimental effects of mental fatigue (MF) have been established in sports, such as soccer, volleyball, and basketball. Mindfulness interventions are considered a promising method to help players counteract MF, but whether it could improve basketball tactical performance after MF in competition is not clear. This study aims to investigate the effect of brief mindfulness intervention on basketball tactical performance under MF. Method This study employed a cluster randomized controlled trial (cRCT) design. It involved 54 male basketball players aged 18 to 24 from three universities. The participants were randomly assigned to one of three groups: control group (CG), mental fatigue group (MFG), and mental fatigue-mindfulness group (MF- MG). Players in the MFG and MF-MG underwent a 30-minute Stroop task to induce MF. Subsequently, players in the MF-MG engaged in a 30-minute audio mindfulness intervention. Basketball tactical performance was assessed in the small side games (SSG). Results There were no significant differences in total tactics observed across groups and over time. However, when examining specific tactical sub-variables, significant differences were found in ball reversal, dribble penetration into the key area, and off-ball screen between the CG and MFG in the post-test. Furthermore, significant differences were noted in ball reversal, dribble penetration into the key area, on-ball screen, and off-ball screen between the MFG and MF-MG in the post-test. Conclusion The basketball tactical performances, particularly in areas such as ball reversal, dribble penetration into the key area, on-ball screen, and off-ball screen, were negatively impacted by MF. Notably, the brief mindfulness intervention effectively restored these performance aspects. This suggests that coaches and trainers should place increased emphasis on players' mental well-being and consider incorporating brief mindfulness interventions into their training programs. More studies that investigate mindfulness intervention on the comprehensive aspects of basketball performance should be focused on in the future.

456	Impact of offensive team variables on goal scoring in the first division of the spanish soccer league: a comprehensive 10-year study	Prieto-Gonzalez P.,Martin V.,Pacholek M.,Sal-de-Rellan A.,Marcelino R.	Scientific Reports	2024	S3	Scopus	This study assessed the impact of team offensive variables on goals scored in Spain's first-division soccer league from 2012–2013 to 2021–2022. A nomothetic multidimensional study was conducted, following the STROBE checklist. From 115 team variables available in the INSTAT database, 57 offensive metrics were selected. The selection was performed independently by five researchers to ensure comprehensive inclusion of relevant metrics. Th data included 38 league matches each season, involving 30 teams. The study identified key offensive performance metrics strongly correlated with goals scored. These include shots on target (r = 0.898), chances created (r = 0.871), penalty box entries (r = 0.852), positional attacks with shots (r = 0.818), total shots (r = 0.807), central attacks with shots (r = 0.804), accurate passes (r = 0.760), and efficiency in positional attacks (r = 0.755). The findings underscore th importance of specific offensive metrics in enhancing goal-scoring capabilities. Coaches can improve their team's offensive performance by focusing on passing accuracy, dribbling, and maximizing scoring chances. Successful teams consistently convert attacks into shots on goal through various situations including central attacks, flanks, counterattacks, corners, and set pieces. Tactical adjustments based on these insights can optimize offensive effectiveness.
457	Bystander Intervention in Football and Sports. A Quasi-Experimental Feasibility Study of a Bystander Violence Prevention Program in the United Kingdom	Kovalenko A.G.,Fenton R.A.	Journal of Interpersonal Violence	2024	S3	Scopus	In recent years, social campaigns and high-profile cases have brought increased attention to violence against women. Athletes can be role models, shaping both prosocial and antisocial attitudes. Their engagement in violence prevention could be an effective tool to tackle violence against women through bystander intervention. This part of a mixed-method feasibility study reports on the quantitative evaluation of an evidence-led bystander program, Football Onside, implemented at a football club in South West England in June 2018 to February 2020. The study employed a quasi-experimenta design with intervention and control groups. Football coaches and club members (n = 50) completed measures of rape and domestic abuse myths, bystander intent and efficacy, self-reported bystander behaviors, readiness for change, perceptions of peer helping and myth acceptance, law knowledge, and program evaluation. Fidelity was also assessed. The analysis compared between- and within-group differences in mean changes over time using mixed-effects models. Participant ratings of learning outcomes were high, and fidelity was maintained throughout the intervention. Between-group comparison revealed mixed results, with greater improvements in the intervention group for bystander intent and efficacy at post-test and follow-up, domestic abuse myths at post-test, and rape myth acceptance at follow-up. Model contrasts for within intervention group revealed improvements in rape and domestic abuse myth acceptance, bystander intent and efficacy, perceived law knowledge at both time points, and perceived peer myths and helping at post-test. At follow-up, intervention participants reported significantly higher engagement in bystander behaviors. No significant effects were found fo perceived importance of legal knowledge. Our research highlights the potential efficacy of a bystander program tailored for football club members. Cluster-randomized control trials are now required to examine bystander attitudes and behavior change processes among professional athletes.
458	Eye-Tracking Analyses of a Coach's Pointing Gestures Timed With Speech: Implications for Players' Recall of Basketball Tactical Instructions	Ben Chikha H.,Mguidich H.,Zoudji B.,Khacharem A.	Perceptual and Motor Skills	2024	S3	Scopus	Coaches often use pointing gestures alongside their speech to reinforce their message and emphasize important concepts during instructional communications, but the impact of simultaneous pointing gestures and speech on learners' recall remains unclear. We used eye-tracking and recalled performance to investigate the impact of a coach's variously timed pointing gestures and speech on two groups of learners' (novices and experts) visual attention and recall of tactical instructions. Participants were 96 basketball players (48 novice and 48 expert) who attempted to recall instructions about the evolution of a basketball game system under two teaching conditions: speech accompanied by gestures and speech followed by gestures. Overall, the results showed that novice players benefited more from instructional speech accompanied by gestures than from speech followed by gestures alone. This was evidenced by their greater visual attention to the diagrams, demonstrated through a higher fixation count and decreased saccadic shifts between the coach and the diagrams. Additionally, they exhibited improved recall and experienced reduced mental effort, despite having the same fixation time on the diagrams and equivalent recall time. Conversely, experts benefited more from instructional speech followed by gestures, indicating an expertise reversal effect. These results suggest that coaches and educators may improve their tactical instructions by timing the pairing of their hand gestures and speech in relation to the learner's level of expertise.

459	Match analysis and probability of winning a point in elite men's singles tennis	Prieto-Lage I.,Parames-Gonzalez A.,Torres-Santos D.,... Reguera-Lopez-de-la-Osa X.,GutierrezSantiago A.	Plos One	2023	S3	Scopus	Notational analysis and new technologies have allowed a better understanding of tactical actions in tennis. In particular, the combined analysis of different variables affecting performance is necessary to understand the relationships between actions in competition. The aim of this research was to analyse the probability of winning a point in men's professional tennis based on the most relevant variables affecting performance in this sport. A total of 4,669 points were analysed on three different court surfaces from the final rounds (from the quarter-finals onwards) of three of the four Grand Slam tournaments in the 2021 season. An observational methodology was applied. Different analysis techniques were used to obtain the results: descriptive and chi-square with a significance level of p<0.05. First serve effectiveness (point won) was 69% on clay, 75% on grass and 75% on hard court. Second serve effectiveness (point won) was around 55% regardless of the surface. The majority of points, between 65% and 77% depending on the court surface, ended with a short rally (between one and four shots). Approximately 80% of the points played with first serve and short rally were won by the serving player. With first serve and medium length rallies, the probability of winning the point is similar between server (range 49–55%) and receiver on any court surface The study reveals a set of patterns (based on the combination of information from the variables analysed) that determine the probability of winning a point. Descriptive data from this research could help coaches and players on match strategy at the highest levels of elite men's single tennis.
460	Elite lean athletes at sports high schools face multiple risks for mental health concerns and are in need of psychosocial support	Lundqvist C.,Schary D.P.,Eklof E.,Zand S.,Jacobsson J.	Plos One	2023	S3	Scopus	The combined demands on an adolescence in an elite sports high school can negatively affect mental health (eg, stress, burnout, depression, anxiety). Lat adolescence is also when elite-striving athletes typically transition from junior-to-senior level programs. In addition, adolescent elite lean sports athletes have an increased risk of suffering from abnormal eating pathology. The purpose of this study was to investigate the perceived psychosocial needs that young, elite-striving lean sports athletes in sports high schools find essential for their sports careers and transition from junior-to-senior level sports. Eigh Swedish elite-striving lean sports athletes (2 men and 6 women; median age 17.0 years, range: 16-18 years) participated in this study and sports represented were athletics (n = 1), gymnastics (n = 3), and wrestling (n = 4). Semi-structured interviews were performed, and data was analyzed by themati analysis. Results showed integration, both in the present situation and for the future, as an overarching theme perceived as essential for a successful elite sports career over time. Three additional themes were also identified: (a) Psychosocial stress (combined performance demands, diminished social life, sports-related body weight demands, taboo talking about eating disorders, injuries), (b) Protective psychosocial factors (social support and psychological safety, communication and coordination school and sports, self-care/health behaviors), and (c) support needs junior-to-senior transition (career advice an mentorship, individualized support). As a result, elite-striving, lean sports athletes attending sports high schools need additional support to excel in their academic and athletic endeavors. Sports developmental programs continue to provide inadequate support before and during the junior-to-senior level transition. Similarly, despite the continued calls for reducing the stigma of mental health, athletes in sports schools still encounter barriers from coaches and peers, making the subject taboo, particularly surrounding eating pathologies.
461	Amplifying perceptual demands: How changes in the colour vests affect youth players performance during medium-sided games	Coutinho D.,Goncalves B.,Folgado H.,... Santos S.,Sampaio J.	Plos One	2022	S3	Scopus	This study explored how manipulating the colour of training vests affects footballers' individual and collective performance during a Gk+6vs6+Gk medium sided game. A total of 21 under-17 years old players were involved in three experimental conditions in a random order for a total of four days: i) CONTROL, two teams using two different colour vests; ii) SAME, both teams wearing blue vests; iii) MIXED, all 6 players per team wore different colour vests. Players' positional data was used to compute time-motion and tactical-related variables, while video analysis was used to collect technical variables. Further, these variables were synchronized with spatiotemporal data allowing to capture ball-related actions in a horizontal 2D plan. All variables were analysed from the offensive and defensive perspective. From the offensive perspective, players performed more and further shots to goal during the CONTROL than in SAME and MIXED (small effects) conditions, with a decreased distance to the nearest defender (small effects). While defending, results revealed lower distance to the nearest teammate (small effects) in the CONTROL than in the SAME and MIXED conditions, and higher team longitudinal synchronization (small effects). In addition, the CONTROL showed in general lower values of team width while defending than in the other 2 conditions. Overall, coaches may use the CONTROL condition to emphasize offensive performance and defensive behaviour over the longitudinal direction with increased physical demands. In turn, coaches may use the manipulation of players vests to emphasize defensive performance, as players seem to behave more cohesively under such scenarios.

462	Redesigning a Youth Soccer Program: Holistic Development of Athletes	Moore M.,Blom L.,Califano K.,... Gabler T.,Sullivan M.	Child and Adolescent Social Work Journal	2021	S3	Scopus	Research connects organized sport participation with greater psychological and social benefits in youth athletes. In order to best promote these positive outcomes, youth sport programs must be active in their implementation of effective models that promote positive youth development. This study explored the implementation of the Five C's model in a youth academy soccer program. This longitudinal study explored the perceptions of youth athletes and members of their ecological system (e.g., coaches and parents). Using a mixed method approach, 145 youth athletes, 128 parents, and 23 coaches responded to surveys and/or participated in focus groups. Survey results indicate the implementation of the Five C's led to more opportunities for positive development among youth athletes, an increase in the positive affects youth athletes experienced throughout the program, an increase in parental support of the academy, and a shift in coaching philosophies that promote the Five C's. Together these results indicate change can be made when youth sport organizations implement positive youth development models. Additionally, these models could serve as a foundation for improving a long-lasting commitment to youth sport.
463	In-season training responses and perceived wellbeing and recovery status in professional soccer players	Mateus N.,Goncalves B.,Felipe J.L.,... Weldon A.,Sampaio J.	Plos One	2021	S3	Scopus	This study aimed to describe professional soccer players' training responses during a competitive season and to investigate the relationship between these responses with wellbeing and recovery indices. Thirteen professional soccer players from the same Spanish Second Division team were monitored during sixteen-week in-season period. Players' external loads were analyzed using global positioning measurement units (GPS). Additionally, subjective reporting of sleep quality, sleep duration, fatigue, muscle soreness, and stress were assessed with a customized wellness questionnaire at the beginning of each training session. A two-step cluster analysis identified profiles of different training responses generally described as lower-demand sessions, intermediate demand sessions, running-based sessions, and sprint-based sessions; which were discriminated by different total distance covered and high-intensity actions. Interestingly, no probabilistic interactions were found between these training responses with wellbeing and recovery markers (i.e., Bayes factor < suggesting no evidence, for all the variables). These findings may raise concerns about using self-reporting tools, as they show that players' wellness data is probably not accounted for when coaching staff plan and optimize the training process. However, results should be interpreted with caution, due to representing a single team and coaching staff.
464	Run-up strategies in competitive long jumping: How an ecological dynamics rationale can support coaches to design individualised practice tasks	McCosker C.,Renshaw I.,Polman R.,Greenwood D.,Davids K.	Human Movement Science	2021	S3	Scopus	Understanding how individuals navigate challenging accuracy demands required to register a legal jump is important in furthering knowledge of competitive long jumping. Identification of co-ordination tendencies unique to each individual emphasises the need to examine the presence of unique movement solutions and presents important information for individualisation of training environments. In this study, key measures of gait were recorded during the long jump run-ups of 8 athletes at 8 national level competitions in the 2015 and 2016 Australian track and field seasons. These gait measures were examined to identify whether different visual regulation strategies emerged for legal and foul jumps for each competitor. Emergence of different footfall variability data curves, illustrating how step adjustments were distributed across the run-up for each athlete, suggests that athletes interacted differently with features of the competition environment. This observation highlights the importance of movement adaptability as constraints change and emerge across each performance trial. Results provided further support in conceptualising the run-up as a continuous interceptive action task consisting of a series of interconnected events (i.e., individual step lengths) influencing the regulation of gait towards the take-off board. This information can be use by coaches and practitioners in designing training environments that promote athlete adaptation of more functional movement solutions closely matched to the dynamics of competition environments. Results suggest that training designs that help athletes to search, explore and exploit key sources of information from the competition environment will enhance the fit between the individual and the environment and the development of rich, adaptable movement solutions for competitive performance.
465	Balance dynamics are related to age and levels of expertise. Application in young and adult tennis players	Caballero C.,Barbado D.,Hernandez-Davo H.,Hernandez-Davo J.L.,Moreno F.J.	Plos One	2021	S3	Scopus	In tennis, coaches consider balance fundamental for the acquisition of skilled motor performance. However, the potential relationship between balance and tennis expertise and performance has not been explored yet. Therefore, this study assessed the relationship between balance and tennis performance using linear and non-linear parameters through 1) the comparison of tennis players of different ages and levels of expertise, and 2) analyzing the relationship between balance and tennis serving speed and accuracy. One hundred and six recreational and expert male tennis players took part in the study (age range 10–35 years old). Temporal dynamics of postural control during a balance task on an unstable surface were analyzed through the mean velocity and the detrended fluctuation analysis (DFAV) of center of pressure (COP). Tennis serve performance was quantified by measuring accuracy and speed. Traditional variables measuring balance performance only showed differences according to age but not to sport performance. COP showed a reduction of auto-correlated variability (reflected by DFAV) with age but mainly in expert players. COP dynamics was the only balance parameter discriminating sport expertise and it was related to age. Balance dynamics exhibited by expert tennis players DFAV results support the idea that, along the years, sport experience induces balance adaptations characterized by a higher ability to perform postural adjustments. These results also reinforce the us of non-linear analysis to reveal subtle balance adaptations produced by sport practice. Finally, the lack of correlations suggests that balance, measured with scattering variables, in a non-specific task is not a main determinant of sport performance in tennis serve.

466	Facilitating Positive Youth Development in Elite Youth Hockey: Exploring Coaches' Capabilities, Opportunities, and Motivations	Preston C.,Allan V.,Fraser-Thomas J.	Journal of Applied Sport Psychology	2021	S3	Scopus	Youth sport has been identified as a viable means to facilitate positive youth development (PYD) and nurture fully prepared and contributing adults. However, questions have arisen regarding elite youth sport as a context to effectively foster PYD, and specifically the role of the coach in this process. To explore how coaches facilitate PYD in elite youth sport, the first author immersed himself in the elite youth hockey setting as an assistant coach. Using an ethnographic research design, he observed four AAA minor hockey head coaches of four teams in a large urban center in Ontario over the course of one season. In addition, each coach participated in two semistructured interviews. Framed within the COM-B model, we examined how coaches' capabilities, opportunities, and motivations influenced their behaviors related to facilitating PYD. The coaches discussed and demonstrated several behaviors (e.g., setting high standards, providing leadership opportunities, communicating with the athletes) with the potential to facilitate PYD; however, the sport structure (i.e., professional sports model that focuses on competition and performance from early childhood) often restricted their ability to do so. Further coaches' own motivations to achieve performance success presented a challenge to fostering PYD on some occasions. The results are situated within PYD literature and may evoke practical considerations for elite youth sport coaches aiming to foster PYD more effectively. Lay summary: Four AAA minor hocke coaches were interviewed and observed over the course of a season. Although the coaches were capable and motivated when it came to fostering the personal development of their players, the performance-oriented structure of the minor hockey system meant that coaches often prioritized winning over player development.
467	Athletes' Perception of Leadership According to Their Perceptions of Goal Achievement and Sport Results	Gomes A.R.,Almeida A.,Resende R.	Perceptual and Motor Skills	2020	S3	Scopus	In this study, we investigated whether athletes' perceptions of coaches' leadership differ according to their perceptions of individual and team goal achievement and their sport performance. We collected data at the beginning and end of the sport season from 180 soccer players (aged 16–18 years). We evaluated three leadership areas (transformational, transactional, and decision-making) and the participants' perceptions of individual and team performance during the sport season. Our results showed that (a) athletes with perceptions of higher individual goal achievement evaluated their coaches more positively, (b) athletes with perceptions of higher team goal achievement started the sport season with a less positive evaluation of their coaches but ended the season with a more positive coach evaluation, and (c) athletes with higher sport performance evaluated their coaches less positively in two domains of transformational leadership but ended the season by attributing less negative feedback and passive management to their coaches. In conclusion, these athletes' evaluations of coaches' leadership behaviors differed according to their perceptions of goal achievement and their own sport performance.
468	What does it mean to be "mentally tough" as a NCAA division I collegiate coach?	Weinberg R.,Freysinger V.,Vealey R.,Block C.	Journal of Applied Sport Psychology	2020	S3	Scopus	The purpose of the present investigation was to investigate what being a mentally tough coach means to NCAA Division I collegiate coaches who see themselves as, or becoming, mentally tough coaches. Twelve coaches were recruited who met the criteria concerning years coaching, winning percentage, and years with the same team. Qualitative interviews were conducted with the main question being "what does it mean to be a mentally tough coach in NCAA Division I (DI) collegiate sport today." Results revealed the themes of Emotional Regulation and Endurance, embracing a particular Philosophy/Way of Thinking, and exhibiting certain Behaviors/Actions. These three themes of coach mental toughness were situated in and interacted with the Contexts of DI Collegiate Sport. In addition, these components of coach mental toughness were Developed Over Time and Required Balance across many areas. In relation to the extant coaching literature, these results suggest that study participants felt that coach mental toughness means productive self- management and modeling, and leadership within inherently inequitable and highly stressful and scrutinized contexts. Future directions for research include more ethnographic and case studies for more in-depth responses as well as including how cultural values may influence the conceptualization of mentally tough coach. Lay summary: Results from interviews revealed that common themes for mentally tough NCAA Division I coaches included Emotional Regulation and Endurance, embracing a particular Philosophy/Way of Thinking, and exhibiting certain Behaviors/Actions. These components o coach mental toughness were Developed Over Time and Required Balance between being coaches, being educators and focusing on athlete development.Implications for Practice NCAA Division I coaches have to pursue high performance and winning to keep their jobs but also need to balance this with being educators and focusing on athlete development and well-being. Emotional regulation and self-control are key aspects of mental toughnes in NCAA Division I coaches, and thus coaches need to develop these psychological skills to complement their other coaching skills (e.g., strategy, instruction). Having a consistent philosophy or way of thinking serves as a foundation for being a mentally tough NCAA Division I coach, including consistency in expectations, setting high standards, being committed to student-athlete development, adopting a growth mindset of professional (coach) development, and being genuine and

469	Young Dutch commercially sponsored kite surfers: free as a bird?	Smits F.	Sport in Society	2019	S3	Scopus	While coaches and organizational structures play a pivotal role in the disciplining of young athletes in traditional mainstream organized sports, young kit surfers seem to be free to develop their own skills and create their own identity and subcultures. However, the feeling of freedom of young commercially sponsored kite surfers may be more complex than the popular discourses within action sport cultures suggest. I use a Foucauldian framework to explore how disciplinary power, embedded in time and space, produces athletic skill and identity in adolescent kite surfers and informs their practices of freedom In this paper, I show how young kite surfers negotiate constraints of space and time that are shaped by relationships with their parents, their kite surfing peers and their commercial sponsors. Semi-structured interviews were conducted with eleven commercially sponsored male kite surfers, between 11 and 17 years old. In so doing, I found that although the discourse of freedom associated with kite surfing acts on their experiences, these young athletes were also subjected to disciplinary power produced by temporal and spatial limitations and capabilities, their subculture, digital media and commercialization
470	The Optimal Team Functioning Model: A Grounded Theory Framework to Guide Teamwork in Curling	Collins J.,Durand-Bush N.	Journal of Applied Sport Psychology	2019	S3	Scopus	A constructivist grounded theory approach was used to investigate teamwork and team effectiveness in the sport of curling. Focus group and individual interviews were conducted with 78 athletes and 10 coaches from 19 high-performance curling teams. Data analysis led to the creation of the Optimal Team Functioning (OTF) model, which comprises 8 key components for optimal team functioning: (a) individual attributes, (b) team attributes, (c) foundational process of communication, (d) structural team processes, (e) individual regulation processes, (f) team regulation processes, (g) context, and (h) desired outcomes. Components and relationships within the model, along with practical implications, are discussed. Lay Summary: Developing effective sport teams is a challenging and complex process. Unfortunately, few resources are available to guide coaches and athletes in their efforts to optimally function and perform as a unit on a daily basis. This novel team dynamics research was carried out with high-performance coaches and athletes to uncover a comprehensive and ecologically valid model mapping out effective teamwork within the sport of curling. The Optimal Team Functioning Model focuses on concrete actions that can be applied to successfully set up and manage a team and its individual members throughout a season.
471	Extracting spatial-temporal features that describe a team match demands when considering the effects of the quality of opposition in elite football	Goncalves B.,Coutinho D.,Exel J.,... Lago C.,Sampaio J.	Plos One	2019	S3	Scopus	Spatiotemporal patterns of play can be extracted from competitive environments to design representative training tasks and underlying processes that sustain performance outcomes. To support this statement, the aims of this study were: (i) describe the collective behavioural patterns that relies upon the use of player positioning in interaction with teammates, opponents and ball positioning; (ii) and define the underlying structure among the variables through application of a factorial analysis. The sample comprised a total of 1,413 ball possession sequences, obtained from twelve elite football matches from one team (the team ended the season in the top-5 position). The dynamic position of the players (from both competing teams), as well as the ball, were captured and transformed to two-dimensional coordinates. Data included the ball possession sequences from six matches played against top opponents (TOP, the three teams classified in the first 3 places at the end of the season) and six matches against bottom opponents (BOTTOM, the three teams classified in the last 3 at the end of the season). The variables calculated for each ball possession were the following: ball position; team space in possession; game space (comprising the outfield players of both teams); position and space at the end of ball possession. Statistical comparisons were carried with magnitude-based decisions and null-hypothesis analysis and factor analysis to define the underlying structure among variables according to the considered contexts. Results showed that playing against TOP opponents, there was ~38 meters game length per ~43 meters game width with 12% of coefficient of variation (%). Ball possessions lasted for ~28 seconds and tended to end at ~83m of pitch length. Against BOTTOM opponents, a decrease in the game length with an increase in game width and in the deepest location was observed in comparison with playing against TOP opponents. The duration of ball possession increased considerable (~37 seconds), and the ball speed entropy was higher, suggesting lower levels of regularity in comparison with TOP opponents. The BOTTOM teams revealed a small EPS. The Principal Component Analysis showed a strong association of the ball speed, entropy of the ball speed and the coefficient of variation (%) of the ball speed. The EPS of the team in possession was well correlated with the gam space, especially the game width facing TOP opponents. Against BOTTOM opponents, there was a strong association of ball possession duration, game width,

472	Overcoming the problem of multicollinearity in sports performance data: A novel application of partial least squares correlation analysis	Weaving D.,Jones B.,Ireton M.,... Till K.,Beggs C.B.	Plos One	2019	S3	Scopus	Objectives Professional sporting organisations invest considerable resources collecting and analysing data in order to better understand the factors that influence performance. Recent advances in non-invasive technologies, such as global positioning systems (GPS), mean that large volumes of data are now readily available to coaches and sport scientists. However analysing such data can be challenging, particularly when sample sizes are small and data sets contain multiple highly correlated variables, as is often the case in a sporting context. Multicollinearity in particular, if not treated appropriately, can be problematic and might lead to erroneous conclusions. In this paper we present a novel 'leave one variable out' (LOVO) partial least squares correlation analysis (PLSCA) methodology, designed to overcome the problem of multicollinearity, and show how this can be used to identify the training load (TL) variables that influence most 'end fitness' in young rugby league players. Methods The accumulated TL of sixteen male professional youth rugby league players (17.7 ± 0.9 years) was quantified via GPS, a micro-electrical-mechanical-system (MEMS), and players' session-rating-of-perceived-exertion (sRPE) over a 6-week pre-season training period. Immediately prior to and following this training period, participants undertook a 30–15 intermittent fitness test (30-15IFT), which was used to determine a players 'starting fitness' and 'end fitness'. In total twelve TL variables were collected, and these along with 'starting fitness' as a covariate were regressed against 'end fitness'. However, considerable multicollinearity in the data (VIF >1000 for nine variables) meant that the multiple linear regression (MLR) process was unstable and so we developed a novel LOVO PLSCA adaptation to quantify the relative importance of the predictor variables and thus minimise multicollinearity issues. As such, the LOVO PLSCA was used as a tool to inform and refine the MLR process. Results The LOVO PLSCA identified the distance accumulated at very-high speed (>7 ms-1) as being the most important TL variable to influence improvement in player fitness, with this variable causing the largest decrease in singular value inertia (5.93). When included in a refined linear regression model, this variable, along with 'starting fitness' as a covariate, explained 73% of the variance in v30-15IFT 'end fitness' (p<0.001) and eliminated complete any multicollinearity issues. Conclusions The LOVO PLSCA technique appears to be a useful tool for evaluating the relative importance of predictor variables in data sets
473	Relationships between technique and bat speed, post-impact ball speed, and carry distance during a range hitting task in cricket	Peploe C.,McErlain-Naylor S.A.,Harland A.R.,King M.A.	Human Movement Science	2019	S3	Scopus	The ability of a batsman to clear the boundary is a major contributor to success in modern cricket. The aim of this study was to identify technique parameters characterising those batsmen able to generate greater bat speeds, ball launch speeds, and carry distances during a range hitting task in cricket. Kinematic data were collected for 20 batsmen ranging from international to club standard, and a series of ball launch, bat-ball impact, and technique parameters were calculated for each trial. A stepwise multiple linear regression analysis found impact location on the bat face in the medio- lateral and longitudinal directions and bat speed at impact to explain 68% of the observed variation in instantaneous post-impact ball speed. A further regression analysis found the X-factor (separation between the pelvis and thorax segments in the transverse plane) at the commencement of the downswing, lead elbow extension, and wrist uncocking during the downswing to explain 78% of the observed variation in maximum bat speed during the downswing. These findings indicate that players and coaches should focus on generating central impacts with the highest possible bat speed. Training an conditioning programmes should be developed to improve the important kinematic parameters shown to generate greater bat speeds, particularly focussing on increased pelvis to upper thorax separation in the transverse plane.
474	The Development and Validation of the Adolescent Sport Drug Inventory (ASDI) Among Athletes From Four Continents	Nicholls A.R.,Meir R.,Jones L.,... Baghurst T.,Perry J.L.	Psychological Assessment	2019	S3	Scopus	A significant barrier to understanding the psychosocial antecedents of doping use among adolescent athletes is the lack of valid measures. In order to address this issue, the first aim of this paper was to develop and validate the Adolescent Sport Drug Inventory (ASDI) among adolescent athletes from Asia Europe, North America, and Oceania. The second aim was to assess the construct validity of the ASDI. As such, this paper is divided into two parts. Part 1 relates to the development of the ASDI and contains two studies: item development (Study 1) and factorial validity (Study 2). Part 2 contains information o how the psychosocial variables measured in the ASDI are associated with situational temptation, and honesty (Study 3), maturation (Study 4), stress and coping (Study 5), and coaching (Study 6). In devising the ASDI, 19 different models were examined, which culminated in a 9-factor, 43-item ASDI. Coping, mastery-approach goals, and cognitive–social maturity were associated with doping attitudes. Caring motivational climates, strong coach–athlete relationships, and positive coach behaviors were associated with athletes being less susceptible toward doping, which provides construct validity for the ASDI. The ASDI is a valid tool to assess the psychosocial factors associated with doping among adolescent athletes. This questionnaire can be used to identify athletes who are the most at risk of doping, assess how the psychosocial factors associated with doping change over time, and to monitor the impact of antidoping interventions for adolescent athletes.

475	Do coaching style and game circumstances predict athletes' perceived justice of their coach? A longitudinal study in elite handball and volleyball teams	De Backer M.,Reynders B.,Boen F.,Van Puyenbroeck S.,Broek G.V.	Plos One	2018	S3	Scopus	Objective The present longitudinal study is the first to examine game to game fluctuations of perceived justice of elite volleyball and handball coaches. More specifically, we studied whether coaching style (i.e., need support versus control), coach behaviors (decision justifications), player's status (i.e., starter or substitute), and game result (win/loss) predicted athletes' perceived justice and its fluctuations. Methods A longitudinal questionnaire study was performed during 6 consecutive weeks among Belgian female volleyball (N = 57) and male handball players (N = 39). We administered a general questionnaire (i.e., need support/control) the first week, and game-specific questionnaires (i.e., justice, decision justifications, game circumstances) after six consecutive games. Because game-to-game measures (i.e., within-athlete) were nested into individuals (between-athletes) we conducted Hierarchical Linear Modeling to examine the hypotheses. Results Multilevel analyses showed that 49% of the variance of perceived justice was situated at the within- athlete level. Furthermore, coaches' need support and the provision of decision justifications were positive predictors of athletes' perceived justice of the coach. More specific, the impact of justifications was less strong in a high need supportive environment and stronger in a high controlling environment. Finally, both the status of the player and the game result were negative predictors of athletes' perceived justice. Conclusions We can conclude that athletes' perceived justice of their coach shifts considerably from game-to-game. Furthermore, the coaching style and coaching behaviors can help to overcome the negative effects of specific game circumstances such as being a substitute or losing a game on athletes' perceived justice of the coach.
476	Physiological, perceptual, and technical responses to continuous and intermittent small-sided games in lacrosse players	Hauer R.,Tessitore A.,Binder N.,Tschan H.	Plos One	2018	S3	Scopus	Purpose The present study was designed to investigate the influence of two distinct small-sided game (SSG) regimes on physiological, perceptual, and technical parameters in male elite lacrosse players. Method Data were collected in twelve elite male Austrian lacrosse players (25.8 ± 5.5 years; 80.1 ± 7.7 kg; 178.5 ± 6.2 cm). Players' were assigned to an intermittent (SSG-I) or a continuous (SSG-C) SSG regime, respectively. Regimes were equated for total practice time, but not active playing time. SSG data from eight sessions of 3 vs. 3 self-regulated match-play were collected along a 4-week pre-season period. Players' YoYo-Level 1 (YYL1) performance before and after the training intervention was recorded. Further, heart-rate (HR), rating of perceived exertion (RPE), physical activity enjoyment scale (PACES), and technical actions during and after SSG sessions were analyzed. Results Both SSG regimes showed improvement with medium to very large effect sizes (ES) in YYL1 total distance covered pre- to post-intervention (SSG-C mean-difference ± SD: 840 ± 299 m; p = 0.003; d = 1.08; CI = 0.60 to 1.56 and SSG-I: 607 ± 274 m; p = 0.003; d = 1.25; CI = 0.66 to 1.85 respectively). Higher %HR max values with very larg ES (92 ± 0.6%; p = 0.002; d = 5.33; CI = 2.78 to 7.88) and time spent in HR zone 4 (1248.0 ± 122.7 s; p = 0.000; d = 3.43; CI = 2.31 to 4.55) were observed for SSG-C. No differences between regimes were found for any of the assessed technical actions, global RPE, and PACES scores. Conclusions Both SSG regimes investigated in this study were effective in improving YYL1 performance. Further, findings indicate that the regime does not influence players' subjective feelings and technical actions in SSG play. However, SSG-I in lacrosse specific training could have additional benefits such as lower signs of fatigue. Further, breaks can be used to give technical and tactical inputs by coaches.
477	Relationships between physical qualities and key performance indicators during matchplay in senior international rugby union players	Cunningham D.J.,Shearer D.A.,Drawer S.,... Russell M.,Kilduff L.P.	Plos One	2018	S3	Scopus	The use of physical tests to profile physical capabilities, and provide training direction to athletes is common practice. Likewise, in professional team sports, notational analysis codes the key contributions of each player during competition. Limited studies have however investigated relationships betwee physical capabilities and key performance indicators (KPIs) of rugby union match-play. Elite professional players, categorised as forwards (n = 15) or backs (n = 14), from an international rugby union squad (n = 29) undertook assessments of isometric mid-thigh pull (IMTP), bilateral and unilateral countermovement jumps (CMJ) and drop jumps (DJ; from 40 and 20 cm, respectively), and assessment of acceleration (10 m), a 5 m weighted sled drive, and a Yo-Yo intermittent recovery test level 1 (Yo-Yo IRTL1). Game statistics of the same players from 92 matches (~23 matches per player) during the 2014– 15 season were analysed for effort and performance-based metrics. For forwards, Yo-Yo IRTL1 correlated significantly with; number of tackles made (r = 0.717), first three players at a ruck in both attack (r = 0.568) and defence (r = 0.581), number of effective rucks (r = 0.630), total possessions (r = 0.522), passes made (r = 0.651), percentage of carries over the gainline (r = 0.610), effective ruck success (r = 0.600), tackle success (r = 0.540), and the number of turnovers made (r = 0.518). Drop jump performance in forwards was associated with; the number of clean breaks (r = 0.558), dominant collisions (r = 0.589), and offloads (r = 0.594). For backs, the sled-drive test correlated with; number of carries (r = -0.751), first three players at an attacking ruck (r = -0.613), effective attacking rucks (r = -0.584), number of dominant collisions (r = -0.792) and offloads (r = -0.814). Likewise, for backs, IMTP peak force was related to; the number of possessions (r = 0.793), passes made (r = 0.792), effective attacking ruck percentage (r = 0.628), and the number of offloads (r = 0.621) whilst relative peak force correlated with; the percentage of carries over the gainline (r = 0.533), percent tackle success (r = 0.603) and effective attacking ruck percentage (r = 0.584). Regression analyses highlighted that only a small number of variables (i.e., carries, tackles, attacking and defensive first three at ruck) returned practically achievable changes (<20%) in physical qualities. In spite of this, and while leaving scope identification of further physical and/o performance predictors, greater strength, power and intermittent running performance were positively related to match-

478	Positive youth development and observed athlete behavior in recreational sport	Vierimaa M.,Bruner M.W.,Cote J.	Plos One	2018	S3	Scopus	Objectives Competence, confidence, connection, and character are regarded as outcomes of positive youth development (PYD) in sport. However, the specific athlete behaviors associated with different PYD profiles are not well understood. Thus, the purpose of this study was to investigate the relationshi between athletes' observed behavior during sport competitions and their perceptions of PYD outcomes. Design Cross-sectional study with systematic behavioral observation. Method Sixty-seven youth athletes were observed during basketball games near the end of their season, and the content of their behavior was systematically coded. Athletes also completed measures of the 4 Cs (competence, confidence connection, and character). A person-centered analysis approach was used to examine the relationship between PYD profiles and observed behavior. Results A cluster analysis identified two homogenous groups of athletes characterized by relatively high and low perceptions of confidence, connection, and character. A MANCOVA revealed that after controlling for gender and years of playing experience, the high Cs group engaged in more frequent sport communication with their coaches. Conclusions Results re-affirm the critical role that coaches play in the developmental experiences of young athletes, and highlight the importance of contextual factors of the youth sport environment.
479	Concussion Knowledge and Communication Behaviors of Collegiate Wrestling Coaches	Kroshus E.,Kerr Z.Y.,DeFreese J.D.,Parsons J.T.	Health Communication	2017	S3	Scopus	Sport coaches can play an important role in shaping a team's approach to concussion safety through their communication with team members. However across all sports, there is limited knowledge about factors that make coaches more or less likely to engage in safety-supportive communication. The objectives of this study were to assess the concussion-related knowledge and attitudes of wrestling coaches, as well as the extent to which they engage in autonomy-supportive coaching practices, and to determine how these factors are related to communication with athletes in support of concussion safety. Data were collected through an online survey of head coaches of National Collegiate Athletic Association (NCAA) wrestling teams (n = 89, 40.5% response rate). On average, coaches answered five out of a possible nine knowledge questions correctly and were significantly more likely to think it was acceptable for an athlete to continue playing after sustaining a concussion during a national qualifying competition as compared to during an early-season competition. Engaging in autonomy-supportive coaching behaviors was the coach factor explaining the largest percentage of variability in communication. Findings suggest that while knowledge deficits and attitudes about the acceptability of continued play while symptomatic during more consequential competitive matches should be addressed in educational programming for collegiate wrestling coaches, these changes alone may not be a sufficient for adequately increasing concussion safety communication. Targeting more distal factors such as autonomy-supportive approaches to coachin may hold promise for intervention design and should be explored in future prospective research.
480	A longitudinal examination of stressors, appraisals, and coping in youth swimming	Hayward F.P.I.,Knight C.J.,Mellalieu S.D.	Psychology of Sport and Exercise	2017	S3	Scopus	Purpose Involvement in sport has the potential to cause athletes, coaches, and parents to experience stress. However, the extent to which experiences of stress are shared within the athletic triad is unknown. The purpose of this study was to examine the individual and shared stress experiences among youth swimmers, their mothers, and coach within the context of training, tapering, and competition. Design Multi-case study design. Method Four female swimmers, their mother, and one coach completed daily diaries for six weeks and up to three semi-structured interviews. Data were analyzed through within- and cross-case thematic analysis. Results The study showed evidence of shared stress experiences between all three members of the athletic triad Participants predominately encountered organizational stressors, which they appraised in relation to movements between squad, interpersonal relationships, and overall progress towards performance goals/outcomes. Numerous coping strategies were employed by participants, with varying degrees of effectiveness, such as seeking social support, distancing, and lift sharing. The coping strategies used by coaches, swimmers, and parents were often interrelated with participants frequently seeking emotional support from one another. The majority of stressors and appraisals cited by parents and swimmers were shared, with both heavily relying on social support to help each other cope with the stressors encountered. Conclusion Athletes, parents, and coaches have the capacity to influence one another's stress experiences and as such their experiences should be considered simultaneously to maximize the impact of interventions.
481	Patterns of nutrition and dietary supplements use in young Egyptian athletes: A community-based cross-sectional survey	Tawfik S.,El Koofy N.,Moawad E.M.I.	Plos One	2016	S3	Scopus	The aim of this study was to investigate the pattern of basic and sport nutrition as well as perspectives of young Egyptian athletes. Structured interview survey measuring knowledge, attitudes, beliefs and behaviors about basic and sport nutrition was administered to adolescent athletes recruited from 4 sport clubs and 2 fitness centers in Greater Cairo governorate. A total of 358 participants aged 13-18 years completed questionnaires. Basic nutrition knowledge was reasonable in almost all domains except fast food. Fixed breakfast (78.5%), home meals (lunch, 70.7%), and healthy snacks (55.8%) were the most positive features of the basic dietary pattern. More than 70% perceived themselves as knowledgeable about sport nutrition. The prevalence rate of sport supplement intake was (48.9%, n = 175), predominantly sport drinks (66.9%) and creatine (54.3%). Coaches were the primary source of sport nutrition information. Forty-four percent of participants (n = 77/175) reported supplement consumption during competition seasons only. Better physical appearance and enhancement of athletic performance were the major motivations for supplement intake. These findings indicate the necessity of a comprehensive nutrition education program targeting not only athletes and parents, but also coaching staff, health trainers and all sport team officials.

Match category	Scopus search	Scopus record	Scopus title	Matched master ID	Matched master title	Match method	Decision
Internal Scopus duplicate	S3	86	Elite Athletes' Experience of Coping With Emotional Abuse in the Coach– Athlete Relationship		Elite Athletes' Experience of Coping With Emotional Abuse in the Coach– Athlete Relationship	Exact normalized title	Merged
Internal Scopus duplicate	S3	13	Interpersonal climate and moral conduct in competitive sport: A dual-route model with serial mediation		Interpersonal climate and moral conduct in competitive sport: A dual-route model with serial mediation	Exact normalized title	Merged
Internal Scopus duplicate	S3	63	Olympic and Paralympic coach perspectives on effective skill acquisition support and coach development		Olympic and Paralympic coach perspectives on effective skill acquisition support and coach development	Exact normalized title	Merged
Internal Scopus duplicate	S3	44	Quality of coach-athlete relationship and coping as associated factors of stress, anxiety, burnout, and depression symptoms of soccer players in transition to professional: a prospective study		Quality of coach-athlete relationship and coping as associated factors of stress, anxiety, burnout, and depression symptoms of soccer players in transition to professional: a prospective study	Exact normalized title	Merged
Internal Scopus duplicate	S3	19	Using a Bayesian network to classify time to return to sport based on football injury epidemiological data		Using a Bayesian network to classify time to return to sport based on football injury epidemiological data	Exact normalized title	Merged
Cross-database overlap	S1	1	Effects of basic psychological needs on Chinese youth athlete burnout under coach burnout: Using hierarchical linear modeling	364	Effects of basic psychological needs on Chinese youth athlete burnout under coach burnout: Using hierarchical linear modeling	Exact normalized title	Merged
Cross-database overlap	S1	3	Beyond the podium: A Delphi approach aimed at empowering coaches with practical strategies to identify and support athlete mental health	119	Beyond the podium: A Delphi approach aimed at empowering coaches with practical strategies to identify and support athlete mental health	Exact normalized title	Merged
Cross-database overlap	S1	6	The coach as an architect of Danish high- performance sport environments	145	The coach as an architect of Danish high- performance sport environments	Exact normalized title	Merged
Cross-database overlap	S1	7	The Influence of Parent-Initiated Motivational Climate on Adolescent Golfers' Flow Experiences: The Mediating Role of Basic Psychological Needs	372	The Influence of Parent-Initiated Motivational Climate on Adolescent Golfers' Flow Experiences: The Mediating Role of Basic Psychological Needs	Exact normalized title	Merged
Cross-database overlap	S1	9	Understanding the motivators and barriers of elite athletes as role models	17	Understanding the motivators and barriers of elite athletes as role models	Exact normalized title	Merged
Cross-database overlap	S1	10	Care as a strategy to navigate political tensions within the China–Norway sports coaching collaboration	190	Care as a strategy to navigate political tensions within the China-Norway sports coaching collaboration	Manual fuzzy verification (>0.98 sequence similarity)	Merged
Cross-database overlap	S1	11	Resilience profiles of elite athletes and their associations with health-related behaviors, well-being, and performance: A latent profile analysis	29	Resilience profiles of elite athletes and their associations with health-related behaviors, well-being, and performance: A latent profile analysis	Exact normalized title	Merged
Cross-database overlap	S1	12	Developing guidelines for selection-deselection in high performance sport for athletes, coaches, and organisations: A delphi study	28	Developing guidelines for selection-deselection in high performance sport for athletes, coaches, and organisations: A delphi study	Exact normalized title	Merged
Cross-database overlap	S1	13	NCAA coaches' perceptions of the inclusion of transgender athletes: a qualitative analysis	189	NCAA coaches' perceptions of the inclusion of transgender athletes: a qualitative analysis	Exact normalized title	Merged
Cross-database overlap	S1	14	How is coaches' social identity leadership related to mental health in elite athletes? The mediating role of satisfaction with performance	30	How is coaches' social identity leadership related to mental health in elite athletes? The mediating role of satisfaction with performance	Exact normalized title	Merged
Cross-database overlap	S1	15	Effect of boxers' social support on mental fatigue: Chain mediating effects of coach leadership behaviors and psychological resilience	239	Effect of boxers' social support on mental fatigue: Chain mediating effects of coach leadership behaviors and psychological resilience.	Exact normalized title	Merged
Cross-database overlap	S1	16	Interpersonal Behaviors Questionnaire in Sport: Psychometric Analysis With Romanian Professional Athletes	43	Interpersonal Behaviors Questionnaire in Sport: Psychometric Analysis With Romanian Professional Athletes	Exact normalized title	Merged
Cross-database overlap	S1	18	A cluster analysis of high-performance female team players' perceived motivational climate: Implications on perceived motor competence and autonomous behaviour	67	A cluster analysis of high-performance female team players' perceived motivational climate: Implications on perceived motor competence and autonomous behaviour.	Exact normalized title	Merged
Cross-database overlap	S1	20	Perceived motivational climate influences athletes' emotion regulation strategies, emotions, and psychobiosocial experiences	244	Perceived motivational climate influences athletes' emotion regulation strategies, emotions, and psychobiosocial experiences	Exact normalized title	Merged
Cross-database overlap	S1	22	Satisfaction with life in Special Olympic athletes: the role of autonomy support and basic need fulfilment	58	Satisfaction with life in Special Olympic athletes: the role of autonomy support and basic need fulfilment	Exact normalized title	Merged
Cross-database overlap	S1	25	The 5C's Model of Successful Partnerships in Elite Beach Volleyball Dyads	81	The 5C's Model of Successful Partnerships in Elite Beach Volleyball Dyads	Manual fuzzy verification (>0.98 sequence similarity)	Merged
Cross-database overlap	S1+S3	26	Olympic and Paralympic coach perspectives on effective skill acquisition support and coach development	79	Olympic and Paralympic coach perspectives on effective skill acquisition support and coach development	Exact normalized title	Merged
Cross-database overlap	S1	28	Positive youth development and gender differences in high performance sport	78	Positive youth development and gender differences in high performance sport	Exact normalized title	Merged
Cross-database overlap	S1	30	'I was excited to train, so I didn't have problems with the coach': dual career athletes' experiences of (dis)empowering motivational climates	293	'I was excited to train, so I didn't have problems with the coach': dual career athletes' experiences of (dis)empowering motivational climates	Manual fuzzy verification (>0.98 sequence similarity)	Merged
Cross-database overlap	S1+S3	38	Elite Athletes' Experience of Coping With Emotional Abuse in the Coach– Athlete Relationship	111	Elite Athletes' Experience of Coping With Emotional Abuse in the Coach-Athlete Relationship	Exact normalized title	Merged
Cross-database overlap	S1	39	Scarred for the rest of my career? Career-long effects of abusive leadership on professional athlete aggression and task performance	114	Scarred for the Rest of My Career? Career-Long Effects of Abusive Leadership on Professional Athlete Aggression and Task Performance	Exact normalized title	Merged
Cross-database overlap	S2	1	Acute weight cycling and hydration status in elite kickboxers: a repeated- measures field study	13	Acute weight cycling and hydration status in elite kickboxers: a repeated-measures field study	Exact normalized title	Merged
Cross-database overlap	S2	3	Digital twin for Taekwondo athletes: integrating sports nutrition and psychological readiness using artificial intelligence	122	Digital twin for Taekwondo athletes: integrating sports nutrition and psychological readiness using artificial intelligence	Exact normalized title	Merged

Cross-database overlap	S2	5	Differences in the relationship among grit, self-regulation, competition preparation, and sport confidence across performance levels: a multi-group analysis	154	Differences in the relationship among grit, self-regulation, competition preparation, and sport confidence across performance levels: a multi-group analysis	Exact normalized title	Merged
Cross-database overlap	S2	6	Comparative effects of French Contrast Method vs. Complex Training on explosive power and its endurance in youth badminton athletes	373	Comparative effects of French Contrast Method vs. Complex Training on explosive power and its endurance in youth badminton athletes	Exact normalized title	Merged
Cross-database overlap	S2	7	Relative age effect, morphological status and physical performance of U-15 Croatian football players	157	Relative age effect, morphological status and physical performance of U-15 Croatian football players	Exact normalized title	Merged
Cross-database overlap	S2	8	Factor analysis on the performance of elite male fencing athletes	33	Factor analysis on the performance of elite male fencing athletes	Exact normalized title	Merged
Cross-database overlap	S2+S3	9	Using a Bayesian network to classify time to return to sport based on football injury epidemiological data	156	Using a Bayesian network to classify time to return to sport based on football injury epidemiological data	Exact normalized title	Merged
Cross-database overlap	S2	12	The impact of low energy availability risk on pre-competition physiological function in Chinese female combat athletes	153	The impact of low energy availability risk on pre-competition physiological function in Chinese female combat athletes	Exact normalized title	Merged
Cross-database overlap	S2	13	Weight loss practices in Chinese national and international-level Sanda athletes	155	Weight loss practices in Chinese national and international-level Sanda athletes	Exact normalized title	Merged
Cross-database overlap	S2	14	The epidemiology of injuries in professional sitting volleyball athletes	21	The epidemiology of injuries in professional sitting volleyball athletes	Exact normalized title	Merged
Cross-database overlap	S2	15	Orienteering Is More than Just Running! Acute Effect of Competitive Pressure on Autonomic Cardiac Activity among Elite Orienteering Athletes	34	Orienteering Is More than Just Running! Acute Effect of Competitive Pressure on Autonomic Cardiac Activity among Elite Orienteering Athletes	Exact normalized title	Merged
Cross-database overlap	S2	16	Coping profiles of adolescent football players and association with interpersonal coping: Do emotional competence and psychological need satisfaction matter?	378	Coping profiles of adolescent football players and association with interpersonal coping: Do emotional competence and psychological need satisfaction matter?	Exact normalized title	Merged
Cross-database overlap	S2	17	The effect of post-activation enhancement on the performance of Chinese national skeleton athletes in the “ice push sled”-a first cross-sectional study	193	The effect of post-activation enhancement on the performance of Chinese national skeleton athletes in the ice push sled-a first cross-sectional study	Exact normalized title	Merged
Cross-database overlap	S2	18	Narcissistic coaches and athletes’ individual rowing performance	221	Narcissistic coaches and athletes' individual rowing performance	Exact normalized title	Merged
Cross-database overlap	S2	20	A randomized controlled study of mindful sport performance enhancement and psychological skills training with collegiate track and field athletes	222	A randomized controlled study of mindful sport performance enhancement and psychological skills training with collegiate track and field athletes	Exact normalized title	Merged
Cross-database overlap	S2	21	Stakeholder insights into athlete attrition in the high-performance pathway	59	Stakeholder insights into athlete attrition in the high-performance pathway	Exact normalized title	Merged
Cross-database overlap	S2	22	Rapid weight loss among elite-level judo athletes: methods and nutrition in relation to competition performance	60	Rapid weight loss among elite-level judo athletes: methods and nutrition in relation to competition performance	Exact normalized title	Merged
Cross-database overlap	S2	23	The prevalence of pre-conditioning and recovery strategies in senior elite and non-elite amateur boxing	61	The prevalence of pre-conditioning and recovery strategies in senior elite and non-elite amateur boxing	Exact normalized title	Merged
Cross-database overlap	S2	25	The personal and interpersonal components of perfectionism: The italian validation of “multidimensional inventory of perfectionism in sport”	273	The Personal and Interpersonal Components of Perfectionism: The Italian Validation of Multidimensional Inventory of Perfectionism in Sport	Exact normalized title	Merged
Cross-database overlap	S2	26	Mindfulness training for a college team: Feasibility, acceptability, and effectiveness from within an athletic department	275	Mindfulness training for a college team: Feasibility, acceptability, and effectiveness from within an athletic department	Exact normalized title	Merged
Cross-database overlap	S2	27	Heart rate variability and pre-competitive anxiety according to the demanding level of the match in female soccer athletes	295	Heart rate variability and pre-competitive anxiety according to the demanding level of the match in female soccer athletes	Exact normalized title	Merged
Cross-database overlap	S2	28	The development of a tournament preparation framework for competitive golf: A Delphi study	329	The development of a tournament preparation framework for competitive golf: A Delphi study	Exact normalized title	Merged
Cross-database overlap	S2	29	Emotional intelligence, motivational climate and levels of anxiety in athletes from different categories of sports: Analysis through structural equations	331	Emotional Intelligence, Motivational Climate and Levels of Anxiety in Athletes from Different Categories of Sports: Analysis through Structural Equations	Exact normalized title	Merged
Cross-database overlap	S3	2	Athletic identity and sport injury: a systematic review and meta-aggregation	180	Athletic identity and sport injury: a systematic review and meta-aggregation	Exact normalized title	Merged
Cross-database overlap	S3	4	Development and evaluation of a coach-athlete relationship intervention program	128	Development and evaluation of a coach-athlete relationship intervention program	Exact normalized title	Merged
Cross-database overlap	S3	8	Leveraging Student-Athlete Mental Health Through an AI-Augmented Mobile Platform: The ThriveNudge Study Protocol	365	Leveraging Student-Athlete Mental Health Through an AI-Augmented Mobile Platform: The ThriveNudge Study Protocol	Exact normalized title	Merged
Cross-database overlap	S3	9	Applying the 5Cs Framework to Elite Youth Tennis: Impact Factors in a Talent Development Environment	16	Applying the 5Cs Framework to Elite Youth Tennis: Impact Factors in a Talent Development Environment	Exact normalized title	Merged
Cross-database overlap	S3	10	From social identity to team performance: Linking transactive memory systems and teamwork	135	From social identity to team performance: Linking transactive memory systems and teamwork	Exact normalized title	Merged
Cross-database overlap	S3	11	Structural modelling of student volleyball athletes’ intimacy, social adjustment, perceived stress, and learning-related anxiety: mediating role of psychological safety	170	Structural modelling of student volleyball athletes' intimacy, social adjustment, perceived stress, and learning-related anxiety: mediating role of psychological safety	Exact normalized title	Merged
Cross-database overlap	S3	22	The role of transformational leadership in the associations between coach- athlete relationship and team resilience: A study on elite football players	35	The role of transformational leadership in the associations between coach-athlete relationship and team resilience: A study on elite football players	Exact normalized title	Merged
Cross-database overlap	S3	23	Factors influencing sport persistence still represent a knowledge gap – the experience of a systematic review	198	Factors influencing sport persistence still represent a knowledge gap - the experience of a systematic review	Exact normalized title	Merged
Cross-database overlap	S3	28	A Longitudinal Multicase Study About the Board-Game Format of an Educational Self-Talk Intervention	201	A Longitudinal Multicase Study About the Board-Game Format of an Educational Self-Talk Intervention	Exact normalized title	Merged

Cross-database overlap	S3	29	Initiatives to facilitate the social inclusion of people with intellectual disability in physical activities with others in community-based sporting, recreation and leisure activities: A scoping review	202	Initiatives to facilitate the social inclusion of people with intellectual disability in physical activities with others in community-based sporting, recreation and leisure activities: A scoping review	Exact normalized title	Merged
Cross-database overlap	S3	30	Exploring the Dynamics of Athletes’ Enjoyment and Self-Determined Motivation, and of the Motivational Climate in Youth Football: A Longitudinal Perspective	379	Exploring the Dynamics of Athletes' Enjoyment and Self-Determined Motivation, and of the Motivational Climate in Youth Football: A Longitudinal Perspective	Exact normalized title	Merged
Cross-database overlap	S3	31	Motivational climate dimensions predict youth soccer players’ psychosocial well-being over time	380	Motivational climate dimensions predict youth soccer players' psychosocial well-being over time	Exact normalized title	Merged
Cross-database overlap	S3	32	Psychological stress and psychological well-being among sports coaches: A close proximity longitudinal daily diary study	194	Psychological stress and psychological well-being among sports coaches: A close proximity longitudinal daily diary study	Exact normalized title	Merged
Cross-database overlap	S3	33	A framework for mental training in sport: Enhancing mental skills, wellbeing, and performance	207	A framework for mental training in sport: Enhancing mental skills, wellbeing, and performance	Exact normalized title	Merged
Cross-database overlap	S3	34	Effect of coaches’ interpersonal style on young athletes’ individual resilience and team adherence intention: a season-long investigation	387	Effect of coaches' interpersonal style on young athletes' individual resilience and team adherence intention: a season-long investigation	Exact normalized title	Merged
Cross-database overlap	S3	36	Understanding mental toughness at a Women's Australian rules football club	237	Understanding mental toughness at a Women?s Australian rules football club	Exact normalized title	Merged
Cross-database overlap	S3	38	A longitudinal investigation of the role of perceived autonomy support from coaches in reducing athletes’ experiential avoidance: The mediating role of subjective vitality	224	A longitudinal investigation of the role of perceived autonomy support from coaches in reducing athletes' experiential avoidance: The mediating role of subjective vitality	Exact normalized title	Merged
Cross-database overlap	S3	39	Exploring the Relationship Between Athletes’ Perceptions of Their Team Motivational Climate and Their Sport Shame	255	Exploring the Relationship Between Athletes' Perceptions of Their Team Motivational Climate and Their Sport Shame	Exact normalized title	Merged
Cross-database overlap	S3	40	The Effects of an Exploratory Mindfulness Intervention on Collegiate Performance	266	The Effects of an Exploratory Mindfulness Intervention on Collegiate Performance	Exact normalized title	Merged
Cross-database overlap	S3	41	Examining links between coaching behaviors and collegiate athletes’ burnout levels using a longitudinal approach	271	Examining links between coaching behaviors and collegiate athletes' burnout levels using a longitudinal approach.	Exact normalized title	Merged
Cross-database overlap	S3	42	A Coach-Delivered Dating Violence Prevention Program: Coach Perspectives on Implementation	263	A Coach-Delivered Dating Violence Prevention Program: Coach Perspectives on Implementation	Exact normalized title	Merged
Cross-database overlap	S3	43	Transcultural Adaptation and Psychometric Support for a Brazilian Portuguese Version of the Flow State Scale (FSS-2)	261	Transcultural Adaptation and Psychometric Support for a Brazilian Portuguese Version of the Flow State Scale (FSS-2)	Exact normalized title	Merged
Cross-database overlap	S3	45	Athletes’ motivational and emotional outcomes related to a Need-Supportive intervention in intensive training centers	253	Athletes' motivational and emotional outcomes related to a Need-Supportive intervention in intensive training centers	Exact normalized title	Merged
Cross-database overlap	S3	46	Longitudinal trajectories of emotions among athletes in sports competitions: Does emotional intelligence matter?	257	Longitudinal trajectories of emotions among athletes in sports competitions: Does emotional intelligence matter?	Exact normalized title	Merged
Cross-database overlap	S3	47	Mindful self-reflection to support sustainable high-performance coaching: A process evaluation of a novel method development in elite sport	63	Mindful self-reflection to support sustainable high-performance coaching: A process evaluation of a novel method development in elite sport	Exact normalized title	Merged
Cross-database overlap	S3	48	The quest for professional self-understanding: Sense making and the interpersonal nature of applied sport psychology practice	65	The quest for professional self-understanding: Sense making and the interpersonal nature of applied sport psychology practice	Exact normalized title	Merged
Cross-database overlap	S3	50	Athletes’ perspectives of preparation strategies in open-skill sports	270	Athletes' perspectives of preparation strategies in open-skill sports	Exact normalized title	Merged
Cross-database overlap	S3	51	The effects of self-compassion on daily emotion regulation and performance rebound among college athletes: Comparisons with confidence, grit, and hope	258	The effects of self-compassion on daily emotion regulation and performance rebound among college athletes: Comparisons with confidence, grit, and hope	Exact normalized title	Merged
Cross-database overlap	S3	52	Role of psychological ownership and authenticity strength in the relationship between authentic leadership and sport team performance: A multilevel analysis	281	Role of psychological ownership and authenticity strength in the relationship between authentic leadership and sport team performance: A multilevel analysis	Exact normalized title	Merged
Cross-database overlap	S3	55	A longitudinal examination of thriving in sport performers	277	A longitudinal examination of thriving in sport performers	Exact normalized title	Merged
Cross-database overlap	S3	58	Home Team (Dis)Advantage Patterns in the National Hockey League: Changes Through Increased Emphasis on Individual Performance With the 3-on-3 Overtime Rule	291	Home Team (Dis)Advantage Patterns in the National Hockey League: Changes Through Increased Emphasis on Individual Performance With the 3-on-3 Overtime Rule	Exact normalized title	Merged
Cross-database overlap	S3	60	Athlete perspectives on the enablers and barriers to nutritional adherence in high-performance sport	76	Athlete perspectives on the enablers and barriers to nutritional adherence in high-performance sport	Exact normalized title	Merged
Cross-database overlap	S3	61	Coach identity leadership behaviours are positively associated with athlete resource appraisals: The mediating roles of relational and group identification	306	Coach identity leadership behaviours are positively associated with athlete resource appraisals: The mediating roles of relational and group identification	Exact normalized title	Merged
Cross-database overlap	S3	62	Development, Implementation, and Evaluation of anAthlete-Informed Mental Skills Training Program for EliteYouth Tennis Players	85	Development, Implementation, and Evaluation of anAthlete-Informed Mental Skills Training Program for EliteYouth Tennis Players	Exact normalized title	Merged
Cross-database overlap	S3	64	The emergence and perpetuation of a destructive culture in an elite sport in the United Kingdom	87	The emergence and perpetuation of a destructive culture in an elite sport in the United Kingdom	Exact normalized title	Merged
Cross-database overlap	S3	65	Understanding social networks and social support resources with sports coaches	302	Understanding social networks and social support resources with sports coaches	Exact normalized title	Merged
Cross-database overlap	S3	67	Individual Pathways Through the Junior-to-Senior Transition: Narratives of Two Swedish Team Sport Athletes	304	Individual Pathways Through the Junior-to-Senior Transition: Narratives of Two Swedish Team Sport Athletes	Exact normalized title	Merged
Cross-database overlap	S3	68	Psychological characteristics, sport engagement, and performance in alpine skiers	301	Psychological characteristics, sport engagement, and performance in alpine skiers	Exact normalized title	Merged
Cross-database overlap	S3	73	Longitudinal trajectories of emotions among young athletes involving in intense training centres: Do emotional intelligence and emotional regulation matter?	404	Longitudinal trajectories of emotions among young athletes involving in intense training centres: Do emotional intelligence and emotional regulation matter?	Exact normalized title	Merged

Cross-database overlap	S3	74	Development of perfectionism in junior athletes: A three-sample study of coach and parental pressure	315	Development of Perfectionism in Junior Athletes: A Three-Sample Study of Coach and Parental Pressure	Exact normalized title	Merged
Cross-database overlap	S3	78	Leveling the playing field: can psychological coping resources reduce the influence of physical and technical skills on athletic performance?*	339	Leveling the playing field: can psychological coping resources reduce the influence of physical and technical skills on athletic performance?	Exact normalized title	Merged
Cross-database overlap	S3	79	A longitudinal examination of elite youth soccer players: The role of passion and basic need satisfaction in athletes’ optimal functioning	107	A longitudinal examination of elite youth soccer players: The role of passion and basic need satisfaction in athletes' optimal functioning	Exact normalized title	Merged
Cross-database overlap	S3	83	Self-talk and competitive balance beam performance	334	Self-Talk and Competitive Balance Beam Performance	Exact normalized title	Merged
Cross-database overlap	S3	85	Stressors, coping, and well-being among sports coaches: A systematic review	341	Stressors, coping, and well-being among sports coaches: A systematic review	Exact normalized title	Merged
Cross-database overlap	S3	88	Linking coach interpersonal style with Athlete doping intentions and doping use: A prospective study	347	Linking Coach Interpersonal Style With Athlete Doping Intentions and Doping Use: A Prospective Study	Exact normalized title	Merged
Cross-database overlap	S3	89	The two roads from passion to sport performance and psychological well- being: The mediating role of need satisfaction, deliberate practice, and achievement goals	344	The two roads from passion to sport performance and psychological well-being: The mediating role of need satisfaction, deliberate practice, and achievement goals	Exact normalized title	Merged
Cross-database overlap	S3	90	Understanding psychological changes in adolescent wrestlers participating in an intensive training camp: A mixed-methods investigation	410	Understanding psychological changes in adolescent wrestlers participating in an intensive training camp: A mixed-methods investigation	Exact normalized title	Merged
Cross-database overlap	S3	92	How perceived autonomy support and controlling coach behaviors are related to well- and ill-being in elite soccer players: A within-person changes and between-person differences analysis	110	How perceived autonomy support and controlling coach behaviors are related to well- and ill-being in elite soccer players: A within-person changes and between-person differences analysis	Exact normalized title	Merged
Cross-database overlap	S3	93	High school athletes' self-determined motivation: The independent and interactive effects of coach, father, and mother autonomy support	414	High school athletes' self-determined motivation: The independent and interactive effects of coach, father, and mother autonomy support	Exact normalized title	Merged
Cross-database overlap	S3	95	The development, implementation, and evaluation of an athlete leadership development program with female varsity athletes	363	The Development, Implementation, and Evaluation of an Athlete Leadership Development Program With Female Varsity Athletes	Exact normalized title	Merged
Scopus-only	S1	2	Assessing toxic leadership in high-performance sports: Psychometric validation of a Swedish Version of the Toxic Leadership Scale for Sports (TLS- S)	418		No verified match	Added
Scopus-only	S1	4	Acceptable or Not: An In-depth Analysis of Adolescent Competitive Athletes’ Perceptions on Abusive Coaching Behaviors	419		No verified match	Added
Scopus-only	S1+S3	5	Interpersonal climate and moral conduct in competitive sport: A dual-route model with serial mediation	420		No verified match	Added
Scopus-only	S1	8	Qualitative analysis of elite football players’ causal attribution to not playing: A perspective of attribution theory	421		No verified match	Added
Scopus-only	S1	17	Using the Conceptual Framework for Examining Sport Teams to Understand Group Dynamics in Professional Soccer	422		No verified match	Added
Scopus-only	S1	19	Examining the psychometric properties of the Controlling Coach Behaviors Scale in Chinese Elite Athletes	423		No verified match	Added
Scopus-only	S1+S3	21	Quality of coach-athlete relationship and coping as associated factors of stress, anxiety, burnout, and depression symptoms of soccer players in transition to professional: a prospective study	424		No verified match	Added
Scopus-only	S1	23	Motivational beliefs and subjective effort in adolescent athletes	425		No verified match	Added
Scopus-only	S1	24	Which are the most determinant psychological factors in olympic shooting performance? A self-perspective from elite shooters	426		No verified match	Added
Scopus-only	S1	27	Perceptions of low back pain in elite gymnastics: A multi-disciplinary qualitative focus group study	427		No verified match	Added
Scopus-only	S1	29	"Coach really knew what i needed and understood me well as a person": Effective communication acts in coach–athlete interactions among Korean olympic archers	428		No verified match	Added
Scopus-only	S1	31	Emotion, psychological well-being and their influence on resilience. A study with semi-professional athletes	429		No verified match	Added
Scopus-only	S1	32	Motivation in the practice of judo in non-professional athletes | Motivación en la práctica del judo en deportistas no profesionales	430		No verified match	Added
Scopus-only	S1	33	Using vignettes to analyze potential influences of effective strength and conditioning coaching on athlete development	431		No verified match	Added
Scopus-only	S1	34	Providing social support to female Olympic athletes	432		No verified match	Added
Scopus-only	S1	35	Influences of perfectionism and motivational climate on attitudes towards doping among Korean national athletes: A cross sectional study	433		No verified match	Added
Scopus-only	S1	36	Pathos and orchestration in elite sport: The experiences of NCAA DI student- athletes	434		No verified match	Added
Scopus-only	S1	37	‘This might be him; the guy I’m gonna marry’: Love and sexual relationships between female elite-athletes and male coaches	435		No verified match	Added
Scopus-only	S2	2	Offended by the algorithm: The hidden interpersonal costs of clients seeking AI second opinion	436		No verified match	Added

Scopus-only	S2	4	One Sprinter, Two Olympic Preparations: A Single-Athlete Longitudinal Observational Study of Training-Intensity Distribution and Implications for Future 50 m Events	437		No verified match	Added
Scopus-only	S2	10	Training Stress, Sleep Behaviour, and Recovery Self-Regulation in Elite Female Boxers: A Behavioural Pattern Analysis Using Data-Driven Methods	438		No verified match	Added
Scopus-only	S2	11	Strategies to reduce hyperglycemia-related anxiety in elite athletes with type 1 diabetes: A qualitative analysis	439		No verified match	Added
Scopus-only	S2	19	Sport Anxiety, Fear of Negative Evaluation, Stress and Coping as Predictors of Athlete's Sensitivity to the Behavior of Supporters	440		No verified match	Added
Scopus-only	S2	24	Depressive symptoms and burnout in football players: A systematic review	441		No verified match	Added
Scopus-only	S3	1	PoseShot: hybrid CNN–BiLSTM transformer model for free throw action recognition via pose analysis	442		No verified match	Added
Scopus-only	S3	3	Prediction of athlete performance based on a gradient regression model	443		No verified match	Added
Scopus-only	S3	5	The development of mental toughness: A journey into the perceptions and experiences of a world-class padel athlete	444		No verified match	Added
Scopus-only	S3	6	Performance inflation in junior tennis: Longitudinal analysis and Bayesian forecasting of ranking thresholds, efficiency, and access equity	445		No verified match	Added
Scopus-only	S3	7	The Lions Sports Academy TackleTEK Tool: The intra-and inter-coach reliability of assessing tackling competency in rugby union	446		No verified match	Added
Scopus-only	S3	12	An intelligent taekwondo coaching system based on augmented reality technology with real-time feedback mechanisms	447		No verified match	Added
Scopus-only	S3	14	Adverse competition-related cognitions and it's relation to satisfaction and subjective performance: a validation study in a sample of English-speaking athletes	448		No verified match	Added
Scopus-only	S3	15	Investigating sport persistence through the development of the Sport Persistence Questionnaire	449		No verified match	Added
Scopus-only	S3	16	Analyzing the impact of prolonged pauses on soccer performance	450		No verified match	Added
Scopus-only	S3	17	Enhancing team dynamics: Unveiling synergy effects through social network analysis in professional basketball	451		No verified match	Added
Scopus-only	S3	18	Adaptation and psychometric properties of Psychological Skills Inventory for Sport (PSIS-R5) in Latvian athletes: Insights and implications for practice	452		No verified match	Added
Scopus-only	S3	20	Longitudinal relationships between emotional processes and perceived performances among athletes: The moderating effect of emotional intelligence	453		No verified match	Added
Scopus-only	S3	21	Creatine kinase and neuromuscular fatigue responses following differing spells of simulated cricket fast bowling	454		No verified match	Added
Scopus-only	S3	24	Effects of brief mindfulness intervention on mental fatigue and recovery in basketball tactical performance	455		No verified match	Added
Scopus-only	S3	25	Impact of offensive team variables on goal scoring in the first division of the spanish soccer league: a comprehensive 10-year study	456		No verified match	Added
Scopus-only	S3	26	Bystander Intervention in Football and Sports. A Quasi-Experimental Feasibility Study of a Bystander Violence Prevention Program in the United Kingdom	457		No verified match	Added
Scopus-only	S3	27	Eye-Tracking Analyses of a Coach's Pointing Gestures Timed With Speech: Implications for Players' Recall of Basketball Tactical Instructions	458		No verified match	Added
Scopus-only	S3	35	Match analysis and probability of winning a point in elite men's singles tennis	459		No verified match	Added
Scopus-only	S3	37	Elite lean athletes at sports high schools face multiple risks for mental health concerns and are in need of psychosocial support	460		No verified match	Added
Scopus-only	S3	49	Amplifying perceptual demands: How changes in the colour vests affect youth players performance during medium-sided games	461		No verified match	Added
Scopus-only	S3	53	Redesigning a Youth Soccer Program: Holistic Development of Athletes	462		No verified match	Added
Scopus-only	S3	54	In-season training responses and perceived wellbeing and recovery status in professional soccer players	463		No verified match	Added
Scopus-only	S3	56	Run-up strategies in competitive long jumping: How an ecological dynamics rationale can support coaches to design individualised practice tasks	464		No verified match	Added
Scopus-only	S3	57	Balance dynamics are related to age and levels of expertise. Application in young and adult tennis players	465		No verified match	Added
Scopus-only	S3	59	Facilitating Positive Youth Development in Elite Youth Hockey: Exploring Coaches' Capabilities, Opportunities, and Motivations	466		No verified match	Added

Scopus-only	S3	66	Athletes' Perception of Leadership According to Their Perceptions of Goal Achievement and Sport Results	467	No verified match	Added
Scopus-only	S3	69	What does it mean to be "mentally tough" as a NCAA division I collegiate coach?	468	No verified match	Added
Scopus-only	S3	70	Young Dutch commercially sponsored kite surfers: free as a bird?	469	No verified match	Added
Scopus-only	S3	71	The Optimal Team Functioning Model: A Grounded Theory Framework to Guide Teamwork in Curling	470	No verified match	Added
Scopus-only	S3	72	Extracting spatial-temporal features that describe a team match demands when considering the effects of the quality of opposition in elite football	471	No verified match	Added
Scopus-only	S3	75	Overcoming the problem of multicollinearity in sports performance data: A novel application of partial least squares correlation analysis	472	No verified match	Added
Scopus-only	S3	76	Relationships between technique and bat speed, post-impact ball speed, and carry distance during a range hitting task in cricket	473	No verified match	Added
Scopus-only	S3	77	The Development and Validation of the Adolescent Sport Drug Inventory (ASDI) Among Athletes From Four Continents	474	No verified match	Added
Scopus-only	S3	80	Do coaching style and game circumstances predict athletes' perceived justice of their coach? A longitudinal study in elite handball and volleyball teams	475	No verified match	Added
Scopus-only	S3	81	Physiological, perceptual, and technical responses to continuous and intermittent small-sided games in lacrosse players	476	No verified match	Added
Scopus-only	S3	82	Relationships between physical qualities and key performance indicators during matchplay in senior international rugby union players	477	No verified match	Added
Scopus-only	S3	84	Positive youth development and observed athlete behavior in recreational sport	478	No verified match	Added
Scopus-only	S3	87	Concussion Knowledge and Communication Behaviors of Collegiate Wrestling Coaches	479	No verified match	Added
Scopus-only	S3	91	A longitudinal examination of stressors, appraisals, and coping in youth swimming	480	No verified match	Added
Scopus-only	S3	94	Patterns of nutrition and dietary supplements use in young Egyptian athletes: A community-based cross-sectional survey	481	No verified match	Added

ID	Title	Authors	Year	DOI	Databases	Strings	Preliminary decision	Title/abstract decision	Screening reason	Population code	Pillar code	Temporal marker	Eligibility decision	Eligibility reason	Evidence tier	Primary phase	Phase evidence	Appraisal total	Evidence weight
1	Coaching under pressure in Australian high-performance sport: A qualitative study of coach mental performance	Fryer, RP; Mallett, CJ; Kirby, JN; Kashdan, TB	2026	10.1177/17479541261430313	WoS	S1	INCLUDE	RETAIN	Potentially eligible; advanced to full-text or extended-abstract assessment	elite	P2, P3	TRUE	INCLUDE	Retained in narrative synthesis	Core	Phase III	Direct	8	High
2	Olympic swimmers' perspectives on how they want to be led	Ferguson, C; Mason, C	2026	10.1177/17479541261446432	WoS	S1	INCLUDE	RETAIN	Potentially eligible; advanced to full-text or extended-abstract assessment	unspecified	P2	TRUE	INCLUDE	Retained in narrative synthesis	Core	Cross-cutting	Indirect	7	Moderate
3	Positive Experiences Among Elite Athletes in Finland: Associations With Basic Psychological Needs, Sport Conditions, and Background Factors	Kaski, SS; Kinnunen, U	2026	10.1123/jcsp.2024-0004	WoS	S1	INCLUDE	RETAIN	Potentially eligible; advanced to full-text or extended-abstract assessment	elite	P2	TRUE	EXCLUDE_AT_ELIGIBILITY	Did not examine the intersection of at least two construct pillars					
4	Understanding coach burnout in high-performance sport: the sequential effects of knowledge, psychological safety, and coach-athlete relationships	Tao, RC; Zhang, NY; Guo, C	2026	10.3389/fpsyg.2026.1785482	SPORTDiscus+WoS	S1	INCLUDE	RETAIN	Potentially eligible; advanced to full-text or extended-abstract assessment	unspecified	P2	TRUE	EXCLUDE_AT_ELIGIBILITY	Did not examine the intersection of at least two construct pillars					
5	Exploring stressors in elite rugby league (NRL/NRLW) players	Humphreys, C; Goddard, SG; Poulus, D	2026	10.1080/10413200.2025.2500963	WoS	S1	INCLUDE	RETAIN	Potentially eligible; advanced to full-text or extended-abstract assessment	elite	P2	TRUE	EXCLUDE_AT_ELIGIBILITY	Did not examine the intersection of at least two construct pillars					
6	Exploring the relationship between burnout and mental health in elite athletes: the role of moderators	de Subijana, CL; Wergin, VV; Mallett, CJ; Haslam, A	2026	10.1080/1612197X.2025.2479715	WoS	S1+S3	INCLUDE	RETAIN	Potentially eligible; advanced to full-text or extended-abstract assessment	elite	P2	TRUE	EXCLUDE_AT_ELIGIBILITY	Did not examine the intersection of at least two construct pillars					
7	Why do elite athletes consider leaving their sports? Longitudinal evidence on the development of dropout considerations	Blessing, J; Hilpisch, C; Krüger, K; Raab, M; Wiese, L; Zentgraf, K; Mutz, M	2026	10.1177/17479541261438700	WoS	S1+S3	INCLUDE	RETAIN	Potentially eligible; advanced to full-text or extended-abstract assessment	elite	P2	TRUE	EXCLUDE_AT_ELIGIBILITY	Did not examine the intersection of at least two construct pillars					
8	I even called him coach at home: A qualitative study on role conflict and coping in parent-coached elite athletes	Alp, AF; Karagün, E	2026	10.1177/17479541251393447	WoS	S1	INCLUDE	RETAIN	Potentially eligible; advanced to full-text or extended-abstract assessment	elite	P3	TRUE	EXCLUDE_AT_ELIGIBILITY	Did not examine the intersection of at least two construct pillars					
9	Unveiling the coach-athlete relationship in Major international competitions: A qualitative study of national team coaches	Braga-Pereira, R; Bernardo-Ramos, J; Castro-Vigário, P; Duarte, D; Teques, P	2026	10.1177/17479541261431543	WoS	S1	INCLUDE	RETAIN	Potentially eligible; advanced to full-text or extended-abstract assessment	elite	P3	TRUE	INCLUDE	Retained in narrative synthesis	Core	Phase III	Direct	8	High
10	Shame following performance failure as a potential mediator between sport contingent self-esteem and both competitive anxiety and burnout among elite athletes	Funke, A; Gustafsson, H	2026	10.1016/j.actpsy.2026.106694	WoS	S1	INCLUDE	RETAIN	Potentially eligible; advanced to full-text or extended-abstract assessment	unspecified	P3	TRUE	EXCLUDE_AT_ELIGIBILITY	Did not examine the intersection of at least two construct pillars					
11	Leadership micro-behaviors and performance stability under competitive pressure: a stepped-wedge field study in professional football	Paasch, R; Mau, G	2026	10.3389/fpsyg.2026.1833589	SPORTDiscus+WoS	S2+S3	INCLUDE	RETAIN	Potentially eligible; advanced to full-text or extended-abstract assessment	elite	P3	TRUE	EXCLUDE_AT_ELIGIBILITY	Did not examine the intersection of at least two construct pillars					
12	Point-ending outcome patterns in break-point situations in professional tennis: Effects of player position and court surface	Mourtzios, C; Gouvelis, N; Mourtziou, MA	2026	10.1177/17479541261442085	WoS	S2	INCLUDE	RETAIN	Potentially eligible; advanced to full-text or extended-abstract assessment	elite	P3	TRUE	EXCLUDE_AT_ELIGIBILITY	Did not examine the intersection of at least two construct pillars					
13	Acute weight cycling and hydration status in elite kickboxers: a repeated-measures field study	Ceylan, B; Taskin, HB; Sarikaya, U; Pekel, HA; Sabirli, TN	2026	10.1186/s13102-026-01669-x	Scopus+WoS	S2	INCLUDE	RETAIN	Potentially eligible; advanced to full-text or extended-abstract assessment	elite	P3	TRUE	EXCLUDE_AT_ELIGIBILITY	Did not examine the intersection of at least two construct pillars					
14	Workload and travel demands of professional basketball staff: A season-long case study across the National Basketball Association and G League	Mercer, RAJ; Russell, JL; Strack, DS; Coutts, AJ; McLean, BD	2026	10.1177/17479541261449029	WoS	S3	INCLUDE	RETAIN	Potentially eligible; advanced to full-text or extended-abstract assessment	unspecified	P3	TRUE	EXCLUDE_AT_ELIGIBILITY	Did not examine the intersection of at least two construct pillars					
15	Features of successful high-performance sport environments: Stakeholder insights	Schlawe, A; Christiansen, AV; Henriksen, K	2026	10.1080/10413200.2026.2683956	WoS	S3	INCLUDE	RETAIN	Potentially eligible; advanced to full-text or extended-abstract assessment	unspecified	P1, P2	TRUE	INCLUDE	Retained in narrative synthesis	Contextual	Cross-cutting	Indirect	7	Moderate
16	Applying the 5Cs Framework to Elite Youth Tennis: Impact Factors in a Talent Development Environment	Harwood, C; Porter, K	2026	10.3390/bs16020166	Scopus+WoS	S3	INCLUDE	RETAIN	Potentially eligible; advanced to full-text or extended-abstract assessment	elite	P1, P2	TRUE	EXCLUDE_AT_ELIGIBILITY	Insufficient fit with the temporal cascade after eligibility assessment					
17	Understanding the motivators and barriers of elite athletes as role models	Praet, L; De Bosscher, V; Smismans, S; Derom, I	2025	10.1080/17430437.2025.2563822	Scopus+WoS	S1	INCLUDE	RETAIN	Potentially eligible; advanced to full-text or extended-abstract assessment	unspecified	P1	TRUE	EXCLUDE_AT_ELIGIBILITY	Did not examine the intersection of at least two construct pillars					
18	Exploring the barriers to athlete personal development within UK Olympic and Paralympic sport	Holden, J; Wagstaff, CRD; Wadey, R; Brown, P	2025	10.1016/j.psychsport.2025.102966	WoS	S1	INCLUDE	RETAIN	Potentially eligible; advanced to full-text or extended-abstract assessment	unspecified	P1	TRUE	EXCLUDE_AT_ELIGIBILITY	Did not examine the intersection of at least two construct pillars					
19	Evaluating a behaviour change intervention to enhance athlete monitoring system engagement: Insights from elite sport	Neupert, EC; Holder, T; Jobson, SA	2025	10.1177/17479541251338804	WoS	S1	INCLUDE	RETAIN	Potentially eligible; advanced to full-text or extended-abstract assessment	elite	P1	TRUE	EXCLUDE_AT_ELIGIBILITY	Did not examine the intersection of at least two construct pillars					
20	Disciplinary strategies or reactive measures? An analysis of coach sanctions in elite Danish handball leagues	Gençoglu, C; Gümüs, H	2025	10.1177/17479541251360847	WoS	S2	INCLUDE	RETAIN	Potentially eligible; advanced to full-text or extended-abstract assessment	elite	P1	TRUE	EXCLUDE_AT_ELIGIBILITY	Did not examine the intersection of at least two construct pillars					
21	The epidemiology of injuries in professional sitting volleyball athletes	Mahmoudkhani, M; Alizadeh, F; Khodsiyani, E; Norouzi, M	2025	10.1080/00913847.2025.2498317	Scopus+WoS	S2	INCLUDE	RETAIN	Potentially eligible; advanced to full-text or extended-abstract assessment	elite	P1	TRUE	EXCLUDE_AT_ELIGIBILITY	Did not examine the intersection of at least two construct pillars					
22	Burnout in medical and performance professionals working in elite football in the United Kingdom: a pilot study	Mifsud, D; Acampora, N; Rao, NL; Shafik, A; Carmody, S; Chandran, A	2025	10.1080/24733938.2025.2476486	WoS	S3	INCLUDE	RETAIN	Potentially eligible; advanced to full-text or extended-abstract assessment	elite	P1	TRUE	EXCLUDE_AT_ELIGIBILITY	Did not examine the intersection of at least two construct pillars					
23	A Longitudinal Study of Organizational Stressors and Mental Health in the Irish Olympic Team Before and After the Tokyo 2020 (2021) Olympic Games	Browne, JA; Paradis, KF; Kirby, K; Arnold, R	2025	10.1123/tsp.2024-0007	WoS	S3	INCLUDE	RETAIN	Potentially eligible; advanced to full-text or extended-abstract assessment	elite	P1	TRUE	EXCLUDE_AT_ELIGIBILITY	Did not examine the intersection of at least two construct pillars					
24	Optimizing recovery: the impact of training load in elite volleyball players	Rebelo, A; Pereira, JR; Vande Broek, G; Coelho-e-Silva, MJ; Elferink-Gemser, MT; Valente-dos-Santos, J	2025	10.1055/a-2519-9330	WoS	S3	INCLUDE	RETAIN	Potentially eligible; advanced to full-text or extended-abstract assessment	elite	P1	TRUE	EXCLUDE_AT_ELIGIBILITY	Did not examine the intersection of at least two construct pillars					
25	Examining the relationship of training load and menstrual cycle on a web application-based load and recovery score: A longitudinal observational study in elite women's soccer	Brefin, SL; Anderegg, J; Nigg, CR; Koschnick, D; Paul, C; Ketelhut, S	2025	10.1177/17479541251349332	WoS	S3	INCLUDE	RETAIN	Potentially eligible; advanced to full-text or extended-abstract assessment	elite	P1	TRUE	EXCLUDE_AT_ELIGIBILITY	Did not examine the intersection of at least two construct pillars					
26	Evidence for the reversal of the relative age effect in European professional basketball	Akinci, Y; Yapar, A	2025	10.1080/24748668.2025.2532271	WoS	S3	INCLUDE	RETAIN	Potentially eligible; advanced to full-text or extended-abstract assessment	elite	P1	TRUE	EXCLUDE_AT_ELIGIBILITY	Did not examine the intersection of at least two construct pillars					
27	Influence of Mental Skills on Competitive Anxiety in Professional Futsal Players: Difference by Successful and Unsuccessful Players.	Kemarat, Supatcharin ; Theanthong, Apiluk ; Yeemin, Wichai ; Suwankan, Sutima ; Makaje, Niromlee	2025	10.1007/s43076-023-00330-7	SPORTDiscus	S2	INCLUDE	RETAIN	Potentially eligible; advanced to full-text or extended-abstract assessment	elite	P2, P3	FALSE	EXCLUDE_AT_ELIGIBILITY	No direct or phase-relevant temporal content					
28	Developing guidelines for selection-deselection in high performance sport for athletes, coaches, and organisations: A delphi study	Slade, K; Jowett, S; Rhind, D	2024	10.1080/02640414.2024.2387968	Scopus+WoS	S1	INCLUDE	RETAIN	Potentially eligible; advanced to full-text or extended-abstract assessment	elite	P2	FALSE	INCLUDE	Retained in narrative synthesis	Core	Selection/transition	Indirect	7	Moderate
29	Resilience profiles of elite athletes and their associations with health-related behaviors, well-being, and performance: A latent profile analysis	Chretien, A; Hayotte, M; Vuillemin, A; Longueville, FD	2024	10.1016/j.psychsport.2024.102689	Scopus+WoS	S1	INCLUDE	RETAIN	Potentially eligible; advanced to full-text or extended-abstract assessment	elite	P2	FALSE	EXCLUDE_AT_ELIGIBILITY	Did not examine the intersection of at least two construct pillars					
30	How is coaches' social identity leadership related to mental health in elite athletes? The mediating role of satisfaction with performance	de Subijana, CL; Pons, J; Mallett, CJ	2024	10.1080/02640414.2024.2425908	Scopus+WoS	S1	INCLUDE	RETAIN	Potentially eligible; advanced to full-text or extended-abstract assessment	elite	P2	FALSE	EXCLUDE_AT_ELIGIBILITY	Did not examine the intersection of at least two construct pillars					
31	The successful experience of gymnastics world champion coach: an interview analysis	Liu, XX; Weng, XH; Qin, HH; Ma, S; Wang, GQ	2024	10.3389/fpsyg.2024.1405589	WoS	S1+S3	INCLUDE	RETAIN	Potentially eligible; advanced to full-text or extended-abstract assessment	elite	P2	FALSE	EXCLUDE_AT_ELIGIBILITY	Did not examine the intersection of at least two construct pillars					
32	Determinants of motivation in world-class musicians and Olympic athletes: Exploring the front and the back side of the medallion	Hatfield, JL	2024	10.1177/03057356231155973	WoS	S1	INCLUDE	RETAIN	Potentially eligible; advanced to full-text or extended-abstract assessment	elite	P2	FALSE	EXCLUDE_AT_ELIGIBILITY	Did not examine the intersection of at least two construct pillars					
33	Factor analysis on the performance of elite male fencing athletes	Kim, HC; Park, KJ	2024	10.23736/S0022-4707.24.16381-5	Scopus+WoS	S2	INCLUDE	RETAIN	Potentially eligible; advanced to full-text or extended-abstract assessment	elite	P2	FALSE	EXCLUDE_AT_ELIGIBILITY	Did not examine the intersection of at least two construct pillars					
34	Orienteering Is More than Just Running! Acute Effect of Competitive Pressure on Autonomic Cardiac Activity among Elite Orienteering Athletes	Gorgulu, R; Oruç, H; Vasile, C; Corlaci, I; Voinea, F	2024	10.3390/medicina60091547	SPORTDiscus+Scopus+WoS	S2	INCLUDE	RETAIN	Potentially eligible; advanced to full-text or extended-abstract assessment	elite	P2	FALSE	EXCLUDE_AT_ELIGIBILITY	Did not examine the intersection of at least two construct pillars					
35	The role of transformational leadership in the associations between coach-athlete relationship and team resilience: A study on elite football players	Karayel, E; Adilogullari, I; Senel, E	2024	10.1186/s40359-024-02043-7	SPORTDiscus+Scopus+WoS	S3	INCLUDE	RETAIN	Potentially eligible; advanced to full-text or extended-abstract assessment	elite	P2	FALSE	INCLUDE	Retained in narrative synthesis	Core	Phase II	Indirect	8	High
36	The use of technology in the assessment and monitorization of a season in a professional padel player: A case study	Carnero-Diaz, A; Pecci, J; Reverte-Pagola, G; Da Silva-Grigoletto, ME	2024	10.1177/17543371231199812	WoS	S3	INCLUDE	RETAIN	Potentially eligible; advanced to full-text or extended-abstract assessment	elite	P2	FALSE	EXCLUDE_AT_ELIGIBILITY	Did not examine the intersection of at least two construct pillars					
37	The Role of Athlete Leadership Quality in the Characteristics of Team Resilience in Elite Soccer Teams: A Cross-Sectional and Longitudinal Mediation of Team Identification	López-Gajardo, MA; González-Ponce, I; García-Calvo, T; Enrich-Alturo, E; Leo, FM	2024	10.1123/jcsp.2022-0022	WoS	S3	INCLUDE	RETAIN	Potentially eligible; advanced to full-text or extended-abstract assessment	elite	P2	FALSE	EXCLUDE_AT_ELIGIBILITY	Did not examine the intersection of at least two construct pillars					
38	The Inducers of an Elite Male Table Tennis Player's Emotional Experience Throughout His Career: A Single Case Study Based on the Critical-Incident Method	Petiot, O; Kermarrec, G; Visioli, J; Martin, G	2024	10.1123/tsp.2023-0094	WoS	S3	INCLUDE	RETAIN	Potentially eligible; advanced to full-text or extended-abstract assessment	elite	P2	FALSE	EXCLUDE_AT_ELIGIBILITY	Did not examine the intersection of at least two construct pillars					
39	Integration of golf practise and strength and conditioning in golf: Insights from professional golf coaches	Bliss, A; Langdown, B	2024	10.1177/17479541231199657	WoS	S3	INCLUDE	RETAIN	Potentially eligible; advanced to full-text or extended-abstract assessment	elite	P2	FALSE	EXCLUDE_AT_ELIGIBILITY	Did not examine the intersection of at least two construct pillars					
40	PRE-COMPETITION TAPERING IN HIGH PERFORMANCE SPORTS -- A THEORETICAL APPROACH.	NEGRU, Ioan Niculaie ; NUŢ, Ramona Ancuţa	2024	10.24193/subbeag.69(4).31	SPORTDiscus	S2	INCLUDE	RETAIN	Potentially eligible; advanced to full-text or extended-abstract assessment	elite	P2	FALSE	EXCLUDE_AT_ELIGIBILITY	Did not examine the intersection of at least two construct pillars					
41	Coach-athlete relationship and burnout symptoms among young elite athletes and the role of mental toughness as a moderator.	Gerber, Markus ; Gygax, Basil ; Cody, Robyn	2024	10.1024/2674-0052/a000071	SPORTDiscus	S3	INCLUDE	RETAIN	Potentially eligible; advanced to full-text or extended-abstract assessment	elite	P2	FALSE	EXCLUDE_AT_ELIGIBILITY	Did not examine the intersection of at least two construct pillars					
42	The under representation of women coaches in high-performance sport	Serpell, BG; Harrison, D; Dower, R; Cook, CJ	2023	10.1177/17479541231160229	WoS	S1	INCLUDE	RETAIN	Potentially eligible; advanced to full-text or extended-abstract assessment	elite	P2	FALSE	EXCLUDE_AT_ELIGIBILITY	Did not examine the intersection of at least two construct pillars					
43	Interpersonal Behaviors Questionnaire in Sport: Psychometric Analysis With Romanian Professional Athletes	Alexe, DI; Cîrtita, BC; Tohanean, DI; Larion, A; Alexe, CI; Dragos, P; Burgueño, R	2023	10.1177/00315125221135669	Scopus+WoS	S1	INCLUDE	RETAIN	Potentially eligible; advanced to full-text or extended-abstract assessment	elite	P3	FALSE	EXCLUDE_AT_ELIGIBILITY	Did not examine the intersection of at least two construct pillars					
44	The impact of training environment on Tokyo Olympic athletes' wellbeing: a qualitative multiple case study	Paoletti, V; Mayglothling, T; Mills, JP	2023	10.7352/IJSP.2023.54.348	WoS	S1	INCLUDE	RETAIN	Potentially eligible; advanced to full-text or extended-abstract assessment	elite	P3	FALSE	EXCLUDE_AT_ELIGIBILITY	Did not examine the intersection of at least two construct pillars					
45	Inescapable tensions: performance and/or psychological well-being in Olympic and Paralympic athletes during sport disruption	Trainor, LR; Bennett, EV; Bundon, AM; Tremblay, M; Mannella, S; Crocker, PRE	2023	10.1080/2159676X.2023.2175899	WoS	S1	INCLUDE	RETAIN	Potentially eligible; advanced to full-text or extended-abstract assessment	elite	P3	FALSE	EXCLUDE_AT_ELIGIBILITY	Did not examine the intersection of at least two construct pillars					
46	Lifestyle challenges and mental health of professional tennis players: an exploratory case study	Shrom, SJ; Cumming, J; Fenton, SJ	2023	10.1080/1612197X.2022.2099947	WoS	S1	INCLUDE	RETAIN	Potentially eligible; advanced to full-text or extended-abstract assessment	elite	P3	FALSE	EXCLUDE_AT_ELIGIBILITY	Did not examine the intersection of at least two construct pillars					
47	Heart Rate Variability in Elite International ITF Junior Davis Cup Tennis Players	Villafaina, S; Crespo, M; Martinez-Gallego, R; Fuentes-Garcia, JP	2023	10.3390/biology12010017	WoS	S2	INCLUDE	RETAIN	Potentially eligible; advanced to full-text or extended-abstract assessment	elite	P3	FALSE	EXCLUDE_AT_ELIGIBILITY	Did not examine the intersection of at least two construct pillars					

48	Balancing risk-taking and self-care: The ecology of athlete health behaviour during the Olympic qualification phase	Schubring, A; Hallten, M; Barker-Ruchti, N; Post, A	2023	10.1177/10126902231168098	WoS	S3	INCLUDE	RETAIN	Potentially eligible; advanced to full-text or extended-abstract assessment	elite	P3	FALSE	EXCLUDE_AT_ELIGIBILITY	Did not examine the intersection of at least two construct pillars					
49	Thinking About Elite Performance: The Experience and Impact of Mental Fatigue in Elite Sport Coaching	Russell, S; Halson, SL; Jenkins, DG; Rynne, SB; Roelands, B; Kelly, VG	2023	10.1123/ijspp.2023-0033	WoS	S3	INCLUDE	RETAIN	Potentially eligible; advanced to full-text or extended-abstract assessment	elite	P3	FALSE	EXCLUDE_AT_ELIGIBILITY	Did not examine the intersection of at least two construct pillars					
50	Are Metabolic Power Distribution and Accelerometer-Based Global Positioning System Variables Associated With Odds Ratios of Noncontact Injuries in Professional Soccer Players?	Nobari, H; Alves, AR; Abbasi, H; Khezri, D; Zamorano, AD; Bowman, TG	2023	10.1519/JSC.0000000000004475	WoS	S3	INCLUDE	RETAIN	Potentially eligible; advanced to full-text or extended-abstract assessment	elite	P3	FALSE	EXCLUDE_AT_ELIGIBILITY	Did not examine the intersection of at least two construct pillars					
51	Multilevel Analysis of Coach Leadership, Group Cohesion and Collective Efficacy in Semiprofessional Football Teams	Leo, FM; González-Ponce, ; Pulido, JJ; López-Gajardo, MA; García-Calvo, T	2022	10.7352/IJSP.2022.53.378	WoS	S1	INCLUDE	RETAIN	Potentially eligible; advanced to full-text or extended-abstract assessment	elite	P3	FALSE	EXCLUDE_AT_ELIGIBILITY	Did not examine the intersection of at least two construct pillars					
52	Desired coaching behaviours of elite divers during competition	Henderson, S; Bloom, GA; Alexander, D	2022	10.1080/1612197X.2021.2003417	WoS	S1	INCLUDE	RETAIN	Potentially eligible; advanced to full-text or extended-abstract assessment	elite	P3	FALSE	EXCLUDE_AT_ELIGIBILITY	Did not examine the intersection of at least two construct pillars					
53	An Evidence-Informed Framework to Promote Mental Wellbeing in Elite Sport	Purcell, R; Pilkington, V; Carberry, S; Reid, D; Gwyther, K; Hall, K; Deacon, A; Manon, R; Walton, CC; Rice, S	2022	10.3389/fpsyg.2022.780359	WoS	S1	INCLUDE	RETAIN	Potentially eligible; advanced to full-text or extended-abstract assessment	elite	P3	FALSE	EXCLUDE_AT_ELIGIBILITY	Did not examine the intersection of at least two construct pillars					
54	Well-being in elite sport: The specific role of sport-related demands, resources, and recovery from sport	Balk, YA; de Jonge, J; Geurts, SAE	2022	10.5117/Go2022.3.001.BALK	WoS	S1	INCLUDE	RETAIN	Potentially eligible; advanced to full-text or extended-abstract assessment	elite	P3	FALSE	EXCLUDE_AT_ELIGIBILITY	Did not examine the intersection of at least two construct pillars					
55	Predicting sports performance from well-being: A mapping of professional athletes', amateur athletes' and non-athletes' positions	Fruchart, E; Rulence-Pâques, P	2022	10.1016/j.erap.2022.100793	WoS	S1	INCLUDE	RETAIN	Potentially eligible; advanced to full-text or extended-abstract assessment	unspecified	P3	FALSE	EXCLUDE_AT_ELIGIBILITY	Did not examine the intersection of at least two construct pillars					
56	Orientation and motivational climate in elite handball players: Multivariate modeling of performance	Kristjánsdóttir, H; Matthíasdóttir, A; Saavedra, JM	2022	10.1080/19012276.2021.1922304	SPORTDiscus+WoS	S1	INCLUDE	RETAIN	Potentially eligible; advanced to full-text or extended-abstract assessment	elite	P3	FALSE	EXCLUDE_AT_ELIGIBILITY	Did not examine the intersection of at least two construct pillars					
57	Creating an environment for thriving: An ethnographic exploration of a British decentralised Olympic and Paralympic Sport Organisation	Passaportis, MJR; Brown, DJ; Wagstaff, CRD; Arnold, R; Hays, K	2022	10.1016/j.psychsport.2022.102247	WoS	S1	INCLUDE	RETAIN	Potentially eligible; advanced to full-text or extended-abstract assessment	elite	P3	FALSE	EXCLUDE_AT_ELIGIBILITY	Did not examine the intersection of at least two construct pillars					
58	Satisfaction with life in Special Olympic athletes: the role of autonomy support and basic need fulfilment	Komenda, S; Springstein, T; Zrnic, I; Zeilinger, E; Franken, F; Weber, G	2022	10.1080/20473869.2021.1917110	Scopus+WoS	S1	INCLUDE	RETAIN	Potentially eligible; advanced to full-text or extended-abstract assessment	elite	P3	FALSE	EXCLUDE_AT_ELIGIBILITY	Did not examine the intersection of at least two construct pillars					
59	Stakeholder insights into athlete attrition in the high-performance pathway	Guevara, SA; Donaldson, A; Keegan, RJ; Appaneal, RN; Smyth, EA; Waddington, G; Mahony, K; Drew, MK	2022	10.1016/j.jsams.2022.05.011	Scopus+WoS	S2	INCLUDE	RETAIN	Potentially eligible; advanced to full-text or extended-abstract assessment	elite	P3	FALSE	EXCLUDE_AT_ELIGIBILITY	Did not examine the intersection of at least two construct pillars					
60	Rapid weight loss among elite-level judo athletes: methods and nutrition in relation to competition performance	Stangar, M; Stangar, A; Shtyrba, V; Cigic, B; Benedik, E	2022	10.1080/15502783.2022.2099231	Scopus+WoS	S2	INCLUDE	RETAIN	Potentially eligible; advanced to full-text or extended-abstract assessment	elite	P1, P2	FALSE	EXCLUDE_AT_ELIGIBILITY	No direct or phase-relevant temporal content					
61	The prevalence of pre-conditioning and recovery strategies in senior elite and non-elite amateur boxing	Finlay, MJ; Page, RM; Greig, M; Bridge, CA	2022	10.1080/00913847.2021.1931525	Scopus+WoS	S2	INCLUDE	RETAIN	Potentially eligible; advanced to full-text or extended-abstract assessment	elite	P1, P2	FALSE	EXCLUDE_AT_ELIGIBILITY	No direct or phase-relevant temporal content					
62	An exploration of reciprocity between female athletes and their coach in elite junior swimming: a shared reality theory perspective	Solstad, BE; Granerud, M; Haraldsen, HM; Gustafsson, H; Knight, CJ	2022	10.1080/2159676X.2021.1941211	WoS	S3	INCLUDE	RETAIN	Potentially eligible; advanced to full-text or extended-abstract assessment	elite	P1, P2	FALSE	EXCLUDE_AT_ELIGIBILITY	No direct or phase-relevant temporal content					
63	Mindful self-reflection to support sustainable high-performance coaching: A process evaluation of a novel method development in elite sport	Hägglund, K; Kenttä, G; Thelwell, R; Wagstaff, CRD	2022	10.1080/10413200.2021.1925782	Scopus+WoS	S3	INCLUDE	RETAIN	Potentially eligible; advanced to full-text or extended-abstract assessment	elite	P1, P2	FALSE	EXCLUDE_AT_ELIGIBILITY	No direct or phase-relevant temporal content					
64	Stroke Kinematics, Temporal Patterns, Neuromuscular Activity, Pacing and Kinetics in Elite Breaststroke Swimming: A Systematic Review	Nicol, E; Pearson, S; Saxby, D; Minahan, C; Tor, E	2022	10.1186/s40798-022-00467-2	WoS	S3	INCLUDE	RETAIN	Potentially eligible; advanced to full-text or extended-abstract assessment	elite	P1, P2	FALSE	EXCLUDE_AT_ELIGIBILITY	No direct or phase-relevant temporal content					
65	The quest for professional self-understanding: Sense making and the interpersonal nature of applied sport psychology practice	Haluch, P; Radcliffe, J; Rowley, C	2022	10.1080/10413200.2021.1914772	Scopus+WoS	S3	INCLUDE	RETAIN	Potentially eligible; advanced to full-text or extended-abstract assessment	elite	P1, P3	FALSE	EXCLUDE_AT_ELIGIBILITY	No direct or phase-relevant temporal content					
66	Implementing Skill Acquisition Research in High-Performance Sport: Reflecting on the Importance of Autonomy-Support for Successful Collaboration.	Morris-Binelli, Khaya ; Müller, Sean ; van Rens, Fleur E. C. A. ; Staniforth, Dave ; Appleby, Brendyn ; Rosalie, Simon M.	2022	10.1080/21520704.2021.1964661	SPORTDiscus	S1	INCLUDE	RETAIN	Potentially eligible; advanced to full-text or extended-abstract assessment	elite	P1, P3	FALSE	EXCLUDE_AT_ELIGIBILITY	No direct or phase-relevant temporal content					
67	A cluster analysis of high-performance female team players' perceived motivational climate: Implications on perceived motor competence and autonomous behaviour.	Abraldes, J Arturo ; Conte Marín, Luis ; Manzano-Sánchez, David ; Gómez-López, Manuel ; Sánchez-Alcaraz, Bernardino J	2022	10.1371/journal.pone.0278572	SPORTDiscus+Scopus	S1	INCLUDE	RETAIN	Potentially eligible; advanced to full-text or extended-abstract assessment	elite	P1, P3	FALSE	EXCLUDE_AT_ELIGIBILITY	No direct or phase-relevant temporal content					
68	Dark traits as a potential feature of leadership in the high-performance sports coach	Serpell, BG; Harrison, D; Lyons, M; Cook, CJ	2021	10.1177/1747954120964059	WoS	S1	INCLUDE	RETAIN	Potentially eligible; advanced to full-text or extended-abstract assessment	elite	P1, P3	FALSE	EXCLUDE_AT_ELIGIBILITY	No direct or phase-relevant temporal content					
69	The podium illusion: a phenomenological study of the influence of social support on well-being and performance in elite para swimmers	Aitchison, B; Rushton, AB; Martin, P; Soundy, A; Heneghan, NR	2021	10.1186/s13102-021-00269-1	WoS	S1	INCLUDE	RETAIN	Potentially eligible; advanced to full-text or extended-abstract assessment	elite	P1	FALSE	EXCLUDE_AT_ELIGIBILITY	Did not examine the intersection of at least two construct pillars					
70	Psychosocial functioning of Olympic coaches and its perceived effect on athlete performance: a systematic review	Cook, GM; Fletcher, D; Carroll, C	2021	10.1080/1750984X.2020.1802769	WoS	S1	INCLUDE	RETAIN	Potentially eligible; advanced to full-text or extended-abstract assessment	elite	P1	FALSE	EXCLUDE_AT_ELIGIBILITY	Did not examine the intersection of at least two construct pillars					
71	A repeated-measures examination of organizational stressors, perceived psychological and physical health, and perceived performance in semi-elite athletes	Simms, M; Arnold, R; Turner, JE; Hays, K	2021	10.1080/02640414.2020.1804801	WoS	S3	INCLUDE	RETAIN	Potentially eligible; advanced to full-text or extended-abstract assessment	elite	P1	FALSE	EXCLUDE_AT_ELIGIBILITY	Did not examine the intersection of at least two construct pillars					
72	A Field-Based Approach to Determine Soft Tissue Injury Risk in Elite Futsal Using Novel Machine Learning Techniques	Ruiz-Pérez, I; López-Valenciano, A; Hernández-Sánchez, S; Puerta-Callejón, JM; Croix, MD; de Baranda, PS; Ayala, F	2021	10.3389/fpsyg.2021.610210	WoS	S3	INCLUDE	RETAIN	Potentially eligible; advanced to full-text or extended-abstract assessment	elite	P1	FALSE	EXCLUDE_AT_ELIGIBILITY	Did not examine the intersection of at least two construct pillars					
73	Longitudinal Physiological and Fitness Evaluations in Elite Ice Hockey: A Systematic Review	Chiarlitti, NA; Crozier, M; Insogna, JA; Reid, RER; Delisle-Houde, P	2021	10.1519/JSC.0000000000004115	WoS	S3	INCLUDE	RETAIN	Potentially eligible; advanced to full-text or extended-abstract assessment	elite	P1	FALSE	EXCLUDE_AT_ELIGIBILITY	Did not examine the intersection of at least two construct pillars					
74	The influence of macrocultural change on national governing bodies in British olympic sports	Feddersen, NB; Morris, R; Abrahamsen, FE; Littlewood, MA; Richardson, DJ	2021	10.1080/17430437.2020.1771306	WoS	S3	INCLUDE	RETAIN	Potentially eligible; advanced to full-text or extended-abstract assessment	elite	P1	FALSE	EXCLUDE_AT_ELIGIBILITY	Did not examine the intersection of at least two construct pillars					
75	A Longitudinal Study of Power Relations in a British Olympic Sport Organization	Feddersen, NB; Morris, R; Storm, LK; Littlewood, MA; Richardson, DJ	2021	10.1123/jsm.2020-0119	WoS	S3	INCLUDE	RETAIN	Potentially eligible; advanced to full-text or extended-abstract assessment	elite	P1	FALSE	EXCLUDE_AT_ELIGIBILITY	Did not examine the intersection of at least two construct pillars					
76	Athlete perspectives on the enablers and barriers to nutritional adherence in high-performance sport	Bentley, MRN; Patterson, LB; Mitchell, N; Backhouse, SH	2021	10.1016/j.psychsport.2020.101831	Scopus+WoS	S1	INCLUDE	RETAIN	Potentially eligible; advanced to full-text or extended-abstract assessment	elite	P1	FALSE	EXCLUDE_AT_ELIGIBILITY	Did not examine the intersection of at least two construct pillars					
77	More than just another bib: group dynamics in an elite Nordic ski team	Cascagnette, JCW; Benson, AJ; Cruwys, T; Haslam, SA; Bruner, MW	2021	10.1080/02640414.2020.1840040	WoS	S3	INCLUDE	RETAIN	Potentially eligible; advanced to full-text or extended-abstract assessment	elite	P1	FALSE	EXCLUDE_AT_ELIGIBILITY	Did not examine the intersection of at least two construct pillars					
78	Positive youth development and gender differences in high performance sport	O'Connor, D; Gardner, L; Larkin, P; Pope, A; Williams, AM	2020	10.1080/02640414.2019.1698001	Scopus+WoS	S1	INCLUDE	RETAIN	Potentially eligible; advanced to full-text or extended-abstract assessment	elite	P1	FALSE	EXCLUDE_AT_ELIGIBILITY	Did not examine the intersection of at least two construct pillars					
79	Olympic and Paralympic coach perspectives on effective skill acquisition support and coach development	Dehghansai, N; Headrick, J; Renshaw, ; Pinder, RA; Barris, S	2020	10.1080/13573322.2019.1631784	Scopus+WoS	S1+S3	INCLUDE	RETAIN	Potentially eligible; advanced to full-text or extended-abstract assessment	elite	P1	FALSE	INCLUDE	Retained in narrative synthesis	Contextual	Cross-cutting	Indirect	6	Moderate
80	Enhancement of gratitude in the context of elite athletes: outcomes and challenges	Howells, K; Fitzallen, N	2020	10.1080/2159676X.2019.1679868	WoS	S1	INCLUDE	RETAIN	Potentially eligible; advanced to full-text or extended-abstract assessment	elite	P1	FALSE	EXCLUDE_AT_ELIGIBILITY	Did not examine the intersection of at least two construct pillars					
81	The 5C's Model of Successful Partnerships in Elite Beach Volleyball Dyads	Poczwardowski, A; Lamphere, B; Allen, K; Marican, R; Haberl, P	2020	10.1080/10413200.2019.1573205	Scopus+WoS	S1	INCLUDE	RETAIN	Potentially eligible; advanced to full-text or extended-abstract assessment	elite	P1	FALSE	EXCLUDE_AT_ELIGIBILITY	Did not examine the intersection of at least two construct pillars					
82	Interplay of motivation and self-regulation throughout the development of elite athletes	Jordalen, G; Lemyre, PN; Durand-Bush, N	2020	10.1080/2159676X.2019.1585388	WoS	S3	INCLUDE	RETAIN	Potentially eligible; advanced to full-text or extended-abstract assessment	elite	P1	FALSE	EXCLUDE_AT_ELIGIBILITY	Did not examine the intersection of at least two construct pillars					
83	Is the Acute: Chronic Workload Ratio (ACWR) Associated with Risk of Time-Loss Injury in Professional Team Sports? A Systematic Review of Methodology, Variables and Injury Risk in Practical Situations	Andrade, R; Wik, EH; Rebelo-Marques, A; Blanch, P; Whiteley, R; Espregueira-Mendes, J; Gabbett, TJ	2020	10.1007/s40279-020-01308-6	WoS	S3	INCLUDE	RETAIN	Potentially eligible; advanced to full-text or extended-abstract assessment	elite	P1	FALSE	EXCLUDE_AT_ELIGIBILITY	Did not examine the intersection of at least two construct pillars					
84	Does the longitudinal development of physical and anthropometric characteristics associate with professional career attainment in adolescent Australian footballers?	Cripps, AJ; Banyard, HG; Woods, CT; Joyce, C; Hopper, LS	2020	10.1177/1747954120927117	WoS	S3	INCLUDE	RETAIN	Potentially eligible; advanced to full-text or extended-abstract assessment	elite	P1	FALSE	EXCLUDE_AT_ELIGIBILITY	Did not examine the intersection of at least two construct pillars					
85	Development, Implementation, and Evaluation of anAthlete-Informed Mental Skills Training Program for EliteYouth Tennis Players	Dohme, LC; Bloom, GA; Piggott, D; Backhouse, S	2020	10.1080/10413200.2019.1573204	Scopus+WoS	S3	INCLUDE	RETAIN	Potentially eligible; advanced to full-text or extended-abstract assessment	elite	P1	FALSE	EXCLUDE_AT_ELIGIBILITY	Did not examine the intersection of at least two construct pillars					
86	Salivary IgA as a Predictor of Upper Respiratory Tract Infections and Relationship to Training Load in Elite Rugby Union Players	Tiernan, C; Lyons, M; Comyns, T; Nevill, AM; Warrington, G	2020	10.1519/JSC.0000000000003019	WoS	S3	INCLUDE	RETAIN	Potentially eligible; advanced to full-text or extended-abstract assessment	elite	P1	FALSE	EXCLUDE_AT_ELIGIBILITY	Did not examine the intersection of at least two construct pillars					
87	The emergence and perpetuation of a destructive culture in an elite sport in the United Kingdom	Feddersen, NB; Morris, R; Littlewood, MA; Richardson, DJ	2020	10.1080/17430437.2019.1680639	Scopus+WoS	S3	INCLUDE	RETAIN	Potentially eligible; advanced to full-text or extended-abstract assessment	elite	P1	FALSE	EXCLUDE_AT_ELIGIBILITY	Did not examine the intersection of at least two construct pillars					
88	Measurement Characteristics of Athlete Monitoring Tools in Professional Australian Football	Ryan, S; Pacecca, E; Tebble, J; Hocking, J; Kempton, T; Coutts, AJ	2020	10.1123/ijspp.2019-0060	WoS	S3	INCLUDE	RETAIN	Potentially eligible; advanced to full-text or extended-abstract assessment	elite	P1	FALSE	EXCLUDE_AT_ELIGIBILITY	Did not examine the intersection of at least two construct pillars					
89	A Pre-Match Video Self-Modeling Intervention in Elite Youth Football	Middlemas, S; Harwood, C	2020	10.1080/10413200.2019.1590481	WoS	S3	INCLUDE	RETAIN	Potentially eligible; advanced to full-text or extended-abstract assessment	elite	P1	FALSE	EXCLUDE_AT_ELIGIBILITY	Did not examine the intersection of at least two construct pillars					
90	Tapering practices of elite CrossFit athletes	Pritchard, HJ; Keogh, JW; Winwood, PW	2020	10.1177/1747954120934924	WoS	S3	INCLUDE	RETAIN	Potentially eligible; advanced to full-text or extended-abstract assessment	elite	P1	FALSE	EXCLUDE_AT_ELIGIBILITY	Did not examine the intersection of at least two construct pillars					
91	The Cognitive Component of Elite High Jumpers' Preperformance Routines	Gretton, T; Blom, L; Hankemeier, D; Judge, L	2020	10.1123/tsp.2019-0093	WoS	S3	INCLUDE	RETAIN	Potentially eligible; advanced to full-text or extended-abstract assessment	elite	P1	FALSE	EXCLUDE_AT_ELIGIBILITY	Did not examine the intersection of at least two construct pillars					
92	Sports performers' perspectives on facilitating thriving in professional rugby contexts	Brown, DJ; Arnold, R	2019	10.1016/j.psychsport.2018.09.008	WoS	S1	INCLUDE	RETAIN	Potentially eligible; advanced to full-text or extended-abstract assessment	elite	P1	FALSE	EXCLUDE_AT_ELIGIBILITY	Did not examine the intersection of at least two construct pillars					
93	A heuristic for the relationship between caring coaching and elite athlete performance	Fisher, LA; Larsen, LK; Bejar, MP; Shigeno, TC	2019	10.1177/1747954119827192	WoS	S1	MAYBE	RETAIN	Potentially eligible; advanced to full-text or extended-abstract assessment	unspecified	P3	TRUE	EXCLUDE_AT_ELIGIBILITY	Did not examine the intersection of at least two construct pillars					
94	Exploring the Coach's Role in Fostering Resilience in Elite Athletes	Kegelaers, J; Wylleman, P	2019	10.1037/spy0000151	WoS	S1	MAYBE	RETAIN	Potentially eligible; advanced to full-text or extended-abstract assessment	unspecified	P1	FALSE	INCLUDE	Retained in narrative synthesis	Contextual	Cross-cutting	Indirect	7	Moderate
95	Antecedents and consequences of perceived autonomy support in elite sport: A diary study linking coaches' off-job recovery and athletes' performance satisfaction	Balk, YA; de Jonge, J; Geurts, SAE; Oerlemans, WGM	2019	10.1016/j.psychsport.2019.04.020	SPORTDiscus+WoS	S1	MAYBE	RETAIN	Potentially eligible; advanced to full-text or extended-abstract assessment	mixed	P2	FALSE	INCLUDE	Retained in narrative synthesis	Core	Phase II	Direct	9	High
96	MOTIVATION IN THE PRACTICE OF JUDO IN NON-PROFESSIONAL ATHLETES	Castro-Sánchez, M; Zurita-Ortega, F; Zafra-Santos, E; Rodríguez-Fernández, S; Chacón-Cuberos, R; Valdivia-Moral, P	2019	10.15366/rimcafd2019.74.005	WoS	S1	MAYBE	RETAIN	Potentially eligible; advanced to full-text or extended-abstract assessment	unspecified	P3	FALSE	EXCLUDE_AT_ELIGIBILITY	Did not examine the intersection of at least two construct pillars					
97	Burnout symptoms and recovery processes in eight elite soccer coaches over 10 years	Hassmén, P; Kenttä, G; Hjälm, S; Lundkvist, E; Gustafsson, H	2019	10.1177/1747954119851246	WoS	S3	MAYBE	RETAIN	Potentially eligible; advanced to full-text or extended-abstract assessment	unspecified	P3	FALSE	EXCLUDE_AT_ELIGIBILITY	Did not examine the intersection of at least two construct pillars					

98	It's not about sport, it's about you: An interpretative phenomenological analysis of mentoring elite athletes	Sandardos, SS; Chambers, TP	2019	10.1016/j.psychsport.2019.02.003	WoS	S3	MAYBE	RETAIN	Potentially eligible; advanced to full-text or extended-abstract assessment	unspecified	P1	TRUE	EXCLUDE_AT_ELIGIBILITY	Did not examine the intersection of at least two construct pillars					
99	Motivational processes affecting the perception of organizational and media stressors among professional football players: A longitudinal mixed methods research study	Kristiansen, E; Ivarsson, A; Solstad, BE; Roberts, GC	2019	10.1016/j.psychsport.2019.02.009	WoS	S3	MAYBE	RETAIN	Potentially eligible; advanced to full-text or extended-abstract assessment	unspecified	P2	TRUE	EXCLUDE_AT_ELIGIBILITY	Did not examine the intersection of at least two construct pillars					
100	The relationship of team and individual athlete performances on match quarter outcome in elite women's Australian Rules football	Cust, EE; Sweeting, AJ; Ball, K; Anderson, H; Robertson, S	2019	10.1016/j.jsams.2019.05.004	WoS	S3	MAYBE	RETAIN	Potentially eligible; advanced to full-text or extended-abstract assessment	unspecified	P2	TRUE	EXCLUDE_AT_ELIGIBILITY	Did not examine the intersection of at least two construct pillars					
101	Training, match and non-rugby activities in elite male youth rugby union players in England	Hendricks, S; Till, K; Weaving, D; Powell, A; Kemp, S; Stokes, K; Jones, B	2019	10.1177/1747954119829289	WoS	S3	MAYBE	RETAIN	Potentially eligible; advanced to full-text or extended-abstract assessment	unspecified	P2	TRUE	EXCLUDE_AT_ELIGIBILITY	Did not examine the intersection of at least two construct pillars					
102	Coaching Efficacy, Player Perceptions of Coaches' Leadership Styles, and Team Performance in Premier League Soccer	Keatlholetswe, L; Malete, L	2019	10.1080/02701367.2018.1563277	WoS	S3	MAYBE	RETAIN	Potentially eligible; advanced to full-text or extended-abstract assessment	unspecified	P1	TRUE	EXCLUDE_AT_ELIGIBILITY	Elite/professional status not confirmed					
103	Elite female footballers' stories of sociocultural factors, emotions, and behaviours prior to anterior cruciate ligament injury	Ivarsson, A; Johnson, U; Karlsson, J; Börjesson, M; Hägglund, M; Andersen, MB; Waldén, M	2019	10.1080/1612197X.2018.1462227	WoS	S3	MAYBE	RETAIN	Potentially eligible; advanced to full-text or extended-abstract assessment	mixed	P1, P3	FALSE	EXCLUDE_AT_ELIGIBILITY	No direct or phase-relevant temporal content					
104	Effects of Self-Talk Training on Competitive Anxiety, Self-Efficacy, Volitional Skills, and Performance: An Intervention Study with Junior Sub-Elite Athletes.	Walter, Nadja ; Nikoleizig, Lucie ; Alfermann, Dorothee	2019	10.3390/sports7060148	SPORTDiscus	S2	MAYBE	RETAIN	Potentially eligible; advanced to full-text or extended-abstract assessment	unspecified	P3	FALSE	EXCLUDE_AT_ELIGIBILITY	Did not examine the intersection of at least two construct pillars					
105	Need-supportiveness and athlete well-being: Coaches' competence-support at risk in the elite sport context throughout the season.	Berntsen, Hedda ; Ivarsson, Andreas ; Kristiansen, Elsa	2019	10.15203/CISS_2019.010	SPORTDiscus	S3	MAYBE	RETAIN	Potentially eligible; advanced to full-text or extended-abstract assessment	unspecified	P3	FALSE	EXCLUDE_AT_ELIGIBILITY	Did not examine the intersection of at least two construct pillars					
106	Optimal Development of Youth Athletes Toward Elite Athletic Performance: How to Coach Their Motivation, Plan Exercise Training, and Pace the Race.	Menting, Stein G P ; Hendry, David T ; Schiphof-Godart, Lieke ; Elferink-Gemser, Marije T ; Hettinga, Florentina J	2019	10.3389/fspor.2019.00014	SPORTDiscus	S3	MAYBE	RETAIN	Potentially eligible; advanced to full-text or extended-abstract assessment	unspecified	P3	FALSE	EXCLUDE_AT_ELIGIBILITY	Did not examine the intersection of at least two construct pillars					
107	A longitudinal examination of elite youth soccer players: The role of passion and basic need satisfaction in athletes' optimal functioning	Verner-Filion, J; Vallerand, RJ	2018	10.1016/j.psychsport.2018.07.005	Scopus+WoS	S3	MAYBE	RETAIN	Potentially eligible; advanced to full-text or extended-abstract assessment	unspecified	P3	FALSE	INCLUDE	Retained in narrative synthesis	Contextual	Phase II	Direct	8	High
108	Champions are racers, not pacers: an analysis of qualification patterns of Olympic and IAAF World Championship middle distance runners	Hanley, B; Hettinga, FJ	2018	10.1080/02640414.2018.1472200	WoS	S3	MAYBE	RETAIN	Potentially eligible; advanced to full-text or extended-abstract assessment	unspecified	P2	TRUE	EXCLUDE_AT_ELIGIBILITY	Did not examine the intersection of at least two construct pillars					
109	Perceptions of the Coach-Athlete Relationship Predict the Attainment of Mastery Achievement Goals Six Months Later: A Two-Wave Longitudinal Study among F. A. Premier League Academy Soccer Players	Nicholls, AR; Earle, K; Earle, F; Madigan, DJ	2017	10.3389/fpsyg.2017.00684	SPORTDiscus+WoS	S3	MAYBE	RETAIN	Potentially eligible; advanced to full-text or extended-abstract assessment	unspecified	P2	TRUE	INCLUDE	Retained in narrative synthesis	Core	Phase I	Direct	9	High
110	How perceived autonomy support and controlling coach behaviors are related to well- and ill-being in elite soccer players: A within-person changes and between-person differences analysis	Cheval, B; Chalabaev, A; Quested, E; Courvoisier, DS; Sarrazin, P	2017	10.1016/j.psychsport.2016.10.006	Scopus+WoS	S3	MAYBE	RETAIN	Potentially eligible; advanced to full-text or extended-abstract assessment	unspecified	P1	TRUE	INCLUDE	Retained in narrative synthesis	Core	Phase II	Direct	9	High
111	Elite Athletes' Experience of Coping With Emotional Abuse in the Coach-Athlete Relationship	Kavanagh, E; Brown, L; Jones, I	2017	10.1080/10413200.2017.1298165	SPORTDiscus+Scopus+WoS	S1+S3	MAYBE	RETAIN	Potentially eligible; advanced to full-text or extended-abstract assessment	unspecified	P2	TRUE	INCLUDE	Retained in narrative synthesis	Core	Relational aftermath	Indirect	7	Moderate
112	Managing player load in professional rugby union: a review of current knowledge and practices	Quarrie, KL; Raftery, M; Blackie, J; Cook, CJ; Fuller, CW; Gabbett, TJ; Gray, AJ; Gill, N; Hennessy, L; Kemp, S; Lambert, M; Nichol, R; Mellalieu, SD; Piscione, J; Stadelmann, J; Tucker, R	2017	10.1136/bjsports-2016-096191	WoS	S3	MAYBE	RETAIN	Potentially eligible; advanced to full-text or extended-abstract assessment	unspecified	P2	TRUE	EXCLUDE_AT_ELIGIBILITY	Did not examine the intersection of at least two construct pillars					
113	The Relationship Between Training Load and Injury in Men's Professional Basketball	Weiss, KJ; Allen, SV; McGuigan, MR; Whatman, CS	2017	10.1123/ijspp.2016-0726	WoS	S3	MAYBE	RETAIN	Potentially eligible; advanced to full-text or extended-abstract assessment	unspecified	P3	FALSE	EXCLUDE_AT_ELIGIBILITY	Did not examine the intersection of at least two construct pillars					
114	Scarred for the Rest of My Career? Career-Long Effects of Abusive Leadership on Professional Athlete Aggression and Task Performance	Carleton, EL; Barling, J; Christie, AM; Trivisonno, M; Tulloch, K; Beauchamp, MR	2016	10.1123/jsep.2015-0333	Scopus+WoS	S1+S3	MAYBE	RETAIN	Potentially eligible; advanced to full-text or extended-abstract assessment	unspecified	P2	FALSE	INCLUDE	Retained in narrative synthesis	Contextual	Relational aftermath	Direct	9	High
115	The Mental Health of Elite Athletes: A Narrative Systematic Review	Rice, SM; Purcell, R; De Silva, S; Mawren, D; McGorry, PD; Parker, AG	2016	10.1007/s40279-016-0492-2	WoS	S1	MAYBE	RETAIN	Potentially eligible; advanced to full-text or extended-abstract assessment	unspecified	P3	FALSE	EXCLUDE_AT_ELIGIBILITY	Did not examine the intersection of at least two construct pillars					
116	Predicting Self-Reported Illness for Professional Team-Sport Athletes	Thornton, HR; Delaney, JA; Duthie, GM; Scott, BR; Chivers, WJ; Sanctuary, CE; Dascombe, BJ	2016	10.1123/ijspp.2015-0330	WoS	S3	MAYBE	RETAIN	Potentially eligible; advanced to full-text or extended-abstract assessment	unspecified	P3	FALSE	EXCLUDE_AT_ELIGIBILITY	Did not examine the intersection of at least two construct pillars					
117	Emotion-centered and action-centered coping in elite sport: Task execution design approach	Hanin, Y; Hanina, M; Sasek, H; Kobilsek, A	2016	10.1177/1747954116654782	WoS	S3	MAYBE	RETAIN	Potentially eligible; advanced to full-text or extended-abstract assessment	unspecified	P3	TRUE	EXCLUDE_AT_ELIGIBILITY	Did not examine the intersection of at least two construct pillars					
118	A Longitudinal Case Study of a Sport Psychology Intervention Designed to Facilitate Team Culture Change	Glaude, M; Alexander-Urquhart, D; Henderson, S; Sheppard, MB; Loughead, TM; Bloom, GA	2026	10.1037/spy0000414	WoS	S1	MAYBE	RETAIN	Potentially eligible; advanced to full-text or extended-abstract assessment	unspecified	P2	TRUE	EXCLUDE_AT_ELIGIBILITY	Elite/professional status not confirmed					
119	Beyond the podium: A Delphi approach aimed at empowering coaches with practical strategies to identify and support athlete mental health	Sankey, C; Brockett, C; Slattery, K; Lederman, O; Warmenhoven, J; Menzies-Stegbauer, T; Langan, E; Caperchione, CM	2026	10.1080/02640414.2025.2576311	Scopus+WoS	S1	MAYBE	RETAIN	Potentially eligible; advanced to full-text or extended-abstract assessment	unspecified	P1, P2	TRUE	EXCLUDE_AT_ELIGIBILITY	Insufficient fit with the temporal cascade after eligibility assessment					
120	Authentic leadership and well-being in sport: the mediating role of psychological safety and the moderating role of interpersonal violence	Hurst, P; Kavussanu, M	2026	10.1080/1612197X.2025.2477163	WoS	S1	MAYBE	RETAIN	Potentially eligible; advanced to full-text or extended-abstract assessment	unspecified	P2	FALSE	EXCLUDE_AT_ELIGIBILITY	Elite/professional status not confirmed					
121	Effects of a six-week basketball-specific individual skills development program on mental toughness and sport competition anxiety	Yazici, A; Tasçioglu, R; Solmaz, S; Sari, I; Kan, EMR; Bayköse, N	2026	10.3389/fpsyg.2026.1794140	WoS	S2+S3	MAYBE	RETAIN	Potentially eligible; advanced to full-text or extended-abstract assessment	unspecified	P3	TRUE	EXCLUDE_AT_ELIGIBILITY	Did not examine the intersection of at least two construct pillars					
122	Digital twin for Taekwondo athletes: integrating sports nutrition and psychological readiness using artificial intelligence	Amawi, AT; Grivas, GV; Alkasasbeh, WJ	2026	10.3389/fpubh.2026.1822194	Scopus+WoS	S2	MAYBE	RETAIN	Potentially eligible; advanced to full-text or extended-abstract assessment	unspecified	P1	FALSE	EXCLUDE_AT_ELIGIBILITY	Did not examine the intersection of at least two construct pillars					
123	Perilous games: Gendered moral disengagement, winning culture, and COVID-19 safety in NCAA division I athletics	Hollis, LP; Oaxaca, GC	2026	10.1177/17479541261445526	WoS	S2	MAYBE	RETAIN	Potentially eligible; advanced to full-text or extended-abstract assessment	unspecified	P3	FALSE	EXCLUDE_AT_ELIGIBILITY	Did not examine the intersection of at least two construct pillars					
124	Sudden cardiac arrest preparedness in African football: evidence from Cameroon and implications for global sport	Tankeng, LT; Akwa, GM; Ossaih, AULS; Bilong, VAN; Tasong, LAM; Ghassi, HT; Ndom, MS; Douryang, M	2026	10.1136/bjsports-2025-110869	WoS	S2	MAYBE	RETAIN	Potentially eligible; advanced to full-text or extended-abstract assessment	unspecified	P1, P3	TRUE	EXCLUDE_AT_ELIGIBILITY	Insufficient fit with the temporal cascade after eligibility assessment					
125	The role of leaders in the communal coping process in sports teams: A social network analysis perspective	Mahot, K; Lienhart, N; Doron, J	2026	10.1080/10413200.2026.2613793	WoS	S2	MAYBE	RETAIN	Potentially eligible; advanced to full-text or extended-abstract assessment	mixed	P3	FALSE	EXCLUDE_AT_ELIGIBILITY	Did not examine the intersection of at least two construct pillars					
126	Implications of perceived empowering motivational climate on athletes' goal-regulatory processes: A longitudinal analysis	Martínez-González, N; Atienza, FL; Balaguer, I	2026	10.1080/02640414.2025.2598159	WoS	S3	MAYBE	RETAIN	Potentially eligible; advanced to full-text or extended-abstract assessment	unspecified	P1, P2	TRUE	EXCLUDE_AT_ELIGIBILITY	Elite/professional status not confirmed					
127	Burnout Syndrome in Sport-Related Jobs: A Meta-analysis of Recent Evidence	Viego, V; Camilo, JAD; Santos, BIA; Lima-Sena, I; Hamodi, C	2026	10.1007/s40279-026-02463-y	WoS	S3	MAYBE	RETAIN	Potentially eligible; advanced to full-text or extended-abstract assessment	unspecified	P2	TRUE	EXCLUDE_AT_ELIGIBILITY	Did not examine the intersection of at least two construct pillars					
128	Development and evaluation of a coach-athlete relationship intervention program	Davis, L; Sommer, M; Brown, DJ; Jowett, S; Ekelund, R; Mårs, T; Wachsmuth, S	2026	10.1016/j.psychsport.2026.103141	Scopus+WoS	S3	MAYBE	RETAIN	Potentially eligible; advanced to full-text or extended-abstract assessment	unspecified	P3	FALSE	INCLUDE	Retained in narrative synthesis	Core	Phase II	Direct	8	High
129	How does need satisfaction mediate the relation between harmonious passion and positive affect in coaches and athletes? A dyadic longitudinal study in coach-athlete dyads	Denul, A; Haerens, L; Loeys, T	2026	10.1177/17479541251366906	WoS	S3	MAYBE	RETAIN	Potentially eligible; advanced to full-text or extended-abstract assessment	unspecified	P3	TRUE	EXCLUDE_AT_ELIGIBILITY	Elite/professional status not confirmed					
130	The relationship between China university basketball association athletes' mindfulness, self-efficacy in sports training, and training competition satisfaction: a cross-sectional and longitudinal study	Zhao, YL; Liu, JW; Yang, XB; Wu, SM	2026	10.1186/s40359-026-04069-5	WoS	S3	MAYBE	RETAIN	Potentially eligible; advanced to full-text or extended-abstract assessment	unspecified	P3	FALSE	EXCLUDE_AT_ELIGIBILITY	Elite/professional status not confirmed					
131	Psychometric properties of the French athlete version of the coach-athlete relationship questionnaire (Fr-CART-Q)	Roux, V; Trouilloud, D	2026	10.1177/17479541251399639	SPORTDiscus+WoS	S3	MAYBE	RETAIN	Potentially eligible; advanced to full-text or extended-abstract assessment	unspecified	P1	TRUE	EXCLUDE_AT_ELIGIBILITY	Elite/professional status not confirmed					
132	Are winning teams more attentive to coach feedback and correction? A longitudinal study across a national-level volleyball season	Malisse, F; Van Puyenbroeck, S; Hofmans, J; Vande Broek, G	2026	10.1080/1612197X.2026.2652876	WoS	S3	MAYBE	RETAIN	Potentially eligible; advanced to full-text or extended-abstract assessment	unspecified	P1	TRUE	EXCLUDE_AT_ELIGIBILITY	Elite/professional status not confirmed					
133	Early Sport Specialization and Intense Training in Junior Tennis Players: A Sport-Specific Review	Thurber, L; Kantrowitz, DE; Wang, KC; Jayanthi, N; Colvin, A	2026	10.1177/19417381251393642	WoS	S3	MAYBE	RETAIN	Potentially eligible; advanced to full-text or extended-abstract assessment	unspecified	P2	TRUE	EXCLUDE_AT_ELIGIBILITY	Did not examine the intersection of at least two construct pillars					
134	Guiding growth: the influence of coach-created climate on passion, athletic identity, and perfectionism in teen female athletes	Martin, EM; Horn, TS	2026	10.1080/1612197X.2025.2481420	WoS	S3	MAYBE	RETAIN	Potentially eligible; advanced to full-text or extended-abstract assessment	unspecified	P1, P3	TRUE	EXCLUDE_AT_ELIGIBILITY	Elite/professional status not confirmed					
135	From social identity to team performance: Linking transactive memory systems and teamwork	Ozlu, C; Sezer, U; Durdubas, D; Koruc, Z	2026	10.1016/j.psychsport.2025.103007	Scopus+WoS	S3	MAYBE	RETAIN	Potentially eligible; advanced to full-text or extended-abstract assessment	mixed	P1	TRUE	EXCLUDE_AT_ELIGIBILITY	Elite/professional status not confirmed					
136	Perceived Coach and Teammate Weight/Shape-Related Beliefs and Disordered Eating in NCAA Swimmers	Pinson, CK; Fox, KR; Frank, GKW; Hooley, JM; Javaras, KN	2026	10.1002/eat.70040	WoS	S3	MAYBE	RETAIN	Potentially eligible; advanced to full-text or extended-abstract assessment	unspecified	P1	FALSE	EXCLUDE_AT_ELIGIBILITY	Did not examine the intersection of at least two construct pillars					
137	The Relationship Between the Somatosensory System and Growth and Maturation in Pathway Triathletes; a Pilot Study	Fitch, AS; Mara, J; Waddington, G	2026	10.1177/00315125261459743	WoS	S3	MAYBE	RETAIN	Potentially eligible; advanced to full-text or extended-abstract assessment	unspecified	P3	FALSE	EXCLUDE_AT_ELIGIBILITY	Did not examine the intersection of at least two construct pillars					
138	Artificial intelligence in sport psychology: Implications for the identification and development of talent	Sartor, F; Rosito, SA; Raimo, M; Beato, M; Bertollo, M	2026	10.1016/j.psychsport.2025.102977	WoS	S3	MAYBE	RETAIN	Potentially eligible; advanced to full-text or extended-abstract assessment	unspecified	P2	TRUE	EXCLUDE_AT_ELIGIBILITY	Elite/professional status not confirmed					
139	Developing Equestrian Athlete Confidence After Experiencing the Setback of a Fall	Collins, A; Smith, MJ	2026	10.1037/spy0000411	WoS	S3	MAYBE	RETAIN	Potentially eligible; advanced to full-text or extended-abstract assessment	unspecified	P2	TRUE	EXCLUDE_AT_ELIGIBILITY	Elite/professional status not confirmed					
140	Crossing the line: Players perspectives on the leap from junior to senior football	Tomás, J; Sousa, H; Araújo, D; Field, A; Ribeiro, J; Sarmento, H	2026	10.1177/17479541261441668	WoS	S3	MAYBE	RETAIN	Potentially eligible; advanced to full-text or extended-abstract assessment	unspecified	P2	FALSE	EXCLUDE_AT_ELIGIBILITY	Did not examine the intersection of at least two construct pillars					
141	Active success drives momentum, but late-stage errors destroy it: a real-time analysis of volleyball	Asai, Y; Okuda, T; Shide, N; Kida, N	2026	10.3389/fpsyg.2026.1839311	WoS	S3	MAYBE	RETAIN	Potentially eligible; advanced to full-text or extended-abstract assessment	unspecified	P3	TRUE	EXCLUDE_AT_ELIGIBILITY	Elite/professional status not confirmed					
142	An evaluation of the hot-hand hypothesis in the NBA when accounting for shot difficulty	Kondur, A; Shen, WN	2026	10.1177/17479541251374788	WoS	S3	MAYBE	RETAIN	Potentially eligible; advanced to full-text or extended-abstract assessment	mixed	P1	FALSE	EXCLUDE_AT_ELIGIBILITY	Elite/professional status not confirmed					
143	Motivational Climate Collaboration Between a Collegiate Native American Volleyball Program and Sport Psychology Researchers	Claunch, JL; Fry, MD; Brown, TC	2026	10.1123/tsp.2025-0028	WoS	S3	MAYBE	RETAIN	Potentially eligible; advanced to full-text or extended-abstract assessment	unspecified	P2	FALSE	EXCLUDE_AT_ELIGIBILITY	Elite/professional status not confirmed					

144	The relationship between coaching leadership practices and athlete burnout among college athletes in China: the mediating role of psychological resilience and emotion regulation.	Chen, Xiaoying ; Yue, Yuan ; Xiang, Jun ; Zhang, Fang	2026	10.1186/s40359-026-04891-x	SPORTDiscus	S3	MAYBE	RETAIN	Potentially eligible; advanced to full-text or extended-abstract assessment	unspecified	P3	FALSE	EXCLUDE_AT_ELIGIBILITY	Elite/professional status not confirmed					
145	The coach as an architect of Danish high- performance sport environments	Henriksen, K; Dideriksen, S; Kuettel, A; Schlawe, A; Storm, LK	2025	10.1016/j.psychsport.2025.102877	Scopus+WoS	S1	MAYBE	RETAIN	Potentially eligible; advanced to full-text or extended-abstract assessment	unspecified	P1	TRUE	INCLUDE	Retained in narrative synthesis	Core	Phase I	Indirect	7	Moderate
146	My biggest learning curve: an interpretive phenomenological analysis of coaches' experiences of working with athletes who experience a menstrual cycle	Jorgensen, H; Davenport, MH; Kavic, K; McHugh, TL	2025	10.1080/2159676X.2025.2495012	WoS	S1	MAYBE	RETAIN	Potentially eligible; advanced to full-text or extended-abstract assessment	unspecified	P1	FALSE	EXCLUDE_AT_ELIGIBILITY	Did not examine the intersection of at least two construct pillars					
147	Impact of competitive anxiety and coach leadership style on track and field athlete performance: moderating role of coping strategies and emotional intelligence	Yue, Y; Zubing, X; Yasmin, F; Poulova, P	2025	10.1007/s12144-024-07125-8	SPORTDiscus+WoS	S1+S2	MAYBE	RETAIN	Potentially eligible; advanced to full-text or extended-abstract assessment	mixed	P2	FALSE	EXCLUDE_AT_ELIGIBILITY	Did not examine the intersection of at least two construct pillars					
148	Achieving international podium success with a positive sport experience	Willson, E; Kerr, G	2025	10.1080/1612197X.2025.2510252	WoS	S1	MAYBE	RETAIN	Potentially eligible; advanced to full-text or extended-abstract assessment	unspecified	P2	FALSE	EXCLUDE_AT_ELIGIBILITY	Did not examine the intersection of at least two construct pillars					
149	Relationship between growth mindset and competitive motivation: a moderated parallel mediation model and feature importance analysis	Deng, JC; Wang, ZP; Chen, HW; Song, B	2025	10.3389/fpsyg.2025.1576649	WoS	S1	MAYBE	RETAIN	Potentially eligible; advanced to full-text or extended-abstract assessment	mixed	P1	FALSE	EXCLUDE_AT_ELIGIBILITY	Did not examine the intersection of at least two construct pillars					
150	Mental well-being in young Taekwondo athletes: psychological-related parameters, associated factors and cluster analysis	Tozetto, WR; Kons, RL; Bussato, E; Felix, G; Ishibashi, M; Milistetd, M	2025	10.1080/1612197X.2025.2547362	WoS	S1	MAYBE	RETAIN	Potentially eligible; advanced to full-text or extended-abstract assessment	unspecified	P1, P2	TRUE	EXCLUDE_AT_ELIGIBILITY	Insufficient fit with the temporal cascade after eligibility assessment					
151	Do coaches' and athletes' perceptions of relative energy deficiency in sport align? A narrative review	Delaney, D; McGuire, A; Warrington, G; Doyle, L	2025	10.1177/17479541251362409	WoS	S1	MAYBE	RETAIN	Potentially eligible; advanced to full-text or extended-abstract assessment	unspecified	P3	FALSE	EXCLUDE_AT_ELIGIBILITY	Did not examine the intersection of at least two construct pillars					
152	Motivation, Sportspersonship, and Gender Equality in a Quidditch Sport Season: Analysis With Mixed Methodology	Méndez-Giménez, A; Collado-Martínez, M	2025	10.1123/jtpe.2024-0250	WoS	S1	MAYBE	RETAIN	Potentially eligible; advanced to full-text or extended-abstract assessment	unspecified	P3	FALSE	EXCLUDE_AT_ELIGIBILITY	Elite/professional status not confirmed					
153	The impact of low energy availability risk on pre-competition physiological function in Chinese female combat athletes	Liang, YH; Li, YX; Chen, Y; Meng, K; Zhou, FY; Pei, YR; Liu, Y; Qiu, JQ	2025	10.1080/15502783.2025.2490170	Scopus+WoS	S2	MAYBE	RETAIN	Potentially eligible; advanced to full-text or extended-abstract assessment	mixed	P2	FALSE	EXCLUDE_AT_ELIGIBILITY	Did not examine the intersection of at least two construct pillars					
154	Differences in the relationship among grit, self-regulation, competition preparation, and sport confidence across performance levels: a multi-group analysis	Shen, YQ; Sim, YK; Bae, J; Bang, IJ; Seo, JP	2025	10.1038/s41598-025-16448-w	Scopus+WoS	S2	MAYBE	RETAIN	Potentially eligible; advanced to full-text or extended-abstract assessment	unspecified	P2	TRUE	EXCLUDE_AT_ELIGIBILITY	Did not examine the intersection of at least two construct pillars					
155	Weight loss practices in Chinese national and international-level Sanda athletes	Zhong, YM; Tang, WW; Gee, T; Li, MF; Jiang, HD; Yin, MY; Xu, K; Lakicevic, N; Drid, P; Kirk, C; Langan-Evans, C; Artioli, GG; Reale, R; Weldon, A; Li, YM	2025	10.1080/15502783.2025.2551216	Scopus+WoS	S2	MAYBE	RETAIN	Potentially eligible; advanced to full-text or extended-abstract assessment	unspecified	P2	TRUE	EXCLUDE_AT_ELIGIBILITY	Did not examine the intersection of at least two construct pillars					
156	Using a Bayesian network to classify time to return to sport based on football injury epidemiological data	Yung, KKY; Wu, PPY; der Fünten, KAD; Hecksteden, A; Meyer, T	2025	10.1371/journal.pone.0314184	Scopus+WoS	S2+S3	MAYBE	RETAIN	Potentially eligible; advanced to full-text or extended-abstract assessment	unspecified	P1	TRUE	EXCLUDE_AT_ELIGIBILITY	Did not examine the intersection of at least two construct pillars					
157	Relative age effect, morphological status and physical performance of U-15 Croatian football players	Rada, A; Marasovic, J; Erceg, M; Krustrup, P; Randers, MB; Ardigò, LP	2025	10.1371/journal.pone.0327878	Scopus+WoS	S2	MAYBE	RETAIN	Potentially eligible; advanced to full-text or extended-abstract assessment	mixed	P3	FALSE	EXCLUDE_AT_ELIGIBILITY	Did not examine the intersection of at least two construct pillars					
158	From adversity to adaptation: the struggle between resilience and athlete burnout in stressful situations	Cai, CY; Mei, ZY; Yang, Y; Luo, S	2025	10.3389/fpsyg.2025.1578198	WoS	S3	MAYBE	RETAIN	Potentially eligible; advanced to full-text or extended-abstract assessment	mixed	P1	FALSE	EXCLUDE_AT_ELIGIBILITY	Elite/professional status not confirmed					
159	The relationship between self-oriented perfectionism and athlete burnout: a longitudinal study	Fu, YT; Li, XY; Sun, JJ; Li, CX; Peng, Y; Hong, FX; Pan, JH	2025	10.3389/fpsyg.2025.1656816	WoS	S3	MAYBE	RETAIN	Potentially eligible; advanced to full-text or extended-abstract assessment	unspecified	P3	FALSE	EXCLUDE_AT_ELIGIBILITY	Elite/professional status not confirmed					
160	A systematic review of the factors associated with athlete burnout in team sports	Woods, S; Dunne, S; Gallagher, P; McNicholl, A	2025	10.1080/1750984X.2022.2148225	WoS	S3	MAYBE	RETAIN	Potentially eligible; advanced to full-text or extended-abstract assessment	unspecified	P1	TRUE	EXCLUDE_AT_ELIGIBILITY	Elite/professional status not confirmed					
161	Coach-athlete attachment and athlete burnout: testing longitudinal mediation via cognitive reappraisal and expressive suppression	Xin, ZY; Li, TE; Li, QL; Chen, YA; Wang, MH	2025	10.1080/1612197X.2024.2389207	SPORTDiscus+WoS	S3	MAYBE	RETAIN	Potentially eligible; advanced to full-text or extended-abstract assessment	mixed	P3	TRUE	EXCLUDE_AT_ELIGIBILITY	Elite/professional status not confirmed					
162	Longitudinal associations between coach-athlete relationship quality and individual injury rate among young handball players: A variable and person-centered approach in intensive training centers	Roux, V; Trouilloud, D	2025	10.1177/17479541251334632	WoS	S3	MAYBE	RETAIN	Potentially eligible; advanced to full-text or extended-abstract assessment	unspecified	P3	FALSE	EXCLUDE_AT_ELIGIBILITY	Elite/professional status not confirmed					
163	Canadian University Sport Coaches' Experiences Participating in a Social Learning Space on Topics Related to and Impacting Coach Mental Health	Henderson, S; Bloom, GA; Durand-Bush, N; Kenttä, G	2025	10.1037/spy0000394	WoS	S3	MAYBE	RETAIN	Potentially eligible; advanced to full-text or extended-abstract assessment	unspecified	P1	FALSE	EXCLUDE_AT_ELIGIBILITY	Elite/professional status not confirmed					
164	The relation between perceived need-supportive coaching behaviours, winning games, and team resilience: a longitudinal examination among basketball and soccer teams	Van Puyenbroeck, S; Hermans, F; Boen, F; Fransen, K; Vande Broek, G	2025	10.1080/1612197X.2024.2366421	WoS	S3	MAYBE	RETAIN	Potentially eligible; advanced to full-text or extended-abstract assessment	mixed	P2	TRUE	EXCLUDE_AT_ELIGIBILITY	Elite/professional status not confirmed					
165	The threshold moderating effect of self-efficacy: a nonlinear transformation mechanism of work passion on job satisfaction among sports practitioners	Wang, KY; Tan, M	2025	10.3389/fpsyg.2025.1667520	WoS	S3	MAYBE	RETAIN	Potentially eligible; advanced to full-text or extended-abstract assessment	unspecified	P1	TRUE	EXCLUDE_AT_ELIGIBILITY	Elite/professional status not confirmed					
166	The motivational climate perceived by young soccer players regarding their coaches, parents, and peers on sport optimal functioning: a cluster analysis	Martínez-González, N; Atienza-González, FL; González-García, L; Balaguer, I	2025	10.3389/fpsyg.2025.1564391	WoS	S3	MAYBE	RETAIN	Potentially eligible; advanced to full-text or extended-abstract assessment	mixed	P1	TRUE	EXCLUDE_AT_ELIGIBILITY	Elite/professional status not confirmed					
167	Resilience and Well-Being Over the Course of an National Collegiate Athletic Association Division I Gymnastics Season	Williams, SM; Baez, SE; Derrico, D; Robinson, H; Coyne, K; Settle, H; Jochimsen, KN	2025	10.1123/jsr.2023-0379	WoS	S3	MAYBE	RETAIN	Potentially eligible; advanced to full-text or extended-abstract assessment	unspecified	P1	FALSE	EXCLUDE_AT_ELIGIBILITY	Did not examine the intersection of at least two construct pillars					
168	Smartphone use and mental health - findings from a 30-day longitudinal study among young talented volleyball players	Hilpisch, C; Zentgraf, K; Mutz, M	2025	10.1080/1612197X.2025.2563317	WoS	S3	MAYBE	RETAIN	Potentially eligible; advanced to full-text or extended-abstract assessment	unspecified	P1	TRUE	EXCLUDE_AT_ELIGIBILITY	Elite/professional status not confirmed					
169	Fostering self-efficacy in Chinese university athletes: the mediating roles of psychological resilience and emotional regulation and the moderating role of autonomy-supportive coaching	Zhang, X; Khan, SU	2025	10.3389/fpsyg.2025.1664339	SPORTDiscus+WoS	S3	MAYBE	RETAIN	Potentially eligible; advanced to full-text or extended-abstract assessment	unspecified	P1	FALSE	EXCLUDE_AT_ELIGIBILITY	Elite/professional status not confirmed					
170	Structural modelling of student volleyball athletes' intimacy, social adjustment, perceived stress, and learning-related anxiety: mediating role of psychological safety	Li, YS	2025	10.1186/s40359-024-02299-z	Scopus+WoS	S3	MAYBE	RETAIN	Potentially eligible; advanced to full-text or extended-abstract assessment	unspecified	P1	TRUE	EXCLUDE_AT_ELIGIBILITY	Elite/professional status not confirmed					
171	Ethnographic research on coaching behaviour to enhance athlete skill learning: A scoping review	Taylor, S; Renshaw, I; Pinder, R; Polman, R; Russell, S; Gorman, AD	2025	10.1177/17479541251350035	WoS	S3	MAYBE	RETAIN	Potentially eligible; advanced to full-text or extended-abstract assessment	unspecified	P3	TRUE	EXCLUDE_AT_ELIGIBILITY	Elite/professional status not confirmed					
172	The Effect of an Educational Session to Improve Knowledge About the Menstrual Cycle and Hormonal Contraceptives in Junior Endurance Athletes and Their Coaches : An Exploratory Investigation	Solli, GS; Topranin, VD; Svantorp-Tveiten, KME; Noordhof, DA	2025	10.1123/ijspp.2025-0068	WoS	S3	EXCLUDE	EXCLUDE	No eligible intersection of coaching/relationship/competitive psychological constructs	elite	none	FALSE	NOT ASSESSED	No eligible intersection of coaching/relationship/competitive psychological constructs					
173	Proactive personality and positional competition: exploring the impact on soccer players' performance and gender differences	Çar, B; Kurtoglu, A; Eken, Ö; Sufaru, C; Ciaccioni, S; Baskaya, G; Panaet, EA; Dragomir, L; Alexe, CI; Bulzan, CO	2025	10.1186/s13102-025-01086-6	WoS	S3	EXCLUDE	EXCLUDE	No eligible intersection of coaching/relationship/competitive psychological constructs	elite	none	TRUE	NOT ASSESSED	No eligible intersection of coaching/relationship/competitive psychological constructs					
174	Implementing pressure on athletes during training: a scoping review	Bouthiller, E; Lautenbach, F	2025	10.1080/1750984X.2025.2578781	WoS	S3	EXCLUDE	EXCLUDE	No eligible intersection of coaching/relationship/competitive psychological constructs	elite	none	FALSE	NOT ASSESSED	No eligible intersection of coaching/relationship/competitive psychological constructs					
175	The Relationship Between Preseason Upper Extremity Function, Pain, and Training and Normalized Division III Collegiate Swimming Performance	Tate, AR; Woznicki, L; Strouse, G; Wisseman, D; Thomas, S	2025	10.4085/1062-6050-0080.24	WoS	S3	EXCLUDE	EXCLUDE	Off-topic: physiological, technical, technological, or non-relational focus	elite	P3	FALSE	NOT ASSESSED	Off-topic: physiological, technical, technological, or non-relational focus					
176	A model of relationships of teammate relations, body image, and eating behaviors among athletes	Runquist, ER III; Cox, M; Meyer, A; Rodgers, RF	2025	10.1016/j.bodyim.2024.101818	WoS	S3	EXCLUDE	EXCLUDE	No eligible intersection of coaching/relationship/competitive psychological constructs	elite	none	FALSE	NOT ASSESSED	No eligible intersection of coaching/relationship/competitive psychological constructs					
177	Evaluating external load responses to cumulative playing time and position in the European Handball Federation Women's Euro 2022 through an IoT and Big Data architecture approach	Karcher, C; Font, R; Marcos-Jorquera, D; Gilart-Iglesias, V; Manchado, C	2025	10.5114/biolsport.2025.144409	WoS	S3	EXCLUDE	EXCLUDE	Off-topic: physiological, technical, technological, or non-relational focus	unspecified	P3	FALSE	NOT ASSESSED	Off-topic: physiological, technical, technological, or non-relational focus					
178	An Examination of the Relationship Between Single Leg Jump and Balance Performance in Female Collegiate Basketball Players	Zahn, D; Willardson, JM	2025	10.1519/JSC.0000000000005112	WoS	S3	EXCLUDE	EXCLUDE	Population not eligible or elite status not established	youth_only	P2	TRUE	NOT ASSESSED	Population not eligible or elite status not established					
179	The story just brings the science to life: exploring the use of stories to translate knowledge about self-regulatory processes during goal striving in running	Jackman, PC; Whitehead, AE; Brick, NE; Wadey, R	2025	10.1080/1612197X.2024.2372404	WoS	S3	EXCLUDE	EXCLUDE	No eligible intersection of coaching/relationship/competitive psychological constructs	elite	none	TRUE	NOT ASSESSED	No eligible intersection of coaching/relationship/competitive psychological constructs					
180	Athletic identity and sport injury: a systematic review and meta-aggregation	Liu, SQ; Noh, YE; Kim, J	2025	10.1186/s40359-025-03902-7	Scopus+WoS	S3	EXCLUDE	EXCLUDE	Population not eligible or elite status not established	youth_only	none	TRUE	NOT ASSESSED	Population not eligible or elite status not established					
181	A Novel Approach for Monitoring Training Load and Wellness in Women's College Gymnastics	Leupold, O; Cheng, J; Wimberly, A; Nguyen, J; Tilley, D; Gabbett, TJ; Casey, E	2025	10.1177/19417381241296855	WoS	S3	EXCLUDE	EXCLUDE	Population not eligible or elite status not established	youth_only	P1	TRUE	NOT ASSESSED	Population not eligible or elite status not established					
182	Examining the Role of Needs Satisfaction in the Coach-Athlete Relationship and in Athletes' Well-Being.	Caling, Jester Vann Y. ; Longakit, Jet C. ; Jariol, Kiana Polline Z. ; Lobo, Joseph ; Rodriguez, Deljun R.	2025	10.2478/pcssr-2025-0010	SPORTDiscus	S1+S3	EXCLUDE	EXCLUDE	Population not eligible or elite status not established	youth_only	P3	TRUE	NOT ASSESSED	Population not eligible or elite status not established					
183	Burnout, Help Seeking, and Perceptions of Psychological Safety and Stigma Among National Collegiate Athletic Association Coaches.	Slowiak, Julie M. ; Osborne, Rebecca R. ; Thomas, Jordyn ; Haasan, Adna	2025	10.1123/iscj.2023-0107	SPORTDiscus	S3	EXCLUDE	EXCLUDE	No eligible intersection of coaching/relationship/competitive psychological constructs	elite	none	TRUE	NOT ASSESSED	No eligible intersection of coaching/relationship/competitive psychological constructs					
184	The processes of developing the coach-athlete relationship: a case study in women's university sport.	Corkery, Erin ; Fletcher, Tim	2025	10.1080/21640629.2023.2256139	SPORTDiscus	S3	EXCLUDE	EXCLUDE	No eligible intersection of coaching/relationship/competitive psychological constructs	elite	none	TRUE	NOT ASSESSED	No eligible intersection of coaching/relationship/competitive psychological constructs					
185	Coaching Psychology in Athletic Bilbao: The Story From Its Beginnings.	O'Neill, John ; Campbell, Mark J. ; Sherwin, Ian	2025	10.1123/iscj.2023-0096	SPORTDiscus	S3	EXCLUDE	EXCLUDE	Population not eligible or elite status not established	youth_only	none	FALSE	NOT ASSESSED	Population not eligible or elite status not established					
186	Systematic review and meta-analysis of Chinese coach leadership and athlete satisfaction and cohesion	Zhu, J; Wang, M; Cruz, AB; Kim, HD	2024	10.3389/fpsyg.2024.1385178	WoS	S1	EXCLUDE	EXCLUDE	Population not eligible or elite status not established	youth_only	P1	FALSE	NOT ASSESSED	Population not eligible or elite status not established					
187	The coach-athlete relationship within a cross-boundary team of experts: a conceptual analysis	Jowett, S	2024	10.1080/1750984X.2024.2416968	WoS	S1	EXCLUDE	EXCLUDE	Population not eligible or elite status not established	youth_only	P1	TRUE	NOT ASSESSED	Population not eligible or elite status not established					

188	The influence of athletes' gratitude on burnout: the sequential mediating roles of the coach-athlete relationship and hope	Dong, LS; Zou, SS; Fan, R; Wang, B; Ye, L	2024	10.3389/fpsyg.2024.1358799	WoS	S1	EXCLUDE	EXCLUDE	Population not eligible or elite status not established	youth_only	P1	TRUE	NOT ASSESSED	Population not eligible or elite status not established					
189	NCAA coaches' perceptions of the inclusion of transgender athletes: a qualitative analysis	Arnold, ML; Chambers, K; Petrie, TA	2024	10.1080/13573322.2023.2283078	Scopus+WoS	S1	EXCLUDE	EXCLUDE	No eligible intersection of coaching/relationship/competitive psychological constructs	unspecified	none	TRUE	NOT ASSESSED	No eligible intersection of coaching/relationship/competitive psychological constructs					
190	Care as a strategy to navigate political tensions within the China-Norway sports coaching collaboration	Tjonndal, A; Haugen, HO	2024	10.1177/10126902241247579	Scopus+WoS	S1	EXCLUDE	RETAIN	Potentially eligible; advanced to full-text or extended-abstract assessment	youth_only	none	FALSE	INCLUDE	Retained in narrative synthesis	Contextual	Cross-cutting	Indirect	7	Moderate
191	Adverse competition-related cognitions questionnaire (ACCQ): Development and preliminary validation of a measure identifying different factors of athletes' cognitions related to competitions	Michel-Kröhler, A; Wessa, M; Berti, S	2024	10.1016/j.psychsport.2023.102582	WoS	S1	EXCLUDE	EXCLUDE	No eligible intersection of coaching/relationship/competitive psychological constructs	unspecified	none	TRUE	NOT ASSESSED	No eligible intersection of coaching/relationship/competitive psychological constructs					
192	Exploring strength and conditioning practitioners' perceptions about using priming exercise as a pre-competition strategy to improve performance	Holmberg, PM; Russell, S; O'Brien, KA; James, LP; Kelly, VG	2024	10.1177/17479541231207951	WoS	S2	EXCLUDE	EXCLUDE	Population not eligible or elite status not established	youth_only	none	TRUE	NOT ASSESSED	Population not eligible or elite status not established					
193	The effect of post-activation enhancement on the performance of Chinese national skeleton athletes in the ice push sled-a first cross-sectional study	Tian, G; Li, HJ; Zhu, H; Gao, BH	2024	10.7717/peerj.18271	Scopus+WoS	S2	EXCLUDE	EXCLUDE	No eligible intersection of coaching/relationship/competitive psychological constructs	unspecified	none	FALSE	NOT ASSESSED	No eligible intersection of coaching/relationship/competitive psychological constructs					
194	Psychological stress and psychological well-being among sports coaches: A close proximity longitudinal daily diary study	Potts, AJ; Didymus, FF; Kaiseler, M	2024	10.1080/10413200.2024.2344847	Scopus+WoS	S3	EXCLUDE	EXCLUDE	Off-topic: physiological, technical, technological, or non-relational focus	unspecified	none	TRUE	NOT ASSESSED	Off-topic: physiological, technical, technological, or non-relational focus					
195	Reciprocal relationships between teamwork execution, emergent states, and perceived team performance: a longitudinal study	López-Gajardo, MA; Leo, FM; Díaz-García, J; McEwan, D	2024	10.1080/1612197X.2023.2263863	WoS	S3	EXCLUDE	EXCLUDE	No eligible intersection of coaching/relationship/competitive psychological constructs	unspecified	none	TRUE	NOT ASSESSED	No eligible intersection of coaching/relationship/competitive psychological constructs					
196	Changes in Sports Participation, Specialization, and Burnout From 7th to 12th Grade: Final Results From a 6-Year Longitudinal Study	Valenzuela-Moss, J; Sini, M; Wren, TAL; Edison, BR	2024	10.1177/19417381231224792	WoS	S3	EXCLUDE	EXCLUDE	Population not eligible or elite status not established	youth_only	none	FALSE	NOT ASSESSED	Population not eligible or elite status not established					
197	Organizational stress and well-being in competitive sport: a systematic review	Simpson, RAC; Didymus, FF; Williams, TL	2024	10.1080/1750984X.2021.1975305	WoS	S3	EXCLUDE	EXCLUDE	No eligible intersection of coaching/relationship/competitive psychological constructs	unspecified	none	TRUE	NOT ASSESSED	No eligible intersection of coaching/relationship/competitive psychological constructs					
198	Factors influencing sport persistence still represent a knowledge gap - the experience of a systematic review	Kovács, KE; Szakál, Z	2024	10.1186/s40359-024-02098-6	Scopus+WoS	S3	EXCLUDE	EXCLUDE	Population not eligible or elite status not established	youth_only	P1, P3	FALSE	NOT ASSESSED	Population not eligible or elite status not established					
199	The mediating roles of pre-competitive coping and affective states in the relationships between coach-athlete relationship, satisfaction and attainment of achievement goals	González-García, H; Martinent, G; Nicolas, M	2024	10.1080/1612197X.2023.2190346	SPORTDiscus+WoS	S3	EXCLUDE	RETAIN	Potentially eligible; advanced to full-text or extended-abstract assessment	unspecified	none	FALSE	INCLUDE	Retained in narrative synthesis	Core	Phase III	Direct	8	High
200	Validation of the Coach Behavior in Sports Questionnaire: Towards dynamic assessments using the circumplex model for coach behavior	Van Meervelt, K; Reynders, B; Van Puyenbroeck, S; De Backer, M; Hofmans, J; Vande Broek, G	2024	10.1016/j.psychsport.2024.102691	WoS	S3	EXCLUDE	EXCLUDE	Population not eligible or elite status not established	youth_only	P1	TRUE	NOT ASSESSED	Population not eligible or elite status not established					
201	A Longitudinal Multicase Study About the Board-Game Format of an Educational Self-Talk Intervention	Mathas, V; Solomon-Turay, P; Hatzigeorgiadis, A; Hardy, J; Latinjak, AT	2024	10.1123/tsp.2023-0150	Scopus+WoS	S3	EXCLUDE	EXCLUDE	No eligible intersection of coaching/relationship/competitive psychological constructs	elite	none	FALSE	NOT ASSESSED	No eligible intersection of coaching/relationship/competitive psychological constructs					
202	Initiatives to facilitate the social inclusion of people with intellectual disability in physical activities with others in community-based sporting, recreation and leisure activities: A scoping review	Ristevski, E; McCook, F; Thompson, S; Duncan, Z; Lawler, B; Broxham, C	2024	10.1111/jar.13238	Scopus+WoS	S3	EXCLUDE	EXCLUDE	No eligible intersection of coaching/relationship/competitive psychological constructs	elite	none	FALSE	NOT ASSESSED	No eligible intersection of coaching/relationship/competitive psychological constructs					
203	Changes in Body Composition and Athletic Performance in National Collegiate Athletic Association Division I Female Field Hockey Athletes Throughout a Competitive Season	Summer, LC; Cheng, R; Moran, JT; Lee, M; Belanger, AJ; Taylor, WL IV; Gardner, EC	2024	10.1519/JSC.0000000000004591	WoS	S3	EXCLUDE	EXCLUDE	No eligible intersection of coaching/relationship/competitive psychological constructs	elite	none	FALSE	NOT ASSESSED	No eligible intersection of coaching/relationship/competitive psychological constructs					
204	Association between concussion understanding and stakeholder knowledge translation in collegiate sports	Giguere, D; Barnes, M; Faught, BE	2024	10.1177/17479541231224273	WoS	S3	EXCLUDE	EXCLUDE	No eligible intersection of coaching/relationship/competitive psychological constructs	elite	none	FALSE	NOT ASSESSED	No eligible intersection of coaching/relationship/competitive psychological constructs					
205	The effects of mobile phone dependence on athletic performance and its mechanisms	Mei, ZY; Zhang, YZ; Fan, Q; Luo, SL; Luo, S	2024	10.3389/fpsyg.2024.1391258	WoS	S3	EXCLUDE	EXCLUDE	No eligible intersection of coaching/relationship/competitive psychological constructs	unspecified	none	TRUE	NOT ASSESSED	No eligible intersection of coaching/relationship/competitive psychological constructs					
206	College Football Kids: Infantilizing Language in Football Bowl Subdivision Bowl Game Broadcasts	Corr, C; Southall, C; Hawkins, B; Southall, RM	2024	10.1123/ssj.2022-0185	WoS	S3	EXCLUDE	EXCLUDE	Off-topic: physiological, technical, technological, or non-relational focus	unspecified	P3	FALSE	NOT ASSESSED	Off-topic: physiological, technical, technological, or non-relational focus					
207	A framework for mental training in sport: Enhancing mental skills, wellbeing, and performance	Vealey, RS	2024	10.1080/10413200.2023.2274459	Scopus+WoS	S3	EXCLUDE	EXCLUDE	Population not eligible or elite status not established	youth_only	P3	FALSE	NOT ASSESSED	Population not eligible or elite status not established					
208	A review of the interpersonal experience, expression, and regulation of emotions in sport	Tamminen, K; Wolf, SA; Dunn, R; Bissett, JE	2024	10.1080/1750984X.2022.2132526	WoS	S3	EXCLUDE	EXCLUDE	Population not eligible or elite status not established	youth_only	P3	FALSE	NOT ASSESSED	Population not eligible or elite status not established					
209	The Relationship Between Mental Fatigue and Shooting Performance Over the Course of a National Collegiate Athletic Association Division I Basketball Season	Daub, BD; Mclean, BD; Heishman, AD; Peak, KM; Coutts, AJ	2024	10.1519/JSC.0000000000004624	WoS	S3	EXCLUDE	EXCLUDE	No eligible intersection of coaching/relationship/competitive psychological constructs	unspecified	none	TRUE	NOT ASSESSED	No eligible intersection of coaching/relationship/competitive psychological constructs					
210	Tracking relations between development of tactical knowledge and tactical behaviour: a season-long action research study	Ramos, A; Davids, K; Coutinho, P; Mesquita, I	2024	10.1080/17408989.2022.2083099	WoS	S3	EXCLUDE	EXCLUDE	No eligible intersection of coaching/relationship/competitive psychological constructs	elite	none	TRUE	NOT ASSESSED	No eligible intersection of coaching/relationship/competitive psychological constructs					
211	Making the Invisible, Visible: An Exploration of Track-and-Field Coaches' Perspectives of Their Planning Processes	Johns, KL; Potrac, P; Hayes, PR	2024	10.1123/ijspp.2023-0467	WoS	S3	EXCLUDE	EXCLUDE	No eligible intersection of coaching/relationship/competitive psychological constructs	unspecified	none	TRUE	NOT ASSESSED	No eligible intersection of coaching/relationship/competitive psychological constructs					
212	Optimization of Sprint Training Among European Coaches: Quality Over Quantity	Agudo-Ortega, A; Sandbakk, O; Salinero, JJ; Johansen, B; González-Rave, JM	2024	10.1123/ijspp.2024-0042	WoS	S3	EXCLUDE	EXCLUDE	Population not eligible or elite status not established	youth_only	P1	FALSE	NOT ASSESSED	Population not eligible or elite status not established					
213	THE INFLUENCE OF CLIMATE ON OUTDOOR ATHLETIC TRAINING REGIMES IN NORWAY	Ostgaard, N	2024	10.15366/rimcafd2024.95.022	WoS	S3	EXCLUDE	EXCLUDE	Population not eligible or elite status not established	youth_only	P3	TRUE	NOT ASSESSED	Population not eligible or elite status not established					
214	Research note: Evaluating the efficacy of an online post event reflection tool in competitive sport	Bruner, MW; Mclaren, CD; Turcotte, D; Marshall, C	2024	10.1080/10413200.2023.2296902	WoS	S3	EXCLUDE	EXCLUDE	No eligible intersection of coaching/relationship/competitive psychological constructs	elite	none	TRUE	NOT ASSESSED	No eligible intersection of coaching/relationship/competitive psychological constructs					
215	A Systematic Review with a Meta-Analysis of the Motivational Climate and Hedonic Well-Being Constructs: The Importance of the Athlete Level.	Lochbaum, Marc ; Sisneros, Cassandra	2024	10.3390/ejihpe14040064	SPORTDiscus	S1	EXCLUDE	EXCLUDE	Population not eligible or elite status not established	youth_only	none	TRUE	NOT ASSESSED	Population not eligible or elite status not established					
216	Examining the Impact of Coach Leadership Styles on Athlete Motivation in Collegiate Athletics.	Awan, Shah Hussain ; Khan, Awais Alam ; Zulkifal, Syed ; Ullah, Abrar	2024		SPORTDiscus	S3	EXCLUDE	EXCLUDE	No eligible intersection of coaching/relationship/competitive psychological constructs	elite	none	TRUE	NOT ASSESSED	No eligible intersection of coaching/relationship/competitive psychological constructs					
217	The Gold Medal Profile for Sport Psychology (GMP-SP)	Durand-Bush, N; Baker, J; Van den Berg, F; Richard, V; Bloom, GA	2023	10.1080/10413200.2022.2055224	WoS	S1	EXCLUDE	EXCLUDE	Population not eligible or elite status not established	youth_only	none	TRUE	NOT ASSESSED	Population not eligible or elite status not established					
218	THE COACH-ATHLETE RELATIONSHIP: PSYCHOMETRIC PROPERTIES OF THE SPANISH VERSION OF THE CART-Q	Sanz-Fernández, C; Alonso-Arbiol, I; Pérez-Albéniz, A	2023	10.15366/rimcafd2022.89.013	WoS	S1	EXCLUDE	EXCLUDE	Population not eligible or elite status not established	youth_only	P1, P3	TRUE	NOT ASSESSED	Population not eligible or elite status not established					
219	Can't jump, won't jump: Affordances of the horse-rider dyad underpin skill adaptation in showjumping using a constraints-led approach	Davies, M; Stone, JA; Davids, K; Williams, J; O'Sullivan, M	2023	10.1177/17479541221107379	WoS	S1	EXCLUDE	EXCLUDE	Population not eligible or elite status not established	youth_only	none	TRUE	NOT ASSESSED	Population not eligible or elite status not established					
220	A Qualitative Exploration of Coaches' Perceptions of Performance Under Pressure in Sport	Hufton, JR; Vella, SA; Schweickle, MJ	2023	10.1037/spy0000324	WoS	S2+S3	EXCLUDE	EXCLUDE	No eligible intersection of coaching/relationship/competitive psychological constructs	unspecified	none	FALSE	NOT ASSESSED	No eligible intersection of coaching/relationship/competitive psychological constructs					
221	Narcissistic coaches and athletes' individual rowing performance	Nevicka, B; van Gerven, EJG; Sedikides, C	2023	10.1038/s41598-023-48239-6	Scopus+WoS	S2	EXCLUDE	EXCLUDE	No eligible intersection of coaching/relationship/competitive psychological constructs	unspecified	none	TRUE	NOT ASSESSED	No eligible intersection of coaching/relationship/competitive psychological constructs					
222	A randomized controlled study of mindful sport performance enhancement and psychological skills training with collegiate track and field athletes	Hut, M; Minkler, TO; Glass, CR; Weppner, CH; Thomas, HM; Flannery, CB	2023	10.1080/10413200.2021.1989521	Scopus+WoS	S2	EXCLUDE	EXCLUDE	No eligible intersection of coaching/relationship/competitive psychological constructs	unspecified	none	TRUE	NOT ASSESSED	No eligible intersection of coaching/relationship/competitive psychological constructs					
223	Moderators of the coach leadership and athlete motivation relationship	de Subijana, CL; Martin, LJ; McGuire, CS; Côté, J	2023	10.1080/17461391.2022.2041101	SPORTDiscus+WoS	S3	EXCLUDE	EXCLUDE	No eligible intersection of coaching/relationship/competitive psychological constructs	unspecified	none	TRUE	NOT ASSESSED	No eligible intersection of coaching/relationship/competitive psychological constructs					
224	A longitudinal investigation of the role of perceived autonomy support from coaches in reducing athletes' experiential avoidance: The mediating role of subjective vitality	Wang, Y; Wu, CH; Chen, LH	2023	10.1016/j.psychsport.2022.102304	Scopus+WoS	S3	EXCLUDE	EXCLUDE	No eligible intersection of coaching/relationship/competitive psychological constructs	unspecified	none	FALSE	NOT ASSESSED	No eligible intersection of coaching/relationship/competitive psychological constructs					
225	The mediator role of communication skill in the relationship between empathy, team cohesion, and competition performance in curlers	Bedir, D; Agduman, F; Bedir, F; Erhan, SE	2023	10.3389/fpsyg.2023.1115402	WoS	S3	EXCLUDE	EXCLUDE	No eligible intersection of coaching/relationship/competitive psychological constructs	unspecified	none	TRUE	NOT ASSESSED	No eligible intersection of coaching/relationship/competitive psychological constructs					
226	Application of a nutrition support protocol to encourage optimisation of nutrient intake in provincial academy rugby union athletes in New Zealand: Practical considerations and challenges from a team-based case study	Roberts, CJ; Gill, ND; Beaven, CM; Posthumus, LR; Sims, ST	2023	10.1177/17479541221124119	WoS	S3	EXCLUDE	EXCLUDE	No eligible intersection of coaching/relationship/competitive psychological constructs	unspecified	none	FALSE	NOT ASSESSED	No eligible intersection of coaching/relationship/competitive psychological constructs					
227	Understanding the Role of Self-Efficacy in Sports Performance: A Longitudinal Study	Yang, XK; Mojtahe, K	2023		WoS	S3	EXCLUDE	EXCLUDE	No eligible intersection of coaching/relationship/competitive psychological constructs	unspecified	none	TRUE	NOT ASSESSED	No eligible intersection of coaching/relationship/competitive psychological constructs					
228	Noncontact Injury Distribution and Relationship With Preseason Training Load and Nonmodifiable Risk Factors in Rugby Union Players Across Multiple Seasons	Evans, SL; Whittaker, G; Davis, OE; Jones, ES; Hardy, J; Owen, JA	2023	10.1519/JSC.0000000000004402	WoS	S3	EXCLUDE	EXCLUDE	No eligible intersection of coaching/relationship/competitive psychological constructs	unspecified	none	TRUE	NOT ASSESSED	No eligible intersection of coaching/relationship/competitive psychological constructs					
229	Assessing the Use of Heart-Rate Monitoring for Competitive Swimmers	Sixsmith, H; Crowcroft, S; Slattery, K	2023	10.1123/ijspp.2023-0009	WoS	S3	EXCLUDE	EXCLUDE	No eligible intersection of coaching/relationship/competitive psychological constructs	elite	none	FALSE	NOT ASSESSED	No eligible intersection of coaching/relationship/competitive psychological constructs					

230	Monitoring mood state to improve performance in soccer players: A brief review	Selmi, O; Ouergui, I; Muscella, A; Levitt, DE; Suzuki, K; Bouassida, A	2023	10.3389/fpsyg.2023.1095238	WoS	S3	EXCLUDE	EXCLUDE	Population not eligible or elite status not established	youth_only	none	FALSE	NOT ASSESSED	Population not eligible or elite status not established					
231	Relationship Between Athlete-Reported Outcome Measures and Subsequent Match Performance in Team Sports: A Systematic Review	Sansone, P; Rago, V; Kellmann, M; Alcaraz, PE	2023	10.1519/JSC.0000000000004605	WoS	S3	EXCLUDE	EXCLUDE	No eligible intersection of coaching/relationship/competitive psychological constructs	elite	none	FALSE	NOT ASSESSED	No eligible intersection of coaching/relationship/competitive psychological constructs					
232	Research on Optimization of Post-Competition Training Methods for Competitive Sports Athletes	Xia, CY; Lei, XM	2023		WoS	S3	EXCLUDE	EXCLUDE	Off-topic: physiological, technical, technological, or non-relational focus	elite	P2	TRUE	NOT ASSESSED	Off-topic: physiological, technical, technological, or non-relational focus					
233	Precompetition Strategies to Improve Performance in Endurance or Repeated Intermittent Activity: Evidence and Practical Suggestions	Valcarce-Merayo, E; Latella, C	2023	10.1519/SSC.0000000000000753	WoS	S3	EXCLUDE	EXCLUDE	No eligible intersection of coaching/relationship/competitive psychological constructs	unspecified	none	TRUE	NOT ASSESSED	No eligible intersection of coaching/relationship/competitive psychological constructs					
234	Using Physiological Laboratory Tests and Neuromuscular Functions to Predict Extreme Ultratriathlon Performance	Abad, CCC; Carmo, EC; Do Prado, DML; Reis, V; Pereira, L; Loturco, I; Koehle, M	2023	10.1080/02701367.2022.2094307	WoS	S3	EXCLUDE	EXCLUDE	No eligible intersection of coaching/relationship/competitive psychological constructs	elite	none	TRUE	NOT ASSESSED	No eligible intersection of coaching/relationship/competitive psychological constructs					
235	The Psychology of Athletic Tapering in Sport: A Scoping Review	Stone, MJ; Knight, CJ; Hall, R; Shearer, C; Nicholas, R; Shearer, DA	2023	10.1007/s40279-022-01798-6	WoS	S3	EXCLUDE	EXCLUDE	No eligible intersection of coaching/relationship/competitive psychological constructs	elite	none	FALSE	NOT ASSESSED	No eligible intersection of coaching/relationship/competitive psychological constructs					
236	Secondary School Athletic Coaches' Perceptions and Knowledge of the Athletic Training Profession	Lacy, AMP; Stearns, RL; Eason, CM; Casa, DJ	2023	10.4085/1062-6050-0369.21	WoS	S3	EXCLUDE	EXCLUDE	No eligible intersection of coaching/relationship/competitive psychological constructs	elite	none	FALSE	NOT ASSESSED	No eligible intersection of coaching/relationship/competitive psychological constructs					
237	Understanding mental toughness at a Women?s Australian rules football club	Clark, JD; Mallett, CJ; Moyle, GM; Coulter, TJ	2023	10.1016/j.psychsport.2023.102432	Scopus+WoS	S3	EXCLUDE	EXCLUDE	No eligible intersection of coaching/relationship/competitive psychological constructs	elite	none	FALSE	NOT ASSESSED	No eligible intersection of coaching/relationship/competitive psychological constructs					
238	The effects of coach autonomy support, sports mental training, experiential avoidance, and sports anxiety among badminton college athletes.	CHUAN-CHEN HUANG	2023	10.7752/jpes.2023.09289	SPORTDiscus	S1	EXCLUDE	EXCLUDE	No eligible intersection of coaching/relationship/competitive psychological constructs	elite	none	FALSE	NOT ASSESSED	No eligible intersection of coaching/relationship/competitive psychological constructs					
239	Effect of boxers' social support on mental fatigue: Chain mediating effects of coach leadership behaviors and psychological resilience.	Zhang, Defa ; Lyu, Bei ; Wu, Jingtao ; Li, Wangze ; Zhang, Kaiyuan	2023	10.3233/WOR-220478	SPORTDiscus+Scopus	S1	EXCLUDE	EXCLUDE	No eligible intersection of coaching/relationship/competitive psychological constructs	unspecified	none	FALSE	NOT ASSESSED	No eligible intersection of coaching/relationship/competitive psychological constructs					
240	Features and Effects of Athlete Burnout Among Top Amateur Female Rugby Union Players	Martin, K; Hong, HJ	2022	10.1123/tsp.2022-0034	WoS	S1	EXCLUDE	EXCLUDE	Off-topic: physiological, technical, technological, or non-relational focus	elite	P3	FALSE	NOT ASSESSED	Off-topic: physiological, technical, technological, or non-relational focus					
241	Relationships between coach's leadership, group cohesion, affective states, sport satisfaction and goal attainment in competitive settings	González-García, H; Martinent, G; Nicolas, M	2022	10.1177/17479541211053229	WoS	S1	EXCLUDE	EXCLUDE	Population not eligible or elite status not established	youth_only	P1	TRUE	NOT ASSESSED	Population not eligible or elite status not established					
242	Understanding Sport Expertise through a Cultural Perspective: An Exploratory Study	Castillo, EA; Lebeau, JC; Cho, S; Sáenz-Moncaleano, C; Chow, GM	2022	10.7352/IJSP.2021.52.099	WoS	S1	EXCLUDE	EXCLUDE	Population not eligible or elite status not established	youth_only	P1	TRUE	NOT ASSESSED	Population not eligible or elite status not established					
243	Body shaming and associated practices as abuse: athlete entourage as perpetrators of abuse	McMahon, J; McGannon, KR; Palmer, C	2022	10.1080/13573322.2021.1890571	WoS	S1	EXCLUDE	EXCLUDE	No eligible intersection of coaching/relationship/competitive psychological constructs	unspecified	none	TRUE	NOT ASSESSED	No eligible intersection of coaching/relationship/competitive psychological constructs					
244	Perceived motivational climate influences athletes' emotion regulation strategies, emotions, and psychobiosocial experiences	Robazza, C; Moran, M; Bortoli, L; Ruiz, MC	2022	10.1016/j.psychsport.2021.102110	Scopus+WoS	S1	EXCLUDE	RETAIN	Potentially eligible; advanced to full-text or extended-abstract assessment	unspecified	none	TRUE	INCLUDE	Retained in narrative synthesis	Core	Phase II	Indirect	6	Moderate
245	Don't think, just shoot - The paradox of shooting three-point shots in basketball	Lidor, R; Lipshits, L; Arnon, M; Bar-Eli, M	2022	10.1080/1612197X.2021.1987957	WoS	S2	EXCLUDE	EXCLUDE	Population not eligible or elite status not established	youth_only	none	TRUE	NOT ASSESSED	Population not eligible or elite status not established					
246	Upper-Body Post-activation Performance Enhancement for Athletic Performance: A Systematic Review with Meta-analysis and Recommendations for Future Research	Finlay, MJ; Bridge, CA; Greig, M; Page, RM	2022	10.1007/s40279-021-01598-4	WoS	S2	EXCLUDE	EXCLUDE	No eligible intersection of coaching/relationship/competitive psychological constructs	unspecified	none	TRUE	NOT ASSESSED	No eligible intersection of coaching/relationship/competitive psychological constructs					
247	Autonomy Support in Sport Contexts: The Role of Parents and Coaches in the Promotion of Athlete Well-Being and Performance	Lemelin, E; Verner-Filion, J; Carpentier, J; Carbonneau, N; Mageau, GA	2022	10.1037/spy0000287	WoS	S3	EXCLUDE	EXCLUDE	No eligible intersection of coaching/relationship/competitive psychological constructs	unspecified	none	TRUE	NOT ASSESSED	No eligible intersection of coaching/relationship/competitive psychological constructs					
248	Unlocking the power of 'us': Longitudinal evidence that identity leadership predicts team functioning and athlete well-being	Fransen, K; Boen, F; Haslam, SA; McLaren, CD; Mertens, N; Steffens, NK; Bruner, MW	2022	10.1080/02640414.2023.2193005	WoS	S3	EXCLUDE	EXCLUDE	Population not eligible or elite status not established	youth_only	P3	TRUE	NOT ASSESSED	Population not eligible or elite status not established					
249	Six-month stability of individual differences in sports coaches' burnout, self-compassion and social support	Ackeret, N; Röthlin, P; Allemand, M; Krieger, T; Berger, T; Znoj, H; Klenttä, G; Birrer, D; Horvath, S	2022	10.1016/j.psychsport.2022.102207	WoS	S3	EXCLUDE	EXCLUDE	No eligible intersection of coaching/relationship/competitive psychological constructs	unspecified	none	FALSE	NOT ASSESSED	No eligible intersection of coaching/relationship/competitive psychological constructs					
250	A Prospective Study of Teammate Factors on Athletes' Well-Being, Disordered Eating, and Compulsive Exercise	Scott, CL; Haycraft, E; Plateau, CR	2022	10.1037/spy0000293	WoS	S3	EXCLUDE	EXCLUDE	No eligible intersection of coaching/relationship/competitive psychological constructs	unspecified	none	TRUE	NOT ASSESSED	No eligible intersection of coaching/relationship/competitive psychological constructs					
251	A longitudinal study on the psychological and physiological predictors of burnout in NCAA collegiate swimmers	Martin, J; Byrd, B; Hew-Butler, T; Moore, EWG	2022	10.1080/10413200.2021.1974603	WoS	S3	EXCLUDE	EXCLUDE	No eligible intersection of coaching/relationship/competitive psychological constructs	elite	none	TRUE	NOT ASSESSED	No eligible intersection of coaching/relationship/competitive psychological constructs					
252	Performance Profiling in Strength and Conditioning	Johnson, DL; Bird, MD	2022	10.1519/SSC.0000000000000684	WoS	S3	EXCLUDE	EXCLUDE	No eligible intersection of coaching/relationship/competitive psychological constructs	unspecified	none	TRUE	NOT ASSESSED	No eligible intersection of coaching/relationship/competitive psychological constructs					
253	Athletes' motivational and emotional outcomes related to a Need-Supportive intervention in intensive training centers	Cece, V; Guillet-Descas, E; Tessier, D; Martinent, G	2022	10.1080/10413200.2021.1941425	Scopus+WoS	S3	EXCLUDE	RETAIN	Potentially eligible; advanced to full-text or extended-abstract assessment	elite	none	TRUE	INCLUDE	Retained in narrative synthesis	Core	Phase II	Direct	9	High
254	Implementation and evaluation of a standardized performance profile intervention with collegiate athletes: A comparison of the original and revised techniques	Castillo, EA; Bird, MD; Chow, GM	2022	10.1080/10413200.2020.1862358	WoS	S3	EXCLUDE	EXCLUDE	No eligible intersection of coaching/relationship/competitive psychological constructs	elite	none	FALSE	NOT ASSESSED	No eligible intersection of coaching/relationship/competitive psychological constructs					
255	Exploring the Relationship Between Athletes' Perceptions of Their Team Motivational Climate and Their Sport Shame	Fontana, MS; Fry, MD; Moore, EWG	2022	10.1123/tsp.2021-0145	Scopus+WoS	S3	EXCLUDE	EXCLUDE	No eligible intersection of coaching/relationship/competitive psychological constructs	mixed	none	TRUE	NOT ASSESSED	No eligible intersection of coaching/relationship/competitive psychological constructs					
256	A Coach Development Program: A Guided Online Reflective Practice Intervention Study	Da Silva, EJ; Mallett, CJ; Sánchez-Oliva, D; Dias, A; Palmeira, A	2022	10.1080/02640414.2022.2045795	WoS	S3	EXCLUDE	EXCLUDE	No eligible intersection of coaching/relationship/competitive psychological constructs	unspecified	none	TRUE	NOT ASSESSED	No eligible intersection of coaching/relationship/competitive psychological constructs					
257	Longitudinal trajectories of emotions among athletes in sports competitions: Does emotional intelligence matter?	Levillain, G; Martinent, G; Vacher, P; Nicolas, M	2022	10.1016/j.psychsport.2021.102012	Scopus+WoS	S3	EXCLUDE	RETAIN	Potentially eligible; advanced to full-text or extended-abstract assessment	unspecified	none	FALSE	INCLUDE	Retained in narrative synthesis	Contextual	Phases II–III	Direct	7	Moderate
258	The effects of self-compassion on daily emotion regulation and performance rebound among college athletes: Comparisons with confidence, grit, and hope	Doorley, JD; Kashdan, TB; Weppner, CH; Glass, CR	2022	10.1016/j.psychsport.2021.102081	Scopus+WoS	S3	EXCLUDE	EXCLUDE	Population not eligible or elite status not established	youth_only	none	TRUE	NOT ASSESSED	Population not eligible or elite status not established					
259	A shame of inches: Are teams with black head coaches more heavily penalized in Division 1 college football?	Davis, AP; Leppard, TR; Kinney, AB	2022	10.1111/ssqu.13117	WoS	S3	EXCLUDE	EXCLUDE	No eligible intersection of coaching/relationship/competitive psychological constructs	elite	none	FALSE	NOT ASSESSED	No eligible intersection of coaching/relationship/competitive psychological constructs					
260	Understanding the sleep of ultra-marathon swimmers: Guidance for coaches and swimmers	Dunican, IC; Perry, EL; Gemma, M; Nesci, E; Roberts, S	2022	10.1177/17479541221089385	WoS	S3	EXCLUDE	EXCLUDE	No eligible intersection of coaching/relationship/competitive psychological constructs	unspecified	none	FALSE	NOT ASSESSED	No eligible intersection of coaching/relationship/competitive psychological constructs					
261	Transcultural Adaptation and Psychometric Support for a Brazilian Portuguese Version of the Flow State Scale (FSS-2)	Garcia, WF; Nascimento, JRA Jr; Mizoguchi, MV; Brandao, MRF; Fiorese, L	2022	10.1177/00315125221093917	Scopus+WoS	S3	EXCLUDE	EXCLUDE	No eligible intersection of coaching/relationship/competitive psychological constructs	elite	none	FALSE	NOT ASSESSED	No eligible intersection of coaching/relationship/competitive psychological constructs					
262	The impact of verbal and physical abuse on distress, mental health, and intentions to quit in sports officials	Brick, NE; Breslin, G; Shevlin, M; Shannon, S	2022	10.1016/j.psychsport.2022.102274	WoS	S3	EXCLUDE	EXCLUDE	No eligible intersection of coaching/relationship/competitive psychological constructs	unspecified	none	FALSE	NOT ASSESSED	No eligible intersection of coaching/relationship/competitive psychological constructs					
263	A Coach-Delivered Dating Violence Prevention Program: Coach Perspectives on Implementation	Fields, AD; Fenn, M; Ripper, L; Hill, A; Mulbah, P; Kass, NM; Miller, E	2022	10.1177/08862605221092073	Scopus+WoS	S3	EXCLUDE	EXCLUDE	No eligible intersection of coaching/relationship/competitive psychological constructs	unspecified	none	FALSE	NOT ASSESSED	No eligible intersection of coaching/relationship/competitive psychological constructs					
264	The role of the anaerobic speed reserve in female middle-distance running	Hallam, LC; Ducharme, JB; Mang, ZA; Amorim, FT	2022	10.1016/j.scispo.2021.07.006	WoS	S3	EXCLUDE	EXCLUDE	No eligible intersection of coaching/relationship/competitive psychological constructs	elite	none	FALSE	NOT ASSESSED	No eligible intersection of coaching/relationship/competitive psychological constructs					
265	Effectiveness of the Activate injury prevention exercise programme to prevent injury in schoolboy rugby union	Barden, C; Hancock, M; Stokes, KA; Roberts, SP; McKay, CD	2022	10.1136/bjsports-2021-105170	WoS	S3	EXCLUDE	EXCLUDE	No eligible intersection of coaching/relationship/competitive psychological constructs	elite	none	FALSE	NOT ASSESSED	No eligible intersection of coaching/relationship/competitive psychological constructs					
266	The Effects of an Exploratory Mindfulness Intervention on Collegiate Performance	Trajkovski, M; Newl, A	2022	10.1123/tsp.2021-0088	Scopus+WoS	S3	EXCLUDE	EXCLUDE	No eligible intersection of coaching/relationship/competitive psychological constructs	elite	none	TRUE	NOT ASSESSED	No eligible intersection of coaching/relationship/competitive psychological constructs					
267	Mental training in the opinion of the Polish national karate kyokushin team	Piepiora, P; Baginska, J; Jaskowska, K; Witkowski, K	2022	10.2154/matarts.8754	WoS	S3	EXCLUDE	EXCLUDE	Population not eligible or elite status not established	youth_only	none	TRUE	NOT ASSESSED	Population not eligible or elite status not established					
268	Winning the second half: The perceived and actual impact of the coach's half-time speech on basketball players' performance	Zach, S; Avugos, S; Bakalo-Kuffler, L; Bar-Eli, M	2022	10.1177/17479541221089743	WoS	S3	EXCLUDE	EXCLUDE	Population not eligible or elite status not established	youth_only	P1	TRUE	NOT ASSESSED	Population not eligible or elite status not established					
269	Why Does Teamwork Execution Breakdown? Experiences of University Team Sport Athletes	McEwan, D; Crawford, KL	2022	10.1037/spy0000290	WoS	S3	EXCLUDE	EXCLUDE	Off-topic: physiological, technical, technological, or non-relational focus	unspecified	none	FALSE	NOT ASSESSED	Off-topic: physiological, technical, technological, or non-relational focus					
270	Athletes' perspectives of preparation strategies in open-skill sports	Bonk, D; Tamminen, KA	2022	10.1080/10413200.2021.1875517	Scopus+WoS	S3	EXCLUDE	EXCLUDE	No eligible intersection of coaching/relationship/competitive psychological constructs	unspecified	none	TRUE	NOT ASSESSED	No eligible intersection of coaching/relationship/competitive psychological constructs					
271	Examining links between coaching behaviors and collegiate athletes' burnout levels using a longitudinal approach.	Mellano, Kathleen T. ; Horn, Thelma S. ; Mann, Mallory	2022	10.1016/j.psychsport.2022.102189	SPORTDiscus+Scopus	S3	EXCLUDE	EXCLUDE	No eligible intersection of coaching/relationship/competitive psychological constructs	unspecified	none	TRUE	NOT ASSESSED	No eligible intersection of coaching/relationship/competitive psychological constructs					
272	Resolving uncertainties of the factor structures of the Coach-Athlete Relationship Questionnaire (CART-Q)	Woolliams, D; Spencer, K; Walters, S; Krägeloh, C	2021	10.1080/00049530.2021.1882275	WoS	S1	EXCLUDE	EXCLUDE	No eligible intersection of coaching/relationship/competitive psychological constructs	unspecified	none	TRUE	NOT ASSESSED	No eligible intersection of coaching/relationship/competitive psychological constructs					
273	The Personal and Interpersonal Components of Perfectionism: The Italian Validation of Multidimensional Inventory of Perfectionism in Sport	De Maria, A; Mallia, L; Lombardo, C; Vacca, M; Zelli, A	2021	10.3390/ijerph18052657	Scopus+WoS	S2	EXCLUDE	EXCLUDE	Population not eligible or elite status not established	youth_only	none	TRUE	NOT ASSESSED	Population not eligible or elite status not established					

274	Stressors in Indoor and Field Brazilian Soccer: Are They Perceived as a Distress or Eustress?	Brandao, MRF; Polito, LF; Hernandes, V; Correa, M; Mastrocola, AP; Oliveira, D; Oliveira, A; Moura, L; Boas, MV Jr; Angelo, D	2021	10.3389/fpsyg.2021.623719	WoS	S2	EXCLUDE	EXCLUDE	No eligible intersection of coaching/relationship/competitive psychological constructs	unspecified	none	TRUE	NOT ASSESSED	No eligible intersection of coaching/relationship/competitive psychological constructs					
275	Mindfulness training for a college team: Feasibility, acceptability, and effectiveness from within an athletic department	Minkler, TO; Glass, CR; Hut, M	2021	10.1080/10413200.2020.1739169	Scopus+WoS	S2	EXCLUDE	EXCLUDE	No eligible intersection of coaching/relationship/competitive psychological constructs	unspecified	none	FALSE	NOT ASSESSED	No eligible intersection of coaching/relationship/competitive psychological constructs					
276	Research on Pre-Competition Mood State of Sports Athletes and Their Individual Differences	Wang, ML	2021		WoS	S2	EXCLUDE	EXCLUDE	No eligible intersection of coaching/relationship/competitive psychological constructs	unspecified	none	FALSE	NOT ASSESSED	No eligible intersection of coaching/relationship/competitive psychological constructs					
277	A longitudinal examination of thriving in sport performers	Brown, DJ; Arnold, R; Standage, M; Fletcher, D	2021	10.1016/j.psychsport.2021.101934	Scopus+WoS	S3	EXCLUDE	EXCLUDE	No eligible intersection of coaching/relationship/competitive psychological constructs	elite	none	TRUE	NOT ASSESSED	No eligible intersection of coaching/relationship/competitive psychological constructs					
278	Inhibitory Control Across Athletic Expertise and Its Relationship With Sport Performance	Hagyard, J; Brimmell, J; Edwards, EJ; Vaughan, RS	2021	10.1123/jsep.2020-0043	WoS	S3	EXCLUDE	EXCLUDE	No eligible intersection of coaching/relationship/competitive psychological constructs	elite	none	FALSE	NOT ASSESSED	No eligible intersection of coaching/relationship/competitive psychological constructs					
279	Extraversion in sport: a scoping review	Allen, MS; Mison, EA; Robson, DA; Laborde, S	2021	10.1080/1750984X.2020.1790024	WoS	S3	EXCLUDE	EXCLUDE	No eligible intersection of coaching/relationship/competitive psychological constructs	unspecified	none	FALSE	NOT ASSESSED	No eligible intersection of coaching/relationship/competitive psychological constructs					
280	A pilot study investigating cortical haemodynamic and physiological correlates of exercise cognition in trained and untrained cyclists over an incremental self-paced performance test, while thinking aloud	Robinson, NJ; Montgomery, C; Swettenham, L; Whitehead, A	2021	10.1016/j.psychsport.2021.101912	WoS	S3	EXCLUDE	EXCLUDE	Population not eligible or elite status not established	youth_only	none	FALSE	NOT ASSESSED	Population not eligible or elite status not established					
281	Role of psychological ownership and authenticity strength in the relationship between authentic leadership and sport team performance: A multilevel analysis	Grégoire, B; Laure, G; Sarah, M; Séverine, C; Evelyne, F	2021	10.1016/j.psychsport.2021.101965	Scopus+WoS	S3	EXCLUDE	EXCLUDE	Population not eligible or elite status not established	youth_only	P1	TRUE	NOT ASSESSED	Population not eligible or elite status not established					
282	Methodological intervention with soccer coaches to improve athlete-perceived coaching competency, satisfaction with the coach, enjoyment and intention to persist	Pulido, JJ; Leo, FM; González-Ponce, I; López-Gajardo, MA; Sánchez-Oliva, D	2021	10.1177/1747954120952069	WoS	S3	EXCLUDE	EXCLUDE	No eligible intersection of coaching/relationship/competitive psychological constructs	elite	none	FALSE	NOT ASSESSED	No eligible intersection of coaching/relationship/competitive psychological constructs					
283	It's not just about football all the time either: transnational athletes' stories about the choice to migrate	Ely, G; Ronkainen, NJ	2021	10.1080/1612197X.2019.1637364	WoS	S3	EXCLUDE	EXCLUDE	No eligible intersection of coaching/relationship/competitive psychological constructs	elite	none	FALSE	NOT ASSESSED	No eligible intersection of coaching/relationship/competitive psychological constructs					
284	Follow-up evaluation of the Coaching for Life Skills online training program	Turgeon, S; Camiré, M; Rathwell, S	2021	10.1177/1747954120964075	WoS	S3	EXCLUDE	EXCLUDE	No eligible intersection of coaching/relationship/competitive psychological constructs	elite	none	FALSE	NOT ASSESSED	No eligible intersection of coaching/relationship/competitive psychological constructs					
285	Consent, capacity and compliance in concussion management: cave ergo medicus (let the doctor beware)	Turner, M; Maddocks, D; Hassan, M; Anderson, A; McCrory, P	2021	10.1136/bjsports-2020-102108	WoS	S3	EXCLUDE	EXCLUDE	No eligible intersection of coaching/relationship/competitive psychological constructs	elite	none	FALSE	NOT ASSESSED	No eligible intersection of coaching/relationship/competitive psychological constructs					
286	Acceptance and Commitment Training for Ice Hockey Players: A Randomized Controlled Trial	Lundgren, T; Reinebo, G; Fröjmark, MJ; Jäder, E; Näslund, M; Svartvadet, P; Samuelsson, U; Parling, T	2021	10.3389/fpsyg.2021.685260	WoS	S3	EXCLUDE	EXCLUDE	Off-topic: physiological, technical, technological, or non-relational focus	elite	P3	FALSE	NOT ASSESSED	Off-topic: physiological, technical, technological, or non-relational focus					
287	Harnessing the power of 'us': A randomized wait-list controlled trial of the 5R shared leadership development program (5Rs) in basketball teams	Mertens, N; Boen, F; Steffens, NK; Haslam, SA; Bruner, M; Barker, JB; Slater, MJ; Fransen, K	2021	10.1016/j.psychsport.2021.101936	WoS	S3	EXCLUDE	EXCLUDE	No eligible intersection of coaching/relationship/competitive psychological constructs	unspecified	none	FALSE	NOT ASSESSED	No eligible intersection of coaching/relationship/competitive psychological constructs					
288	Perceived Coach-Created Motivational Climates as Predictors of Athletes' Goal Reengagement: The Mediational Role of Goal Motives	Martinez-Gonzalez, N; Atienza, FL; Tomas, I; Balaguer, I	2021	10.3389/fpsyg.2021.740060	WoS	S3	EXCLUDE	EXCLUDE	No eligible intersection of coaching/relationship/competitive psychological constructs	unspecified	none	TRUE	NOT ASSESSED	No eligible intersection of coaching/relationship/competitive psychological constructs					
289	Health Conditions, Substance Use, Physical Activity, and Quality of Life in Current and Former Baseball Players	Bullock, GS; Nicholson, KF; Waterman, BR; Niesen, E; Salamh, P; Thigpen, CA; Shanley, E; Devaney, L; Collins, GS; Arden, NK; Filbay, SR	2021	10.1177/23259671211056645	WoS	S3	EXCLUDE	EXCLUDE	Population not eligible or elite status not established	youth_only	none	TRUE	NOT ASSESSED	Population not eligible or elite status not established					
290	An exploratory investigation into the reception of verbal and video feedback provided to players in an Australian Football League club	Mason, RJ; Farrow, D; Hattie, JAC	2021	10.1177/1747954120951080	WoS	S3	EXCLUDE	EXCLUDE	No eligible intersection of coaching/relationship/competitive psychological constructs	unspecified	none	TRUE	NOT ASSESSED	No eligible intersection of coaching/relationship/competitive psychological constructs					
291	Home Team (Dis)Advantage Patterns in the National Hockey League: Changes Through Increased Emphasis on Individual Performance With the 3-on-3 Overtime Rule	Hoffmann, MD; McEwan, D; Baumeister, RF; Barnes, JD; Guerrero, MD	2021	10.1177/0031512520966138	Scopus+WoS	S3	EXCLUDE	EXCLUDE	No eligible intersection of coaching/relationship/competitive psychological constructs	elite	none	TRUE	NOT ASSESSED	No eligible intersection of coaching/relationship/competitive psychological constructs					
292	Dyadic coping in coach-athlete relationships: A grounded theory	Staff, HR; Didymus, FF; Backhouse, SH	2020	10.1016/j.psychsport.2020.101741	WoS	S1	EXCLUDE	EXCLUDE	No eligible intersection of coaching/relationship/competitive psychological constructs	elite	none	TRUE	NOT ASSESSED	No eligible intersection of coaching/relationship/competitive psychological constructs					
293	'I was excited to train, so I didn't have problems with the coach': dual career athletes' experiences of (dis)empowering motivational climates	Saarinen, M; Ryba, T; Ronkainen, NJ; Rintala, H; Aunola, K	2020	10.1080/17430437.2019.1669322	Scopus+WoS	S1	EXCLUDE	EXCLUDE	No eligible intersection of coaching/relationship/competitive psychological constructs	unspecified	none	TRUE	NOT ASSESSED	No eligible intersection of coaching/relationship/competitive psychological constructs					
294	Effects of Motivational Climate on Fear of Failure and Anxiety in Teen Handball Players	Gómez-López, M; Chicau Borrego, C; da Silva, CM; Granero-Gallegos, A; González-Hernández, J	2020	10.3390/ijerph17020592	WoS	S2	EXCLUDE	EXCLUDE	No eligible intersection of coaching/relationship/competitive psychological constructs	unspecified	none	TRUE	NOT ASSESSED	No eligible intersection of coaching/relationship/competitive psychological constructs					
295	Heart rate variability and pre-competitive anxiety according to the demanding level of the match in female soccer athletes	Ayuso-Moreno, R; Fuentes-García, JP; Collado-Mateo, D; Villafaina, S	2020	10.1016/j.physbeh.2020.112926	Scopus+WoS	S2	EXCLUDE	EXCLUDE	No eligible intersection of coaching/relationship/competitive psychological constructs	unspecified	none	TRUE	NOT ASSESSED	No eligible intersection of coaching/relationship/competitive psychological constructs					
296	Methods and Approaches of College Counselors to Deal with Students' Psychological Crisis	Guan, RX; Zhang, JB	2020		WoS	S2	EXCLUDE	EXCLUDE	No eligible intersection of coaching/relationship/competitive psychological constructs	unspecified	none	TRUE	NOT ASSESSED	No eligible intersection of coaching/relationship/competitive psychological constructs					
297	Psychometric Properties of the Psychological State Test for Athletes (TEP)	Díaz-Tendero, P; Pérez-Llantada, MC; de la Llave, AL	2020	10.3389/fpsyg.2020.566828	WoS	S2	EXCLUDE	EXCLUDE	No eligible intersection of coaching/relationship/competitive psychological constructs	unspecified	none	TRUE	NOT ASSESSED	No eligible intersection of coaching/relationship/competitive psychological constructs					
298	Performance Trajectories for Competitive Swimmers: The Role of Coach Interpersonal Behaviors and Athlete Motivation	Rocchi, MA; Guertin, C; Pelletier, LG; Sweet, SN	2020	10.1037/mot0000156	WoS	S3	EXCLUDE	EXCLUDE	No eligible intersection of coaching/relationship/competitive psychological constructs	unspecified	none	FALSE	NOT ASSESSED	No eligible intersection of coaching/relationship/competitive psychological constructs					
299	Mental Blocks in Artistic Gymnastics and Cheerleading: Longitudinal Analysis of Flikikammo	Maaranen, A; Van Raalte, JL; Brewer, BW	2020	10.1123/jcsp.2018-0101	WoS	S3	EXCLUDE	EXCLUDE	No eligible intersection of coaching/relationship/competitive psychological constructs	elite	none	FALSE	NOT ASSESSED	No eligible intersection of coaching/relationship/competitive psychological constructs					
300	Performance indicators during international rugby union matches are influenced by a combination of physiological and contextual variables	Crewther, BT; Potts, N; Kilduff, LP; Drawer, S; Cook, CJ	2020	10.1016/j.jsams.2019.10.011	WoS	S3	EXCLUDE	EXCLUDE	No eligible intersection of coaching/relationship/competitive psychological constructs	unspecified	none	FALSE	NOT ASSESSED	No eligible intersection of coaching/relationship/competitive psychological constructs					
301	Psychological characteristics, sport engagement, and performance in alpine skiers	Fawver, B; Cowan, RL; DeCouto, BS; Lohse, KR; Podlog, L; Williams, AM	2020	10.1016/j.psychsport.2019.101616	Scopus+WoS	S3	EXCLUDE	EXCLUDE	No eligible intersection of coaching/relationship/competitive psychological constructs	unspecified	none	TRUE	NOT ASSESSED	No eligible intersection of coaching/relationship/competitive psychological constructs					
302	Understanding social networks and social support resources with sports coaches	Norris, LA; Didymus, FF; Kaiseler, M	2020	10.1016/j.psychsport.2020.101665	Scopus+WoS	S3	EXCLUDE	EXCLUDE	No eligible intersection of coaching/relationship/competitive psychological constructs	unspecified	none	FALSE	NOT ASSESSED	No eligible intersection of coaching/relationship/competitive psychological constructs					
303	The 2020 Coronavirus Pandemic as a Change-Event in Sport Performers' Careers: Conceptual and Applied Practice Considerations	Samuel, RD; Tenenbaum, G; Galily, Y	2020	10.3389/fpsyg.2020.567966	WoS	S3	EXCLUDE	EXCLUDE	No eligible intersection of coaching/relationship/competitive psychological constructs	unspecified	none	FALSE	NOT ASSESSED	No eligible intersection of coaching/relationship/competitive psychological constructs					
304	Individual Pathways Through the Junior-to-Senior Transition: Narratives of Two Swedish Team Sport Athletes	Franck, A; Stambulova, NB	2020	10.1080/10413200.2018.1525625	Scopus+WoS	S3	EXCLUDE	EXCLUDE	No eligible intersection of coaching/relationship/competitive psychological constructs	unspecified	none	FALSE	NOT ASSESSED	No eligible intersection of coaching/relationship/competitive psychological constructs					
305	Psychosocial experiences of concussed collegiate athletes: The role of emotional support in the recovery process	Wayment, HA; Huffman, AH	2020	10.1080/07448481.2019.1577863	WoS	S3	EXCLUDE	EXCLUDE	No eligible intersection of coaching/relationship/competitive psychological constructs	elite	none	FALSE	NOT ASSESSED	No eligible intersection of coaching/relationship/competitive psychological constructs					
306	Coach identity leadership behaviours are positively associated with athlete resource appraisals: The mediating roles of relational and group identification	Miller, AJ; Slater, MJ; Turner, MJ	2020	10.1016/j.psychsport.2020.101755	Scopus+WoS	S3	EXCLUDE	RETAIN	Potentially eligible; advanced to full-text or extended-abstract assessment	elite	P1	FALSE	INCLUDE	Retained in narrative synthesis	Core	Phase III	Direct	10	High
307	Relationship Between Power Output and Speed-Related Performance in Brazilian Wheelchair Basketball Players	Loturco, I; McGuigan, MR; Reis, VP; Santos, S; Yanci, J; Pereira, LA; Winckler, C	2020	10.1123/apaq.2019-0158	WoS	S3	EXCLUDE	EXCLUDE	No eligible intersection of coaching/relationship/competitive psychological constructs	elite	none	FALSE	NOT ASSESSED	No eligible intersection of coaching/relationship/competitive psychological constructs					
308	Perceived Coach Leadership Profiles and Relationship With Burnout, Coping, and Emotions	González-García, H; Martinent, G; Morales, AT	2019	10.3389/fpsyg.2019.01785	WoS	S1	EXCLUDE	EXCLUDE	Population not eligible or elite status not established	youth_only	none	TRUE	NOT ASSESSED	Population not eligible or elite status not established					
309	Working alliance in the coach-athlete relationship and athlete burnout The mediating role of athlete resilience	Moen, F; Hrozanova, M; Stiles, T; Stenseng, F	2019	10.7352/IJSP.2019.50.338	WoS	S1	EXCLUDE	EXCLUDE	Off-topic: physiological, technical, technological, or non-relational focus	elite	none	TRUE	NOT ASSESSED	Off-topic: physiological, technical, technological, or non-relational focus					
310	Development of the Sport Mental Health Continuum-Short Form (Sport MHC-SF)	Foster, BJ; Chow, GM	2019	10.1123/jcsp.2017-0057	WoS	S1	EXCLUDE	EXCLUDE	No eligible intersection of coaching/relationship/competitive psychological constructs	unspecified	none	TRUE	NOT ASSESSED	No eligible intersection of coaching/relationship/competitive psychological constructs					
311	Emotional Intelligence (EI) Training Adapted to the International Preparation Constraints in Rugby: Influence of EI Trainer Status on EI Training Effectiveness	Campo, M; Laborde, S; Martinent, G; Louvet, B; Nicolas, M	2019	10.3389/fpsyg.2019.01939	WoS	S1+S3	EXCLUDE	EXCLUDE	No eligible intersection of coaching/relationship/competitive psychological constructs	unspecified	none	TRUE	NOT ASSESSED	No eligible intersection of coaching/relationship/competitive psychological constructs					
312	A practitioner's perspective on psychological issues in football	Heidari, J; Pelka, M; Beckmann, J; Kellmann, M	2019	10.1080/24733938.2018.1526397	WoS	S2	EXCLUDE	EXCLUDE	Population not eligible or elite status not established	youth_only	P1	FALSE	NOT ASSESSED	Population not eligible or elite status not established					
313	The Coach-Athlete Relationship and Athlete Psychological Outcomes	McGee, V; Defreese, JD	2019	10.1123/jcsp.2018-0010	SPORTDiscus+WoS	S3	EXCLUDE	EXCLUDE	No eligible intersection of coaching/relationship/competitive psychological constructs	elite	none	TRUE	NOT ASSESSED	No eligible intersection of coaching/relationship/competitive psychological constructs					
314	Communication Strategies: The Fuel for Quality Coach-Athlete Relationships and Athlete Satisfaction	Davis, L; Jowett, S; Tafvelin, S	2019	10.3389/fpsyg.2019.02156	WoS	S3	EXCLUDE	EXCLUDE	No eligible intersection of coaching/relationship/competitive psychological constructs	unspecified	none	TRUE	NOT ASSESSED	No eligible intersection of coaching/relationship/competitive psychological constructs					
315	Development of Perfectionism in Junior Athletes: A Three-Sample Study of Coach and Parental Pressure	Madigan, DJ; Curran, T; Stoeber, J; Hill, AP; Smith, MM; Passfield, L	2019	10.1123/jsep.2018-0287	Scopus+WoS	S3	EXCLUDE	EXCLUDE	Population not eligible or elite status not established	youth_only	none	TRUE	NOT ASSESSED	Population not eligible or elite status not established					

316	Coach-Created Motivational Climate and Athletes' Adaptation to Psychological Stress: Temporal Motivation-Emotion Interplay	Ruiz, MC; Robazza, C; Tolvanen, A; Haapanen, S; Duda, JL	2019	10.3389/fpsyg.2019.00617	SPORTDiscus+WoS	S3	EXCLUDE	EXCLUDE	No eligible intersection of coaching/relationship/competitive psychological constructs	unspecified	none	TRUE	NOT ASSESSED	No eligible intersection of coaching/relationship/competitive psychological constructs					
317	Coaches' Emotional Intelligence and Reactive Behaviors in Soccer Matches: Mediating Effects of Coach Efficacy Beliefs	Teques, P; Duarte, D; Viana, J	2019	10.3389/fpsyg.2019.01629	WoS	S3	EXCLUDE	EXCLUDE	No eligible intersection of coaching/relationship/competitive psychological constructs	elite	none	TRUE	NOT ASSESSED	No eligible intersection of coaching/relationship/competitive psychological constructs					
318	Dual Career of the U-23 Spanish Canoeing Team	Gavala-González, J; Castillo-Rodríguez, A; Fernández-García, JC	2019	10.3389/fpsyg.2019.01783	WoS	S3	EXCLUDE	EXCLUDE	Off-topic: physiological, technical, technological, or non-relational focus	youth_only	none	FALSE	NOT ASSESSED	Off-topic: physiological, technical, technological, or non-relational focus					
319	Psychometric properties of the Brazilian version of the Coach-Athlete Relationship Questionnaire for coaches	Contreira, AR; do Nascimento, JRA; Pizzo, GC; Sitoe, SA; Moreira, CR; Jowett, S; Fiorese, L	2019	10.1177/1747954119832715	SPORTDiscus+WoS	S3	EXCLUDE	EXCLUDE	No eligible intersection of coaching/relationship/competitive psychological constructs	unspecified	none	FALSE	NOT ASSESSED	No eligible intersection of coaching/relationship/competitive psychological constructs					
320	The roles of coaches, peers, and parents in athletes' basic psychological needs: A mixed-studies review	Chu, TL; Zhang, T	2019	10.1177/1747954119858458	SPORTDiscus+WoS	S3	EXCLUDE	EXCLUDE	No eligible intersection of coaching/relationship/competitive psychological constructs	elite	none	TRUE	NOT ASSESSED	No eligible intersection of coaching/relationship/competitive psychological constructs					
321	The junior to senior transition: a narrative analysis of the pathways of two Swedish athletes	Franck, A; Stambulova, NB	2019	10.1080/2159676X.2018.1479979	WoS	S3	EXCLUDE	EXCLUDE	No eligible intersection of coaching/relationship/competitive psychological constructs	unspecified	none	FALSE	NOT ASSESSED	No eligible intersection of coaching/relationship/competitive psychological constructs					
322	The Temporal Relationship Between Exercise, Recovery Processes, and Changes in Performance	Skorski, S; Mujika, I; Bosquet, L; Meeusen, R; Coutts, AJ; Meyer, T	2019	10.1123/ijspp.2018-0668	WoS	S3	EXCLUDE	EXCLUDE	No eligible intersection of coaching/relationship/competitive psychological constructs	elite	none	FALSE	NOT ASSESSED	No eligible intersection of coaching/relationship/competitive psychological constructs					
323	RELATIONSHIP BETWEEN RUNNING ECONOMY AND KINEMATIC PARAMETERS IN LONG-DISTANCE RUNNERS	Pizzuto, F; de Oliveira, CF; Soares, TSA; Rago, V; Silva, G; Oliveira, J	2019	10.1519/JSC.0000000000003040	WoS	S3	EXCLUDE	EXCLUDE	Population not eligible or elite status not established	youth_only	P1, P2	FALSE	NOT ASSESSED	Population not eligible or elite status not established					
324	Identity salience and the football self: a critical ethnographic study of women and girls in football	Pielichaty, H	2019	10.1080/2159676X.2018.1549094	WoS	S3	EXCLUDE	EXCLUDE	No eligible intersection of coaching/relationship/competitive psychological constructs	unspecified	none	FALSE	NOT ASSESSED	No eligible intersection of coaching/relationship/competitive psychological constructs					
325	When You Watch Your Team Fall Apart - Coaches' and Sport Psychologists' Perceptions on Causes of Collective Sport Team Collapse	Wergin, VV; Mallett, CJ; Mesagno, C; Zimanyi, Z; Beckmann, J	2019	10.3389/fpsyg.2019.01331	WoS	S3	EXCLUDE	EXCLUDE	Population not eligible or elite status not established	youth_only	none	TRUE	NOT ASSESSED	Population not eligible or elite status not established					
326	Self-regulatory skills: Are they helpful in the prevention of overuse injuries in talented tennis players?	van der Sluis, A; Brink, MS; Pluim, BM; Verhagen, EALM; Elferink-Gemser, MT; Visscher, C	2019	10.1111/sms.13420	WoS	S3	EXCLUDE	EXCLUDE	Population not eligible or elite status not established	youth_only	none	FALSE	NOT ASSESSED	Population not eligible or elite status not established					
327	Relationship between total hemoglobin mass and competitive performance in endurance athletes	Zelenkova, IE; Zotkin, SV; Korneev, PV; Koprov, SV; Grushin, AA	2019	10.23736/S0022-4707.18.07865-9	WoS	S3	EXCLUDE	EXCLUDE	Population not eligible or elite status not established	youth_only	P1, P3	TRUE	NOT ASSESSED	Population not eligible or elite status not established					
328	Coaches' interpersonal emotion regulation and the coach-athlete relationship.	Campo, Mickaël ; Braun, Courtney ; Tamminen, Katherine A.	2019	10.1051/sm/2019011	SPORTDiscus	S3	EXCLUDE	EXCLUDE	No eligible intersection of coaching/relationship/competitive psychological constructs	unspecified	none	TRUE	NOT ASSESSED	No eligible intersection of coaching/relationship/competitive psychological constructs					
329	The development of a tournament preparation framework for competitive golf: A Delphi study	Pilgrim, J; Kremer, P; Robertson, S	2018	10.1080/17461391.2018.1469673	Scopus+WoS	S2	EXCLUDE	EXCLUDE	No eligible intersection of coaching/relationship/competitive psychological constructs	unspecified	none	FALSE	NOT ASSESSED	No eligible intersection of coaching/relationship/competitive psychological constructs					
330	Organizational stressors, social support, and implications for subjective performance in high-level sport	Arnold, R; Edwards, T; Rees, T	2018	10.1016/j.psychsport.2018.08.010	WoS	S2	EXCLUDE	EXCLUDE	No eligible intersection of coaching/relationship/competitive psychological constructs	unspecified	none	FALSE	NOT ASSESSED	No eligible intersection of coaching/relationship/competitive psychological constructs					
331	Emotional Intelligence, Motivational Climate and Levels of Anxiety in Athletes from Different Categories of Sports: Analysis through Structural Equations	Castro-Sánchez, M; Zurita-Ortega, F; Chacón-Cuberos, R; López-Gutiérrez, CJ; Zafra-Santos, E	2018	10.3390/ijerph15050894	Scopus+WoS	S2	EXCLUDE	EXCLUDE	No eligible intersection of coaching/relationship/competitive psychological constructs	unspecified	none	FALSE	NOT ASSESSED	No eligible intersection of coaching/relationship/competitive psychological constructs					
332	Athletes' perceptions of coaching competency and team conflict in sport teams: A multilevel analysis	González-Ponce, I; Leo, FM; Jiménez, R; Sánchez-Oliva, D; Sarmento, H; Figueiredo, A; García-Calvo, T	2018	10.1080/17461391.2018.1461245	WoS	S3	EXCLUDE	EXCLUDE	No eligible intersection of coaching/relationship/competitive psychological constructs	unspecified	none	FALSE	NOT ASSESSED	No eligible intersection of coaching/relationship/competitive psychological constructs					
333	Monitoring Training Load and Well-Being During the In-Season Phase in National Collegiate Athletic Association Division I Men's Basketball	Conte, D; Kolb, N; Scanlan, AT; Santolamazza, F	2018	10.1123/ijspp.2017-0689	WoS	S3	EXCLUDE	EXCLUDE	No eligible intersection of coaching/relationship/competitive psychological constructs	elite	none	FALSE	NOT ASSESSED	No eligible intersection of coaching/relationship/competitive psychological constructs					
334	Self-Talk and Competitive Balance Beam Performance	Van Dyke, ED; Van Raalte, JL; Mullin, EM; Brewer, BW	2018	10.1123/tsp.2016-0085	Scopus+WoS	S3	EXCLUDE	EXCLUDE	Population not eligible or elite status not established	youth_only	none	FALSE	NOT ASSESSED	Population not eligible or elite status not established					
335	Neophyte experiences of football (soccer) match analysis: a multiple case study approach	McKenna, M; Cowan, DT; Stevenson, D; Baker, JS	2018	10.1080/15438627.2018.1447473	WoS	S3	EXCLUDE	EXCLUDE	No eligible intersection of coaching/relationship/competitive psychological constructs	elite	none	FALSE	NOT ASSESSED	No eligible intersection of coaching/relationship/competitive psychological constructs					
336	Talent Identification and Development in Male Football: A Systematic Review	Sarmento, H; Anguera, MT; Pereira, A; Araújo, D	2018	10.1007/s40279-017-0851-7	WoS	S3	EXCLUDE	EXCLUDE	No eligible intersection of coaching/relationship/competitive psychological constructs	unspecified	none	TRUE	NOT ASSESSED	No eligible intersection of coaching/relationship/competitive psychological constructs					
337	When Suddenly Nothing Works Anymore Within a Team - Causes of Collective Sport Team Collapse	Wergin, VV; Zimanyi, Z; Mesagno, C; Beckmann, J	2018	10.3389/fpsyg.2018.02115	WoS	S3	EXCLUDE	EXCLUDE	No eligible intersection of coaching/relationship/competitive psychological constructs	elite	none	TRUE	NOT ASSESSED	No eligible intersection of coaching/relationship/competitive psychological constructs					
338	An Integrated, Multifactorial Approach to Periodization for Optimal Performance in Individual and Team Sports	Mujika, I; Halson, S; Burke, LM; Balagué, G; Farrow, D	2018	10.1123/ijspp.2018-0093	WoS	S3	EXCLUDE	EXCLUDE	Population not eligible or elite status not established	youth_only	none	TRUE	NOT ASSESSED	Population not eligible or elite status not established					
339	Leveling the playing field: can psychological coping resources reduce the influence of physical and technical skills on athletic performance?	Christensen, DS; Smith, RE	2018	10.1080/10615806.2018.1506646	Scopus+WoS	S3	EXCLUDE	EXCLUDE	Population not eligible or elite status not established	youth_only	none	TRUE	NOT ASSESSED	Population not eligible or elite status not established					
340	A test of basic psychological needs theory in young soccer players: time-lagged design at the individual and team levels	González, L; Tomás, I; Castillo, I; Duda, JL; Balaguer, I	2017	10.1111/sms.12778	WoS	S3	EXCLUDE	EXCLUDE	No eligible intersection of coaching/relationship/competitive psychological constructs	unspecified	none	TRUE	NOT ASSESSED	No eligible intersection of coaching/relationship/competitive psychological constructs					
341	Stressors, coping, and well-being among sports coaches: A systematic review	Norris, LA; Didymus, FF; Kaiseler, M	2017	10.1016/j.psychsport.2017.08.005	Scopus+WoS	S3	EXCLUDE	EXCLUDE	No eligible intersection of coaching/relationship/competitive psychological constructs	unspecified	none	TRUE	NOT ASSESSED	No eligible intersection of coaching/relationship/competitive psychological constructs					
342	A Self-Determination Theory Perspective on Attachment, Need Satisfaction, and Well-Being in a Sample of Athletes: A Longitudinal Study	Felton, L; Jowett, S	2017	10.1123/jcsp.2016-0013	WoS	S3	EXCLUDE	EXCLUDE	Population not eligible or elite status not established	youth_only	none	TRUE	NOT ASSESSED	Population not eligible or elite status not established					
343	Longitudinal associations between athletes' controlled motivation, ill being, and perceptions of controlling coach behaviors: A Bayesian latent growth curve approach	Stenling, A; Ivarsson, A; Hassmén, P; Lindwall, M	2017	10.1016/j.psychsport.2017.03.002	WoS	S3	EXCLUDE	EXCLUDE	No eligible intersection of coaching/relationship/competitive psychological constructs	unspecified	none	TRUE	NOT ASSESSED	No eligible intersection of coaching/relationship/competitive psychological constructs					
344	The two roads from passion to sport performance and psychological well-being: The mediating role of need satisfaction, deliberate practice, and achievement goals	Verner-Filion, J; Vallerand, RJ; Amiot, CE; Mocanu, I	2017	10.1016/j.psychsport.2017.01.009	Scopus+WoS	S3	EXCLUDE	EXCLUDE	No eligible intersection of coaching/relationship/competitive psychological constructs	unspecified	none	TRUE	NOT ASSESSED	No eligible intersection of coaching/relationship/competitive psychological constructs					
345	Training Load and Fatigue Marker Associations with Injury and Illness: A Systematic Review of Longitudinal Studies	Jones, CM; Griffiths, PC; Mellalieu, SD	2017	10.1007/s40279-016-0619-5	WoS	S3	EXCLUDE	EXCLUDE	No eligible intersection of coaching/relationship/competitive psychological constructs	unspecified	none	TRUE	NOT ASSESSED	No eligible intersection of coaching/relationship/competitive psychological constructs					
346	Development and Validation of the Characteristics of Resilience in Sports Teams Inventory	Decroos, S; Morgan, PBC; Sarkar, M; Lines, RLJ; Fletcher, D; Fransen, K; Boen, F; Broek, GV	2017	10.1037/spy0000089	WoS	S3	EXCLUDE	EXCLUDE	No eligible intersection of coaching/relationship/competitive psychological constructs	unspecified	none	TRUE	NOT ASSESSED	No eligible intersection of coaching/relationship/competitive psychological constructs					
347	Linking Coach Interpersonal Style With Athlete Doping Intentions and Doping Use: A Prospective Study	Ntoumanis, N; Barkoukis, V; Gucciardi, DF; Chan, DKC	2017	10.1123/jsep.2016-0243	Scopus+WoS	S3	EXCLUDE	RETAIN	Potentially eligible; advanced to full-text or extended-abstract assessment	elite	none	FALSE	INCLUDE	Retained in narrative synthesis	Core	Phase I	Direct	8	High
348	The Internalisation of Sociocultural and Thin-Ideal Standards in Sports scale: Development and preliminary validation in female athletes participating in aesthetic sports	Scoffier-Mériaux, S; Ferrand, C; d'Arripe-Longueville, F	2017	10.1080/1612197X.2015.1122720	WoS	S3	EXCLUDE	EXCLUDE	Population not eligible or elite status not established	youth_only	P3	TRUE	NOT ASSESSED	Population not eligible or elite status not established					
349	Long-Term Prognostic Validity of Talent Selections: Comparing National and Regional Coaches, Laypersons and Novices	Schorer, J; Rienhoff, R; Fischer, L; Baker, J	2017	10.3389/fpsyg.2017.01146	WoS	S3	EXCLUDE	EXCLUDE	No eligible intersection of coaching/relationship/competitive psychological constructs	unspecified	none	FALSE	NOT ASSESSED	No eligible intersection of coaching/relationship/competitive psychological constructs					
350	Phases in the junior-to-senior transition of Swedish ice hockey players: From a conceptual framework to an empirical model	Stambulova, NB; Pehrson, S; Olsson, K	2017	10.1177/1747954117694928	WoS	S3	EXCLUDE	EXCLUDE	No eligible intersection of coaching/relationship/competitive psychological constructs	elite	none	TRUE	NOT ASSESSED	No eligible intersection of coaching/relationship/competitive psychological constructs					
351	The Influence of Playing Experience and Position on Injury Risk in NCAA Division I College Football Players	McCunn, R; Fullagar, HHK; Williams, S; Halseth, TJ; Sampson, JA; Murray, A	2017	10.1123/ijspp.2016-0803	WoS	S3	EXCLUDE	EXCLUDE	No eligible intersection of coaching/relationship/competitive psychological constructs	unspecified	none	FALSE	NOT ASSESSED	No eligible intersection of coaching/relationship/competitive psychological constructs					
352	Updated Review of the Applied Physiology of American College Football: Physical Demands, Strength and Conditioning, Nutrition, and Injury Characteristics of America's Favorite Game	Fullagar, HHK; McCunn, R; Murray, A	2017	10.1123/ijspp.2016-0783	WoS	S3	EXCLUDE	EXCLUDE	No eligible intersection of coaching/relationship/competitive psychological constructs	elite	none	TRUE	NOT ASSESSED	No eligible intersection of coaching/relationship/competitive psychological constructs					
353	Training loads and injury risk in Australian football-differing acute: chronic workload ratios influence match injury risk	Carey, DL; Blanch, P; Ong, KL; Crossley, KM; Crow, J; Morris, ME	2017	10.1136/bjsports-2016-096309	WoS	S3	EXCLUDE	EXCLUDE	Population not eligible or elite status not established	youth_only	P2	FALSE	NOT ASSESSED	Population not eligible or elite status not established					
354	Revisiting the empirical model Phases in the junior-to-senior transition of Swedish ice hockey players': External validation through focus groups and interviews	Pehrson, S; Stambulova, NB; Olsson, K	2017	10.1177/1747954117738897	WoS	S3	EXCLUDE	EXCLUDE	No eligible intersection of coaching/relationship/competitive psychological constructs	elite	none	FALSE	NOT ASSESSED	No eligible intersection of coaching/relationship/competitive psychological constructs					
355	Longitudinal associations between athletes' controlled motivation, ill-being, and perceptions of controlling coach behaviors: A Bayesian latent growth curve approach.	Stenling, Andreas ; Ivarsson, Andreas ; Hassmén, Peter ; Lindwall, Magnus	2017	10.1016/j.psychsport.2017.03.002	SPORTDiscus	S3	EXCLUDE	EXCLUDE	Population not eligible or elite status not established	youth_only	P1	FALSE	NOT ASSESSED	Population not eligible or elite status not established					
356	The effect of an additional substitution in association football. Evidence from the Italian Serie A	Varela-Quintana, C; Cuervo, JD; Prieto-Rodríguez, J	2016		WoS	S1	EXCLUDE	EXCLUDE	Population not eligible or elite status not established	youth_only	none	FALSE	NOT ASSESSED	Population not eligible or elite status not established					
357	A review of competitive sport motivation research	Clancy, RB; Herring, MP; MacIntyre, TE; Campbell, MJ	2016	10.1016/j.psychsport.2016.09.003	WoS	S1	EXCLUDE	EXCLUDE	No eligible intersection of coaching/relationship/competitive psychological constructs	elite	none	FALSE	NOT ASSESSED	No eligible intersection of coaching/relationship/competitive psychological constructs					
358	Enhancing Collegiate Women's Soccer Psychosocial and Performance Outcomes by Promoting Intrinsic Sources of Sport Enjoyment	Barnicle, SP; Burton, D	2016		WoS	S3	EXCLUDE	EXCLUDE	No eligible intersection of coaching/relationship/competitive psychological constructs	elite	none	FALSE	NOT ASSESSED	No eligible intersection of coaching/relationship/competitive psychological constructs					

359	EFFECT OF PHYSICAL AND ACADEMIC STRESS ON ILLNESS AND INJURY IN DIVISION 1 COLLEGE FOOTBALL PLAYERS	Mann, JB; Bryant, KR; Johnstone, B; Ivey, PA; Sayers, SP	2016	10.1519/JSC.0000000000001055	WoS	S3	EXCLUDE	EXCLUDE	No eligible intersection of coaching/relationship/competitive psychological constructs	elite	none	FALSE	NOT ASSESSED	No eligible intersection of coaching/relationship/competitive psychological constructs
360	The Coaching Behavior Scale for Sport (CBS-S): A psychometric evaluation of the Swedish version	Carlsson, A; Lundqvist, C	2016	10.1111/sms.12359	WoS	S3	EXCLUDE	EXCLUDE	No eligible intersection of coaching/relationship/competitive psychological constructs	elite	none	FALSE	NOT ASSESSED	No eligible intersection of coaching/relationship/competitive psychological constructs
361	Emotional intelligence in sport and exercise: A systematic review	Laborde, S; Dosseville, F; Allen, MS	2016	10.1111/sms.12510	WoS	S3	EXCLUDE	EXCLUDE	No eligible intersection of coaching/relationship/competitive psychological constructs	elite	none	TRUE	NOT ASSESSED	No eligible intersection of coaching/relationship/competitive psychological constructs
362	MOVEMENT PROFILES, MATCH EVENTS, AND PERFORMANCE IN AUSTRALIAN FOOTBALL	Johnston, RJ; Watsford, ML; Austin, DJ; Pine, MJ; Spurrs, RW	2016	10.1519/JSC.0000000000001333	WoS	S3	EXCLUDE	EXCLUDE	Population not eligible or elite status not established	youth_only	none	TRUE	NOT ASSESSED	Population not eligible or elite status not established
363	The Development, Implementation, and Evaluation of an Athlete Leadership Development Program With Female Varsity Athletes	Duguay, AM; Loughead, TM; Munroe-Chandler, KJ	2016	10.1123/tsp.2015-0050	Scopus+WoS	S3	EXCLUDE	EXCLUDE	No eligible intersection of coaching/relationship/competitive psychological constructs	elite	none	FALSE	NOT ASSESSED	No eligible intersection of coaching/relationship/competitive psychological constructs
364	Effects of basic psychological needs on Chinese youth athlete burnout under coach burnout: Using hierarchical linear modeling	Zhong, JL; Wang, QY; Bao, HS; Wang, Y; Guo, S	2026	10.1016/j.psychsport.2026.103099	Scopus+WoS	S1	EXCLUDE	EXCLUDE	Population not eligible or elite status not established	youth_only	none	FALSE	NOT ASSESSED	Population not eligible or elite status not established
365	Leveraging Student-Athlete Mental Health Through an AI-Augmented Mobile Platform: The ThriveNudge Study Protocol	Chakraborty, S; Mendro, N; Li, LX	2026	10.3390/bs16020268	Scopus+WoS	S3	EXCLUDE	EXCLUDE	No eligible intersection of coaching/relationship/competitive psychological constructs	mixed	none	FALSE	NOT ASSESSED	No eligible intersection of coaching/relationship/competitive psychological constructs
366	Trajectories of sport exhaustion, cynicism and inadequacy among adolescent student-athletes: A three-year longitudinal study of social influences in the Finnish dual career context	Kuokkanen, J; Phipps, DJ; Saarinen, M; Korhonen, J; Romar, JE; Gustafsson, H	2026	10.1016/j.psychsport.2025.103015	WoS	S3	EXCLUDE	EXCLUDE	Population not eligible or elite status not established	youth_only	none	TRUE	NOT ASSESSED	Population not eligible or elite status not established
367	Caring coaching climates emerge as key factor in promoting high school athletes' well-being and motivation, complimented by a task-involving climate	Hogue, CM	2026	10.1080/1612197X.2024.2437481	SPORTDiscus+WoS	S3	EXCLUDE	EXCLUDE	Population not eligible or elite status not established	youth_only	none	TRUE	NOT ASSESSED	Population not eligible or elite status not established
368	Perceived parental autonomy support and moral behavior in youth athletes: applying the trans-contextual model	Choi, Y; Sim, Y; Hwang, K; Bae, J	2026	10.3389/fpsyg.2026.1780952	WoS	S3	EXCLUDE	EXCLUDE	No eligible intersection of coaching/relationship/competitive psychological constructs	unspecified	none	TRUE	NOT ASSESSED	No eligible intersection of coaching/relationship/competitive psychological constructs
369	Changes in perceptions of coach- and peer-created motivational climates during the children-to-youth sport transition	Jorgensen, TM; Erickson, K; Abrahamsen, FE; Gjesdal, S	2026	10.1080/10413200.2026.2661050	WoS	S3	EXCLUDE	EXCLUDE	No eligible intersection of coaching/relationship/competitive psychological constructs	mixed	none	TRUE	NOT ASSESSED	No eligible intersection of coaching/relationship/competitive psychological constructs
370	Talent identification and development recommendations in youth sports: a real-time Delphi study	Bennett, KJM; Mccalman, W; Goddard, SG; Williams, AM; Fransen, J	2026	10.1080/10413200.2026.2636296	WoS	S3	EXCLUDE	EXCLUDE	No eligible intersection of coaching/relationship/competitive psychological constructs	unspecified	none	TRUE	NOT ASSESSED	No eligible intersection of coaching/relationship/competitive psychological constructs
371	The relationships among peer motivational climate, team preparation, and mental toughness in youth soccer players	Hwang, JH; Chung, KY; Cabral, M; Vazou, S; Song, YG	2026	10.1177/17479541261429879	WoS	S3	EXCLUDE	EXCLUDE	No eligible intersection of coaching/relationship/competitive psychological constructs	unspecified	none	TRUE	NOT ASSESSED	No eligible intersection of coaching/relationship/competitive psychological constructs
372	The Influence of Parent-Initiated Motivational Climate on Adolescent Golfers' Flow Experiences: The Mediating Role of Basic Psychological Needs	Sim, Y; Kwon, S; Lee, D	2025	10.1177/00315125251336272	SPORTDiscus+Scopus+WoS	S1	EXCLUDE	EXCLUDE	No eligible intersection of coaching/relationship/competitive psychological constructs	unspecified	none	TRUE	NOT ASSESSED	No eligible intersection of coaching/relationship/competitive psychological constructs
373	Comparative effects of French Contrast Method vs. Complex Training on explosive power and its endurance in youth badminton athletes	Huang, RY; Gao, YH; Yang, K; Mo, Y; Lu, YR; Gao, Z	2025	10.1371/journal.pone.0338383	Scopus+WoS	S2	EXCLUDE	EXCLUDE	No eligible intersection of coaching/relationship/competitive psychological constructs	unspecified	none	FALSE	NOT ASSESSED	No eligible intersection of coaching/relationship/competitive psychological constructs
374	Advancements in research on psychological and emotional aspects of student-athletes	Mao, ZJ	2025	10.3389/fpsyg.2025.1645177	WoS	S3	EXCLUDE	EXCLUDE	No eligible intersection of coaching/relationship/competitive psychological constructs	unspecified	none	FALSE	NOT ASSESSED	No eligible intersection of coaching/relationship/competitive psychological constructs
375	Effects of Cohesion With Teammates on Division-I Student-Athletes' Mental Health: An Application of the Human Need to Belong and Transactional Stress Frameworks	Cranmer, GA; Cox, EG; Rey, RT; Browning, B; Holbert, LG	2025	10.1177/21674795241267197	WoS	S3	EXCLUDE	EXCLUDE	No eligible intersection of coaching/relationship/competitive psychological constructs	unspecified	none	TRUE	NOT ASSESSED	No eligible intersection of coaching/relationship/competitive psychological constructs
376	The predictive role of coach-athlete relationship quality in training engagement and skill development among adolescent basketball players	Luo, YY; Li, SR; Cao, YY; Luo, ZJ	2025	10.3389/fpsyg.2025.1648082	SPORTDiscus+WoS	S3	EXCLUDE	EXCLUDE	No eligible intersection of coaching/relationship/competitive psychological constructs	unspecified	none	FALSE	NOT ASSESSED	No eligible intersection of coaching/relationship/competitive psychological constructs
377	Shorter follow-through is associated with greater accuracy: a kinematic analysis of the tennis forehand in youth athletes	Bottura, RM; Ferrando, V; Placidilli, F; Ruggeri, P; Faelli, E; Bisio, A	2025	10.1080/24748668.2025.2577551	WoS	S3	EXCLUDE	EXCLUDE	No eligible intersection of coaching/relationship/competitive psychological constructs	unspecified	none	FALSE	NOT ASSESSED	No eligible intersection of coaching/relationship/competitive psychological constructs
378	Coping profiles of adolescent football players and association with interpersonal coping: Do emotional competence and psychological need satisfaction matter?	Doron, J; Hayotte, M; d'Arripe-Longueville, F; Leprince, C	2024	10.1111/sms.14550	Scopus+WoS	S2	EXCLUDE	EXCLUDE	No eligible intersection of coaching/relationship/competitive psychological constructs	unspecified	none	TRUE	NOT ASSESSED	No eligible intersection of coaching/relationship/competitive psychological constructs
379	Exploring the Dynamics of Athletes' Enjoyment and Self-Determined Motivation, and of the Motivational Climate in Youth Football: A Longitudinal Perspective	Rodrigues, F; Monteiro, D; Matos, R; Jacinto, M; Antunes, R; Amaro, N	2024	10.1177/00315125231222152	Scopus+WoS	S3	EXCLUDE	EXCLUDE	Population not eligible or elite status not established	youth_only	P2	TRUE	NOT ASSESSED	Population not eligible or elite status not established
380	Motivational climate dimensions predict youth soccer players' psychosocial well-being over time	Kipp, LE; Bolter, ND	2024	10.1016/j.psychsport.2023.102518	Scopus+WoS	S3	EXCLUDE	EXCLUDE	No eligible intersection of coaching/relationship/competitive psychological constructs	mixed	none	TRUE	NOT ASSESSED	No eligible intersection of coaching/relationship/competitive psychological constructs
381	A Longitudinal Mixed Methods Case Study Investigation of the Academic, Athletic, Psychosocial and Psychological Impacts of Being of a Sport School Student Athlete	Thompson, F; Rongen, F; Cowburn, I; Till, K	2024	10.1007/s40279-024-02021-4	WoS	S3	EXCLUDE	EXCLUDE	No eligible intersection of coaching/relationship/competitive psychological constructs	elite	none	FALSE	NOT ASSESSED	No eligible intersection of coaching/relationship/competitive psychological constructs
382	Supporting my daughter, the best way I can: Parental support in youth golf	McIlmoyle, A; Sharp, LA; Woods, D; Paradis, KF	2024	10.1080/10413200.2024.2344841	WoS	S3	EXCLUDE	EXCLUDE	No eligible intersection of coaching/relationship/competitive psychological constructs	elite	none	TRUE	NOT ASSESSED	No eligible intersection of coaching/relationship/competitive psychological constructs
383	Incivility and psychological safety in youth sport: the reciprocal effects and its impact on well-being and social outcomes	Kinoshita, K; Sato, S	2023	10.1080/14413523.2022.2138110	WoS	S3	EXCLUDE	EXCLUDE	Population not eligible or elite status not established	youth_only	none	FALSE	NOT ASSESSED	Population not eligible or elite status not established
384	Temporal dynamics of the quality of the coach-athlete relationship over one season among adolescent handball players: a latent class analysis approach	Roux, V; Martinent, G; Trouilloud, D	2023	10.1080/1612197X.2022.2109185	SPORTDiscus+WoS	S3	EXCLUDE	EXCLUDE	Population not eligible or elite status not established	youth_only	none	TRUE	NOT ASSESSED	Population not eligible or elite status not established
385	Perceptions, facilitators, and barriers regarding use of the injury prevention exercise programme Knee Control among players and coaches in youth floorball: a cross-sectional survey study	Åkerlund, I; Sonesson, S; Lindblom, H; Waldén, M; Hägglund, M	2023	10.1186/s13102-023-00660-0	WoS	S3	EXCLUDE	EXCLUDE	Off-topic: physiological, technical, technological, or non-relational focus	youth_only	none	TRUE	NOT ASSESSED	Off-topic: physiological, technical, technological, or non-relational focus
386	Changes in Quality of Life, Sleep, and Physical Activity During COVID-19: A Longitudinal Study of Adolescent Athletes	Wingerson, MJ; Baugh, CM; Provance, AJ; Armento, A; Walker, GA; Howell, DR	2023	10.4085/1062-6050-0529.22	WoS	S3	EXCLUDE	EXCLUDE	No eligible intersection of coaching/relationship/competitive psychological constructs	unspecified	none	FALSE	NOT ASSESSED	No eligible intersection of coaching/relationship/competitive psychological constructs
387	Effect of coaches' interpersonal style on young athletes' individual resilience and team adherence intention: a season-long investigation	Llanos-Muñoz, R; Pulido, JJ; Nobari, H; Raya-González, J; López-Gajardo, MA	2023	10.1186/s40359-023-01445-3	Scopus+WoS	S3	EXCLUDE	EXCLUDE	No eligible intersection of coaching/relationship/competitive psychological constructs	elite	none	TRUE	NOT ASSESSED	No eligible intersection of coaching/relationship/competitive psychological constructs
388	Putting the Puzzle Back Together-A Narrative Case Study of an Athlete Who Survived Child Sexual Abuse in Sport	Gillard, A; St-Pierre, E; Radziszewski, S; Parent, S	2022	10.3389/fpsyg.2022.856957	WoS	S3	EXCLUDE	EXCLUDE	No eligible intersection of coaching/relationship/competitive psychological constructs	unspecified	none	TRUE	NOT ASSESSED	No eligible intersection of coaching/relationship/competitive psychological constructs
389	High compliance with the injury prevention exercise programme Knee Control is associated with a greater injury preventive effect in male, but not in female, youth floorball players	Åkerlund, I; Waldén, M; Sonesson, S; Lindblom, H; Hägglund, M	2022	10.1007/s00167-021-06644-2	WoS	S3	EXCLUDE	EXCLUDE	No eligible intersection of coaching/relationship/competitive psychological constructs	unspecified	none	FALSE	NOT ASSESSED	No eligible intersection of coaching/relationship/competitive psychological constructs
390	The implementation and evaluation of an athlete leadership development program with male youth ice hockey players	Boisvert, MM; Loughead, TM; Munroe-Chandler, KJ	2022	10.3389/fpsyg.2022.648039	WoS	S3	EXCLUDE	EXCLUDE	Population not eligible or elite status not established	youth_only	none	TRUE	NOT ASSESSED	Population not eligible or elite status not established
391	Mental health protective factors among flourishing Canadian women university student-athletes	Pankow, K; McHugh, TLF; Mosewich, AD; Holt, NL	2021	10.1016/j.psychsport.2020.101847	WoS	S3	EXCLUDE	EXCLUDE	No eligible intersection of coaching/relationship/competitive psychological constructs	unspecified	none	TRUE	NOT ASSESSED	No eligible intersection of coaching/relationship/competitive psychological constructs
392	A united front: Coach and teammate motivational climate and team cohesion among female adolescent athletes	Weiss, MR; Moehnke, HJ; Kipp, LE	2021	10.1177/17479541211006905	WoS	S3	EXCLUDE	EXCLUDE	No eligible intersection of coaching/relationship/competitive psychological constructs	unspecified	none	TRUE	NOT ASSESSED	No eligible intersection of coaching/relationship/competitive psychological constructs
393	Perceived coach-created environment directly predicts high school athletes' physical activity during sport	Chu, TL; Zhang, XX; Lee, J; Zhang, T	2021	10.1177/1747954120959733	WoS	S3	EXCLUDE	EXCLUDE	No eligible intersection of coaching/relationship/competitive psychological constructs	unspecified	none	TRUE	NOT ASSESSED	No eligible intersection of coaching/relationship/competitive psychological constructs
394	The role of growth, maturation and sporting environment on the development of performance and technical and tactical skills in youth basketball players: The INEX study	Guimaraes, E; Baxter-Jones, ADG; Williams, AM; Tavares, F; Janeira, MA; Maia, J	2021	10.1080/02640414.2020.1853334	WoS	S3	EXCLUDE	EXCLUDE	No eligible intersection of coaching/relationship/competitive psychological constructs	elite	none	FALSE	NOT ASSESSED	No eligible intersection of coaching/relationship/competitive psychological constructs
395	Evaluation of the NFHS Online Captains Leadership Course: Student Athletes' Views of Effectiveness	Walker, LF; Gould, DR	2021	10.3389/fpsyg.2021.648559	WoS	S3	EXCLUDE	EXCLUDE	No eligible intersection of coaching/relationship/competitive psychological constructs	unspecified	none	TRUE	NOT ASSESSED	No eligible intersection of coaching/relationship/competitive psychological constructs
396	Transformational parenting and coaching on mental toughness and physical performance in adolescent soccer players: The moderating effect of athlete age	Murray, RM; Dugdale, JH; Habeeb, CM; Arthur, CA	2021	10.1080/17461391.2020.1765027	WoS	S3	EXCLUDE	EXCLUDE	No eligible intersection of coaching/relationship/competitive psychological constructs	unspecified	none	TRUE	NOT ASSESSED	No eligible intersection of coaching/relationship/competitive psychological constructs
397	Perceived parental behaviours and motivational processes among adolescent athletes in intensive training centres: A profile approach	Lienhart, N; Nicaise, V; Martinent, G; Guillet-Descas, E	2020	10.1016/j.psychsport.2020.101708	WoS	S3	EXCLUDE	EXCLUDE	No eligible intersection of coaching/relationship/competitive psychological constructs	elite	none	FALSE	NOT ASSESSED	No eligible intersection of coaching/relationship/competitive psychological constructs
398	Longitudinal associations between perceived programme quality, basic needs support and basic needs satisfaction within youth sport: A person-centred approach	Bean, C; Solstad, BE; Ivarsson, A; Forneris, T	2020	10.1080/1612197X.2018.1462234	WoS	S3	EXCLUDE	EXCLUDE	No eligible intersection of coaching/relationship/competitive psychological constructs	unspecified	none	TRUE	NOT ASSESSED	No eligible intersection of coaching/relationship/competitive psychological constructs

399	Snapchat and child sexual abuse in sport: Protecting child athletes in the social media age	Sanderson, J; Weathers, MR	2020	10.1016/j.smr.2019.04.006	WoS	S3	EXCLUDE	EXCLUDE	No eligible intersection of coaching/relationship/competitive psychological constructs	elite	none	FALSE	NOT ASSESSED	No eligible intersection of coaching/relationship/competitive psychological constructs					
400	Positive youth development related athlete experiences and coach behaviors following a targeted coach education course	MacDonald, DJ; Camiré, M; Erickson, K; Santos, F	2020	10.1177/1747954120942017	WoS	S3	EXCLUDE	EXCLUDE	Population not eligible or elite status not established	youth_only	P3	TRUE	NOT ASSESSED	Population not eligible or elite status not established					
401	The impact of humanistic coach training on youth athletes' development through sport	Falcao, WR; Bloom, GA; Sabiston, CM	2020	10.1177/1747954120933975	WoS	S3	EXCLUDE	EXCLUDE	No eligible intersection of coaching/relationship/competitive psychological constructs	elite	none	TRUE	NOT ASSESSED	No eligible intersection of coaching/relationship/competitive psychological constructs					
402	Physical Performance Measures Correlate with Head Impact Exposure in Youth Football	Kelley, ME; Jones, DA; Espeland, MA; Rosenberg, ML; Miles, CM; Whitlow, CT; Maldjian, JA; Stitzel, JD; Urban, JE	2020	10.1249/MSS.0000000000002144	WoS	S3	EXCLUDE	EXCLUDE	Population not eligible or elite status not established	youth_only	none	TRUE	NOT ASSESSED	Population not eligible or elite status not established					
403	The dynamic nature of connection and its relation to character in youth sport	Herbison, JD; Vierimaa, M; Côté, J; Martin, LJ	2019	10.1080/1612197X.2017.1423507	WoS	S3	EXCLUDE	EXCLUDE	No eligible intersection of coaching/relationship/competitive psychological constructs	mixed	none	FALSE	NOT ASSESSED	No eligible intersection of coaching/relationship/competitive psychological constructs					
404	Longitudinal trajectories of emotions among young athletes involving in intense training centres: Do emotional intelligence and emotional regulation matter?	Cece, V; Guillet-Descas, E; Nicaise, V; Lienhart, N; Martinent, G	2019	10.1016/j.psychsport.2019.01.011	Scopus+WoS	S3	EXCLUDE	EXCLUDE	No eligible intersection of coaching/relationship/competitive psychological constructs	unspecified	none	FALSE	NOT ASSESSED	No eligible intersection of coaching/relationship/competitive psychological constructs					
405	Helping student-athletes learn to self-regulate to alleviate burnout: a multiple case study showcasing their challenging but altering experiences	Dubuc-Charbonneau, N; Durand-Bush, N	2018	10.1080/2159676X.2018.1433226	WoS	S3	EXCLUDE	EXCLUDE	No eligible intersection of coaching/relationship/competitive psychological constructs	elite	none	FALSE	NOT ASSESSED	No eligible intersection of coaching/relationship/competitive psychological constructs					
406	UNDERSTANDING THE RELATIONSHIP BETWEEN COACH AND ATHLETE PERCEPTIONS OF TRAINING INTENSITY IN YOUTH SPORT	Scantlebury, S; Till, K; Sawczuk, T; Weakley, J; Jones, B	2018	10.1519/JSC.0000000000002204	SPORTDiscus+WoS	S3	EXCLUDE	EXCLUDE	No eligible intersection of coaching/relationship/competitive psychological constructs	elite	none	FALSE	NOT ASSESSED	No eligible intersection of coaching/relationship/competitive psychological constructs					
407	An empirical examination comparing the Mindfulness-Acceptance-Commitment approach and Psychological Skills Training for the mental health and sport performance of female student athletes	Gross, M; Moore, ZE; Gardner, FL; Wolanin, AT; Pess, R; Marks, DR	2018	10.1080/1612197X.2016.1250802	WoS	S3	EXCLUDE	EXCLUDE	No eligible intersection of coaching/relationship/competitive psychological constructs	elite	none	FALSE	NOT ASSESSED	No eligible intersection of coaching/relationship/competitive psychological constructs					
408	Youth Sport Coaches' Well-Being Across the Season: The Psychological Costs and Benefits of Giving Empowering and Disempowering Sports Coaching to Athletes.	Solstad, Bård Erlend ; Ivarsson, Andreas ; Haug, Ellen Merethe ; Ommundsen, Yngvar	2018	10.1123/iscj.2017-0026	SPORTDiscus	S3	EXCLUDE	EXCLUDE	Population not eligible or elite status not established	youth_only	P1	TRUE	NOT ASSESSED	Population not eligible or elite status not established					
409	Exploring relationships between the coach-initiated motivational climate and psychological coping skills over the high school American football season	Gano-Overway, LA; Steele, E; Boyce, BA; Whaley, D	2017	10.1177/1747954117738873	WoS	S3	EXCLUDE	EXCLUDE	Population not eligible or elite status not established	youth_only	P1	TRUE	NOT ASSESSED	Population not eligible or elite status not established					
410	Understanding psychological changes in adolescent wrestlers participating in an intensive training camp: A mixed-methods investigation	Driska, AP; Gould, DR; Pierce, S; Cowburn, IHJ	2017	10.7352/IJSP.2017.48.303	Scopus+WoS	S3	EXCLUDE	EXCLUDE	Population not eligible or elite status not established	youth_only	P1	FALSE	NOT ASSESSED	Population not eligible or elite status not established					
411	Just for the fun of it: coaches' perceptions of an exemplary community youth sport program	Vierimaa, M; Turnnidge, J; Bruner, M; Côté, J	2017	10.1080/17408989.2017.1341473	WoS	S3	EXCLUDE	EXCLUDE	No eligible intersection of coaching/relationship/competitive psychological constructs	elite	none	FALSE	NOT ASSESSED	No eligible intersection of coaching/relationship/competitive psychological constructs					
412	Young Athletes' Perceptions of Coach Behaviors and Their Implications on Their Well- and Ill-Being Over Time	González, L; García-Merita, M; Castillo, I; Balaguer, I	2016	10.1519/JSC.0000000000001170	WoS	S3	EXCLUDE	EXCLUDE	No eligible intersection of coaching/relationship/competitive psychological constructs	elite	none	TRUE	NOT ASSESSED	No eligible intersection of coaching/relationship/competitive psychological constructs					
413	A Three-Wave Longitudinal Test of Self-Determination Theory's Mediation Model of Engagement and Disaffection in Youth Sport	Curran, T; Hill, AP; Ntoumanis, N; Hall, HK; Jowett, GE	2016	10.1123/jsep.2015-0016	WoS	S3	EXCLUDE	EXCLUDE	No eligible intersection of coaching/relationship/competitive psychological constructs	elite	none	TRUE	NOT ASSESSED	No eligible intersection of coaching/relationship/competitive psychological constructs					
414	High school athletes' self-determined motivation: The independent and interactive effects of coach, father, and mother autonomy support	Amorose, AJ; Anderson-Butcher, D; Newman, TJ; Fraina, M; Iachini, A	2016	10.1016/j.psychsport.2016.05.005	SPORTDiscus+Scopus+WoS	S3	EXCLUDE	EXCLUDE	Population not eligible or elite status not established	youth_only	P2	TRUE	NOT ASSESSED	Population not eligible or elite status not established					
415	A season-long examination of the intervention tone of coach-athlete interactions and athlete development in youth sport	Erickson, K; Côté, J	2016	10.1016/j.psychsport.2015.08.006	WoS	S3	EXCLUDE	EXCLUDE	No eligible intersection of coaching/relationship/competitive psychological constructs	mixed	none	FALSE	NOT ASSESSED	No eligible intersection of coaching/relationship/competitive psychological constructs					
416	Sports Specialization, Part II: Alternative Solutions to Early Sport Specialization in Youth Athletes	Myer, GD; Jayanthi, N; DiFiori, JP; Faigenbaum, AD; Kiefer, AW; Logerstedt, D; Micheli, LJ	2016	10.1177/1941738115614811	WoS	S3	EXCLUDE	EXCLUDE	No eligible intersection of coaching/relationship/competitive psychological constructs	unspecified	none	FALSE	NOT ASSESSED	No eligible intersection of coaching/relationship/competitive psychological constructs					
417	Rapid Weight Loss Among Adolescents Participating In Competitive Judo	Berkovich, BE; Eliakim, A; Nemet, D; Stark, AH; Sinai, T	2016	10.1123/ijsnem.2015-0196	WoS	S3	EXCLUDE	EXCLUDE	Off-topic: physiological, technical, technological, or non-relational focus	unspecified	P3	FALSE	NOT ASSESSED	Off-topic: physiological, technical, technological, or non-relational focus					
418	Assessing toxic leadership in high-performance sports: Psychometric validation of a Swedish Version of the Toxic Leadership Scale for Sports (TLS- S)	Lundqvist C.,Johansson S.	2026		Scopus	S1	INCLUDE	RETAIN	Potentially eligible; advanced to full-text or extended-abstract assessment	elite	P1, P2	FALSE	EXCLUDE_AT_ELIGIBILITY	No direct or phase-relevant temporal content					
419	Acceptable or Not: An In-depth Analysis of Adolescent Competitive Athletes' Perceptions on Abusive Coaching Behaviors	Marsollier E.,Hauw D.,von Roten F.C.	2025		Scopus	S1	INCLUDE	RETAIN	Potentially eligible; advanced to full-text or extended-abstract assessment	mixed/unclear	P1	FALSE	EXCLUDE_AT_ELIGIBILITY	Elite/professional status not confirmed					
420	Interpersonal climate and moral conduct in competitive sport: A dual-route model with serial mediation	Wang Z.,Liu T.,Hong S.,Wang W.,Wang F.	2025		Scopus	S1+S3	INCLUDE	RETAIN	Potentially eligible; advanced to full-text or extended-abstract assessment	elite	P1, P2, P3	TRUE	EXCLUDE_AT_ELIGIBILITY	Insufficient fit with the temporal cascade after eligibility assessment					
421	Qualitative analysis of elite football players' causal attribution to not playing: A perspective of attribution theory	Ulas M.,Adilogullari I.,Senel E.	2025		Scopus	S1	INCLUDE	RETAIN	Potentially eligible; advanced to full-text or extended-abstract assessment	elite	P1, P2, P3	FALSE	INCLUDE	Retained in narrative synthesis	Core	Phase II / relational aftermath	Indirect	8	High
422	Using the Conceptual Framework for Examining Sport Teams to Understand Group Dynamics in Professional Soccer	Gonzalez-Ponce I.,Diaz-Garcia J.,Ponce-Bordon J.C.,Jimenez-Castuera R.,Lopez-Gajardo M.A.	2022		Scopus	S1	INCLUDE	RETAIN	Potentially eligible; advanced to full-text or extended-abstract assessment	elite	P1	FALSE	EXCLUDE_AT_ELIGIBILITY	Did not examine the intersection of at least two construct pillars					
423	Examining the psychometric properties of the Controlling Coach Behaviors Scale in Chinese Elite Athletes	Zhao D.,Zhou Y.	2022		Scopus	S1	MAYBE	RETAIN	Potentially eligible; advanced to full-text or extended-abstract assessment	elite	P1, P3	FALSE	EXCLUDE_AT_ELIGIBILITY	No direct or phase-relevant temporal content					
424	Quality of coach-athlete relationship and coping as associated factors of stress, anxiety, burnout, and depression symptoms of soccer players in transition to professional: a prospective study	da Silva A.A.,de Sousa Fortes L.,da Cruz Carvalho L.P.,... do Nascimento Junior J.R.A.,Barbieri R.A.	2022		Scopus	S1+S3	INCLUDE	RETAIN	Potentially eligible; advanced to full-text or extended-abstract assessment	elite	P1, P2, P3	TRUE	INCLUDE	Retained in narrative synthesis	Core	Phase II	Direct	9	High
425	Motivational beliefs and subjective effort in adolescent athletes	Mudrak J.,Slepicka P.,Slepickova I.,Zabrodska K.,Knoblochova M.	2021		Scopus	S1	EXCLUDE	EXCLUDE	Population not eligible or elite status not established	mixed/unclear	P1, P2, P3	FALSE	NOT ASSESSED	Population not eligible or elite status not established					
426	Which are the most determinant psychological factors in olympic shooting performance? A self-perspective from elite shooters	Moreira da Silva F.,Malico Sousa P.,Pinheiro V.B.,... Refoyo Roman I.,Mon-Lopez D.	2021		Scopus	S1	INCLUDE	RETAIN	Potentially eligible; advanced to full-text or extended-abstract assessment	elite	P1, P2, P3	TRUE	INCLUDE	Retained in narrative synthesis	Core	Phase III	Indirect	7	Moderate
427	Perceptions of low back pain in elite gymnastics: A multi-disciplinary qualitative focus group study	Fawcett L.,Heneghan N.R.,James S.,Rushton A.	2020		Scopus	S1	EXCLUDE	EXCLUDE	Off-topic: physiological, technical, technological, or non-relational focus	elite	P1, P2	FALSE	NOT ASSESSED	Off-topic: physiological, technical, technological, or non-relational focus					
428	"Coach really knew what i needed and understood me well as a person": Effective communication acts in coach–athlete interactions among Korean olympic archers	Kim Y.,Park I.	2020		Scopus	S1	INCLUDE	RETAIN	Potentially eligible; advanced to full-text or extended-abstract assessment	elite	P1, P2, P3	FALSE	INCLUDE	Retained in narrative synthesis	Core	Phase III	Direct	9	High
429	Emotion, psychological well-being and their influence on resilience. A study with semi-professional athletes	Trigueros R.,Aguilar-Parra J.M.,Alvarez J.F.,Gonzalez-Bernal J.J.,Lopez-Liria R.	2019		Scopus	S1	MAYBE	RETAIN	Potentially eligible; advanced to full-text or extended-abstract assessment	elite	P1, P2, P3	TRUE	EXCLUDE_AT_ELIGIBILITY	Insufficient fit with the temporal cascade after eligibility assessment					
430	Motivation in the practice of judo in non-professional athletes | Motivación en la práctica del judo en deportistas no profesionales	Castro-Sanchez M.,Zurita-Ortega F.,Zafra-Santos E.,... Chacon-Cuberos R.,Valdivia-Moral P.	2019		Scopus	S1	EXCLUDE	EXCLUDE	Population not eligible or elite status not established	elite	P1, P2, P3	FALSE	NOT ASSESSED	Population not eligible or elite status not established					
431	Using vignettes to analyze potential influences of effective strength and conditioning coaching on athlete development	Szedlak C.,Smith M.,Day M.,Callary B.	2018		Scopus	S1	INCLUDE	RETAIN	Potentially eligible; advanced to full-text or extended-abstract assessment	elite	P1, P3	FALSE	EXCLUDE_AT_ELIGIBILITY	No direct or phase-relevant temporal content					
432	Providing social support to female Olympic athletes	Poucher Z.A.,Tamminen K.A.,Kerr G.	2018		Scopus	S1	INCLUDE	RETAIN	Potentially eligible; advanced to full-text or extended-abstract assessment	elite	P2	FALSE	INCLUDE	Retained in narrative synthesis	Contextual	Cross-cutting	Indirect	6	Moderate
433	Influences of perfectionism and motivational climate on attitudes towards doping among Korean national athletes: A cross sectional study	Bae M.,Yoon J.,Kang H.,Kim T.	2017		Scopus	S1	INCLUDE	RETAIN	Potentially eligible; advanced to full-text or extended-abstract assessment	elite	P1, P3	FALSE	EXCLUDE_AT_ELIGIBILITY	No direct or phase-relevant temporal content					
434	Pathos and orchestration in elite sport: The experiences of NCAA DI student- athletes	Raabe J.,Readdy T.,Zakrajsek R.A.	2017		Scopus	S1	MAYBE	RETAIN	Potentially eligible; advanced to full-text or extended-abstract assessment	elite	P1, P2, P3	FALSE	EXCLUDE_AT_ELIGIBILITY	No direct or phase-relevant temporal content					
435	'This might be him; the guy I'm gonna marry': Love and sexual relationships between female elite-athletes and male coaches	Johansson S.,Larsson H.	2017		Scopus	S1	INCLUDE	RETAIN	Potentially eligible; advanced to full-text or extended-abstract assessment	elite	P1, P2, P3	FALSE	EXCLUDE_AT_ELIGIBILITY	No direct or phase-relevant temporal content					
436	Offended by the algorithm: The hidden interpersonal costs of clients seeking AI second opinion	Spassova G.,Palmeira M.	2026		Scopus	S2	EXCLUDE	EXCLUDE	Off-topic: physiological, technical, technological, or non-relational focus	elite	P2, P3	FALSE	NOT ASSESSED	Off-topic: physiological, technical, technological, or non-relational focus					
437	One Sprinter, Two Olympic Preparations: A Single-Athlete Longitudinal Observational Study of Training-Intensity Distribution and Implications for Future 50 m Events	Papadimitriou K.,Margaritelis N.V.,Tsalis G.	2026		Scopus	S2	EXCLUDE	EXCLUDE	Off-topic: physiological, technical, technological, or non-relational focus	elite	P1	TRUE	NOT ASSESSED	Off-topic: physiological, technical, technological, or non-relational focus					
438	Training Stress, Sleep Behaviour, and Recovery Self-Regulation in Elite Female Boxers: A Behavioural Pattern Analysis Using Data-Driven Methods	Rattanasateankij W.,Senakham T.,Punthipayanon S.,Puttasimma T.,Senakham N.	2025		Scopus	S2	MAYBE	RETAIN	Potentially eligible; advanced to full-text or extended-abstract assessment	elite	P1, P3	TRUE	EXCLUDE_AT_ELIGIBILITY	Insufficient fit with the temporal cascade after eligibility assessment					
439	Strategies to reduce hyperglycemia-related anxiety in elite athletes with type 1 diabetes: A qualitative analysis	Katz A.,Shulkin A.,Fortier M.-A.,... Rabasa-Lhoret R.,Brazeau A.-S.	2025		Scopus	S2	MAYBE	RETAIN	Potentially eligible; advanced to full-text or extended-abstract assessment	elite	P2, P3	TRUE	EXCLUDE_AT_ELIGIBILITY	Insufficient fit with the temporal cascade after eligibility assessment					
440	Sport Anxiety, Fear of Negative Evaluation, Stress and Coping as Predictors of Athlete's Sensitivity to the Behavior of Supporters	Gabrys K.,Wontorczyk A.	2023		Scopus	S2	MAYBE	RETAIN	Potentially eligible; advanced to full-text or extended-abstract assessment	elite	P1, P2, P3	FALSE	EXCLUDE_AT_ELIGIBILITY	No direct or phase-relevant temporal content					
441	Depressive symptoms and burnout in football players: A systematic review	Sarmento H.,Frontini R.,Marques A.,... Campos M.J.,Clemente F.M.	2021		Scopus	S2	MAYBE	RETAIN	Potentially eligible; advanced to full-text or extended-abstract assessment	elite	P1, P3	TRUE	EXCLUDE_AT_ELIGIBILITY	Measurement or review paper not retained in the empirical synthesis					
442	PoseShot: hybrid CNN–BiLSTM transformer model for free throw action recognition via pose analysis	Hsu W.-C.,Lee C.-C.,Lee Y.-H.,... Ho C.-C.,Chang Y.-C.	2026		Scopus	S3	EXCLUDE	EXCLUDE	Off-topic: physiological, technical, technological, or non-relational focus	mixed/unclear	P1, P2	FALSE	NOT ASSESSED	Off-topic: physiological, technical, technological, or non-relational focus					
443	Prediction of athlete performance based on a gradient regression model	Wei X.,Liang S.,Diao W.	2026		Scopus	S3	EXCLUDE	EXCLUDE	Off-topic: physiological, technical, technological, or non-relational focus	mixed/unclear	P1, P2, P3	TRUE	NOT ASSESSED	Off-topic: physiological, technical, technological, or non-relational focus					

444	The development of mental toughness: A journey into the perceptions and experiences of a world-class padel athlete	Barreiros A.,Maia M.,Fonseca A.M.,Dias C.	2026	Scopus	S3	INCLUDE	RETAIN	Potentially eligible; advanced to full-text or extended-abstract assessment	elite	P1, P2, P3	TRUE	EXCLUDE_AT_ELIGIBILITY	Insufficient fit with the temporal cascade after eligibility assessment					
445	Performance inflation in junior tennis: Longitudinal analysis and Bayesian forecasting of ranking thresholds, efficiency, and access equity	Bozdech M.	2026	Scopus	S3	EXCLUDE	EXCLUDE	Population not eligible or elite status not established	elite	P1	TRUE	NOT ASSESSED	Population not eligible or elite status not established					
446	The Lions Sports Academy TackleTEK Tool: The intra-and inter-coach reliability of assessing tackling competency in rugby union	Owen R.,Knight C.J.,Page W.,... Kilduff L.,Waldron M.	2026	Scopus	S3	EXCLUDE	EXCLUDE	Off-topic: physiological, technical, technological, or non-relational focus	mixed/unclear	P1, P2	TRUE	NOT ASSESSED	Off-topic: physiological, technical, technological, or non-relational focus					
447	An intelligent taekwondo coaching system based on augmented reality technology with real-time feedback mechanisms	Yang F.,Wang Z.	2025	Scopus	S3	EXCLUDE	EXCLUDE	Off-topic: physiological, technical, technological, or non-relational focus	mixed/unclear	P1, P3	FALSE	NOT ASSESSED	Off-topic: physiological, technical, technological, or non-relational focus					
448	Adverse competition-related cognitions and it's relation to satisfaction and subjective performance: a validation study in a sample of English-speaking athletes	Alena M.-K.,Michele W.,Stefan B.	2025	Scopus	S3	EXCLUDE	EXCLUDE	No eligible intersection of coaching/relationship/competitive psychological constructs	mixed/unclear	P1, P2, P3	TRUE	NOT ASSESSED	No eligible intersection of coaching/relationship/competitive psychological constructs					
449	Investigating sport persistence through the development of the Sport Persistence Questionnaire	Kovacs K.E.,Csukonyi C.	2025	Scopus	S3	EXCLUDE	EXCLUDE	Off-topic: physiological, technical, technological, or non-relational focus	mixed/unclear	P1, P2, P3	TRUE	NOT ASSESSED	Off-topic: physiological, technical, technological, or non-relational focus					
450	Analyzing the impact of prolonged pauses on soccer performance	Atici A.R.,Kamis O.,Edriss S.,... Bowman T.G.,Nobari H.	2025	Scopus	S3	EXCLUDE	EXCLUDE	Off-topic: physiological, technical, technological, or non-relational focus	elite	P1, P2, P3	TRUE	NOT ASSESSED	Off-topic: physiological, technical, technological, or non-relational focus					
451	Enhancing team dynamics: Unveiling synergy effects through social network analysis in professional basketball	Wei B.,Li Z.,Zhou C.,Gomez M.-A.,Zhang M.	2025	Scopus	S3	EXCLUDE	EXCLUDE	Off-topic: physiological, technical, technological, or non-relational focus	elite	P1	TRUE	NOT ASSESSED	Off-topic: physiological, technical, technological, or non-relational focus					
452	Adaptation and psychometric properties of Psychological Skills Inventory for Sport (PSIS-R5) in Latvian athletes: Insights and implications for practice	Volgemute K.,Ulme G.,Perepjolkina V.,... Tihija A.,Grants E.	2025	Scopus	S3	EXCLUDE	EXCLUDE	No eligible intersection of coaching/relationship/competitive psychological constructs	elite	P1, P2, P3	TRUE	NOT ASSESSED	No eligible intersection of coaching/relationship/competitive psychological constructs					
453	Longitudinal relationships between emotional processes and perceived performances among athletes: The moderating effect of emotional intelligence	Levillain G.,Martinent G.,Saby Y.,Nicolas M.	2025	Scopus	S3	INCLUDE	RETAIN	Potentially eligible; advanced to full-text or extended-abstract assessment	mixed/unclear	P1, P2, P3	TRUE	INCLUDE	Retained in narrative synthesis	Contextual	Phase III	Direct	7	Moderate
454	Creatine kinase and neuromuscular fatigue responses following differing spells of simulated cricket fast bowling	Bray J.W.,Towlson C.,Hayes S.C.,Fogarty M.	2025	Scopus	S3	EXCLUDE	EXCLUDE	Off-topic: physiological, technical, technological, or non-relational focus	mixed/unclear	P1, P2, P3	TRUE	NOT ASSESSED	Off-topic: physiological, technical, technological, or non-relational focus					
455	Effects of brief mindfulness intervention on mental fatigue and recovery in basketball tactical performance	Cao S.,Liu J.,Geok S.K.,Sun H.,Wang X.	2024	Scopus	S3	EXCLUDE	EXCLUDE	Off-topic: physiological, technical, technological, or non-relational focus	mixed/unclear	P1	TRUE	NOT ASSESSED	Off-topic: physiological, technical, technological, or non-relational focus					
456	Impact of offensive team variables on goal scoring in the first division of the spanish soccer league: a comprehensive 10-year study	Prieto-Gonzalez P.,Martin V.,Pacholek M.,Sal-de-Rellan A.,Marcelino R.	2024	Scopus	S3	EXCLUDE	EXCLUDE	Off-topic: physiological, technical, technological, or non-relational focus	mixed/unclear	P1	TRUE	NOT ASSESSED	Off-topic: physiological, technical, technological, or non-relational focus					
457	Bystander Intervention in Football and Sports. A Quasi-Experimental Feasibility Study of a Bystander Violence Prevention Program in the United Kingdom	Kovalenko A.G.,Fenton R.A.	2024	Scopus	S3	EXCLUDE	EXCLUDE	No eligible intersection of coaching/relationship/competitive psychological constructs	elite	P1, P3	TRUE	NOT ASSESSED	No eligible intersection of coaching/relationship/competitive psychological constructs					
458	Eye-Tracking Analyses of a Coach's Pointing Gestures Timed With Speech: Implications for Players' Recall of Basketball Tactical Instructions	Ben Chikha H.,Mguidich H.,Zoudji B.,Khacharem A.	2024	Scopus	S3	EXCLUDE	EXCLUDE	No eligible intersection of coaching/relationship/competitive psychological constructs	mixed/unclear	P1	FALSE	NOT ASSESSED	No eligible intersection of coaching/relationship/competitive psychological constructs					
459	Match analysis and probability of winning a point in elite men's singles tennis	Prieto-Lage I.,Parames-Gonzalez A.,Torres-Santos D.,... Reguera-Lopez-de-la-Osa X.,GutierrezSantiago A.	2023	Scopus	S3	EXCLUDE	EXCLUDE	Off-topic: physiological, technical, technological, or non-relational focus	elite	P1, P2	TRUE	NOT ASSESSED	Off-topic: physiological, technical, technological, or non-relational focus					
460	Elite lean athletes at sports high schools face multiple risks for mental health concerns and are in need of psychosocial support	Lundqvist C.,Schary D.P.,Eklof E.,Zand S.,Jacobsson J.	2023	Scopus	S3	EXCLUDE	EXCLUDE	Population not eligible or elite status not established	elite	P1, P2, P3	TRUE	NOT ASSESSED	Population not eligible or elite status not established					
461	Amplifying perceptual demands: How changes in the colour vests affect youth players performance during medium-sided games	Coutinho D.,Goncalves B.,Folgado H.,... Santos S.,Sampaio J.	2022	Scopus	S3	EXCLUDE	EXCLUDE	Population not eligible or elite status not established	mixed/unclear	P1	TRUE	NOT ASSESSED	Population not eligible or elite status not established					
462	Redesigning a Youth Soccer Program: Holistic Development of Athletes	Moore M.,Blom L.,Califano K.,... Gabler T.,Sullivan M.	2021	Scopus	S3	EXCLUDE	EXCLUDE	Population not eligible or elite status not established	mixed/unclear	P1, P2, P3	TRUE	NOT ASSESSED	Population not eligible or elite status not established					
463	In-season training responses and perceived wellbeing and recovery status in professional soccer players	Mateus N.,Goncalves B.,Felipe J.L.,... Weldon A.,Sampaio J.	2021	Scopus	S3	EXCLUDE	EXCLUDE	No eligible intersection of coaching/relationship/competitive psychological constructs	elite	P1, P2, P3	TRUE	NOT ASSESSED	No eligible intersection of coaching/relationship/competitive psychological constructs					
464	Run-up strategies in competitive long jumping: How an ecological dynamics rationale can support coaches to design individualised practice tasks	McCosker C.,Renshaw I.,Polman R.,Greenwood D.,Davids K.	2021	Scopus	S3	EXCLUDE	EXCLUDE	Off-topic: physiological, technical, technological, or non-relational focus	elite	P1, P2	TRUE	NOT ASSESSED	Off-topic: physiological, technical, technological, or non-relational focus					
465	Balance dynamics are related to age and levels of expertise. Application in young and adult tennis players	Caballero C.,Barbado D.,Hernandez-Davo H.,Hernandez-Davo J.L.,Moreno F.J.	2021	Scopus	S3	EXCLUDE	EXCLUDE	Off-topic: physiological, technical, technological, or non-relational focus	mixed/unclear	P1, P2	FALSE	NOT ASSESSED	Off-topic: physiological, technical, technological, or non-relational focus					
466	Facilitating Positive Youth Development in Elite Youth Hockey: Exploring Coaches' Capabilities, Opportunities, and Motivations	Preston C.,Allan V.,Fraser-Thomas J.	2021	Scopus	S3	INCLUDE	RETAIN	Potentially eligible; advanced to full-text or extended-abstract assessment	elite	P1, P3	TRUE	EXCLUDE_AT_ELIGIBILITY	Insufficient fit with the temporal cascade after eligibility assessment					
467	Athletes' Perception of Leadership According to Their Perceptions of Goal Achievement and Sport Results	Gomes A.R.,Almeida A.,Resende R.	2020	Scopus	S3	INCLUDE	RETAIN	Potentially eligible; advanced to full-text or extended-abstract assessment	mixed/unclear	P1	TRUE	EXCLUDE_AT_ELIGIBILITY	Elite/professional status not confirmed					
468	What does it mean to be "mentally tough" as a NCAA division I collegiate coach?	Weinberg R.,Freysinger V.,Vealey R.,Block C.	2020	Scopus	S3	MAYBE	RETAIN	Potentially eligible; advanced to full-text or extended-abstract assessment	elite	P1, P3	TRUE	EXCLUDE_AT_ELIGIBILITY	Insufficient fit with the temporal cascade after eligibility assessment					
469	Young Dutch commercially sponsored kite surfers: free as a bird?	Smits F.	2019	Scopus	S3	EXCLUDE	EXCLUDE	No eligible intersection of coaching/relationship/competitive psychological constructs	mixed/unclear	P1, P2	FALSE	NOT ASSESSED	No eligible intersection of coaching/relationship/competitive psychological constructs					
470	The Optimal Team Functioning Model: A Grounded Theory Framework to Guide Teamwork in Curling	Collins J.,Durand-Bush N.	2019	Scopus	S3	INCLUDE	RETAIN	Potentially eligible; advanced to full-text or extended-abstract assessment	mixed/unclear	P1, P2	TRUE	EXCLUDE_AT_ELIGIBILITY	Elite/professional status not confirmed					
471	Extracting spatial-temporal features that describe a team match demands when considering the effects of the quality of opposition in elite football	Goncalves B.,Coutinho D.,Exel J.,... Lago C.,Sampaio J.	2019	Scopus	S3	EXCLUDE	EXCLUDE	Off-topic: physiological, technical, technological, or non-relational focus	elite	P1, P2	TRUE	NOT ASSESSED	Off-topic: physiological, technical, technological, or non-relational focus					
472	Overcoming the problem of multicollinearity in sports performance data: A novel application of partial least squares correlation analysis	Weaving D.,Jones B.,Ireton M.,... Till K.,Beggs C.B.	2019	Scopus	S3	EXCLUDE	EXCLUDE	Off-topic: physiological, technical, technological, or non-relational focus	elite	P1, P2	TRUE	NOT ASSESSED	Off-topic: physiological, technical, technological, or non-relational focus					
473	Relationships between technique and bat speed, post-impact ball speed, and carry distance during a range hitting task in cricket	Peploe C.,McErlain-Naylor S.A.,Harland A.R.,King M.A.	2019	Scopus	S3	EXCLUDE	EXCLUDE	Off-topic: physiological, technical, technological, or non-relational focus	mixed/unclear	P1, P2	TRUE	NOT ASSESSED	Off-topic: physiological, technical, technological, or non-relational focus					
474	The Development and Validation of the Adolescent Sport Drug Inventory (ASDI) Among Athletes From Four Continents	Nicholls A.R.,Meir R.,Jones L.,... Baghurst T.,Perry J.L.	2019	Scopus	S3	EXCLUDE	EXCLUDE	Population not eligible or elite status not established	mixed/unclear	P1, P2, P3	TRUE	NOT ASSESSED	Population not eligible or elite status not established					
475	Do coaching style and game circumstances predict athletes' perceived justice of their coach? A longitudinal study in elite handball and volleyball teams	De Backer M.,Reynders B.,Boen F.,Van Puyenbroeck S.,Broek G.V.	2018	Scopus	S3	INCLUDE	RETAIN	Potentially eligible; advanced to full-text or extended-abstract assessment	elite	P1, P2	TRUE	INCLUDE	Retained in narrative synthesis	Core	Phase II / III	Direct	9	High
476	Physiological, perceptual, and technical responses to continuous and intermittent small-sided games in lacrosse players	Hauer R.,Tessitore A.,Binder N.,Tschan H.	2018	Scopus	S3	EXCLUDE	EXCLUDE	Off-topic: physiological, technical, technological, or non-relational focus	elite	P1	TRUE	NOT ASSESSED	Off-topic: physiological, technical, technological, or non-relational focus					
477	Relationships between physical qualities and key performance indicators during matchplay in senior international rugby union players	Cunningham D.J.,Shearer D.A.,Drawer S.,... Russell M.,Kilduff L.P.	2018	Scopus	S3	EXCLUDE	EXCLUDE	No eligible intersection of coaching/relationship/competitive psychological constructs	elite	P1, P2	TRUE	NOT ASSESSED	No eligible intersection of coaching/relationship/competitive psychological constructs					
478	Positive youth development and observed athlete behavior in recreational sport	Vierimaa M.,Bruner M.W.,Cote J.	2018	Scopus	S3	EXCLUDE	EXCLUDE	Population not eligible or elite status not established	mixed/unclear	P1, P2, P3	TRUE	NOT ASSESSED	Population not eligible or elite status not established					
479	Concussion Knowledge and Communication Behaviors of Collegiate Wrestling Coaches	Kroshus E.,Kerr Z.Y.,DeFreese J.D.,Parsons J.T.	2017	Scopus	S3	EXCLUDE	EXCLUDE	Population not eligible or elite status not established	mixed/unclear	P1, P2	TRUE	NOT ASSESSED	Population not eligible or elite status not established					
480	A longitudinal examination of stressors, appraisals, and coping in youth swimming	Hayward F.P.I.,Knight C.J.,Mellalieu S.D.	2017	Scopus	S3	MAYBE	RETAIN	Potentially eligible; advanced to full-text or extended-abstract assessment	mixed/unclear	P1, P2, P3	TRUE	EXCLUDE_AT_ELIGIBILITY	Elite/professional status not confirmed					
481	Patterns of nutrition and dietary supplements use in young Egyptian athletes: A community-based cross-sectional survey	Tawfik S.,El Koofy N.,Moawad E.M.I.	2016	Scopus	S3	EXCLUDE	EXCLUDE	Population not eligible or elite status not established	mixed/unclear	P1, P3	TRUE	NOT ASSESSED	Population not eligible or elite status not established					

Citation	Title	Authors	Journal	Year	DOI	Databases	Tier	Pillars	Primary phase	Phase evidence	Design	Population relevance (0-2)	Construct integration (0-2)	Temporal alignment (0-2)	Design strength (0-2)	Reporting adequacy (0-2)	Total (0-10)	Evidence weight	Interpretive note
Fryer et al., 2026	Coaching under pressure in Australian high-performance sport: A qualitative study of coach mental performance	Fryer, RP; Mallett, CJ; Kirby, JN; Kashdan, TB	INTERNATIONAL JOURNAL OF SPORTS SCIENCE & COACHING	2026	10.1177/17479541261430313	WoS	**Core**	P1; P3	Phase III	Direct	Qualitative interviews	2	1	2	1	2	8	High	Major-competition coach mental performance
Ferguson & Mason, 2026	Olympic swimmers' perspectives on how they want to be led	Ferguson, C; Mason, C	INTERNATIONAL JOURNAL OF SPORTS SCIENCE & COACHING	2026	10.1177/17479541261446432	WoS	**Core**	P1; P2	Cross-cutting	Indirect	Qualitative interviews	2	1	1	1	2	7	Moderate	Leadership preferences in hybrid individual/team structure
Braga-Pereira et al., 2026	Unveiling the coach-athlete relationship in Major international competitions: A qualitative study of national team coaches	Braga-Pereira, R; Bernardo-Ramos, J; Castro-Vigário, P; Duarte, D; Teques, P	INTERNATIONAL JOURNAL OF SPORTS SCIENCE & COACHING	2026	10.1177/17479541261431543	WoS	**Core**	P2; P3	Phase III	Direct	Qualitative interviews	2	1	2	1	2	8	High	Relationship management at major competitions
Davis et al., 2026	Development and evaluation of a coach-athlete relationship intervention program	Davis, L; Sommer, M; Brown, DJ; Jowett, S; Ekelund, R; Mårs, T; Wachsmuth, S	PSYCHOLOGY OF SPORT AND EXERCISE	2026	10.1016/j.psychsport.2026.103141	Scopus+WoS	**Core**	P1; P2	Phase II	Direct	Nonrandomized cluster-controlled mixed methods	1	1	2	2	2	8	High	Repeated follow-up during intervention
Henriksen et al., 2025	The coach as an architect of Danish high- performance sport environments	Henriksen, K; Dideriksen, S; Kuettel, A; Schlawe, A; Storm, LK	PSYCHOLOGY OF SPORT AND EXERCISE	2025	10.1016/j.psychsport.2025.102877	Scopus+WoS	**Core**	P1; P2	Phase I	Indirect	Multiple-case qualitative study	2	1	1	1	2	7	Moderate	Environment construction; pre-season placement is interpretive
Ulaş et al., 2025	Qualitative analysis of elite football players' causal attribution to not playing: A perspective of attribution theory	Ulas M.,Adilogullari I.,Senel E.	Plos One	2025		Scopus	**Core**	P1; P2; P3	Phase II / relational aftermath	Indirect	Qualitative thematic analysis	2	2	1	1	2	8	High	Non-selection, communication, motivation
González-García et al., 2024	The mediating roles of pre-competitive coping and affective states in the relationships between coach-athlete relationship, satisfaction and attainment of achievement goals	González-García, H; Martinent, G; Nicolas, M	INTERNATIONAL JOURNAL OF SPORT AND EXERCISE PSYCHOLOGY	2024	10.1080/1612197X.2023.2190346	SPORTDiscus+WoS	**Core**	P2; P3	Phase III	Direct	Three-point prospective design	1	1	2	2	2	8	High	Pre-, during-, and post-competition sequence
Karayel et al., 2024	The role of transformational leadership in the associations between coach-athlete relationship and team resilience: A study on elite football players	Karayel, E; Adilogullari, I; Senel, E	BMC PSYCHOLOGY	2024	10.1186/s40359-024-02043-7	SPORTDiscus+Scopus+WoS	**Core**	P1; P2; P3	Phase II	Indirect	Cross-sectional correlational study	2	2	1	1	2	8	High	Team resilience and transformational leadership
Slade et al., 2024	Developing guidelines for selection-deselection in high performance sport for athletes, coaches, and organisations: A delphi study	Slade, K; Jowett, S; Rhind, D	JOURNAL OF SPORTS SCIENCES	2024	10.1080/02640414.2024.2387968	Scopus+WoS	**Core**	P1; P2	Selection/transition	Indirect	Delphi study	2	1	1	1	2	7	Moderate	Consensus guidelines; not athlete lived-experience interviews
Cece et al., 2022	Athletes' motivational and emotional outcomes related to a Need-Supportive intervention in intensive training centers	Cece, V; Guillet-Descas, E; Tessier, D; Martinent, G	JOURNAL OF APPLIED SPORT PSYCHOLOGY	2022	10.1080/10413200.2021.1941425	Scopus+WoS	**Core**	P1; P3	Phase II	Direct	Longitudinal field intervention	2	1	2	2	2	9	High	Twice-monthly logbooks during competitive season
Robazza et al., 2022	Perceived motivational climate influences athletes' emotion regulation strategies, emotions, and psychobiosocial experiences	Robazza, C; Moran, M; Bortoli, L; Ruiz, MC	PSYCHOLOGY OF SPORT AND EXERCISE	2022	10.1016/j.psychsport.2021.102110	Scopus+WoS	**Core**	P1; P3	Phase II	Indirect	Cross-sectional SEM	1	1	1	1	2	6	Moderate	Phase placement is conceptual
da Silva et al., 2022	Quality of coach-athlete relationship and coping as associated factors of stress, anxiety, burnout, and depression symptoms of soccer players in transition to professional: a prospective study	da Silva A.A.,de Sousa Fortes L.,da Cruz Carvalho L.P.,... do Nascimento Junior J.R.A.,Barbieri R.A.	Motriz Revista De Educacao Fisica	2022		Scopus	**Core**	P2; P3	Phase II	Direct	Prospective beginning-to-end season study	2	1	2	2	2	9	High	Elite-development transition sample
Kim & Park, 2020	"Coach really knew what i needed and understood me well as a person": Effective communication acts in coach–athlete interactions among Korean olympic archers	Kim Y.,Park I.	International Journal of Environmental Research and Public Health	2020		Scopus	**Core**	P1; P2; P3	Phase III	Direct	Qualitative interviews	2	2	2	1	2	9	High	Training, competition, and crisis communication
Moreira da Silva et al., 2021	Which are the most determinant psychological factors in olympic shooting performance? A self-perspective from elite shooters	Moreira da Silva F.,Malico Sousa P.,Pinheiro V.B.,... Refoyo Roman I.,Mon-Lopez D.	International Journal of Environmental Research and Public Health	2021		Scopus	**Core**	P1; P3	Phase III	Indirect	Cross-sectional elite-athlete self-report	2	1	1	1	2	7	Moderate	Coach support and precompetition preparation
Miller et al., 2020	Coach identity leadership behaviours are positively associated with athlete resource appraisals: The mediating roles of relational and group identification	Miller, AJ; Slater, MJ; Turner, MJ	PSYCHOLOGY OF SPORT AND EXERCISE	2020	10.1016/j.psychsport.2020.101755	Scopus+WoS	**Core**	P1; P2; P3	Phase III	Direct	Cross-sectional plus start/end-season prospective study	2	2	2	2	2	10	High	Identity leadership and resource appraisals
Balk et al., 2019	Antecedents and consequences of perceived autonomy support in elite sport: A diary study linking coaches' off-job recovery and athletes' performance satisfaction	Balk, YA; de Jonge, J; Geurts, SAE; Oerlemans, WGM	PSYCHOLOGY OF SPORT AND EXERCISE	2019	10.1016/j.psychsport.2019.04.020	SPORTDiscus+WoS	**Core**	P1; P3	Phase II	Direct	Daily diary study	2	1	2	2	2	9	High	Day-level coach recovery and athlete experience
Cheval et al., 2017	How perceived autonomy support and controlling coach behaviors are related to well- and ill-being in elite soccer players: A within-person changes and between-person differences analysis	Cheval, B; Chalabaev, A; Quested, E; Courvoisier, DS; Sarrazin, P	PSYCHOLOGY OF SPORT AND EXERCISE	2017	10.1016/j.psychsport.2016.10.006	Scopus+WoS	**Core**	P1; P3	Phase II	Direct	Three-wave repeated-measures study	2	1	2	2	2	9	High	Within-person change across final season months
Nicholls et al., 2017	Perceptions of the Coach-Athlete Relationship Predict the Attainment of Mastery Achievement Goals Six Months Later: A Two-Wave Longitudinal Study among F. A. Premier League Academy Soccer Players	Nicholls, AR; Earle, K; Earle, F; Madigan, DJ	FRONTIERS IN PSYCHOLOGY	2017	10.3389/fpsyg.2017.00684	SPORTDiscus+WoS	**Core**	P2; P3	Phase I	Direct	Two-wave six-month longitudinal study	2	1	2	2	2	9	High	Early relationship quality and later mastery goals
Ntoumanis et al., 2017	Linking Coach Interpersonal Style With Athlete Doping Intentions and Doping Use: A Prospective Study	Ntoumanis, N; Barkoukis, V; Gucciardi, DF; Chan, DKC	JOURNAL OF SPORT & EXERCISE PSYCHOLOGY	2017	10.1123/jsep.2016-0243	Scopus+WoS	**Core**	P1; P3	Phase I	Direct	Prospective study	1	1	2	2	2	8	High	Baseline interpersonal style and later doping outcomes
Kavanagh et al., 2017	Elite Athletes' Experience of Coping With Emotional Abuse in the Coach-Athlete Relationship	Kavanagh, E; Brown, L; Jones, I	JOURNAL OF APPLIED SPORT PSYCHOLOGY	2017	10.1080/10413200.2017.1298165	SPORTDiscus+Scopus+WoS	**Core**	P2; P3	Relational aftermath	Indirect	Retrospective qualitative study	2	1	1	1	2	7	Moderate	Long-term sense-making; not a post-season study
De Backer et al., 2018	Do coaching style and game circumstances predict athletes' perceived justice of their coach? A longitudinal study in elite handball and volleyball teams	De Backer M.,Reynders B.,Boen F.,Van Puyenbroeck S.,Broek G.V.	Plos One	2018		Scopus	**Core**	P1; P2	Phase II / III	Direct	Six-week repeated-game longitudinal study	2	1	2	2	2	9	High	Game-to-game fluctuations in perceived justice
Schlawe et al., 2026	Features of successful high-performance sport environments: Stakeholder insights	Schlawe, A; Christiansen, AV; Henriksen, K	JOURNAL OF APPLIED SPORT PSYCHOLOGY	2026	10.1080/10413200.2026.2683956	WoS	Contextual	P1; P2	Cross-cutting	Indirect	Qualitative stakeholder study	2	1	1	1	2	7	Moderate	Organizational moderator
Tjønndal & Haugen, 2024	Care as a strategy to navigate political tensions within the China-Norway sports coaching collaboration	Tjonndal, A; Haugen, HO	INTERNATIONAL REVIEW FOR THE SOCIOLOGY OF SPORT	2024	10.1177/10126902241247579	Scopus+WoS	Contextual	P1; P2	Cross-cutting	Indirect	Qualitative interviews	2	1	1	1	2	7	Moderate	Cultural and political context
Levillain et al., 2025	Longitudinal relationships between emotional processes and perceived performances among athletes: The moderating effect of emotional intelligence	Levillain G.,Martinent G.,Saby Y.,Nicolas M.	Performance Enhancement and Health	2025		Scopus	Contextual	P3	Phase III	Direct	Five-competition longitudinal study	1	0	2	2	2	7	Moderate	Competition-psychology track only
Levillain et al., 2022	Longitudinal trajectories of emotions among athletes in sports competitions: Does emotional intelligence matter?	Levillain, G; Martinent, G; Vacher, P; Nicolas, M	PSYCHOLOGY OF SPORT AND EXERCISE	2022	10.1016/j.psychsport.2021.102012	Scopus+WoS	Contextual	P3	Phases II–III	Direct	Three-wave seasonal longitudinal study	1	0	2	2	2	7	Moderate	Competition-psychology track only
Verner-Filion & Vallerand, 2018	A longitudinal examination of elite youth soccer players: The role of passion and basic need satisfaction in athletes' optimal functioning	Verner-Filion, J; Vallerand, RJ	PSYCHOLOGY OF SPORT AND EXERCISE	2018	10.1016/j.psychsport.2018.07.005	Scopus+WoS	Contextual	P3	Phase II	Direct	Multi-season longitudinal study	2	0	2	2	2	8	High	Motivational process; coach behavior not directly measured
Dehghansai et al., 2020	Olympic and Paralympic coach perspectives on effective skill acquisition support and coach development	Dehghansai, N; Headrick, J; Renshaw, ; Pinder, RA; Barris, S	SPORT EDUCATION AND SOCIETY	2020	10.1080/13573322.2019.1631784	Scopus+WoS	Contextual	P1	Cross-cutting	Indirect	Qualitative coach-perspective study	2	0	1	1	2	6	Moderate	Coach development and adaptation
Poucher et al., 2018	Providing social support to female Olympic athletes	Poucher Z.A.,Tamminen K.A.,Kerr G.	Journal of Sport and Exercise Psychology	2018		Scopus	Contextual	P2	Cross-cutting	Indirect	Paired qualitative interviews	2	0	1	1	2	6	Moderate	Gendered support provision
Kegelaers & Wylleman, 2019	Exploring the Coach's Role in Fostering Resilience in Elite Athletes	Kegelaers, J; Wylleman, P	SPORT EXERCISE AND PERFORMANCE PSYCHOLOGY	2019	10.1037/spy0000151	WoS	Contextual	P1; P3	Cross-cutting	Indirect	Qualitative study	2	1	1	1	2	7	Moderate	Proactive and reactive resilience support
Carleton et al., 2016	Scarred for the Rest of My Career? Career-Long Effects of Abusive Leadership on Professional Athlete Aggression and Task Performance	Carleton, EL; Barling, J; Christie, AM; Trivisonno, M; Tulloch, K; Beauchamp, MR	JOURNAL OF SPORT & EXERCISE PSYCHOLOGY	2016	10.1123/jsep.2015-0333	Scopus+WoS	Contextual	P1; P3	Relational aftermath	Direct	Longitudinal archival multilevel study	2	1	2	2	2	9	High	Career-long carryover of abusive leadership

ID	Title	Authors	Year	Databases	Pillars	Population	Temporal marker	Exclusion reason
3	Positive Experiences Among Elite Athletes in Finland: Associations With Basic Psychological Needs, Sport Conditions, and Background Factors	Kaski, SS; Kinnunen, U	2026	WoS	P2	elite	TRUE	Did not examine the intersection of at least two construct pillars
4	Understanding coach burnout in high-performance sport: the sequential effects of knowledge, psychological safety, and coach-athlete relationships	Tao, RC; Zhang, NY; Guo, C	2026	SPORTDiscus+WoS	P2	unspecified	TRUE	Did not examine the intersection of at least two construct pillars
5	Exploring stressors in elite rugby league (NRL/NRLW) players	Humphreys, C; Goddard, SG; Poulus, D	2026	WoS	P2	elite	TRUE	Did not examine the intersection of at least two construct pillars
6	Exploring the relationship between burnout and mental health in elite athletes: the role of moderators	de Subijana, CL; Wergin, VV; Mallett, CJ; Haslam, A	2026	WoS	P2	elite	TRUE	Did not examine the intersection of at least two construct pillars
7	Why do elite athletes consider leaving their sports? Longitudinal evidence on the development of dropout considerations	Blessing, J; Hilpisch, C; Krüger, K; Raab, M; Wiese, L; Zentgraf, K; Mutz, M	2026	WoS	P2	elite	TRUE	Did not examine the intersection of at least two construct pillars
8	I even called him coach at home: A qualitative study on role conflict and coping in parent-coached elite athletes	Alp, AF; Karagün, E	2026	WoS	P3	elite	TRUE	Did not examine the intersection of at least two construct pillars
10	Shame following performance failure as a potential mediator between sport contingent self-esteem and both competitive anxiety and burnout among elite athletes	Funke, A; Gustafsson, H	2026	WoS	P3	unspecified	TRUE	Did not examine the intersection of at least two construct pillars
11	Leadership micro-behaviors and performance stability under competitive pressure: a stepped-wedge field study in professional football	Paasch, R; Mau, G	2026	SPORTDiscus+WoS	P3	elite	TRUE	Did not examine the intersection of at least two construct pillars
12	Point-ending outcome patterns in break-point situations in professional tennis: Effects of player position and court surface	Mourtzios, C; Gouvelis, N; Mourtziou, MA	2026	WoS	P3	elite	TRUE	Did not examine the intersection of at least two construct pillars
13	Acute weight cycling and hydration status in elite kickboxers: a repeated-measures field study	Ceylan, B; Taskin, HB; Sarikaya, U; Pekel, HA; Sabirli, TN	2026	Scopus+WoS	P3	elite	TRUE	Did not examine the intersection of at least two construct pillars
14	Workload and travel demands of professional basketball staff: A season-long case study across the National Basketball Association and G League	Mercer, RAJ; Russell, JL; Strack, DS; Coutts, AJ; McLean, BD	2026	WoS	P3	unspecified	TRUE	Did not examine the intersection of at least two construct pillars
16	Applying the 5Cs Framework to Elite Youth Tennis: Impact Factors in a Talent Development Environment	Harwood, C; Porter, K	2026	Scopus+WoS	P1, P2	elite	TRUE	Insufficient fit with the temporal cascade after eligibility assessment
17	Understanding the motivators and barriers of elite athletes as role models	Praet, L; De Bosscher, V; Smismans, S; Derom, I	2025	Scopus+WoS	P1	unspecified	TRUE	Did not examine the intersection of at least two construct pillars
18	Exploring the barriers to athlete personal development within UK Olympic and Paralympic sport	Holden, J; Wagstaff, CRD; Wadey, R; Brown, P	2025	WoS	P1	unspecified	TRUE	Did not examine the intersection of at least two construct pillars
19	Evaluating a behaviour change intervention to enhance athlete monitoring system engagement: Insights from elite sport	Neupert, EC; Holder, T; Jobson, SA	2025	WoS	P1	elite	TRUE	Did not examine the intersection of at least two construct pillars
20	Disciplinary strategies or reactive measures? An analysis of coach sanctions in elite Danish handball leagues	Gençoglu, C; Gümüs, H	2025	WoS	P1	elite	TRUE	Did not examine the intersection of at least two construct pillars
21	The epidemiology of injuries in professional sitting volleyball athletes	Mahmoudkhani, M; Alizadeh, F; Khodsiyani, E; Norouzi, M	2025	Scopus+WoS	P1	elite	TRUE	Did not examine the intersection of at least two construct pillars
22	Burnout in medical and performance professionals working in elite football in the United Kingdom: a pilot study	Mifsud, D; Acampora, N; Rao, NL; Shafik, A; Carmody, S; Chandran, A	2025	WoS	P1	elite	TRUE	Did not examine the intersection of at least two construct pillars
23	A Longitudinal Study of Organizational Stressors and Mental Health in the Irish Olympic Team Before and After the Tokyo 2020 (2021) Olympic Games	Browne, JA; Paradis, KF; Kirby, K; Arnold, R	2025	WoS	P1	elite	TRUE	Did not examine the intersection of at least two construct pillars
24	Optimizing recovery: the impact of training load in elite volleyball players	Rebelo, A; Pereira, JR; Vande Broek, G; Coelho-e-Silva, MJ; Elferink-Gemser, MT; Valente-dos-Santos, J	2025	WoS	P1	elite	TRUE	Did not examine the intersection of at least two construct pillars
25	Examining the relationship of training load and menstrual cycle on a web application-based load and recovery score: A longitudinal observational study in elite women's soccer	Brefin, SL; Anderegg, J; Nigg, CR; Koschnick, D; Paul, C; Ketelhut, S	2025	WoS	P1	elite	TRUE	Did not examine the intersection of at least two construct pillars
26	Evidence for the reversal of the relative age effect in European professional basketball	Akinci, Y; Yapar, A	2025	WoS	P1	elite	TRUE	Did not examine the intersection of at least two construct pillars
27	Influence of Mental Skills on Competitive Anxiety in Professional Futsal Players: Difference by Successful and Unsuccessful Players.	Kemarat, Supatcharin ; Theanthong, Apiluk ; Yeemin, Wichai ; Suwankan, Sutima ; Makaje, Niromlee	2025	SPORTDiscus	P2, P3	elite	FALSE	No direct or phase-relevant temporal content
29	Resilience profiles of elite athletes and their associations with health-related behaviors, well-being, and performance: A latent profile analysis	Chretien, A; Hayotte, M; Vuillemin, A; Longueville, FD	2024	Scopus+WoS	P2	elite	FALSE	Did not examine the intersection of at least two construct pillars
30	How is coaches' social identity leadership related to mental health in elite athletes? The mediating role of satisfaction with performance	de Subijana, CL; Pons, J; Mallett, CJ	2024	Scopus+WoS	P2	elite	FALSE	Did not examine the intersection of at least two construct pillars
31	The successful experience of gymnastics world champion coach: an interview analysis	Liu, XX; Weng, XH; Qin, HH; Ma, S; Wang, GQ	2024	WoS	P2	elite	FALSE	Did not examine the intersection of at least two construct pillars
32	Determinants of motivation in world-class musicians and Olympic athletes: Exploring the front and the back side of the medallion	Hatfield, JL	2024	WoS	P2	elite	FALSE	Did not examine the intersection of at least two construct pillars
33	Factor analysis on the performance of elite male fencing athletes	Kim, HC; Park, KJ	2024	Scopus+WoS	P2	elite	FALSE	Did not examine the intersection of at least two construct pillars
34	Orienteering Is More than Just Running! Acute Effect of Competitive Pressure on Autonomic Cardiac Activity among Elite Orienteering Athletes	Gorgulu, R; Oruç, H; Vasile, C; Corlaci, I; Voinea, F	2024	SPORTDiscus+Scopus+WoS	P2	elite	FALSE	Did not examine the intersection of at least two construct pillars

36	The use of technology in the assessment and monitorization of a season in a professional padel player: A case study	Carnero-Diaz, A; Pecci, J; Reverte-Pagola, G; Da Silva-Grigoletto, ME	2024	WoS	P2	elite	FALSE	Did not examine the intersection of at least two construct pillars
37	The Role of Athlete Leadership Quality in the Characteristics of Team Resilience in Elite Soccer Teams: A Cross-Sectional and Longitudinal Mediation of Team Identification	López-Gajardo, MA; González-Ponce, I; García-Calvo, T; Enrich-Alturo, E; Leo, FM	2024	WoS	P2	elite	FALSE	Did not examine the intersection of at least two construct pillars
38	The Inducers of an Elite Male Table Tennis Player's Emotional Experience Throughout His Career: A Single Case Study Based on the Critical-Incident Method	Petiot, O; Kermarrec, G; Visioli, J; Martin, G	2024	WoS	P2	elite	FALSE	Did not examine the intersection of at least two construct pillars
39	Integration of golf practise and strength and conditioning in golf: Insights from professional golf coaches	Bliss, A; Langdown, B	2024	WoS	P2	elite	FALSE	Did not examine the intersection of at least two construct pillars
40	PRE-COMPETITION TAPERING IN HIGH PERFORMANCE SPORTS -- A THEORETICAL APPROACH.	NEGRU, Ioan Niculaie ; NUŢ, Ramona Ancuţa	2024	SPORTDiscus	P2	elite	FALSE	Did not examine the intersection of at least two construct pillars
41	Coach-athlete relationship and burnout symptoms among young elite athletes and the role of mental toughness as a moderator.	Gerber, Markus ; Gygax, Basil ; Cody, Robyn	2024	SPORTDiscus	P2	elite	FALSE	Did not examine the intersection of at least two construct pillars
42	The under representation of women coaches in high-performance sport	Serpell, BG; Harrison, D; Dower, R; Cook, CJ	2023	WoS	P2	elite	FALSE	Did not examine the intersection of at least two construct pillars
43	Interpersonal Behaviors Questionnaire in Sport: Psychometric Analysis With Romanian Professional Athletes	Alexe, DI; Cîrtita, BC; Tohanean, DI; Larion, A; Alexe, CI; Dragos, P; Burgueño, R	2023	Scopus+WoS	P3	elite	FALSE	Did not examine the intersection of at least two construct pillars
44	The impact of training environment on Tokyo Olympic athletes' wellbeing: a qualitative multiple case study	Paoletti, V; Mayglothling, T; Mills, JP	2023	WoS	P3	elite	FALSE	Did not examine the intersection of at least two construct pillars
45	Inescapable tensions: performance and/or psychological well-being in Olympic and Paralympic athletes during sport disruption	Trainor, LR; Bennett, EV; Bundon, AM; Tremblay, M; Mannella, S; Crocker, PRE	2023	WoS	P3	elite	FALSE	Did not examine the intersection of at least two construct pillars
46	Lifestyle challenges and mental health of professional tennis players: an exploratory case study	Shrom, SJ; Cumming, J; Fenton, SJ	2023	WoS	P3	elite	FALSE	Did not examine the intersection of at least two construct pillars
47	Heart Rate Variability in Elite International ITF Junior Davis Cup Tennis Players	Villafaina, S; Crespo, M; Martinez-Gallego, R; Fuentes-Garcia, JP	2023	WoS	P3	elite	FALSE	Did not examine the intersection of at least two construct pillars
48	Balancing risk-taking and self-care: The ecology of athlete health behaviour during the Olympic qualification phase	Schubring, A; Hallten, M; Barker-Ruchti, N; Post, A	2023	WoS	P3	elite	FALSE	Did not examine the intersection of at least two construct pillars
49	Thinking About Elite Performance: The Experience and Impact of Mental Fatigue in Elite Sport Coaching	Russell, S; Halson, SL; Jenkins, DG; Rynne, SB; Roelands, B; Kelly, VG	2023	WoS	P3	elite	FALSE	Did not examine the intersection of at least two construct pillars
50	Are Metabolic Power Distribution and Accelerometer-Based Global Positioning System Variables Associated With Odds Ratios of Noncontact Injuries in Professional Soccer Players?	Nobari, H; Alves, AR; Abbasi, H; Khezri, D; Zamorano, AD; Bowman, TG	2023	WoS	P3	elite	FALSE	Did not examine the intersection of at least two construct pillars
51	Multilevel Analysis of Coach Leadership, Group Cohesion and Collective Efficacy in Semiprofessional Football Teams	Leo, FM; González-Ponce, ; Pulido, JJ; López-Gajardo, MA; García-Calvo, T	2022	WoS	P3	elite	FALSE	Did not examine the intersection of at least two construct pillars
52	Desired coaching behaviours of elite divers during competition	Henderson, S; Bloom, GA; Alexander, D	2022	WoS	P3	elite	FALSE	Did not examine the intersection of at least two construct pillars
53	An Evidence-Informed Framework to Promote Mental Wellbeing in Elite Sport	Purcell, R; Pilkington, V; Carberry, S; Reid, D; Gwyther, K; Hall, K; Deacon, A; Manon, R; Walton, CC; Rice, S	2022	WoS	P3	elite	FALSE	Did not examine the intersection of at least two construct pillars
54	Well-being in elite sport: The specific role of sport-related demands, resources, and recovery from sport	Balk, YA; de Jonge, J; Geurts, SAE	2022	WoS	P3	elite	FALSE	Did not examine the intersection of at least two construct pillars
55	Predicting sports performance from well-being: A mapping of professional athletes', amateur athletes' and non-athletes' positions	Fruchart, E; Rulence-Pâques, P	2022	WoS	P3	unspecified	FALSE	Did not examine the intersection of at least two construct pillars
56	Orientation and motivational climate in elite handball players: Multivariate modeling of performance	Kristjánsdóttir, H; Matthíasdóttir, A; Saavedra, JM	2022	SPORTDiscus+WoS	P3	elite	FALSE	Did not examine the intersection of at least two construct pillars
57	Creating an environment for thriving: An ethnographic exploration of a British decentralised Olympic and Paralympic Sport Organisation	Passaportis, MJR; Brown, DJ; Wagstaff, CRD; Arnold, R; Hays, K	2022	WoS	P3	elite	FALSE	Did not examine the intersection of at least two construct pillars
58	Satisfaction with life in Special Olympic athletes: the role of autonomy support and basic need fulfilment	Komenda, S; Springstein, T; Zrnic, I; Zeilinger, E; Franken, F; Weber, G	2022	Scopus+WoS	P3	elite	FALSE	Did not examine the intersection of at least two construct pillars
59	Stakeholder insights into athlete attrition in the high-performance pathway	Guevara, SA; Donaldson, A; Keegan, RJ; Appaneal, RN; Smyth, EA; Waddington, G; Mahony, K; Drew, MK	2022	Scopus+WoS	P3	elite	FALSE	Did not examine the intersection of at least two construct pillars
60	Rapid weight loss among elite-level judo athletes: methods and nutrition in relation to competition performance	Stangar, M; Stangar, A; Shtyrba, V; Cigic, B; Benedik, E	2022	Scopus+WoS	P1, P2	elite	FALSE	No direct or phase-relevant temporal content
61	The prevalence of pre-conditioning and recovery strategies in senior elite and non-elite amateur boxing	Finlay, MJ; Page, RM; Greig, M; Bridge, CA	2022	Scopus+WoS	P1, P2	elite	FALSE	No direct or phase-relevant temporal content
62	An exploration of reciprocity between female athletes and their coach in elite junior swimming: a shared reality theory perspective	Solstad, BE; Granerud, M; Haraldsen, HM; Gustafsson, H; Knight, CJ	2022	WoS	P1, P2	elite	FALSE	No direct or phase-relevant temporal content
63	Mindful self-reflection to support sustainable high-performance coaching: A process evaluation of a novel method development in elite sport	Hägglund, K; Kenttä, G; Thelwell, R; Wagstaff, CRD	2022	Scopus+WoS	P1, P2	elite	FALSE	No direct or phase-relevant temporal content
64	Stroke Kinematics, Temporal Patterns, Neuromuscular Activity, Pacing and Kinetics in Elite Breaststroke Swimming: A Systematic Review	Nicol, E; Pearson, S; Saxby, D; Minahan, C; Tor, E	2022	WoS	P1, P2	elite	FALSE	No direct or phase-relevant temporal content
65	The quest for professional self-understanding: Sense making and the interpersonal nature of applied sport psychology practice	Haluch, P; Radcliffe, J; Rowley, C	2022	Scopus+WoS	P1, P3	elite	FALSE	No direct or phase-relevant temporal content

66	Implementing Skill Acquisition Research in High-Performance Sport: Reflecting on the Importance of Autonomy-Support for Successful Collaboration.	Morris-Binelli, Khaya ; Müller, Sean ; van Rens, Fleur E. C. A. ; Staniforth, Dave ; Appleby, Brendyn ; Rosalie, Simon M.	2022	SPORTDiscus	P1, P3	elite	FALSE	No direct or phase-relevant temporal content
67	A cluster analysis of high-performance female team players' perceived motivational climate: Implications on perceived motor competence and autonomous behaviour.	Abraldes, J Arturo ; Conte Marín, Luis ; Manzano-Sánchez, David ; Gómez-López, Manuel ; Sánchez-Alcaraz, Bernardino J	2022	SPORTDiscus+Scopus	P1, P3	elite	FALSE	No direct or phase-relevant temporal content
68	Dark traits as a potential feature of leadership in the high-performance sports coach	Serpell, BG; Harrison, D; Lyons, M; Cook, CJ	2021	WoS	P1, P3	elite	FALSE	No direct or phase-relevant temporal content
69	The podium illusion: a phenomenological study of the influence of social support on well-being and performance in elite para swimmers	Aitchison, B; Rushton, AB; Martin, P; Soundy, A; Heneghan, NR	2021	WoS	P1	elite	FALSE	Did not examine the intersection of at least two construct pillars
70	Psychosocial functioning of Olympic coaches and its perceived effect on athlete performance: a systematic review	Cook, GM; Fletcher, D; Carroll, C	2021	WoS	P1	elite	FALSE	Did not examine the intersection of at least two construct pillars
71	A repeated-measures examination of organizational stressors, perceived psychological and physical health, and perceived performance in semi-elite athletes	Simms, M; Arnold, R; Turner, JE; Hays, K	2021	WoS	P1	elite	FALSE	Did not examine the intersection of at least two construct pillars
72	A Field-Based Approach to Determine Soft Tissue Injury Risk in Elite Futsal Using Novel Machine Learning Techniques	Ruiz-Pérez, I; López-Valenciano, A; Hernández-Sánchez, S; Puerta-Callejón, JM; Croix, MD; de Baranda, PS; Ayala, F	2021	WoS	P1	elite	FALSE	Did not examine the intersection of at least two construct pillars
73	Longitudinal Physiological and Fitness Evaluations in Elite Ice Hockey: A Systematic Review	Chiarlitti, NA; Crozier, M; Insogna, JA; Reid, RER; Delisle-Houde, P	2021	WoS	P1	elite	FALSE	Did not examine the intersection of at least two construct pillars
74	The influence of macrocultural change on national governing bodies in British olympic sports	Feddersen, NB; Morris, R; Abrahamsen, FE; Littlewood, MA; Richardson, DJ	2021	WoS	P1	elite	FALSE	Did not examine the intersection of at least two construct pillars
75	A Longitudinal Study of Power Relations in a British Olympic Sport Organization	Feddersen, NB; Morris, R; Storm, LK; Littlewood, MA; Richardson, DJ	2021	WoS	P1	elite	FALSE	Did not examine the intersection of at least two construct pillars
76	Athlete perspectives on the enablers and barriers to nutritional adherence in high-performance sport	Bentley, MRN; Patterson, LB; Mitchell, N; Backhouse, SH	2021	Scopus+WoS	P1	elite	FALSE	Did not examine the intersection of at least two construct pillars
77	More than just another bib: group dynamics in an elite Nordic ski team	Cascagnette, JCW; Benson, AJ; Cruwys, T; Haslam, SA; Bruner, MW	2021	WoS	P1	elite	FALSE	Did not examine the intersection of at least two construct pillars
78	Positive youth development and gender differences in high performance sport	O'Connor, D; Gardner, L; Larkin, P; Pope, A; Williams, AM	2020	Scopus+WoS	P1	elite	FALSE	Did not examine the intersection of at least two construct pillars
80	Enhancement of gratitude in the context of elite athletes: outcomes and challenges	Howells, K; Fitzallen, N	2020	WoS	P1	elite	FALSE	Did not examine the intersection of at least two construct pillars
81	The 5C's Model of Successful Partnerships in Elite Beach Volleyball Dyads	Poczwardowski, A; Lamphere, B; Allen, K; Marican, R; Haberl, P	2020	Scopus+WoS	P1	elite	FALSE	Did not examine the intersection of at least two construct pillars
82	Interplay of motivation and self-regulation throughout the development of elite athletes	Jordalen, G; Lemyre, PN; Durand-Bush, N	2020	WoS	P1	elite	FALSE	Did not examine the intersection of at least two construct pillars
83	Is the Acute: Chronic Workload Ratio (ACWR) Associated with Risk of Time-Loss Injury in Professional Team Sports? A Systematic Review of Methodology, Variables and Injury Risk in Practical Situations	Andrade, R; Wik, EH; Rebelo-Marques, A; Blanch, P; Whiteley, R; Espregueira-Mendes, J; Gabbett, TJ	2020	WoS	P1	elite	FALSE	Did not examine the intersection of at least two construct pillars
84	Does the longitudinal development of physical and anthropometric characteristics associate with professional career attainment in adolescent Australian footballers?	Cripps, AJ; Banyard, HG; Woods, CT; Joyce, C; Hopper, LS	2020	WoS	P1	elite	FALSE	Did not examine the intersection of at least two construct pillars
85	Development, Implementation, and Evaluation of anAthlete-Informed Mental Skills Training Program for EliteYouth Tennis Players	Dohme, LC; Bloom, GA; Piggott, D; Backhouse, S	2020	Scopus+WoS	P1	elite	FALSE	Did not examine the intersection of at least two construct pillars
86	Salivary IgA as a Predictor of Upper Respiratory Tract Infections and Relationship to Training Load in Elite Rugby Union Players	Tiernan, C; Lyons, M; Comyns, T; Nevill, AM; Warrington, G	2020	WoS	P1	elite	FALSE	Did not examine the intersection of at least two construct pillars
87	The emergence and perpetuation of a destructive culture in an elite sport in the United Kingdom	Feddersen, NB; Morris, R; Littlewood, MA; Richardson, DJ	2020	Scopus+WoS	P1	elite	FALSE	Did not examine the intersection of at least two construct pillars
88	Measurement Characteristics of Athlete Monitoring Tools in Professional Australian Football	Ryan, S; Pacecca, E; Tebble, J; Hocking, J; Kempton, T; Coutts, AJ	2020	WoS	P1	elite	FALSE	Did not examine the intersection of at least two construct pillars
89	A Pre-Match Video Self-Modeling Intervention in Elite Youth Football	Middlemas, S; Harwood, C	2020	WoS	P1	elite	FALSE	Did not examine the intersection of at least two construct pillars
90	Tapering practices of elite CrossFit athletes	Pritchard, HJ; Keogh, JW; Winwood, PW	2020	WoS	P1	elite	FALSE	Did not examine the intersection of at least two construct pillars
91	The Cognitive Component of Elite High Jumpers' Preperformance Routines	Gretton, T; Blom, L; Hankemeier, D; Judge, L	2020	WoS	P1	elite	FALSE	Did not examine the intersection of at least two construct pillars
92	Sports performers' perspectives on facilitating thriving in professional rugby contexts	Brown, DJ; Arnold, R	2019	WoS	P1	elite	FALSE	Did not examine the intersection of at least two construct pillars
93	A heuristic for the relationship between caring coaching and elite athlete performance	Fisher, LA; Larsen, LK; Bejar, MP; Shigeno, TC	2019	WoS	P3	unspecified	TRUE	Did not examine the intersection of at least two construct pillars
96	MOTIVATION IN THE PRACTICE OF JUDO IN NON-PROFESSIONAL ATHLETES	Castro-Sánchez, M; Zurita-Ortega, F; Zafra-Santos, E; Rodríguez-Fernández, S; Chacón-Cuberos, R; Valdivia-Moral, P	2019	WoS	P3	unspecified	FALSE	Did not examine the intersection of at least two construct pillars
97	Burnout symptoms and recovery processes in eight elite soccer coaches over 10 years	Hassmén, P; Kenttä, G; Hjälm, S; Lundkvist, E; Gustafsson, H	2019	WoS	P3	unspecified	FALSE	Did not examine the intersection of at least two construct pillars

98	It's not about sport, it's about you: An interpretative phenomenological analysis of mentoring elite athletes	Sandardos, SS; Chambers, TP	2019	WoS	P1	unspecified	TRUE	Did not examine the intersection of at least two construct pillars
99	Motivational processes affecting the perception of organizational and media stressors among professional football players: A longitudinal mixed methods research study	Kristiansen, E; Ivarsson, A; Solstad, BE; Roberts, GC	2019	WoS	P2	unspecified	TRUE	Did not examine the intersection of at least two construct pillars
100	The relationship of team and individual athlete performances on match quarter outcome in elite women's Australian Rules football	Cust, EE; Sweeting, AJ; Ball, K; Anderson, H; Robertson, S	2019	WoS	P2	unspecified	TRUE	Did not examine the intersection of at least two construct pillars
101	Training, match and non-rugby activities in elite male youth rugby union players in England	Hendricks, S; Till, K; Weaving, D; Powell, A; Kemp, S; Stokes, K; Jones, B	2019	WoS	P2	unspecified	TRUE	Did not examine the intersection of at least two construct pillars
102	Coaching Efficacy, Player Perceptions of Coaches' Leadership Styles, and Team Performance in Premier League Soccer	Keatlholetswe, L; Malete, L	2019	WoS	P1	unspecified	TRUE	Elite/professional status not confirmed
103	Elite female footballers' stories of sociocultural factors, emotions, and behaviours prior to anterior cruciate ligament injury	Ivarsson, A; Johnson, U; Karlsson, J; Börjesson, M; Hägglund, M; Andersen, MB; Waldén, M	2019	WoS	P1, P3	mixed	FALSE	No direct or phase-relevant temporal content
104	Effects of Self-Talk Training on Competitive Anxiety, Self-Efficacy, Volitional Skills, and Performance: An Intervention Study with Junior Sub-Elite Athletes.	Walter, Nadja ; Nikoleizig, Lucie ; Alfermann, Dorothee	2019	SPORTDiscus	P3	unspecified	FALSE	Did not examine the intersection of at least two construct pillars
105	Need-supportiveness and athlete well-being: Coaches' competence-support at risk in the elite sport context throughout the season.	Berntsen, Hedda ; Ivarsson, Andreas ; Kristiansen, Elsa	2019	SPORTDiscus	P3	unspecified	FALSE	Did not examine the intersection of at least two construct pillars
106	Optimal Development of Youth Athletes Toward Elite Athletic Performance: How to Coach Their Motivation, Plan Exercise Training, and Pace the Race.	Menting, Stein G P ; Hendry, David T ; Schiphof-Godart, Lieke ; Elferink-Gemser, Marije T ; Hettinga, Florentina J	2019	SPORTDiscus	P3	unspecified	FALSE	Did not examine the intersection of at least two construct pillars
108	Champions are racers, not pacers: an analysis of qualification patterns of Olympic and IAAF World Championship middle distance runners	Hanley, B; Hettinga, FJ	2018	WoS	P2	unspecified	TRUE	Did not examine the intersection of at least two construct pillars
112	Managing player load in professional rugby union: a review of current knowledge and practices	Quarrie, KL; Raftery, M; Blackie, J; Cook, CJ; Fuller, CW; Gabbett, TJ; Gray, AJ; Gill, N; Hennessy, L; Kemp, S; Lambert, M; Nichol, R; Mellalieu, SD; Piscione, J; Stadelmann, J; Tucker, R	2017	WoS	P2	unspecified	TRUE	Did not examine the intersection of at least two construct pillars
113	The Relationship Between Training Load and Injury in Men's Professional Basketball	Weiss, KJ; Allen, SV; McGuigan, MR; Whatman, CS	2017	WoS	P3	unspecified	FALSE	Did not examine the intersection of at least two construct pillars
115	The Mental Health of Elite Athletes: A Narrative Systematic Review	Rice, SM; Purcell, R; De Silva, S; Mawren, D; McGorry, PD; Parker, AG	2016	WoS	P3	unspecified	FALSE	Did not examine the intersection of at least two construct pillars
116	Predicting Self-Reported Illness for Professional Team-Sport Athletes	Thornton, HR; Delaney, JA; Duthie, GM; Scott, BR; Chivers, WJ; Sanctuary, CE; Dascombe, BJ	2016	WoS	P3	unspecified	FALSE	Did not examine the intersection of at least two construct pillars
117	Emotion-centered and action-centered coping in elite sport: Task execution design approach	Hanin, Y; Hanina, M; Sasek, H; Kobilsek, A	2016	WoS	P3	unspecified	TRUE	Did not examine the intersection of at least two construct pillars
118	A Longitudinal Case Study of a Sport Psychology Intervention Designed to Facilitate Team Culture Change	Glaude, M; Alexander-Urquhart, D; Henderson, S; Sheppard, MB; Loughead, TM; Bloom, GA	2026	WoS	P2	unspecified	TRUE	Elite/professional status not confirmed
119	Beyond the podium: A Delphi approach aimed at empowering coaches with practical strategies to identify and support athlete mental health	Sankey, C; Brockett, C; Slattery, K; Lederman, O; Warmenhoven, J; Menzies-Stegbauer, T; Langan, E; Caperchione, CM	2026	Scopus+WoS	P1, P2	unspecified	TRUE	Insufficient fit with the temporal cascade after eligibility assessment
120	Authentic leadership and well-being in sport: the mediating role of psychological safety and the moderating role of interpersonal violence	Hurst, P; Kavussanu, M	2026	WoS	P2	unspecified	FALSE	Elite/professional status not confirmed
121	Effects of a six-week basketball-specific individual skills development program on mental toughness and sport competition anxiety	Yazici, A; Tasçioglu, R; Solmaz, S; Sari, I; Kan, EMR; Bayköse, N	2026	WoS	P3	unspecified	TRUE	Did not examine the intersection of at least two construct pillars
122	Digital twin for Taekwondo athletes: integrating sports nutrition and psychological readiness using artificial intelligence	Amawi, AT; Grivas, GV; Alkasasbeh, WJ	2026	Scopus+WoS	P1	unspecified	FALSE	Did not examine the intersection of at least two construct pillars
123	Perilous games: Gendered moral disengagement, winning culture, and COVID-19 safety in NCAA division I athletics	Hollis, LP; Oaxaca, GC	2026	WoS	P3	unspecified	FALSE	Did not examine the intersection of at least two construct pillars
124	Sudden cardiac arrest preparedness in African football: evidence from Cameroon and implications for global sport	Tankeng, LT; Akwa, GM; Ossaih, AULS; Bilong, VAN; Tasong, LAM; Ghassi, HT; Ndom, MS; Douryang, M	2026	WoS	P1, P3	unspecified	TRUE	Insufficient fit with the temporal cascade after eligibility assessment
125	The role of leaders in the communal coping process in sports teams: A social network analysis perspective	Mahot, K; Lienhart, N; Doron, J	2026	WoS	P3	mixed	FALSE	Did not examine the intersection of at least two construct pillars
126	Implications of perceived empowering motivational climate on athletes' goal-regulatory processes: A longitudinal analysis	Martínez-González, N; Atienza, FL; Balaguer, I	2026	WoS	P1, P2	unspecified	TRUE	Elite/professional status not confirmed
127	Burnout Syndrome in Sport-Related Jobs: A Meta-analysis of Recent Evidence	Viego, V; Camilo, JAD; Santos, BIA; Lima-Sena, I; Hamodi, C	2026	WoS	P2	unspecified	TRUE	Did not examine the intersection of at least two construct pillars
129	How does need satisfaction mediate the relation between harmonious passion and positive affect in coaches and athletes? A dyadic longitudinal study in coach-athlete dyads	Denul, A; Haerens, L; Loeys, T	2026	WoS	P3	unspecified	TRUE	Elite/professional status not confirmed

130	The relationship between China university basketball association athletes' mindfulness, self-efficacy in sports training, and training competition satisfaction: a cross-sectional and longitudinal study	Zhao, YL; Liu, JW; Yang, XB; Wu, SM	2026	WoS	P3	unspecified	FALSE	Elite/professional status not confirmed
131	Psychometric properties of the French athlete version of the coach-athlete relationship questionnaire (Fr-CART-Q)	Roux, V; Trouilloud, D	2026	SPORTDiscus+WoS	P1	unspecified	TRUE	Elite/professional status not confirmed
132	Are winning teams more attentive to coach feedback and correction? A longitudinal study across a national-level volleyball season	Malisse, F; Van Puyenbroeck, S; Hofmans, J; Vande Broek, G	2026	WoS	P1	unspecified	TRUE	Elite/professional status not confirmed
133	Early Sport Specialization and Intense Training in Junior Tennis Players: A Sport-Specific Review	Thurber, L; Kantrowitz, DE; Wang, KC; Jayanthi, N; Colvin, A	2026	WoS	P2	unspecified	TRUE	Did not examine the intersection of at least two construct pillars
134	Guiding growth: the influence of coach-created climate on passion, athletic identity, and perfectionism in teen female athletes	Martin, EM; Horn, TS	2026	WoS	P1, P3	unspecified	TRUE	Elite/professional status not confirmed
135	From social identity to team performance: Linking transactive memory systems and teamwork	Ozlu, C; Sezer, U; Durdubas, D; Koruc, Z	2026	Scopus+WoS	P1	mixed	TRUE	Elite/professional status not confirmed
136	Perceived Coach and Teammate Weight/Shape-Related Beliefs and Disordered Eating in NCAA Swimmers	Pinson, CK; Fox, KR; Frank, GKW; Hooley, JM; Javaras, KN	2026	WoS	P1	unspecified	FALSE	Did not examine the intersection of at least two construct pillars
137	The Relationship Between the Somatosensory System and Growth and Maturation in Pathway Triathletes; a Pilot Study	Fitch, AS; Mara, J; Waddington, G	2026	WoS	P3	unspecified	FALSE	Did not examine the intersection of at least two construct pillars
138	Artificial intelligence in sport psychology: Implications for the identification and development of talent	Sartor, F; Rosito, SA; Raimo, M; Beato, M; Bertollo, M	2026	WoS	P2	unspecified	TRUE	Elite/professional status not confirmed
139	Developing Equestrian Athlete Confidence After Experiencing the Setback of a Fall	Collins, A; Smith, MJ	2026	WoS	P2	unspecified	TRUE	Elite/professional status not confirmed
140	Crossing the line: Players perspectives on the leap from junior to senior football	Tomás, J; Sousa, H; Araújo, D; Field, A; Ribeiro, J; Sarmento, H	2026	WoS	P2	unspecified	FALSE	Did not examine the intersection of at least two construct pillars
141	Active success drives momentum, but late-stage errors destroy it: a real-time analysis of volleyball	Asai, Y; Okuda, T; Shide, N; Kida, N	2026	WoS	P3	unspecified	TRUE	Elite/professional status not confirmed
142	An evaluation of the hot-hand hypothesis in the NBA when accounting for shot difficulty	Kondur, A; Shen, WN	2026	WoS	P1	mixed	FALSE	Elite/professional status not confirmed
143	Motivational Climate Collaboration Between a Collegiate Native American Volleyball Program and Sport Psychology Researchers	Claunch, JL; Fry, MD; Brown, TC	2026	WoS	P2	unspecified	FALSE	Elite/professional status not confirmed
144	The relationship between coaching leadership practices and athlete burnout among college athletes in China: the mediating role of psychological resilience and emotion regulation.	Chen, Xiaoying ; Yue, Yuan ; Xiang, Jun ; Zhang, Fang	2026	SPORTDiscus	P3	unspecified	FALSE	Elite/professional status not confirmed
146	My biggest learning curve: an interpretive phenomenological analysis of coaches' experiences of working with athletes who experience a menstrual cycle	Jorgensen, H; Davenport, MH; Kavic, K; McHugh, TL	2025	WoS	P1	unspecified	FALSE	Did not examine the intersection of at least two construct pillars
147	Impact of competitive anxiety and coach leadership style on track and field athlete performance: moderating role of coping strategies and emotional intelligence	Yue, Y; Zubing, X; Yasmin, F; Poulova, P	2025	SPORTDiscus+WoS	P2	mixed	FALSE	Did not examine the intersection of at least two construct pillars
148	Achieving international podium success with a positive sport experience	Willson, E; Kerr, G	2025	WoS	P2	unspecified	FALSE	Did not examine the intersection of at least two construct pillars
149	Relationship between growth mindset and competitive motivation: a moderated parallel mediation model and feature importance analysis	Deng, JC; Wang, ZP; Chen, HW; Song, B	2025	WoS	P1	mixed	FALSE	Did not examine the intersection of at least two construct pillars
150	Mental well-being in young Taekwondo athletes: psychological-related parameters, associated factors and cluster analysis	Tozetto, WR; Kons, RL; Bussato, E; Felix, G; Ishibashi, M; Milistetd, M	2025	WoS	P1, P2	unspecified	TRUE	Insufficient fit with the temporal cascade after eligibility assessment
151	Do coaches' and athletes' perceptions of relative energy deficiency in sport align? A narrative review	Delaney, D; McGuire, A; Warrington, G; Doyle, L	2025	WoS	P3	unspecified	FALSE	Did not examine the intersection of at least two construct pillars
152	Motivation, Sportspersonship, and Gender Equality in a Quidditch Sport Season: Analysis With Mixed Methodology	Méndez-Giménez, A; Collado-Martínez, M	2025	WoS	P3	unspecified	FALSE	Elite/professional status not confirmed
153	The impact of low energy availability risk on pre-competition physiological function in Chinese female combat athletes	Liang, YH; Li, YX; Chen, Y; Meng, K; Zhou, FY; Pei, YR; Liu, Y; Qiu, JQ	2025	Scopus+WoS	P2	mixed	FALSE	Did not examine the intersection of at least two construct pillars
154	Differences in the relationship among grit, self-regulation, competition preparation, and sport confidence across performance levels: a multi-group analysis	Shen, YQ; Sim, YK; Bae, J; Bang, IJ; Seo, JP	2025	Scopus+WoS	P2	unspecified	TRUE	Did not examine the intersection of at least two construct pillars
155	Weight loss practices in Chinese national and international-level Sanda athletes	Zhong, YM; Tang, WW; Gee, T; Li, MF; Jiang, HD; Yin, MY; Xu, K; Lakicevic, N; Drid, P; Kirk, C; Langan-Evans, C; Artioli, GG; Reale, R; Weldon, A; Li, YM	2025	Scopus+WoS	P2	unspecified	TRUE	Did not examine the intersection of at least two construct pillars
156	Using a Bayesian network to classify time to return to sport based on football injury epidemiological data	Yung, KKY; Wu, PPY; der Fünten, KAD; Hecksteden, A; Meyer, T	2025	Scopus+WoS	P1	unspecified	TRUE	Did not examine the intersection of at least two construct pillars
157	Relative age effect, morphological status and physical performance of U-15 Croatian football players	Rada, A; Marasovic, J; Erceg, M; Krustrup, P; Randers, MB; Ardigò, LP	2025	Scopus+WoS	P3	mixed	FALSE	Did not examine the intersection of at least two construct pillars
158	From adversity to adaptation: the struggle between resilience and athlete burnout in stressful situations	Cai, CY; Mei, ZY; Yang, Y; Luo, S	2025	WoS	P1	mixed	FALSE	Elite/professional status not confirmed
159	The relationship between self-oriented perfectionism and athlete burnout: a longitudinal study	Fu, YT; Li, XY; Sun, JJ; Li, CX; Peng, Y; Hong, FX; Pan, JH	2025	WoS	P3	unspecified	FALSE	Elite/professional status not confirmed
160	A systematic review of the factors associated with athlete burnout in team sports	Woods, S; Dunne, S; Gallagher, P; McNicholl, A	2025	WoS	P1	unspecified	TRUE	Elite/professional status not confirmed

161	Coach-athlete attachment and athlete burnout: testing longitudinal mediation via cognitive reappraisal and expressive suppression	Xin, ZY; Li, TE; Li, QL; Chen, YA; Wang, MH	2025	SPORTDiscus+WoS	P3	mixed	TRUE	Elite/professional status not confirmed
162	Longitudinal associations between coach-athlete relationship quality and individual injury rate among young handball players: A variable and person-centered approach in intensive training centers	Roux, V; Trouilloud, D	2025	WoS	P3	unspecified	FALSE	Elite/professional status not confirmed
163	Canadian University Sport Coaches' Experiences Participating in a Social Learning Space on Topics Related to and Impacting Coach Mental Health	Henderson, S; Bloom, GA; Durand-Bush, N; Kenttä, G	2025	WoS	P1	unspecified	FALSE	Elite/professional status not confirmed
164	The relation between perceived need-supportive coaching behaviours, winning games, and team resilience: a longitudinal examination among basketball and soccer teams	Van Puyenbroeck, S; Hermans, F; Boen, F; Fransen, K; Vande Broek, G	2025	WoS	P2	mixed	TRUE	Elite/professional status not confirmed
165	The threshold moderating effect of self-efficacy: a nonlinear transformation mechanism of work passion on job satisfaction among sports practitioners	Wang, KY; Tan, M	2025	WoS	P1	unspecified	TRUE	Elite/professional status not confirmed
166	The motivational climate perceived by young soccer players regarding their coaches, parents, and peers on sport optimal functioning: a cluster analysis	Martínez-González, N; Atienza-González, FL; González-García, L; Balaguer, I	2025	WoS	P1	mixed	TRUE	Elite/professional status not confirmed
167	Resilience and Well-Being Over the Course of an National Collegiate Athletic Association Division I Gymnastics Season	Williams, SM; Baez, SE; Derrico, D; Robinson, H; Coyne, K; Settle, H; Jochimsen, KN	2025	WoS	P1	unspecified	FALSE	Did not examine the intersection of at least two construct pillars
168	Smartphone use and mental health - findings from a 30-day longitudinal study among young talented volleyball players	Hilpisch, C; Zentgraf, K; Mutz, M	2025	WoS	P1	unspecified	TRUE	Elite/professional status not confirmed
169	Fostering self-efficacy in Chinese university athletes: the mediating roles of psychological resilience and emotional regulation and the moderating role of autonomy-supportive coaching	Zhang, X; Khan, SU	2025	SPORTDiscus+WoS	P1	unspecified	FALSE	Elite/professional status not confirmed
170	Structural modelling of student volleyball athletes' intimacy, social adjustment, perceived stress, and learning-related anxiety: mediating role of psychological safety	Li, YS	2025	Scopus+WoS	P1	unspecified	TRUE	Elite/professional status not confirmed
171	Ethnographic research on coaching behaviour to enhance athlete skill learning: A scoping review	Taylor, S; Renshaw, I; Pinder, R; Polman, R; Russell, S; Gorman, AD	2025	WoS	P3	unspecified	TRUE	Elite/professional status not confirmed
418	Assessing toxic leadership in high-performance sports: Psychometric validation of a Swedish Version of the Toxic Leadership Scale for Sports (TLS- S)	Lundqvist C.,Johansson S.	2026	Scopus	P1, P2	elite	FALSE	No direct or phase-relevant temporal content
419	Acceptable or Not: An In-depth Analysis of Adolescent Competitive Athletes' Perceptions on Abusive Coaching Behaviors	Marsollier E.,Hauw D.,von Roten F.C.	2025	Scopus	P1	mixed/unclear	FALSE	Elite/professional status not confirmed
420	Interpersonal climate and moral conduct in competitive sport: A dual-route model with serial mediation	Wang Z.,Liu T.,Hong S.,Wang W.,Wang F.	2025	Scopus	P1, P2, P3	elite	TRUE	Insufficient fit with the temporal cascade after eligibility assessment
422	Using the Conceptual Framework for Examining Sport Teams to Understand Group Dynamics in Professional Soccer	Gonzalez-Ponce I.,Diaz-Garcia J.,Ponce-Bordon J.C.,Jimenez-Castuera R.,Lopez-Gajardo M.A.	2022	Scopus	P1	elite	FALSE	Did not examine the intersection of at least two construct pillars
423	Examining the psychometric properties of the Controlling Coach Behaviors Scale in Chinese Elite Athletes	Zhao D.,Zhou Y.	2022	Scopus	P1, P3	elite	FALSE	No direct or phase-relevant temporal content
429	Emotion, psychological well-being and their influence on resilience. A study with semi-professional athletes	Trigueros R.,Aguilar-Parra J.M.,Alvarez J.F.,Gonzalez-Bernal J.J.,Lopez-Liria R.	2019	Scopus	P1, P2, P3	elite	TRUE	Insufficient fit with the temporal cascade after eligibility assessment
431	Using vignettes to analyze potential influences of effective strength and conditioning coaching on athlete development	Szedlak C.,Smith M.,Day M.,Callary B.	2018	Scopus	P1, P3	elite	FALSE	No direct or phase-relevant temporal content
433	Influences of perfectionism and motivational climate on attitudes towards doping among Korean national athletes: A cross sectional study	Bae M.,Yoon J.,Kang H.,Kim T.	2017	Scopus	P1, P3	elite	FALSE	No direct or phase-relevant temporal content
434	Pathos and orchestration in elite sport: The experiences of NCAA DI student- athletes	Raabe J.,Readdy T.,Zakrajsek R.A.	2017	Scopus	P1, P2, P3	elite	FALSE	No direct or phase-relevant temporal content
435	'This might be him; the guy I'm gonna marry': Love and sexual relationships between female elite-athletes and male coaches	Johansson S.,Larsson H.	2017	Scopus	P1, P2, P3	elite	FALSE	No direct or phase-relevant temporal content
438	Training Stress, Sleep Behaviour, and Recovery Self-Regulation in Elite Female Boxers: A Behavioural Pattern Analysis Using Data-Driven Methods	Rattanasateankij W.,Senakham T.,Punthipayanon S.,Puttasimma T.,Senakham N.	2025	Scopus	P1, P3	elite	TRUE	Insufficient fit with the temporal cascade after eligibility assessment
439	Strategies to reduce hyperglycemia-related anxiety in elite athletes with type 1 diabetes: A qualitative analysis	Katz A.,Shulkin A.,Fortier M.-A.,... Rabasa-Lhoret R.,Brazeau A.-S.	2025	Scopus	P2, P3	elite	TRUE	Insufficient fit with the temporal cascade after eligibility assessment
440	Sport Anxiety, Fear of Negative Evaluation, Stress and Coping as Predictors of Athlete's Sensitivity to the Behavior of Supporters	Gabrys K.,Wontorczyk A.	2023	Scopus	P1, P2, P3	elite	FALSE	No direct or phase-relevant temporal content
441	Depressive symptoms and burnout in football players: A systematic review	Sarmento H.,Frontini R.,Marques A.,... Campos M.J.,Clemente F.M.	2021	Scopus	P1, P3	elite	TRUE	Measurement or review paper not retained in the empirical synthesis
444	The development of mental toughness: A journey into the perceptions and experiences of a world-class padel athlete	Barreiros A.,Maia M.,Fonseca A.M.,Dias C.	2026	Scopus	P1, P2, P3	elite	TRUE	Insufficient fit with the temporal cascade after eligibility assessment
466	Facilitating Positive Youth Development in Elite Youth Hockey: Exploring Coaches' Capabilities, Opportunities, and Motivations	Preston C.,Allan V.,Fraser-Thomas J.	2021	Scopus	P1, P3	elite	TRUE	Insufficient fit with the temporal cascade after eligibility assessment

467	Athletes' Perception of Leadership According to Their Perceptions of Goal Achievement and Sport Results	Gomes A.R.,Almeida A.,Resende R.	2020	Scopus	P1	mixed/unclear	TRUE	Elite/professional status not confirmed
468	What does it mean to be "mentally tough" as a NCAA division I collegiate coach?	Weinberg R.,Freysinger V.,Vealey R.,Block C.	2020	Scopus	P1, P3	elite	TRUE	Insufficient fit with the temporal cascade after eligibility assessment
470	The Optimal Team Functioning Model: A Grounded Theory Framework to Guide Teamwork in Curling	Collins J.,Durand-Bush N.	2019	Scopus	P1, P2	mixed/unclear	TRUE	Elite/professional status not confirmed
480	A longitudinal examination of stressors, appraisals, and coping in youth swimming	Hayward F.P.I.,Knight C.J.,Mellalieu S.D.	2017	Scopus	P1, P2, P3	mixed/unclear	TRUE	Elite/professional status not confirmed

Operational Definitions and Coding Rules

Code	Operational definition	Direct evidence threshold	Examples / cautions
Phase I: Foundation building	Pre-season or early-season period in which climate, expectations, selection, roles, and initial relational norms are established.	Data collected before the first competitive block, at baseline explicitly identified as early season, or findings explicitly concerning selection/initial formation.	Do not place general environment-design studies here unless the paper explicitly links them to season opening; otherwise code as indirect.
Phase II: Competitive maintenance	Regular-season period between initial formation and championship/major-event preparation; includes day-to-day feedback, autonomy calibration, conflict, fatigue, and motivational maintenance.	Repeated data during the active season, daily diary data, or explicit mid-season/regular-season assessment.	Cross-sectional studies conducted 'during a season' are indirect unless timing is analytically relevant.
Phase III: Peak competition	Immediate precompetition, competition, tournament, championship, or major-event period, including within-event appraisal and communication.	Measurement before/during/after a defined competition or qualitative evidence explicitly focused on major-event pressure.	Statistical mediation is described as an indirect association, not proof of a causal mechanism.
Phase IV: Post-competition/off-season transition	Period after the final event or season, including debriefing, recovery, relationship evaluation, continuation/dissolution, and preparation for the next cycle.	Data explicitly collected after season end or focused on off-season/post-cycle processes.	Emotional abuse, career-long effects, and deselection are not automatically Phase IV; code as relational aftermath unless season-end timing is explicit.
Relational aftermath	Longer-term residue, rupture, reappraisal, or career carryover that is not tied to a defined post-season window.	Retrospective or longitudinal evidence on effects after a relationship/event.	Kept separate from Phase IV to avoid temporal misclassification.
Cross-cutting	Organizational, cultural, gender, sport-structure, or person-level factors that operate across phases.	Evidence not confined to one phase but directly relevant to how the cascade differs.	Do not force phase placement.
Core study	Clearly eligible elite/high-performance sample and examines at least two of P1, P2, and P3.	Meets population, construct-integration, and phase relevance criteria.	Core studies carry the main inferential weight.
Contextual study	Eligible elite/high-performance evidence that informs one track, moderator, or theoretical boundary but does not integrate at least two pillars.	Used to support context or within-track temporal dynamics.	Reported separately from core studies.
Direct phase evidence	Timing is measured or explicitly described in the study design.	Prospective, repeated, diary, event-based, or explicit phase-specific qualitative evidence.	Shown as empirical support.
Indirect phase evidence	The finding is compatible with a phase but the study did not directly test that timing.	Cross-sectional or retrospective evidence assigned by conceptual fit.	Shown as interpretive placement, not a finding about change.
