# Supplementary material for: Coach behaviors, coach–athlete relationships, and competitive psychological processes in elite sport: a temporal narrative synthesis
Source: Front Psychol. 2026 Sep 29;17:1969318. doi: 10.3389/fpsyg.2026.1969318 (PMC13658106; doi:10.3389/fpsyg.2026.1969318)
Supplement: Supplementary file 2 [file Supplementary_file_1.DOCX]

Figure 1. Flow of records through identification, title/abstract screening, eligibility assessment, and inclusion, following PRISMA 2020 reporting guidance (Page et al., 2021).

Figure 2. Temporal synthesis framework for coach behaviors, relationship quality, and competitive psychological processes across an elite competitive cycle. The columns organize observed findings and testable propositions by phase; horizontal arrows indicate temporal progression, not causal carryover. Direct empirical support is strongest during competitive maintenance and is considerable but mixed around peak competition. Foundation-building evidence is limited, and post-season transition remains an empirical gap. Relational aftermath and injury-related disruption are shown separately because they do not constitute direct evidence for Phase IV.
