# Supplementary material for: Coach behaviors, coach–athlete relationships, and competitive psychological processes in elite sport: a temporal narrative synthesis
Source: Front Psychol. 2026 Sep 29;17:1969318. doi: 10.3389/fpsyg.2026.1969318 (PMC13658106; doi:10.3389/fpsyg.2026.1969318)
Supplement: Supplementary file 3 [file Table_2.DOCX]

**Supplementary Materials**

**Coach Behaviors, Coach–Athlete Relationships, and Competitive Psychological Processes in Elite Sport: A Temporal Narrative Synthesis**

**Supplementary material map**

| **Item** | **Content** | **Location / format** |
| --- | --- | --- |
| **Supplementary Table S1** | Measurement instruments relevant to the three construct pillars | This document |
| **Supplementary Table S2** | Full Boolean search strings, field codes, filters, and database yields | This document |
| **Supplementary Table S3** | Operational definitions for seasonal phase and evidence coding | This document |
| **Supplementary Table S4** | Study-level evidence-weight appraisal for all 30 included studies | This document |
| **Supplementary Data File 1** | Final master, deduplication log, screening decisions, 175 article-level eligibility exclusions, included-study matrix, coding guide, and evidence-weight ratings | Separate Excel workbook; repository DOI: 10.6084/m9.figshare.32781993 |

*All citations in the supplementary tables refer to the reference list in the main manuscript. Supplementary Data File 1 is supplied separately and is not embedded in this document.*

**Supplementary Table S1**. *Measurement Instruments Relevant to the Review’s Three Construct Pillars*

| **Instrument** | **Abbreviation** | **Construct(s) measured** | **Source** |
| --- | --- | --- | --- |
| **Pillar 1: Coach Behaviors and Leadership** | | | |
| Coaching Behavior Assessment System | CBAS | Observable coaching behaviors in training and competition settings; 12 behavioral categories across reactive and spontaneous dimensions. | Smith et al., 1977 |
| Leadership Scale for Sport | LSS | Athlete perceptions of coaching leadership across five dimensions: training and instruction, democratic behavior, autocratic behavior, social support, and positive feedback. | Chelladurai & Saleh, 1980 |
| Controlling Coach Behaviors Scale | CCBS | Athlete perceptions of a controlling interpersonal style among coaches; four subscales: controlling use of rewards, negative conditional regard, intimidation, and excessive personal control. | Bartholomew et al., 2010 |
| Perceived Autonomy Support Scale for Exercise Settings | PASSES | Athlete perceptions of autonomy support from coaches; adapted from the Health Care Climate Questionnaire for sport and exercise settings. | Hagger et al., 2007 |
| **Pillar 2: Coach–Athlete Relationship Quality** | | | |
| Coach–Athlete Relationship Questionnaire | CART-Q | Dyadic relationship quality through closeness (affective bonds), commitment (intention to maintain the relationship), and complementarity (cooperative transactions); athlete and coach versions are available. | Jowett & Ntoumanis, 2004 |
| **Pillar 3: Competitive Psychological Processes** | | | |
| Competitive State Anxiety Inventory-2 Revised | CSAI-2R | Precompetition cognitive anxiety, somatic anxiety, and self-confidence; a state-based measure designed for administration before competitive events. | Cox et al., 2003 |
| Sport Anxiety Scale-2 | SAS-2 | Trait sport anxiety across three dimensions: somatic anxiety, worry, and concentration disruption. | Smith et al., 2006 |
| Coping Inventory for Competitive Sport | CICS | Coping strategies deployed during competition; 10 strategies across task-oriented, distraction-oriented, and disengagement-oriented dimensions. | Gaudreau & Blondin, 2002 |
| Test of Performance Strategies | TOPS | Psychological performance strategies in practice and competition; subscales include self-talk, emotional control, automaticity, goal setting, imagery, activation, relaxation, and negative thinking. | Thomas et al., 1999 |
| Flow State Scale-2 | FSS-2 | Flow experience during physical activity; nine dimensions including challenge–skill balance, action–awareness merging, and loss of self-consciousness. | Jackson & Eklund, 2002 |
| Mental Toughness Questionnaire 48 | MTQ48 | Mental toughness across four components: control (life and emotional), commitment, challenge, and confidence (in abilities and interpersonal functioning). | Clough et al., 2002 |
| **Cross-Cutting Measures: Motivation and Climate** | | | |
| Perceived Motivational Climate in Sport Questionnaire-2 | PMCSQ-2 | Athlete perceptions of mastery-oriented and performance-oriented motivational climates created by the coach. | Newton et al., 2000 |
| Sport Motivation Scale-II | SMS-II | Intrinsic motivation, extrinsic motivation (identified, introjected, and external regulation), and amotivation in sport; a revised version with improved factorial structure. | Pelletier et al., 2013 |
| Athlete Burnout Questionnaire | ABQ | Athlete burnout through three subscales: emotional and physical exhaustion, reduced sense of accomplishment, and sport devaluation. | Raedeke & Smith, 2001 |

*Note.* Instruments are organized by their primary construct pillar. Several instruments span more than one pillar in practice; placement reflects their dominant measurement function. The table is a reference to measures relevant to the review domains and does not imply that every instrument was used in every included study. CBAS = Coaching Behavior Assessment System; LSS = Leadership Scale for Sport; CCBS = Controlling Coach Behaviors Scale; PASSES = Perceived Autonomy Support Scale for Exercise Settings; CART-Q = Coach–Athlete Relationship Questionnaire; CSAI-2R = Competitive State Anxiety Inventory-2 Revised; SAS-2 = Sport Anxiety Scale-2; CICS = Coping Inventory for Competitive Sport; TOPS = Test of Performance Strategies; FSS-2 = Flow State Scale-2; MTQ48 = Mental Toughness Questionnaire 48; PMCSQ-2 = Perceived Motivational Climate in Sport Questionnaire-2; SMS-II = Sport Motivation Scale-II; ABQ = Athlete Burnout Questionnaire.

**Supplementary Table S2**. *Full Boolean Search Strings, Field Codes, Filters, and Yields by Database*

| **Database** | **String** | **Full Boolean search query** | **Filters and yield** |
| --- | --- | --- | --- |
| Web of Science | S1: Coaching Dynamics × Elite Sport | TS=("coach* behav*" OR "coaching style*" OR "coach-athlete relationship*" OR "athlete-coach relationship*" OR "motivational climate" OR "autonomy support" OR "coach* leadership") AND TS=("elite athlet*" OR "professional athlet*" OR "professional sport*" OR "high-performance sport*" OR "competitive athlet*" OR "Olympic" OR "national team") AND TS=("psycholog*" OR "motivation" OR "well-being" OR "wellbeing" OR "performance" OR "anxiety" OR "self-regulation") | Initial: 202. Refined to 2016–2026; Article/Review Article; SSCI/SCI-Expanded; English. Result: 96. Further refined by subject categories (Sport Sciences, Psychology Applied, Psychology Social, Psychology Multidisciplinary, and related). Final: 81. |
| Web of Science | S2: Competitive Psychological Processes × Coaching | TS=("competi* anxiety" OR "pre-competition" OR "competitive stress" OR "competitive pressure" OR "choking under pressure" OR "clutch performance" OR "competition psycholog*" OR "sport anxiety") AND TS=("coach*" OR "mentor" OR "leadership" OR "athlete-coach" OR "interpersonal") AND TS=("elite" OR "professional" OR "high-performance" OR "high performance" OR "national level" OR "international level") | Initial: 84. Refined to 2016–2026; Article/Review Article; SSCI/SCI-Expanded; English. Final: 43. |
| Web of Science | S3: Temporal/Seasonal Dynamics | TS=("coach*" AND "athlet*") AND TS=("season*" OR "longitudinal" OR "within-season" OR "pre-season" OR "in-season" OR "temporal" OR "over time" OR "across time") AND TS=("psycholog*" OR "relationship" OR "motivation" OR "burnout" OR "well-being") | Initial: 603. Refined to 2016–2026; Article/Review Article; SSCI/SCI-Expanded; English. Result: 342. Further refined by subject categories as in S1. Final: 283. |
| SPORTDiscus via EBSCOhost | S1: Coaching Dynamics × Elite Sport | ("coach* behav*" OR "coaching style*" OR "coach-athlete relationship*" OR "athlete-coach relationship*" OR "motivational climate" OR "autonomy support" OR "coach* leadership") AND ("elite athlet*" OR "professional athlet*" OR "professional sport*" OR "high-performance sport*" OR "competitive athlet*" OR "Olympic" OR "national team") AND ("psycholog*" OR "motivation" OR "well-being" OR "wellbeing" OR "performance" OR "anxiety" OR "self-regulation") | Default search fields (title, abstract, and subject terms); English; peer reviewed; publication date 2016–2026. Final: 22. |
| SPORTDiscus via EBSCOhost | S2: Competitive Psychological Processes × Coaching | ("competi* anxiety" OR "pre-competition" OR "competitive stress" OR "competitive pressure" OR "choking under pressure" OR "clutch performance" OR "competition psycholog*" OR "sport anxiety") AND ("coach*" OR "mentor" OR "leadership" OR "athlete-coach" OR "interpersonal") AND ("elite" OR "professional" OR "high-performance" OR "high performance" OR "national level" OR "international level") | Default search fields (title, abstract, and subject terms); English; peer reviewed; publication date 2016–2026. Final: 7. |
| SPORTDiscus via EBSCOhost | S3: Temporal/Seasonal Dynamics | ("coach*" AND "athlet*") AND ("season*" OR "longitudinal" OR "within-season" OR "pre-season" OR "in-season" OR "temporal" OR "over time" OR "across time") AND ("psycholog*" OR "relationship" OR "motivation" OR "burnout" OR "well-being") | Default search fields (title, abstract, and subject terms); English; peer reviewed; publication date 2016–2026. Final: 46. |
| Scopus | S1: Coaching Dynamics × Elite Sport | TITLE-ABS-KEY(("coach* behav*" OR "coaching style*" OR "coach-athlete relationship*" OR "athlete-coach relationship*" OR "motivational climate" OR "autonomy support" OR "coach* leadership")) AND TITLE-ABS-KEY(("elite athlet*" OR "professional athlet*" OR "professional sport*" OR "high-performance sport*" OR "competitive athlet*" OR "Olympic" OR "national team")) AND TITLE-ABS-KEY(("psycholog*" OR "motivation" OR "well-being" OR "wellbeing" OR "performance" OR "anxiety" OR "self-regulation")) AND PUBYEAR > 2015 AND PUBYEAR < 2027 | Initial: 120. Filtered by Article/Review; English; subject areas: Medicine, Psychology, Health Professions, Social Sciences, and Multidisciplinary. Final: 39. |
| Scopus | S2: Competitive Psychological Processes × Coaching | TITLE-ABS-KEY(("competi* anxiety" OR "pre-competition" OR "competitive stress" OR "competitive pressure" OR "choking under pressure" OR "clutch performance" OR "competition psycholog*" OR "sport anxiety")) AND TITLE-ABS-KEY(("coach*" OR "mentor" OR "leadership" OR "athlete-coach" OR "interpersonal")) AND TITLE-ABS-KEY(("elite" OR "professional" OR "high-performance" OR "high performance" OR "national level" OR "international level")) AND (LIMIT-TO(DOCTYPE, "ar") OR LIMIT-TO(DOCTYPE, "re")) AND LIMIT-TO(LANGUAGE, "English") | Initial: 116. Filtered by Article/Review; English; 2016–2026. Final: 29. |
| Scopus | S3: Temporal/Seasonal Dynamics | TITLE-ABS-KEY(("coach*" AND "athlet*")) AND TITLE-ABS-KEY(("season*" OR "longitudinal" OR "within-season" OR "pre-season" OR "in-season" OR "temporal" OR "over time" OR "across time")) AND TITLE-ABS-KEY(("psycholog*" OR "relationship" OR "motivation" OR "burnout" OR "well-being" OR "wellbeing")) AND (LIMIT-TO(DOCTYPE, "ar") OR LIMIT-TO(DOCTYPE, "re")) AND LIMIT-TO(LANGUAGE, "English") AND LIMIT-TO(EXACTKEYWORD, "Human") AND LIMIT-TO(PUBSTAGE, "final") AND (LIMIT-TO(SUBJAREA, "PSYC") OR LIMIT-TO(SUBJAREA, "SOCI") OR LIMIT-TO(SUBJAREA, "MULT")) | Initial: 854. Filtered by 2016–2026; Article/Review; English. Result: 319. Further filtered by Human keyword, final publication stage, and Psychology, Social Sciences, or Multidisciplinary subject areas. Final: 95. |

*Note.* All searches were conducted on 22 June 2026. Asterisks (*) indicate truncation. TS = Topic field in Web of Science; TITLE-ABS-KEY = Title, Abstract, and Keywords field in Scopus. S1 = String 1; S2 = String 2; S3 = String 3. The searches yielded 645 records: 407 from Web of Science, 75 from SPORTDiscus, and 163 from Scopus. Five duplicates occurred within Scopus, leaving 158 unique Scopus records. Ninety-four Scopus records overlapped with the 417-record Web of Science/SPORTDiscus master, and 64 were Scopus-only. The final three-database master contained 481 unique records; 164 duplicates were removed in total.

**Supplementary Table S3**

*Operational Definitions for Seasonal Phase and Evidence Coding*

| **Code** | **Operational definition** | **Direct evidence threshold** | **Boundary rule** |
| --- | --- | --- | --- |
| Phase I: Foundation building | Pre-season or early-season period in which climate, expectations, selection, roles, and initial relational norms are established. | Data collected before the first competitive block, at baseline explicitly identified as early season, or findings explicitly concerning selection or initial formation. | Do not place general environment-design studies here unless the paper explicitly links them to season opening; otherwise code as indirect. |
| Phase II: Competitive maintenance | Regular-season period between initial formation and championship or major-event preparation; includes day-to-day feedback, autonomy calibration, conflict, fatigue, and motivational maintenance. | Repeated data during the active season, daily diary data, or explicit mid-season or regular-season assessment. | Cross-sectional studies conducted during a season are indirect unless timing is analytically relevant. |
| Phase III: Peak competition | Immediate precompetition, competition, tournament, championship, or major-event period, including within-event appraisal and communication. | Measurement before, during, or after a defined competition, or qualitative evidence explicitly focused on major-event pressure. | Statistical mediation is described as an indirect association, not proof of a causal mechanism. |
| Phase IV: Post-competition/off-season transition | Period after the final event or season, including debriefing, recovery, relationship evaluation, continuation or dissolution, and preparation for the next cycle. | Data explicitly collected after season end or focused on off-season or post-cycle processes. | Emotional abuse, career-long effects, and deselection are not automatically Phase IV; code as relational aftermath unless season-end timing is explicit. |
| Relational aftermath | Longer-term residue, rupture, reappraisal, or career carryover not tied to a defined post-season window. | Retrospective or longitudinal evidence concerning effects after a relationship or event. | Kept separate from Phase IV to avoid temporal misclassification. |
| Cross-cutting | Organizational, cultural, gender-related, sport-structure, or person-level factors that operate across phases. | Evidence not confined to one phase but directly relevant to how the cascade differs. | Do not force phase placement. |
| Core study | Clearly eligible elite or high-performance sample examining at least two of P1, P2, and P3. | Meets population, construct-integration, and phase-relevance criteria. | Core studies carry the main inferential weight. |
| Contextual study | Eligible elite or high-performance evidence that informs one track, moderator, or theoretical boundary but does not integrate at least two pillars. | Used to support context or within-track temporal dynamics. | Reported separately from core studies. |
| Direct phase evidence | Timing is measured or explicitly described in the study design. | Prospective, repeated, diary, event-based, or explicit phase-specific qualitative evidence. | Shown as empirical support. |
| Indirect phase evidence | The finding is compatible with a phase, but the study did not directly test that timing. | Cross-sectional or retrospective evidence assigned by conceptual fit. | Shown as interpretive placement, not a finding about change. |

*Note.* P1 = coach behavior or leadership; P2 = coach–athlete relationship or directly relevant interpersonal processes; P3 = competitive psychological processes. Phase evidence was coded as direct only when timing was measured or explicitly described; conceptual compatibility alone was coded as indirect.

**Supplementary Table S4**

*Evidence-Weight Appraisal of Included Studies*

| **Study** | **Tier** | **Population relevance** | **Construct integration** | **Temporal alignment** | **Design strength** | **Reporting adequacy** | **Total** | **Weight** |
| --- | --- | --- | --- | --- | --- | --- | --- | --- |
| Fryer et al., 2026 | Core | 2 | 1 | 2 | 1 | 2 | 8 | High |
| Ferguson & Mason, 2026 | Core | 2 | 1 | 1 | 1 | 2 | 7 | Moderate |
| Braga-Pereira et al., 2026 | Core | 2 | 1 | 2 | 1 | 2 | 8 | High |
| Davis et al., 2026 | Core | 1 | 1 | 2 | 2 | 2 | 8 | High |
| Henriksen et al., 2025 | Core | 2 | 1 | 1 | 1 | 2 | 7 | Moderate |
| Ulaş et al., 2025 | Core | 2 | 2 | 1 | 1 | 2 | 8 | High |
| González-García et al., 2024 | Core | 1 | 1 | 2 | 2 | 2 | 8 | High |
| Karayel et al., 2024 | Core | 2 | 2 | 1 | 1 | 2 | 8 | High |
| Slade et al., 2024 | Core | 2 | 1 | 1 | 1 | 2 | 7 | Moderate |
| Cece et al., 2022 | Core | 2 | 1 | 2 | 2 | 2 | 9 | High |
| Robazza et al., 2022 | Core | 1 | 1 | 1 | 1 | 2 | 6 | Moderate |
| da Silva et al., 2022 | Core | 2 | 1 | 2 | 2 | 2 | 9 | High |
| Kim & Park, 2020 | Core | 2 | 2 | 2 | 1 | 2 | 9 | High |
| Moreira da Silva et al., 2021 | Core | 2 | 1 | 1 | 1 | 2 | 7 | Moderate |
| Miller et al., 2020 | Core | 2 | 2 | 2 | 2 | 2 | 10 | High |
| Balk et al., 2019 | Core | 2 | 1 | 2 | 2 | 2 | 9 | High |
| Cheval et al., 2017 | Core | 2 | 1 | 2 | 2 | 2 | 9 | High |
| Nicholls et al., 2017 | Core | 2 | 1 | 2 | 2 | 2 | 9 | High |
| Ntoumanis et al., 2017 | Core | 1 | 1 | 2 | 2 | 2 | 8 | High |
| Kavanagh et al., 2017 | Core | 2 | 1 | 1 | 1 | 2 | 7 | Moderate |
| De Backer et al., 2018 | Core | 2 | 1 | 2 | 2 | 2 | 9 | High |
| Schlawe et al., 2026 | Contextual | 2 | 1 | 1 | 1 | 2 | 7 | Moderate |
| Tjønndal & Haugen, 2024 | Contextual | 2 | 1 | 1 | 1 | 2 | 7 | Moderate |
| Levillain et al., 2025 | Contextual | 1 | 0 | 2 | 2 | 2 | 7 | Moderate |
| Levillain et al., 2022 | Contextual | 1 | 0 | 2 | 2 | 2 | 7 | Moderate |
| Verner-Filion & Vallerand, 2018 | Contextual | 2 | 0 | 2 | 2 | 2 | 8 | High |
| Dehghansai et al., 2020 | Contextual | 2 | 0 | 1 | 1 | 2 | 6 | Moderate |
| Poucher et al., 2018 | Contextual | 2 | 0 | 1 | 1 | 2 | 6 | Moderate |
| Kegelaers & Wylleman, 2019 | Contextual | 2 | 1 | 1 | 1 | 2 | 7 | Moderate |
| Carleton et al., 2016 | Contextual | 2 | 1 | 2 | 2 | 2 | 9 | High |

*Note.* Each domain was scored from 0 to 2. Total scores of 8–10 were classified as high evidence weight and scores of 5–7 as moderate. The appraisal combines methodological strength with relevance to the temporal synthesis and is not a conventional risk-of-bias instrument. The 30-study set comprised 21 core and nine contextual studies; 17 studies were rated high and 13 moderate.
